# Supplementary material for: Tight junction protein LSR is a host defense factor against SARS-CoV-2 infection in the small intestine
Source: EMBO J. 2024 Oct 23;43(23):6124–51. doi: 10.1038/s44318-024-00281-4 (PMC11612383; doi:10.1038/s44318-024-00281-4)
Supplement: Supplementary file 1 — Table EV1 [file 44318_2024_281_MOESM1_ESM.pdf]

Table EV1: primary antibody list

Target	Product name	Host	Applications	Cat. NO.	Company
LSR	LSR Purified MaxPab mouse Polyclonal Antibody (B01P)	Mouse	IF, WB	H00051599-B01P	Abnova
LSR	LSR (D3E3N) XP Rabbit mAb	Rabbit	CoIP	#14804	Cell Signaling Technology
LSR	LSR / LISCH7 Rabbit anti-Mouse Polyclonal (aa35-205) Antibody	Rabbit	WB, IF	LS-C373180	LifeSpan Biosciences
LSR	LSR Polyclonal Antibody	Rabbit	IF	PA5-52412	Invitrogen
ACE2	ACE2 Recombinant Rabbit Monoclonal Antibody (SN0754)	Rabbit	IF	MA5-32307	Invitrogen
ACE2	ACE2 Polyclonal antibody	Rabbit	CoIP, WB	21115-1-AP	Proteintech
Spike	Rabbit polyclonal to SARS-CoV-2 spike glycoprotein - Coronavirus	Rabbit	IF	ab272504	Abcam
Spike	SARS-CoV-2 Spike Protein (S1) (E5S3V) Rabbit mAb	Rabbit	CoIP, WB	#99423	Cell Signaling Technology
Nucleocapsid protein	SARS Nucleocapsid Protein Antibody	Rabbit	IF	NB100-56576	Novus Biologicals
Flag	DYKDDDDK Tag Antibody	Rabbit	IF, CoIP	#2368	Cell Signaling Technology
Flag	DYKDDDDK tag Monoclonal antibody (Binds	Mouse	CoIP	66008-4-Ig	Proteintech

	to FLAG® tag epitope)				
Sodium Potassium ATPase	Anti-Sodium Potassium ATPase [EP1845Y] - Plasma Loading Control	Rabbit	WB	Ab76020	Abcam
LY6G	Purified anti-mouse Ly-6G Antibody (1A8)	Mouse	IF	127602	Biolegend
β-actin	Anti-beta Actin Antibody - [mAbcam 8226] Loading Control	Mouse	WB	ab8226	Abcam
