# Supplementary material for: Tight junction protein LSR is a host defense factor against SARS-CoV-2 infection in the small intestine
Source: EMBO J. 2024 Oct 23;43(23):6124–51. doi: 10.1038/s44318-024-00281-4 (PMC11612383; doi:10.1038/s44318-024-00281-4)
Supplement: Supplementary file 2 — Table EV2 [file 44318_2024_281_MOESM2_ESM.pdf]

**Table EV2: Reagents list**

| Product name | Cat. NO. | Company |
|---|---|---|
| DMEM | 2320136 | vivacell biosciences |
| FBS | 10099141C | Gibco |
| Penicillin/Streptomycin Solution | MA0110 | Meilunbio |
| Puromycin | HY-B1743A | MCE |
| Q5® Site-Directed Mutagenesis Kit | E0554S | NEB |
| Matrigel | 354277 | Corning |
| mTesR1 | 85850 | STEMCELL |
| DMEM/F12 | 36254 | STEMCELL |
| Trypsin-EDTA Solution | E607002 | Sangon Biotech |
| Dispase | 17105041 | Invitrogen |
| Lipofectamine 3000 | L3000015 | Invitrogen |
| STEMdiff™ Intestinal Organoid Kit | 05140 | STEMCELL |
| Gentamicin | HY-A0276 | MCE |
| Cells recovery solution | 354253 | Corning |
| Doxycycline | HY-N0565 | MCE |
| CRD1 | N/A | Cusabio |
| FITC conjugation kit | ab102884 | Abcam |
| TRIzol | 15596018 | Invitrogen |
| Novel Coronavirus (2019-nCoV) Dual Probes qRT-PCR Kit | D8006M | Beyotime |
| Neuraminidase V | N2876 | Sigma-Aldrich |
| Heparan Sulfate | HY-101916 | MCE |
| Camostat | HY-13512 | MCE |
| E-64d | HY-100229 | MCE |
| Bafilomycin A1 | HY-100558 | MCE |
| MG132 | HY-13259 | MCE |
| Cycloheximide (CHX) | HY-12320 | MCE |
| Recombinant SARS-CoV-2 Spike Protein | RP01262 | Abclonal |
| Remdesivir | HY-104077 | MCE |
| Bright-Lumi™ Firefly Luciferase Assay Kit | RG051S | Beyotime |
| Cell Counting Kit-8 Assay | MA0218-2 | meilunbio |
| PrimeScript™ RT reagent Kit with gDNA Eraser | RR047A | Takara |
| SYBR Green PCR Master Mix | K0251 | Thermo Scientific |
| O.C.T. Compound | 4583 | SAKURA |
| BSA | V900933 | Sigma-Aldrich |
| Tween 20 | P9416 | Sigma-Aldrich |
| DAPI | 10236276001 | Sigma-Aldrich |
| Triton X-100 | T8787 | Sigma-Aldrich |
| CellMask™ Deep Red Plasma Membrane Stains | C10046 | Invitrogen |

| | | |
|---|---|---|
| RIPA lysis buffer | C500007 | Sangon Biotech |
| PMSF | A610425 | Sangon Biotech |
| Phosphatase inhibitors | C500017 | Sangon Biotech |
| TaKaRa BCA Protein Assay Kit | T9300A | Takara |
| SuperSignal™ West Pico PLUS (ECL) | 34580 | Thermo Fisher |
| Cell surface protein biotinylation and isolation kit | A44390 | Thermo Fisher |
| Trypsin | EMS0006 | Sigma-Aldrich |
| Rapigest | 186001860 | Waters |
| Acetonitrile | 34851 | Sigma-Aldrich |
| Iodoacetamide | HY-34477 | MCE |
| 2,2′ -thiodiethanol | 719749 | Sigma-Aldrich |
| PNGaseF | P0704S | New England Biolabs |
| TLCK | sc-201296 | Santa cruz |
| Protein A/G-sepharose | 80106G | Invitrogen |
| IFN-α | HY-P7022 | MCE |
| IFN-β | HY-P7024 | MCE |
| IFN-γ | HY-P7025 | MCE |
| IFN-λ | HY-P73202 | MCE |
| Propidium Iodide | 537059 | Sigma-Aldrich |
