# Supplementary material for: Tight junction protein LSR is a host defense factor against SARS-CoV-2 infection in the small intestine
Source: EMBO J. 2024 Oct 23;43(23):6124–51. doi: 10.1038/s44318-024-00281-4 (PMC11612383; doi:10.1038/s44318-024-00281-4)
Supplement: Supplementary file 3 — Table EV3 [file 44318_2024_281_MOESM3_ESM.pdf]

Table EV3: Primer sequences used for qRT-PCR

Gene	Forward	Reverse
Lsr (mouse)	CTACAACCCCTATGTGGAGTGC	CTGCCCTGGTAGTAGTCTCCC
LSR (human)	GACGTTGACAGGAGTAGCTCAG	CCTTCTCCATGTAGTACAGGACC
Cxcl10 (mouse)	CCGTCATTTTCTGCCTCATCC	GGCCCGTCATCGATATGGAT
Il12 (mouse)	CCCTGGAGAAATGGTGGTCC	GAACCTCGCCTCCTTTGTGA
Il6 (mouse)	GAGGATACCACTCCCAACAGACC	AAGTGCATCATCGTTGTTCATACA
Il2 (mouse)	GAGCAGCTGTTGATGGACCT	AATCCAGAACATGCCGCAGA
Tnfα (mouse)	CGTCGTAGCAAACCACCAAG	TTGAAGAGAACCTGGGAGTAGACA
Ifnγ (mouse)	GCAAGGCGAAAAAGGATGCA	CGACTCCTTTTCCGCTTCCT
Ifnα2 (mouse)	CTCCACCAGCAGCTCAATGA	ACAGGGCTCTCCAGACTTCT
F4/80 (mouse)	CGTGTTGTTGGTGGCACTGTGA	CCACATCAGTGTTCCAGGAGAC
Ly6g (mouse)	TATCTGTGCAGCCCTTCTCC	GAGAAGGGGCAGGTAGTTGT
VSV-P	TCTCGTCTGGATCAGGCGG	TGCTCTTCCACTCCATCCTCTTGG
Spike	TTGCTGCTAGAGACCTCATTTGT	AAGTGATTGTACCCGCTAACAGT
Nucleocapsid	CACATTGGCACCCGCAATC	GAGGAACGAGAAGAGGCTTG
β-actin (human)	CGTCACCAACTGGGACGACA	CTTCTCGCGGTTGGCCTTGG
β-actin (mouse)	CATTGCTGACAGGATGCAGAAGG	TGCTGGAAGGTGGACAGTGAGG
LSR siRNA	TTTGAAGGAACACTGATGA	
LSR-CRD1M	GGCACAGGCTGCACCGCACACTGCTG	TGTGCGGTGCAGCCTGTGCCCAG
	CAGCCTACGTCAGGTGCCCCTGCT	CAGATGCCCAGGAGGA
LSR-CRD2M	GCTCCCGCAGCTCCAGACAAGGCAGC	TTGTCTGGAGCTGCGGGAGCCCTG
	TGCCCCCGAGGCCCTGTATGCCG	ACGTAGCAGCAGCAAG
