# Supplementary figures and images for: Tight junction protein LSR is a host defense factor against SARS-CoV-2 infection in the small intestine (part 1 of 4)
Source: EMBO J. 2024 Oct 23;43(23):6124–51. doi: 10.1038/s44318-024-00281-4 (PMC11612383; doi:10.1038/s44318-024-00281-4)

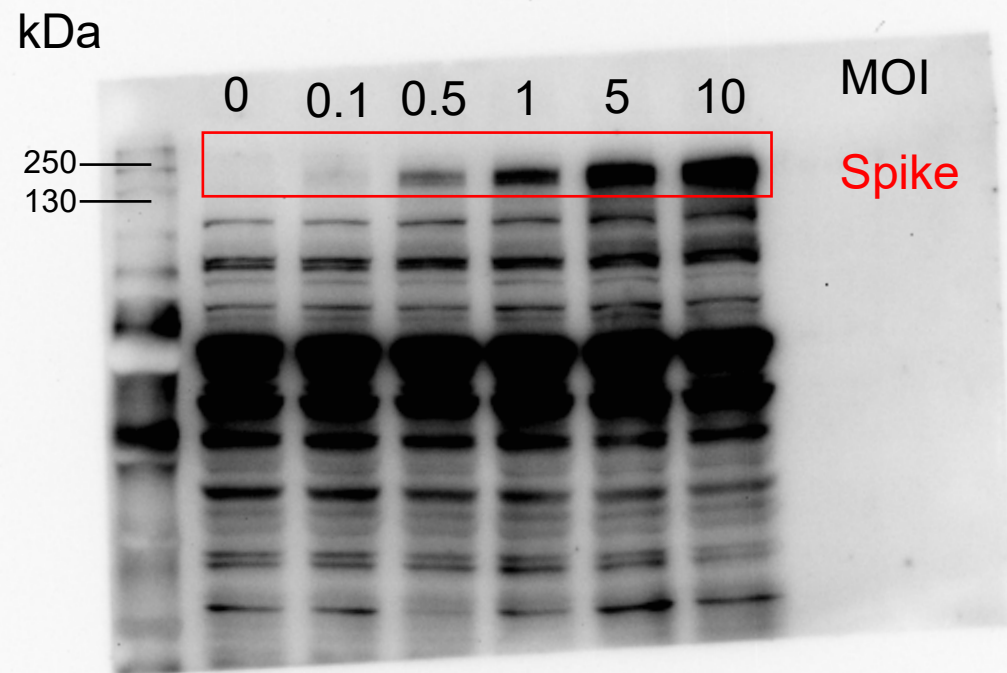

kDa

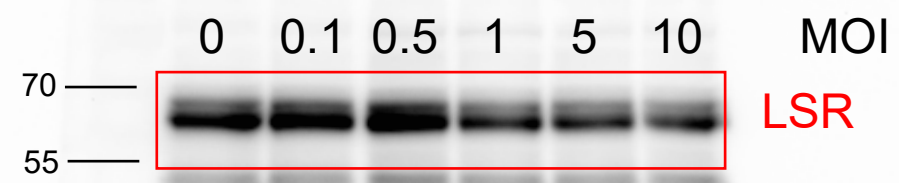

kDa

55 —

40 —

0

0.1

0.5

1

5

10

MOI

$\beta$ -actin

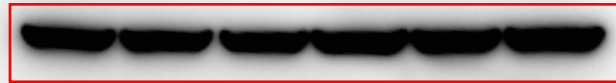

Supplement: Supplementary file 5 — Source data Fig. 1 [file 44318_2024_281_MOESM5_ESM.zip › Figure1/1B/1B.pdf]

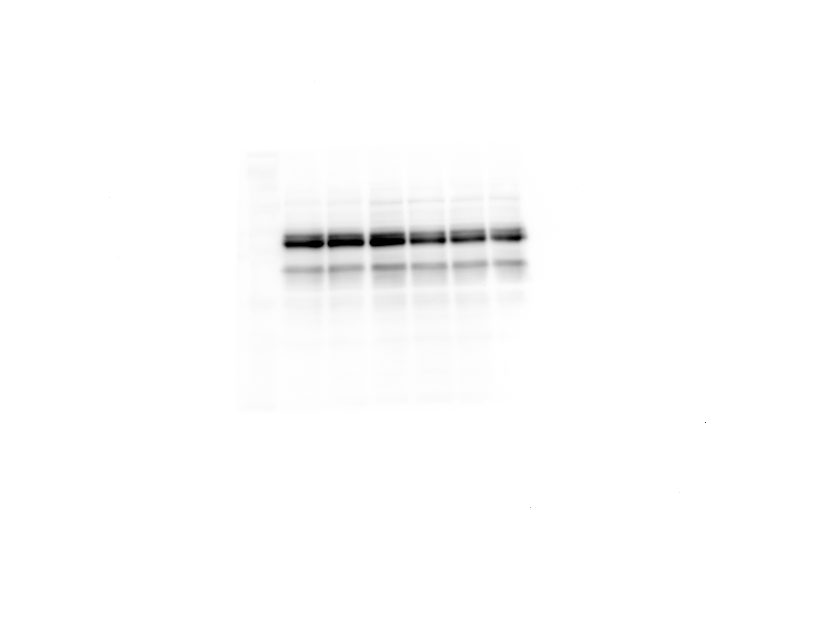

Supplement: Supplementary file 5 — Source data Fig. 1 [file 44318_2024_281_MOESM5_ESM.zip › Figure1/1B/western LSR.png]

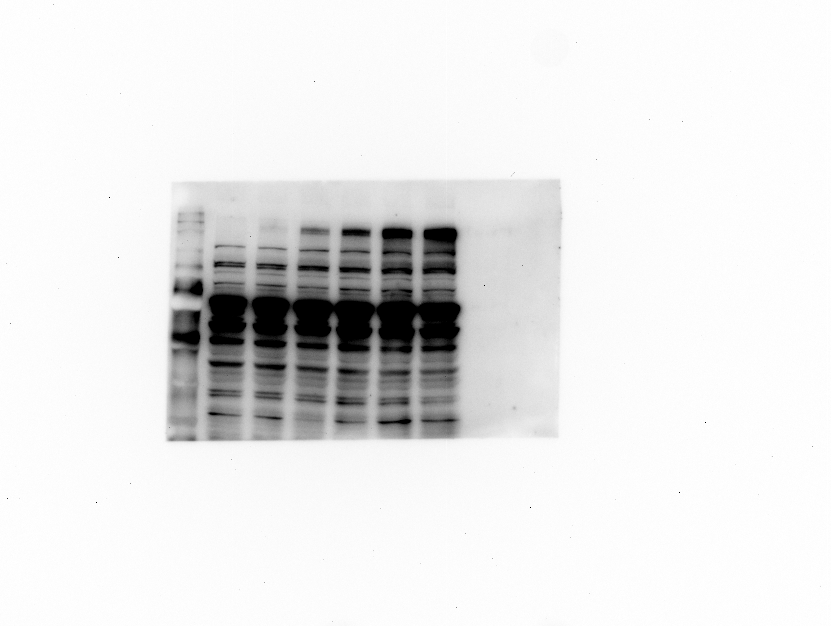

Supplement: Supplementary file 5 — Source data Fig. 1 [file 44318_2024_281_MOESM5_ESM.zip › Figure1/1B/western Spike.png]

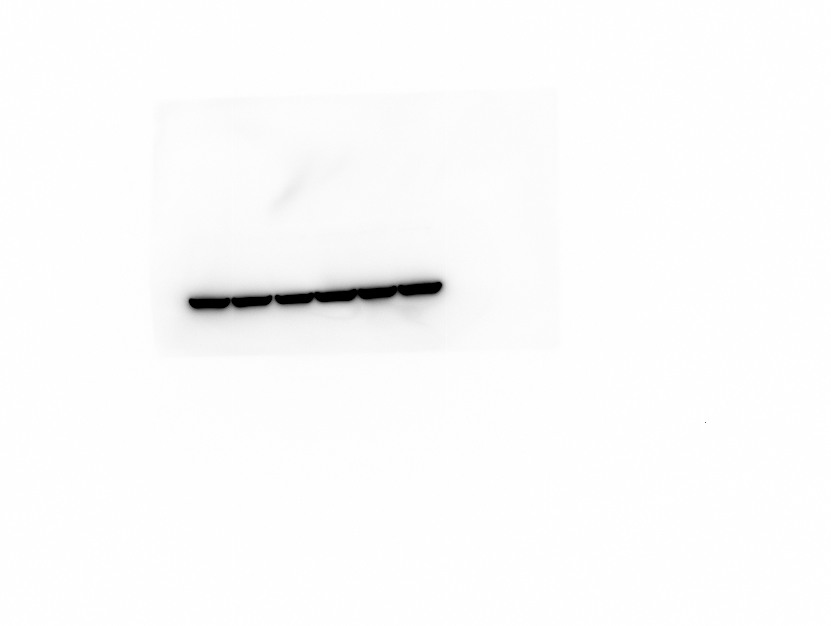

Supplement: Supplementary file 5 — Source data Fig. 1 [file 44318_2024_281_MOESM5_ESM.zip › Figure1/1B/western actin.png]

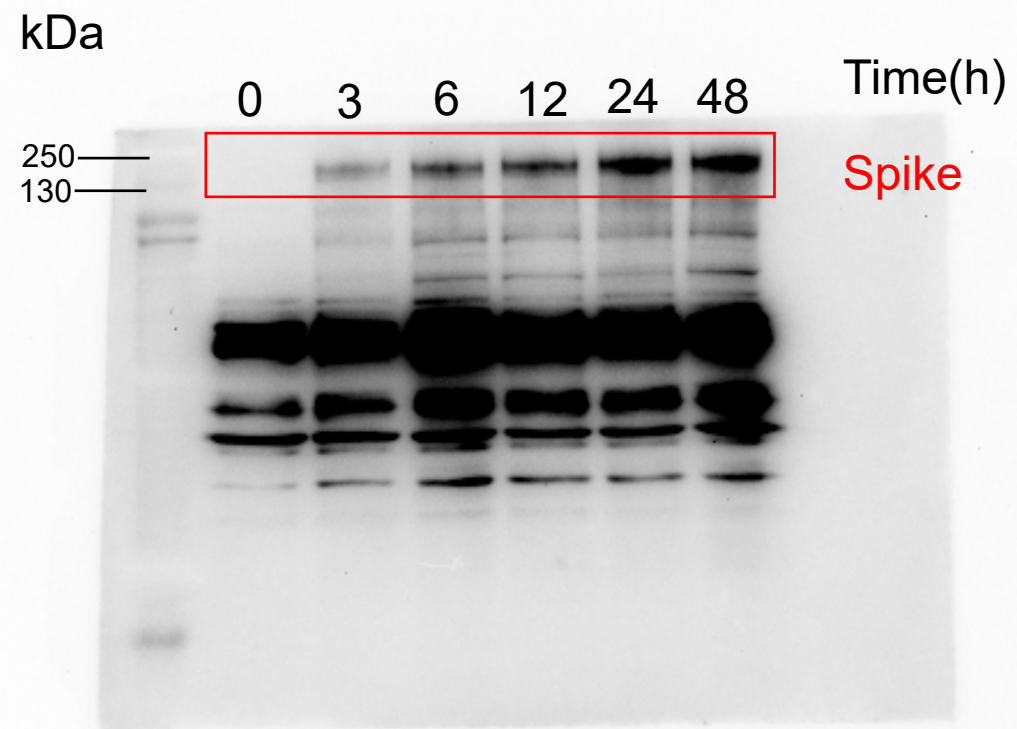

kDa

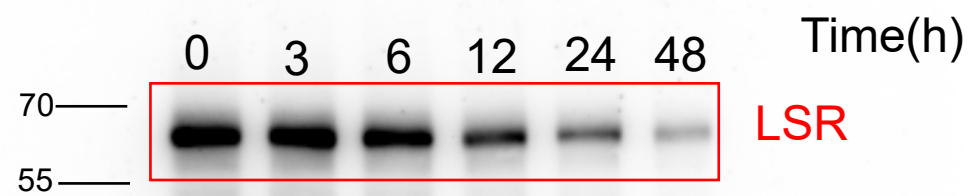

kDa

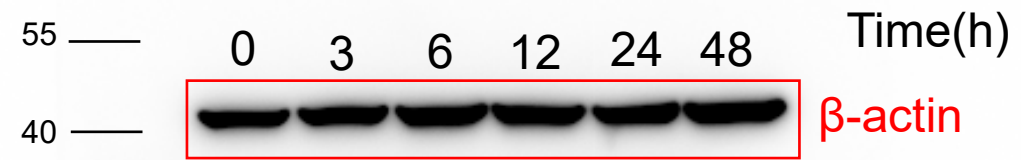

Supplement: Supplementary file 5 — Source data Fig. 1 [file 44318_2024_281_MOESM5_ESM.zip › Figure1/1D/1D.pdf]

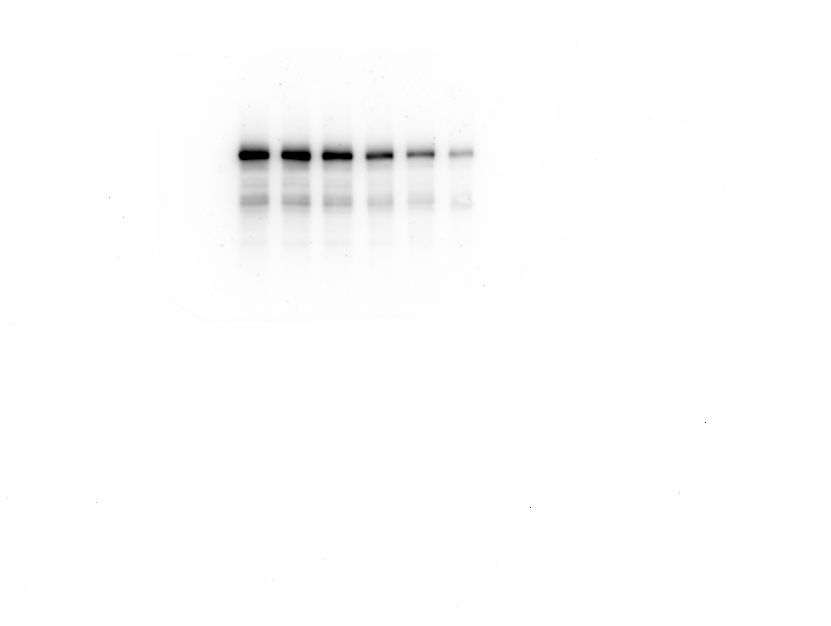

Supplement: Supplementary file 5 — Source data Fig. 1 [file 44318_2024_281_MOESM5_ESM.zip › Figure1/1D/western LSR.png]

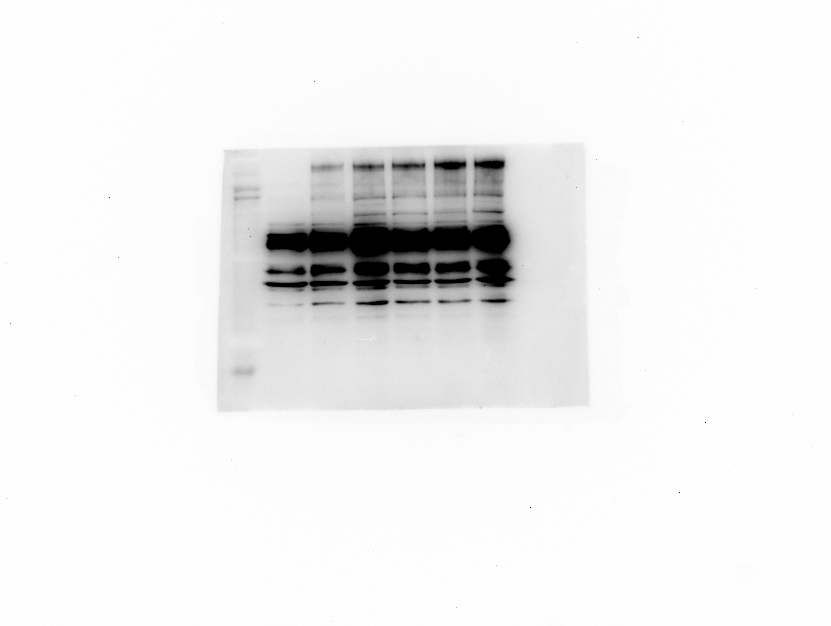

Supplement: Supplementary file 5 — Source data Fig. 1 [file 44318_2024_281_MOESM5_ESM.zip › Figure1/1D/western Spike.png]

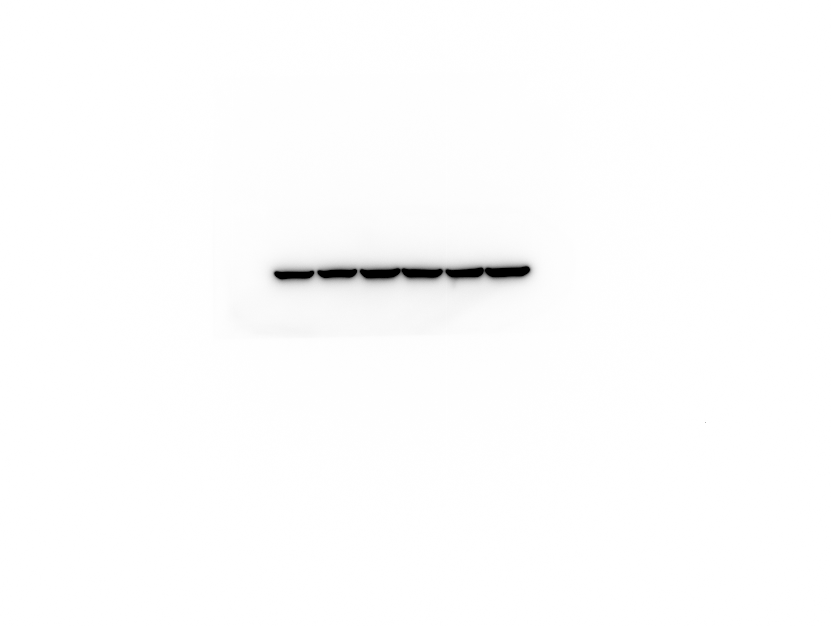

Supplement: Supplementary file 5 — Source data Fig. 1 [file 44318_2024_281_MOESM5_ESM.zip › Figure1/1D/western actin.png]

kDa

Control

VSV-SARS-CoV-2

250—  
130—

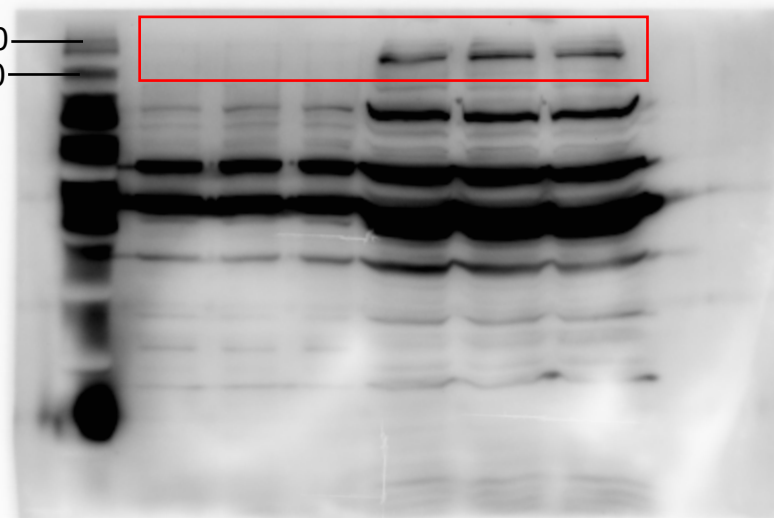

Spike

kDa

70—  
55—

Control VSV-SARS-CoV-2

LSR

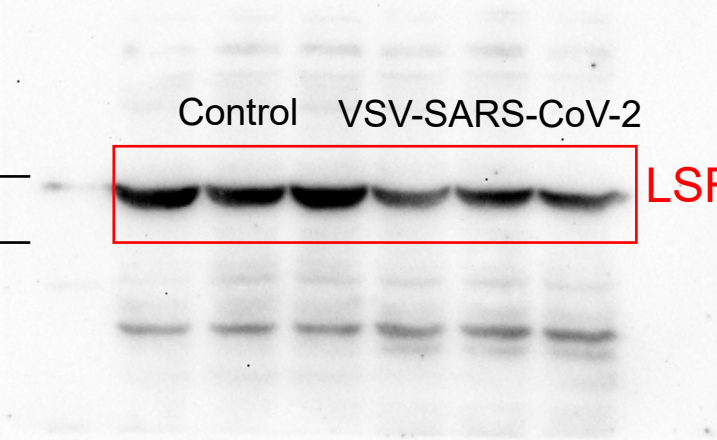

kDa

55 —

40 —

Control

VSV-SARS-CoV-2

$\beta$ -actin

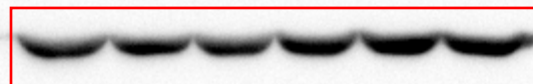

Supplement: Supplementary file 5 — Source data Fig. 1 [file 44318_2024_281_MOESM5_ESM.zip › Figure1/1F/1F.pdf]

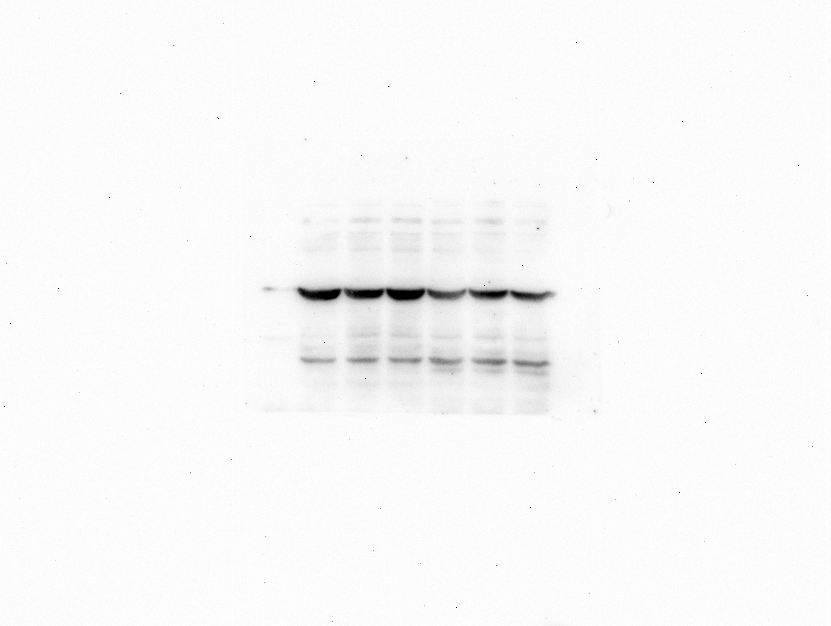

Supplement: Supplementary file 5 — Source data Fig. 1 [file 44318_2024_281_MOESM5_ESM.zip › Figure1/1F/western LSR.png]

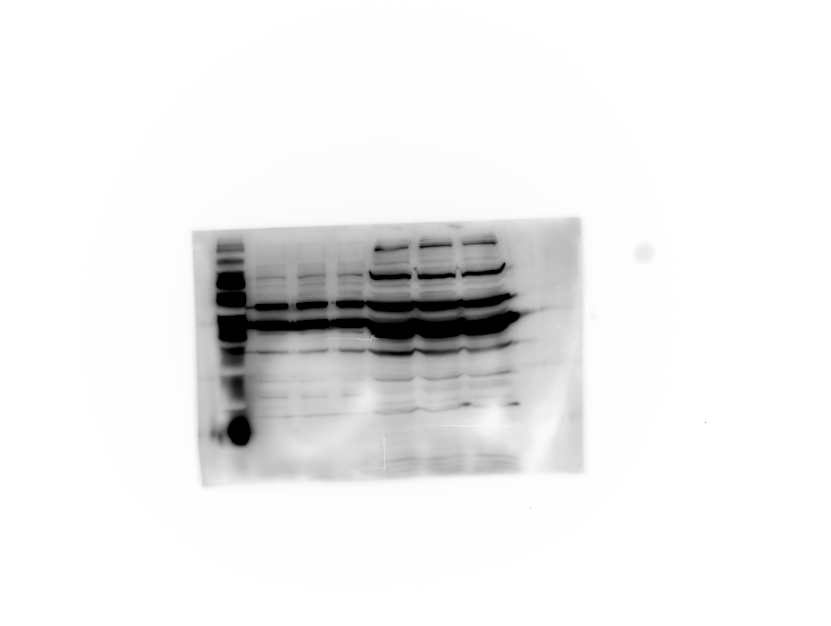

Supplement: Supplementary file 5 — Source data Fig. 1 [file 44318_2024_281_MOESM5_ESM.zip › Figure1/1F/western Spike.png]

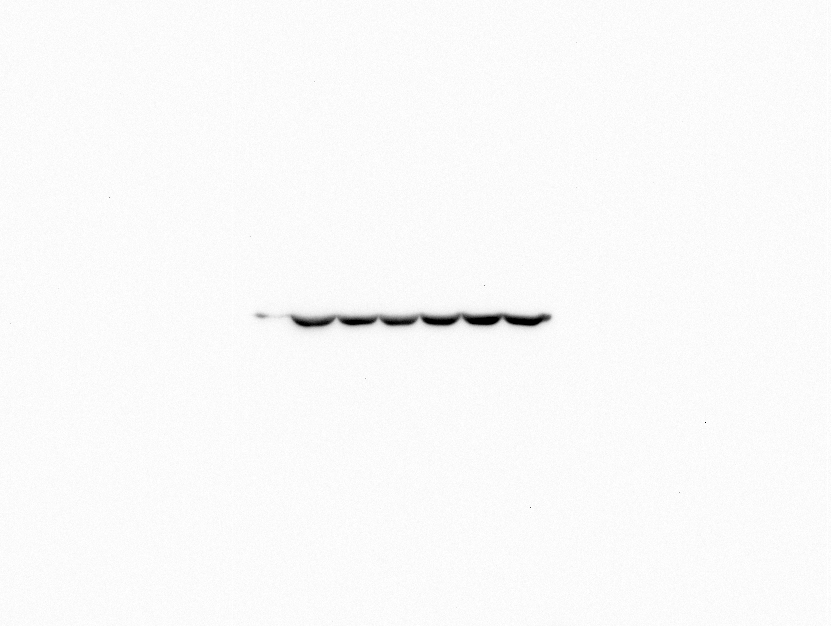

Supplement: Supplementary file 5 — Source data Fig. 1 [file 44318_2024_281_MOESM5_ESM.zip › Figure1/1F/western actin.png]

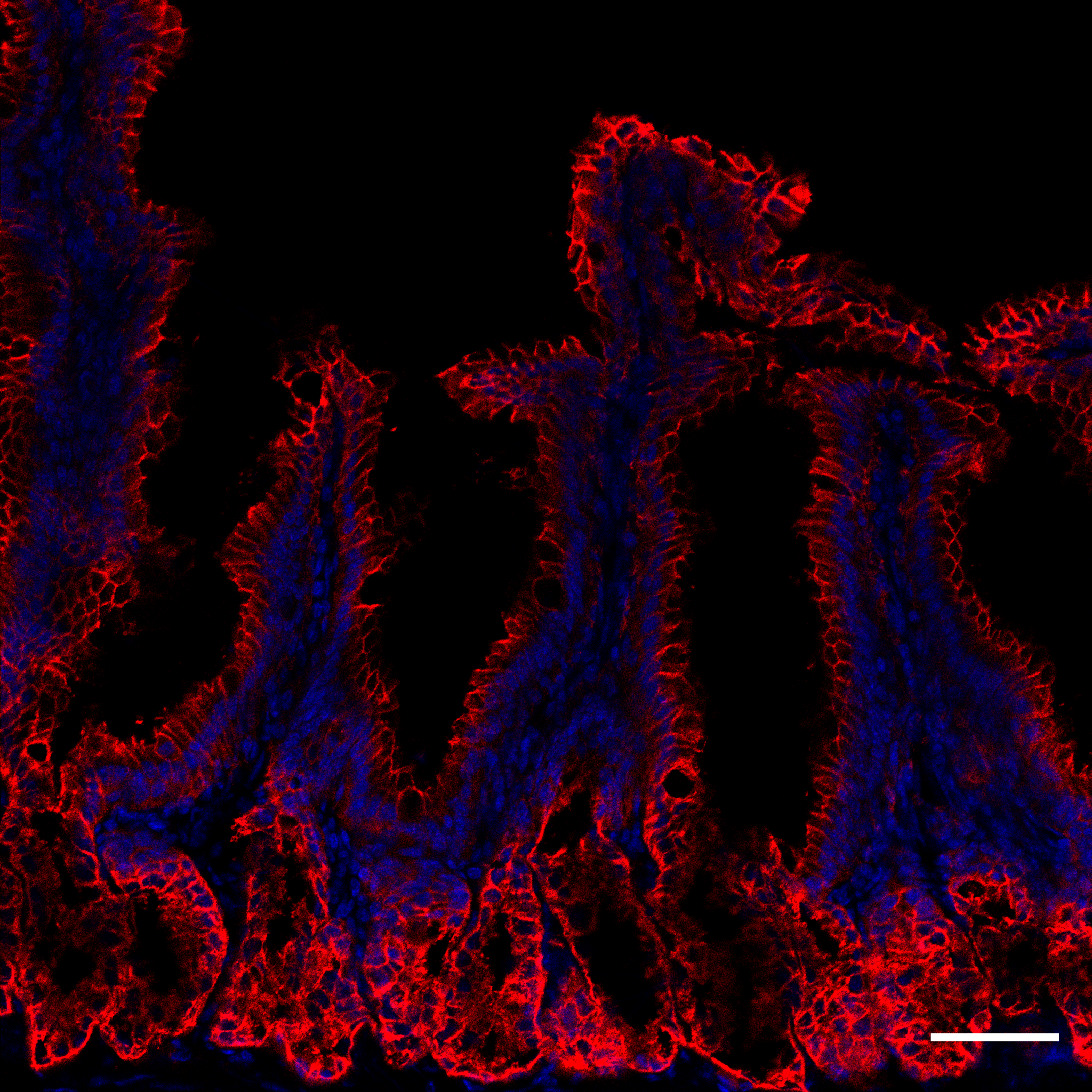

Supplement: Supplementary file 5 — Source data Fig. 1 [file 44318_2024_281_MOESM5_ESM.zip › Figure1/1H/IF LSR Control.tif]

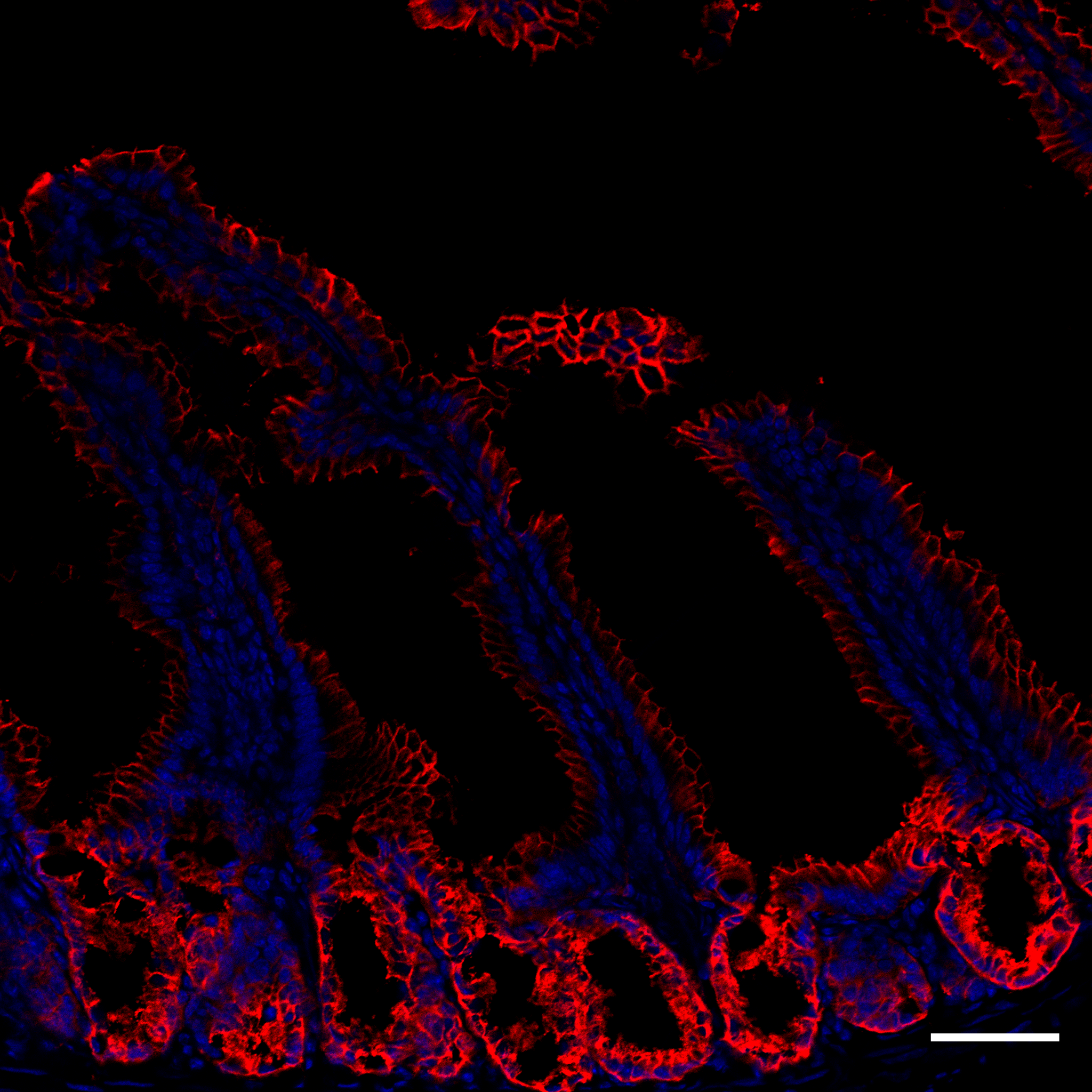

Supplement: Supplementary file 5 — Source data Fig. 1 [file 44318_2024_281_MOESM5_ESM.zip › Figure1/1H/IF LSR VSV-SARS-COV-2.tif]

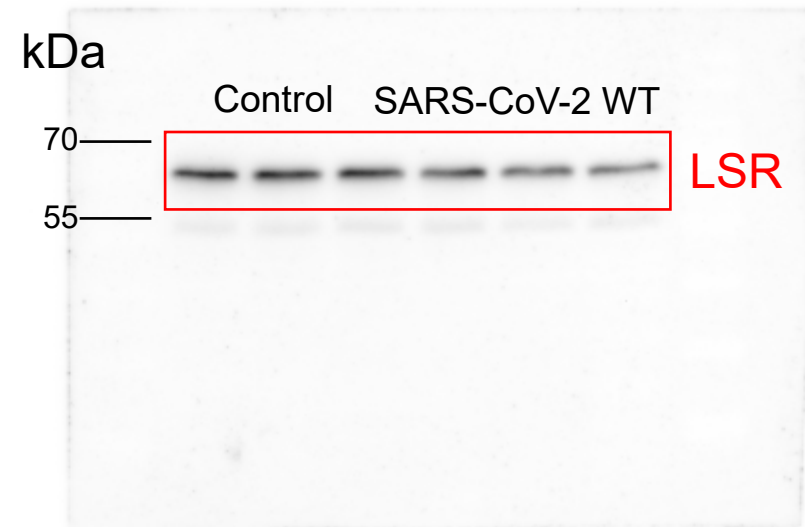

kDa

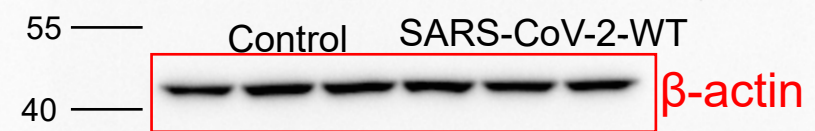

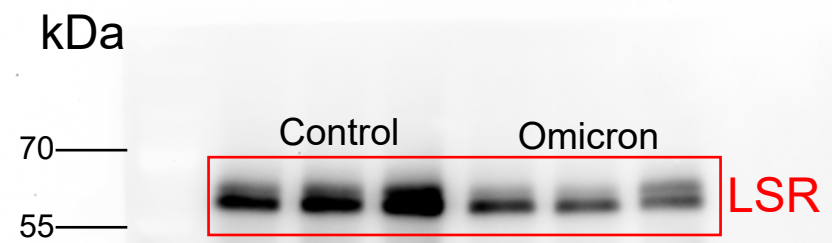

kDa

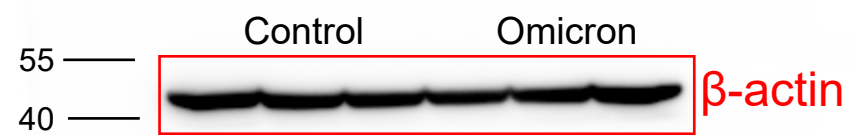

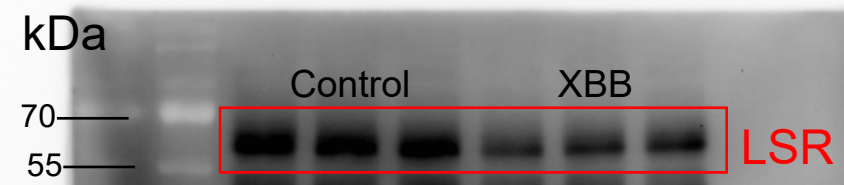

kDa

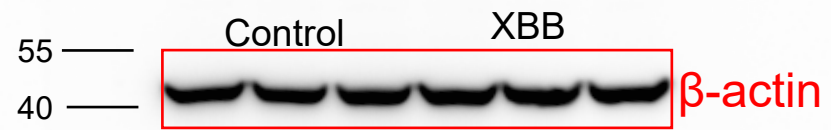

Supplement: Supplementary file 5 — Source data Fig. 1 [file 44318_2024_281_MOESM5_ESM.zip › Figure1/1J/1J.pdf]

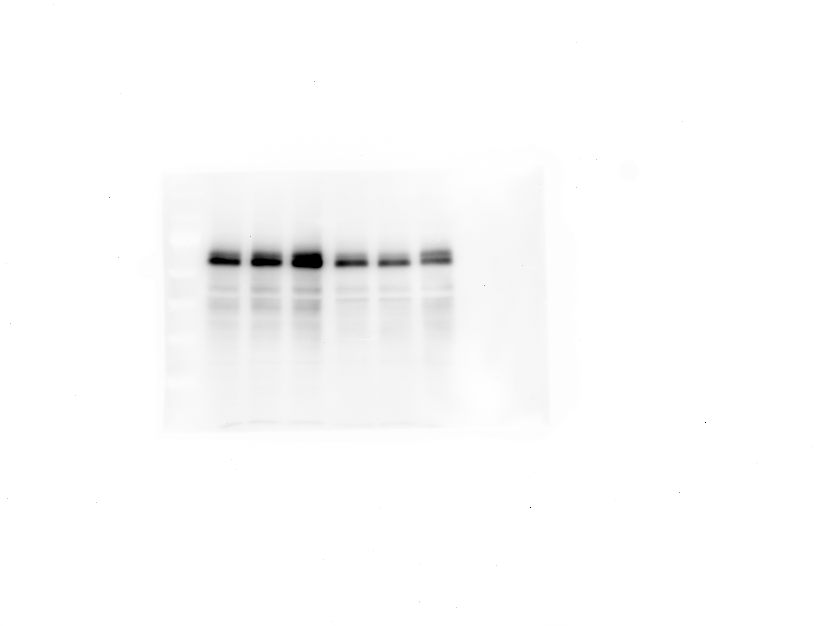

Supplement: Supplementary file 5 — Source data Fig. 1 [file 44318_2024_281_MOESM5_ESM.zip › Figure1/1J/western LSR Omicron.png]

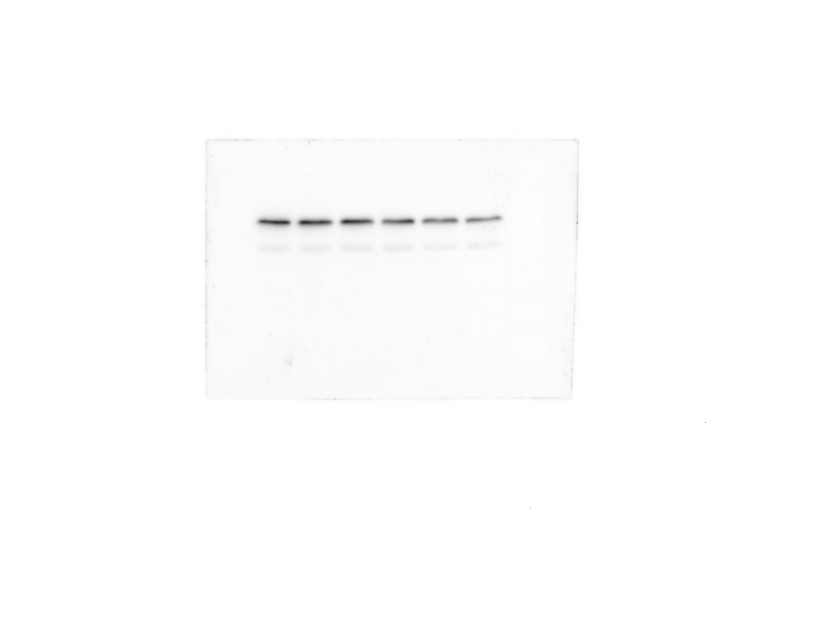

Supplement: Supplementary file 5 — Source data Fig. 1 [file 44318_2024_281_MOESM5_ESM.zip › Figure1/1J/western LSR SARS-COV-2 WT.png]

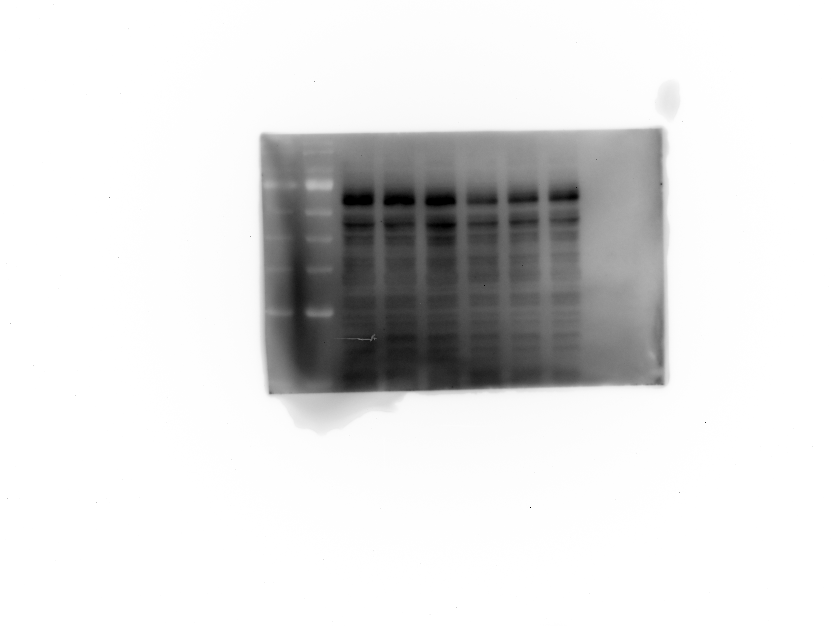

Supplement: Supplementary file 5 — Source data Fig. 1 [file 44318_2024_281_MOESM5_ESM.zip › Figure1/1J/western LSR XBB.png]

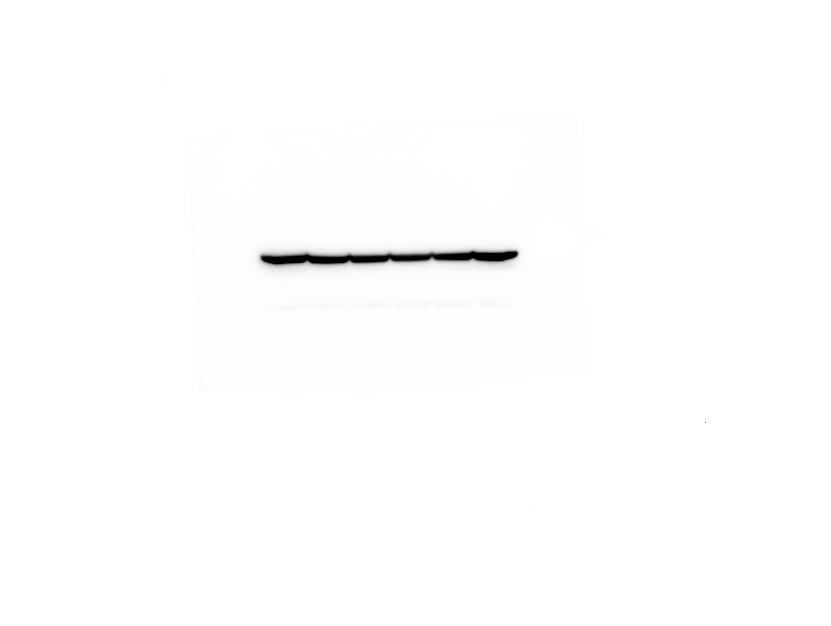

Supplement: Supplementary file 5 — Source data Fig. 1 [file 44318_2024_281_MOESM5_ESM.zip › Figure1/1J/western actin Omicron.png]

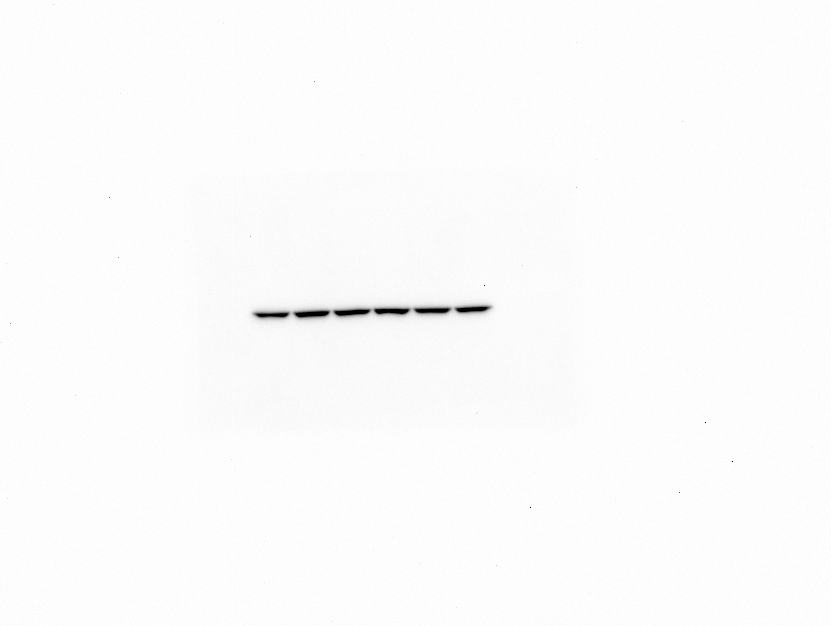

Supplement: Supplementary file 5 — Source data Fig. 1 [file 44318_2024_281_MOESM5_ESM.zip › Figure1/1J/western actin SARS-COV-2 WT.png]

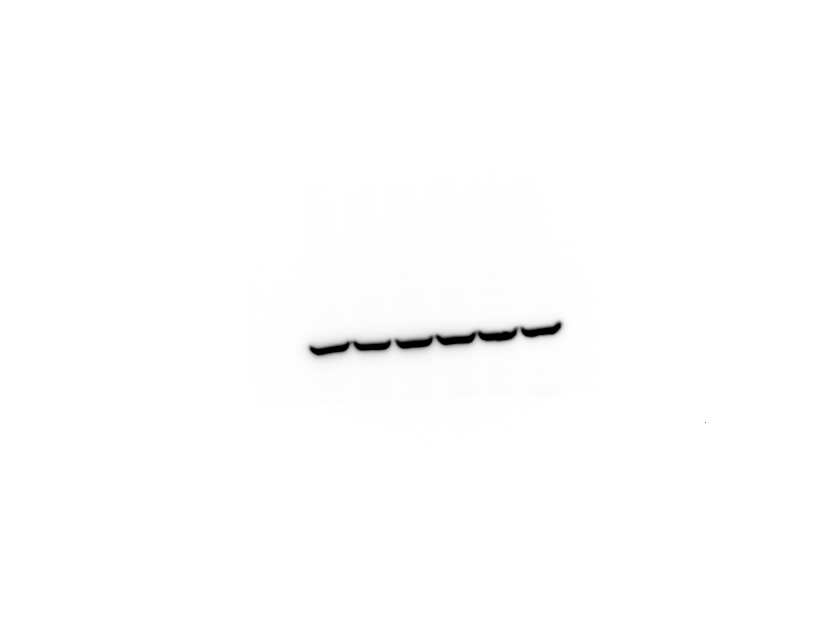

Supplement: Supplementary file 5 — Source data Fig. 1 [file 44318_2024_281_MOESM5_ESM.zip › Figure1/1J/western actin XBB.png]

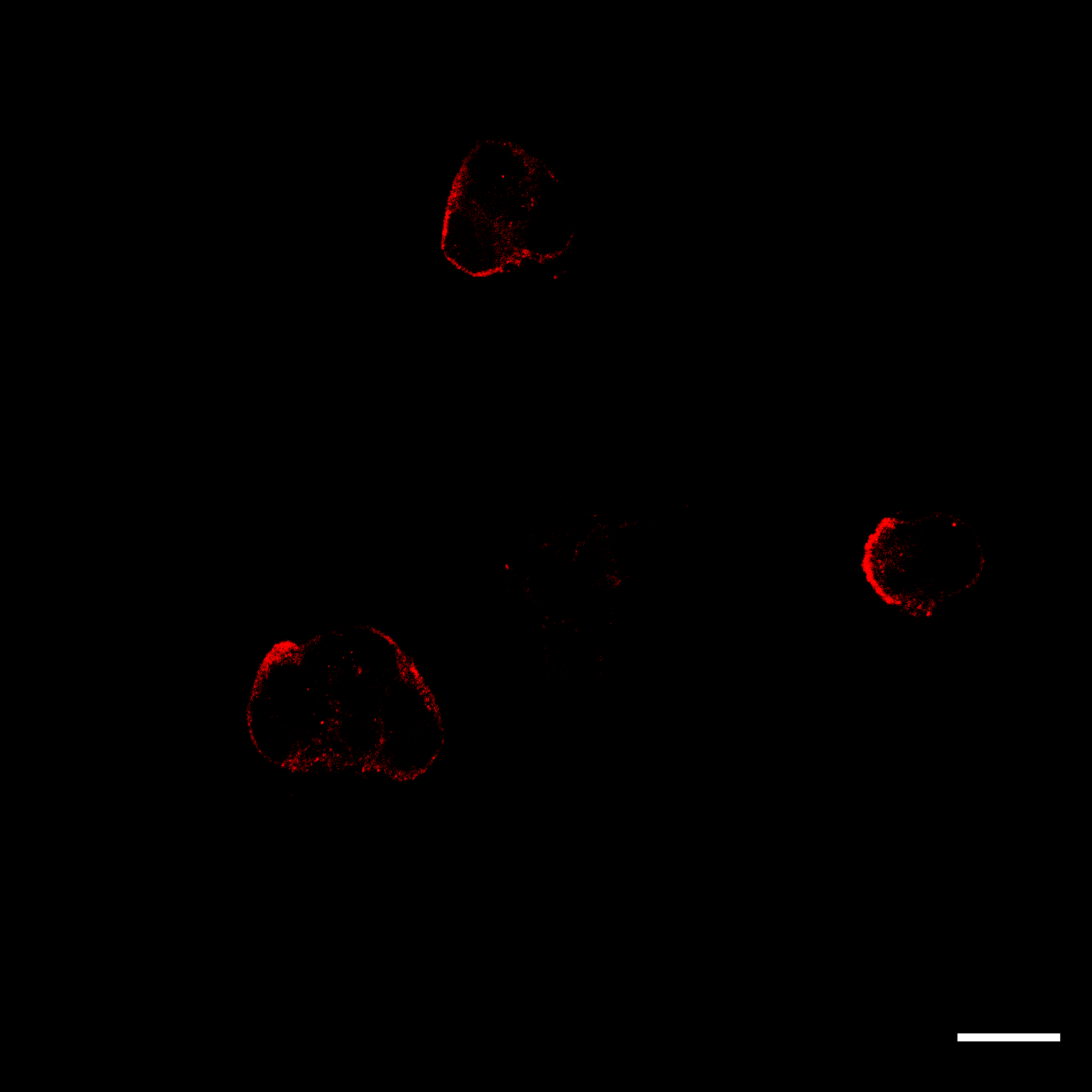

Supplement: Supplementary file 5 — Source data Fig. 1 [file 44318_2024_281_MOESM5_ESM.zip › Figure1/1K/IF LSR SARS-COV-2.tif]

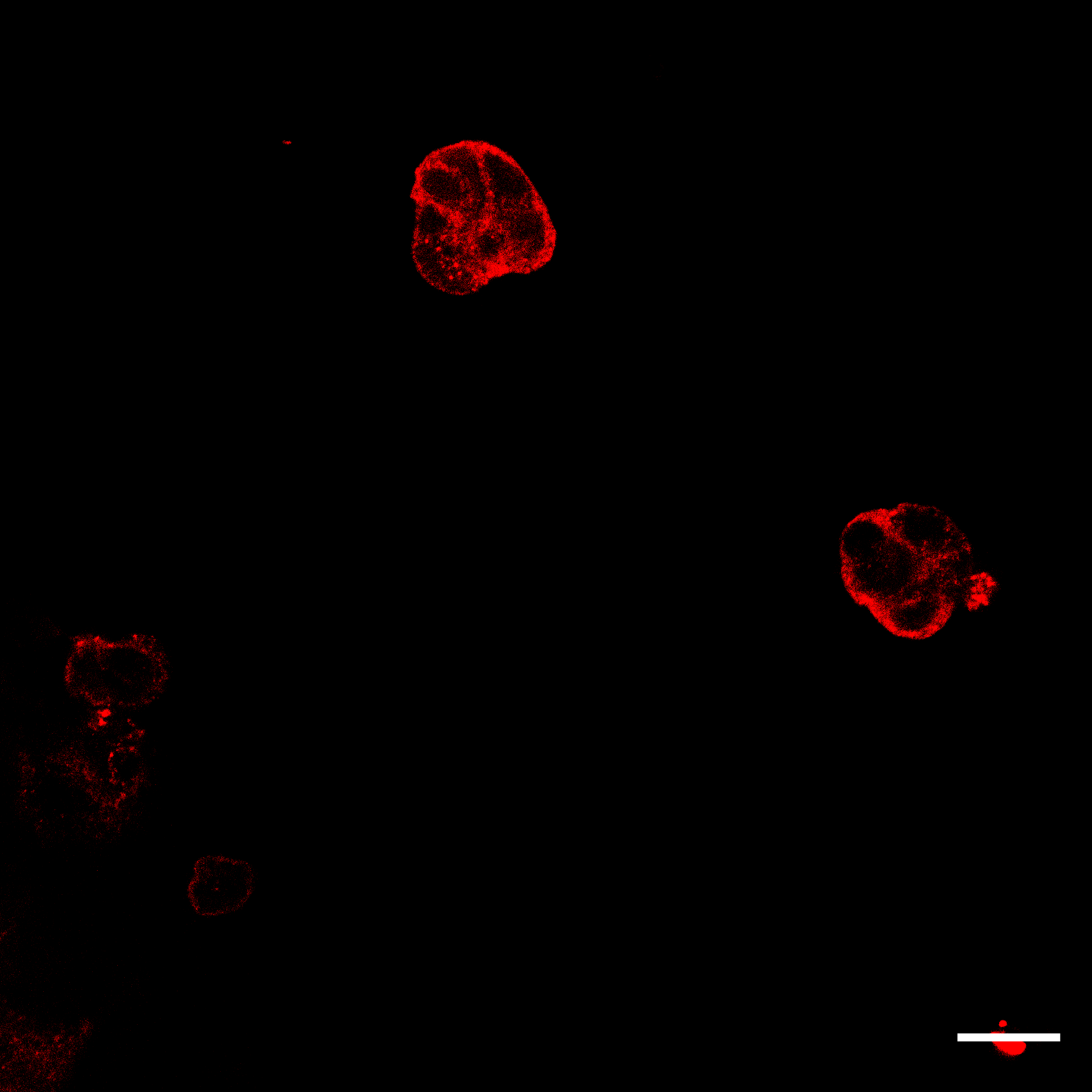

Supplement: Supplementary file 5 — Source data Fig. 1 [file 44318_2024_281_MOESM5_ESM.zip › Figure1/1K/IF LSR control.tif]

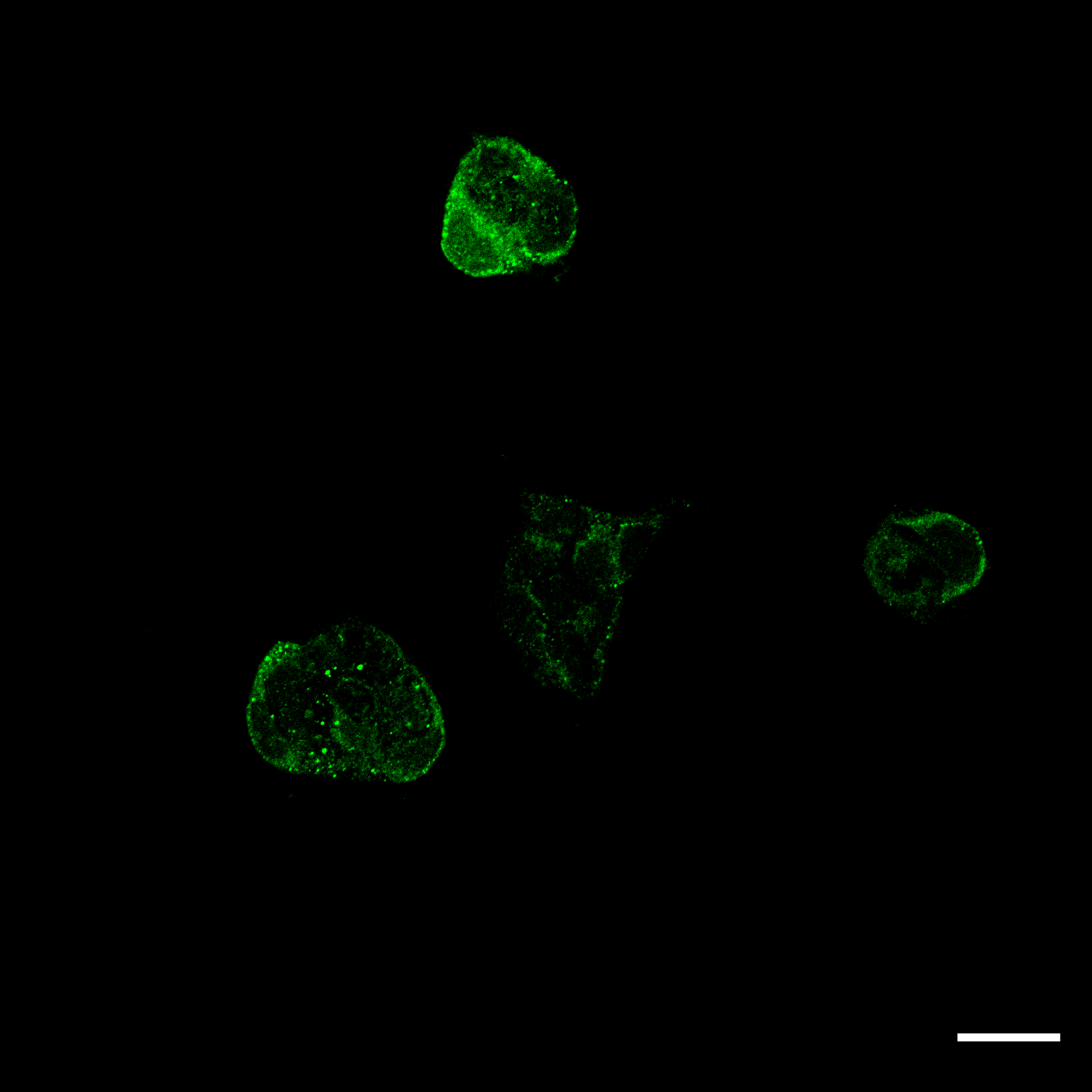

Supplement: Supplementary file 5 — Source data Fig. 1 [file 44318_2024_281_MOESM5_ESM.zip › Figure1/1K/IF Spike SARS-COV-2.tif]

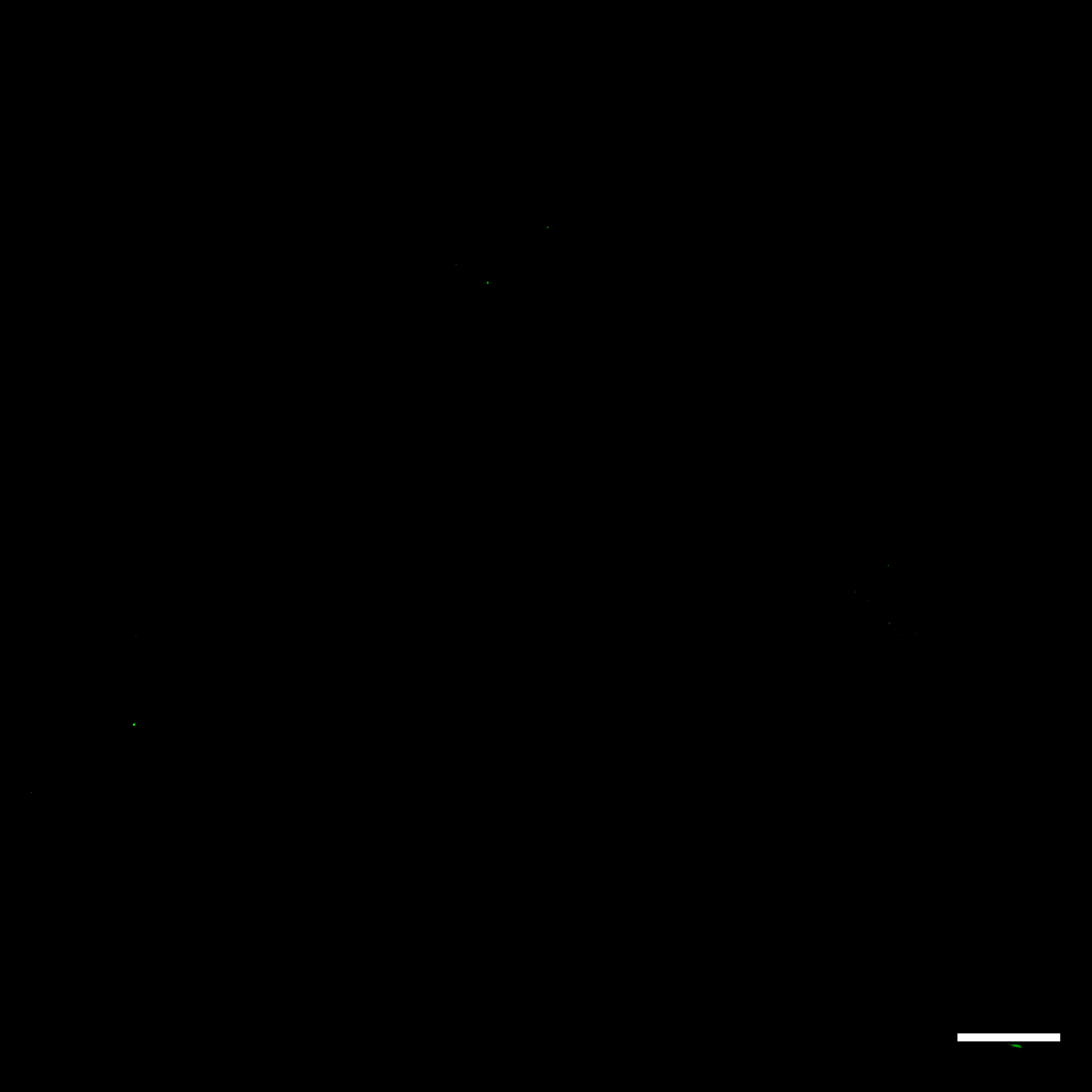

Supplement: Supplementary file 5 — Source data Fig. 1 [file 44318_2024_281_MOESM5_ESM.zip › Figure1/1K/IF Spike control.tif]

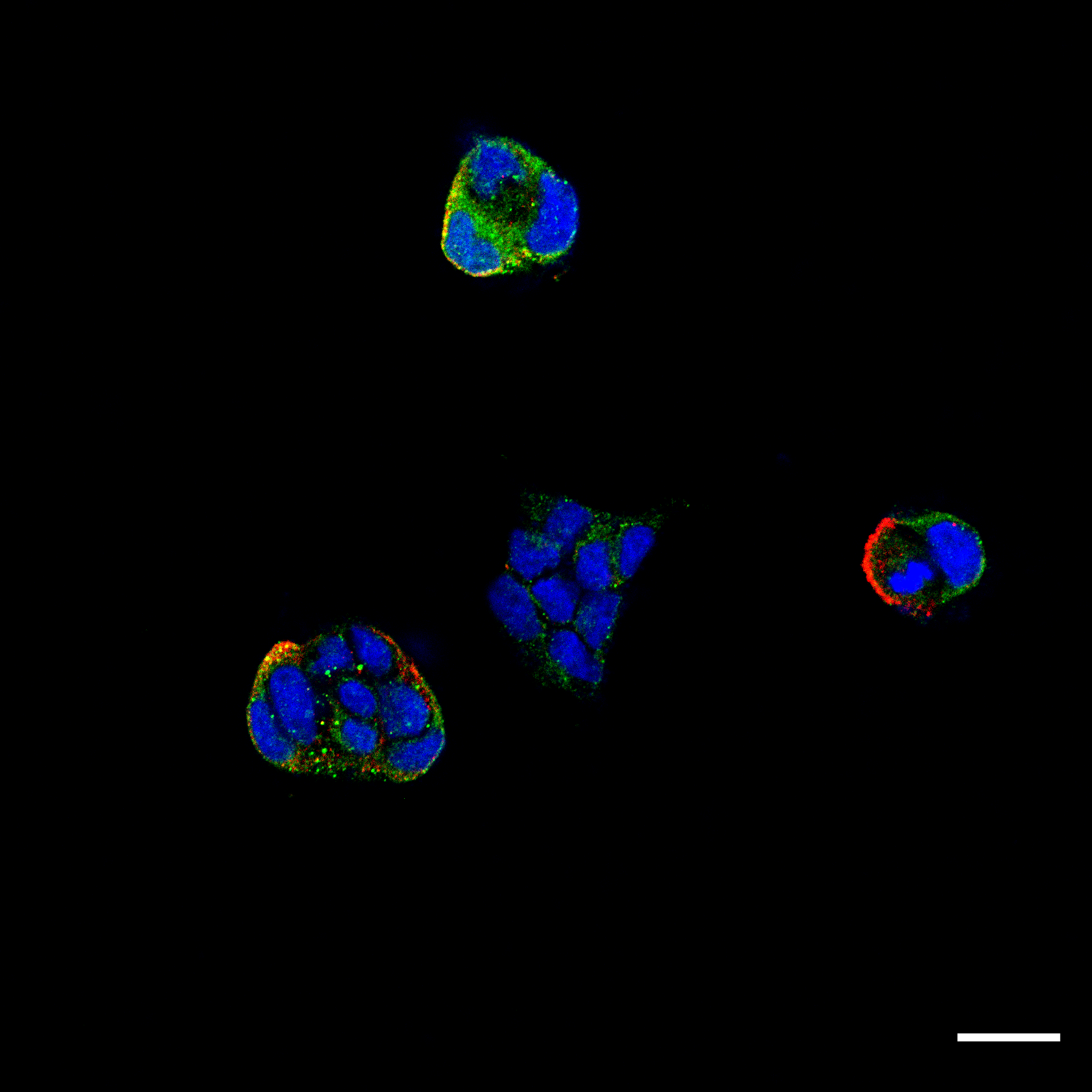

Supplement: Supplementary file 5 — Source data Fig. 1 [file 44318_2024_281_MOESM5_ESM.zip › Figure1/1K/IF merge SARS-COV-2.tif]

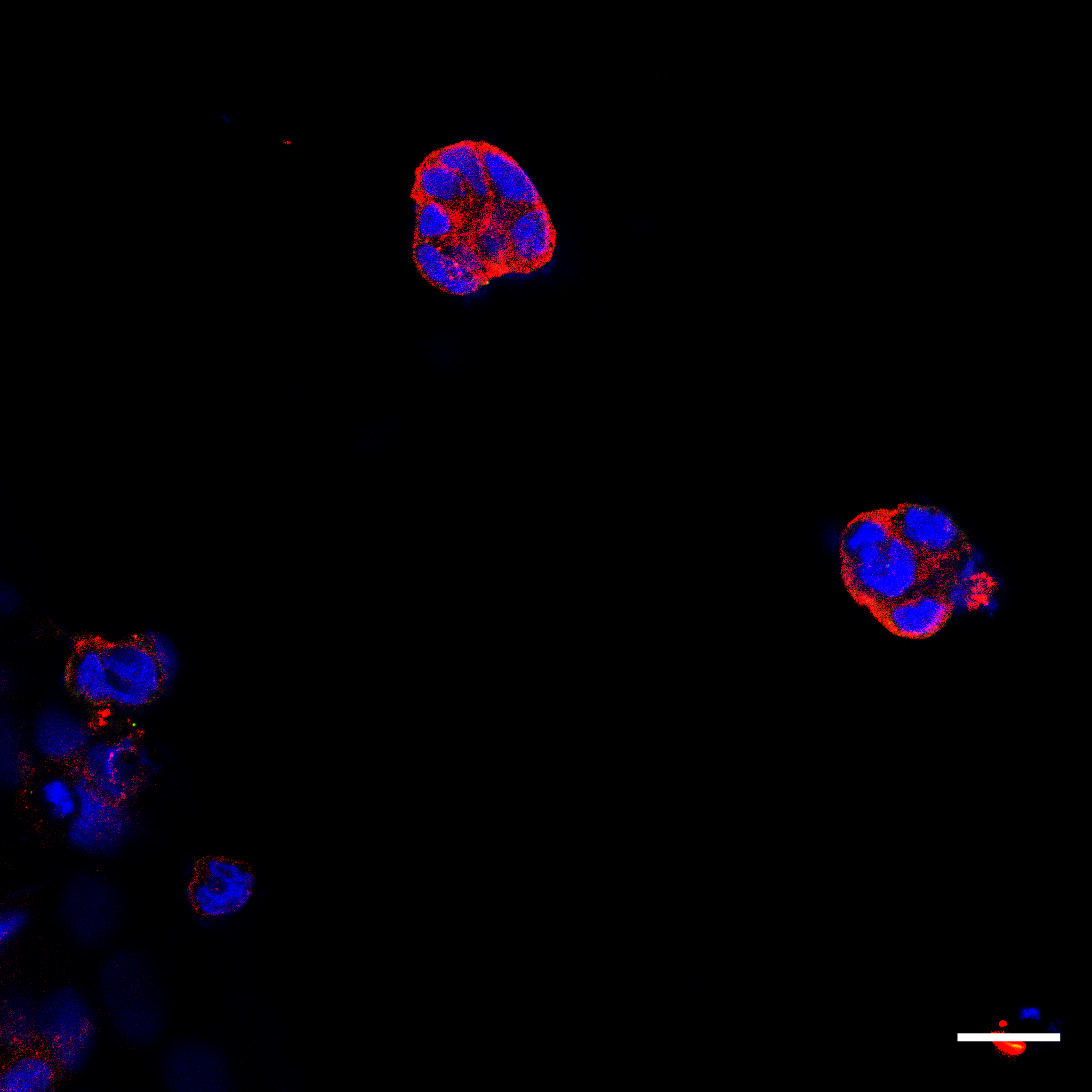

Supplement: Supplementary file 5 — Source data Fig. 1 [file 44318_2024_281_MOESM5_ESM.zip › Figure1/1K/IF merge control.tif]

kDa

70—  
55—

Control

SARS-CoV-2 WT

LSR

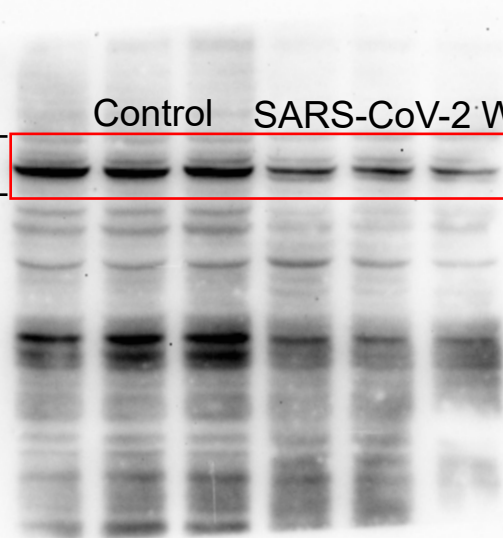

kDa

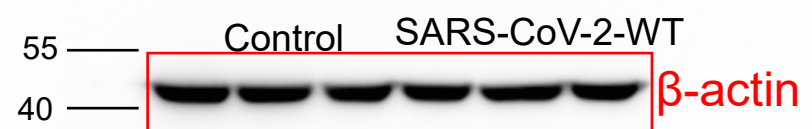

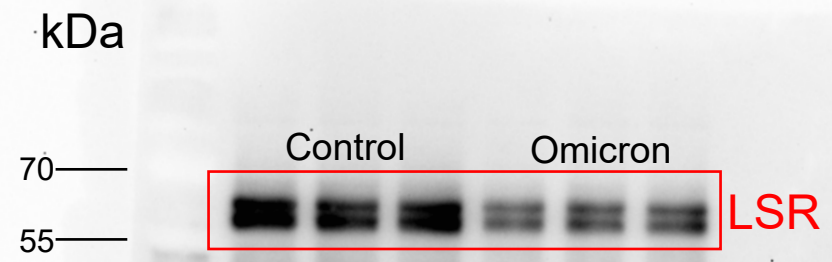

kDa

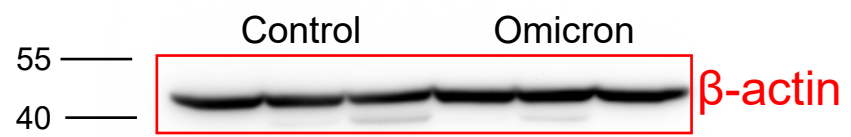

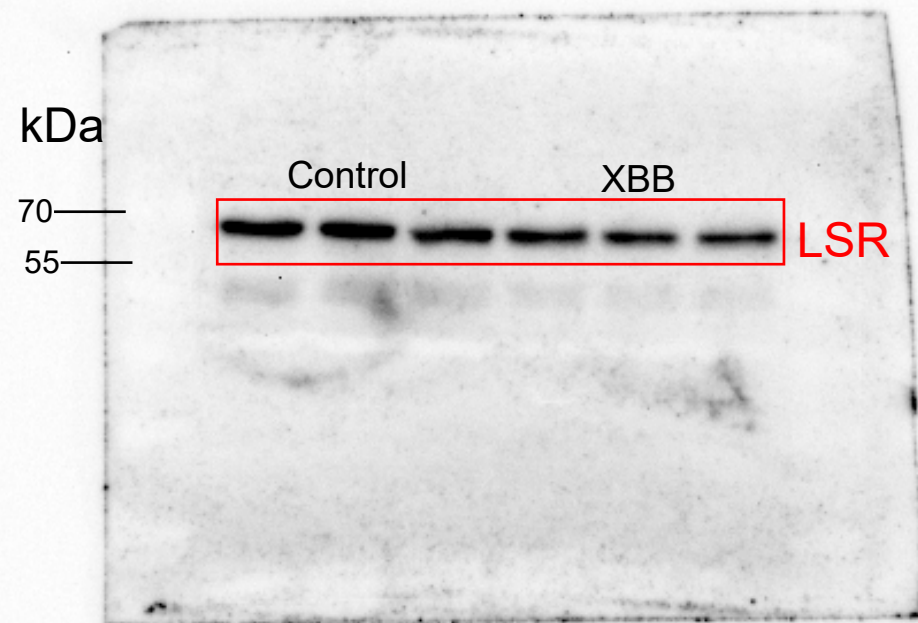

kDa

55 —  
40 —

Control

XBB

$\beta$ -actin

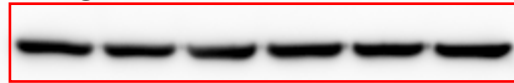

Supplement: Supplementary file 5 — Source data Fig. 1 [file 44318_2024_281_MOESM5_ESM.zip › Figure1/1M/1M.pdf]

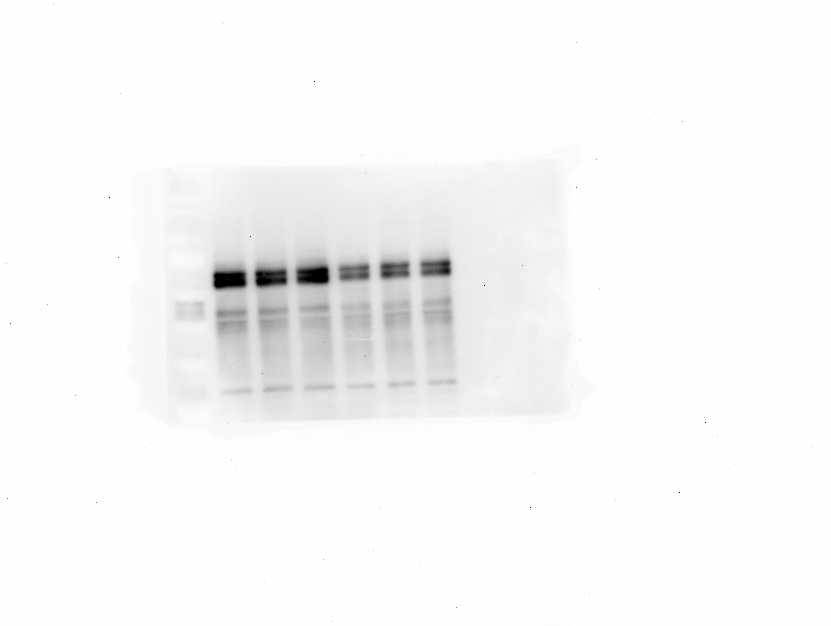

Supplement: Supplementary file 5 — Source data Fig. 1 [file 44318_2024_281_MOESM5_ESM.zip › Figure1/1M/western LSR Omicron.png]

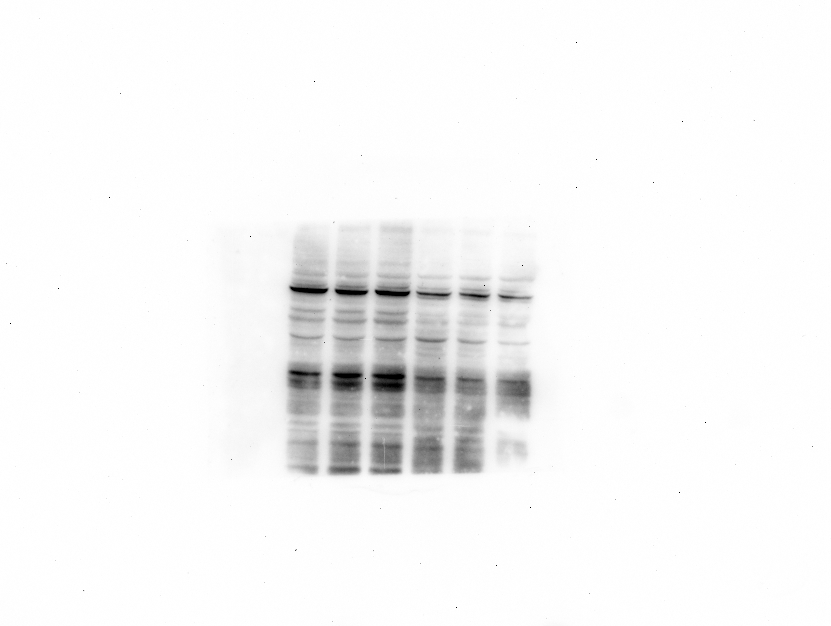

Supplement: Supplementary file 5 — Source data Fig. 1 [file 44318_2024_281_MOESM5_ESM.zip › Figure1/1M/western LSR SARS-COV-2 WT.png]

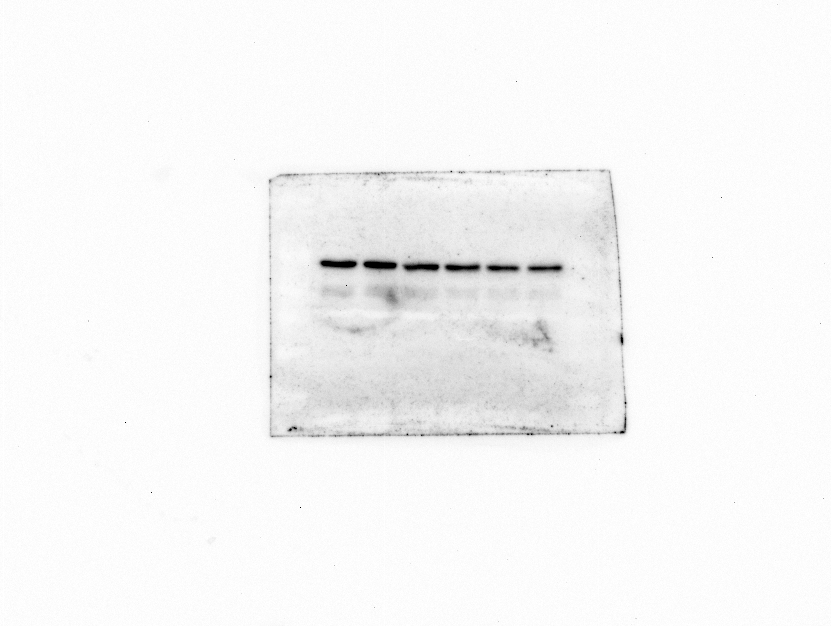

Supplement: Supplementary file 5 — Source data Fig. 1 [file 44318_2024_281_MOESM5_ESM.zip › Figure1/1M/western LSR XBB.png]

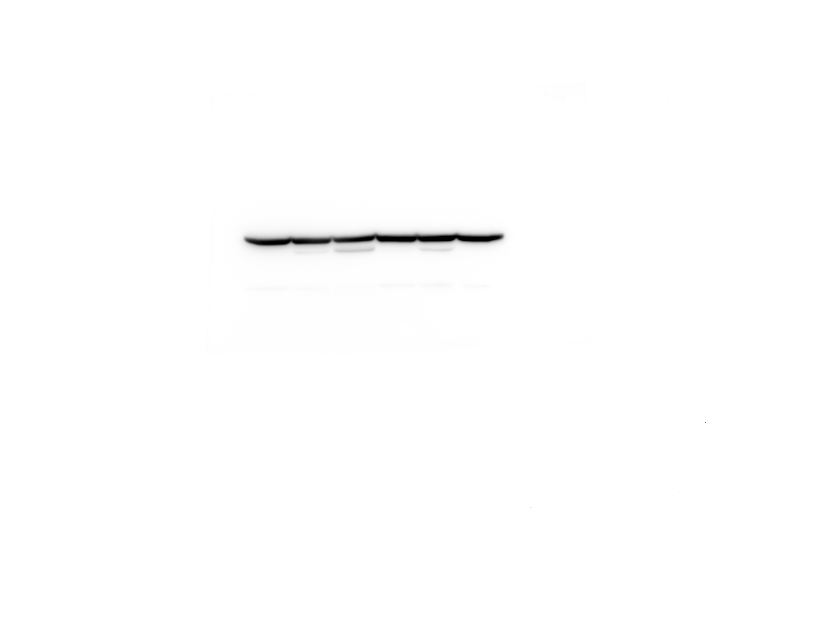

Supplement: Supplementary file 5 — Source data Fig. 1 [file 44318_2024_281_MOESM5_ESM.zip › Figure1/1M/western actin Omicron.png]

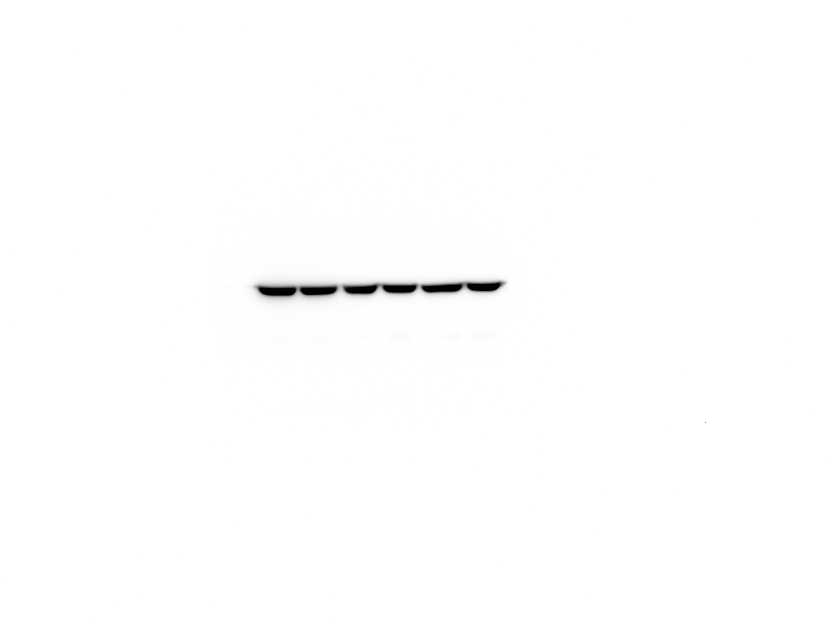

Supplement: Supplementary file 5 — Source data Fig. 1 [file 44318_2024_281_MOESM5_ESM.zip › Figure1/1M/western actin SARS-COV-2 WT.png]

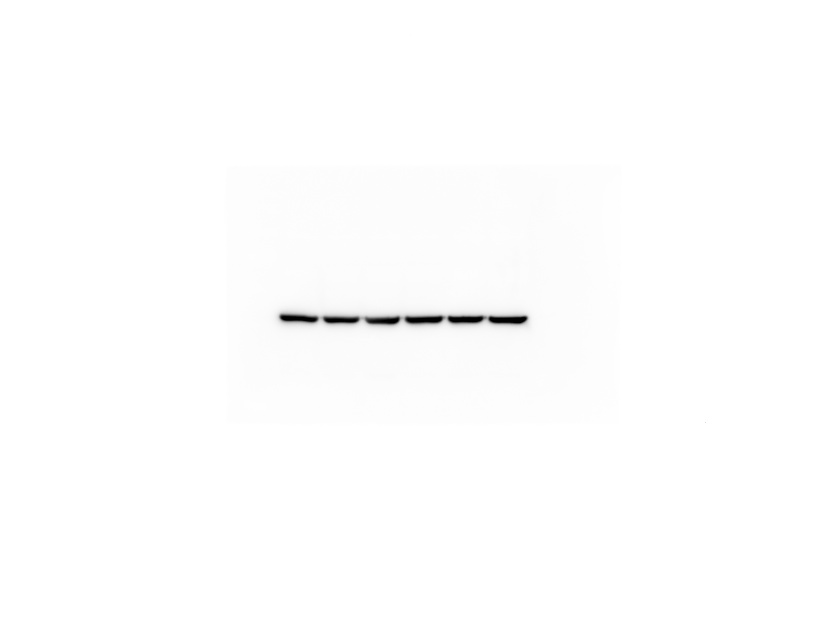

Supplement: Supplementary file 5 — Source data Fig. 1 [file 44318_2024_281_MOESM5_ESM.zip › Figure1/1M/western actin XBB.png]

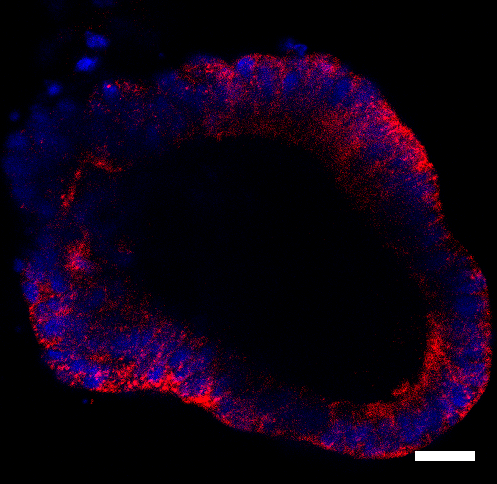

Supplement: Supplementary file 5 — Source data Fig. 1 [file 44318_2024_281_MOESM5_ESM.zip › Figure1/1P/IF LSR Omicron.tif]

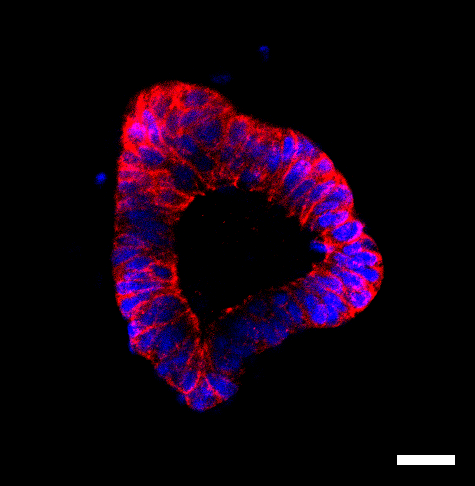

Supplement: Supplementary file 5 — Source data Fig. 1 [file 44318_2024_281_MOESM5_ESM.zip › Figure1/1P/IF LSR VSV-SARS-CoV-2.tif]

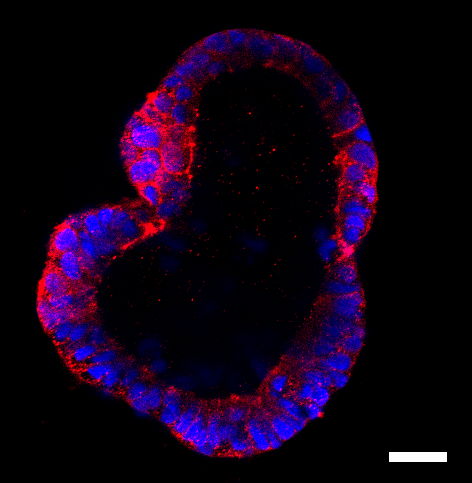

Supplement: Supplementary file 5 — Source data Fig. 1 [file 44318_2024_281_MOESM5_ESM.zip › Figure1/1P/IF LSR WT.tif]

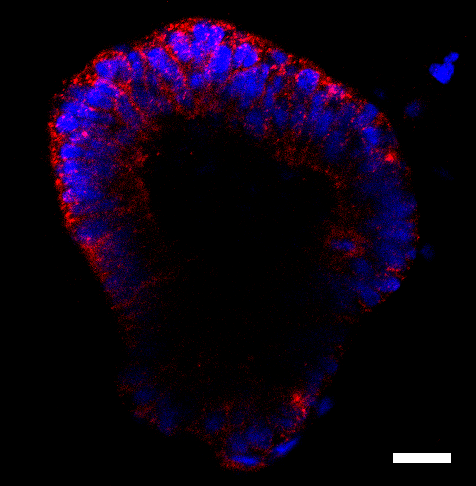

Supplement: Supplementary file 5 — Source data Fig. 1 [file 44318_2024_281_MOESM5_ESM.zip › Figure1/1P/IF LSR XBB.tif]

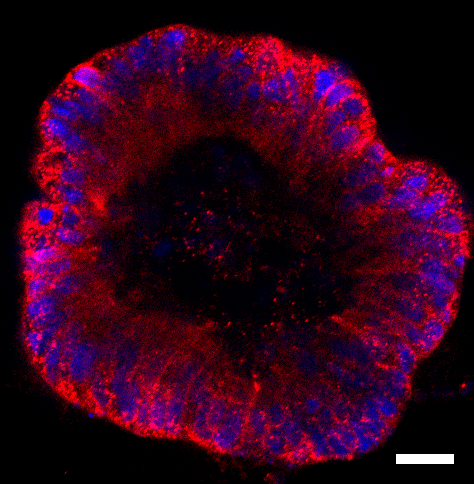

Supplement: Supplementary file 5 — Source data Fig. 1 [file 44318_2024_281_MOESM5_ESM.zip › Figure1/1P/IF LSR control-1.tif]

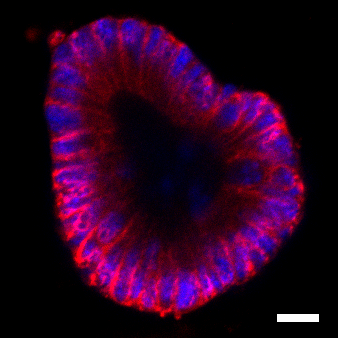

Supplement: Supplementary file 5 — Source data Fig. 1 [file 44318_2024_281_MOESM5_ESM.zip › Figure1/1P/IF LSR control-2.tif]

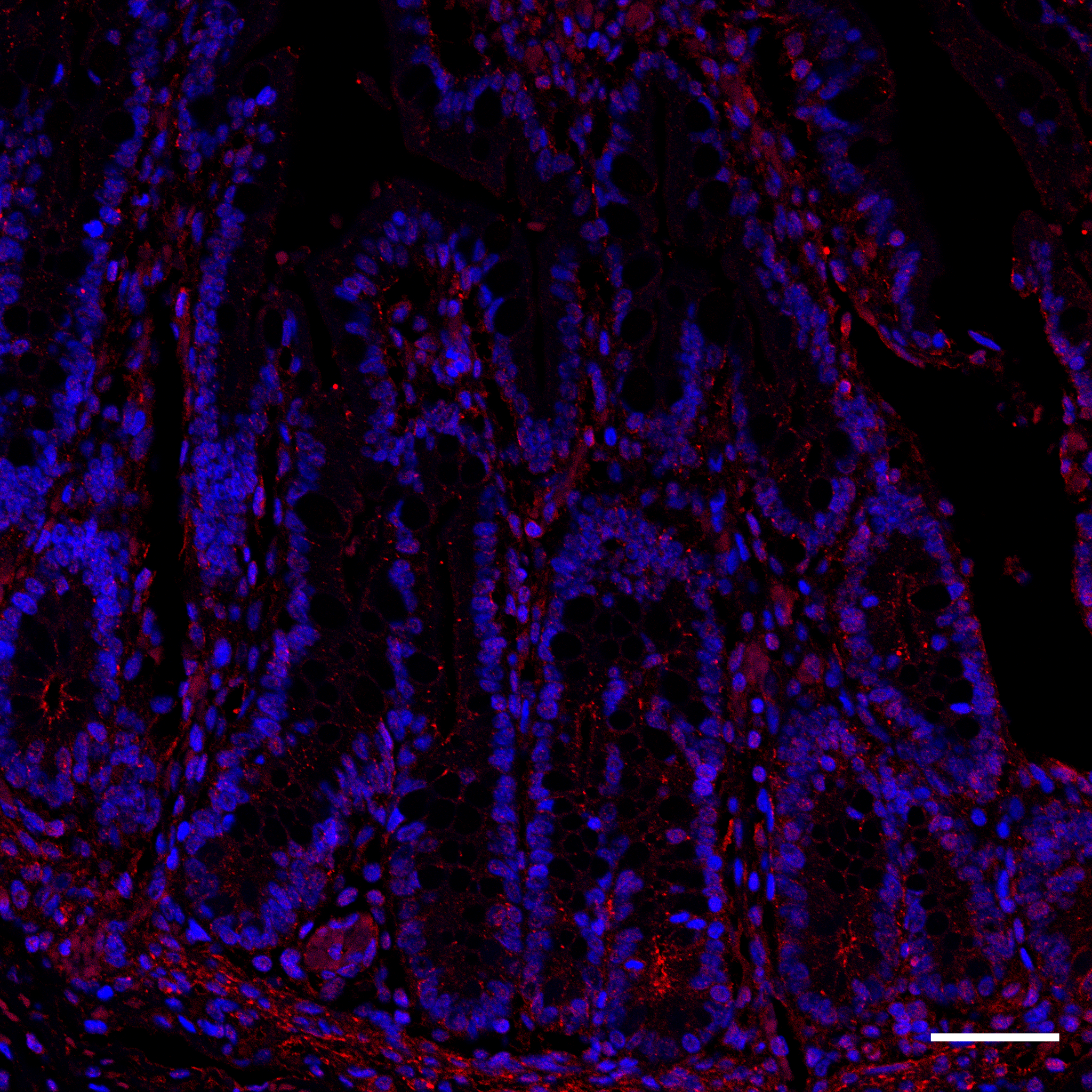

Supplement: Supplementary file 5 — Source data Fig. 1 [file 44318_2024_281_MOESM5_ESM.zip › Figure1/1Q/IF LSR SARS-COV-2.tif]

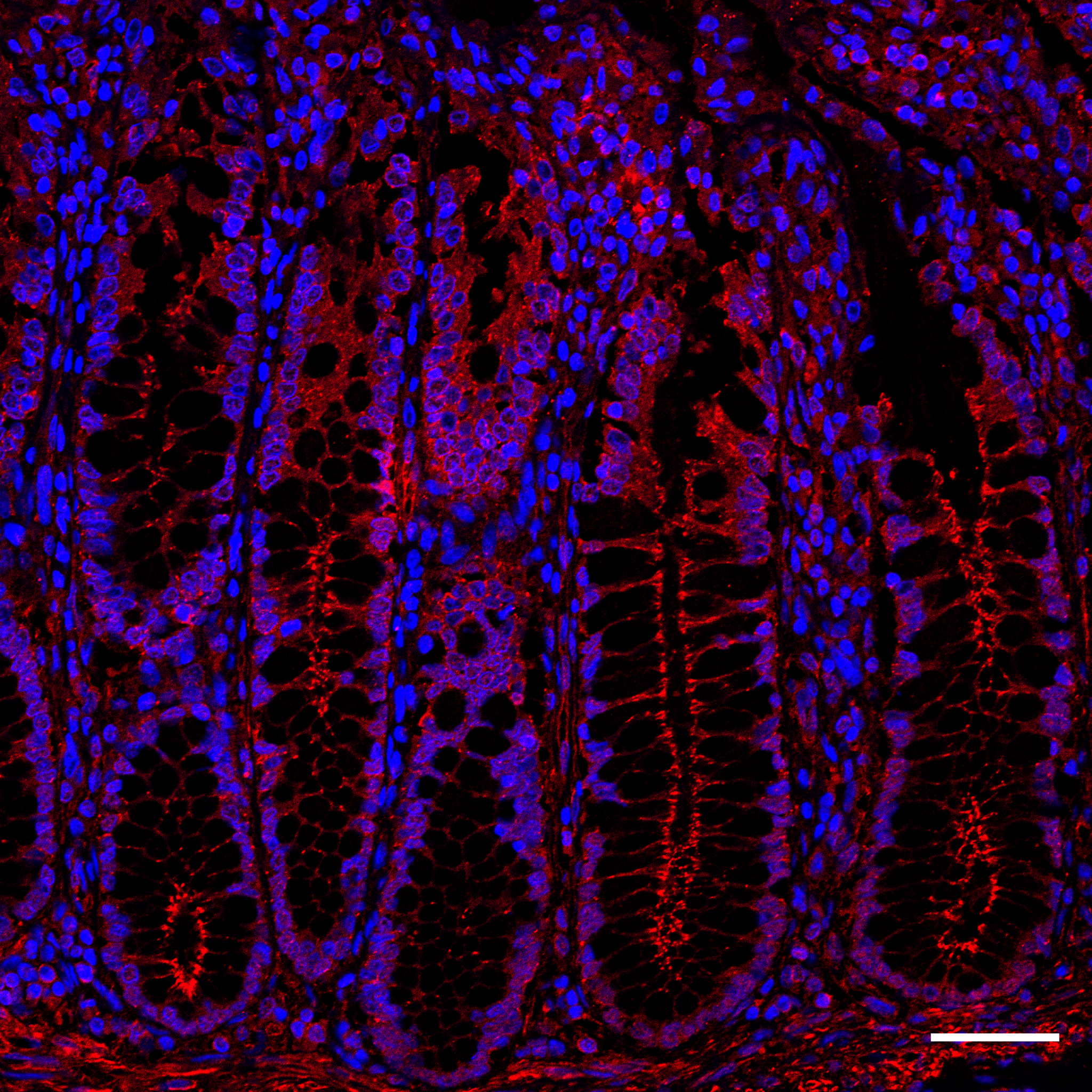

Supplement: Supplementary file 5 — Source data Fig. 1 [file 44318_2024_281_MOESM5_ESM.zip › Figure1/1Q/IF LSR control.tif]

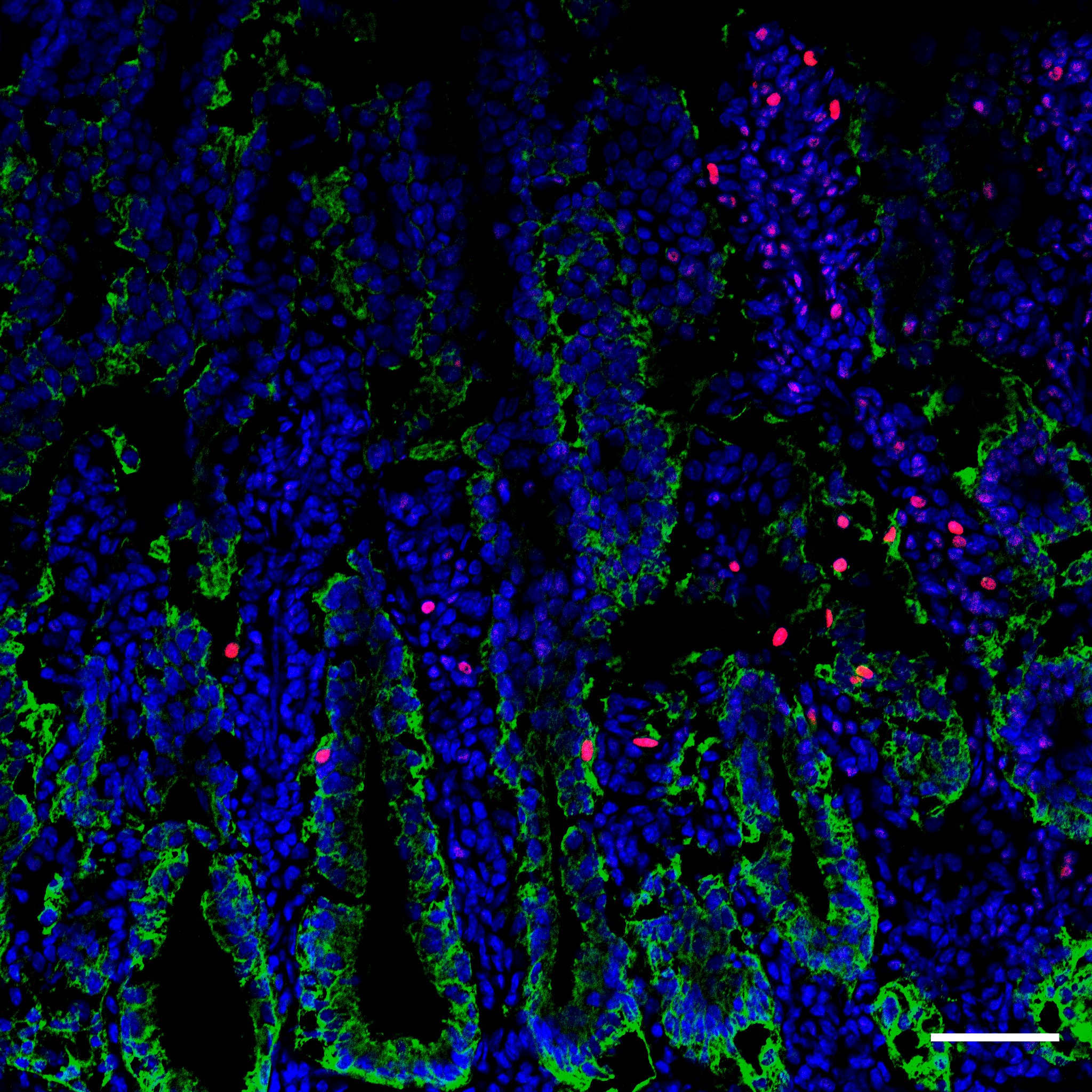

Supplement: Supplementary file 5 — Source data Fig. 1 [file 44318_2024_281_MOESM5_ESM.zip › Figure1/1R/IF PI LSR VSV-SARS-CoV-2.tif]

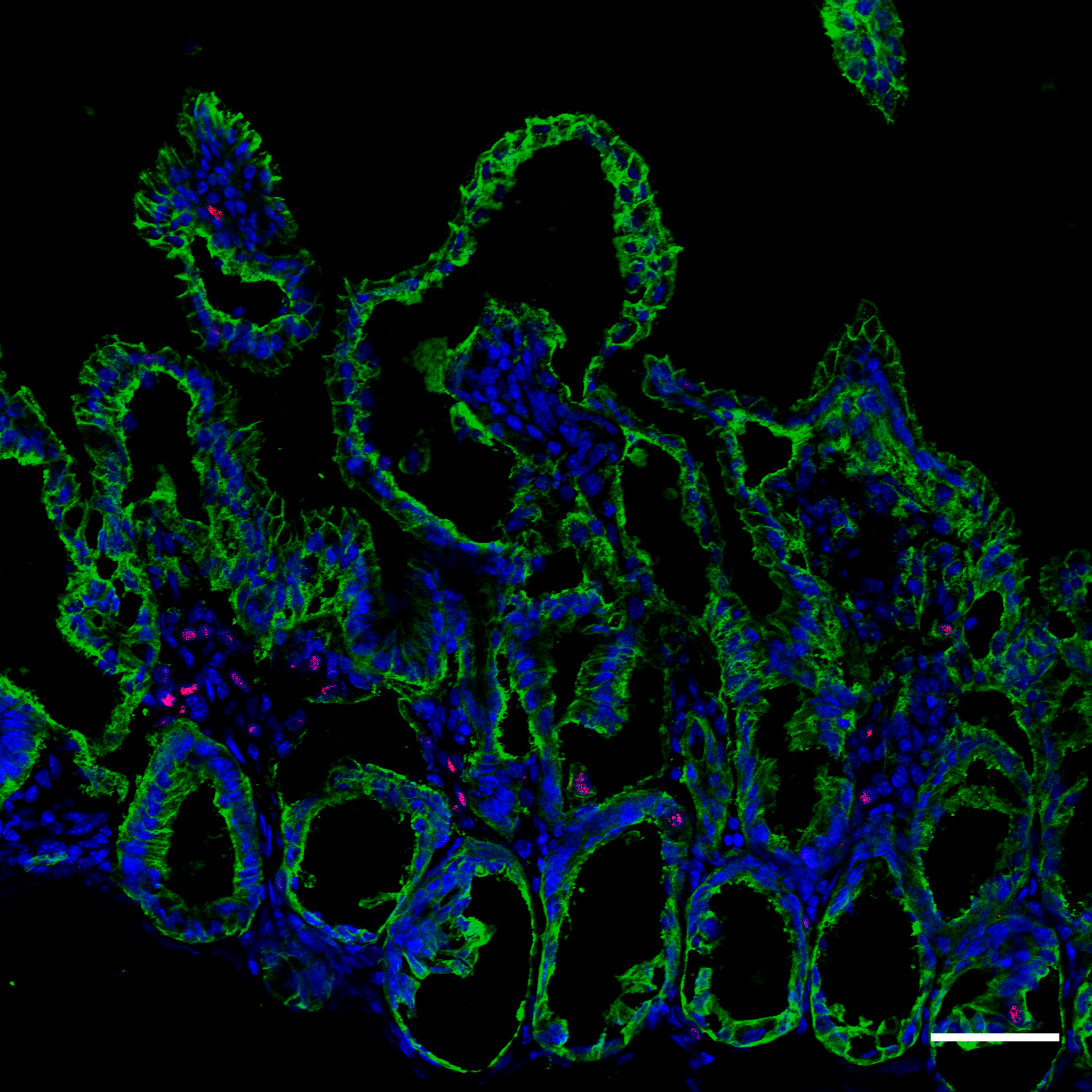

Supplement: Supplementary file 5 — Source data Fig. 1 [file 44318_2024_281_MOESM5_ESM.zip › Figure1/1R/IF PI LSR control.tif]

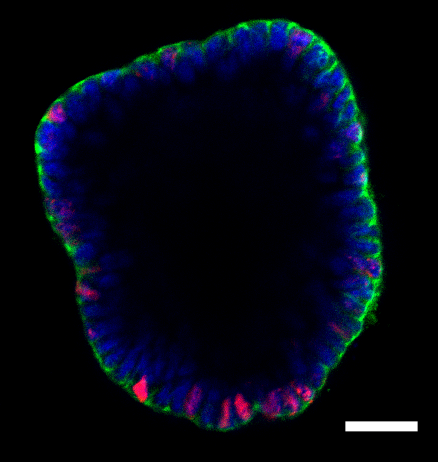

Supplement: Supplementary file 5 — Source data Fig. 1 [file 44318_2024_281_MOESM5_ESM.zip › Figure1/1S/IF PI LSR SARS-CoV-2 WT.tif]

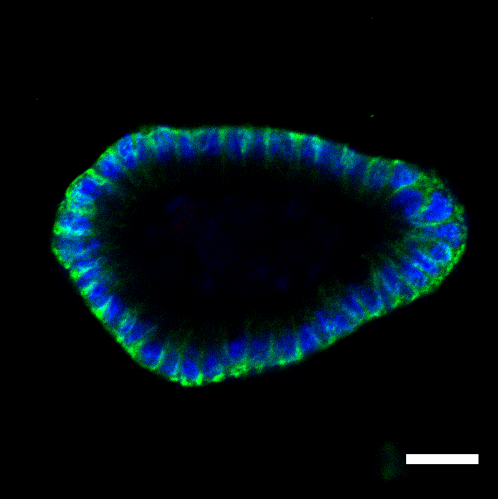

Supplement: Supplementary file 5 — Source data Fig. 1 [file 44318_2024_281_MOESM5_ESM.zip › Figure1/1S/IF PI LSR control.tif]

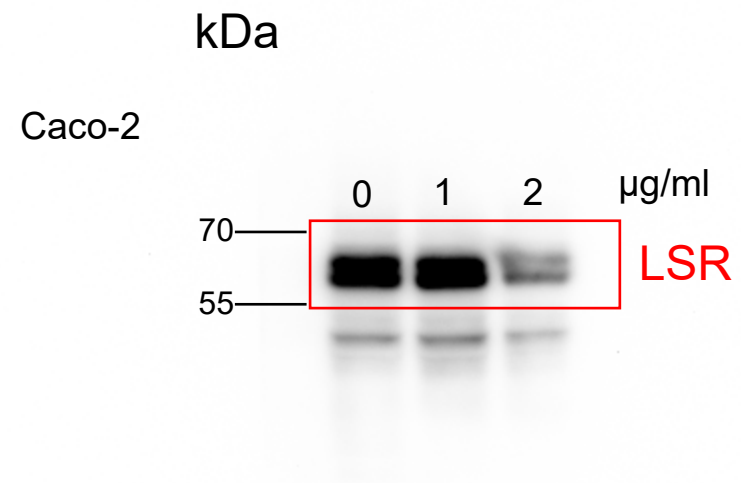

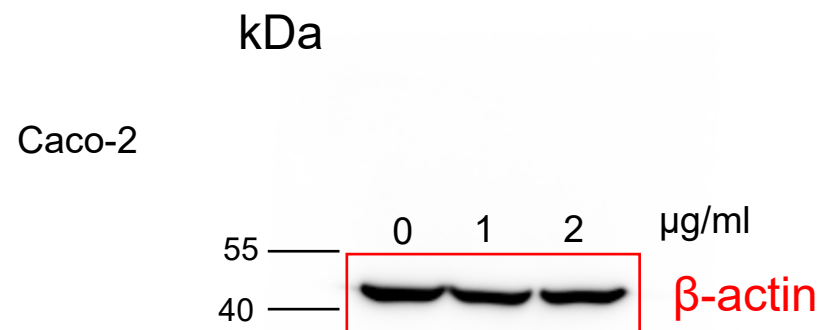

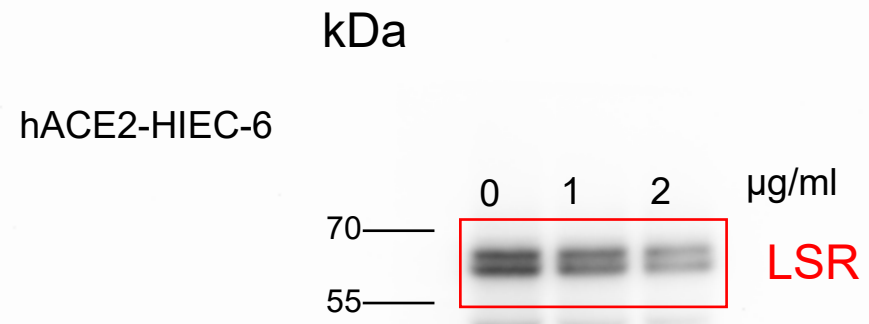

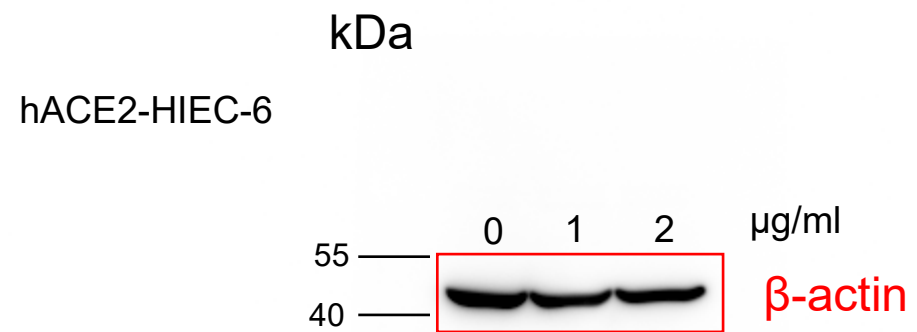

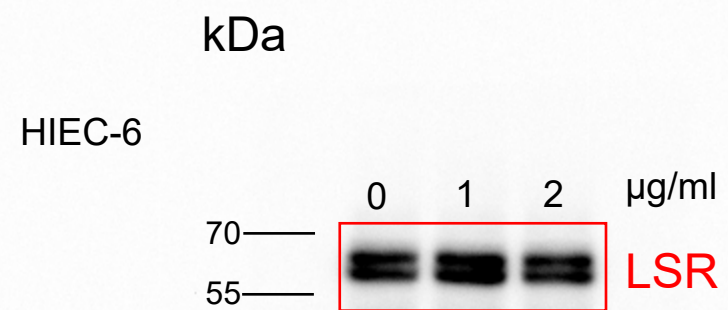

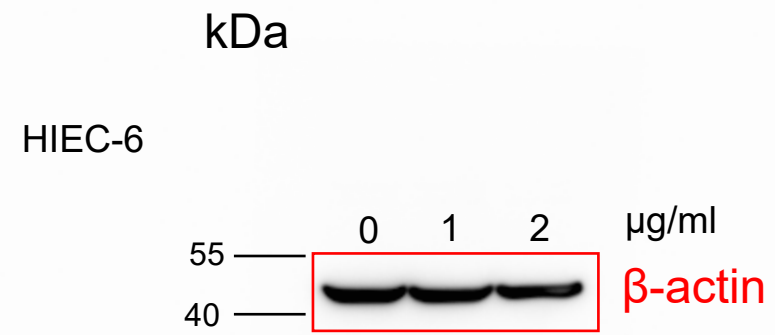

Supplement: Supplementary file 6 — Source data Fig. 2 [file 44318_2024_281_MOESM6_ESM.zip › Figure2/2A/2A.pdf]

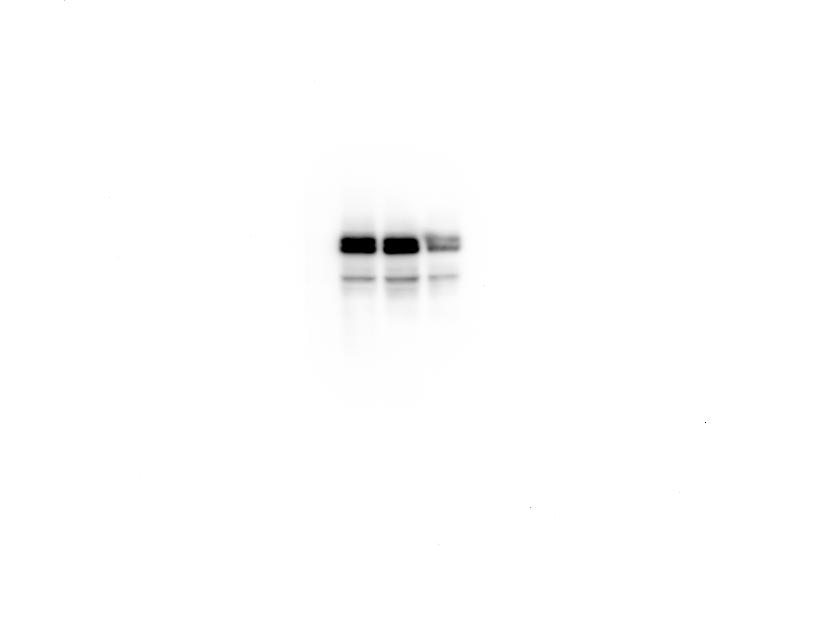

Supplement: Supplementary file 6 — Source data Fig. 2 [file 44318_2024_281_MOESM6_ESM.zip › Figure2/2A/western LSR CACO-2.png]

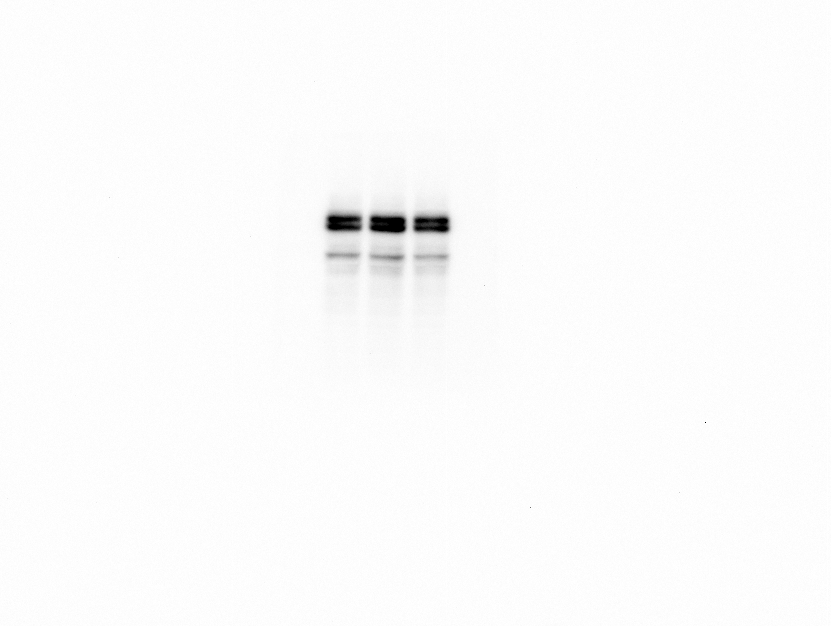

Supplement: Supplementary file 6 — Source data Fig. 2 [file 44318_2024_281_MOESM6_ESM.zip › Figure2/2A/western LSR HIEC-6.png]

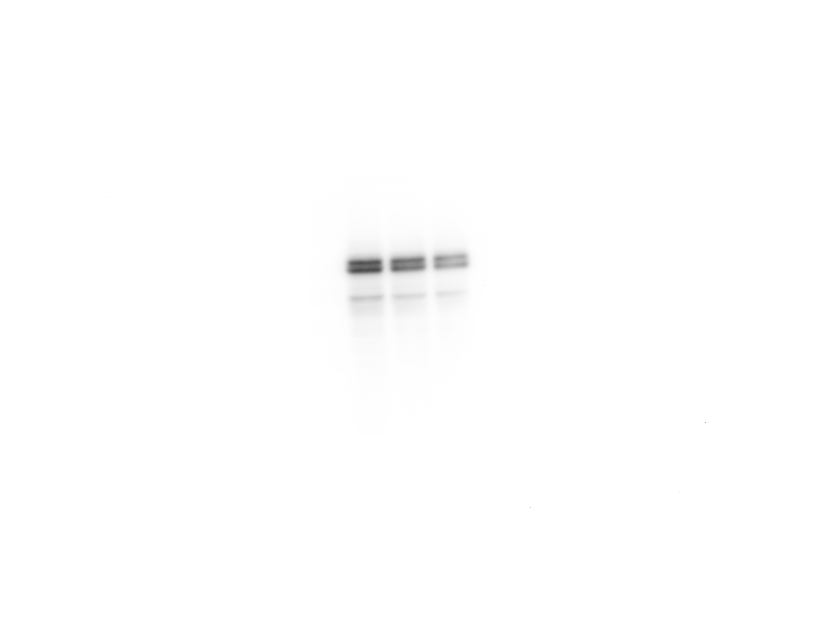

Supplement: Supplementary file 6 — Source data Fig. 2 [file 44318_2024_281_MOESM6_ESM.zip › Figure2/2A/western LSR hACE2-HIEC-6.png]

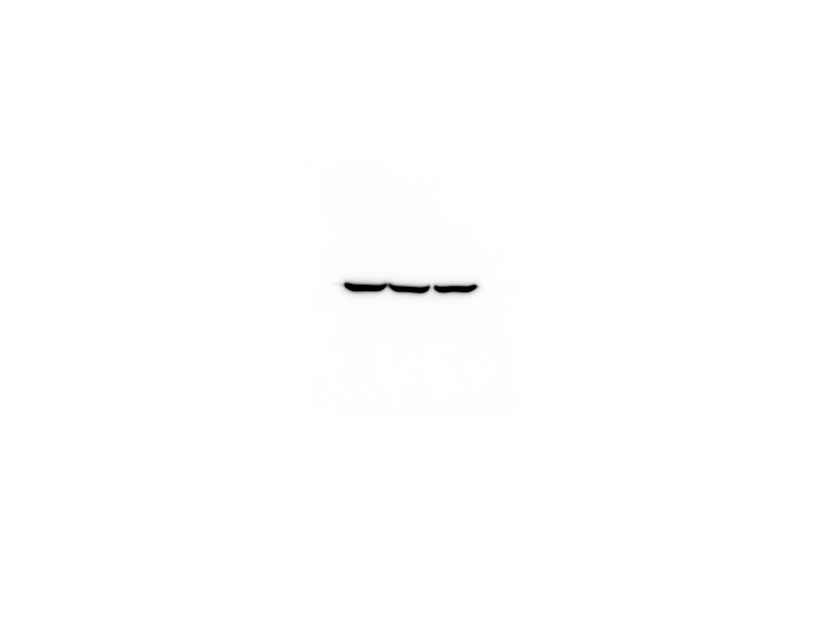

Supplement: Supplementary file 6 — Source data Fig. 2 [file 44318_2024_281_MOESM6_ESM.zip › Figure2/2A/western actin CACO-2.png]

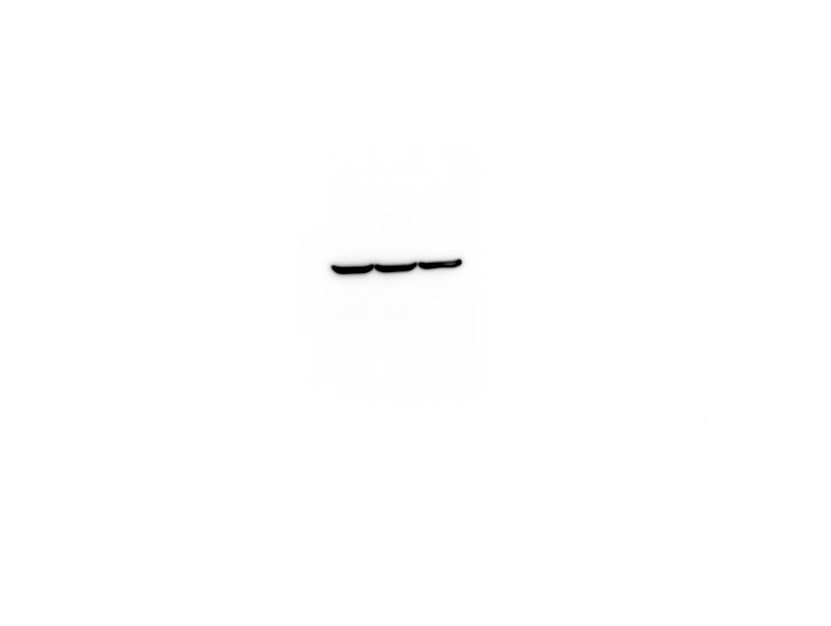

Supplement: Supplementary file 6 — Source data Fig. 2 [file 44318_2024_281_MOESM6_ESM.zip › Figure2/2A/western actin HIEC-6.png]

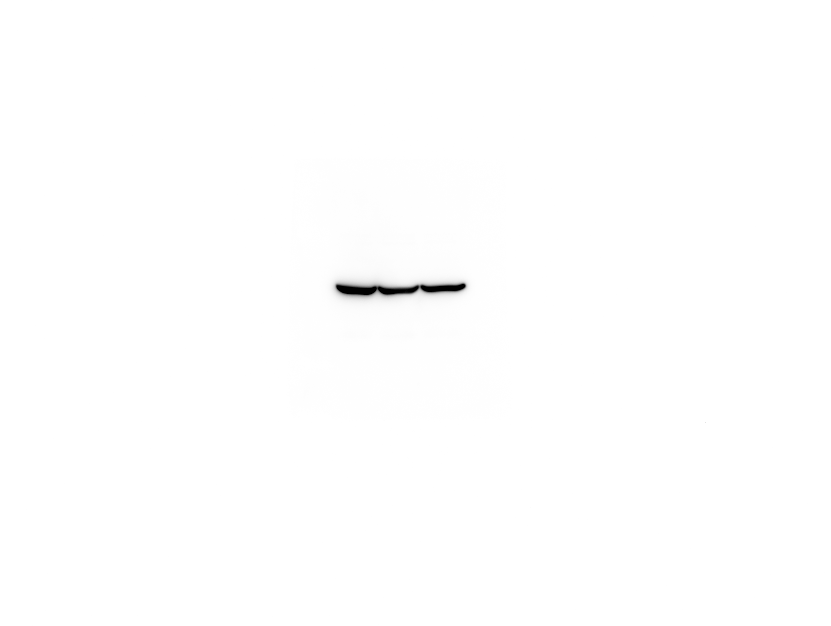

Supplement: Supplementary file 6 — Source data Fig. 2 [file 44318_2024_281_MOESM6_ESM.zip › Figure2/2A/western actin hACE2-HIEC-6.png]

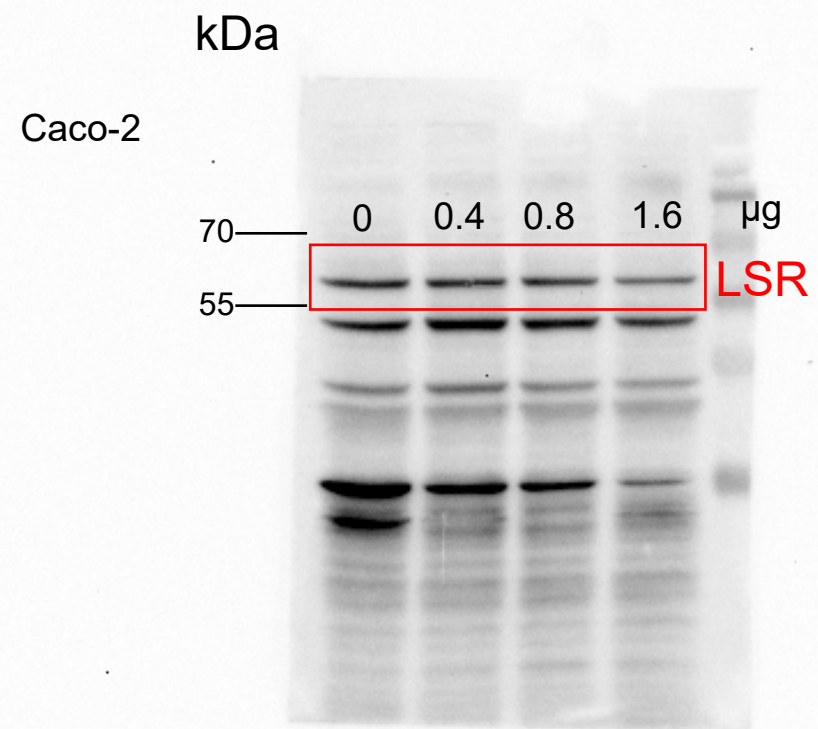

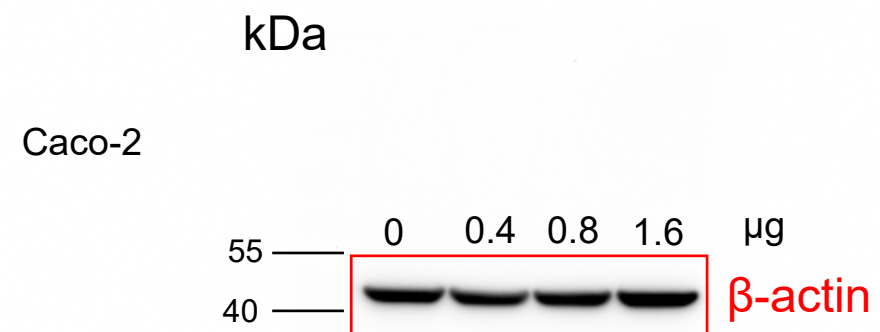

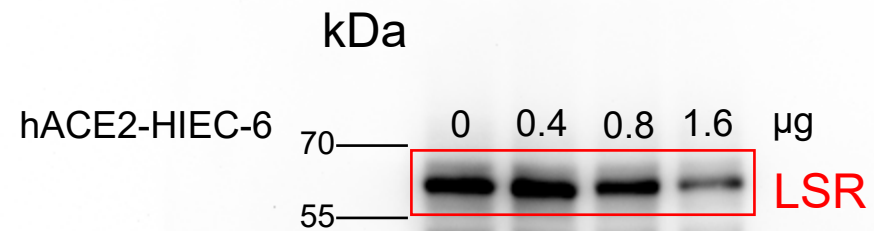

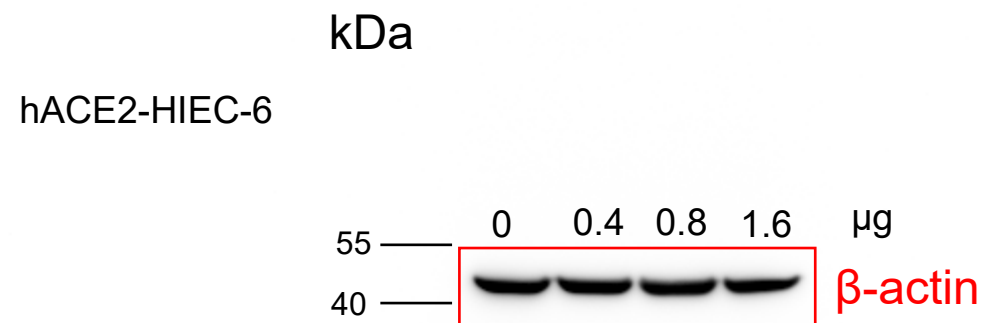

Supplement: Supplementary file 6 — Source data Fig. 2 [file 44318_2024_281_MOESM6_ESM.zip › Figure2/2B/2B.pdf]

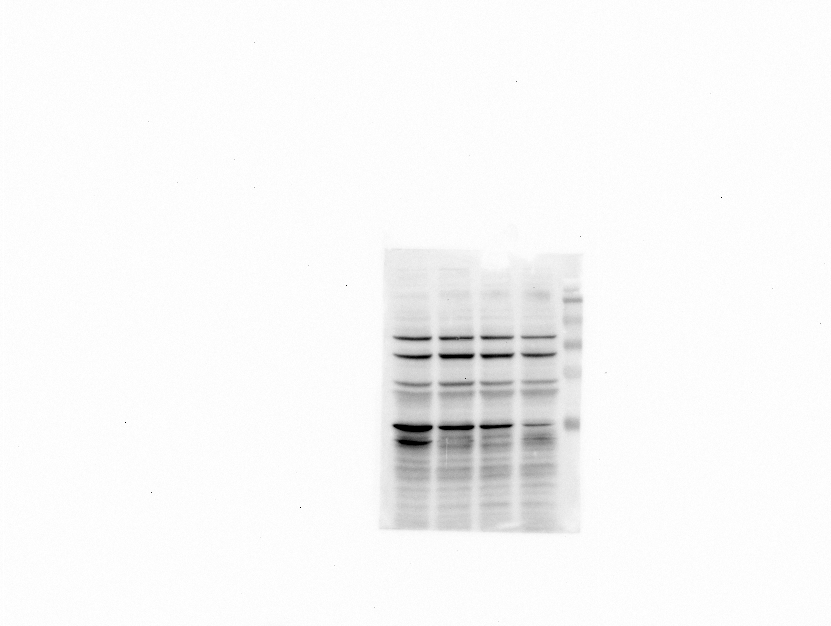

Supplement: Supplementary file 6 — Source data Fig. 2 [file 44318_2024_281_MOESM6_ESM.zip › Figure2/2B/western LSR Caco-2.png]

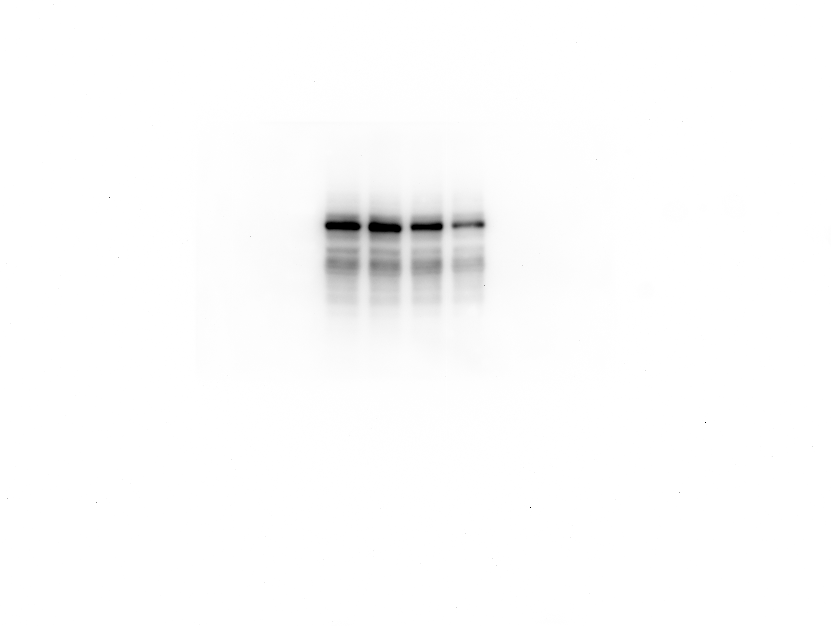

Supplement: Supplementary file 6 — Source data Fig. 2 [file 44318_2024_281_MOESM6_ESM.zip › Figure2/2B/western LSR hACE2-HIEC-6.png]

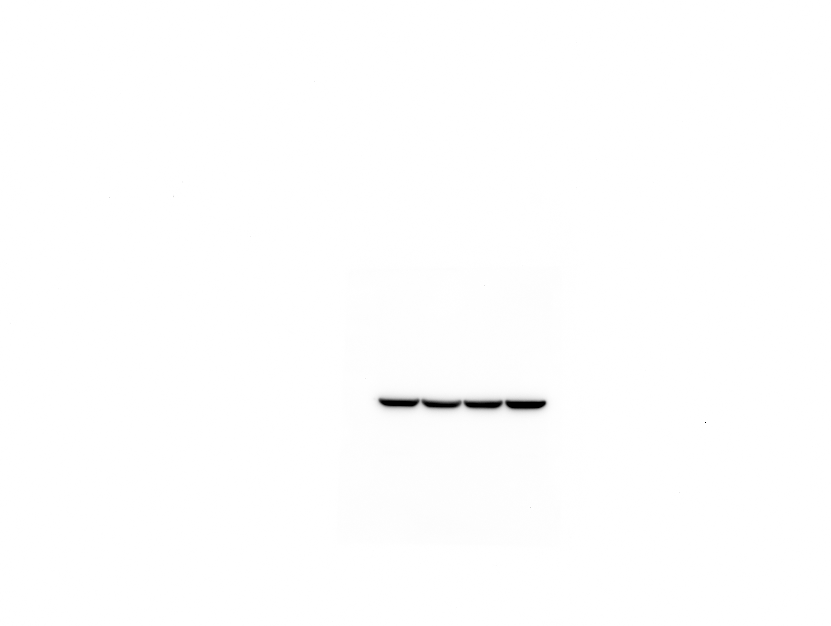

Supplement: Supplementary file 6 — Source data Fig. 2 [file 44318_2024_281_MOESM6_ESM.zip › Figure2/2B/western actin Caco-2.png]

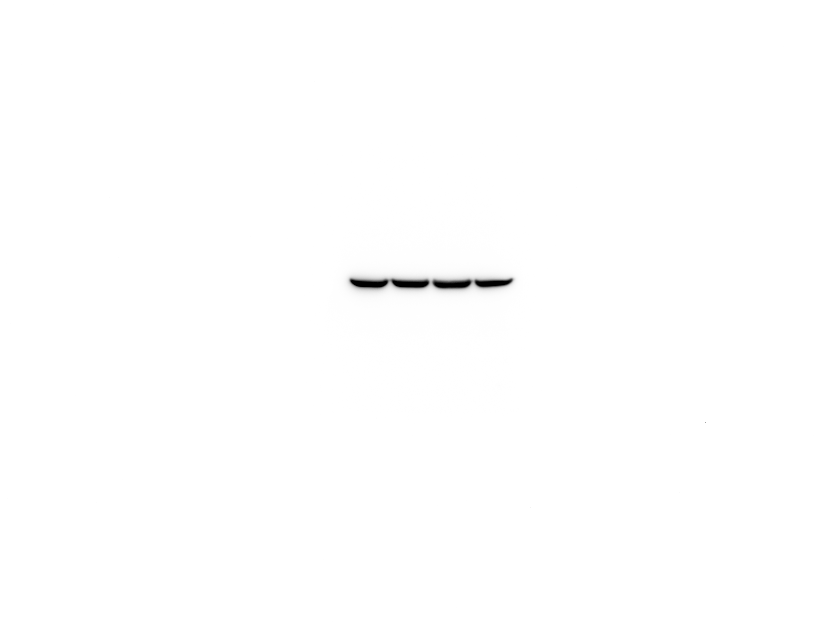

Supplement: Supplementary file 6 — Source data Fig. 2 [file 44318_2024_281_MOESM6_ESM.zip › Figure2/2B/western actin hACE2-HIEC-6.png]

kDa

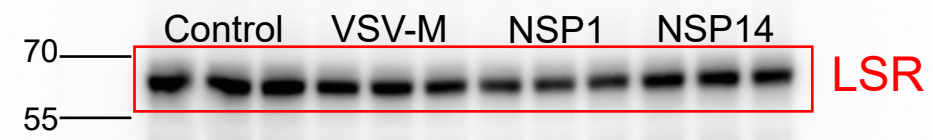

kDa

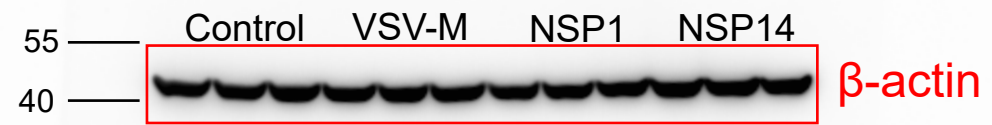

Supplement: Supplementary file 6 — Source data Fig. 2 [file 44318_2024_281_MOESM6_ESM.zip › Figure2/2D/2D.pdf]

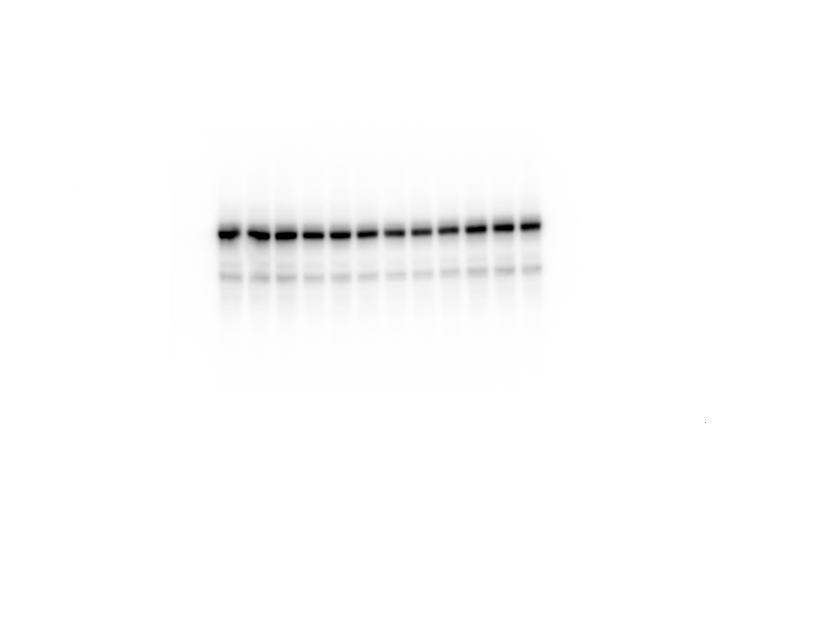

Supplement: Supplementary file 6 — Source data Fig. 2 [file 44318_2024_281_MOESM6_ESM.zip › Figure2/2D/western LSR.png]

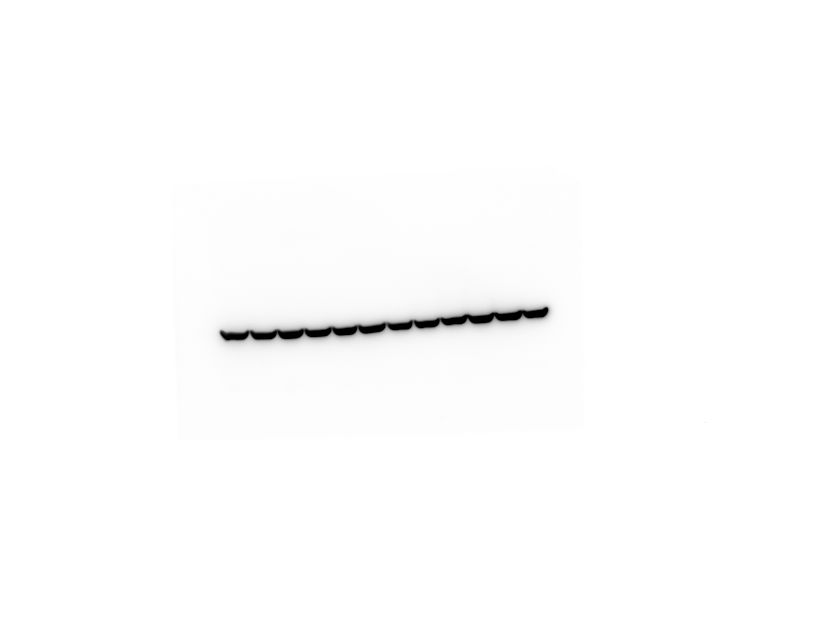

Supplement: Supplementary file 6 — Source data Fig. 2 [file 44318_2024_281_MOESM6_ESM.zip › Figure2/2D/western actin.png]

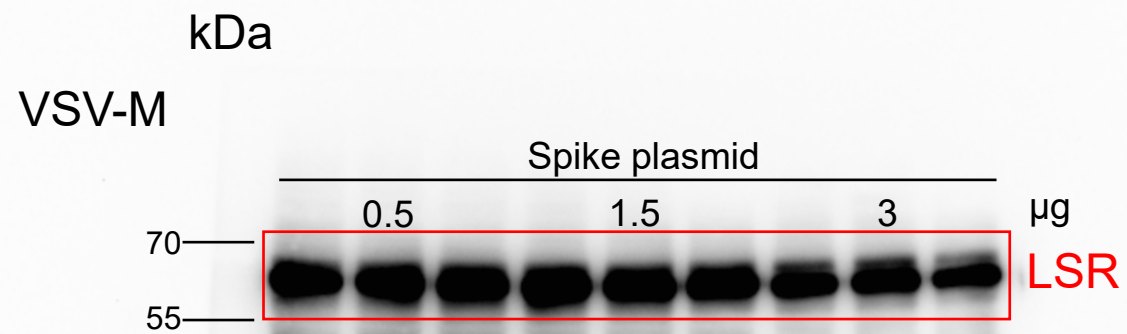

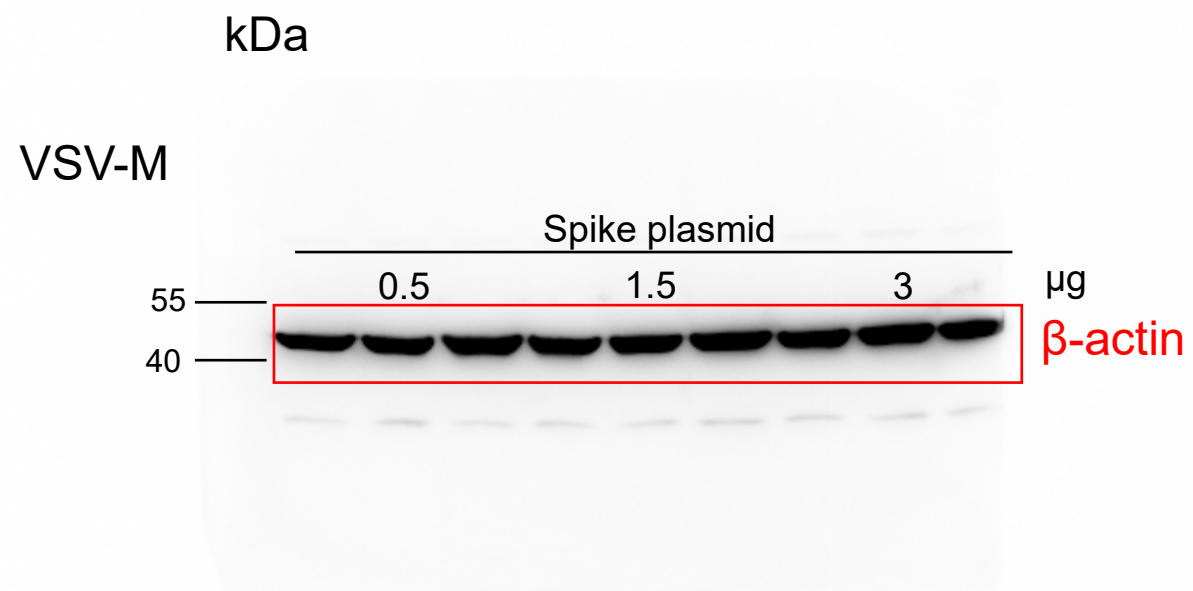

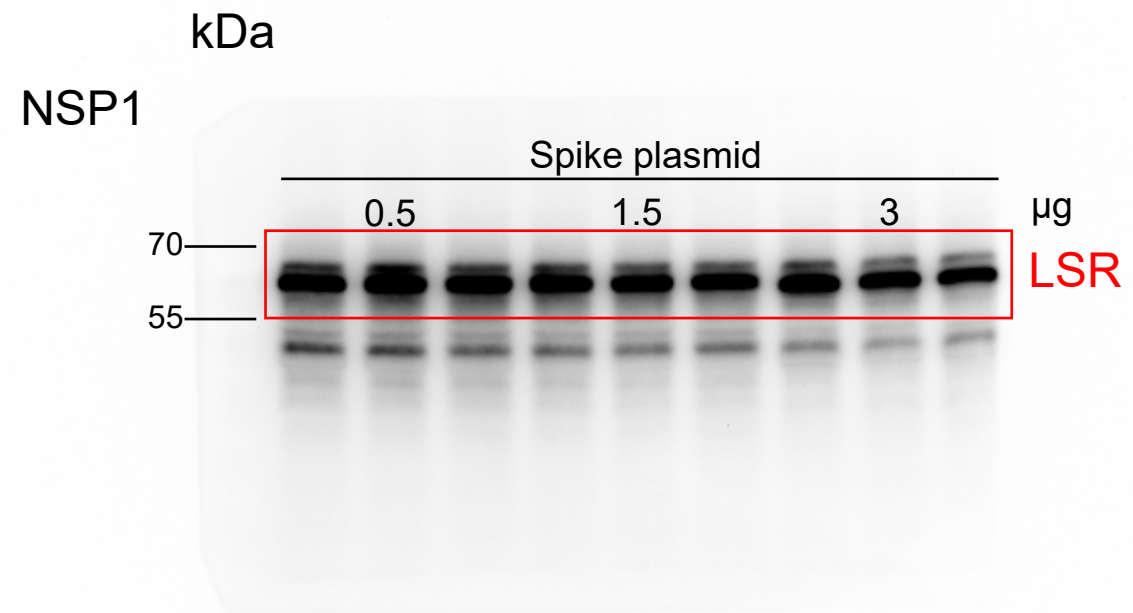

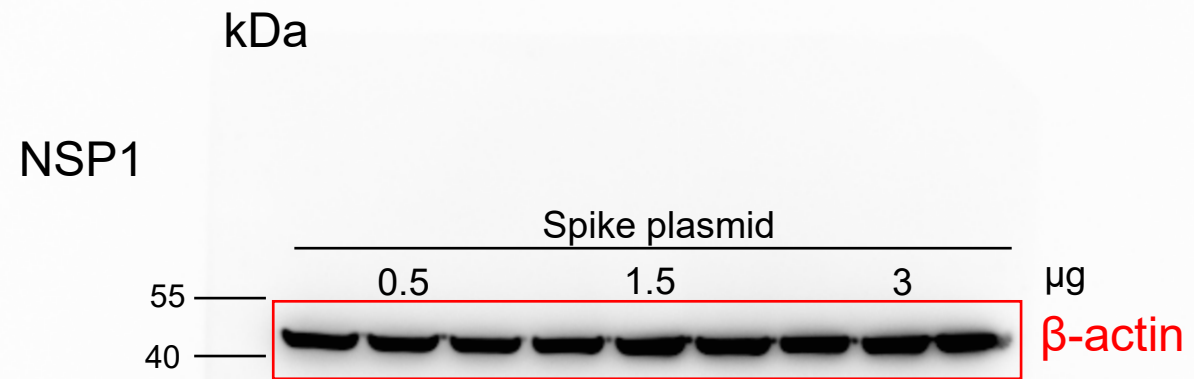

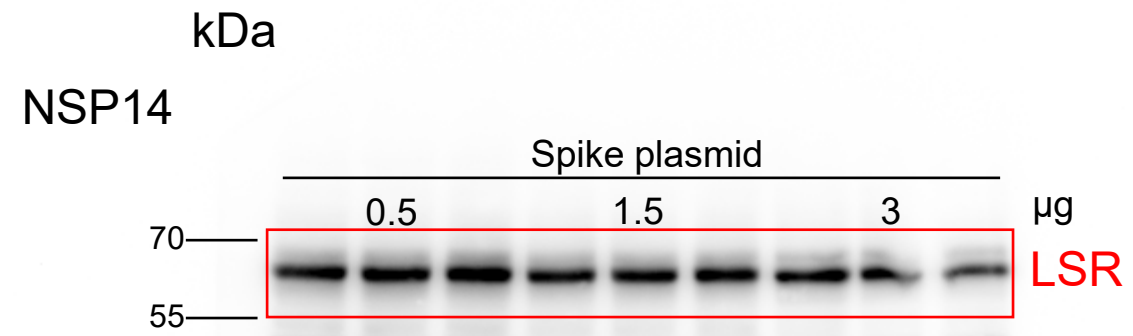

kDa

NSP14

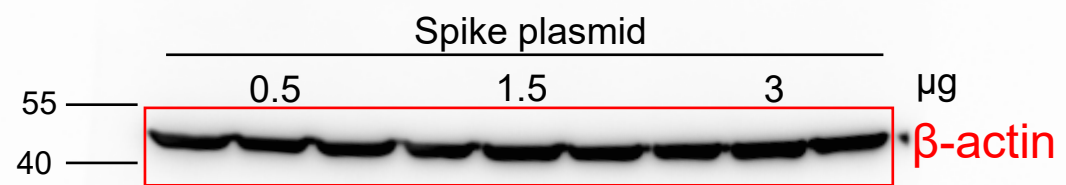

Supplement: Supplementary file 6 — Source data Fig. 2 [file 44318_2024_281_MOESM6_ESM.zip › Figure2/2E/2E.pdf]

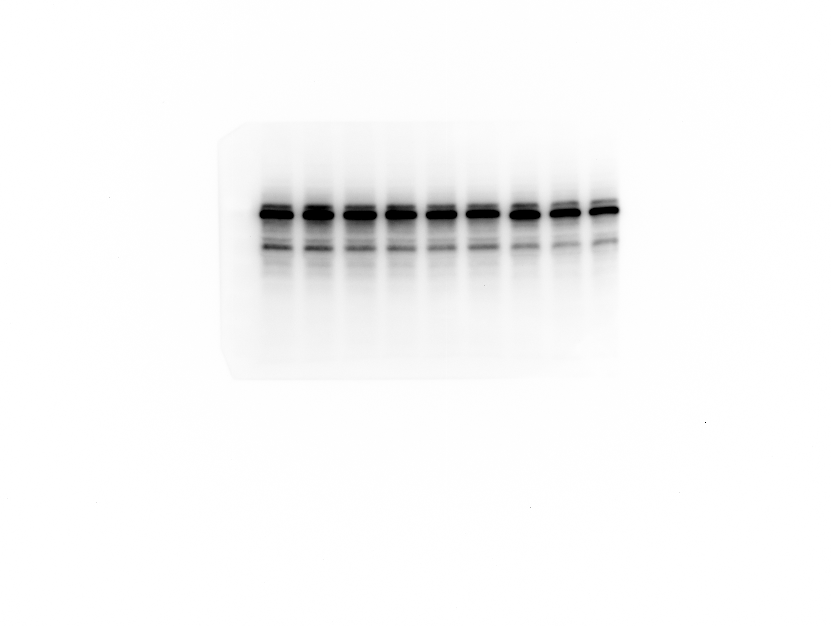

Supplement: Supplementary file 6 — Source data Fig. 2 [file 44318_2024_281_MOESM6_ESM.zip › Figure2/2E/western LSR NSP1.png]

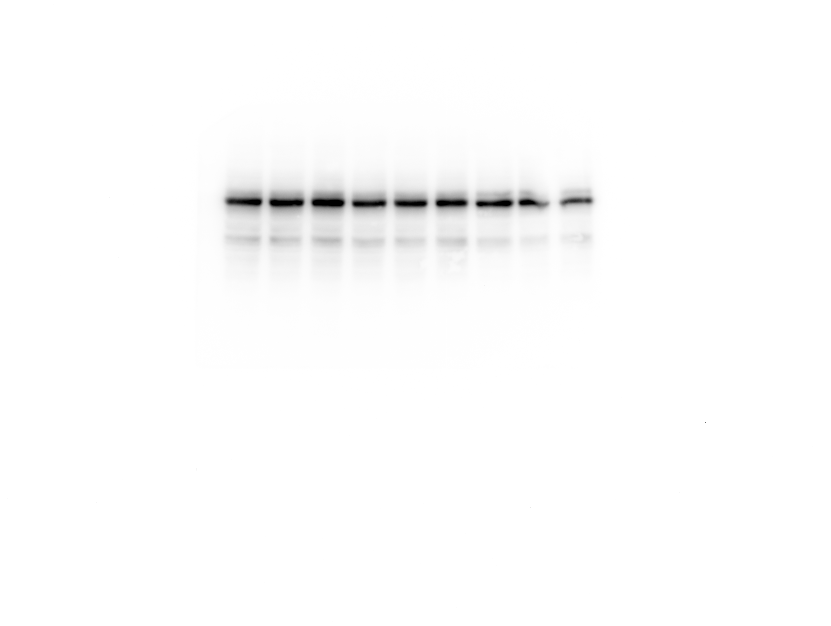

Supplement: Supplementary file 6 — Source data Fig. 2 [file 44318_2024_281_MOESM6_ESM.zip › Figure2/2E/western LSR NSP14.png]

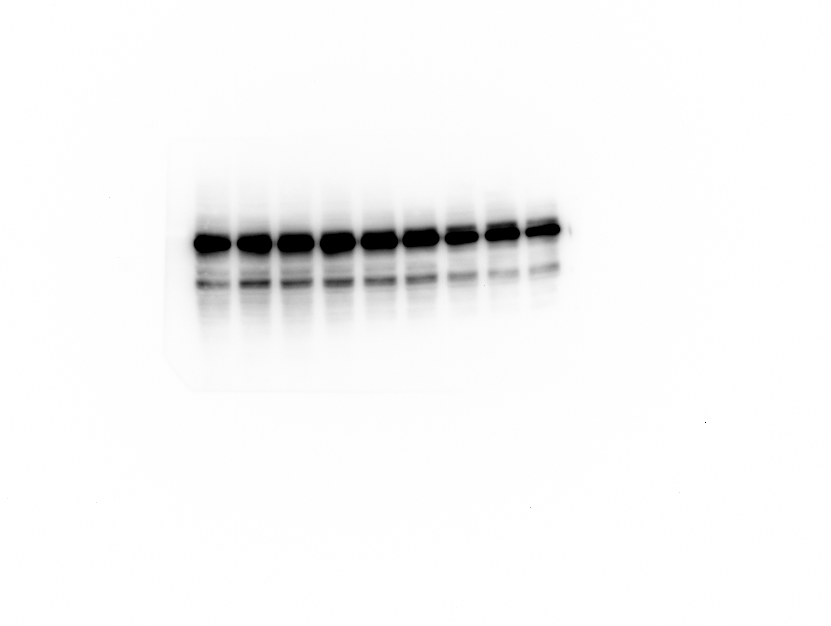

Supplement: Supplementary file 6 — Source data Fig. 2 [file 44318_2024_281_MOESM6_ESM.zip › Figure2/2E/western LSR VSV-M.png]

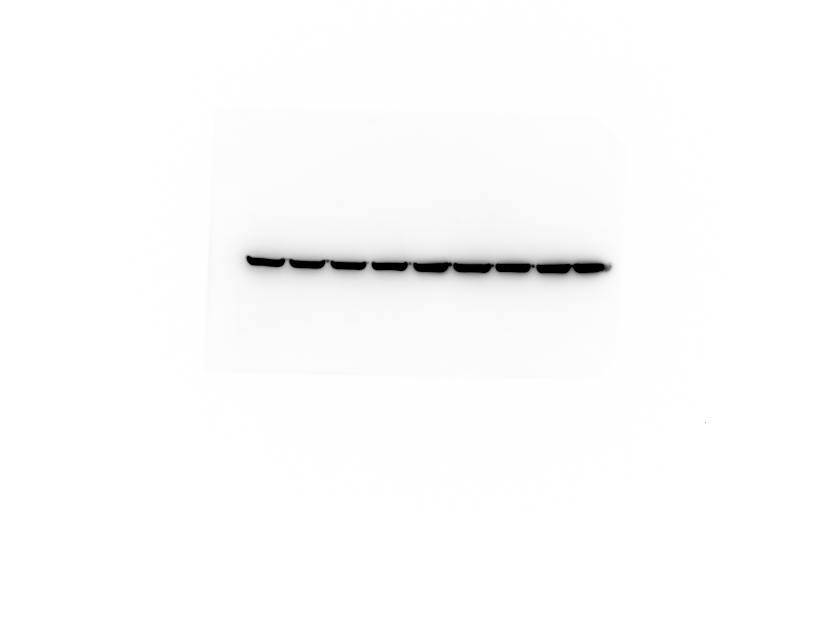

Supplement: Supplementary file 6 — Source data Fig. 2 [file 44318_2024_281_MOESM6_ESM.zip › Figure2/2E/western actin NSP1.png]

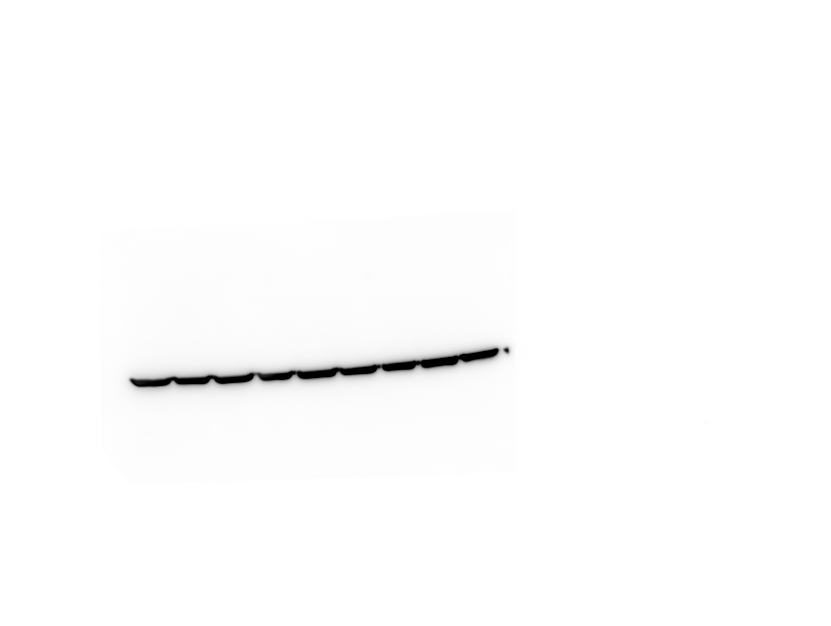

Supplement: Supplementary file 6 — Source data Fig. 2 [file 44318_2024_281_MOESM6_ESM.zip › Figure2/2E/western actin NSP14.png]

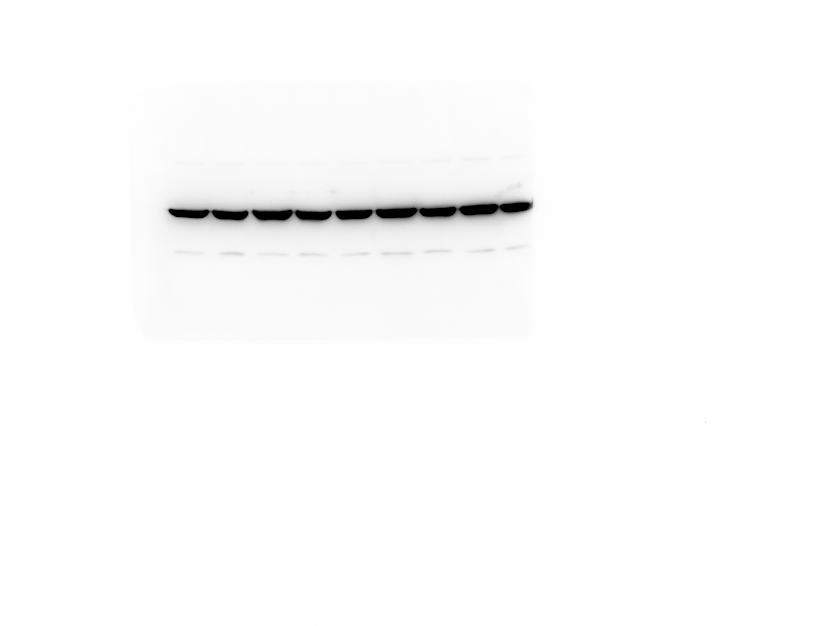

Supplement: Supplementary file 6 — Source data Fig. 2 [file 44318_2024_281_MOESM6_ESM.zip › Figure2/2E/western actin VSV-M.png]

kDa

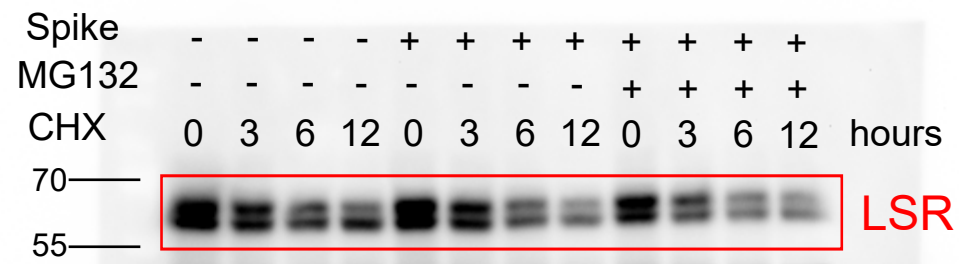

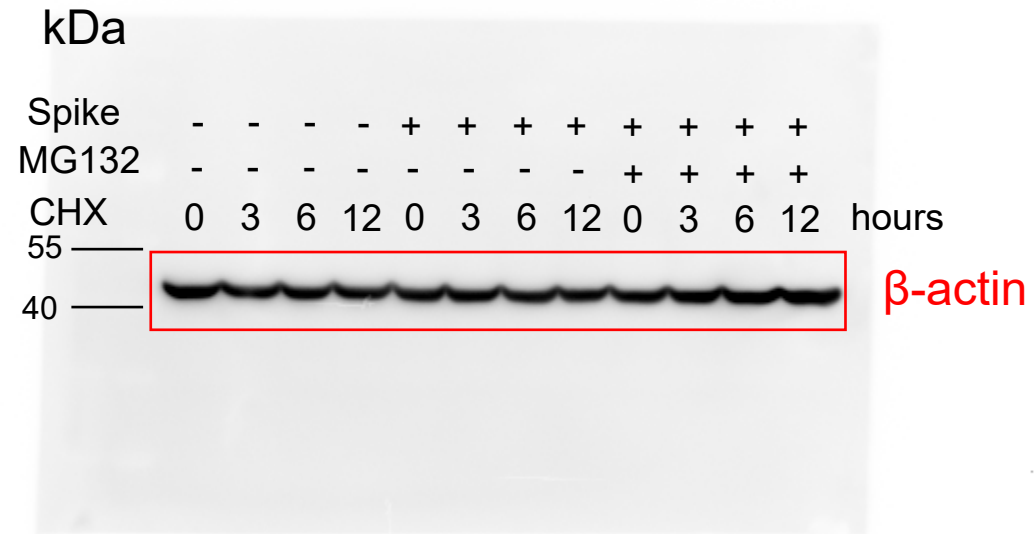

Supplement: Supplementary file 6 — Source data Fig. 2 [file 44318_2024_281_MOESM6_ESM.zip › Figure2/2F/2F.pdf]

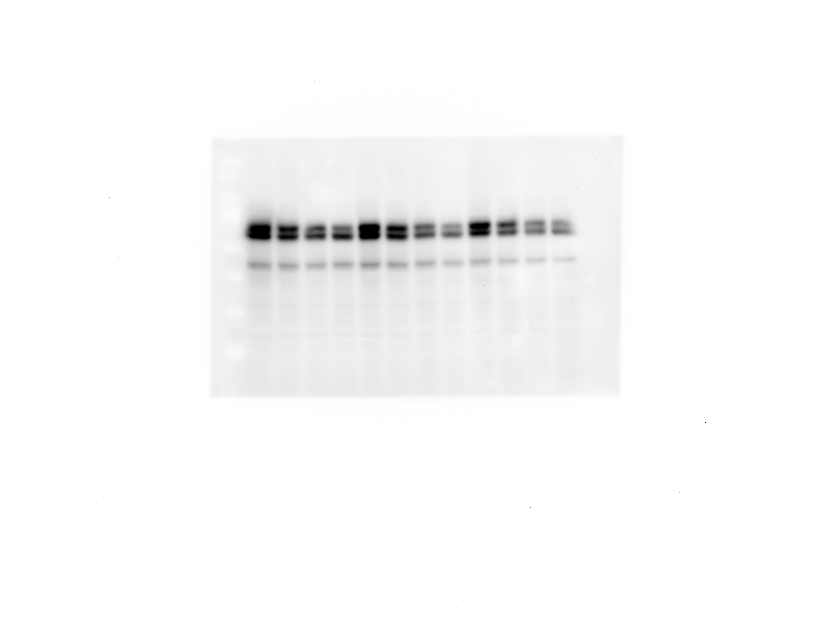

Supplement: Supplementary file 6 — Source data Fig. 2 [file 44318_2024_281_MOESM6_ESM.zip › Figure2/2F/western LSR.png]

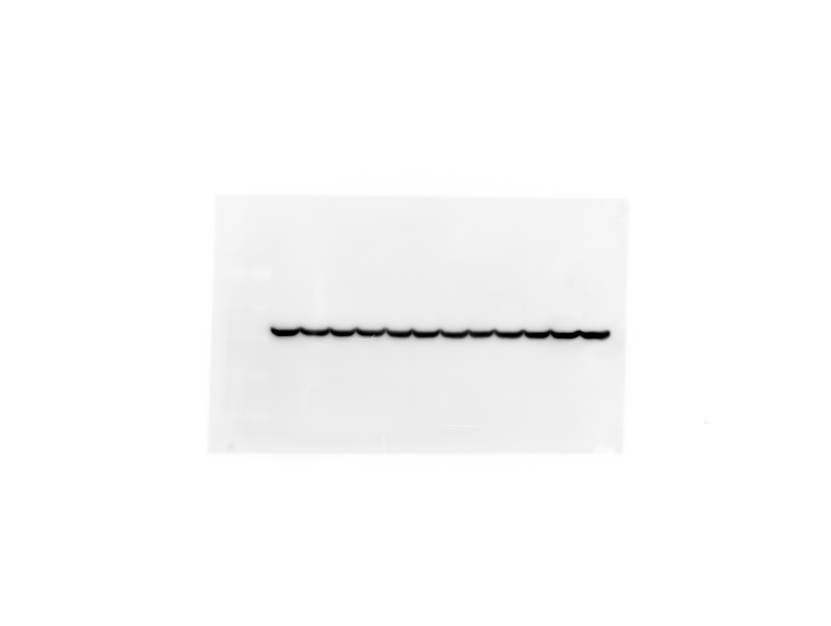

Supplement: Supplementary file 6 — Source data Fig. 2 [file 44318_2024_281_MOESM6_ESM.zip › Figure2/2F/western actin.png]

kDa

|        |   |   |   |    |   |   |   |    |   |   |   |    |       |
|--------|---|---|---|----|---|---|---|----|---|---|---|----|-------|
| Spike  | - | - | - | -  | + | + | + | +  | + | + | + | +  |       |
| Baf-A1 | - | - | - | -  | - | - | - | -  | + | + | + | +  |       |
| CHX    | 0 | 3 | 6 | 12 | 0 | 3 | 6 | 12 | 0 | 3 | 6 | 12 | hours |

70—  
55—

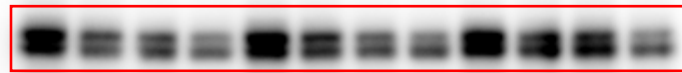

LSR

kDa

|        |       |   |   |    |   |   |   |    |   |   |   |    |
|--------|-------|---|---|----|---|---|---|----|---|---|---|----|
| Spike  | -     | - | - | -  | + | + | + | +  | + | + | + | +  |
| Baf-A1 | -     | - | - | -  | - | - | - | -  | + | + | + | +  |
| CHX    | 0     | 3 | 6 | 12 | 0 | 3 | 6 | 12 | 0 | 3 | 6 | 12 |
|        | hours |   |   |    |   |   |   |    |   |   |   |    |

55 —  
40 —

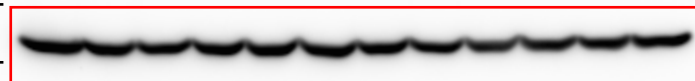

$\beta$ -actin

Supplement: Supplementary file 6 — Source data Fig. 2 [file 44318_2024_281_MOESM6_ESM.zip › Figure2/2G/2G.pdf]

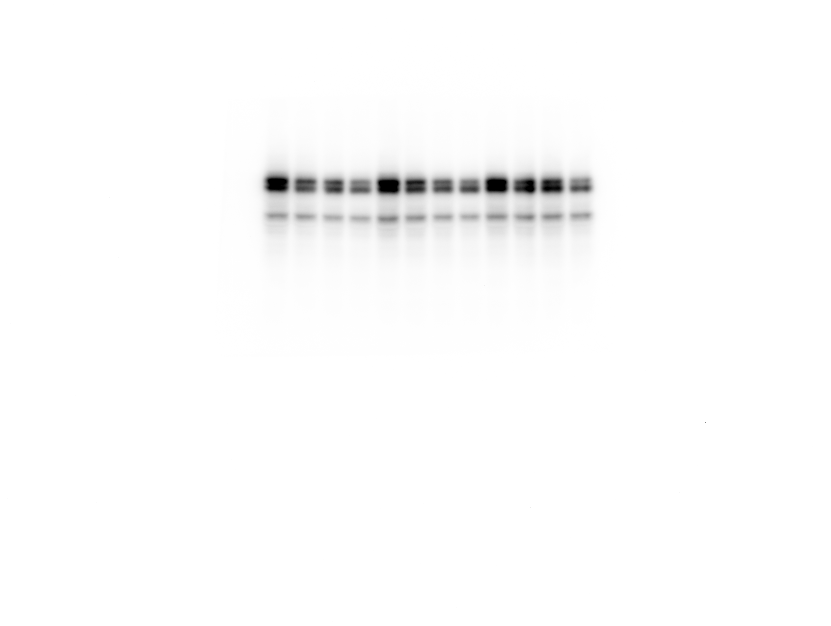

Supplement: Supplementary file 6 — Source data Fig. 2 [file 44318_2024_281_MOESM6_ESM.zip › Figure2/2G/western LSR.png]

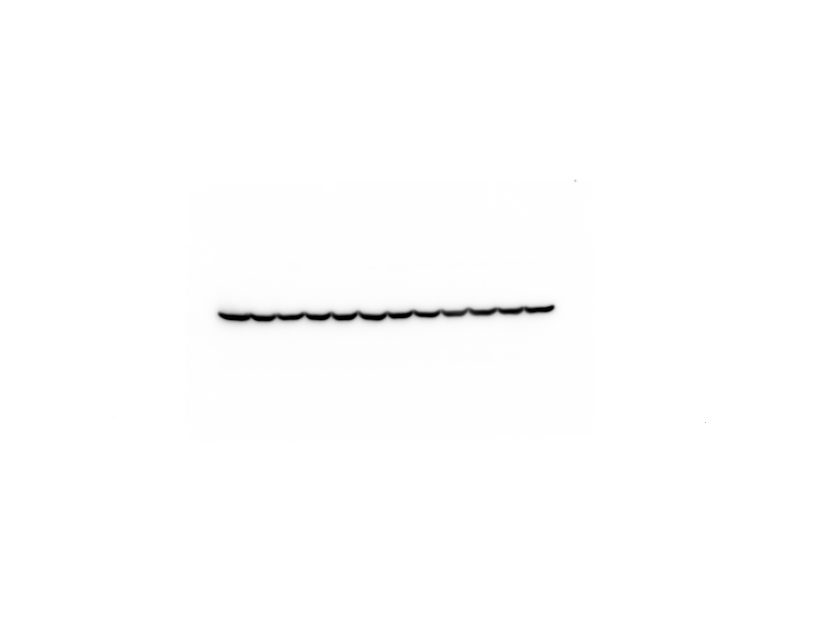

Supplement: Supplementary file 6 — Source data Fig. 2 [file 44318_2024_281_MOESM6_ESM.zip › Figure2/2G/western actin.png]

kDa

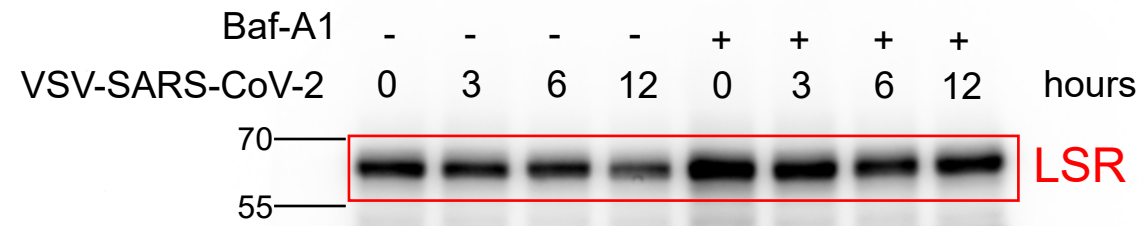

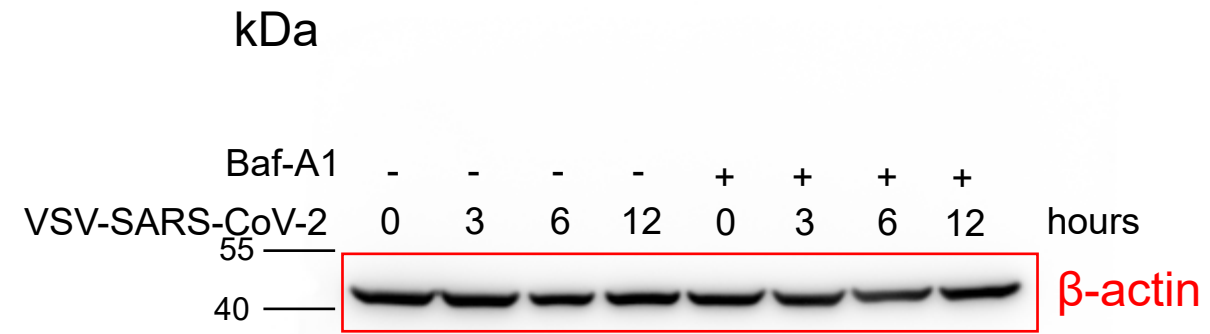

Supplement: Supplementary file 6 — Source data Fig. 2 [file 44318_2024_281_MOESM6_ESM.zip › Figure2/2H/2H.pdf]

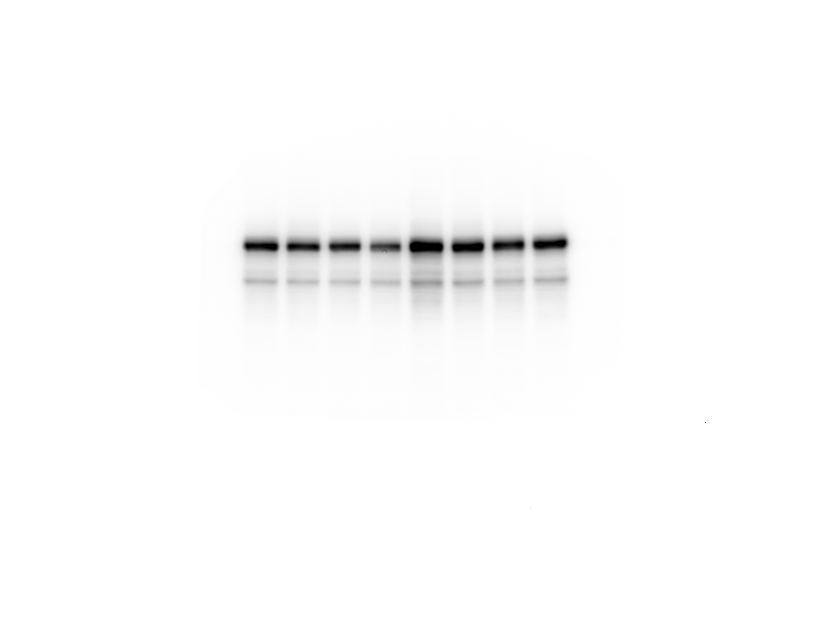

Supplement: Supplementary file 6 — Source data Fig. 2 [file 44318_2024_281_MOESM6_ESM.zip › Figure2/2H/western LSR.png]

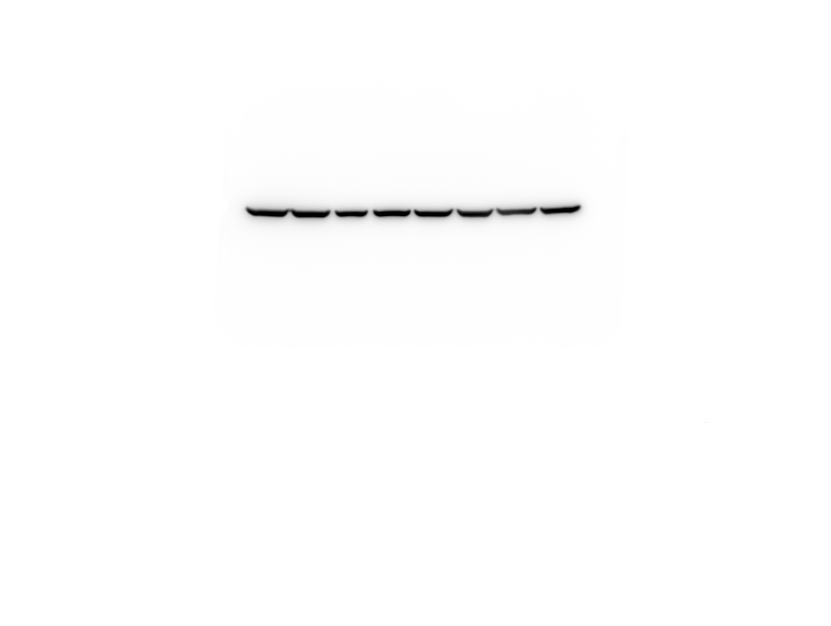

Supplement: Supplementary file 6 — Source data Fig. 2 [file 44318_2024_281_MOESM6_ESM.zip › Figure2/2H/western actin.png]

kDa

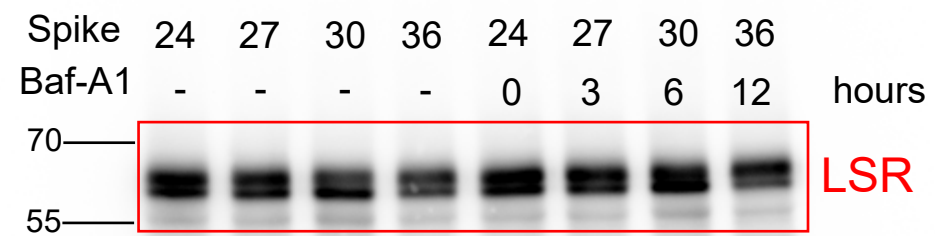

kDa

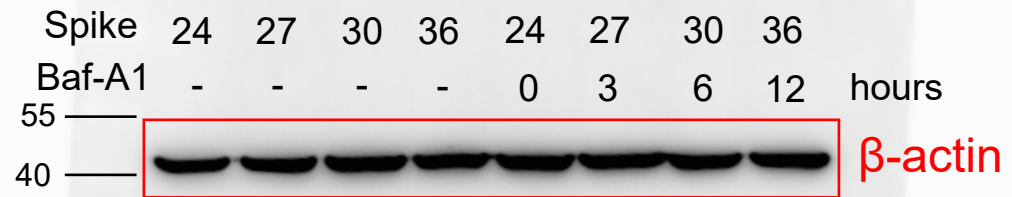

Supplement: Supplementary file 6 — Source data Fig. 2 [file 44318_2024_281_MOESM6_ESM.zip › Figure2/2I/2I.pdf]

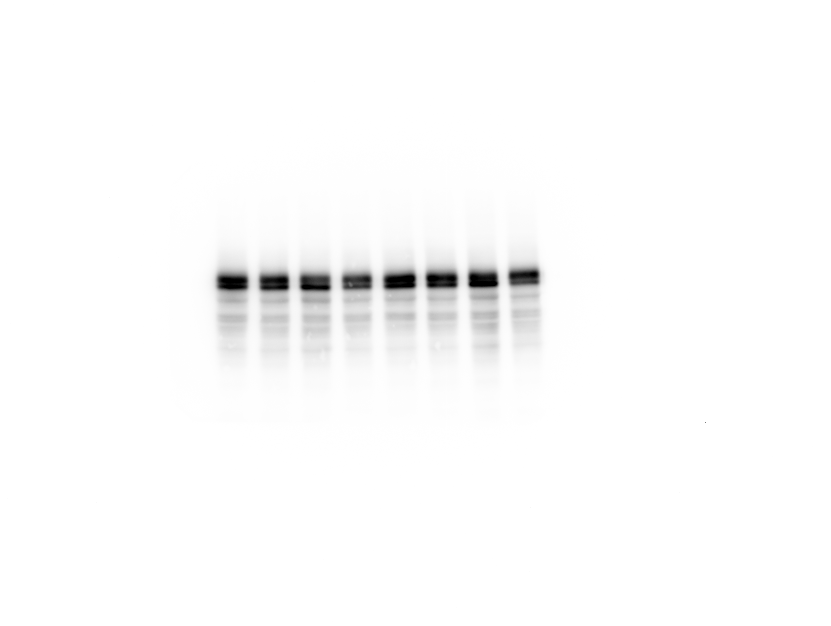

Supplement: Supplementary file 6 — Source data Fig. 2 [file 44318_2024_281_MOESM6_ESM.zip › Figure2/2I/western LSR.png]

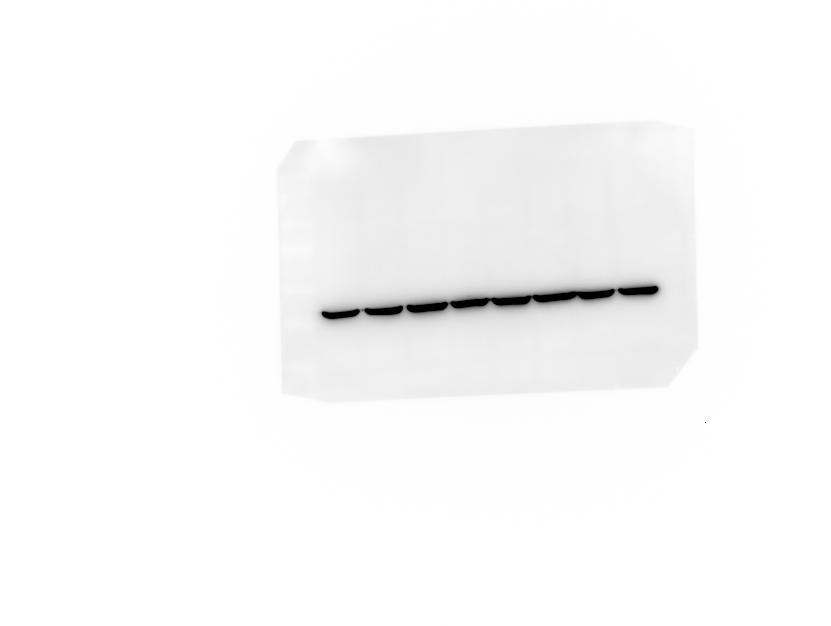

Supplement: Supplementary file 6 — Source data Fig. 2 [file 44318_2024_281_MOESM6_ESM.zip › Figure2/2I/western actin.png]

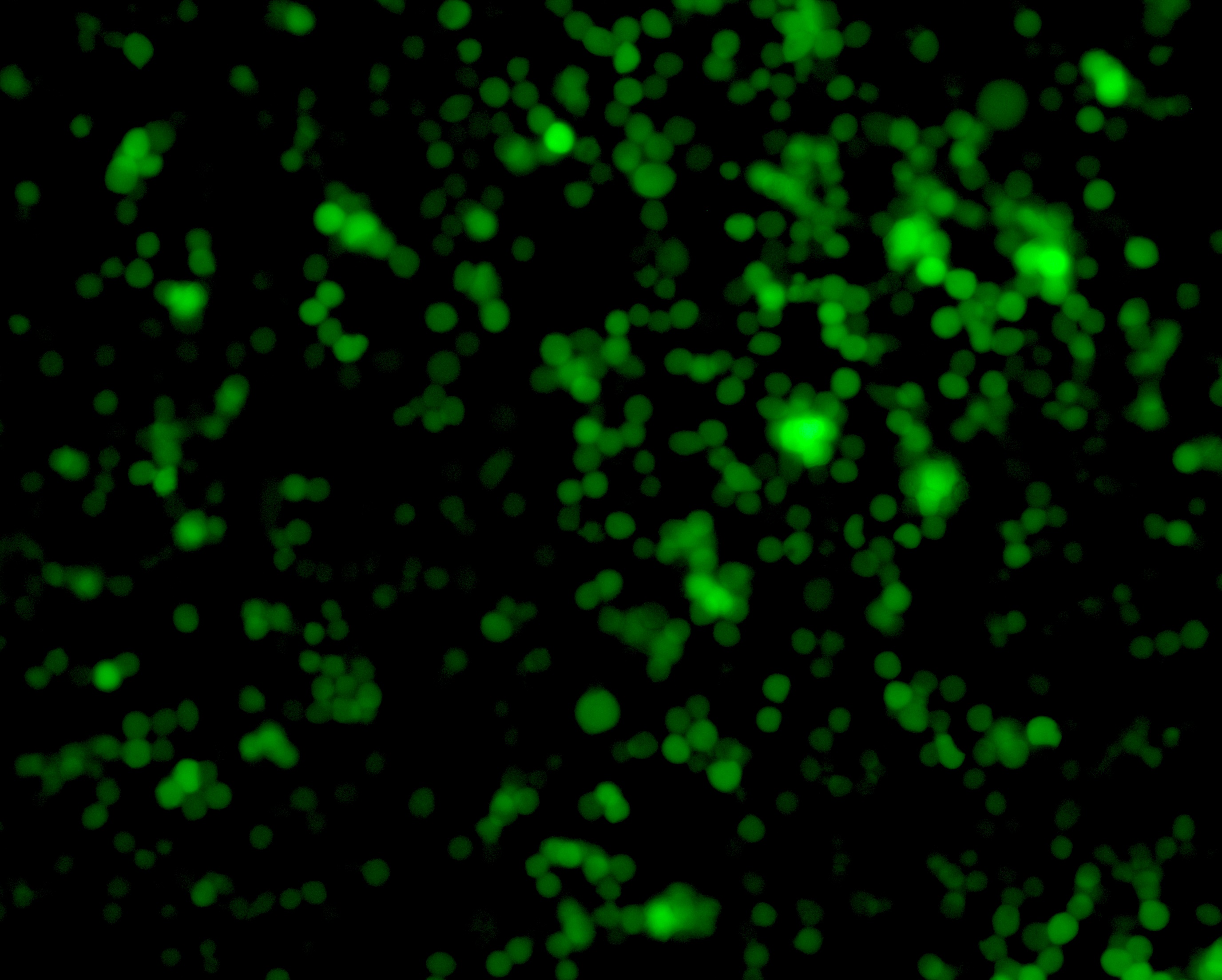

Supplement: Supplementary file 7 — Source data Fig. 3 [file 44318_2024_281_MOESM7_ESM.zip › Figure3/3B/GFP 0.1μg LSR.jpg]

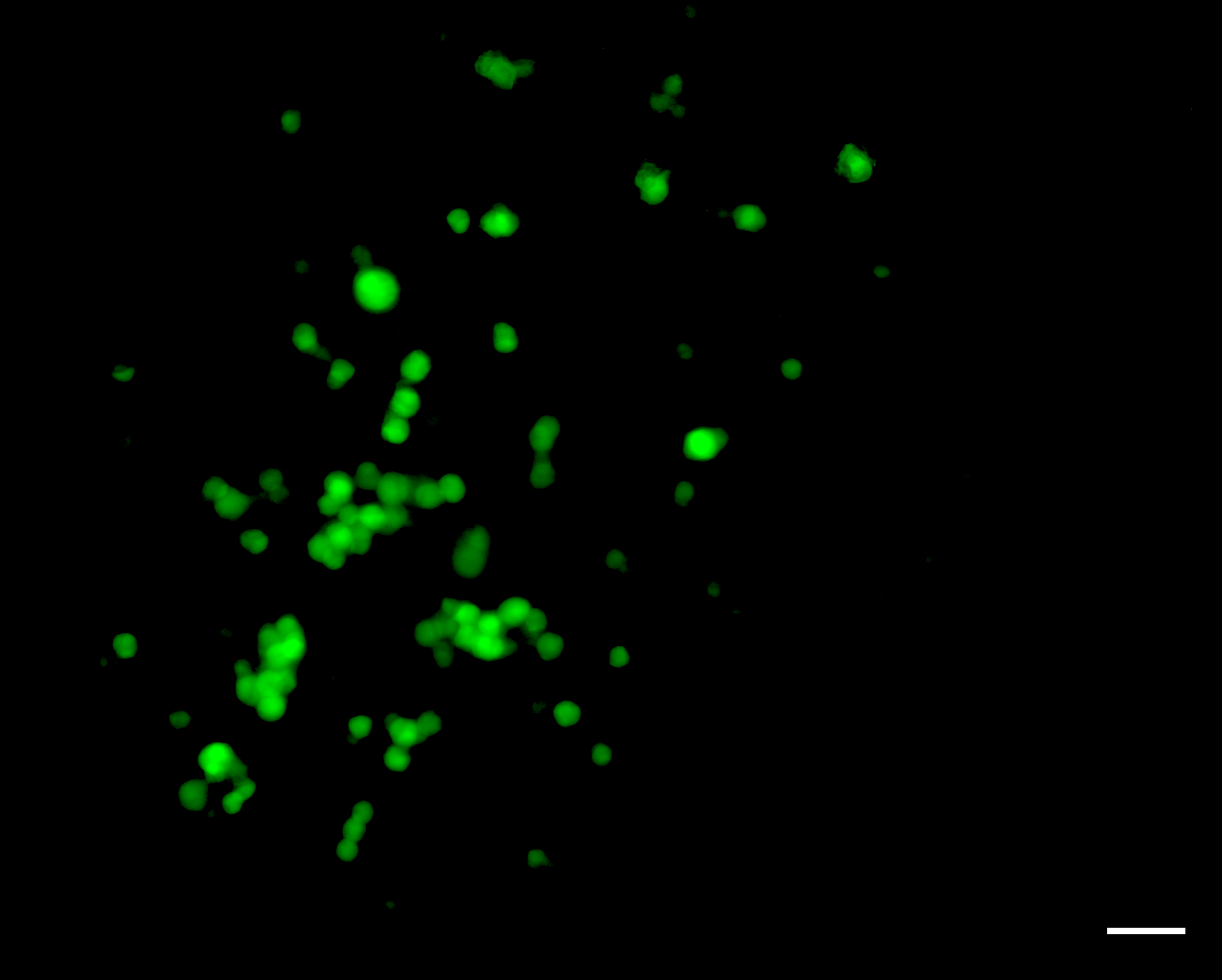

Supplement: Supplementary file 7 — Source data Fig. 3 [file 44318_2024_281_MOESM7_ESM.zip › Figure3/3B/GFP 0.5μg LSR.tif]

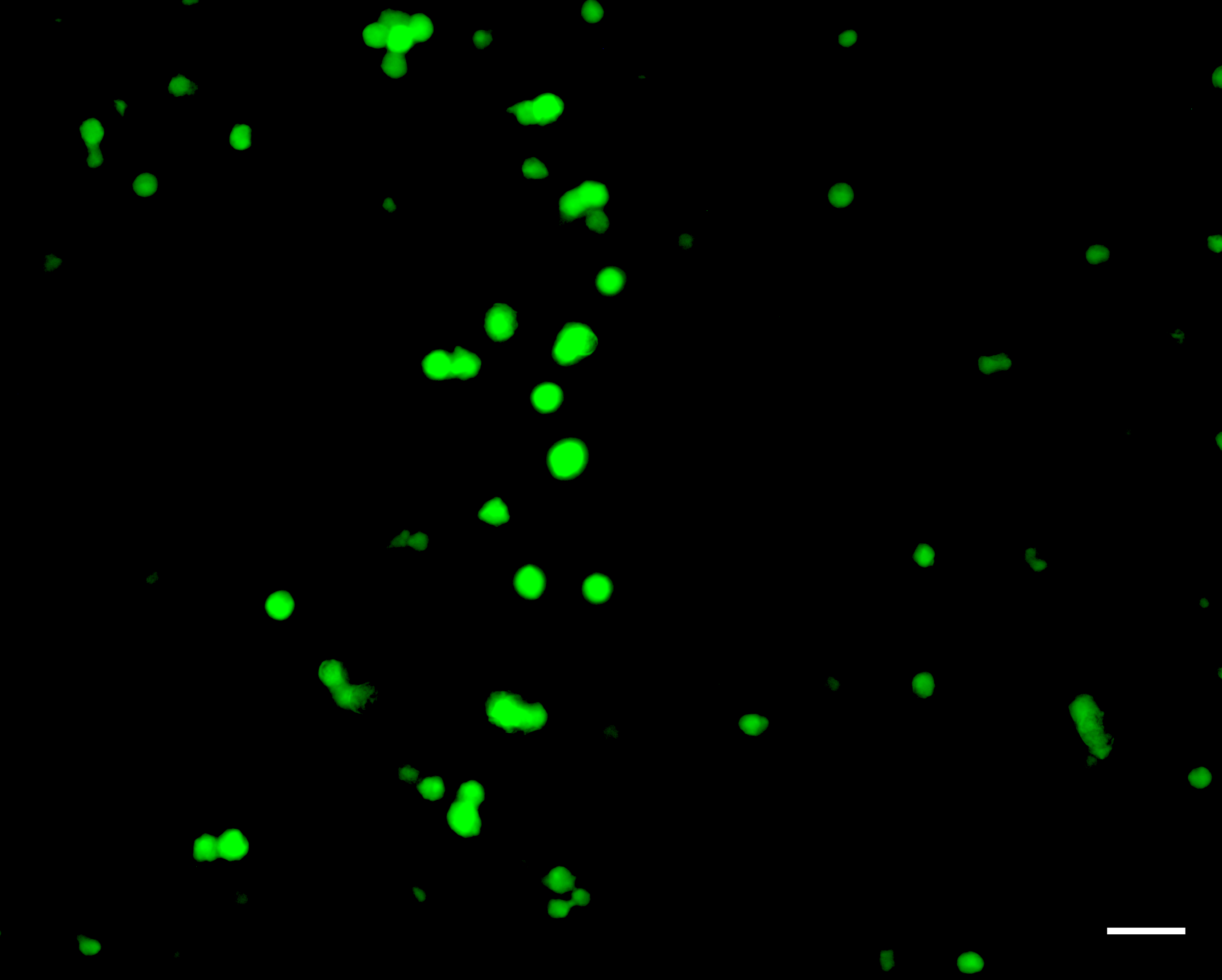

Supplement: Supplementary file 7 — Source data Fig. 3 [file 44318_2024_281_MOESM7_ESM.zip › Figure3/3B/GFP 1 μg LSR.tif]

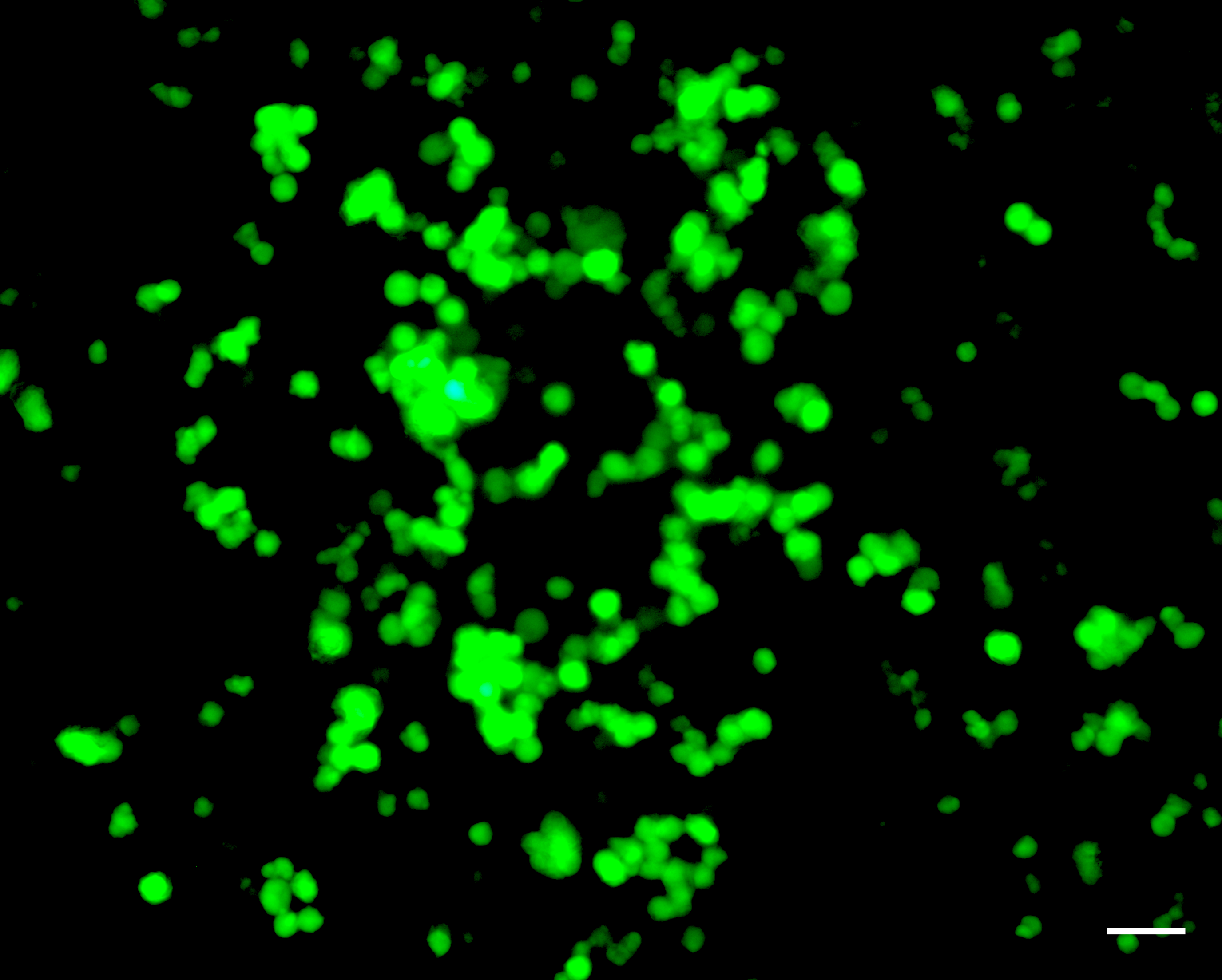

Supplement: Supplementary file 7 — Source data Fig. 3 [file 44318_2024_281_MOESM7_ESM.zip › Figure3/3B/GFP LSR KD.tif]

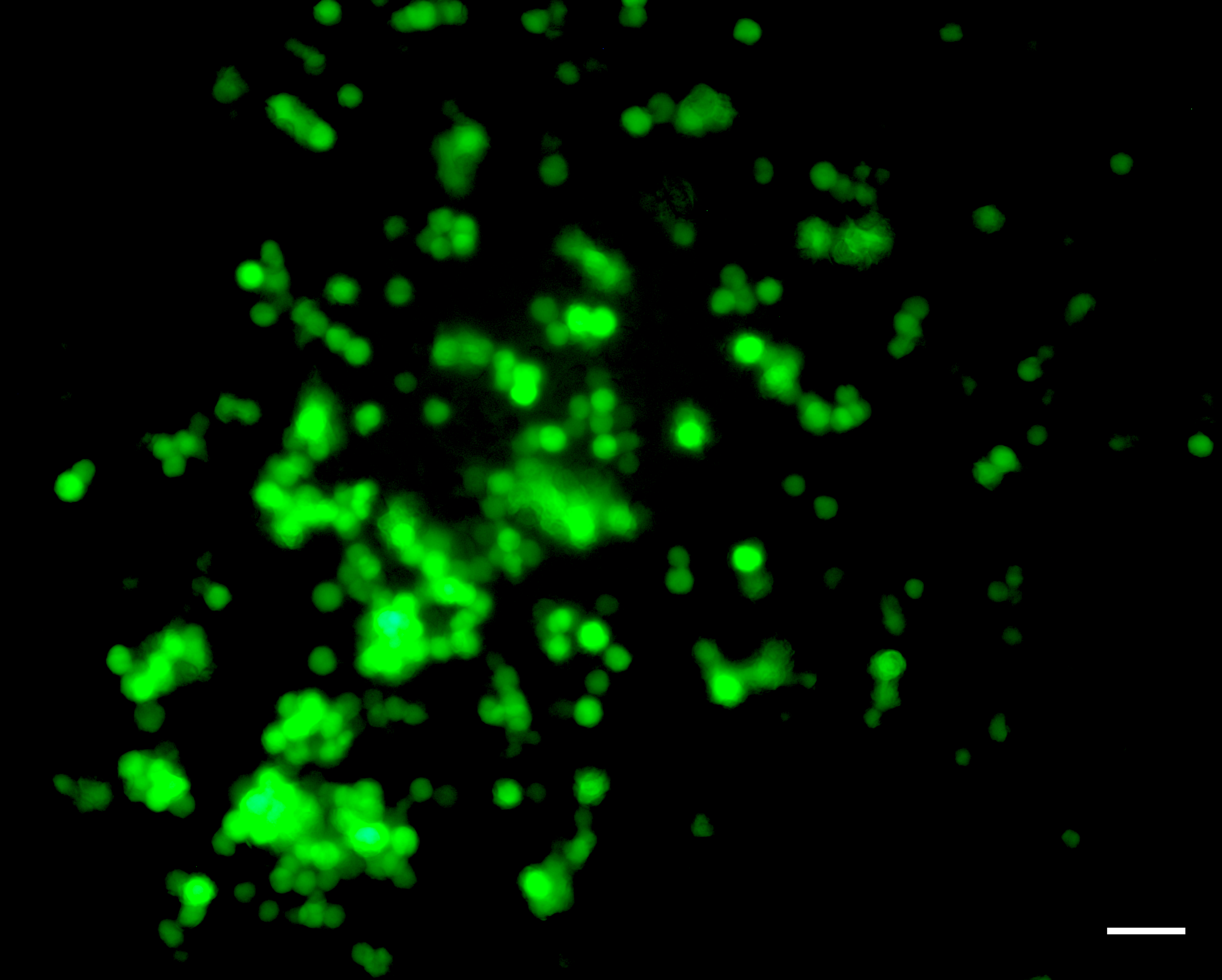

Supplement: Supplementary file 7 — Source data Fig. 3 [file 44318_2024_281_MOESM7_ESM.zip › Figure3/3B/GFP control.tif]

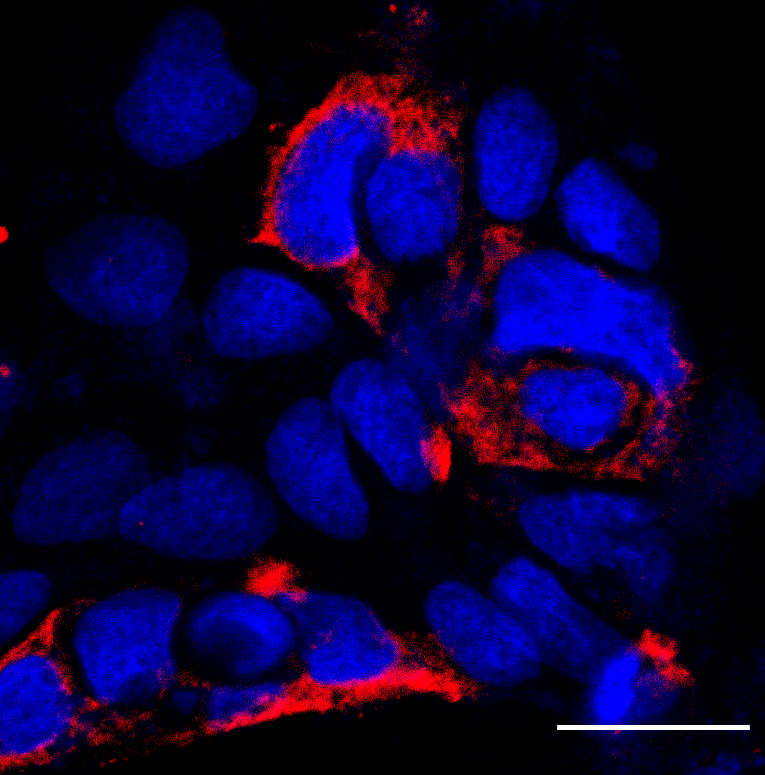

Supplement: Supplementary file 7 — Source data Fig. 3 [file 44318_2024_281_MOESM7_ESM.zip › Figure3/3E/IF N protein Omicron LSR-KD.tif]

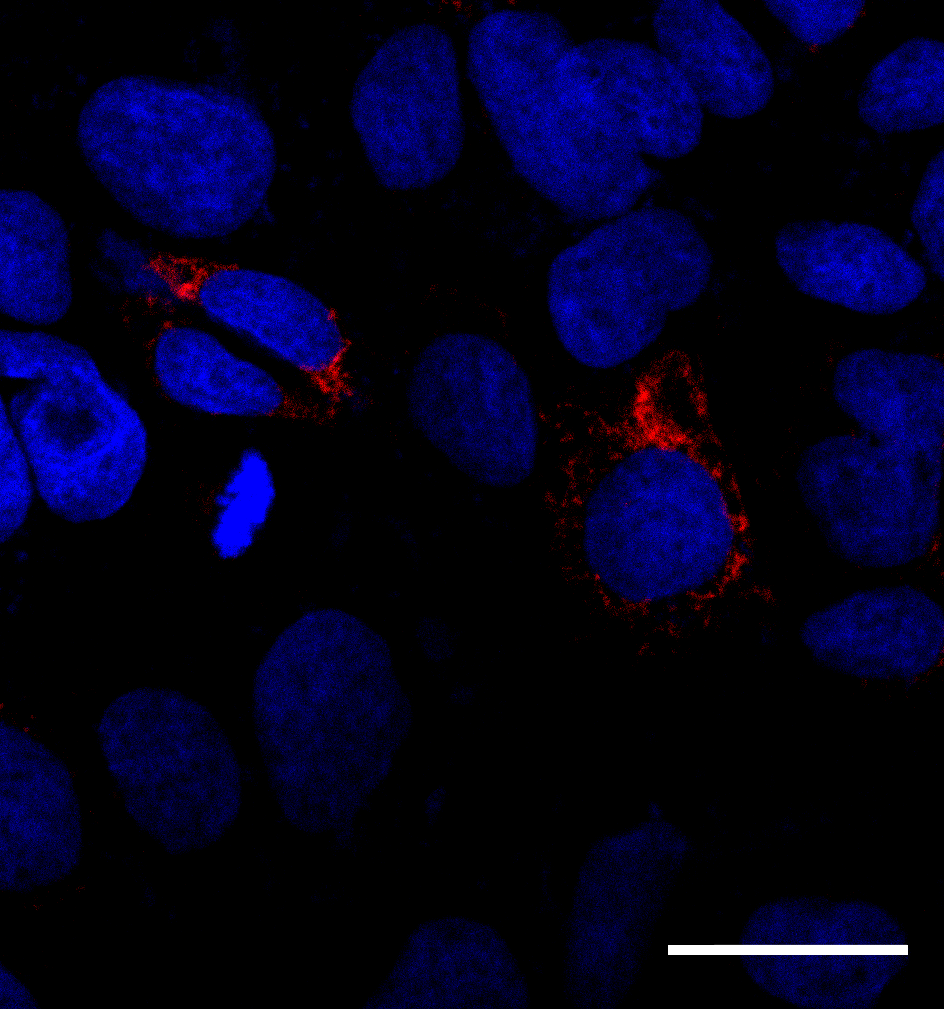

Supplement: Supplementary file 7 — Source data Fig. 3 [file 44318_2024_281_MOESM7_ESM.zip › Figure3/3E/IF N protein Omicron LSR-OE.tif]

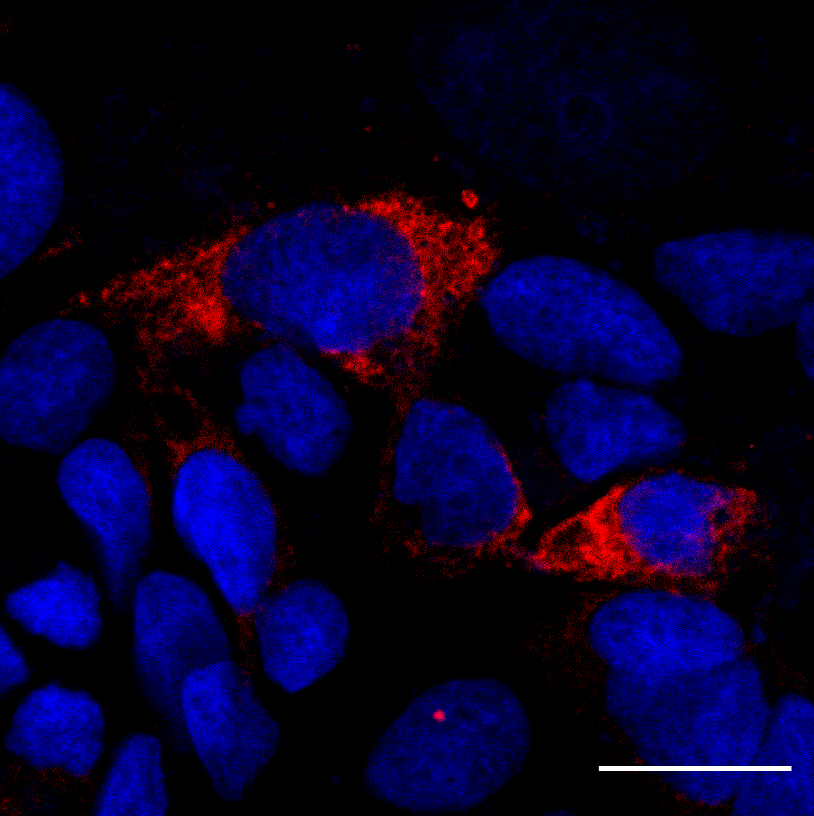

Supplement: Supplementary file 7 — Source data Fig. 3 [file 44318_2024_281_MOESM7_ESM.zip › Figure3/3E/IF N protein Omicron Wildtype.tif]

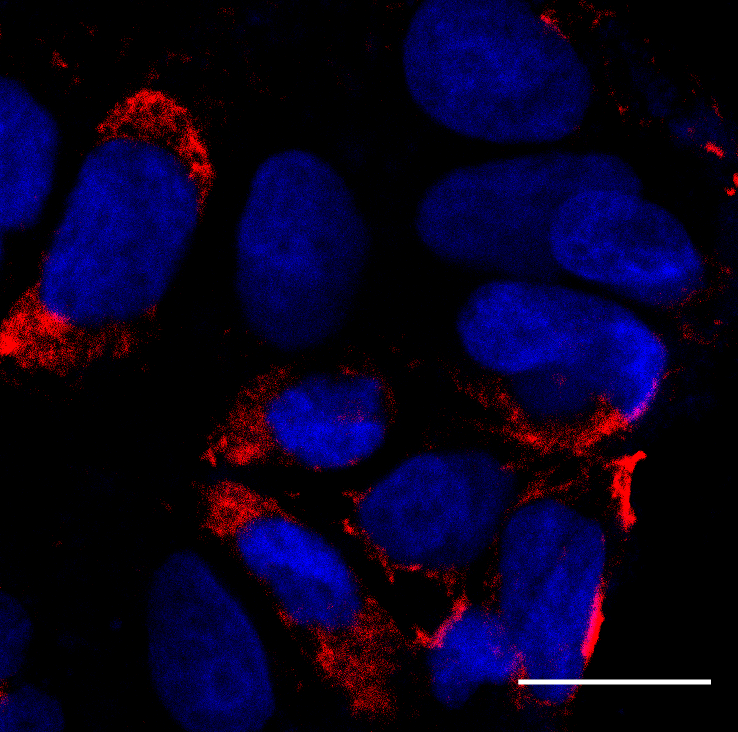

Supplement: Supplementary file 7 — Source data Fig. 3 [file 44318_2024_281_MOESM7_ESM.zip › Figure3/3E/IF N protein SARS-CoV-2 WT LSR-KD.tif]

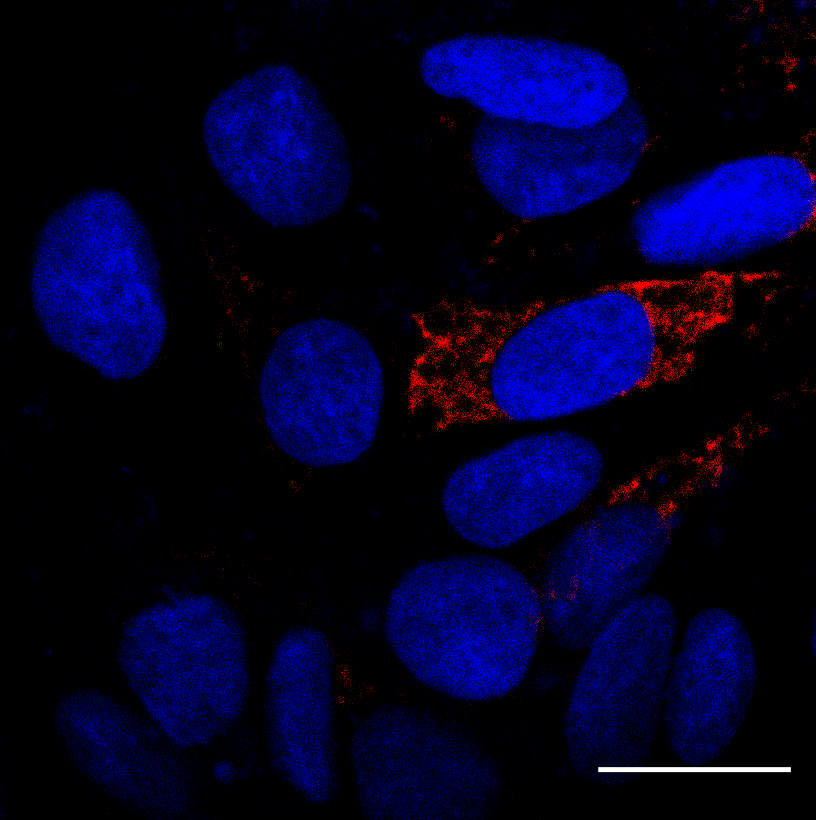

Supplement: Supplementary file 7 — Source data Fig. 3 [file 44318_2024_281_MOESM7_ESM.zip › Figure3/3E/IF N protein SARS-CoV-2 WT LSR-OE.tif]

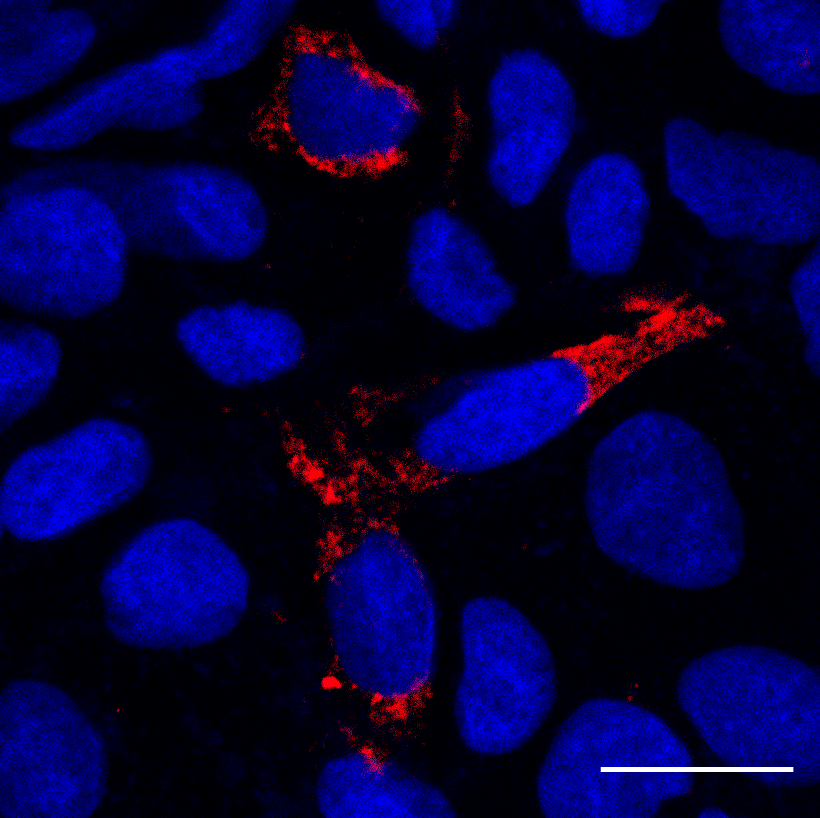

Supplement: Supplementary file 7 — Source data Fig. 3 [file 44318_2024_281_MOESM7_ESM.zip › Figure3/3E/IF N protein SARS-CoV-2 WT Wildtype.tif]

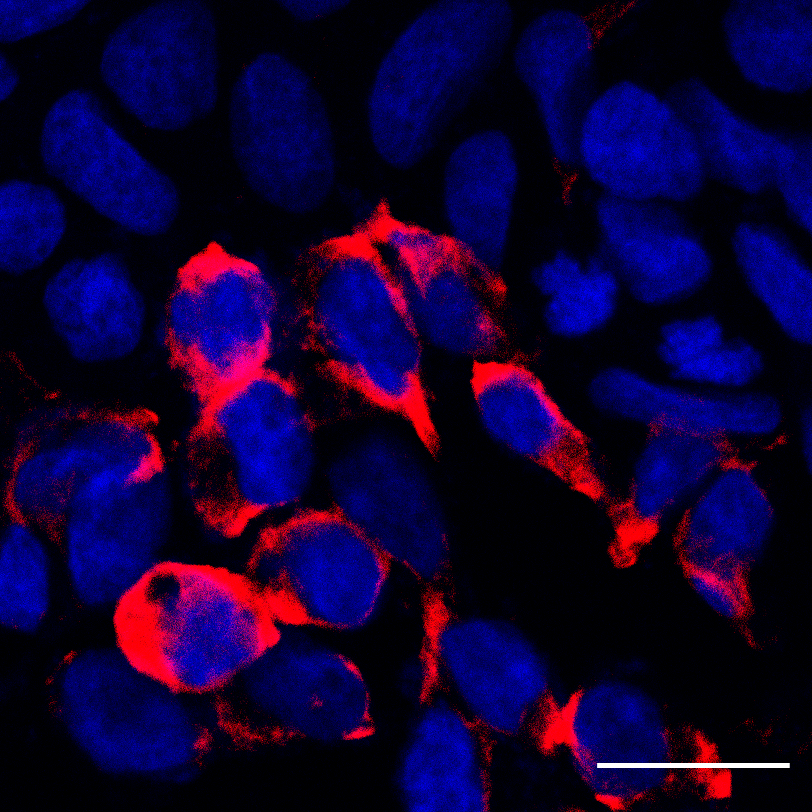

Supplement: Supplementary file 7 — Source data Fig. 3 [file 44318_2024_281_MOESM7_ESM.zip › Figure3/3E/IF N protein XBB LSR-KD.tif]

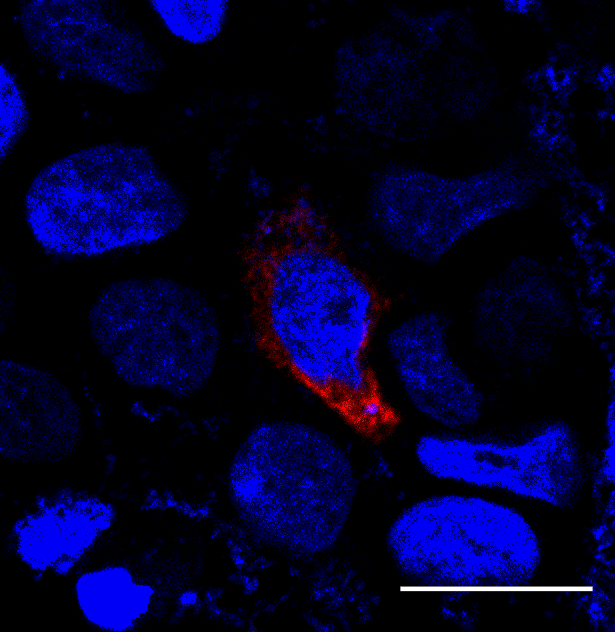

Supplement: Supplementary file 7 — Source data Fig. 3 [file 44318_2024_281_MOESM7_ESM.zip › Figure3/3E/IF N protein XBB LSR-OE.tif]

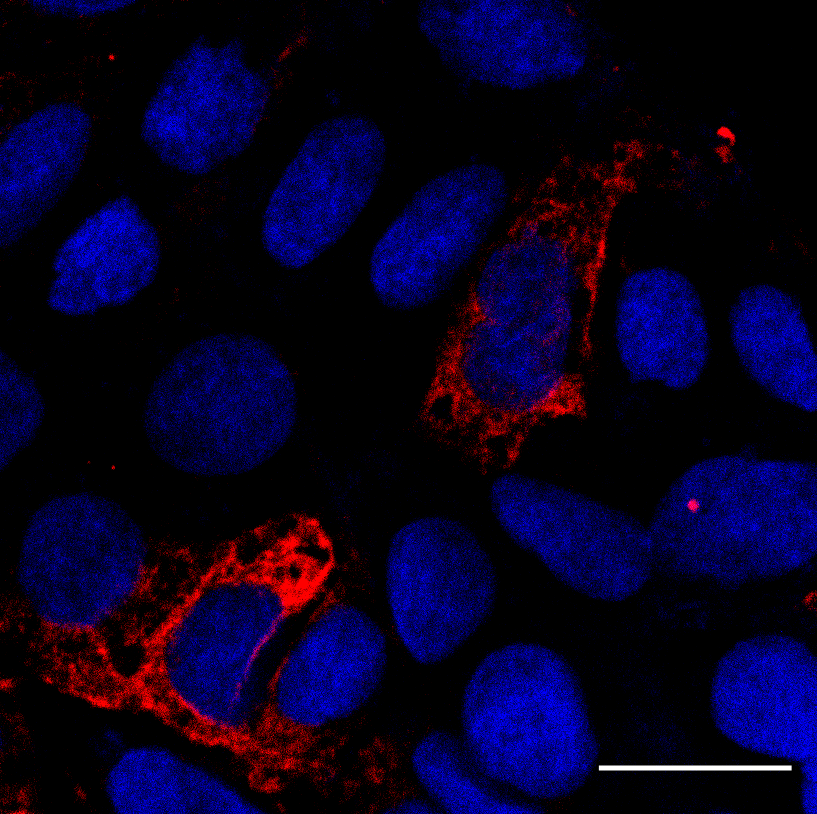

Supplement: Supplementary file 7 — Source data Fig. 3 [file 44318_2024_281_MOESM7_ESM.zip › Figure3/3E/IF N protein XBB Wildtype.tif]

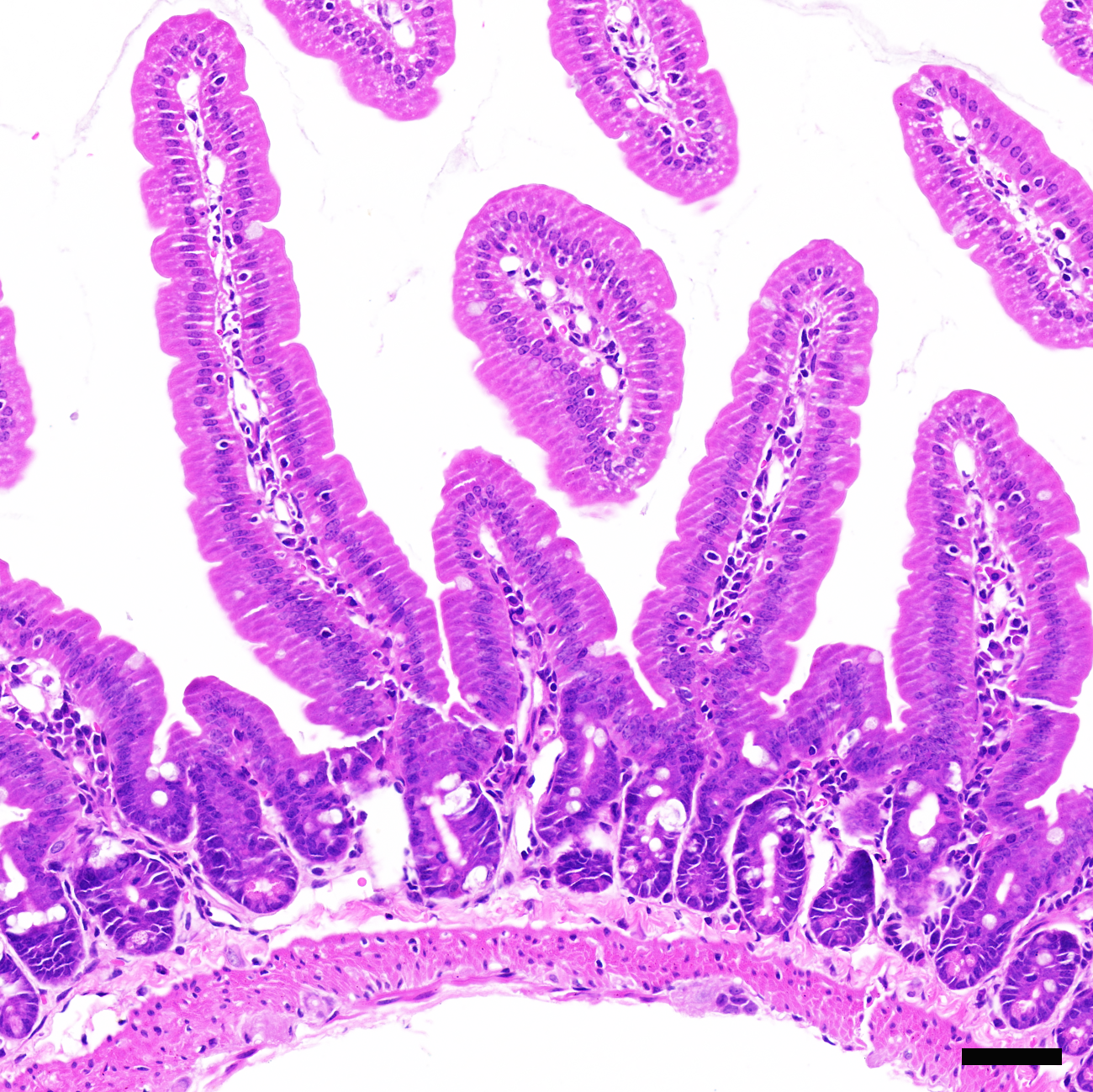

Supplement: Supplementary file 7 — Source data Fig. 3 [file 44318_2024_281_MOESM7_ESM.zip › Figure3/3G/HE LSRvillKO control.tif]

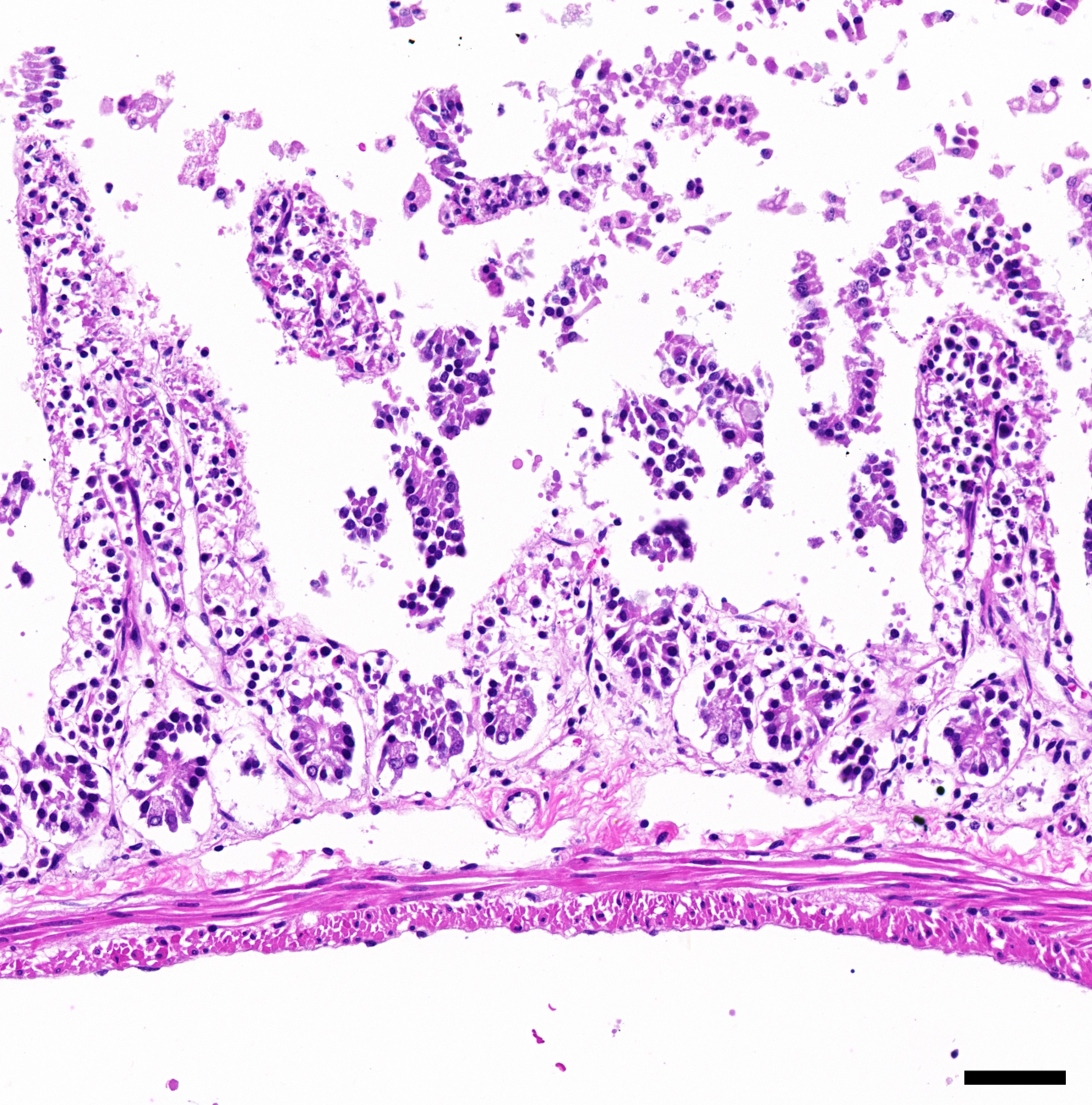

Supplement: Supplementary file 7 — Source data Fig. 3 [file 44318_2024_281_MOESM7_ESM.zip › Figure3/3G/HE LSRvillKO infected.tif]

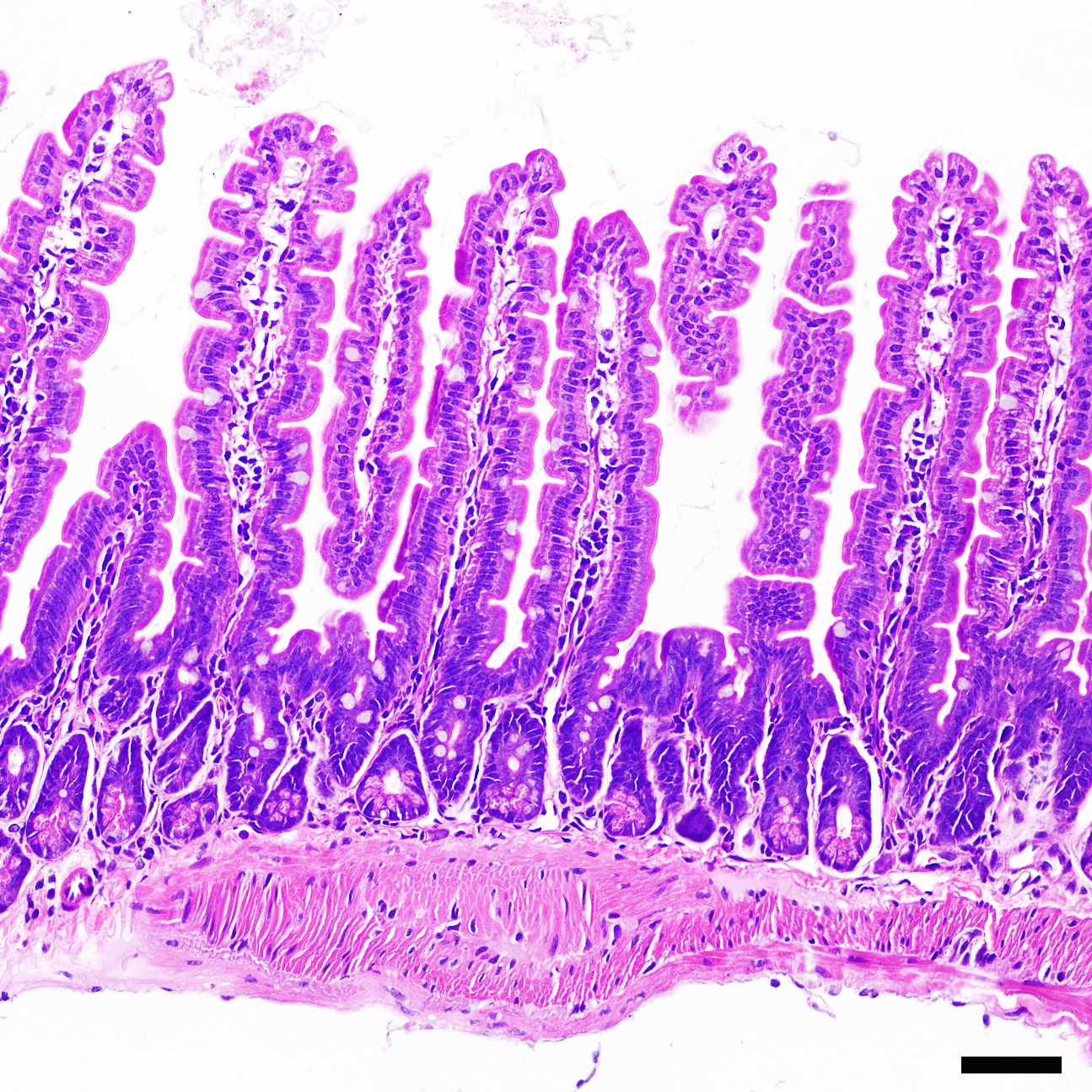

Supplement: Supplementary file 7 — Source data Fig. 3 [file 44318_2024_281_MOESM7_ESM.zip › Figure3/3G/HE WT control.tif]

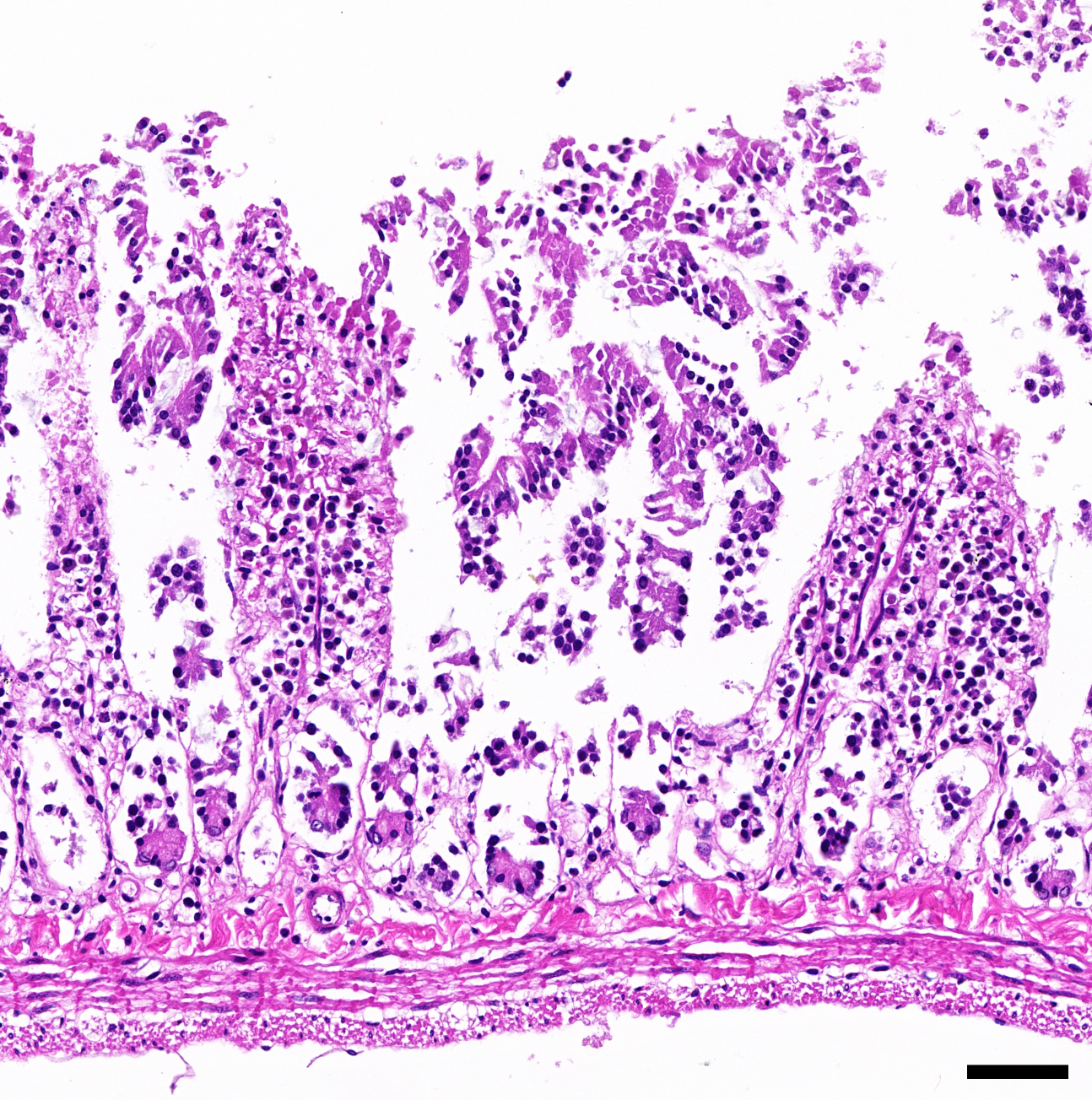

Supplement: Supplementary file 7 — Source data Fig. 3 [file 44318_2024_281_MOESM7_ESM.zip › Figure3/3G/HE WT infected.tif]

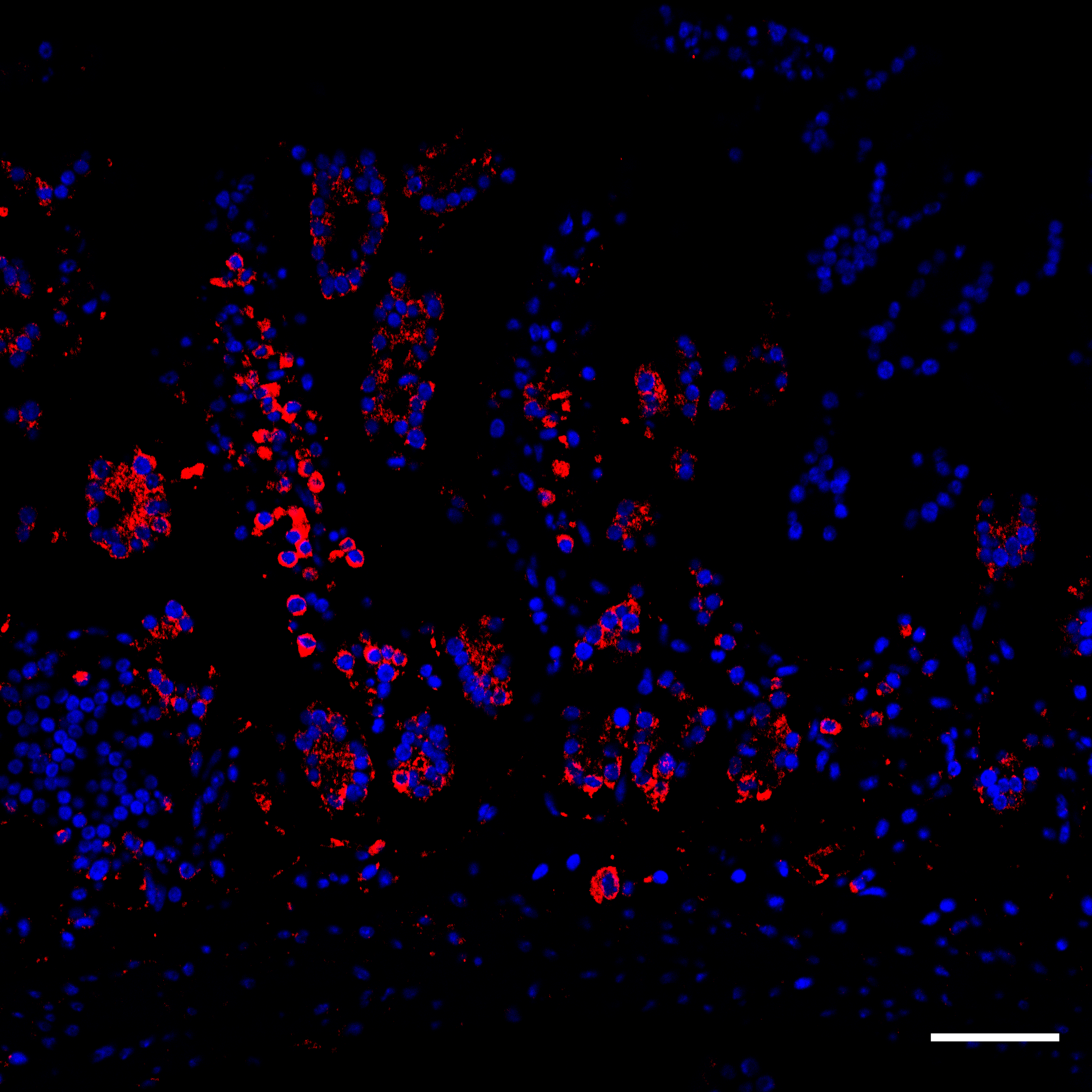

Supplement: Supplementary file 7 — Source data Fig. 3 [file 44318_2024_281_MOESM7_ESM.zip › Figure3/3J/IF N protein LSRvillKO SARS-CoV-2 WT.tif]

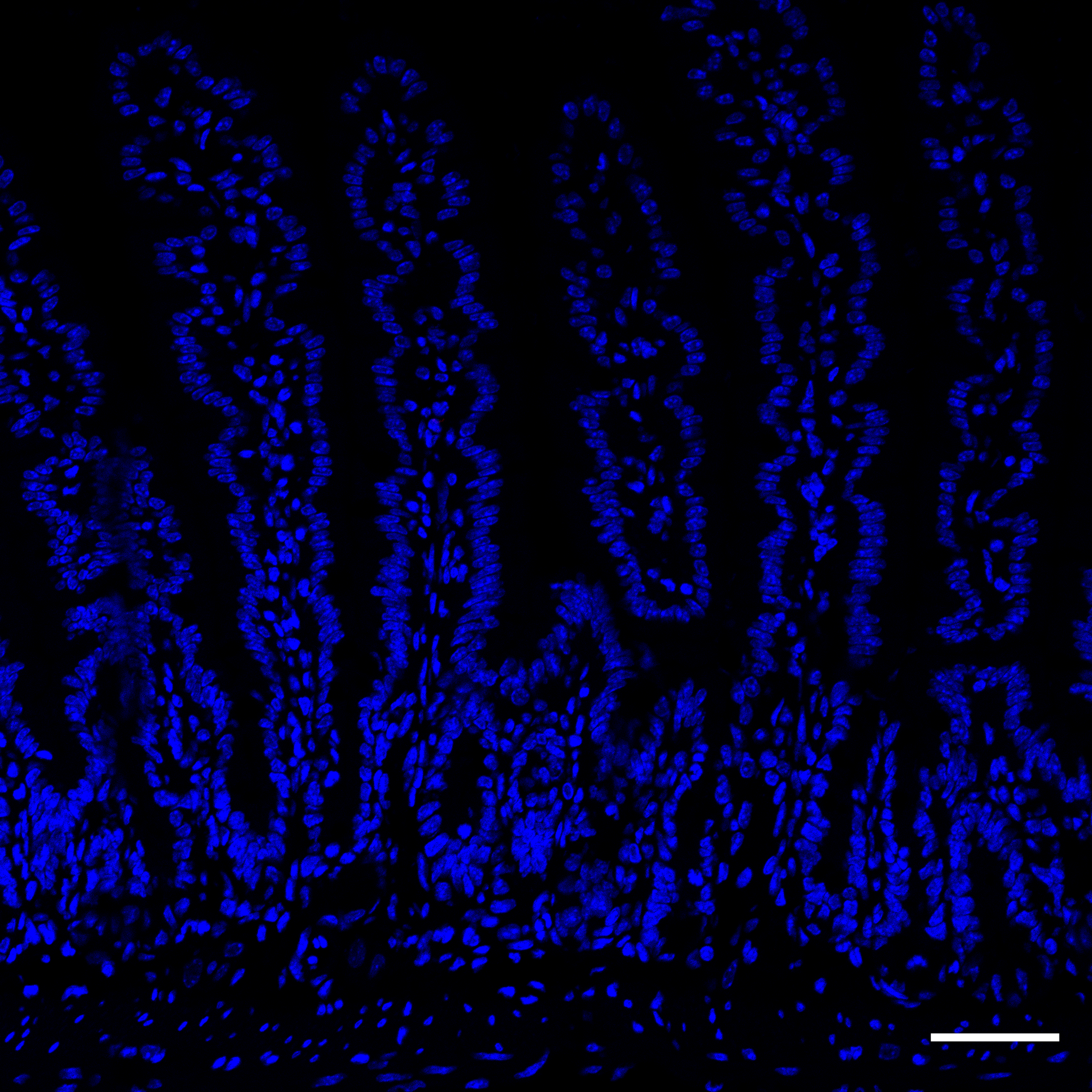

Supplement: Supplementary file 7 — Source data Fig. 3 [file 44318_2024_281_MOESM7_ESM.zip › Figure3/3J/IF N protein LSRvillKO control.tif]
