# Supplementary figures and images for: Tight junction protein LSR is a host defense factor against SARS-CoV-2 infection in the small intestine (part 2 of 4)
Source: EMBO J. 2024 Oct 23;43(23):6124–51. doi: 10.1038/s44318-024-00281-4 (PMC11612383; doi:10.1038/s44318-024-00281-4)

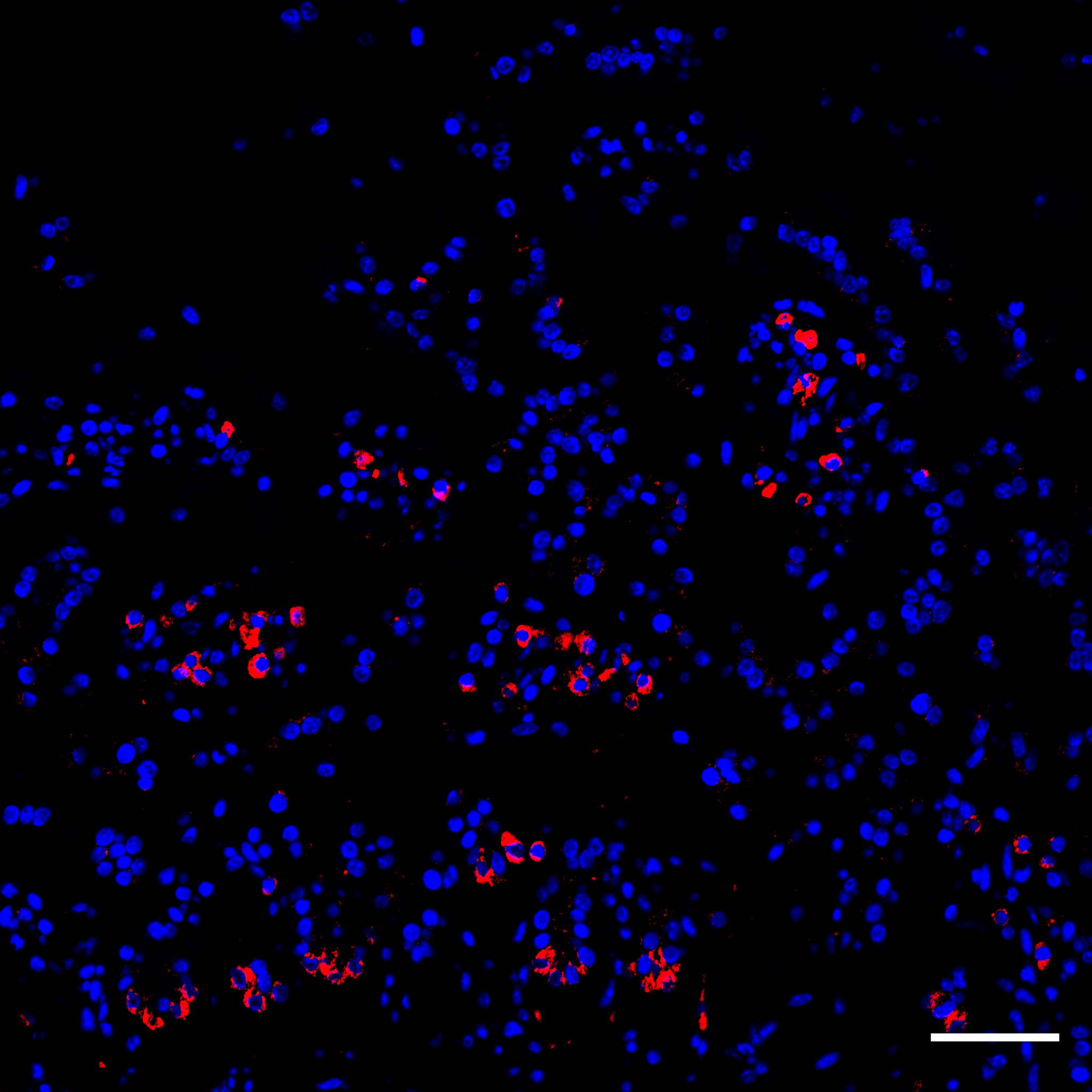

Supplement: Supplementary file 7 — Source data Fig. 3 [file 44318_2024_281_MOESM7_ESM.zip › Figure3/3J/IF N protein WT SARS-CoV-2 WT.tif]

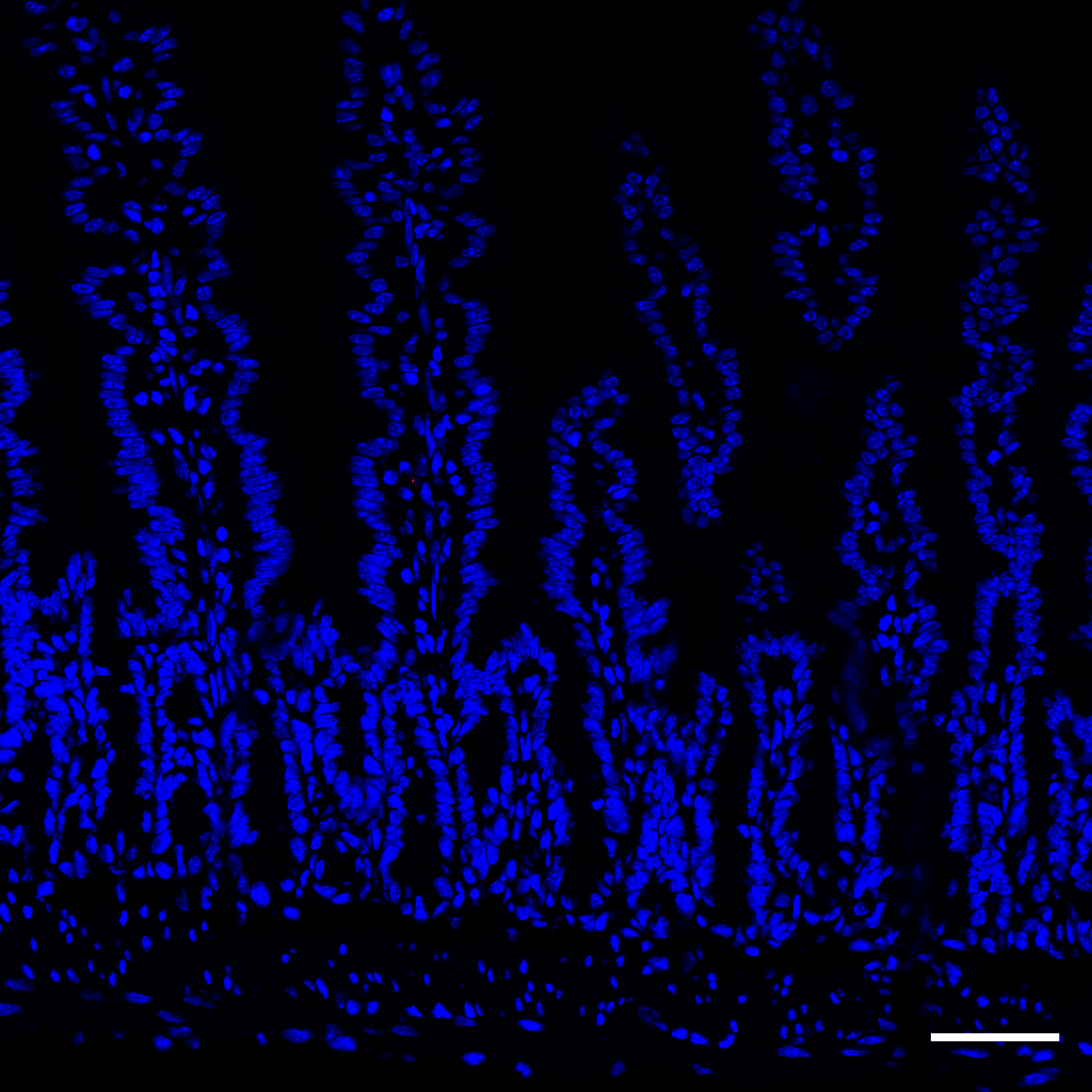

Supplement: Supplementary file 7 — Source data Fig. 3 [file 44318_2024_281_MOESM7_ESM.zip › Figure3/3J/IF N protein WT control.tif]

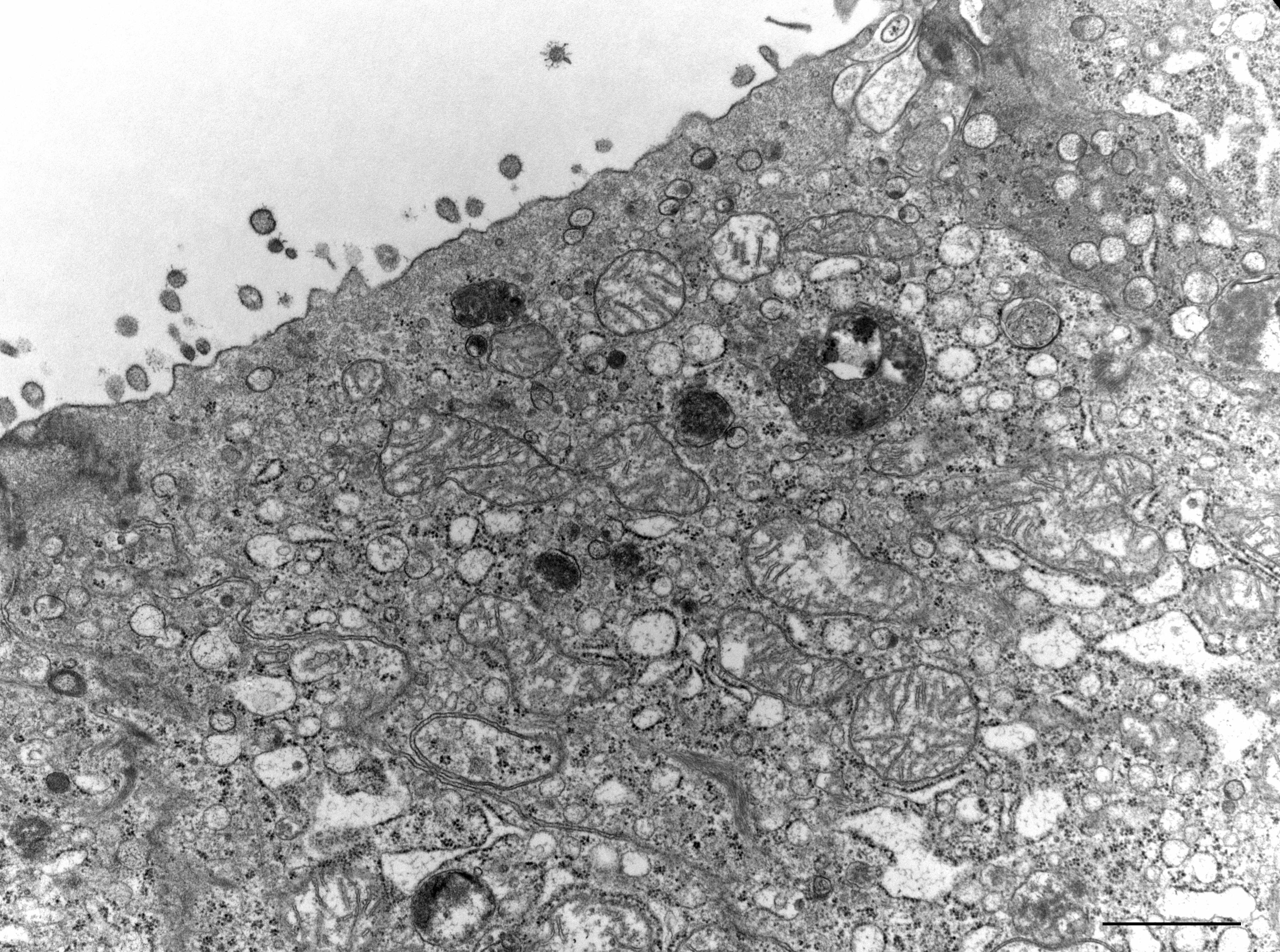

Supplement: Supplementary file 7 — Source data Fig. 3 [file 44318_2024_281_MOESM7_ESM.zip › Figure3/3K/TEM LSRvillKO.tif]

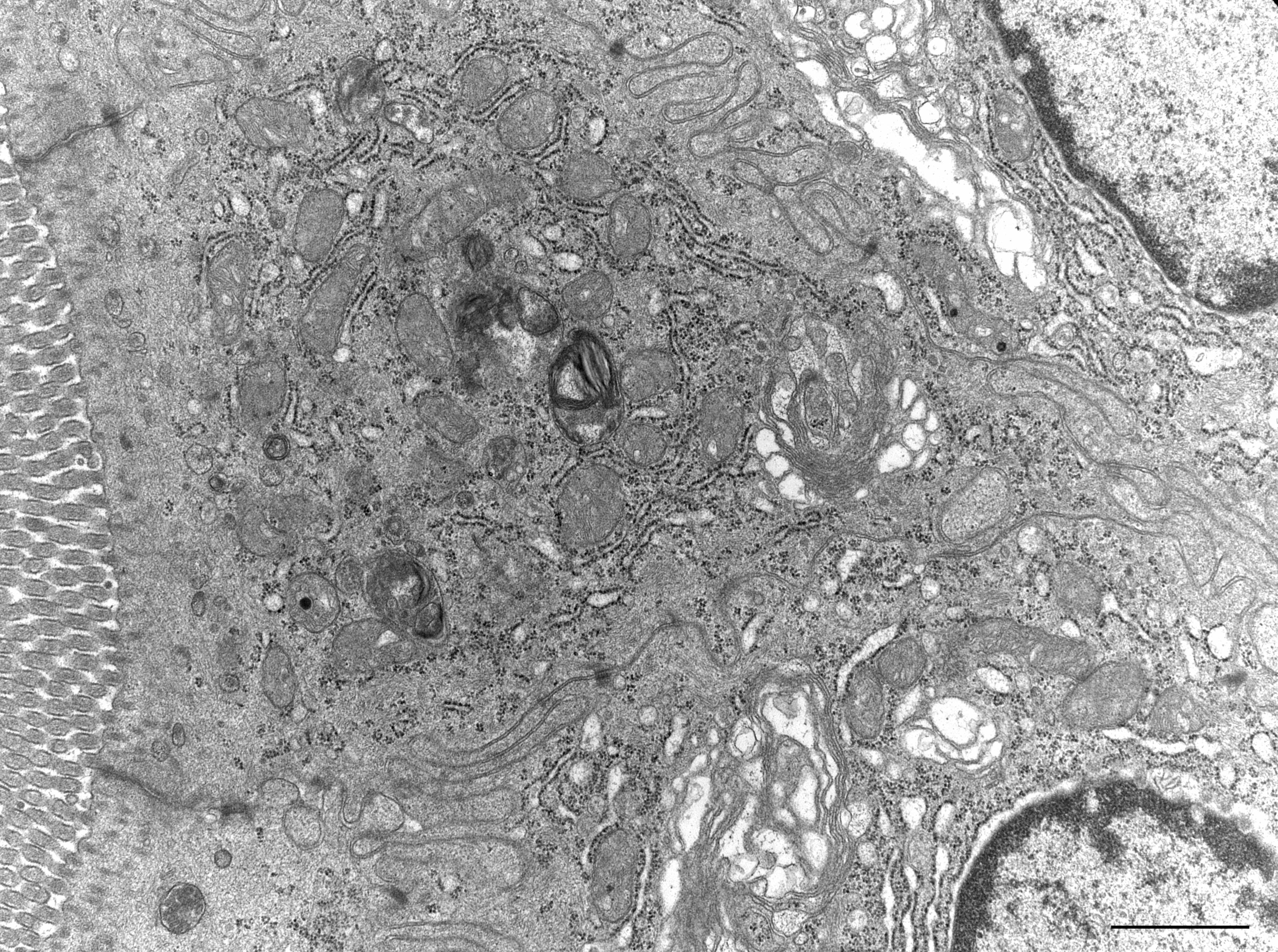

Supplement: Supplementary file 7 — Source data Fig. 3 [file 44318_2024_281_MOESM7_ESM.zip › Figure3/3K/TEM WT.tif]

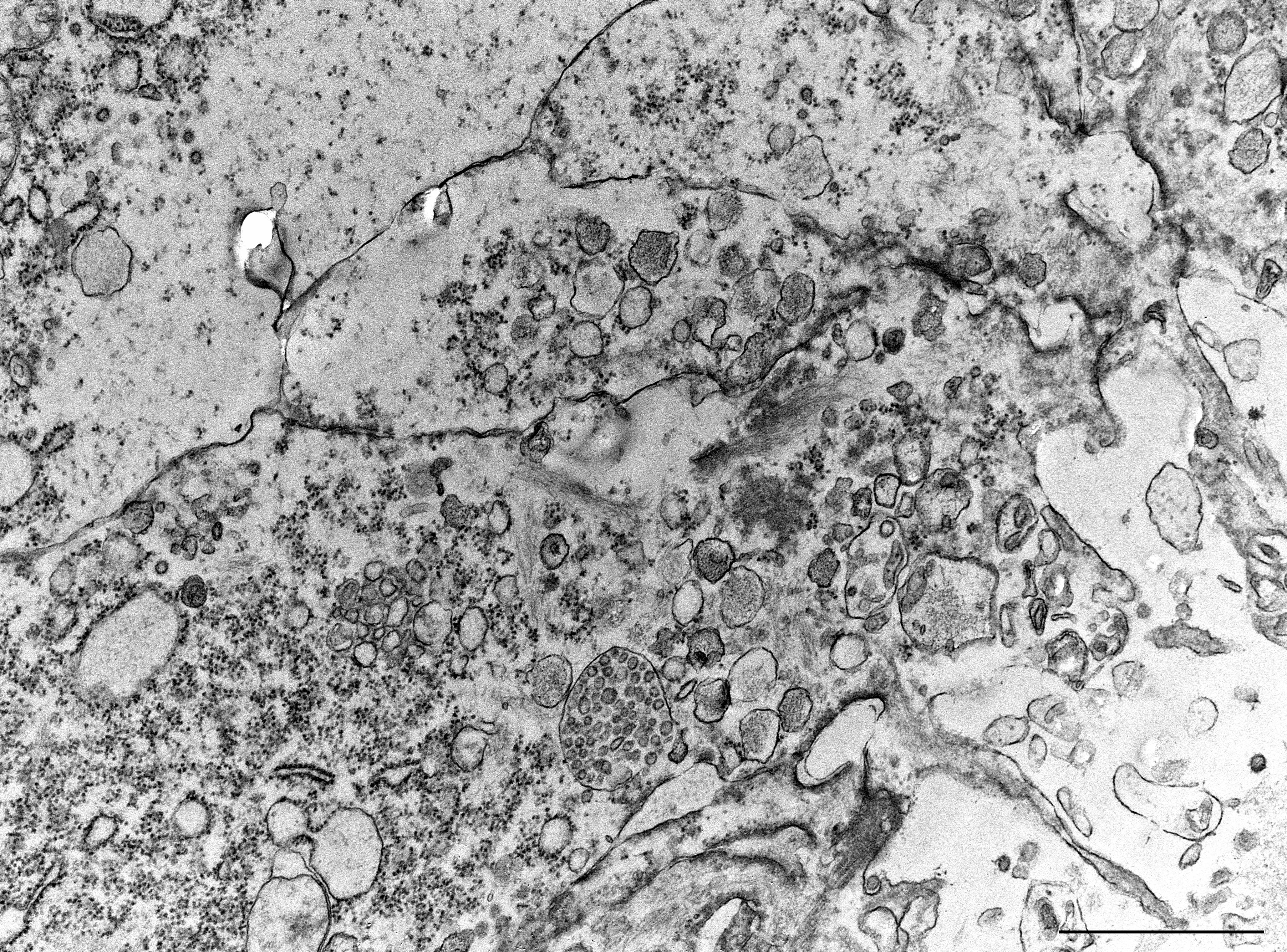

Supplement: Supplementary file 7 — Source data Fig. 3 [file 44318_2024_281_MOESM7_ESM.zip › Figure3/3K/TEM expand LSRvillKO.tif]

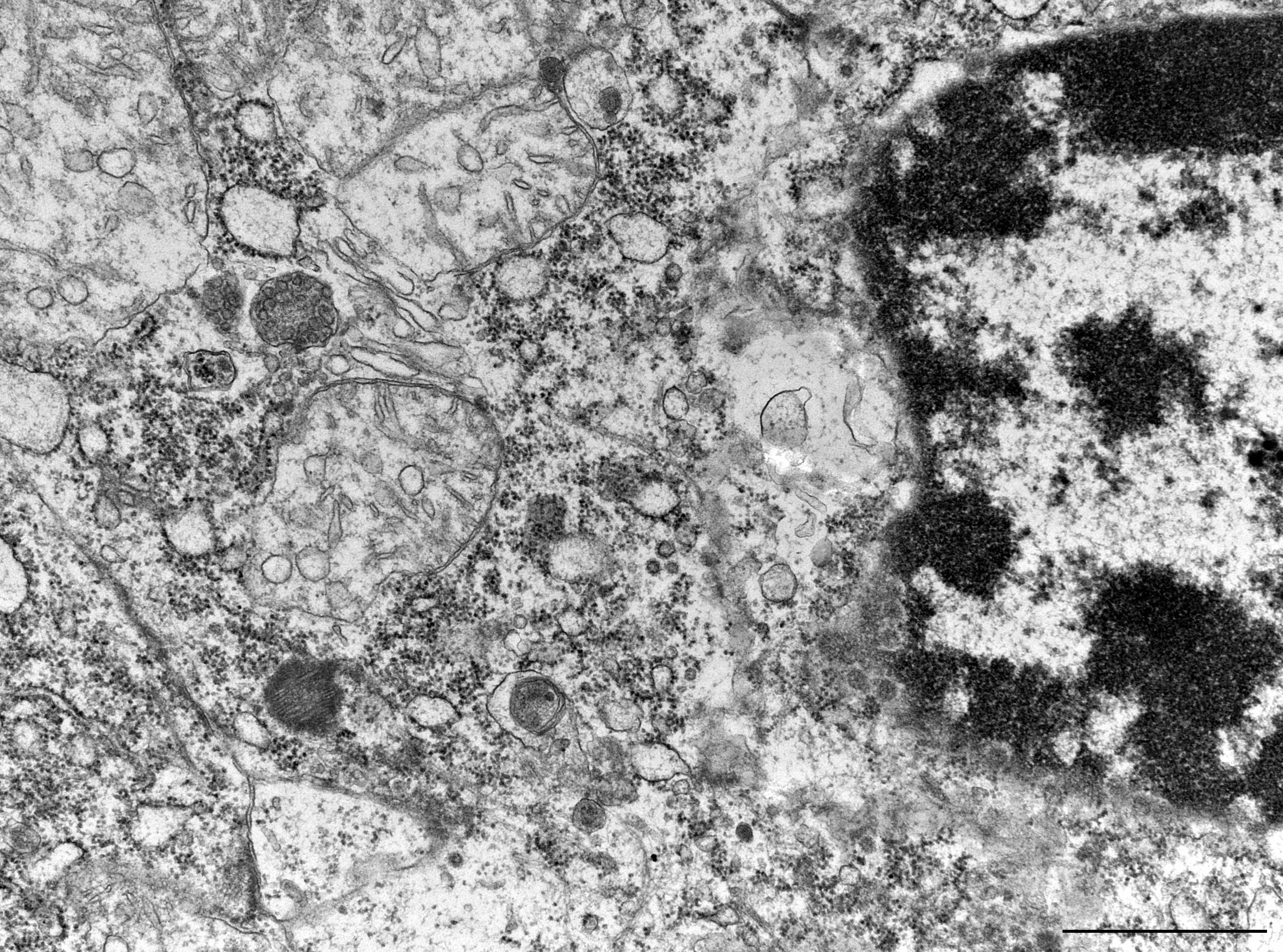

Supplement: Supplementary file 7 — Source data Fig. 3 [file 44318_2024_281_MOESM7_ESM.zip › Figure3/3K/TEM expand WT.tif]

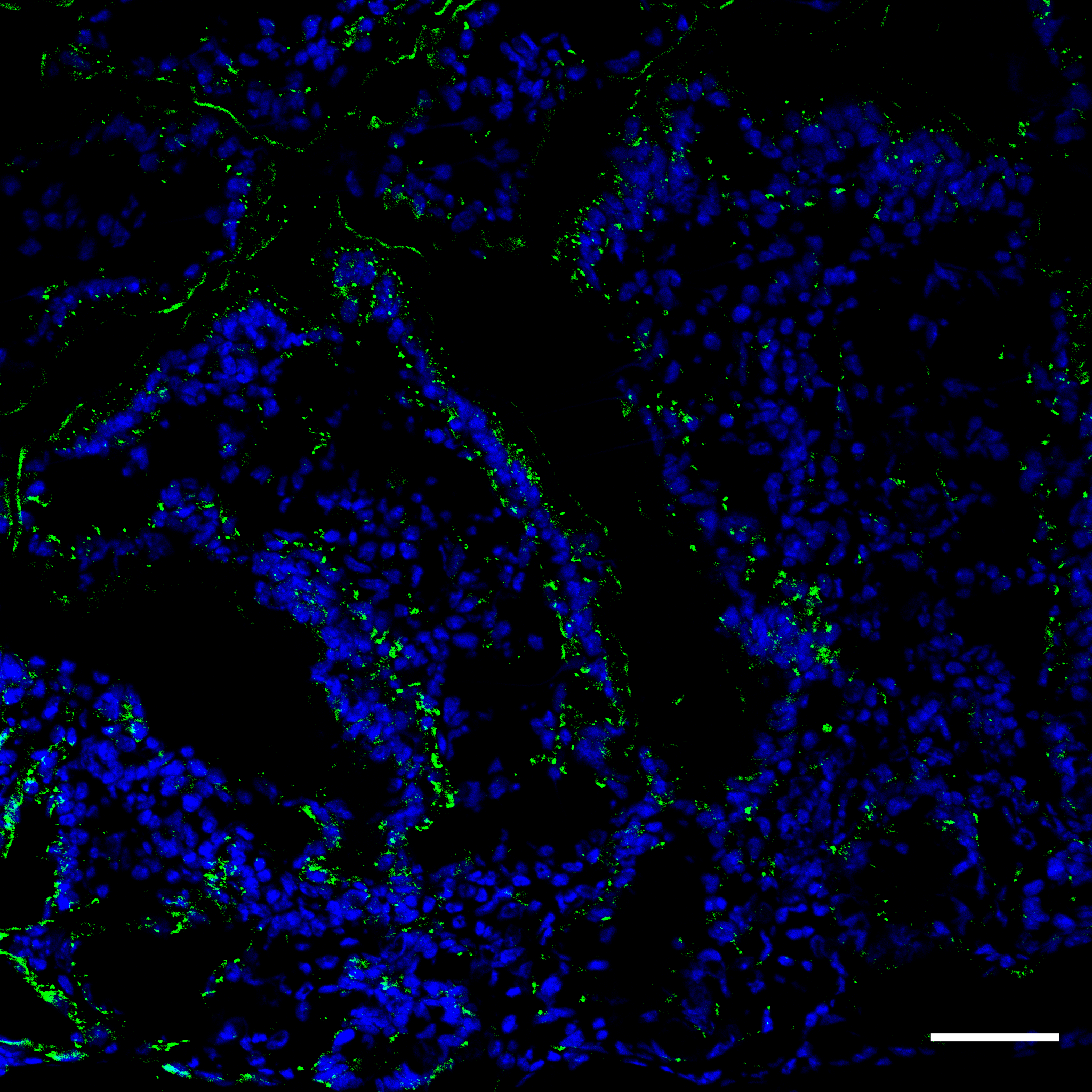

Supplement: Supplementary file 7 — Source data Fig. 3 [file 44318_2024_281_MOESM7_ESM.zip › Figure3/3L/GFP LSR-OE VSV-SARS-CoV-2.tif]

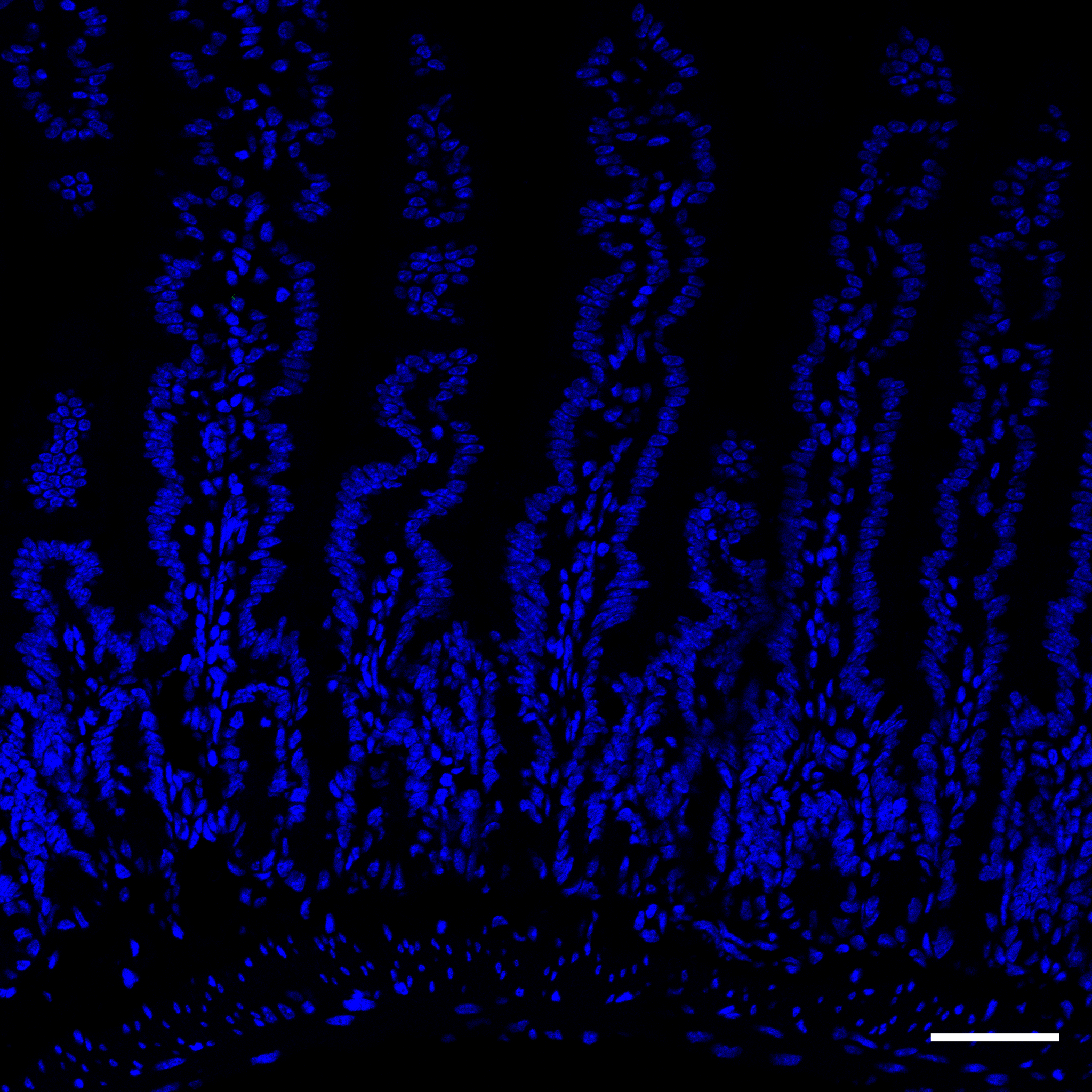

Supplement: Supplementary file 7 — Source data Fig. 3 [file 44318_2024_281_MOESM7_ESM.zip › Figure3/3L/GFP LSR-OE control.tif]

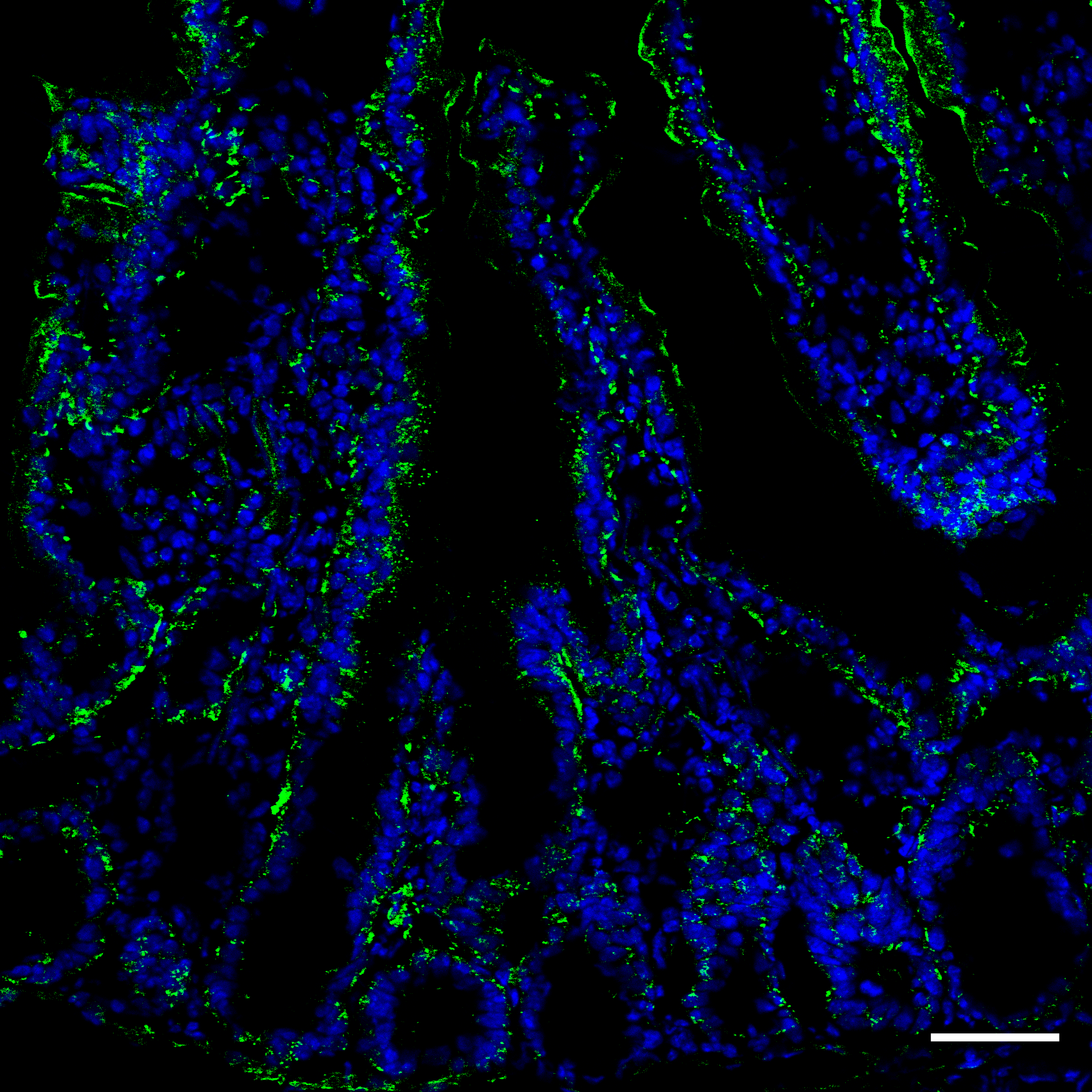

Supplement: Supplementary file 7 — Source data Fig. 3 [file 44318_2024_281_MOESM7_ESM.zip › Figure3/3L/GFP WT VSV-SARS-CoV-2.tif]

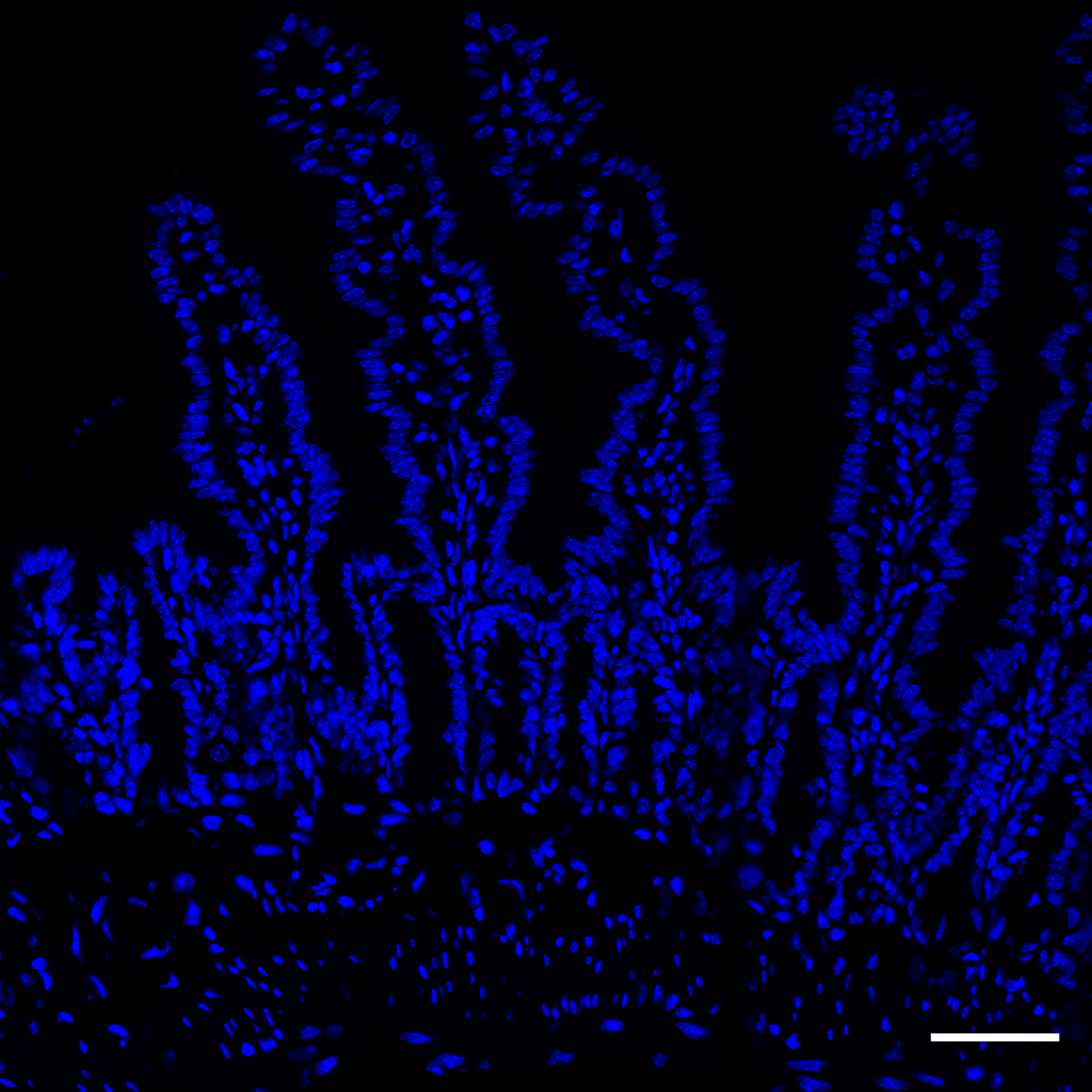

Supplement: Supplementary file 7 — Source data Fig. 3 [file 44318_2024_281_MOESM7_ESM.zip › Figure3/3L/GFP WT control.tif]

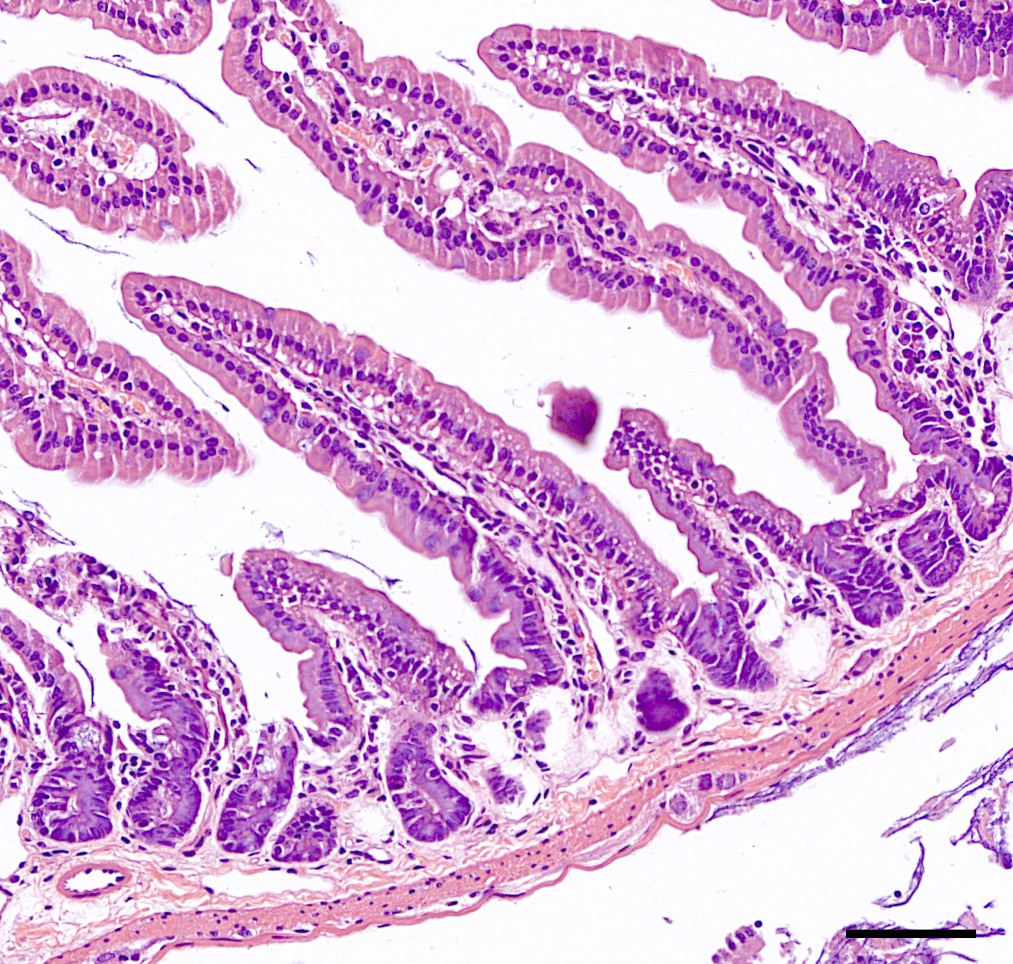

Supplement: Supplementary file 7 — Source data Fig. 3 [file 44318_2024_281_MOESM7_ESM.zip › Figure3/3M/HE LSR-OE Duodenum.tif]

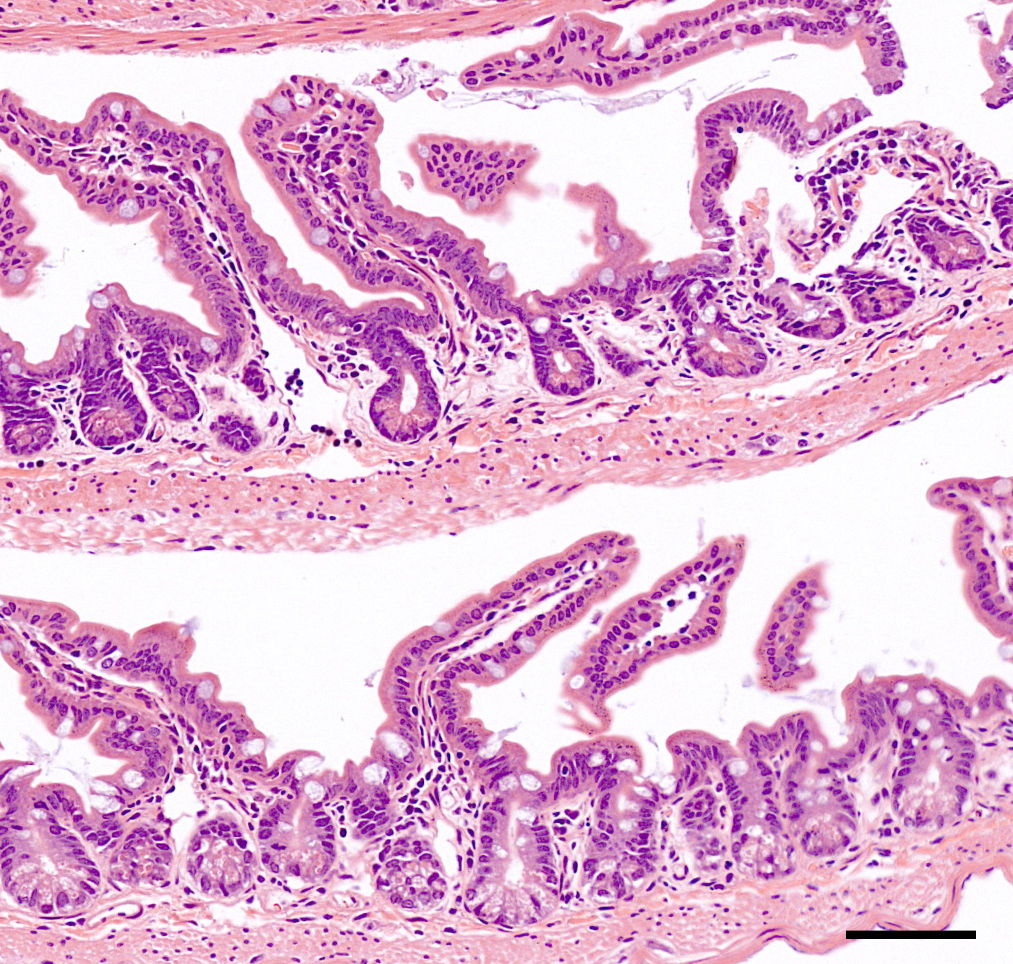

Supplement: Supplementary file 7 — Source data Fig. 3 [file 44318_2024_281_MOESM7_ESM.zip › Figure3/3M/HE LSR-OE Ileum.tif]

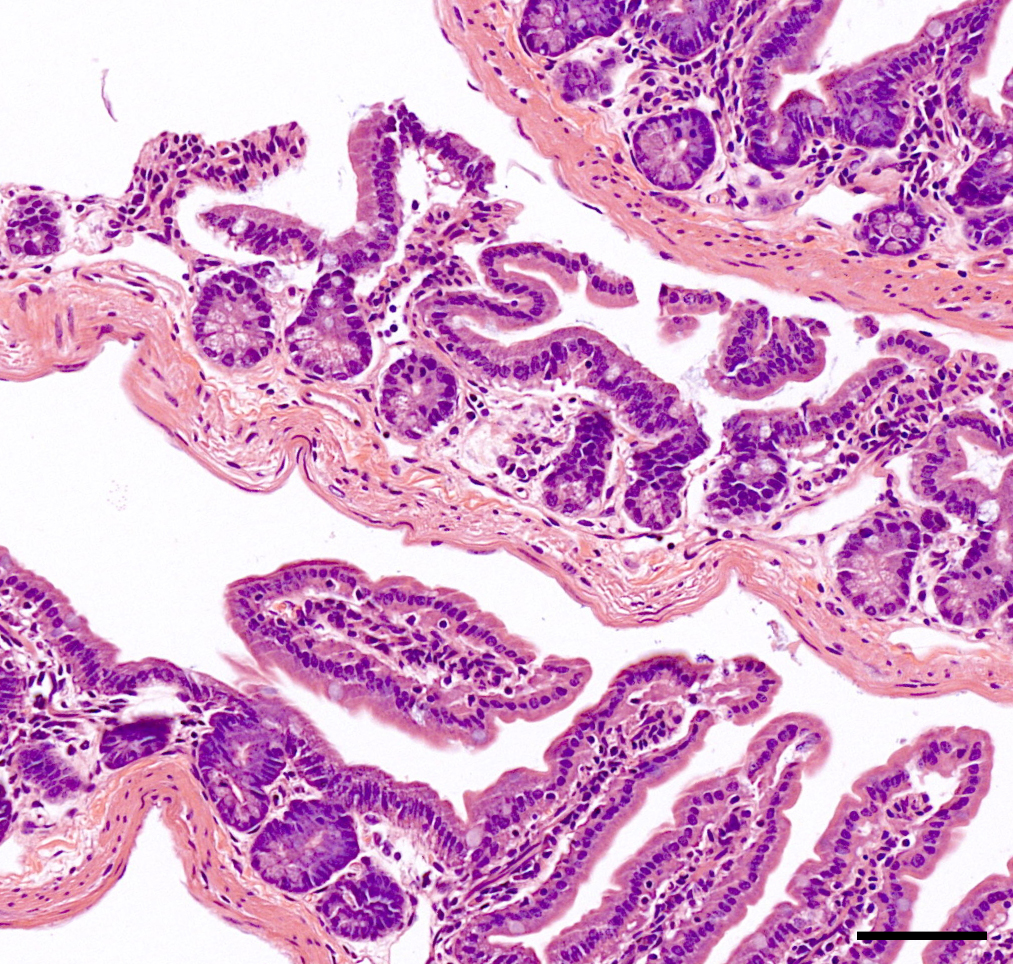

Supplement: Supplementary file 7 — Source data Fig. 3 [file 44318_2024_281_MOESM7_ESM.zip › Figure3/3M/HE LSR-OE Jejunum.tif]

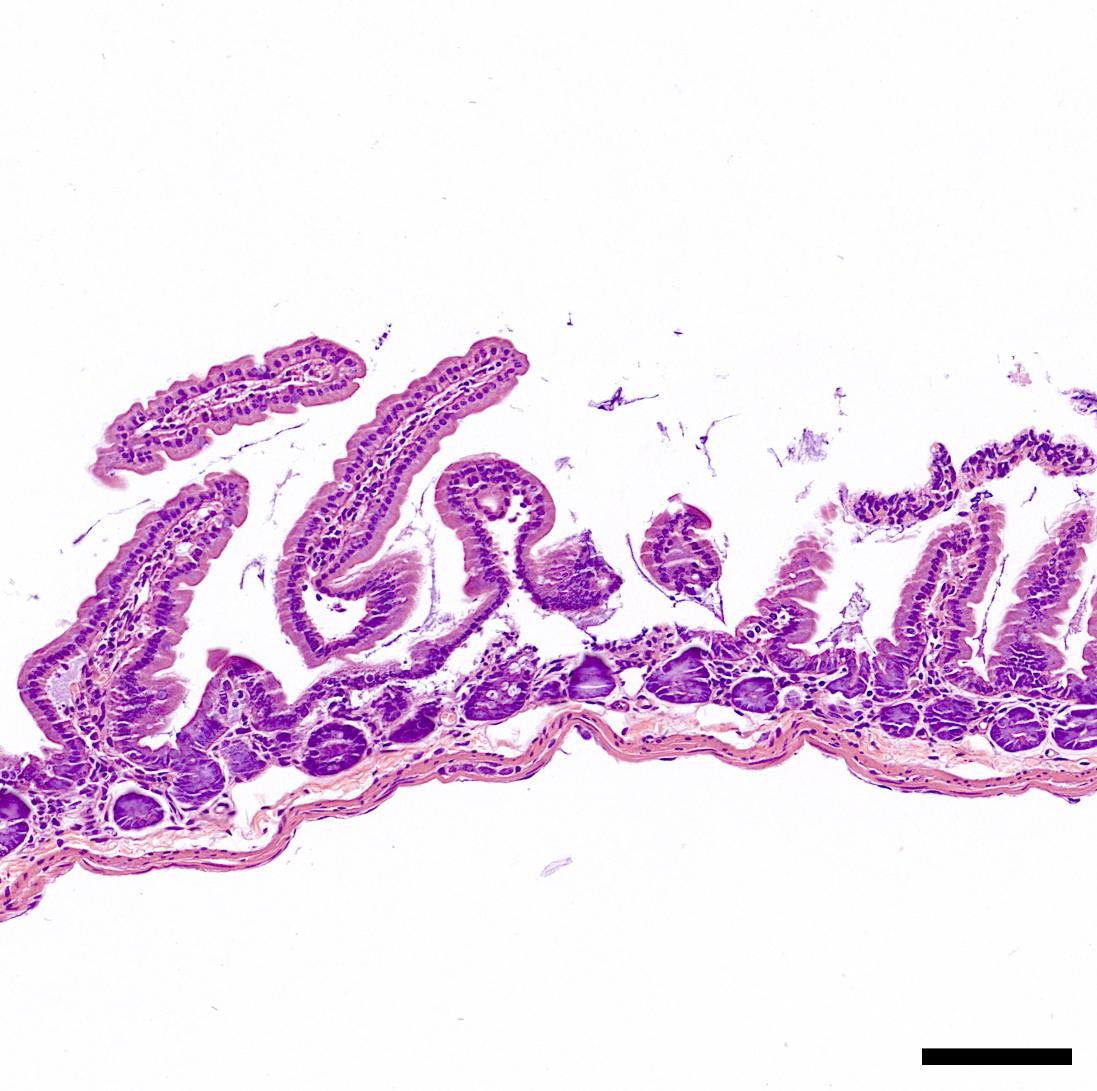

Supplement: Supplementary file 7 — Source data Fig. 3 [file 44318_2024_281_MOESM7_ESM.zip › Figure3/3M/HE WT Duodenum.tif]

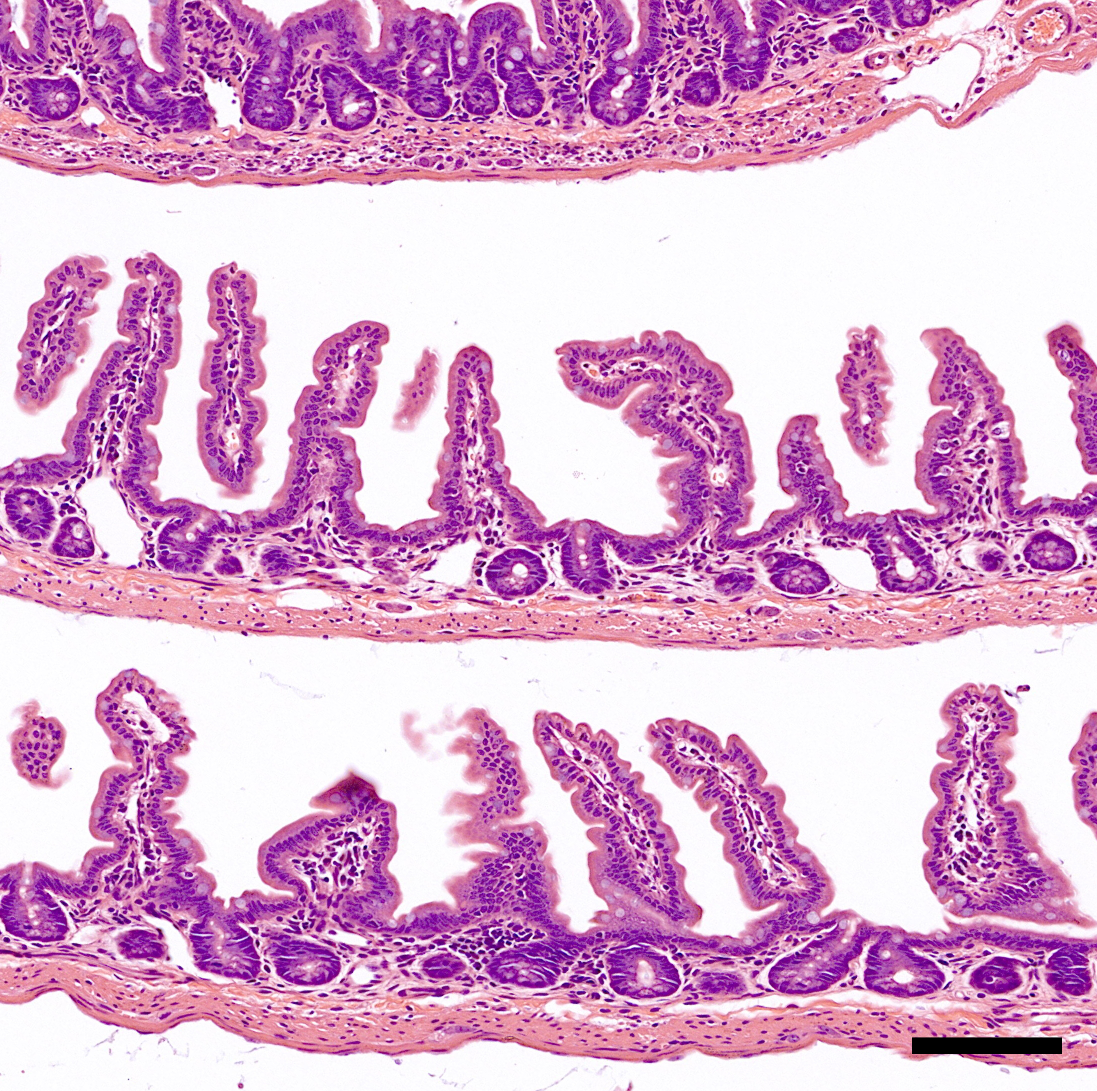

Supplement: Supplementary file 7 — Source data Fig. 3 [file 44318_2024_281_MOESM7_ESM.zip › Figure3/3M/HE WT Ileum.tif]

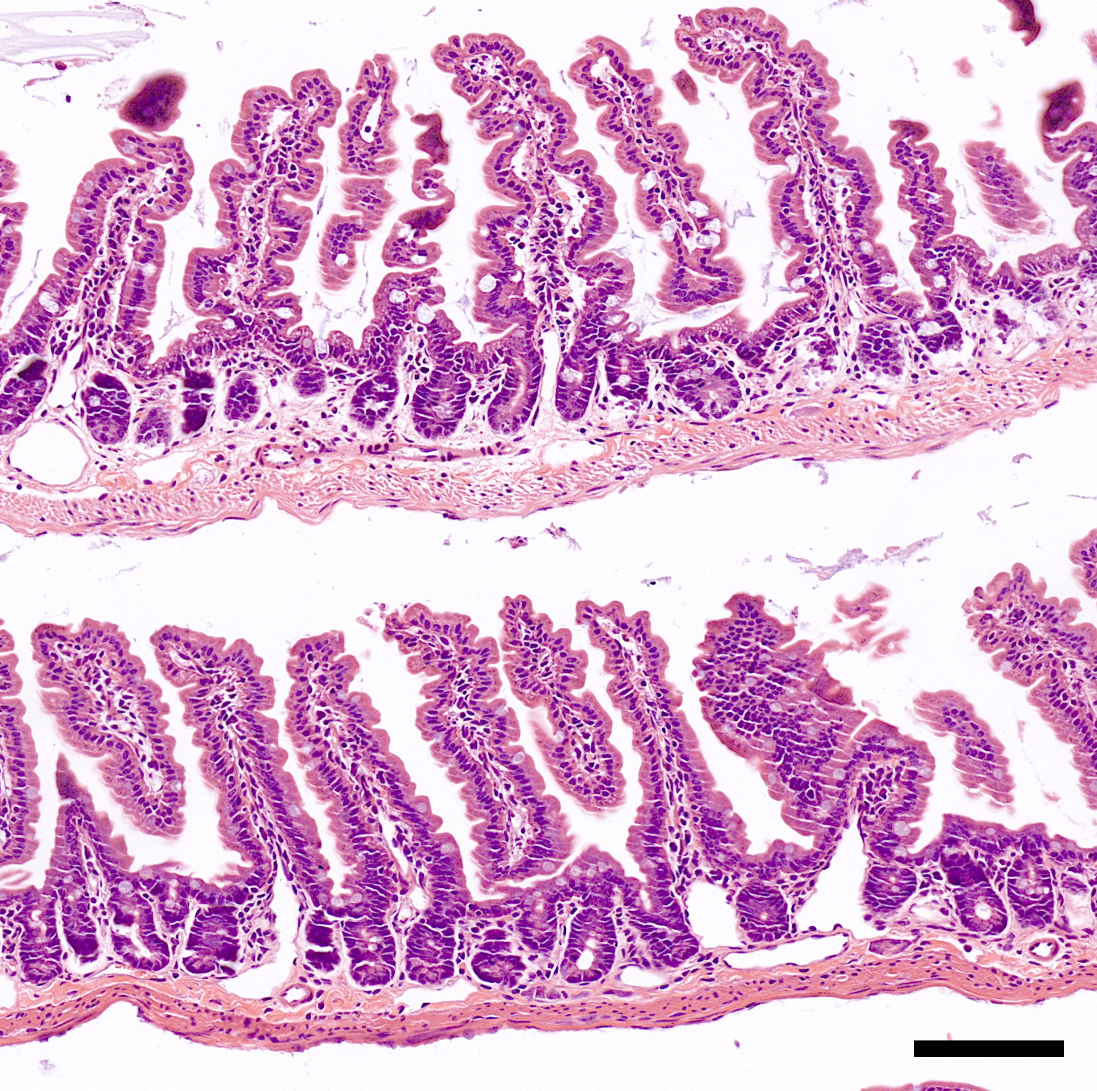

Supplement: Supplementary file 7 — Source data Fig. 3 [file 44318_2024_281_MOESM7_ESM.zip › Figure3/3M/HE WT Jejunum.tif]

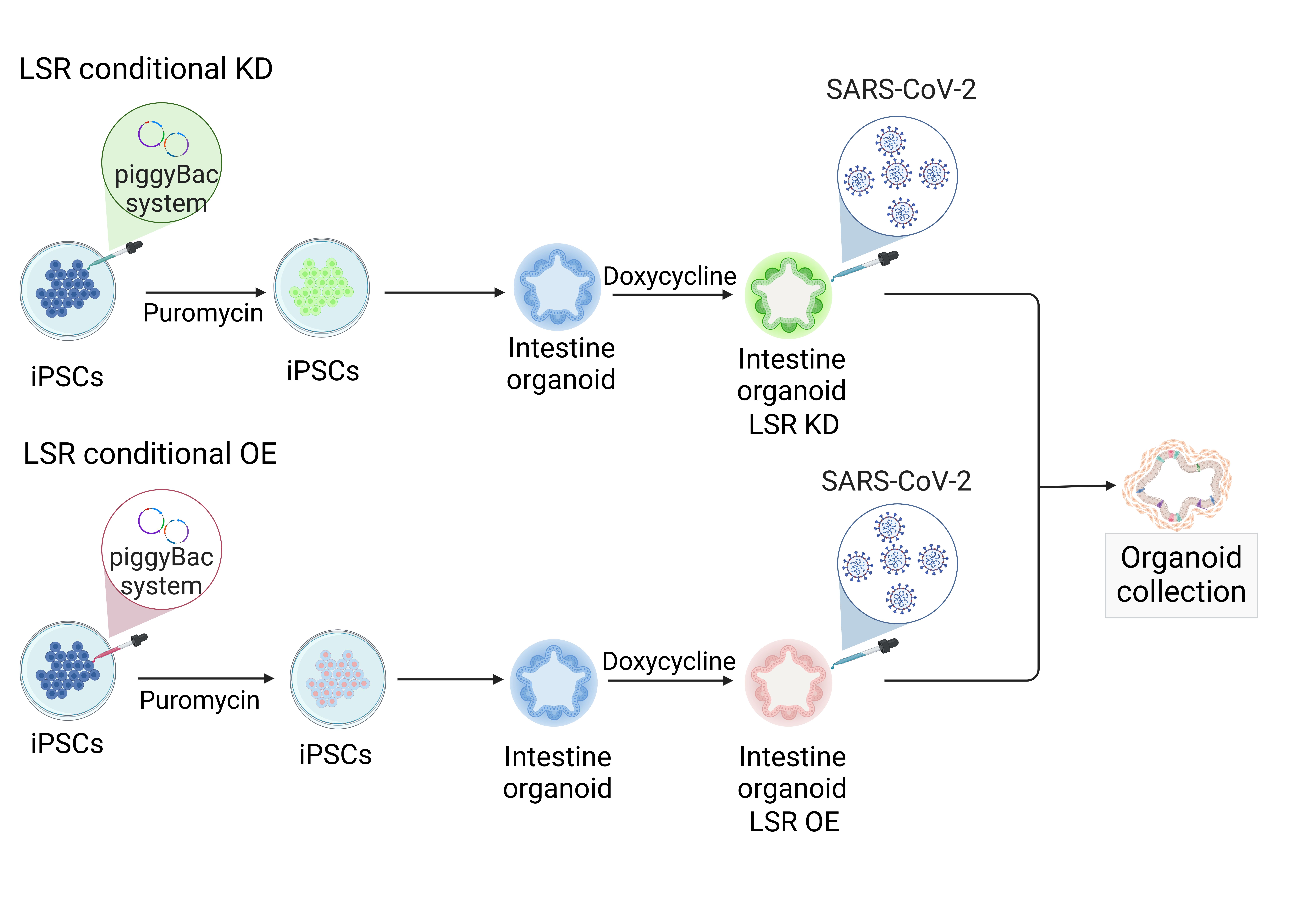

Supplement: Supplementary file 7 — Source data Fig. 3 [file 44318_2024_281_MOESM7_ESM.zip › Figure3/3O/SARS-CoV-2 infected organoids model.jpeg]

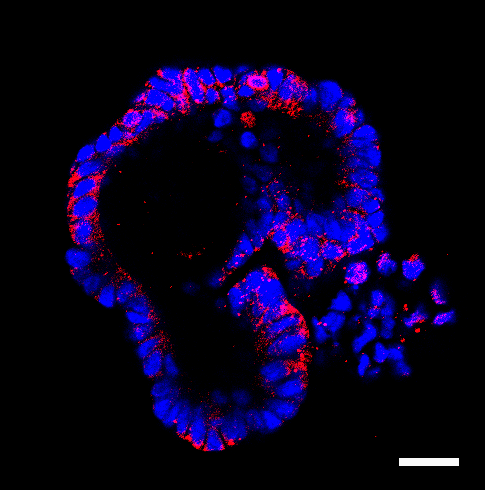

Supplement: Supplementary file 7 — Source data Fig. 3 [file 44318_2024_281_MOESM7_ESM.zip › Figure3/3Q/IF N protein Omicron LSR-KD.tif]

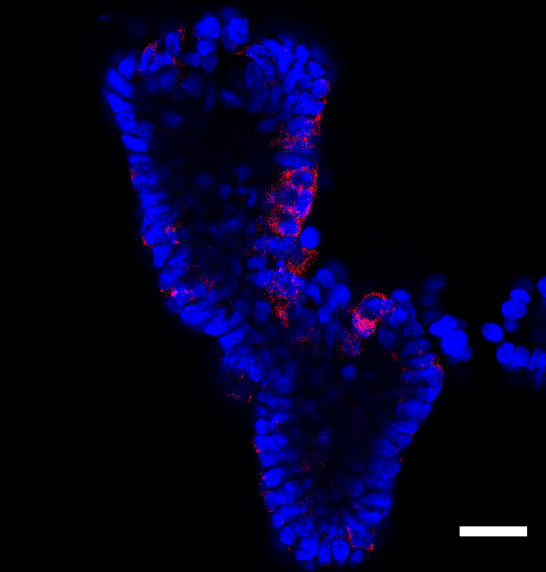

Supplement: Supplementary file 7 — Source data Fig. 3 [file 44318_2024_281_MOESM7_ESM.zip › Figure3/3Q/IF N protein Omicron LSR-OE.tif]

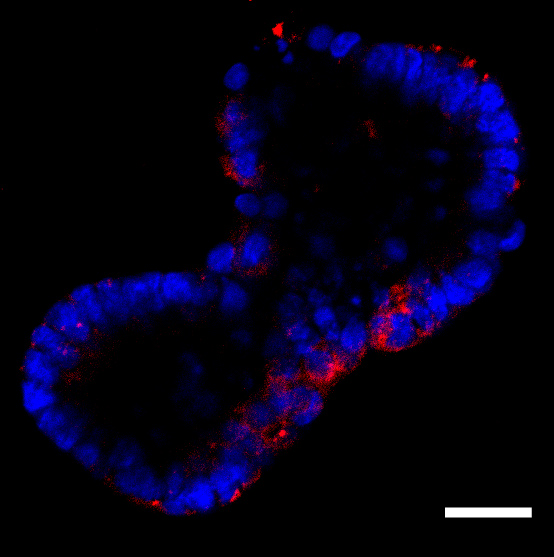

Supplement: Supplementary file 7 — Source data Fig. 3 [file 44318_2024_281_MOESM7_ESM.zip › Figure3/3Q/IF N protein Omicron Wildtype.tif]

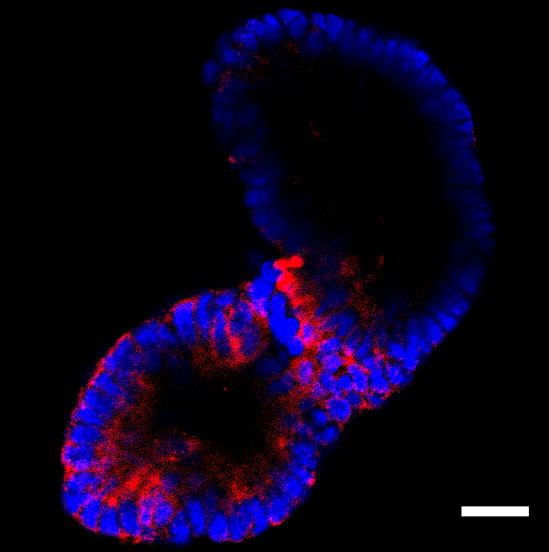

Supplement: Supplementary file 7 — Source data Fig. 3 [file 44318_2024_281_MOESM7_ESM.zip › Figure3/3Q/IF N protein SARS-CoV-2 WT LSR-KD.tif]

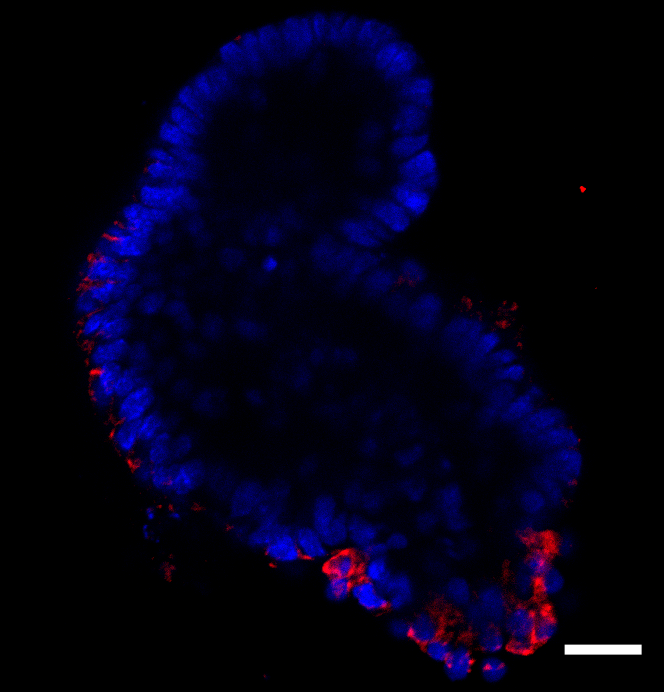

Supplement: Supplementary file 7 — Source data Fig. 3 [file 44318_2024_281_MOESM7_ESM.zip › Figure3/3Q/IF N protein SARS-CoV-2 WT LSR-OE.tif]

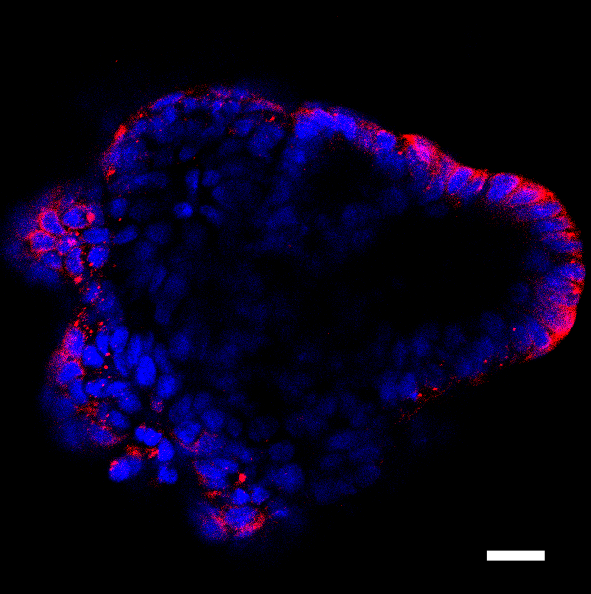

Supplement: Supplementary file 7 — Source data Fig. 3 [file 44318_2024_281_MOESM7_ESM.zip › Figure3/3Q/IF N protein SARS-CoV-2 WT Wildtype.tif]

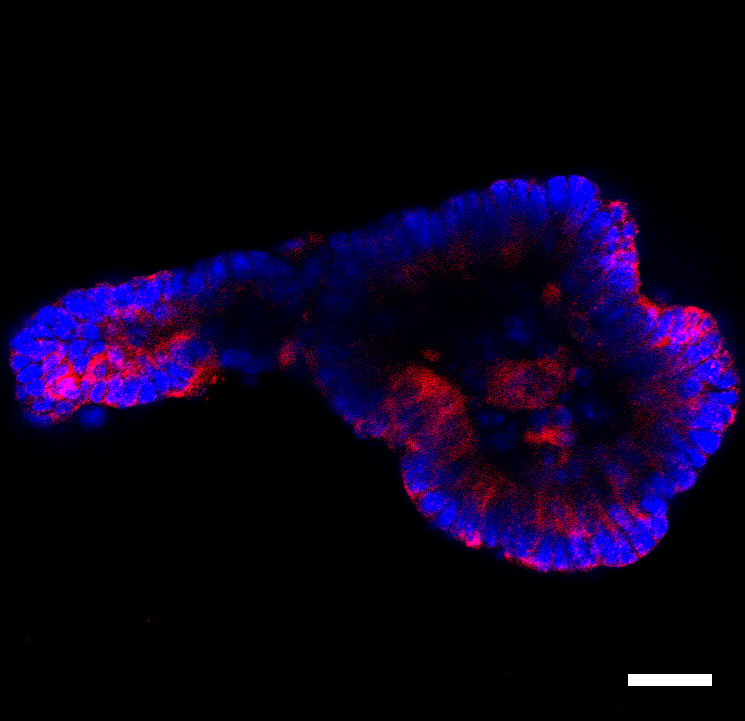

Supplement: Supplementary file 7 — Source data Fig. 3 [file 44318_2024_281_MOESM7_ESM.zip › Figure3/3Q/IF N protein XBB LSR-KD.tif]

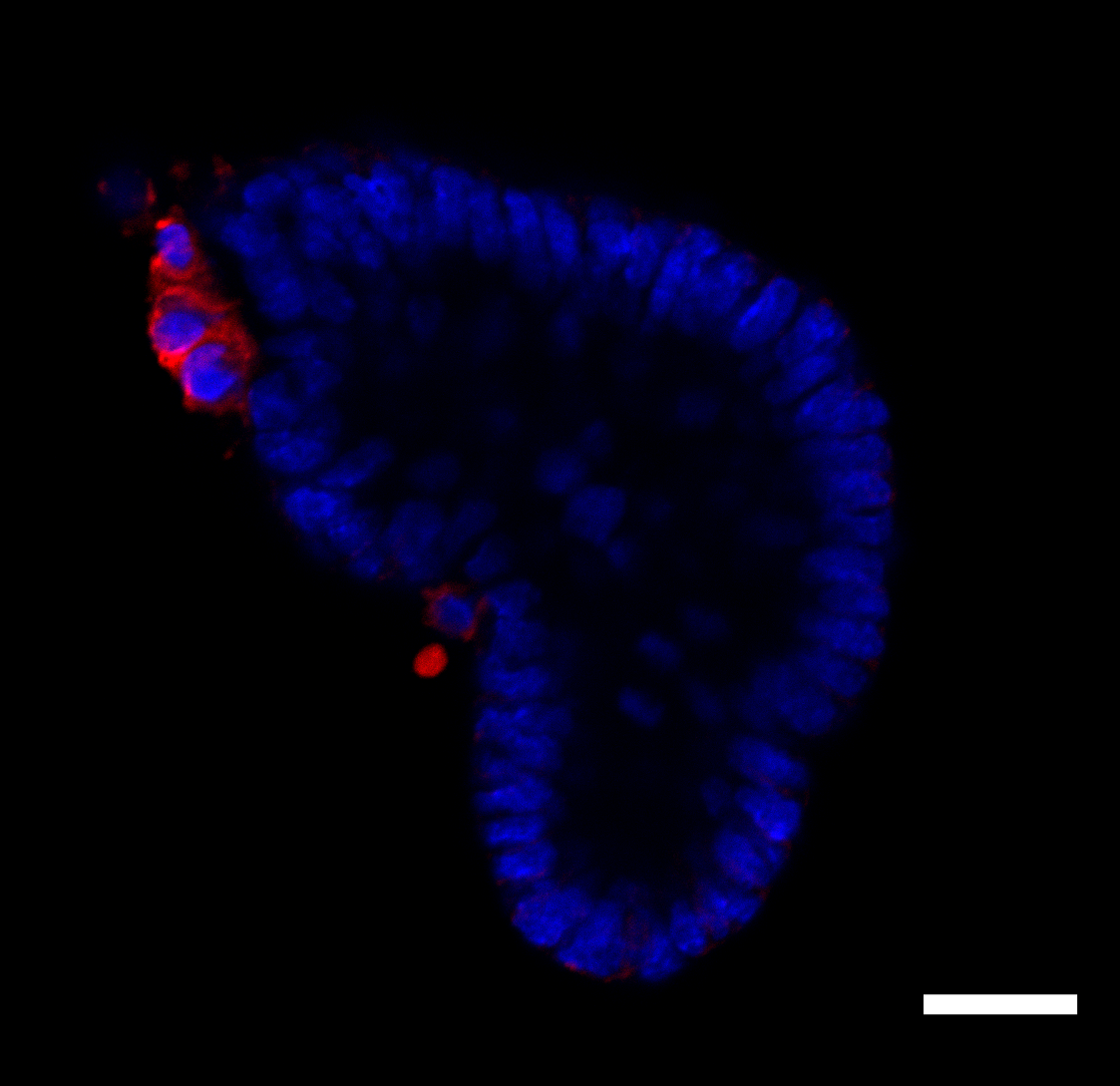

Supplement: Supplementary file 7 — Source data Fig. 3 [file 44318_2024_281_MOESM7_ESM.zip › Figure3/3Q/IF N protein XBB LSR-OE.tif]

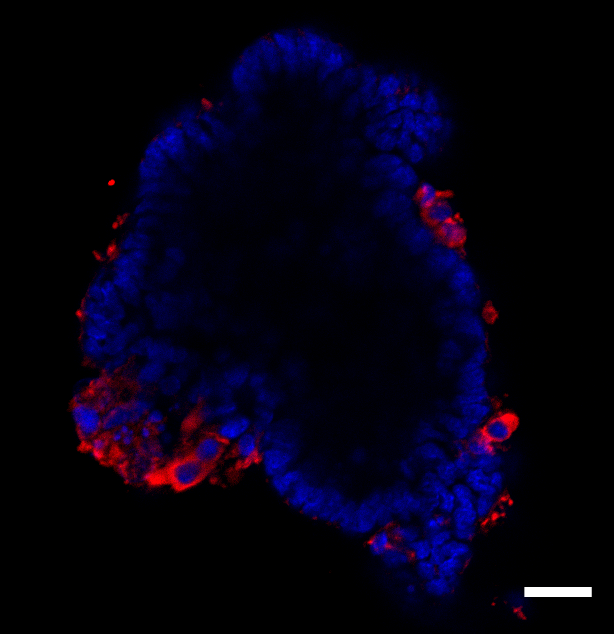

Supplement: Supplementary file 7 — Source data Fig. 3 [file 44318_2024_281_MOESM7_ESM.zip › Figure3/3Q/IF N protein XBB Wildtype.tif]

kDa

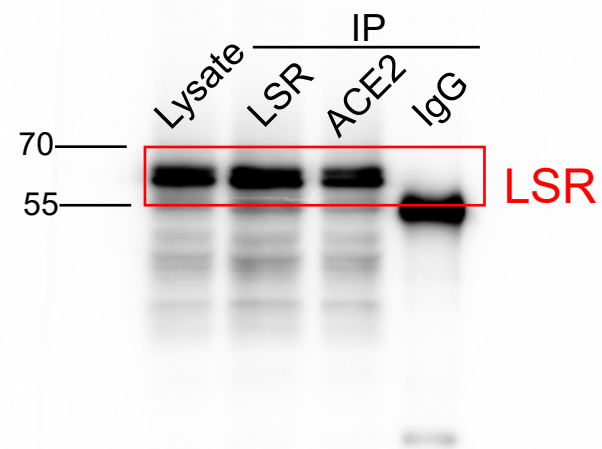

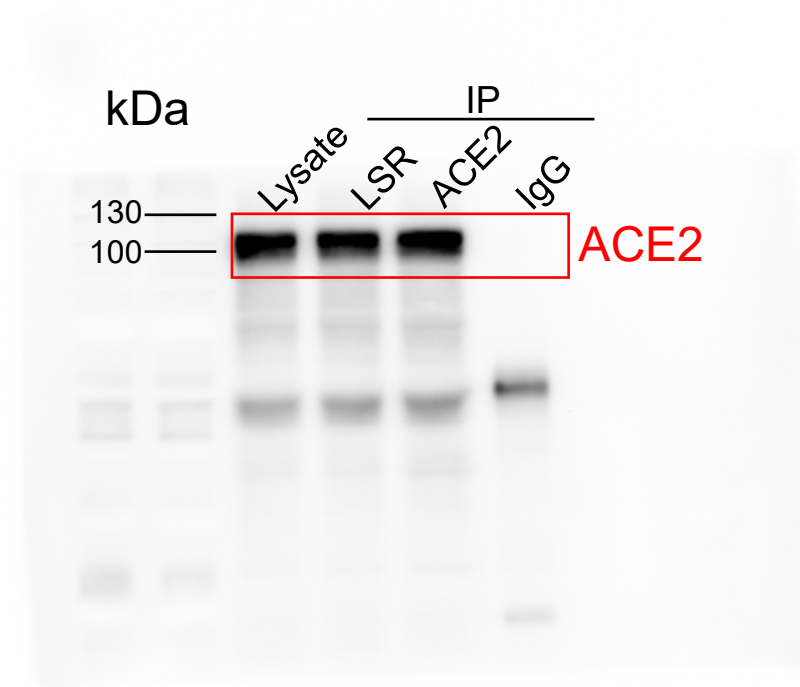

Supplement: Supplementary file 9 — Source data Fig. 5 [file 44318_2024_281_MOESM9_ESM.zip › Figure5/5A/5A.pdf]

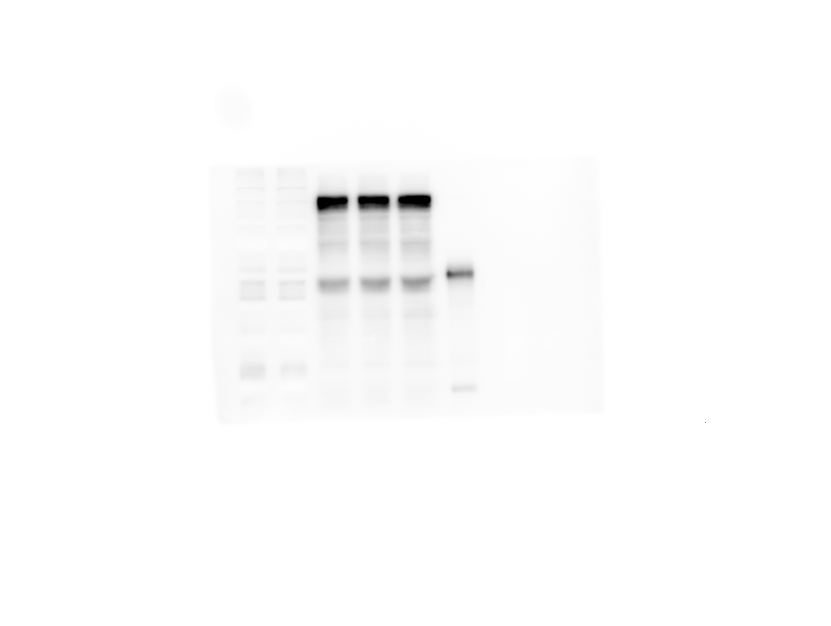

Supplement: Supplementary file 9 — Source data Fig. 5 [file 44318_2024_281_MOESM9_ESM.zip › Figure5/5A/western ACE2.png]

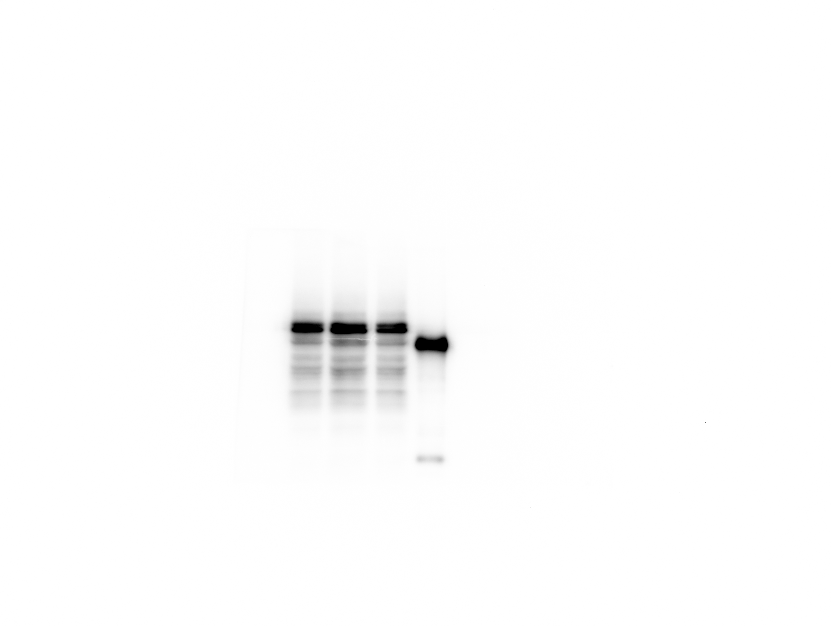

Supplement: Supplementary file 9 — Source data Fig. 5 [file 44318_2024_281_MOESM9_ESM.zip › Figure5/5A/western LSR.png]

kDa

Lysate Wildtype LSR-KD LSR-OE IgG

250

130

Spike

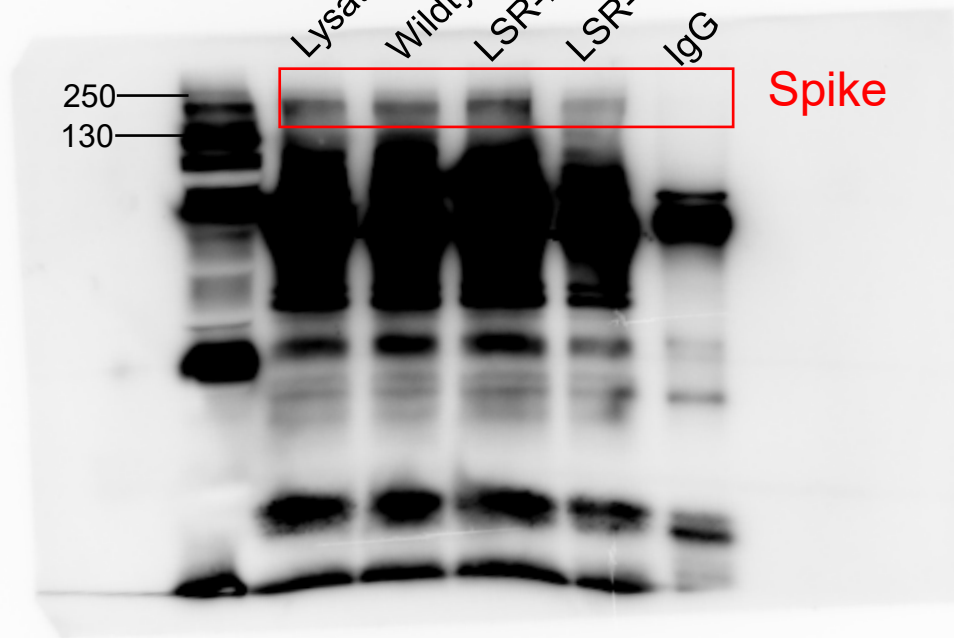

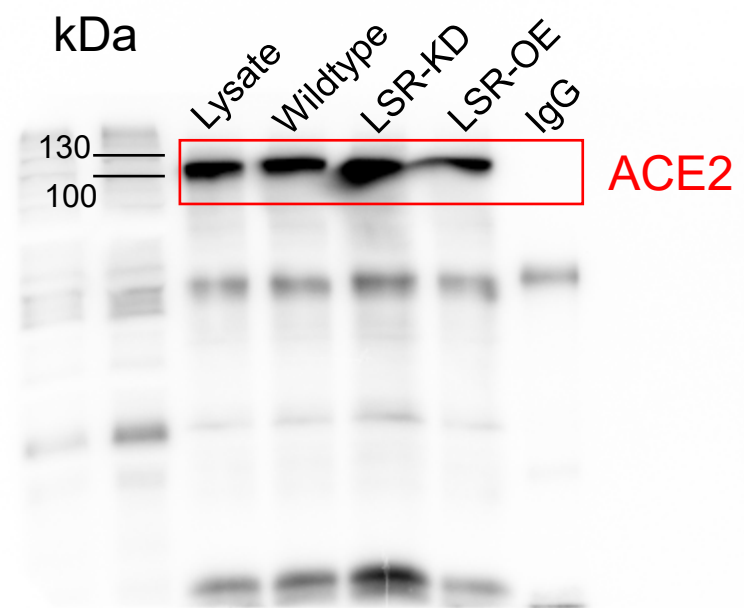

Supplement: Supplementary file 9 — Source data Fig. 5 [file 44318_2024_281_MOESM9_ESM.zip › Figure5/5B/5B.pdf]

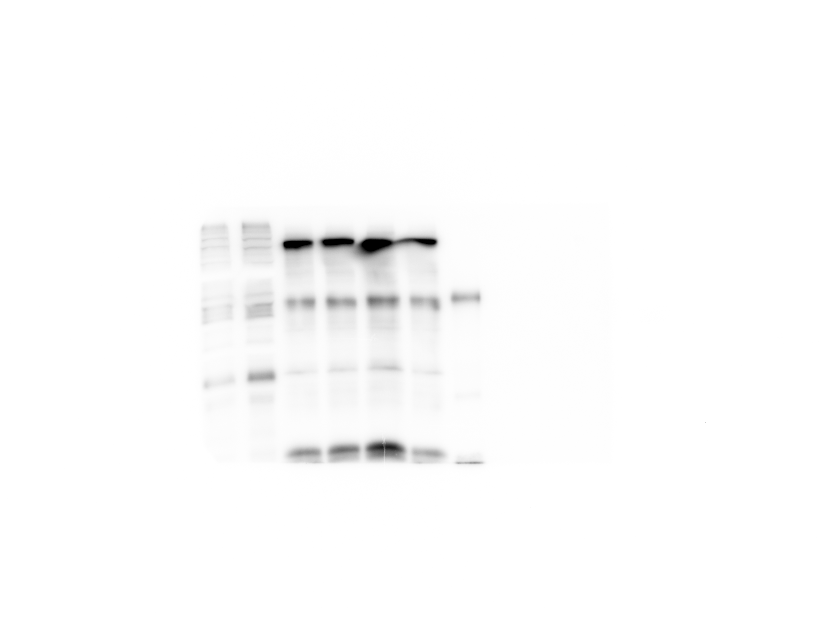

Supplement: Supplementary file 9 — Source data Fig. 5 [file 44318_2024_281_MOESM9_ESM.zip › Figure5/5B/western ACE2.png]

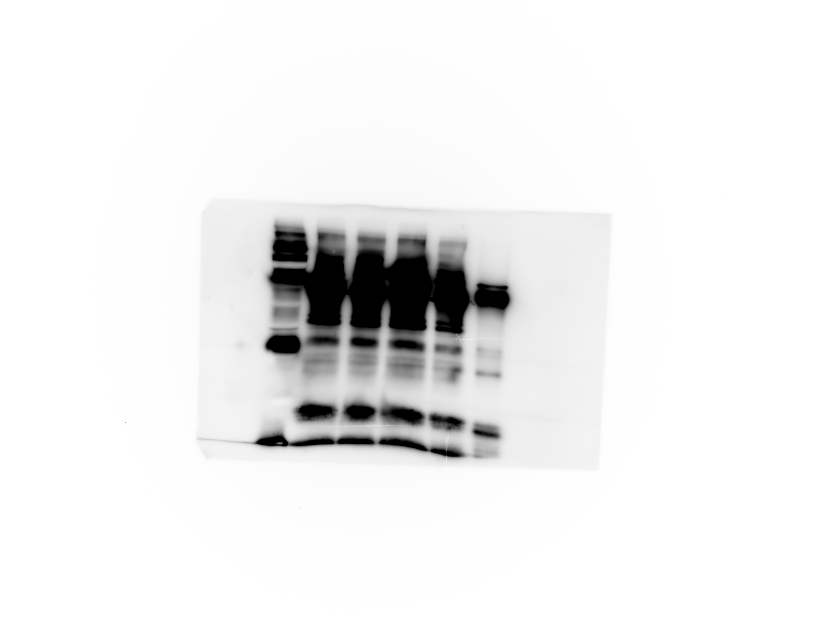

Supplement: Supplementary file 9 — Source data Fig. 5 [file 44318_2024_281_MOESM9_ESM.zip › Figure5/5B/western spike.png]

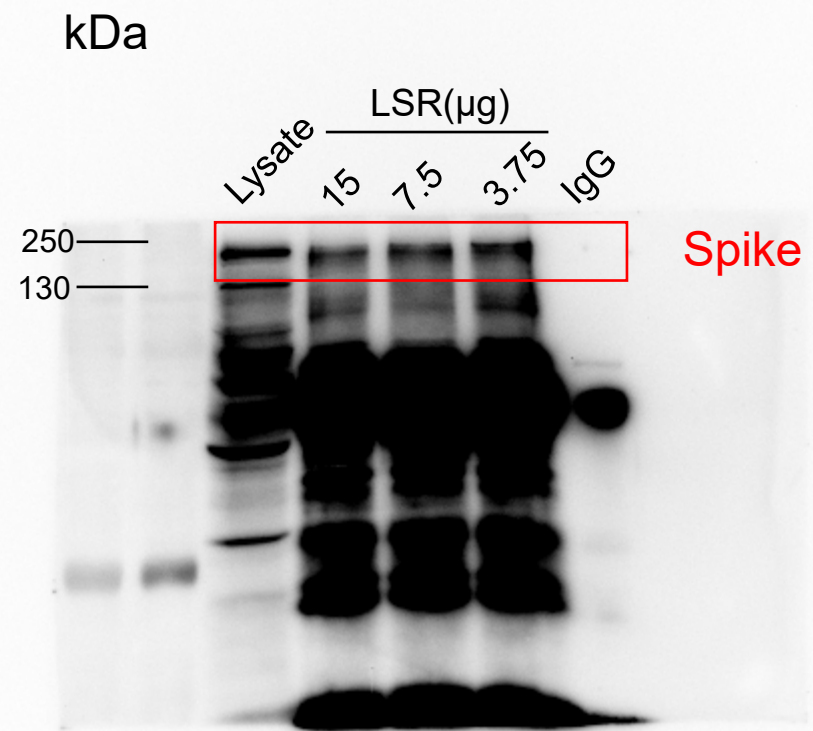

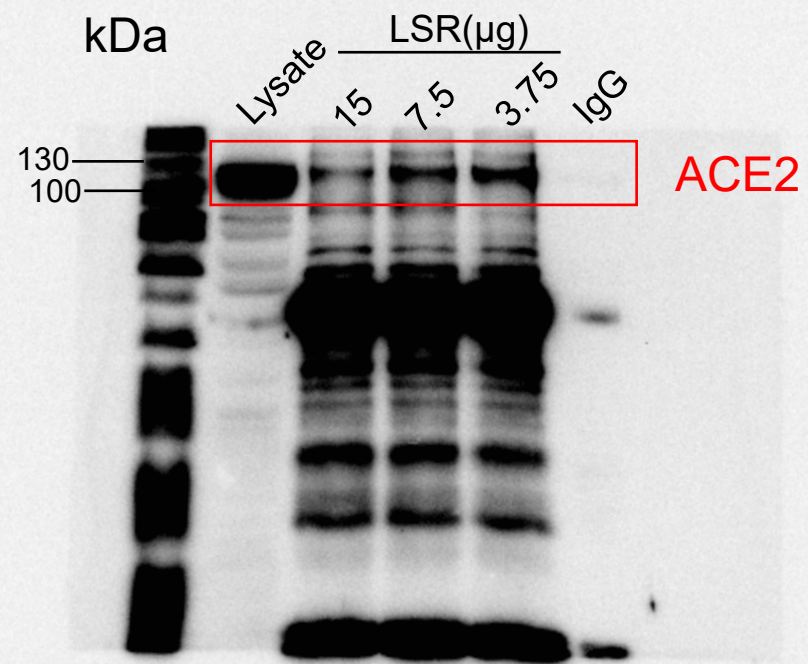

Supplement: Supplementary file 9 — Source data Fig. 5 [file 44318_2024_281_MOESM9_ESM.zip › Figure5/5C/5C.pdf]

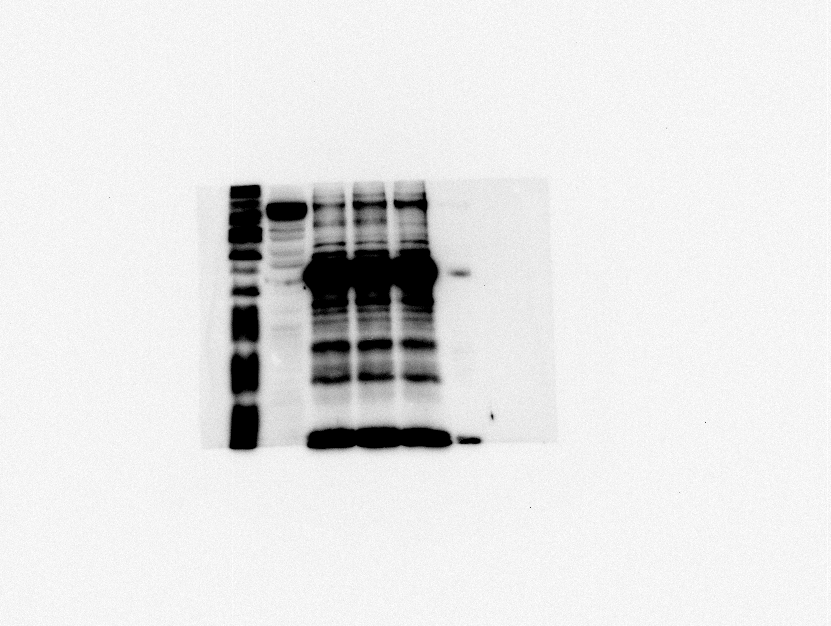

Supplement: Supplementary file 9 — Source data Fig. 5 [file 44318_2024_281_MOESM9_ESM.zip › Figure5/5C/western ACE2.png]

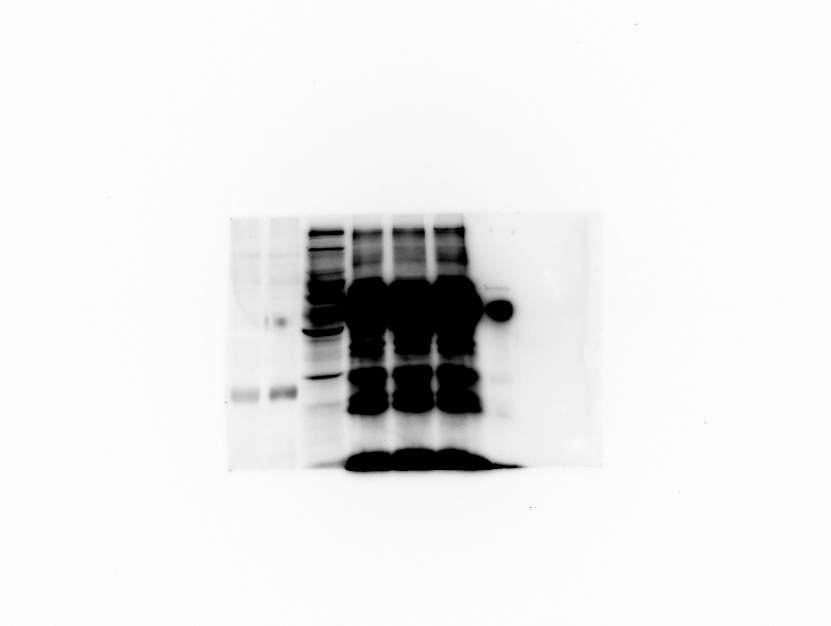

Supplement: Supplementary file 9 — Source data Fig. 5 [file 44318_2024_281_MOESM9_ESM.zip › Figure5/5C/western spike.png]

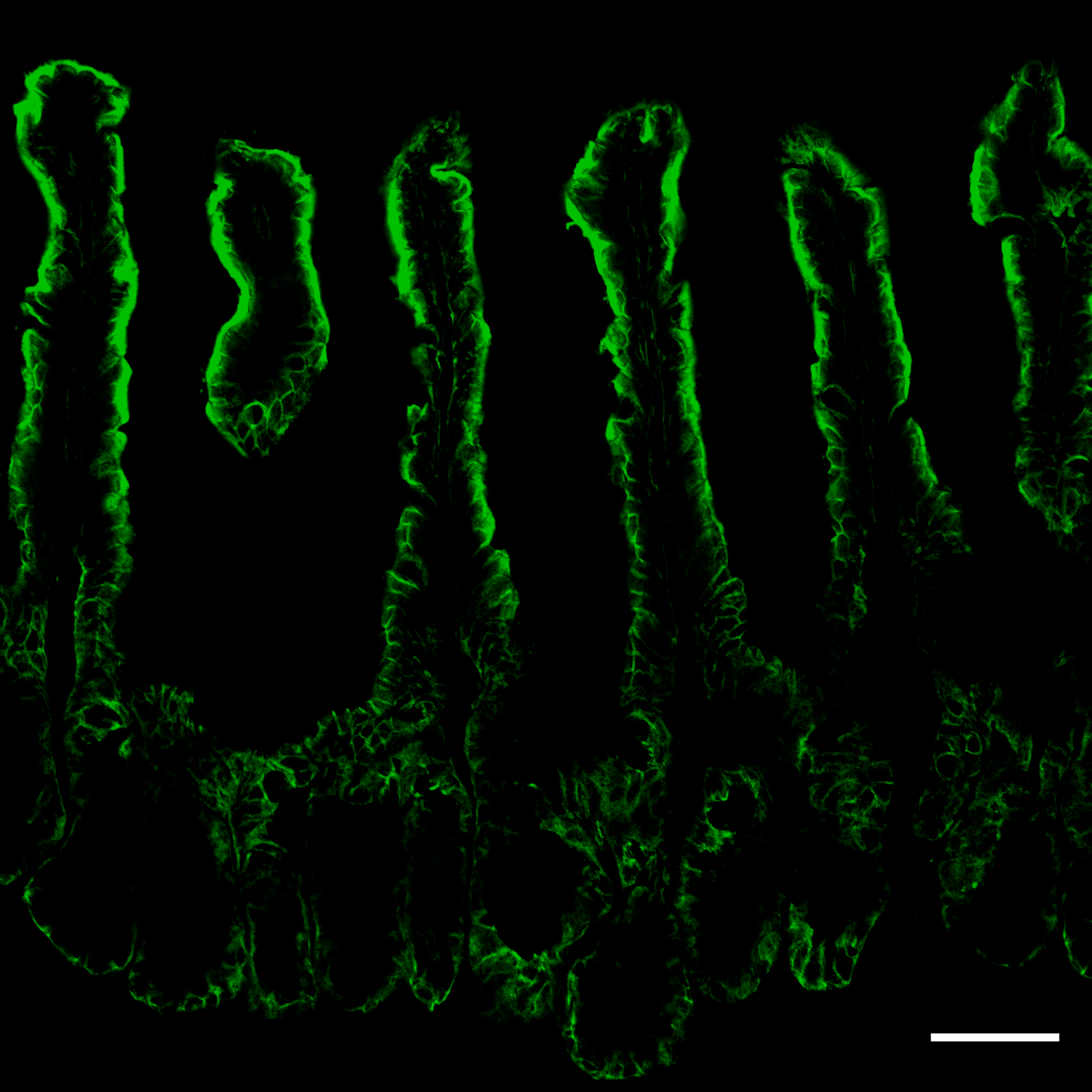

Supplement: Supplementary file 9 — Source data Fig. 5 [file 44318_2024_281_MOESM9_ESM.zip › Figure5/5E/IF ACE2.tif]

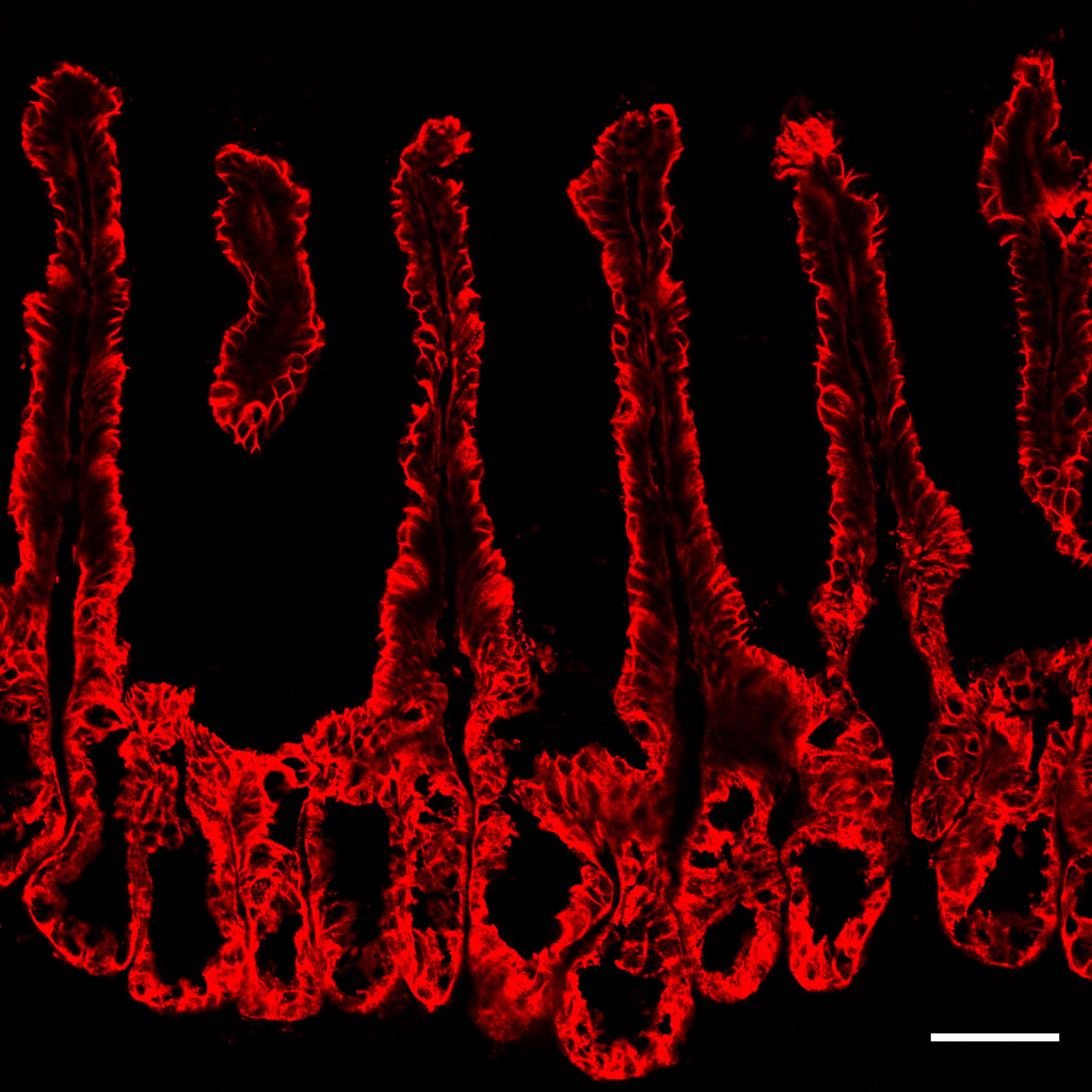

Supplement: Supplementary file 9 — Source data Fig. 5 [file 44318_2024_281_MOESM9_ESM.zip › Figure5/5E/IF LSR.tif]

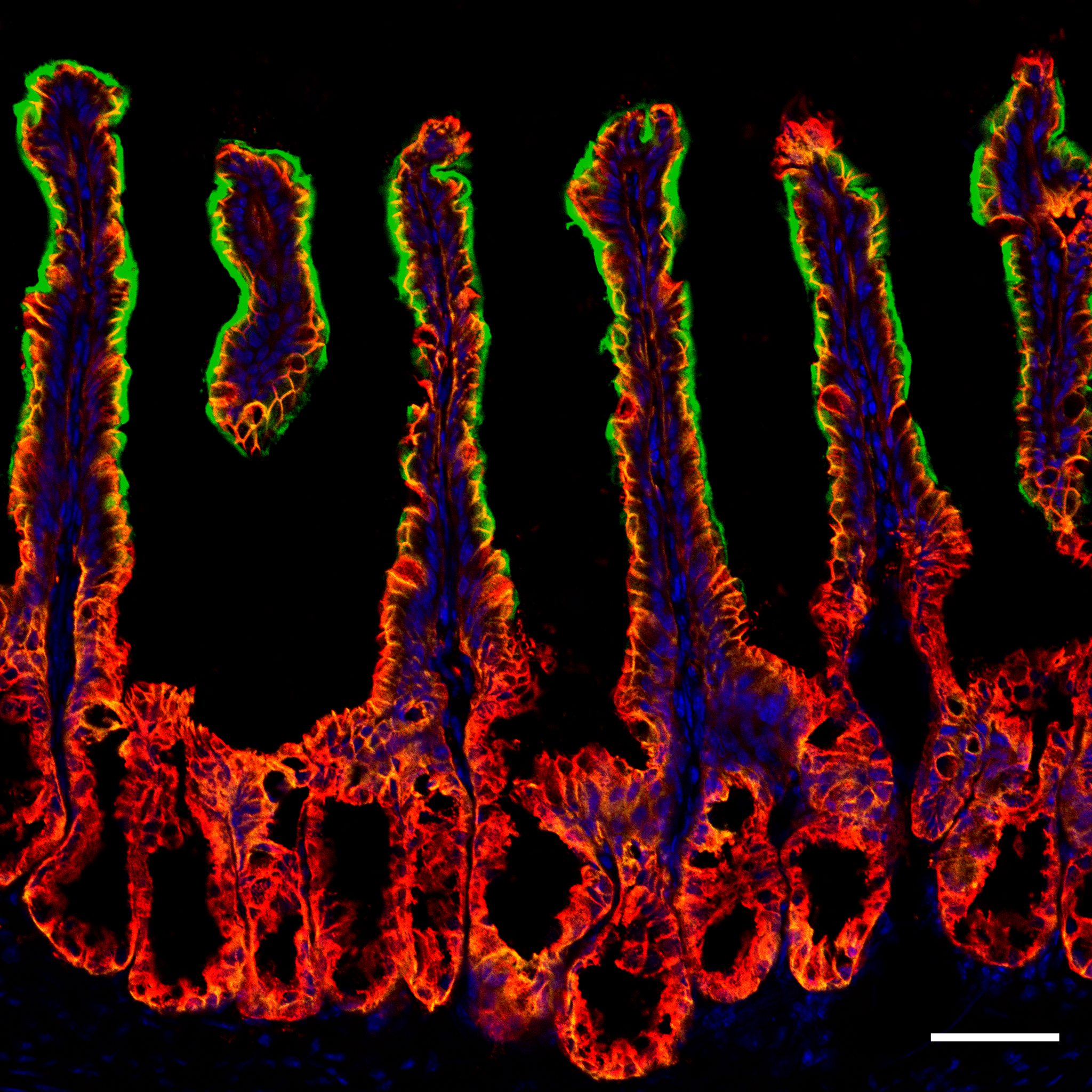

Supplement: Supplementary file 9 — Source data Fig. 5 [file 44318_2024_281_MOESM9_ESM.zip › Figure5/5E/IF merge.tif]

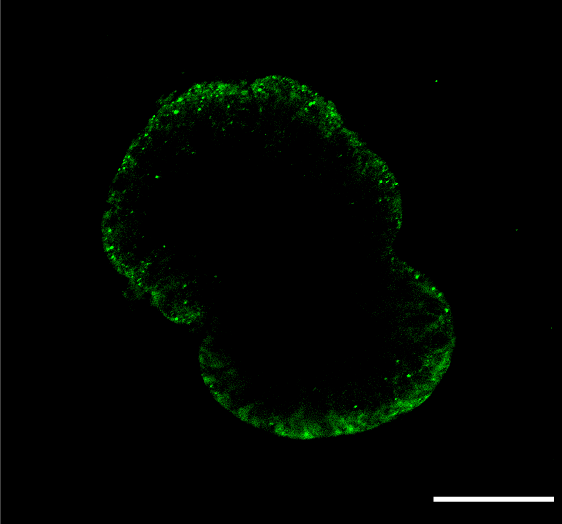

Supplement: Supplementary file 9 — Source data Fig. 5 [file 44318_2024_281_MOESM9_ESM.zip › Figure5/5F/IF ACE2.tif]

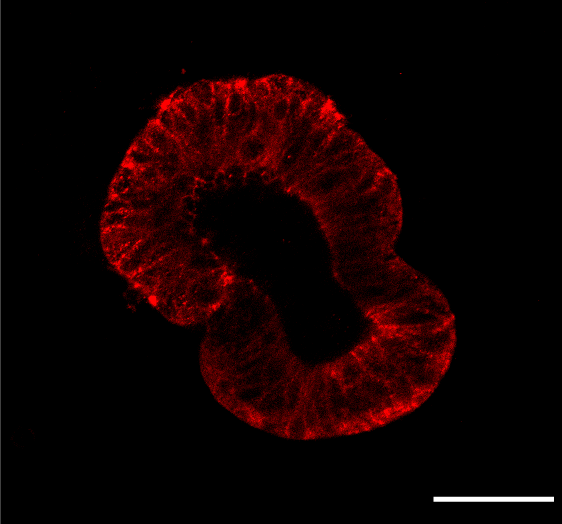

Supplement: Supplementary file 9 — Source data Fig. 5 [file 44318_2024_281_MOESM9_ESM.zip › Figure5/5F/IF LSR.tif]

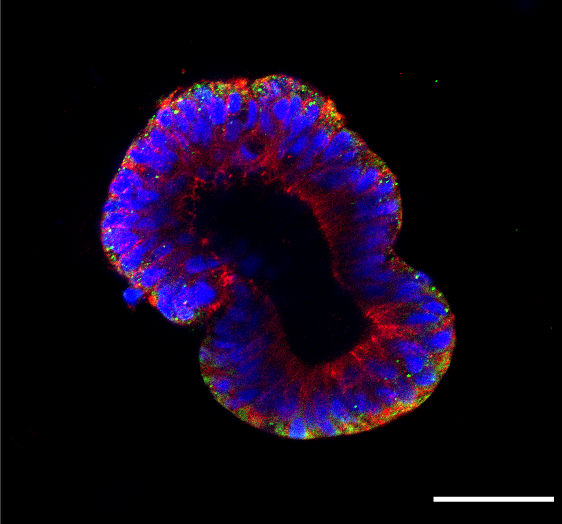

Supplement: Supplementary file 9 — Source data Fig. 5 [file 44318_2024_281_MOESM9_ESM.zip › Figure5/5F/IF merge.tif]

kDa

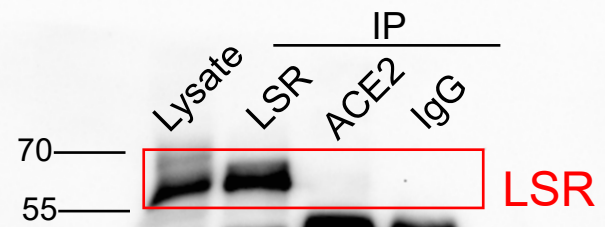

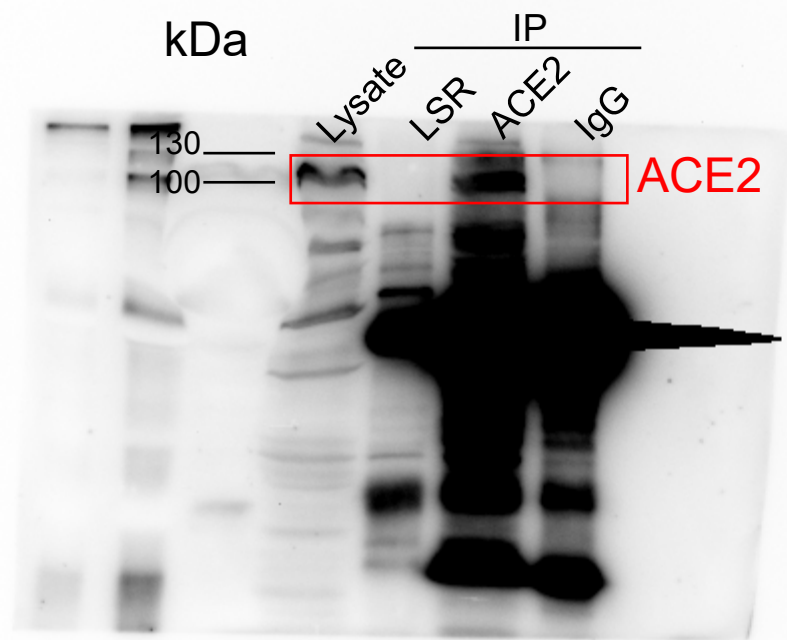

Supplement: Supplementary file 9 — Source data Fig. 5 [file 44318_2024_281_MOESM9_ESM.zip › Figure5/5G/5G.pdf]

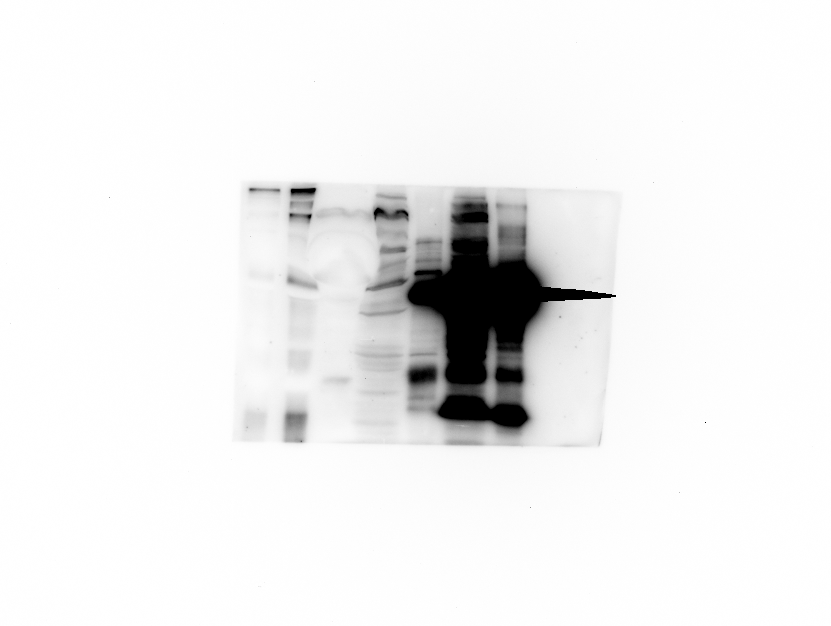

Supplement: Supplementary file 9 — Source data Fig. 5 [file 44318_2024_281_MOESM9_ESM.zip › Figure5/5G/western ACE2.png]

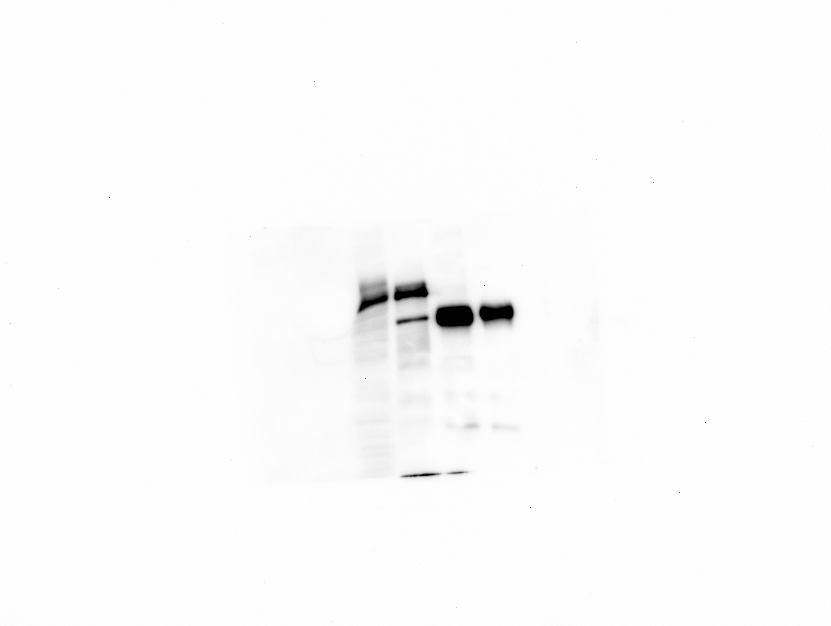

Supplement: Supplementary file 9 — Source data Fig. 5 [file 44318_2024_281_MOESM9_ESM.zip › Figure5/5G/western LSR.png]

kDa

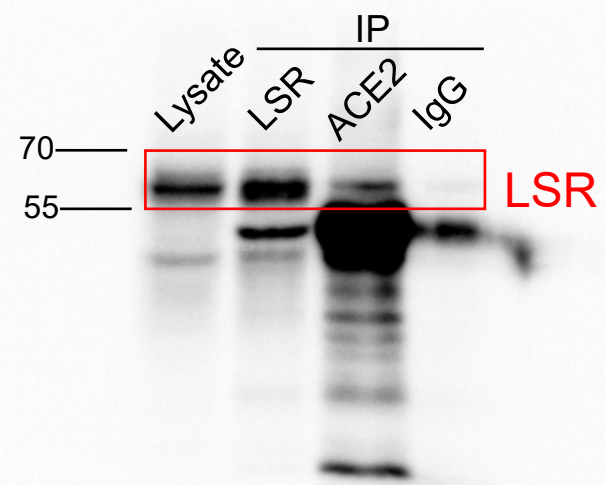

LSR

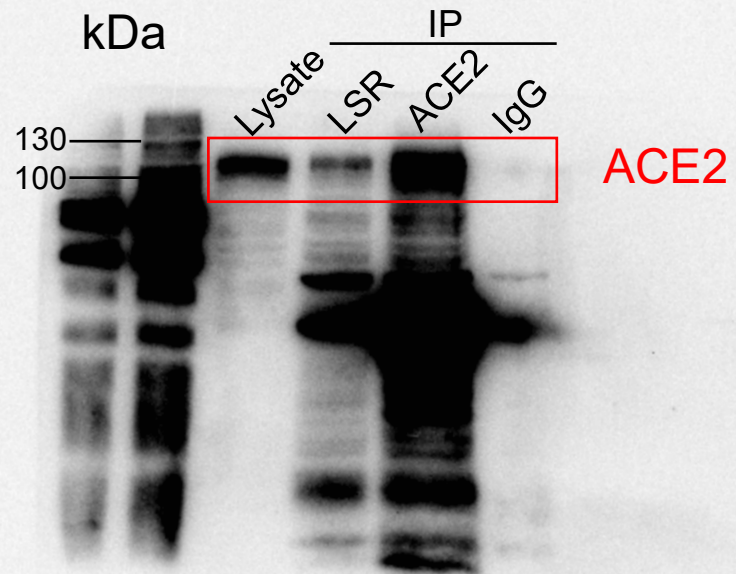

Supplement: Supplementary file 9 — Source data Fig. 5 [file 44318_2024_281_MOESM9_ESM.zip › Figure5/5H/5H.pdf]

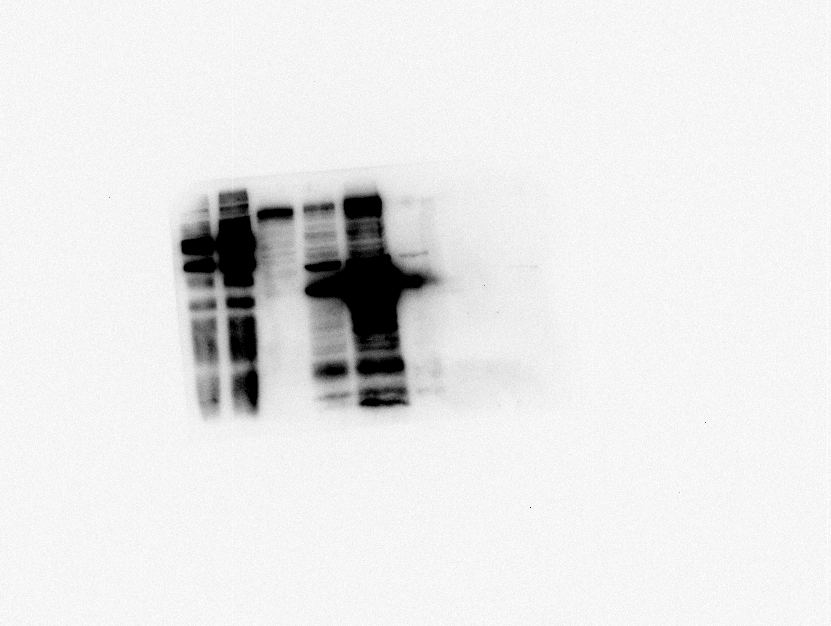

Supplement: Supplementary file 9 — Source data Fig. 5 [file 44318_2024_281_MOESM9_ESM.zip › Figure5/5H/western ACE2.png]

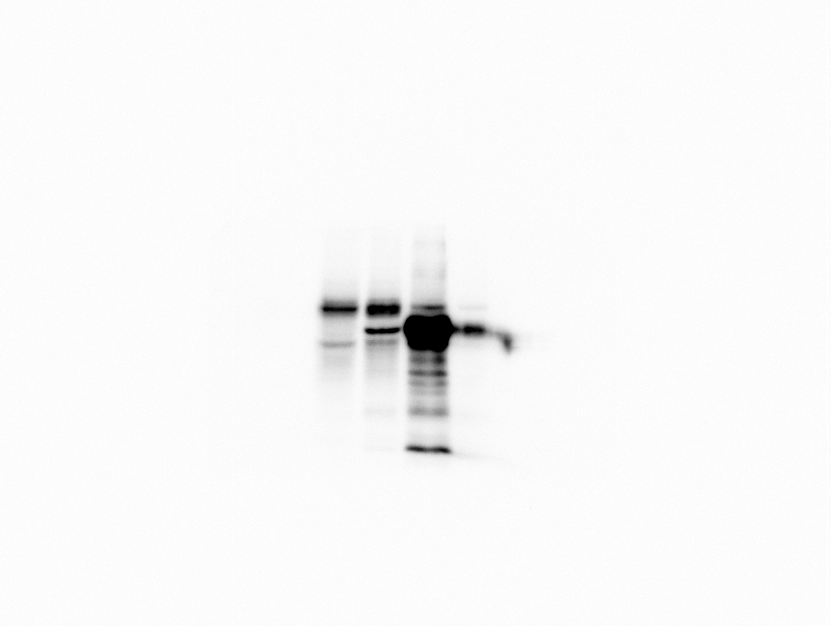

Supplement: Supplementary file 9 — Source data Fig. 5 [file 44318_2024_281_MOESM9_ESM.zip › Figure5/5H/western LSR.png]

kDa

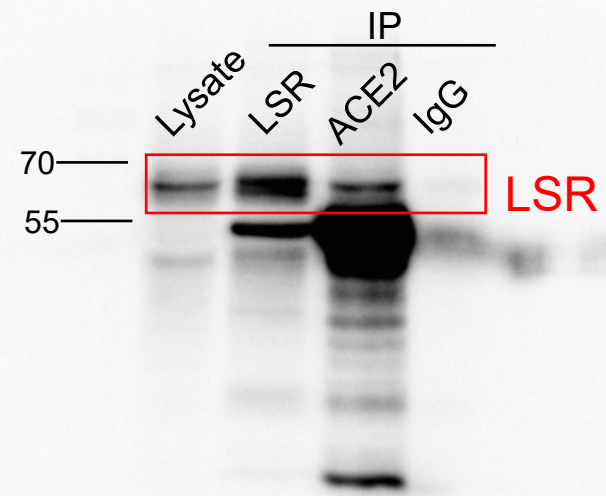

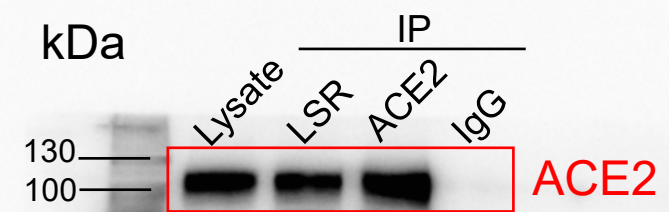

Supplement: Supplementary file 9 — Source data Fig. 5 [file 44318_2024_281_MOESM9_ESM.zip › Figure5/5I/5I.pdf]

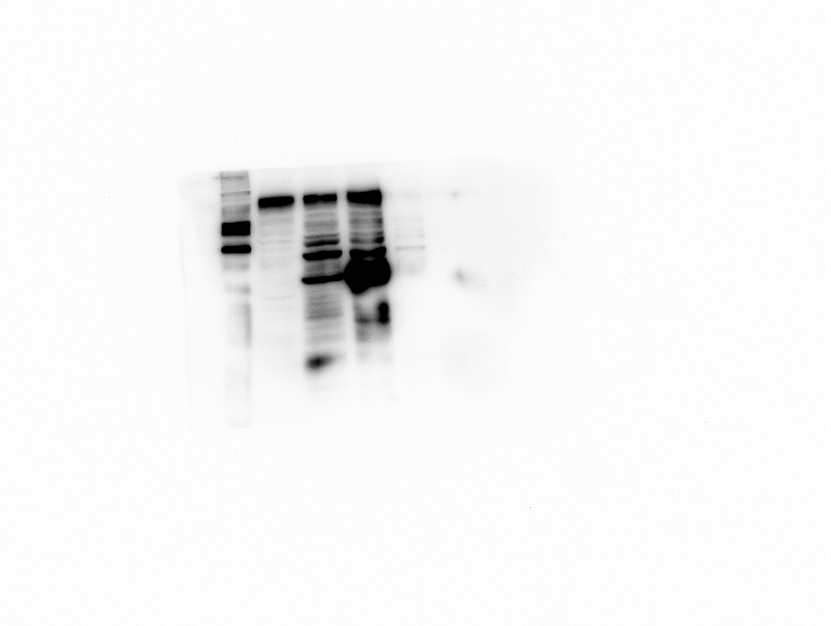

Supplement: Supplementary file 9 — Source data Fig. 5 [file 44318_2024_281_MOESM9_ESM.zip › Figure5/5I/western ACE2.png]

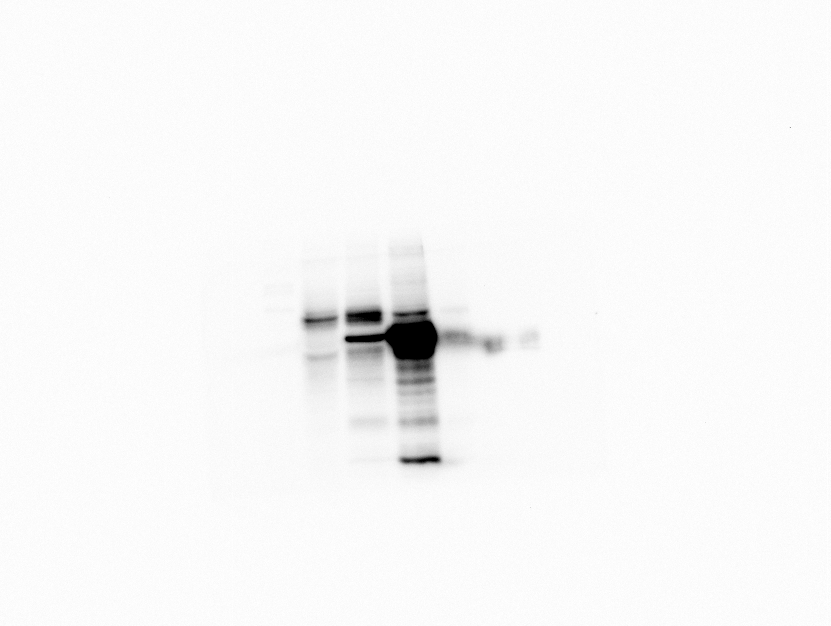

Supplement: Supplementary file 9 — Source data Fig. 5 [file 44318_2024_281_MOESM9_ESM.zip › Figure5/5I/western LSR.png]

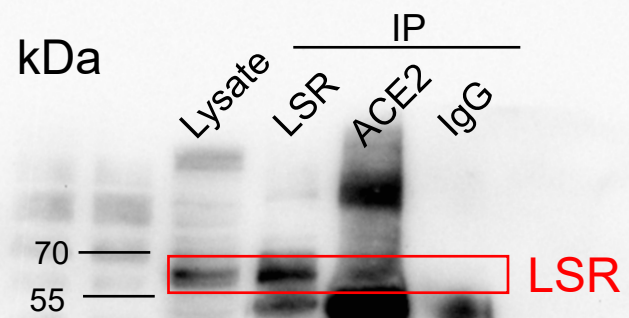

kDa

130

100

Lysate

LSR

ACE2

IgG

IP

ACE2

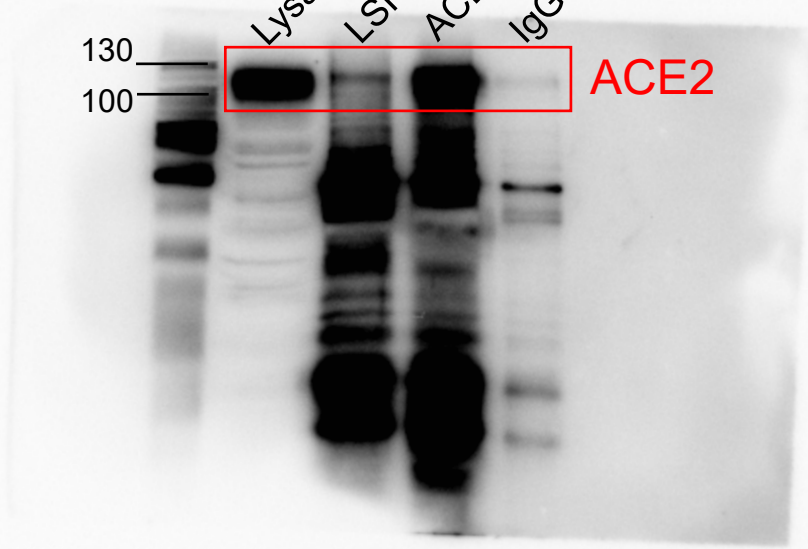

Supplement: Supplementary file 9 — Source data Fig. 5 [file 44318_2024_281_MOESM9_ESM.zip › Figure5/5J/5J.pdf]

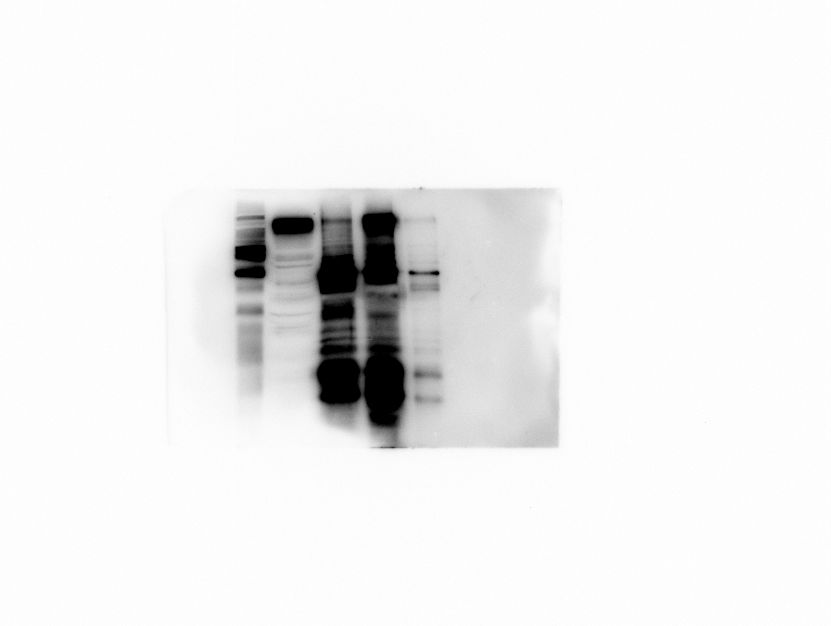

Supplement: Supplementary file 9 — Source data Fig. 5 [file 44318_2024_281_MOESM9_ESM.zip › Figure5/5J/western ACE2.png]

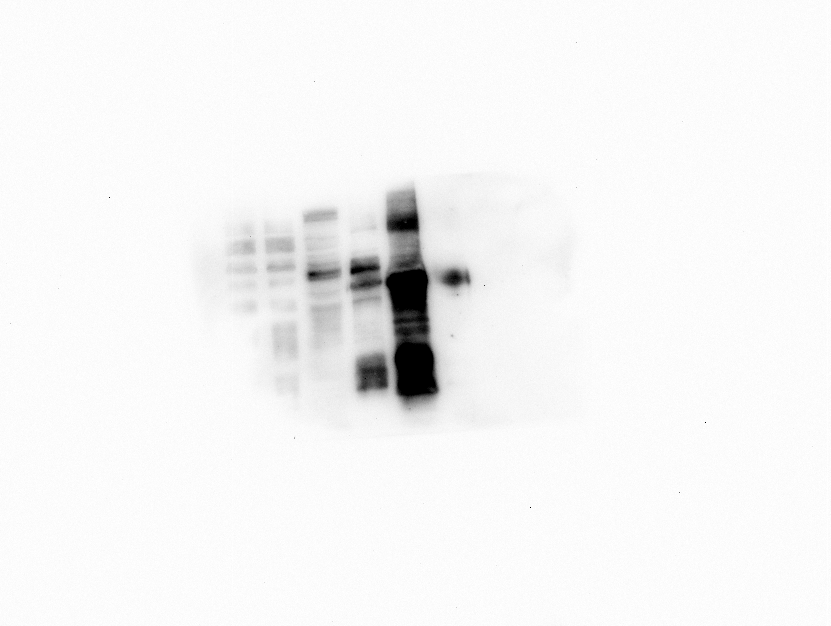

Supplement: Supplementary file 9 — Source data Fig. 5 [file 44318_2024_281_MOESM9_ESM.zip › Figure5/5J/western LSR.png]

kDa

Lysate

IP

LSR

ACE2

IgG

70

55

LSR

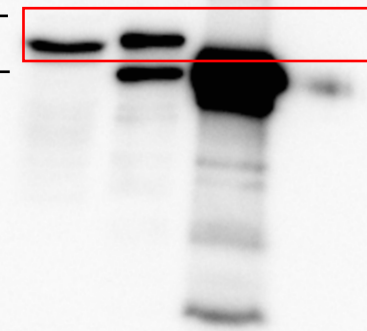

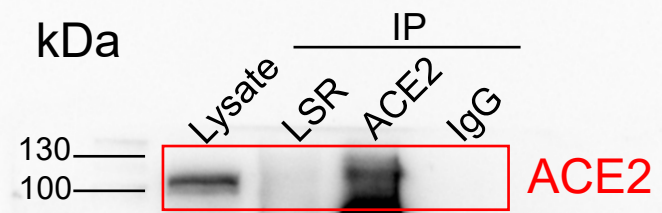

Supplement: Supplementary file 9 — Source data Fig. 5 [file 44318_2024_281_MOESM9_ESM.zip › Figure5/5K/5K.pdf]

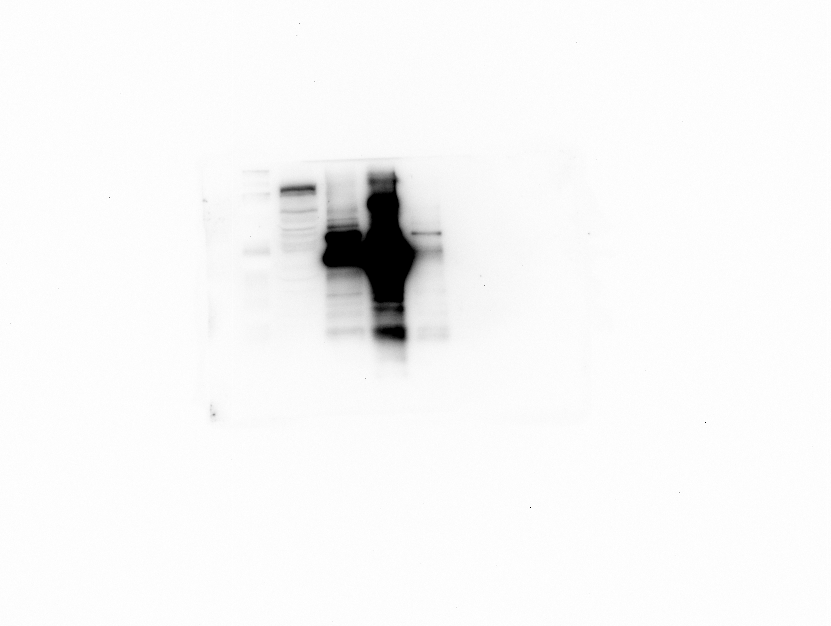

Supplement: Supplementary file 9 — Source data Fig. 5 [file 44318_2024_281_MOESM9_ESM.zip › Figure5/5K/western ACE2.png]

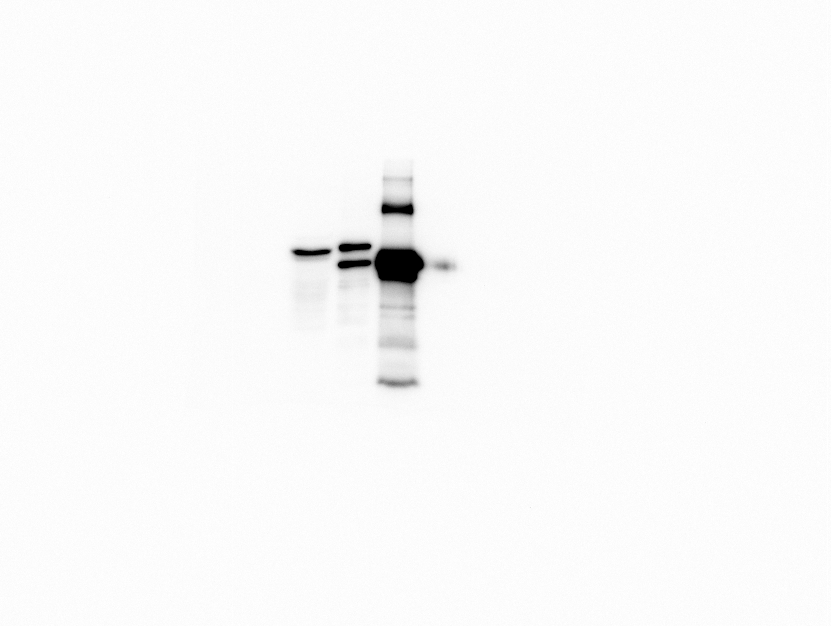

Supplement: Supplementary file 9 — Source data Fig. 5 [file 44318_2024_281_MOESM9_ESM.zip › Figure5/5K/western LSR.png]

kDa

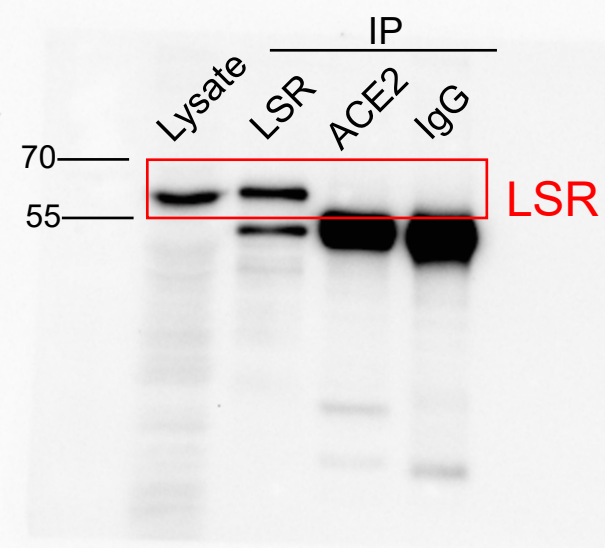

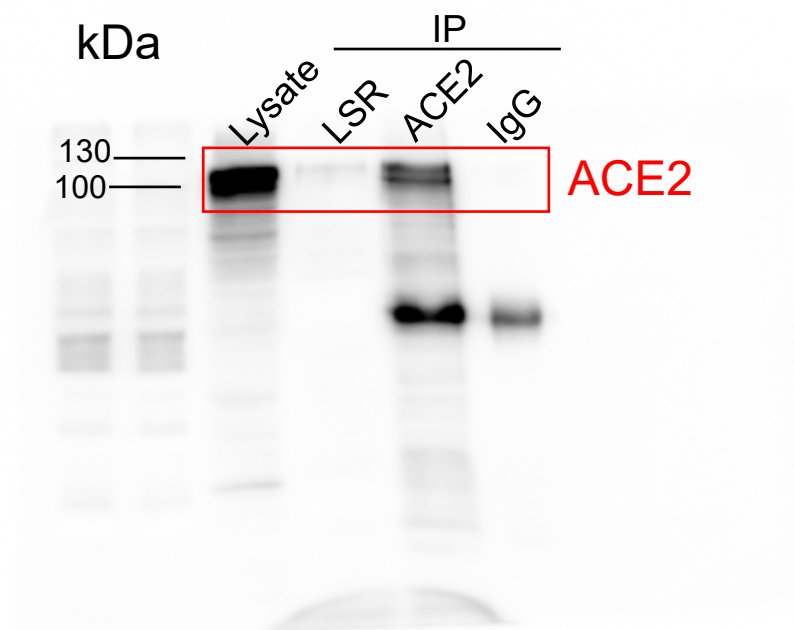

Supplement: Supplementary file 9 — Source data Fig. 5 [file 44318_2024_281_MOESM9_ESM.zip › Figure5/5L/5L.pdf]

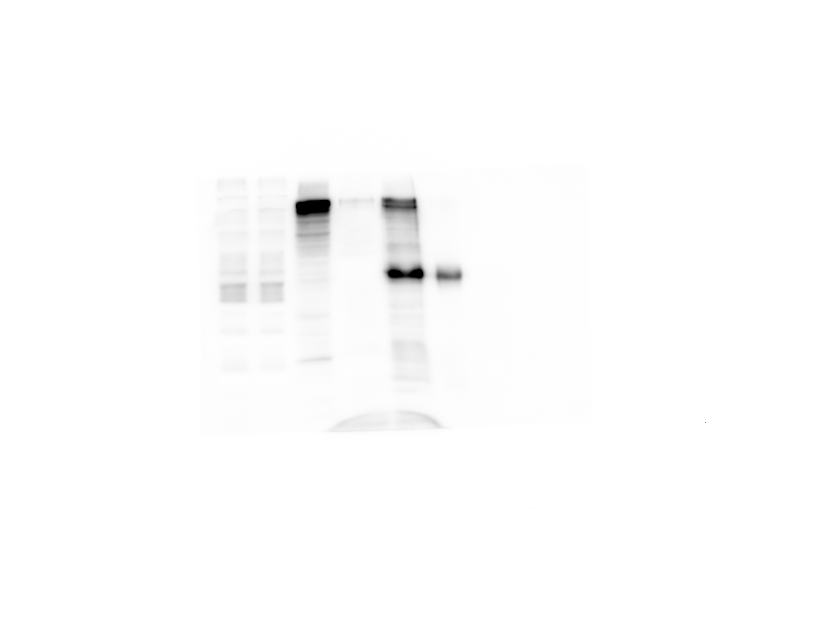

Supplement: Supplementary file 9 — Source data Fig. 5 [file 44318_2024_281_MOESM9_ESM.zip › Figure5/5L/western ACE2.png]

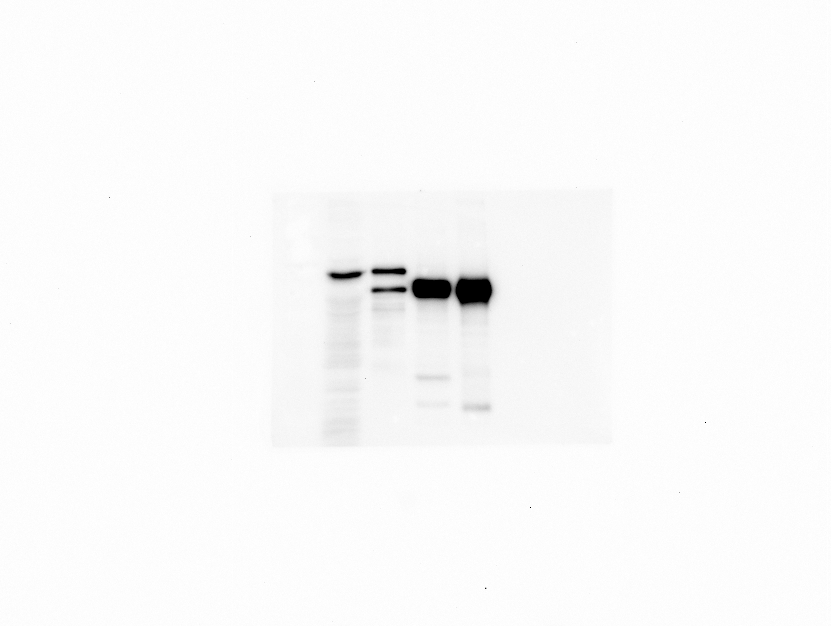

Supplement: Supplementary file 9 — Source data Fig. 5 [file 44318_2024_281_MOESM9_ESM.zip › Figure5/5L/western LSR.png]

kDa

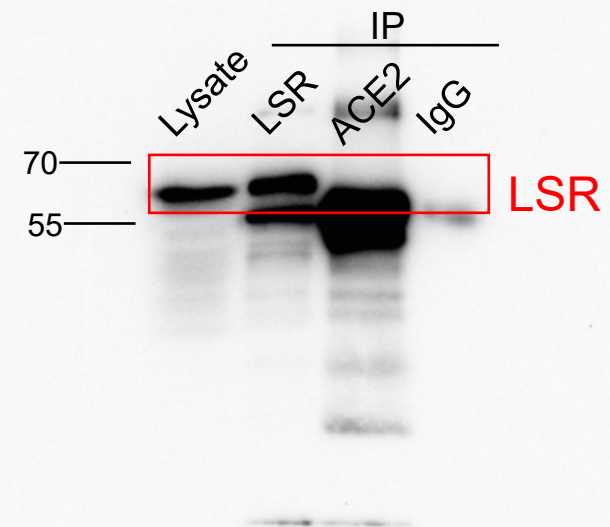

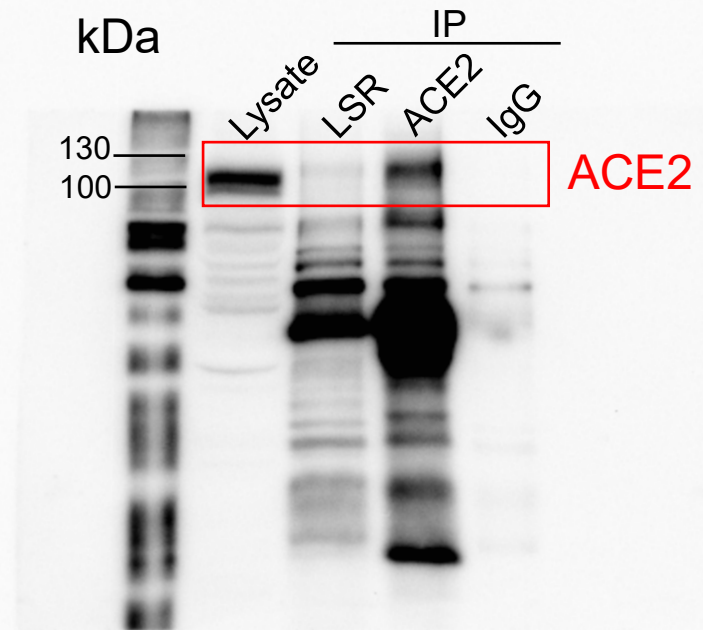

Supplement: Supplementary file 9 — Source data Fig. 5 [file 44318_2024_281_MOESM9_ESM.zip › Figure5/5M/5M.pdf]

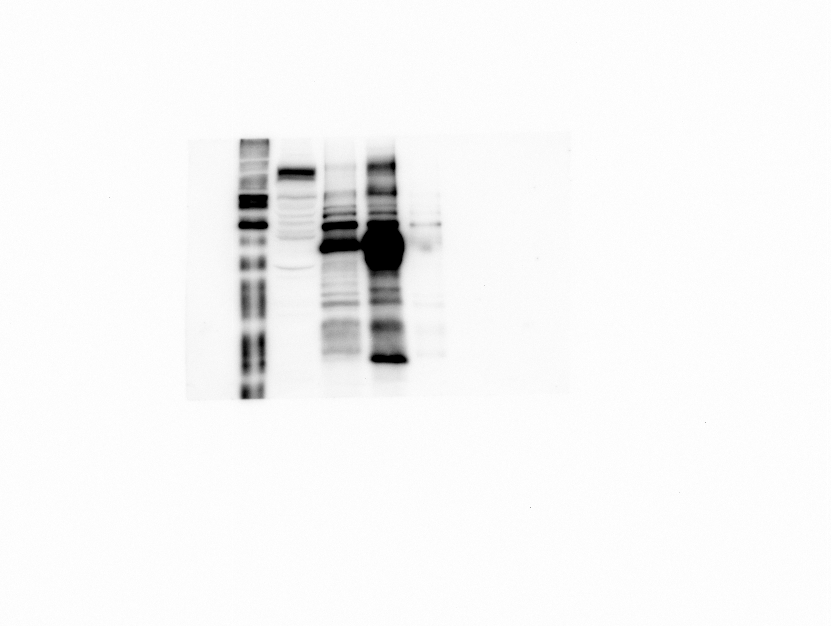

Supplement: Supplementary file 9 — Source data Fig. 5 [file 44318_2024_281_MOESM9_ESM.zip › Figure5/5M/western ACE2.png]

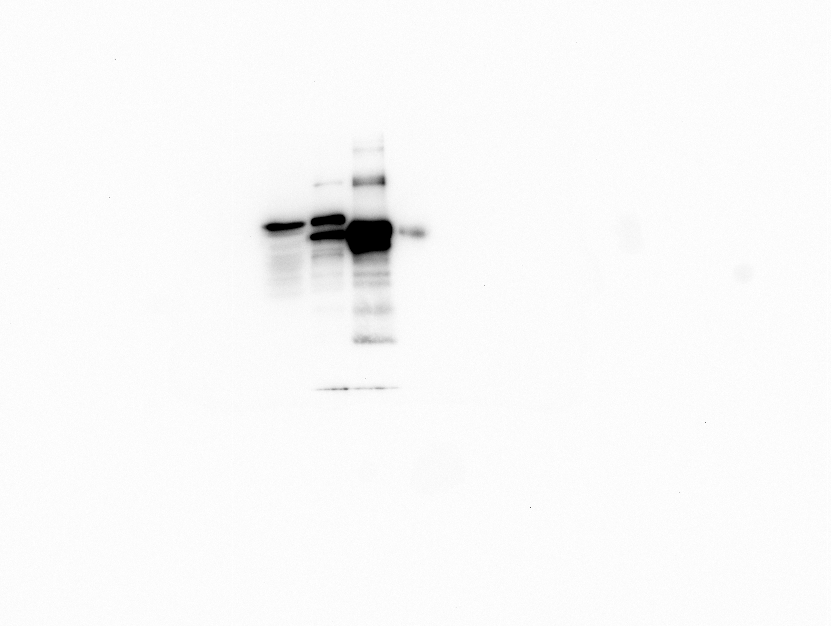

Supplement: Supplementary file 9 — Source data Fig. 5 [file 44318_2024_281_MOESM9_ESM.zip › Figure5/5M/western LSR.png]

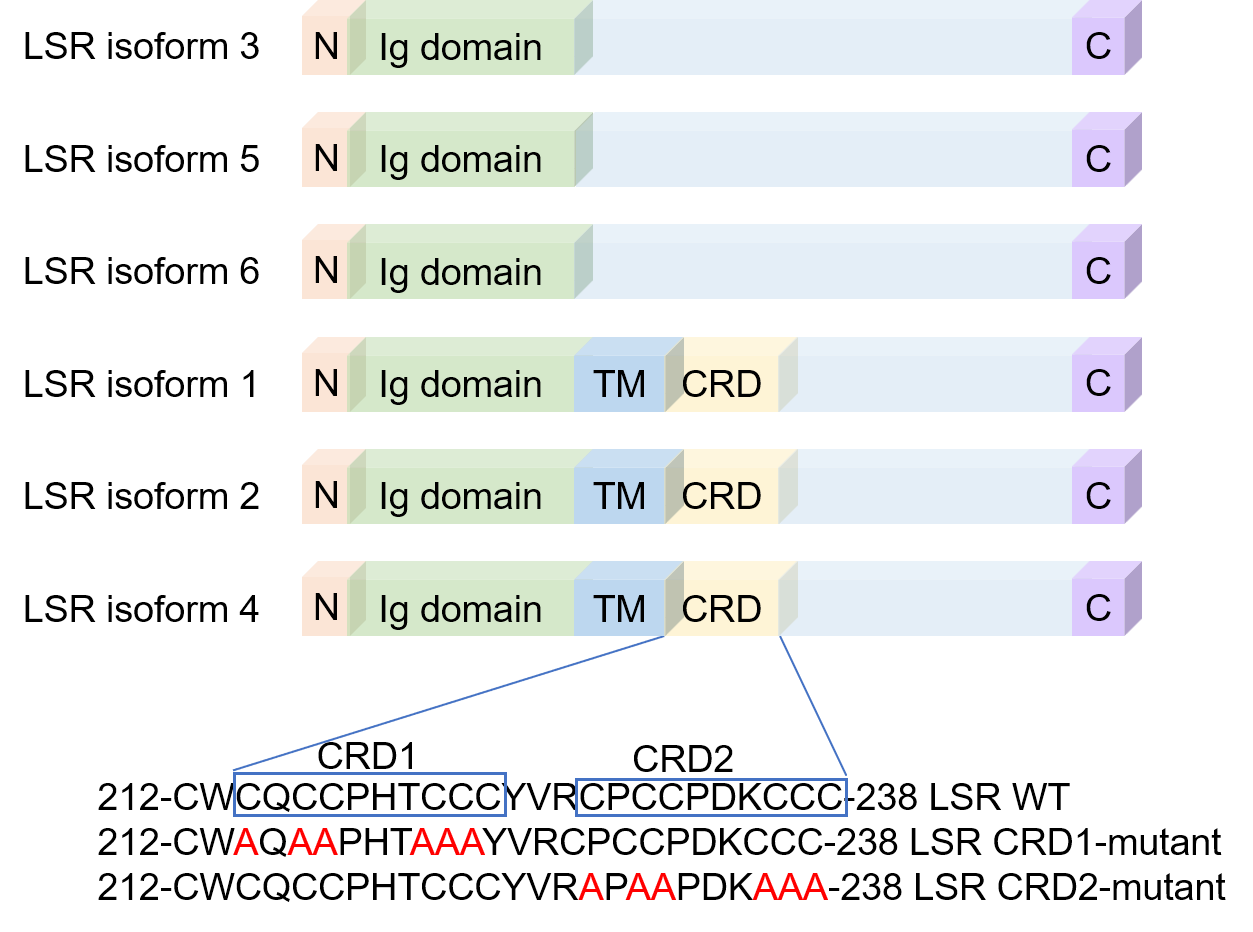

Supplement: Supplementary file 9 — Source data Fig. 5 [file 44318_2024_281_MOESM9_ESM.zip › Figure5/5N/Schematic diagram of different human LSR isoforms and CRD domain mutation.tif]

kDa

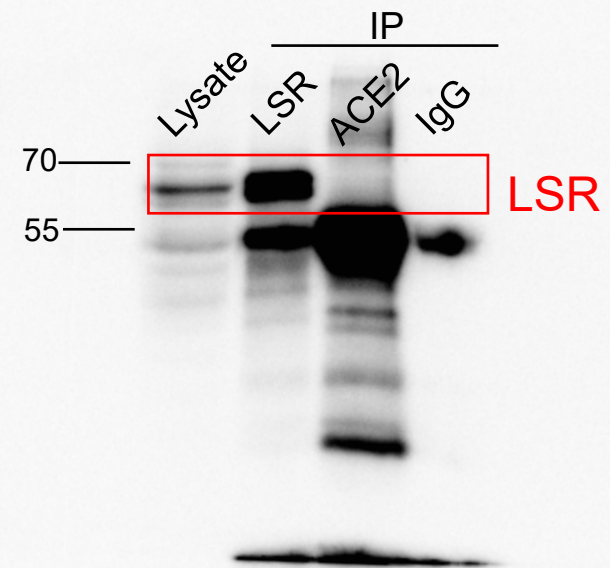

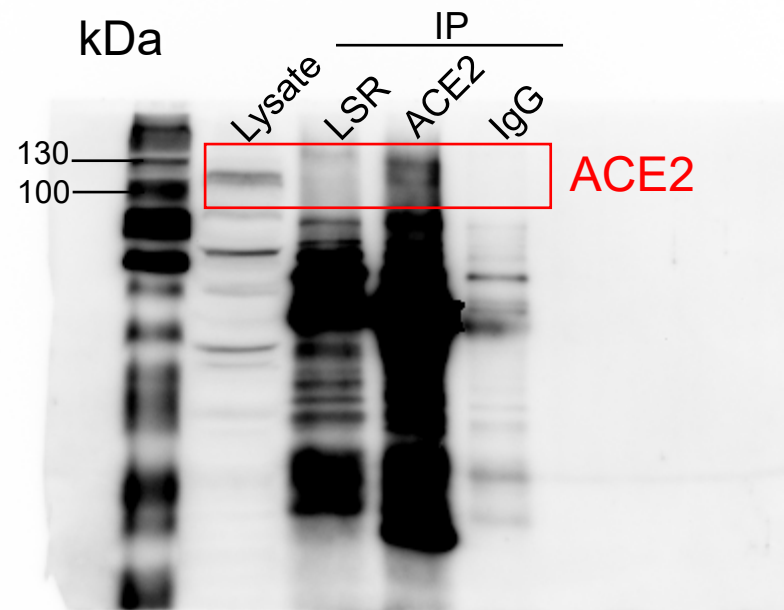

Supplement: Supplementary file 9 — Source data Fig. 5 [file 44318_2024_281_MOESM9_ESM.zip › Figure5/5O/5O.pdf]

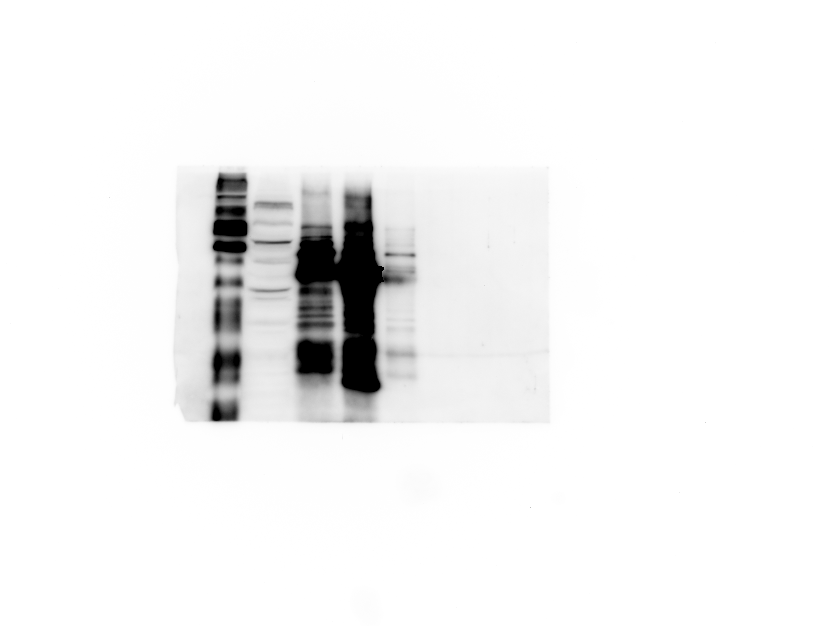

Supplement: Supplementary file 9 — Source data Fig. 5 [file 44318_2024_281_MOESM9_ESM.zip › Figure5/5O/western ACE2.png]

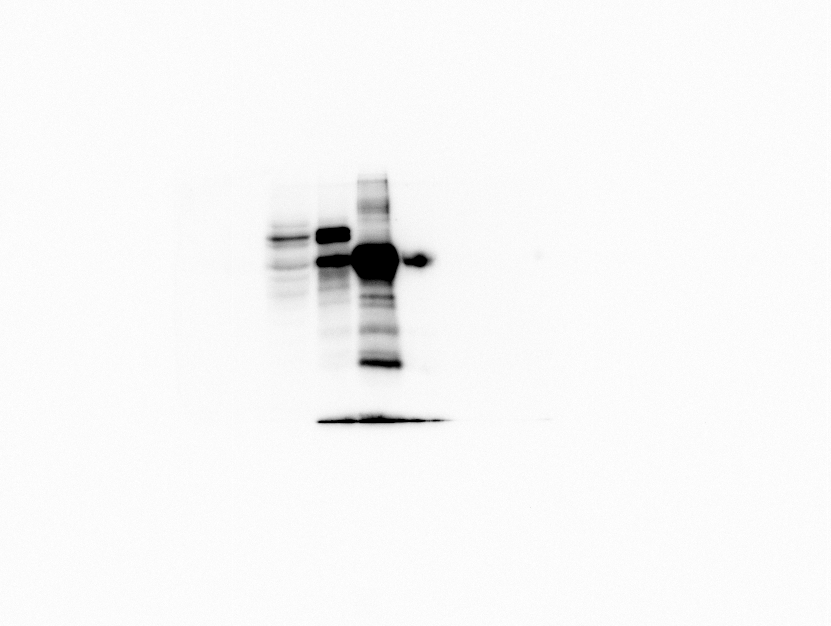

Supplement: Supplementary file 9 — Source data Fig. 5 [file 44318_2024_281_MOESM9_ESM.zip › Figure5/5O/western LSR.png]

kDa

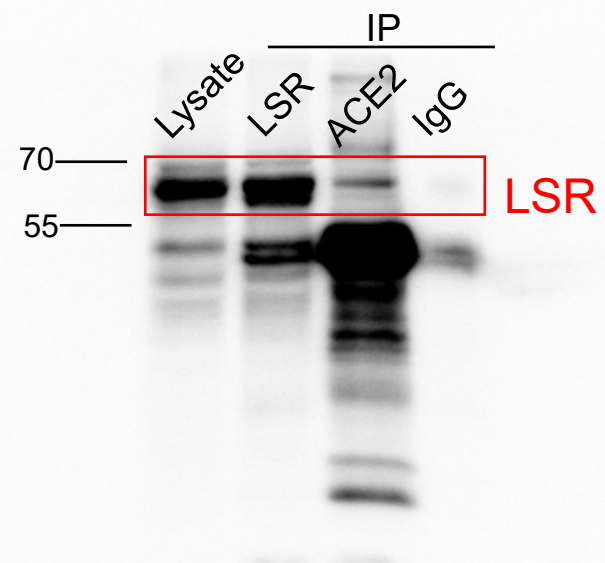

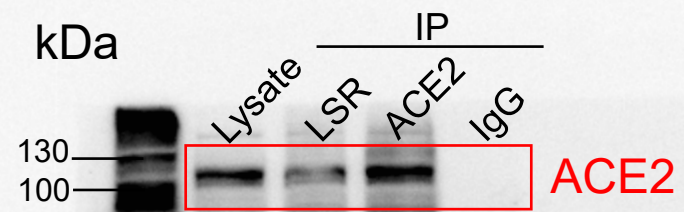

Supplement: Supplementary file 9 — Source data Fig. 5 [file 44318_2024_281_MOESM9_ESM.zip › Figure5/5P/5P.pdf]

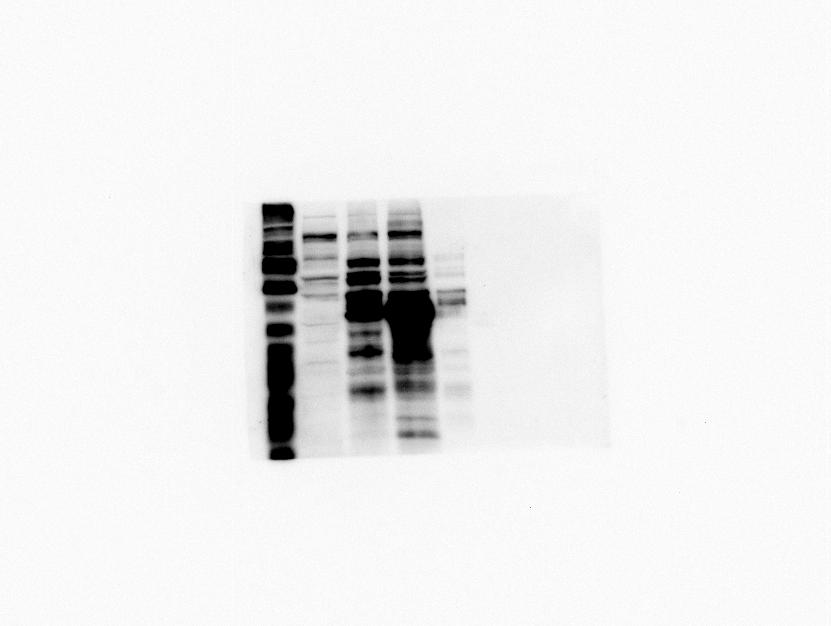

Supplement: Supplementary file 9 — Source data Fig. 5 [file 44318_2024_281_MOESM9_ESM.zip › Figure5/5P/western ACE2.png]

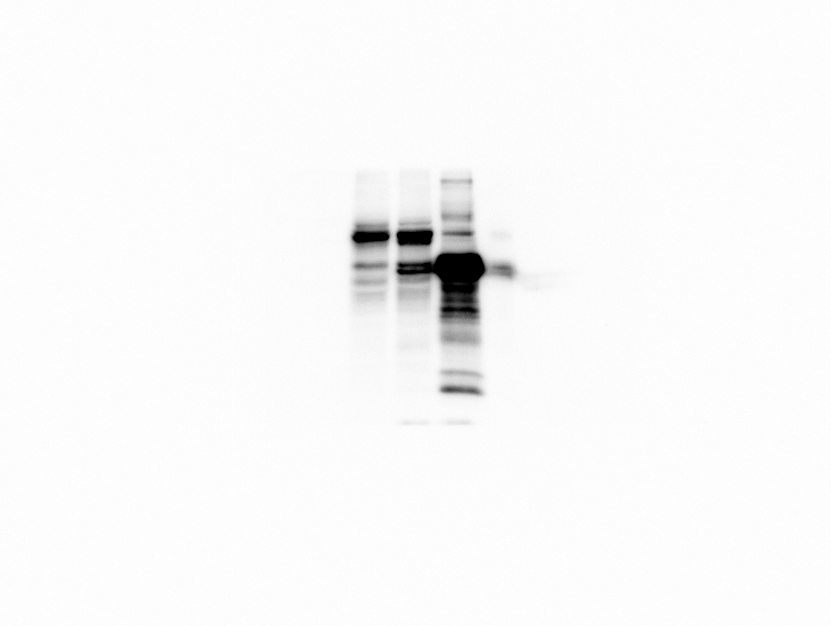

Supplement: Supplementary file 9 — Source data Fig. 5 [file 44318_2024_281_MOESM9_ESM.zip › Figure5/5P/western LSR.png]

kDa

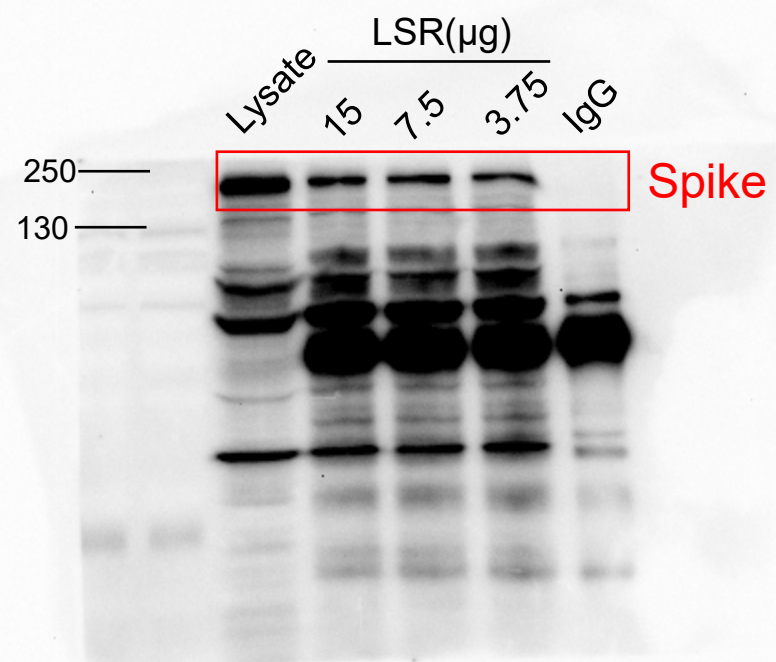

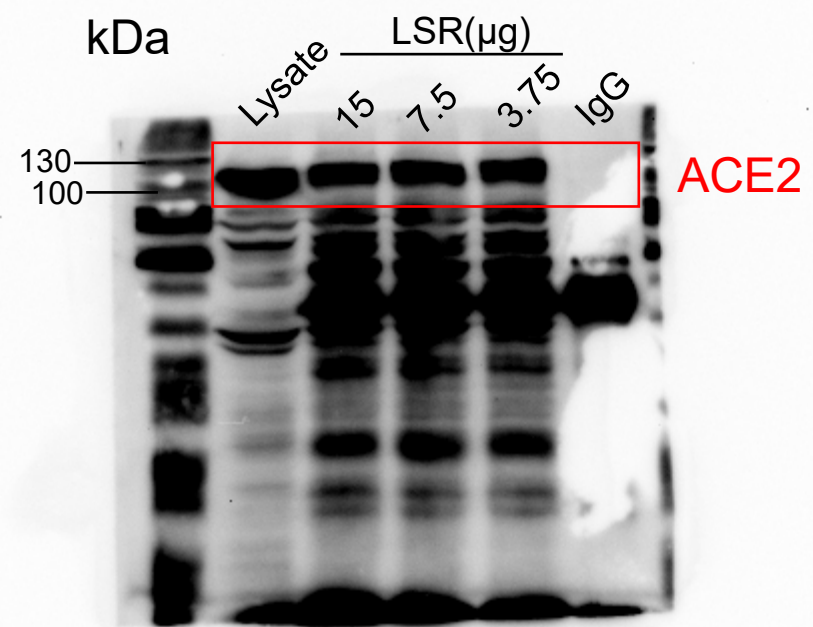

Supplement: Supplementary file 9 — Source data Fig. 5 [file 44318_2024_281_MOESM9_ESM.zip › Figure5/5Q/5Q.pdf]

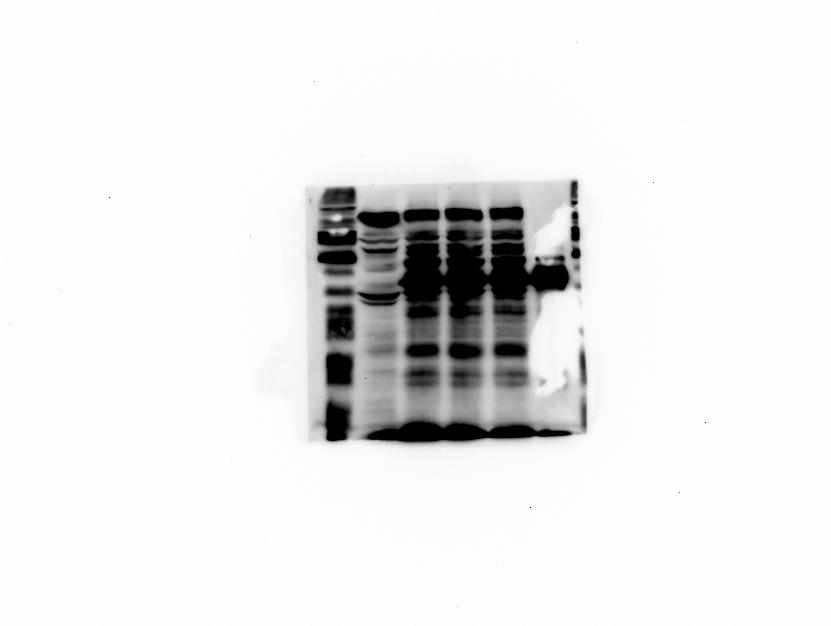

Supplement: Supplementary file 9 — Source data Fig. 5 [file 44318_2024_281_MOESM9_ESM.zip › Figure5/5Q/western ACE2.png]

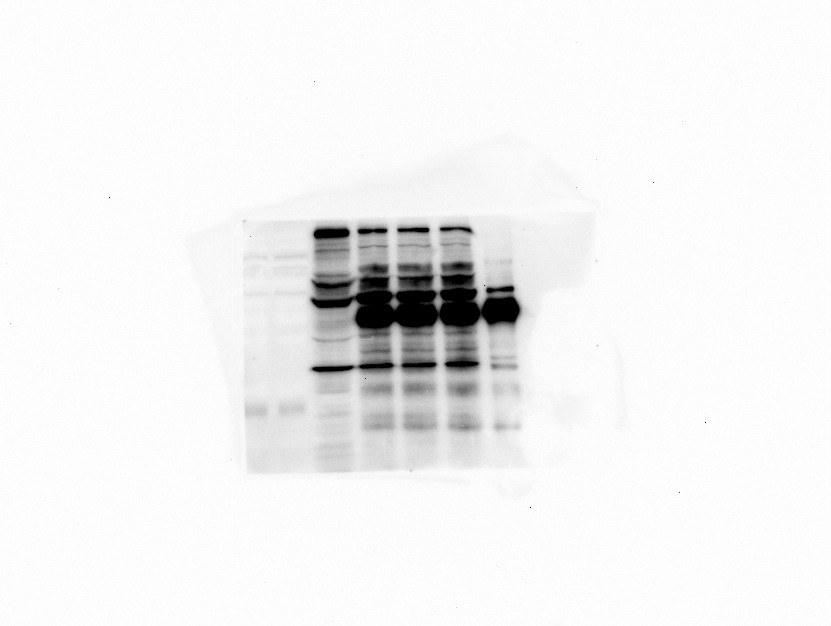

Supplement: Supplementary file 9 — Source data Fig. 5 [file 44318_2024_281_MOESM9_ESM.zip › Figure5/5Q/western spike.png]

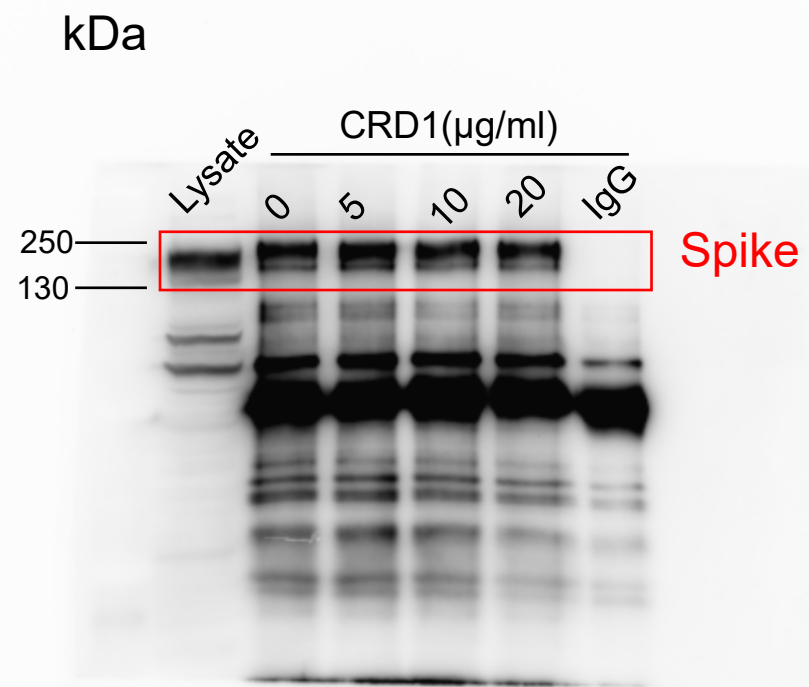

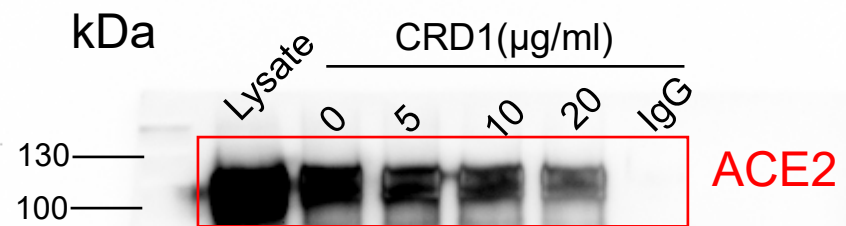

Supplement: Supplementary file 9 — Source data Fig. 5 [file 44318_2024_281_MOESM9_ESM.zip › Figure5/5R/5R.pdf]

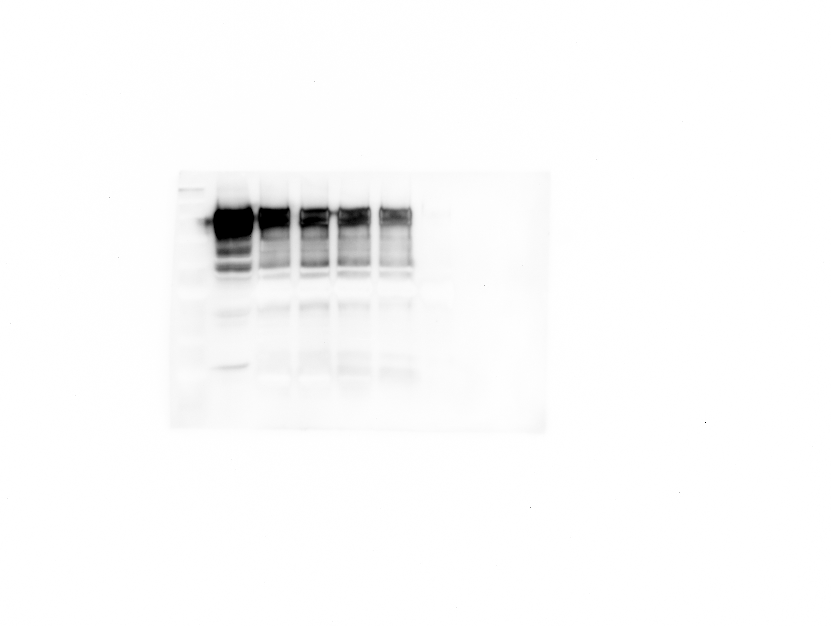

Supplement: Supplementary file 9 — Source data Fig. 5 [file 44318_2024_281_MOESM9_ESM.zip › Figure5/5R/western ACE2.png]

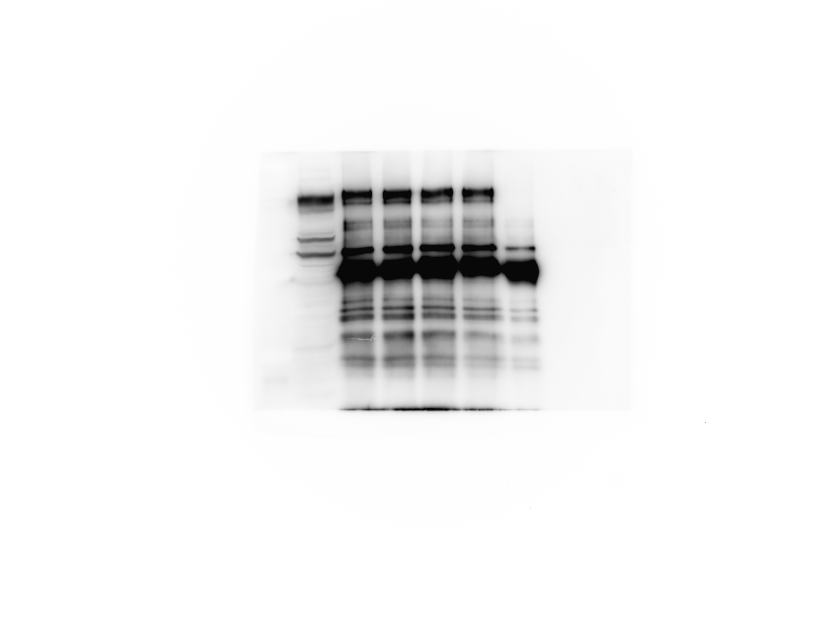

Supplement: Supplementary file 9 — Source data Fig. 5 [file 44318_2024_281_MOESM9_ESM.zip › Figure5/5R/western Spike.png]

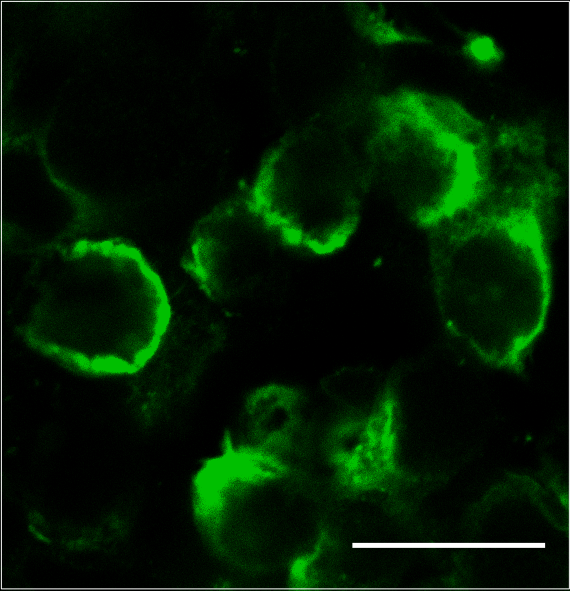

Supplement: Supplementary file 10 — Source data Fig. 6 [file 44318_2024_281_MOESM10_ESM.zip › Figure6/6A/IF LSR nonpermeabilized anti LSR-C.tif]

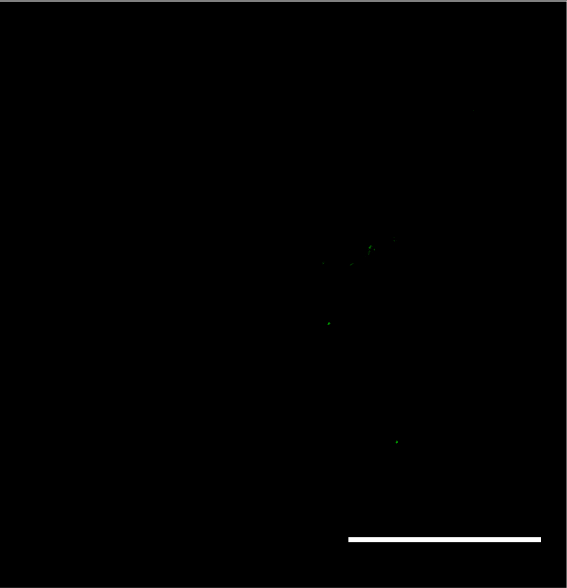

Supplement: Supplementary file 10 — Source data Fig. 6 [file 44318_2024_281_MOESM10_ESM.zip › Figure6/6A/IF LSR nonpermeabilized anti LSR-N.tif]

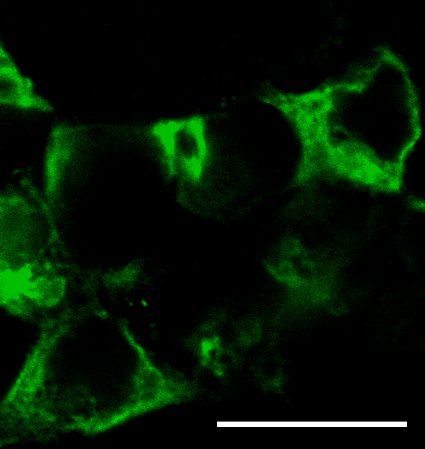

Supplement: Supplementary file 10 — Source data Fig. 6 [file 44318_2024_281_MOESM10_ESM.zip › Figure6/6A/IF LSR permeabilized anti LSR-C.tif]

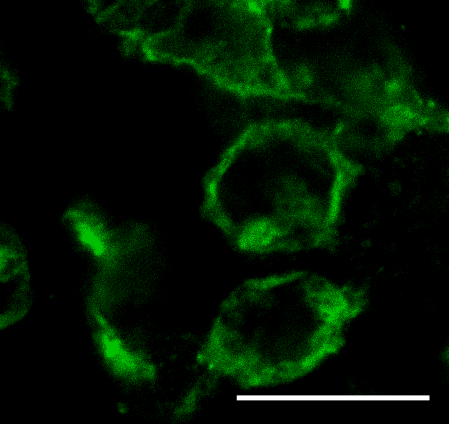

Supplement: Supplementary file 10 — Source data Fig. 6 [file 44318_2024_281_MOESM10_ESM.zip › Figure6/6A/IF LSR permeabilized anti LSR-N.tif]

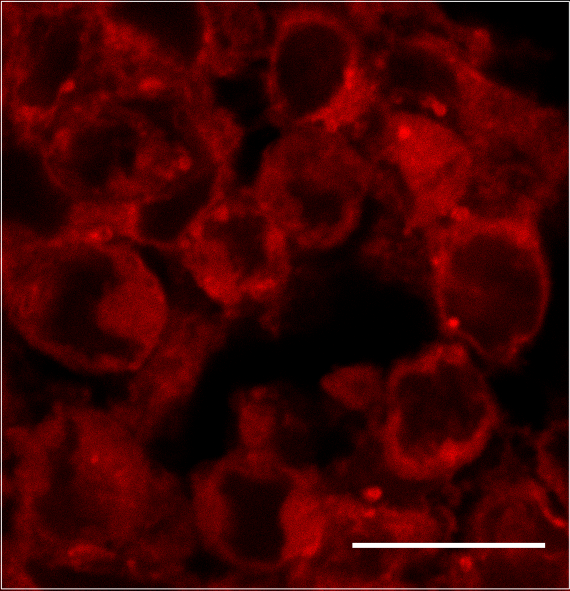

Supplement: Supplementary file 10 — Source data Fig. 6 [file 44318_2024_281_MOESM10_ESM.zip › Figure6/6A/IF cellmask nonpermeabilized anti LSR-C.tif]

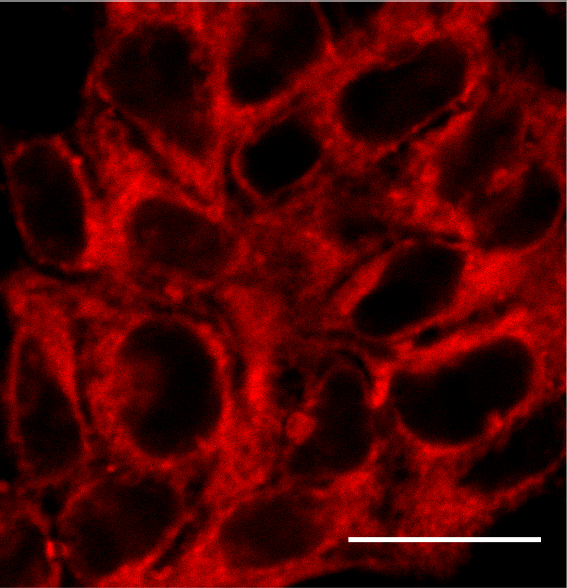

Supplement: Supplementary file 10 — Source data Fig. 6 [file 44318_2024_281_MOESM10_ESM.zip › Figure6/6A/IF cellmask nonpermeabilized anti LSR-N.tif]

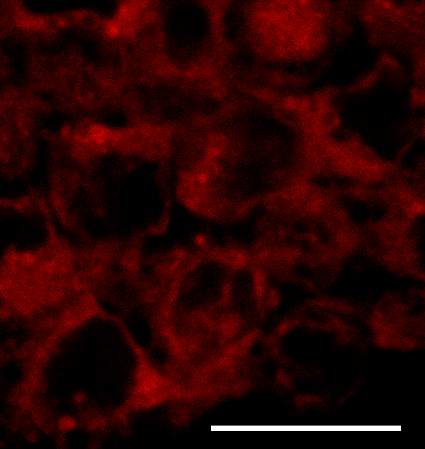

Supplement: Supplementary file 10 — Source data Fig. 6 [file 44318_2024_281_MOESM10_ESM.zip › Figure6/6A/IF cellmask permeabilized anti LSR-C.tif]

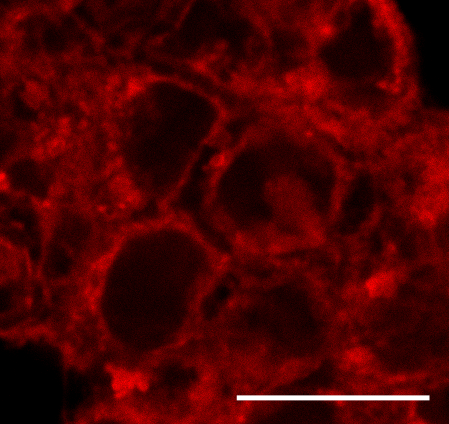

Supplement: Supplementary file 10 — Source data Fig. 6 [file 44318_2024_281_MOESM10_ESM.zip › Figure6/6A/IF cellmask permeabilized anti LSR-N.tif]

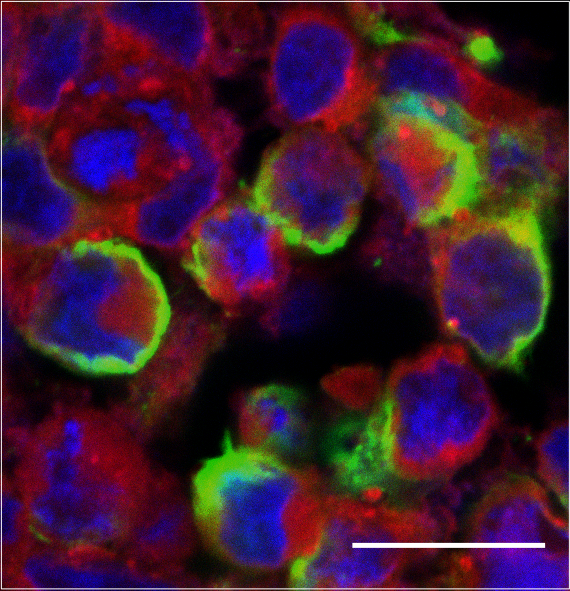

Supplement: Supplementary file 10 — Source data Fig. 6 [file 44318_2024_281_MOESM10_ESM.zip › Figure6/6A/IF merge nonpermeabilized anti LSR-C.tif]

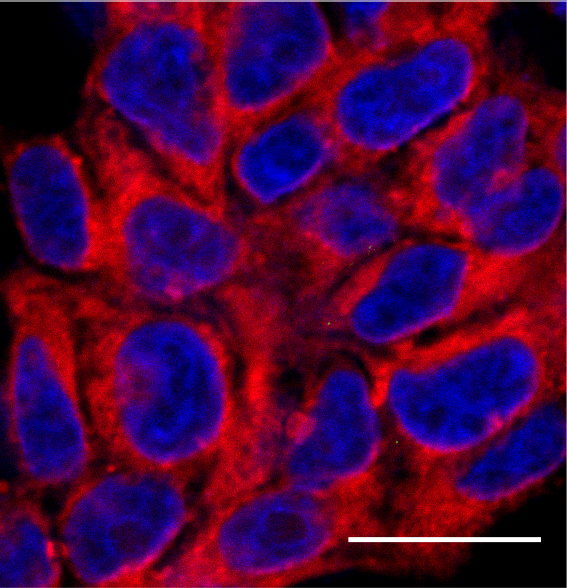

Supplement: Supplementary file 10 — Source data Fig. 6 [file 44318_2024_281_MOESM10_ESM.zip › Figure6/6A/IF merge nonpermeabilized anti LSR-N.tif]

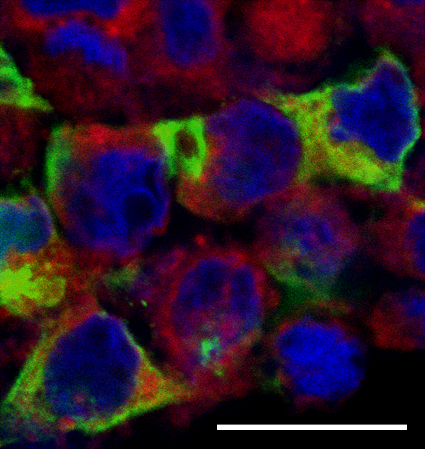

Supplement: Supplementary file 10 — Source data Fig. 6 [file 44318_2024_281_MOESM10_ESM.zip › Figure6/6A/IF merge permeabilized anti LSR-C.tif]

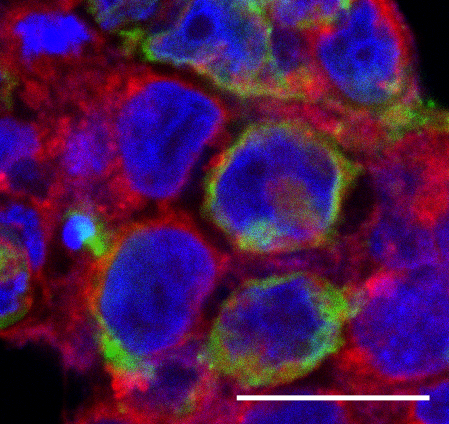

Supplement: Supplementary file 10 — Source data Fig. 6 [file 44318_2024_281_MOESM10_ESM.zip › Figure6/6A/IF merge permeabilized anti LSR-N.tif]

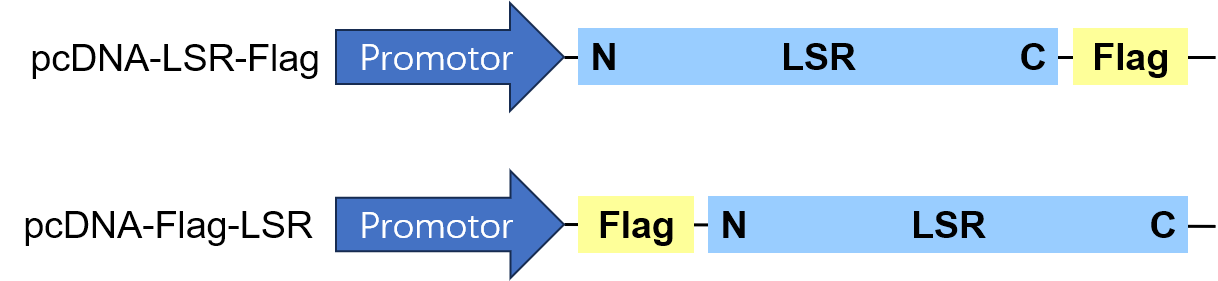

Supplement: Supplementary file 10 — Source data Fig. 6 [file 44318_2024_281_MOESM10_ESM.zip › Figure6/6B/Schematic diagram of the plasmids expressing human LSR with a flag tag in the C-terminal or N-terminal.tif]

kDa

CTL

N-Flag

C-Flag

55

45

35

25

15

Flag

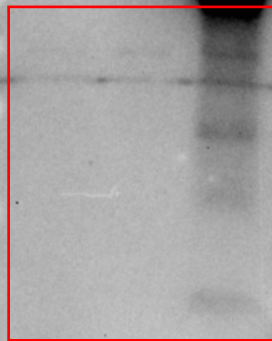

Supplement: Supplementary file 10 — Source data Fig. 6 [file 44318_2024_281_MOESM10_ESM.zip › Figure6/6C/6C.pdf]

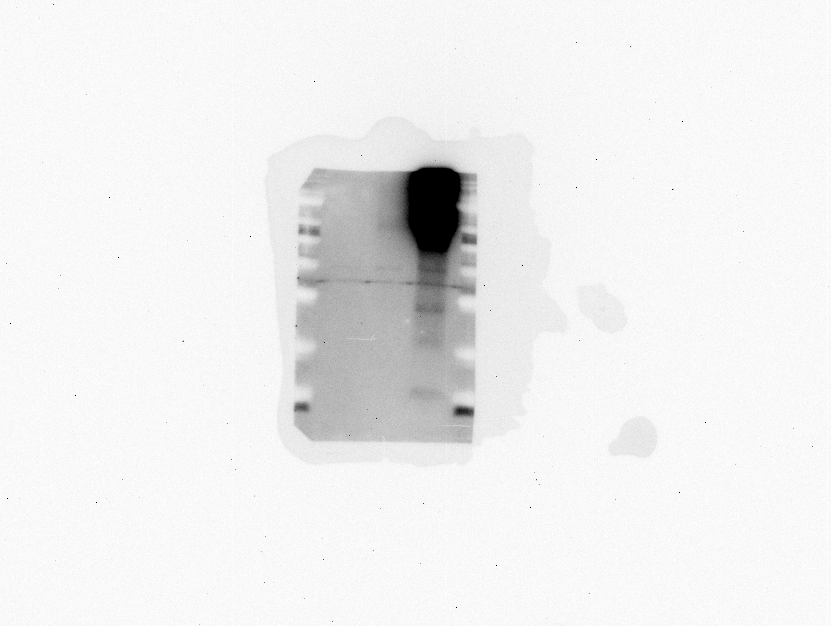

Supplement: Supplementary file 10 — Source data Fig. 6 [file 44318_2024_281_MOESM10_ESM.zip › Figure6/6C/western Flag.png]

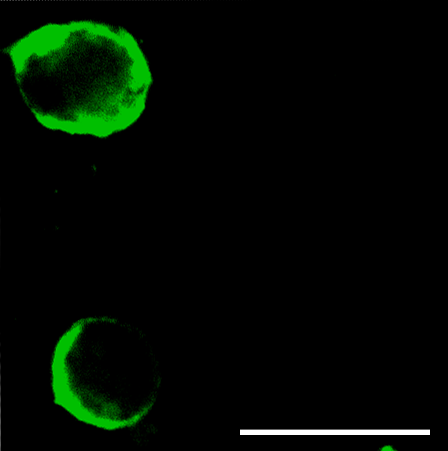

Supplement: Supplementary file 10 — Source data Fig. 6 [file 44318_2024_281_MOESM10_ESM.zip › Figure6/6D/IF Flag C-Flag.tif]

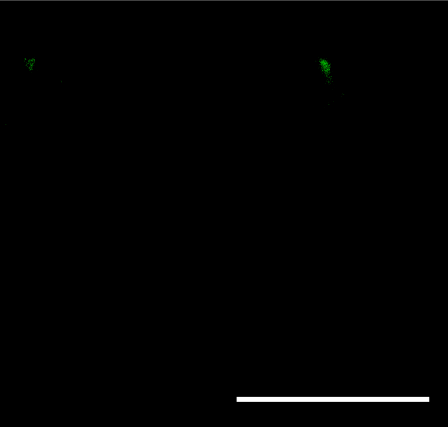

Supplement: Supplementary file 10 — Source data Fig. 6 [file 44318_2024_281_MOESM10_ESM.zip › Figure6/6D/IF Flag N-Flag.tif]

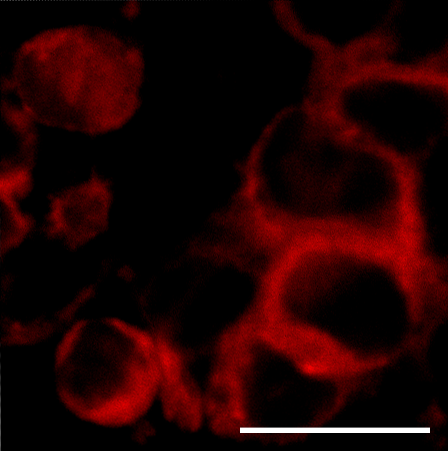

Supplement: Supplementary file 10 — Source data Fig. 6 [file 44318_2024_281_MOESM10_ESM.zip › Figure6/6D/IF cellmask C-Flag.tif]

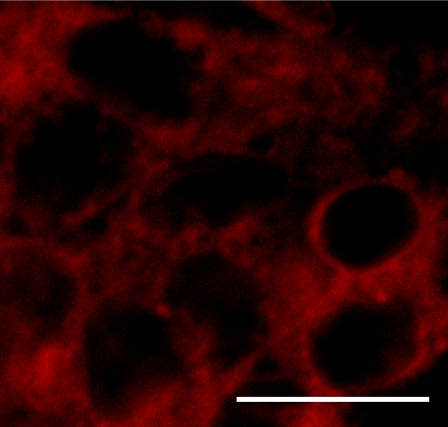

Supplement: Supplementary file 10 — Source data Fig. 6 [file 44318_2024_281_MOESM10_ESM.zip › Figure6/6D/IF cellmask N-Flag.tif]

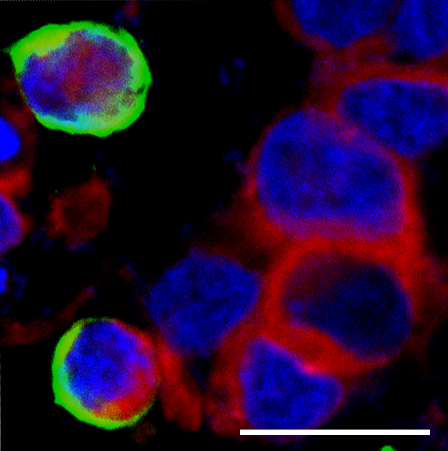

Supplement: Supplementary file 10 — Source data Fig. 6 [file 44318_2024_281_MOESM10_ESM.zip › Figure6/6D/IF merge C-Flag.tif]

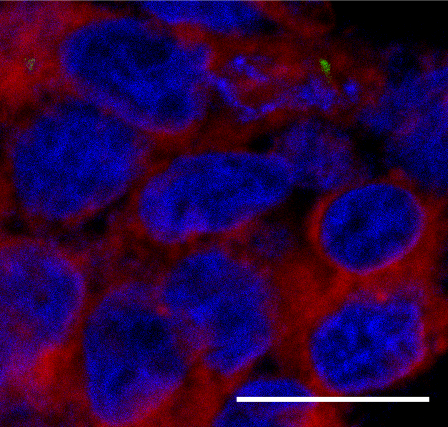

Supplement: Supplementary file 10 — Source data Fig. 6 [file 44318_2024_281_MOESM10_ESM.zip › Figure6/6D/IF merge N-Flag.tif]

kDa

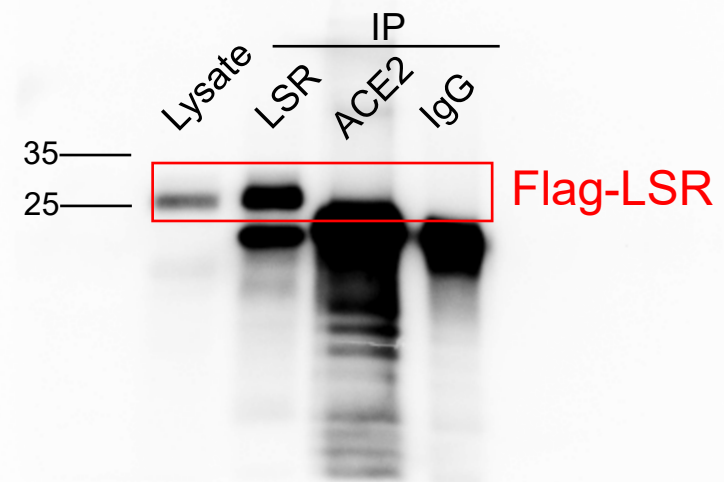

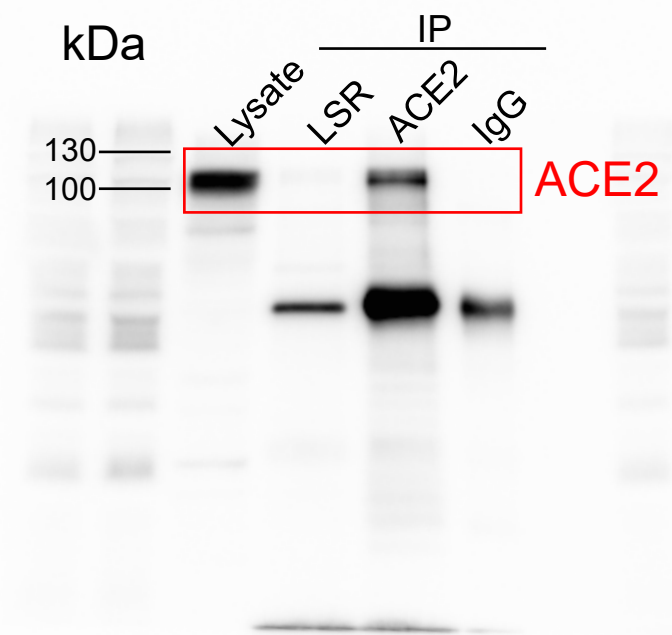

Supplement: Supplementary file 10 — Source data Fig. 6 [file 44318_2024_281_MOESM10_ESM.zip › Figure6/6E/6E.pdf]

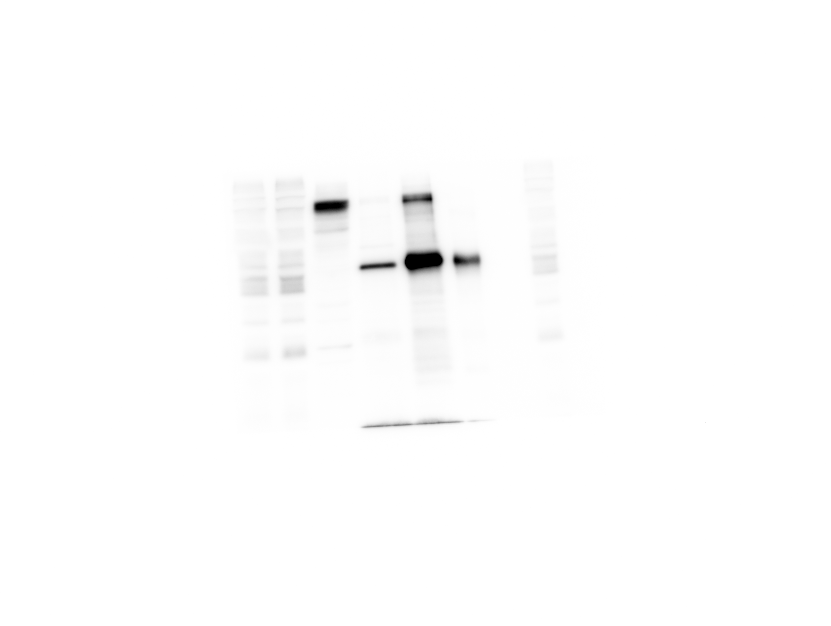

Supplement: Supplementary file 10 — Source data Fig. 6 [file 44318_2024_281_MOESM10_ESM.zip › Figure6/6E/western ACE2.png]

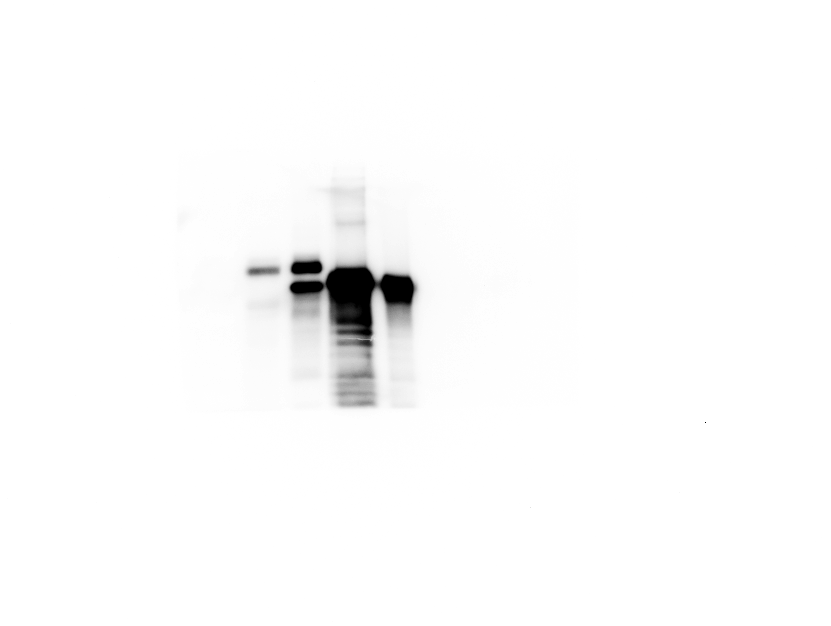

Supplement: Supplementary file 10 — Source data Fig. 6 [file 44318_2024_281_MOESM10_ESM.zip › Figure6/6E/western Flag.png]

kDa

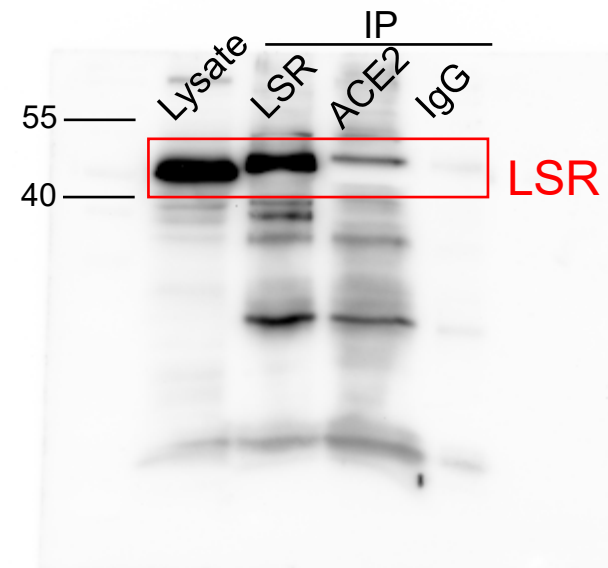

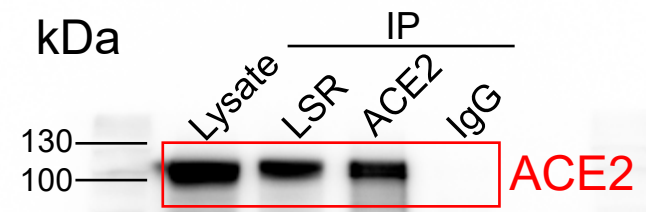

Supplement: Supplementary file 10 — Source data Fig. 6 [file 44318_2024_281_MOESM10_ESM.zip › Figure6/6F/6F.pdf]
