# Supplementary figures and images for: Tight junction protein LSR is a host defense factor against SARS-CoV-2 infection in the small intestine (part 3 of 4)
Source: EMBO J. 2024 Oct 23;43(23):6124–51. doi: 10.1038/s44318-024-00281-4 (PMC11612383; doi:10.1038/s44318-024-00281-4)

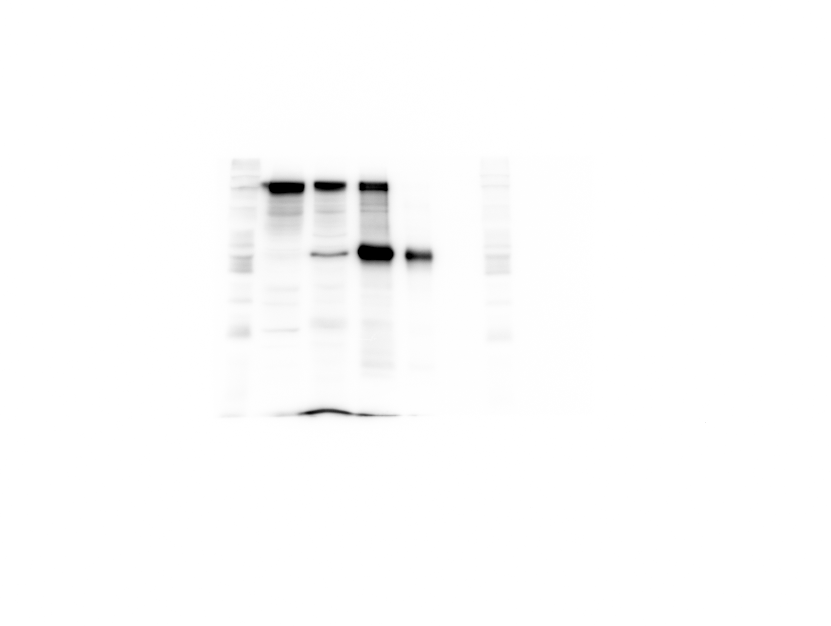

Supplement: Supplementary file 10 — Source data Fig. 6 [file 44318_2024_281_MOESM10_ESM.zip › Figure6/6F/western ACE2.png]

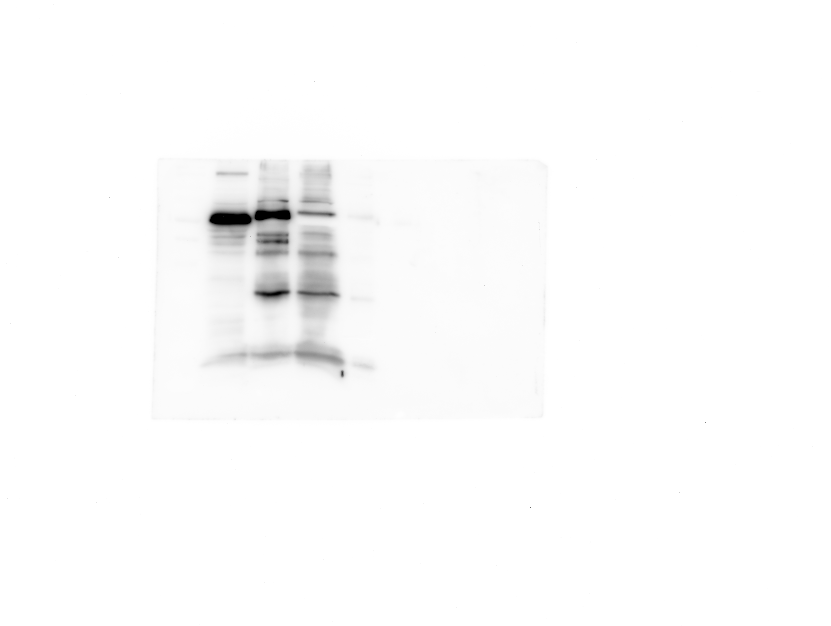

Supplement: Supplementary file 10 — Source data Fig. 6 [file 44318_2024_281_MOESM10_ESM.zip › Figure6/6F/western Flag.png]

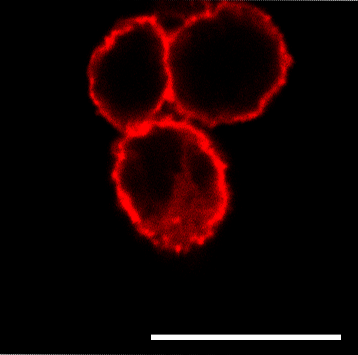

Supplement: Supplementary file 11 — Source data Fig. 7 [file 44318_2024_281_MOESM11_ESM.zip › Figure7/7G/IF ACE2.tif]

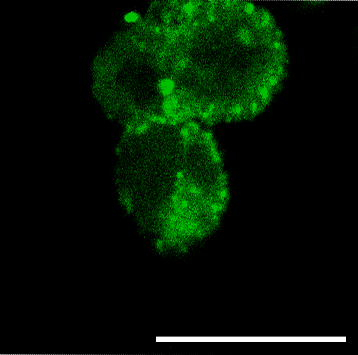

Supplement: Supplementary file 11 — Source data Fig. 7 [file 44318_2024_281_MOESM11_ESM.zip › Figure7/7G/IF CRD1.tif]

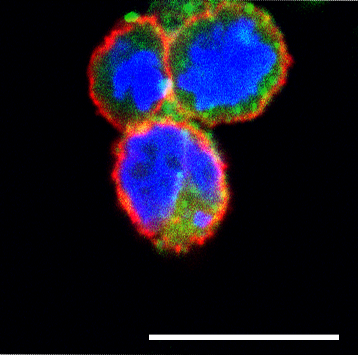

Supplement: Supplementary file 11 — Source data Fig. 7 [file 44318_2024_281_MOESM11_ESM.zip › Figure7/7G/IF merge.tif]

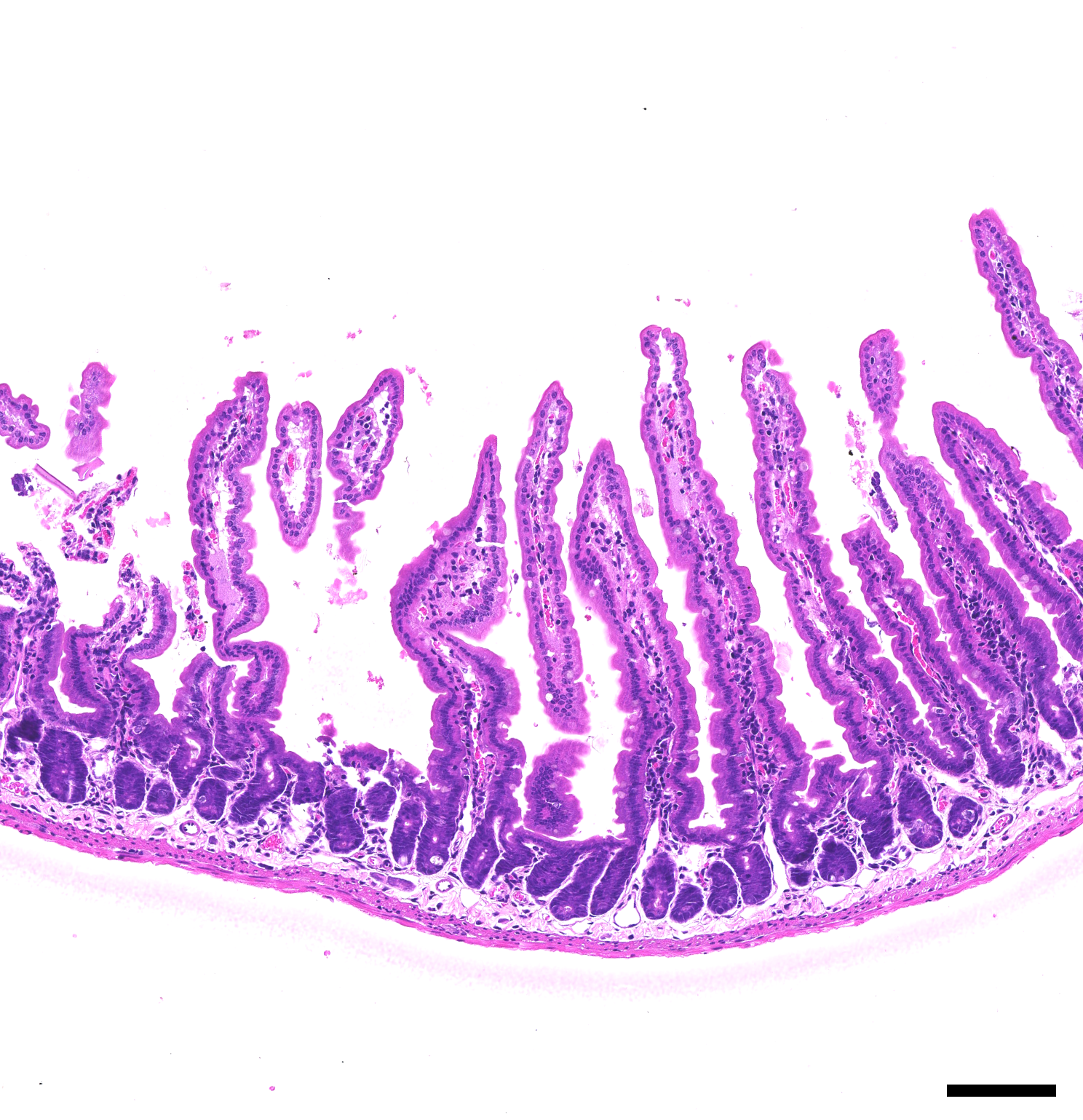

Supplement: Supplementary file 11 — Source data Fig. 7 [file 44318_2024_281_MOESM11_ESM.zip › Figure7/7H/HE VSV-SARS-CoV-2 Duodeum.tif]

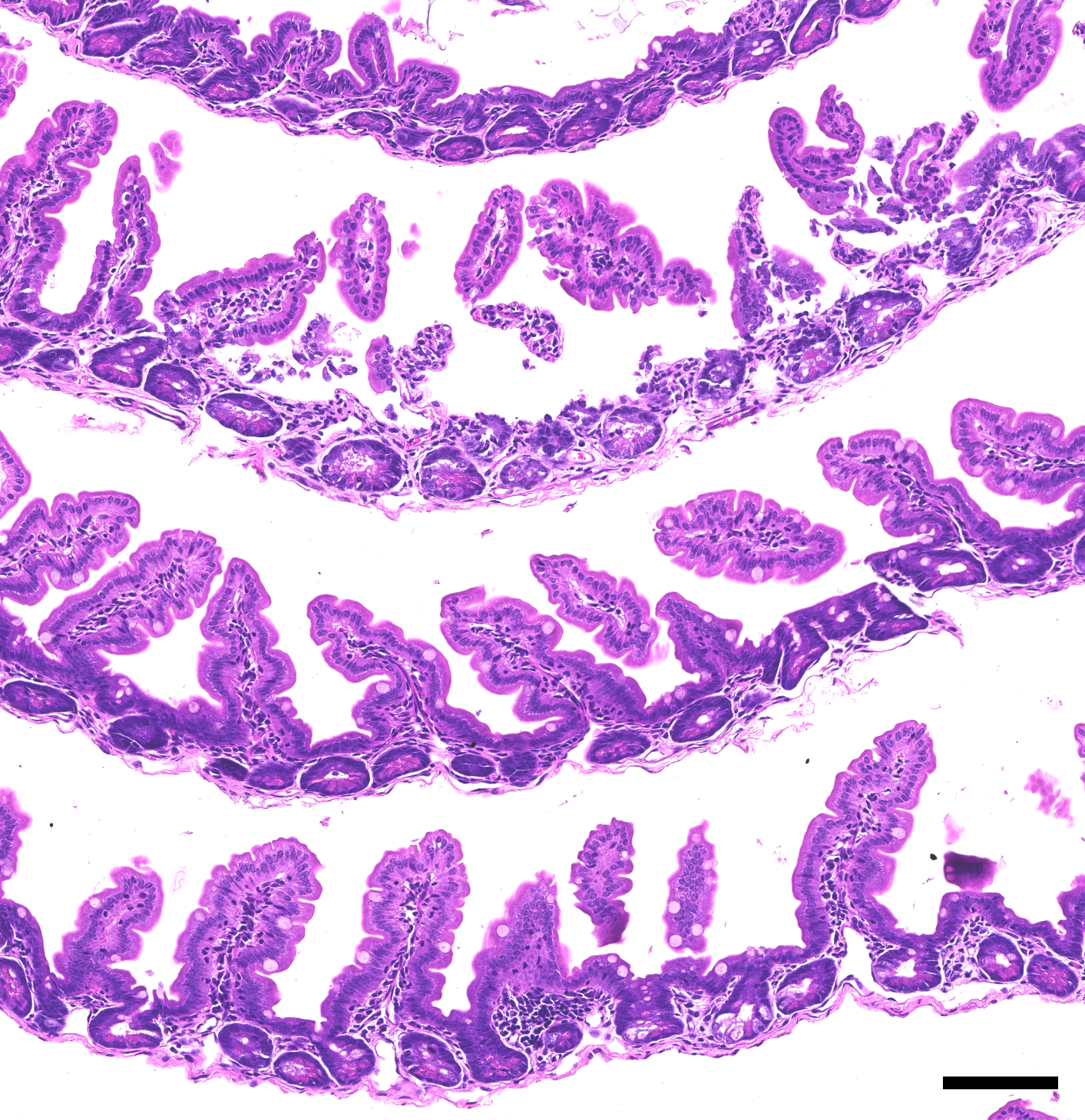

Supplement: Supplementary file 11 — Source data Fig. 7 [file 44318_2024_281_MOESM11_ESM.zip › Figure7/7H/HE VSV-SARS-CoV-2 Ileum.tif]

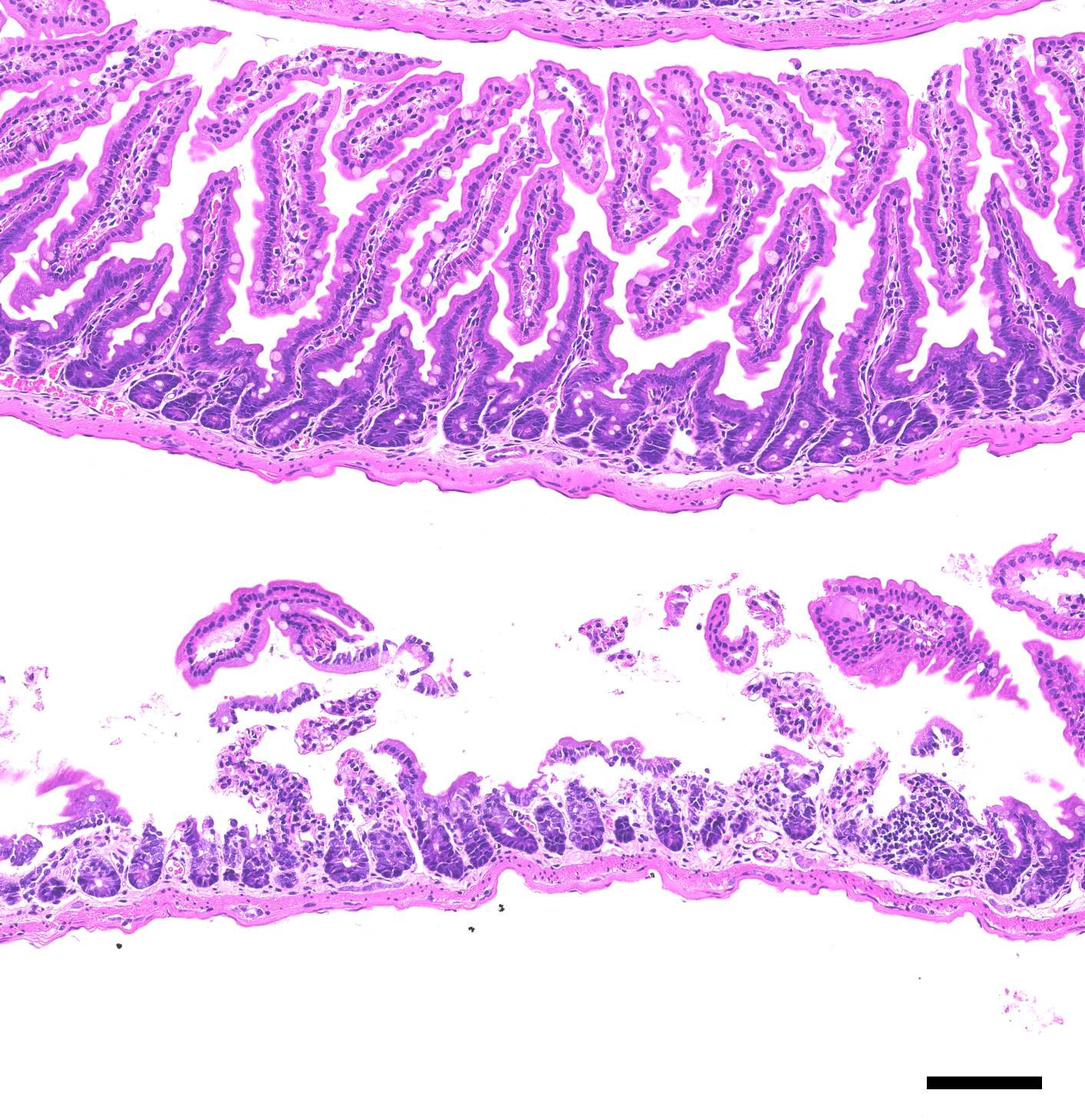

Supplement: Supplementary file 11 — Source data Fig. 7 [file 44318_2024_281_MOESM11_ESM.zip › Figure7/7H/HE VSV-SARS-CoV-2 Jejunum.tif]

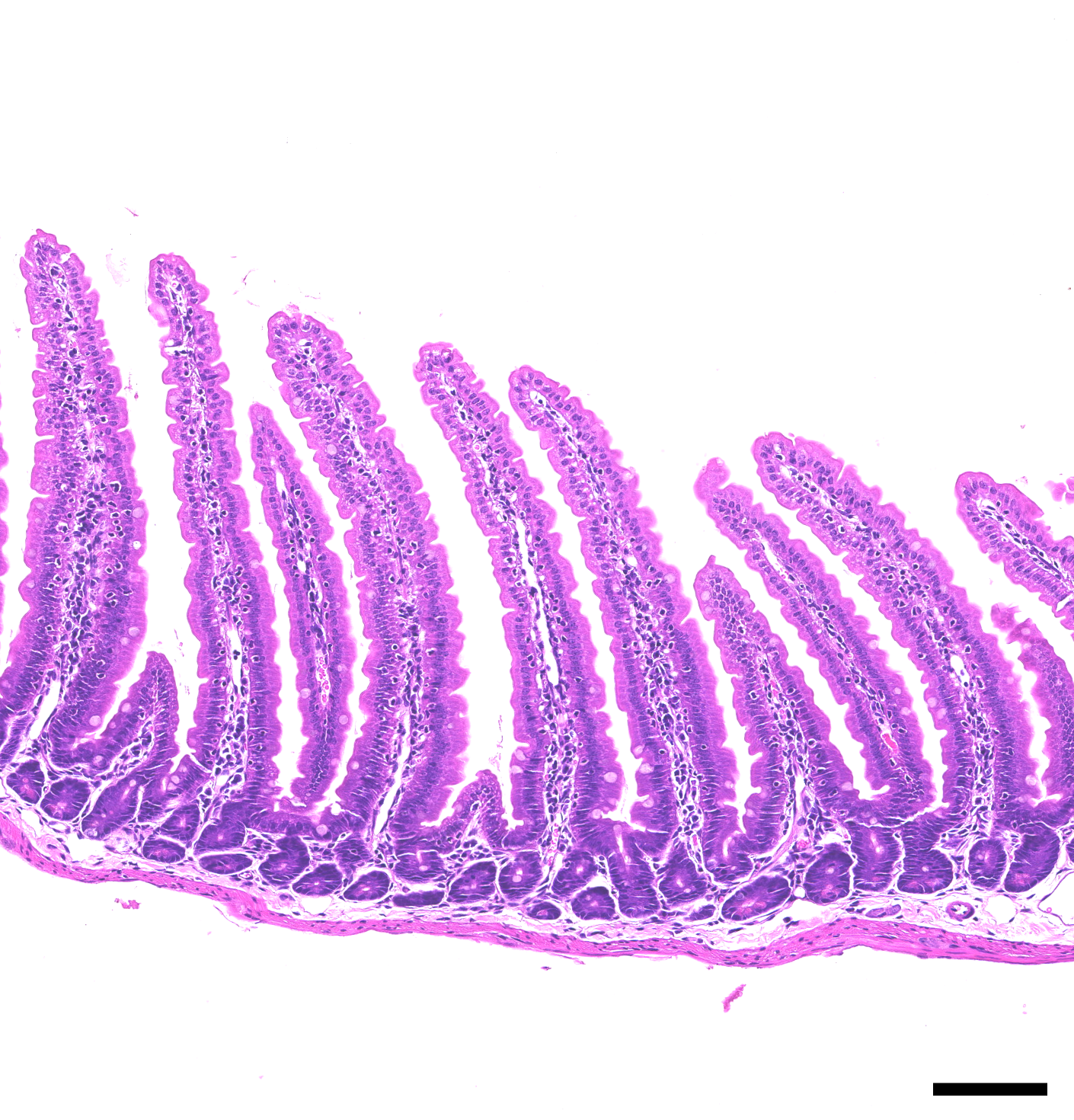

Supplement: Supplementary file 11 — Source data Fig. 7 [file 44318_2024_281_MOESM11_ESM.zip › Figure7/7H/HE VSV-SARS-CoV-2+CRD1 Duodeum.tif]

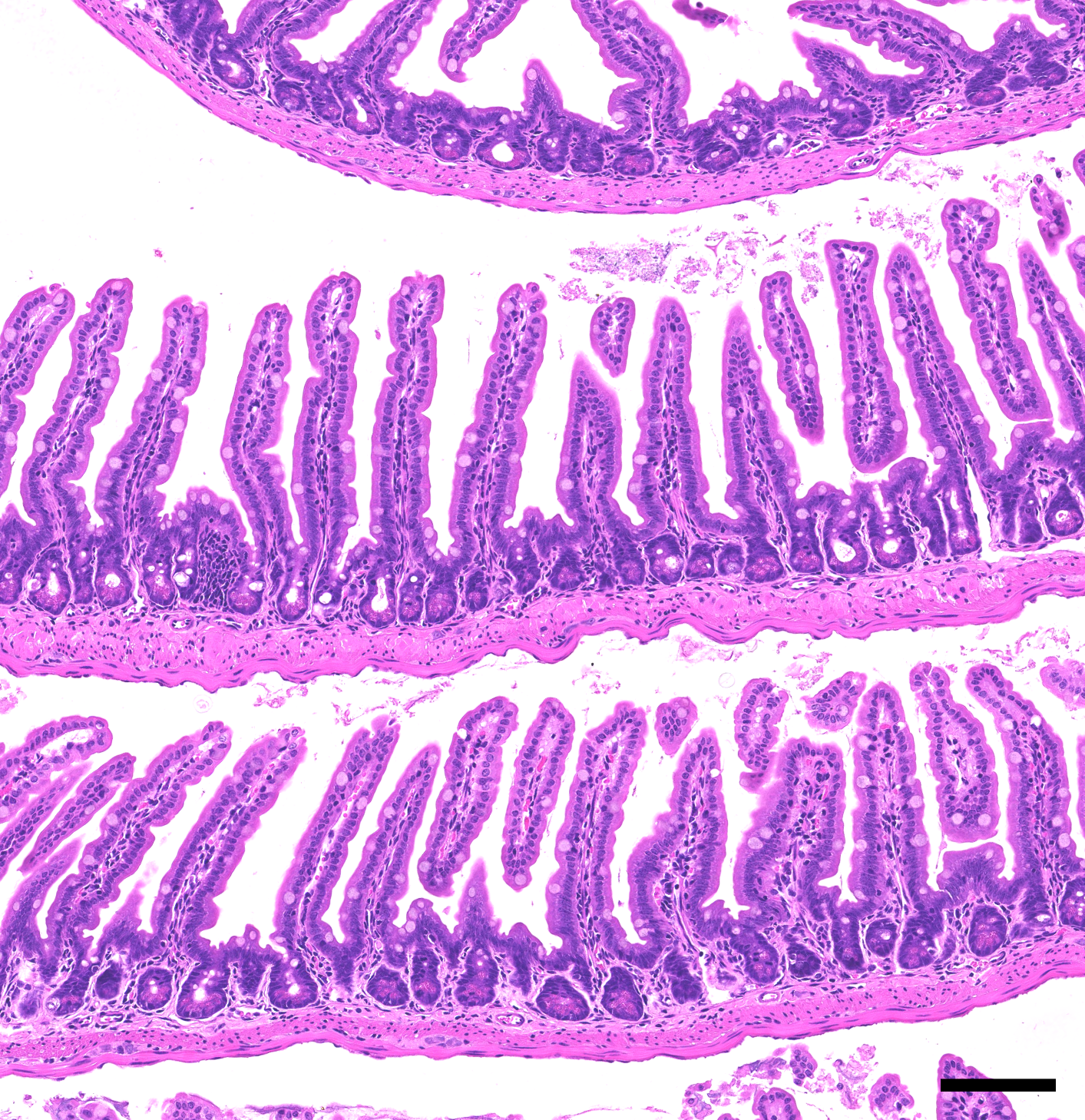

Supplement: Supplementary file 11 — Source data Fig. 7 [file 44318_2024_281_MOESM11_ESM.zip › Figure7/7H/HE VSV-SARS-CoV-2+CRD1 Ileum.tif]

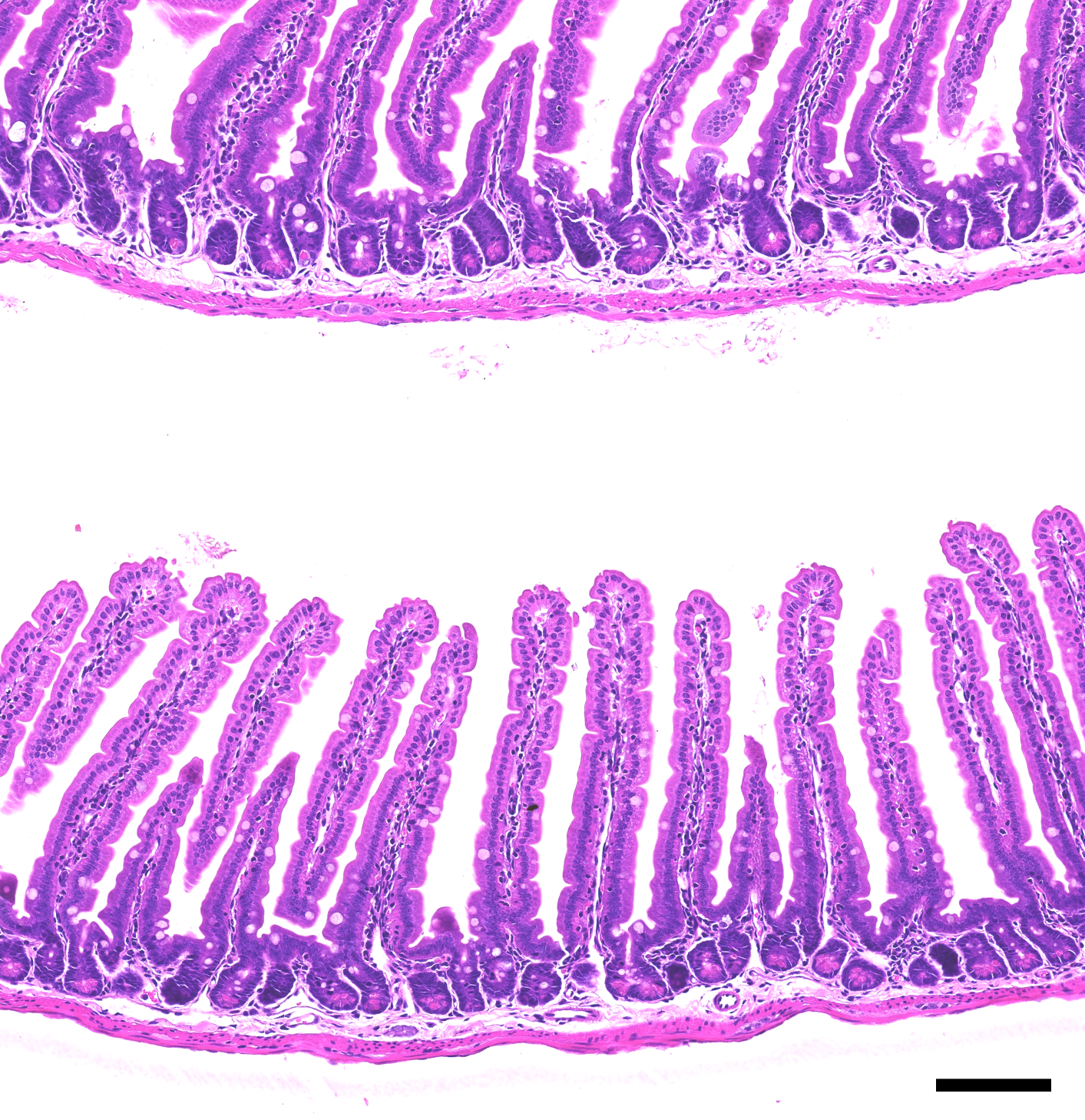

Supplement: Supplementary file 11 — Source data Fig. 7 [file 44318_2024_281_MOESM11_ESM.zip › Figure7/7H/HE VSV-SARS-CoV-2+CRD1 Jejunum.tif]

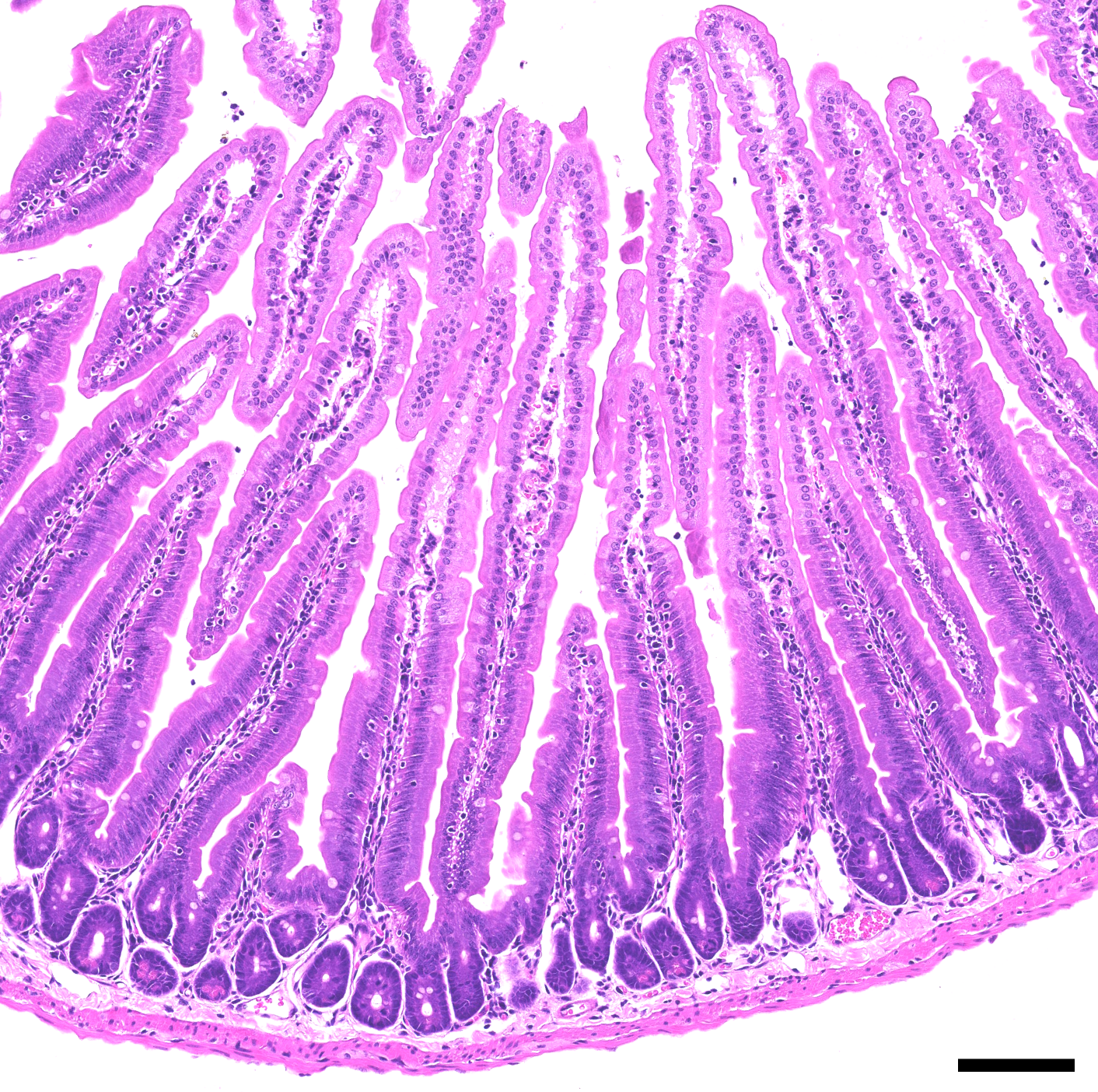

Supplement: Supplementary file 11 — Source data Fig. 7 [file 44318_2024_281_MOESM11_ESM.zip › Figure7/7H/HE control Duodeum.tif]

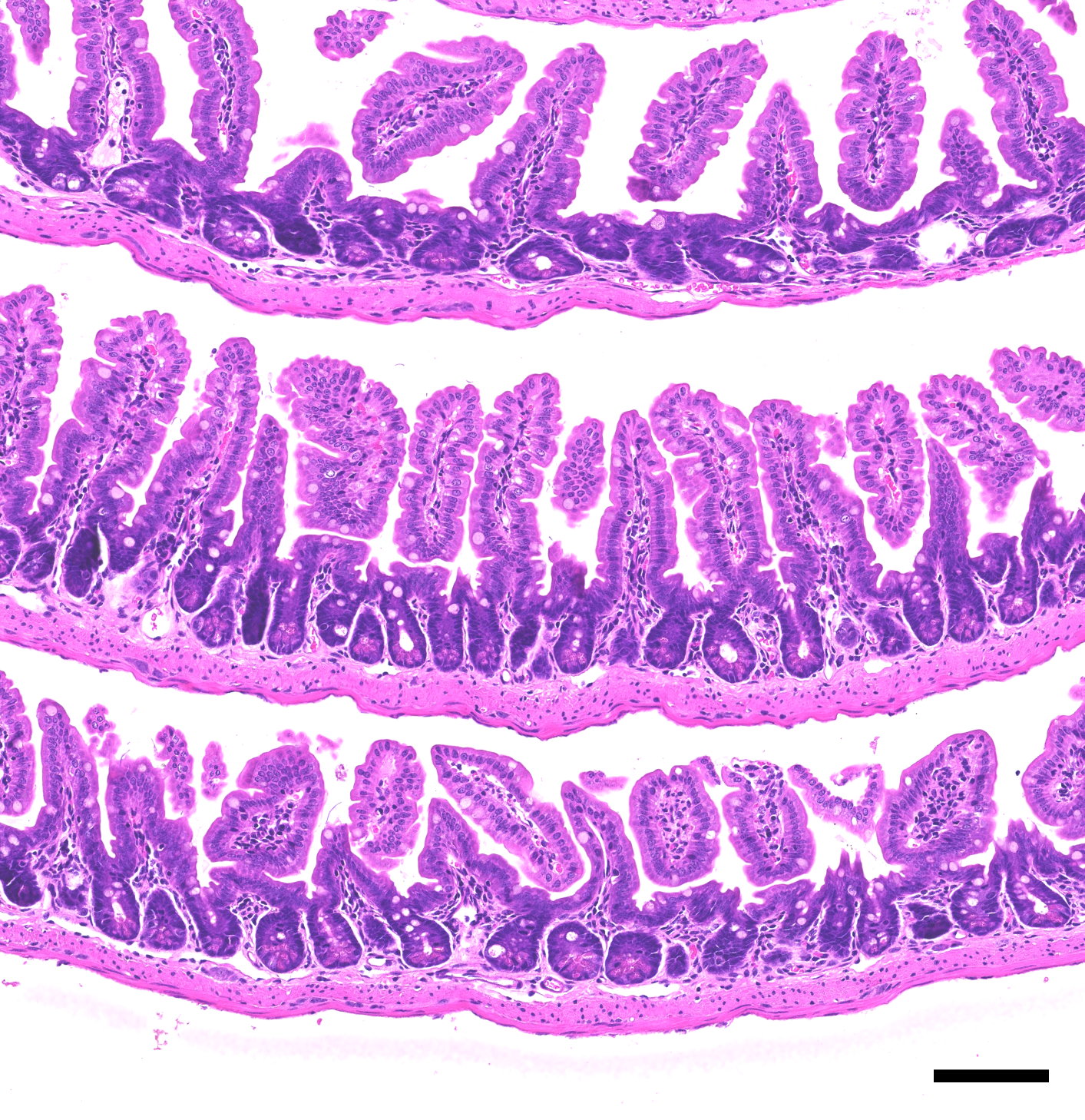

Supplement: Supplementary file 11 — Source data Fig. 7 [file 44318_2024_281_MOESM11_ESM.zip › Figure7/7H/HE control Ileum.tif]

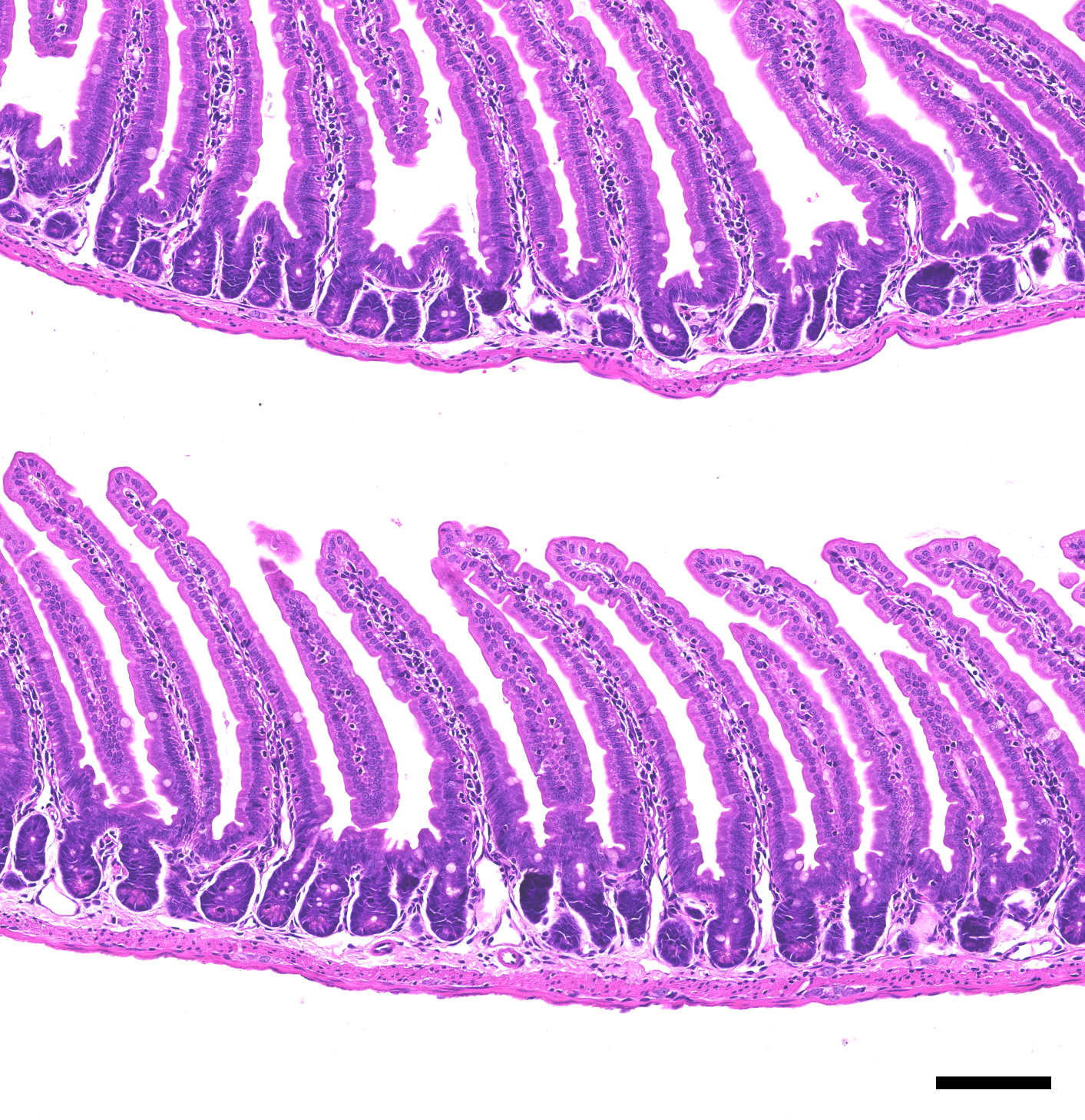

Supplement: Supplementary file 11 — Source data Fig. 7 [file 44318_2024_281_MOESM11_ESM.zip › Figure7/7H/HE control Jejunum.tif]

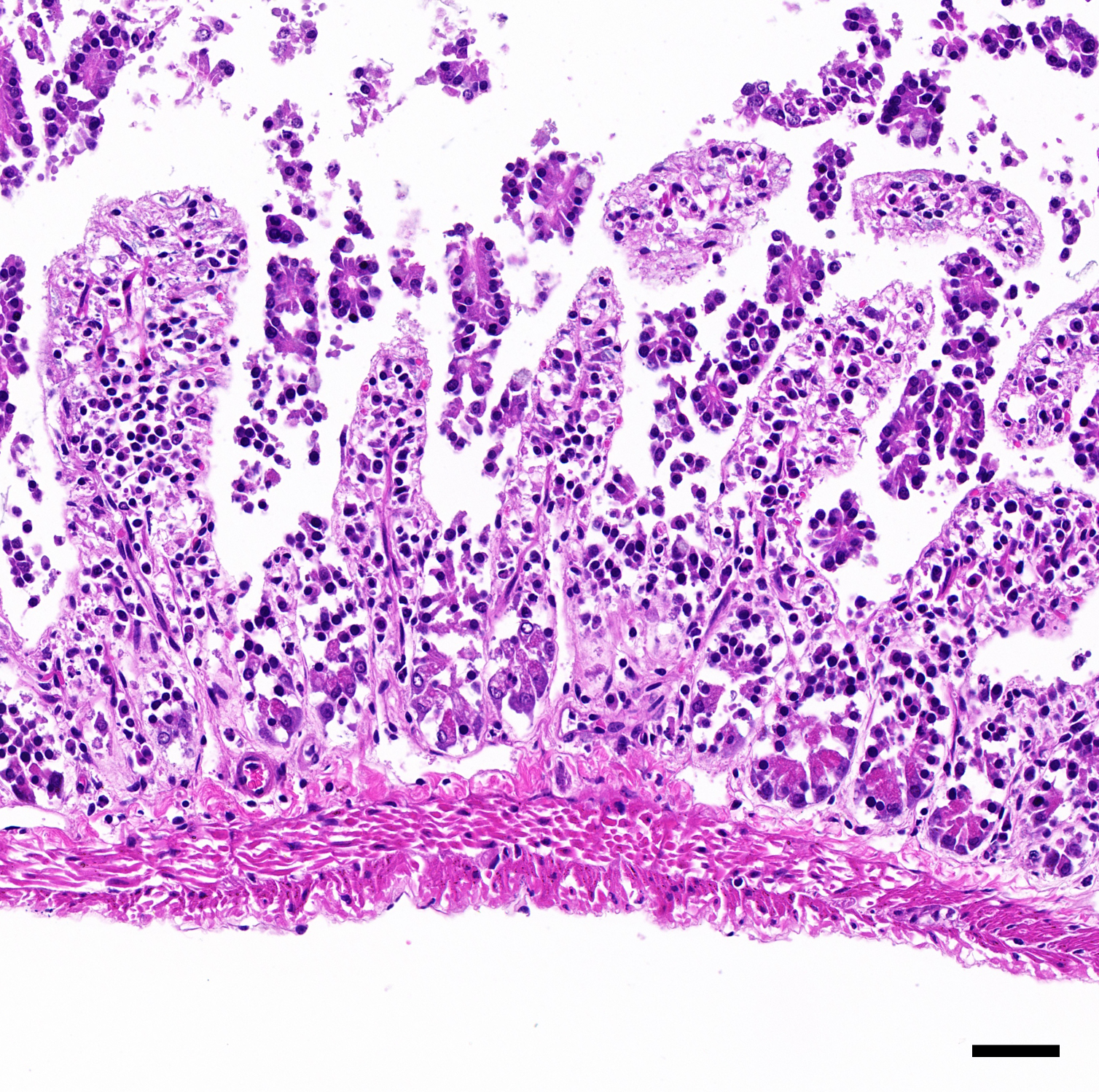

Supplement: Supplementary file 11 — Source data Fig. 7 [file 44318_2024_281_MOESM11_ESM.zip › Figure7/7M/HE Omicron SARS-CoV-2+CRD1.tif]

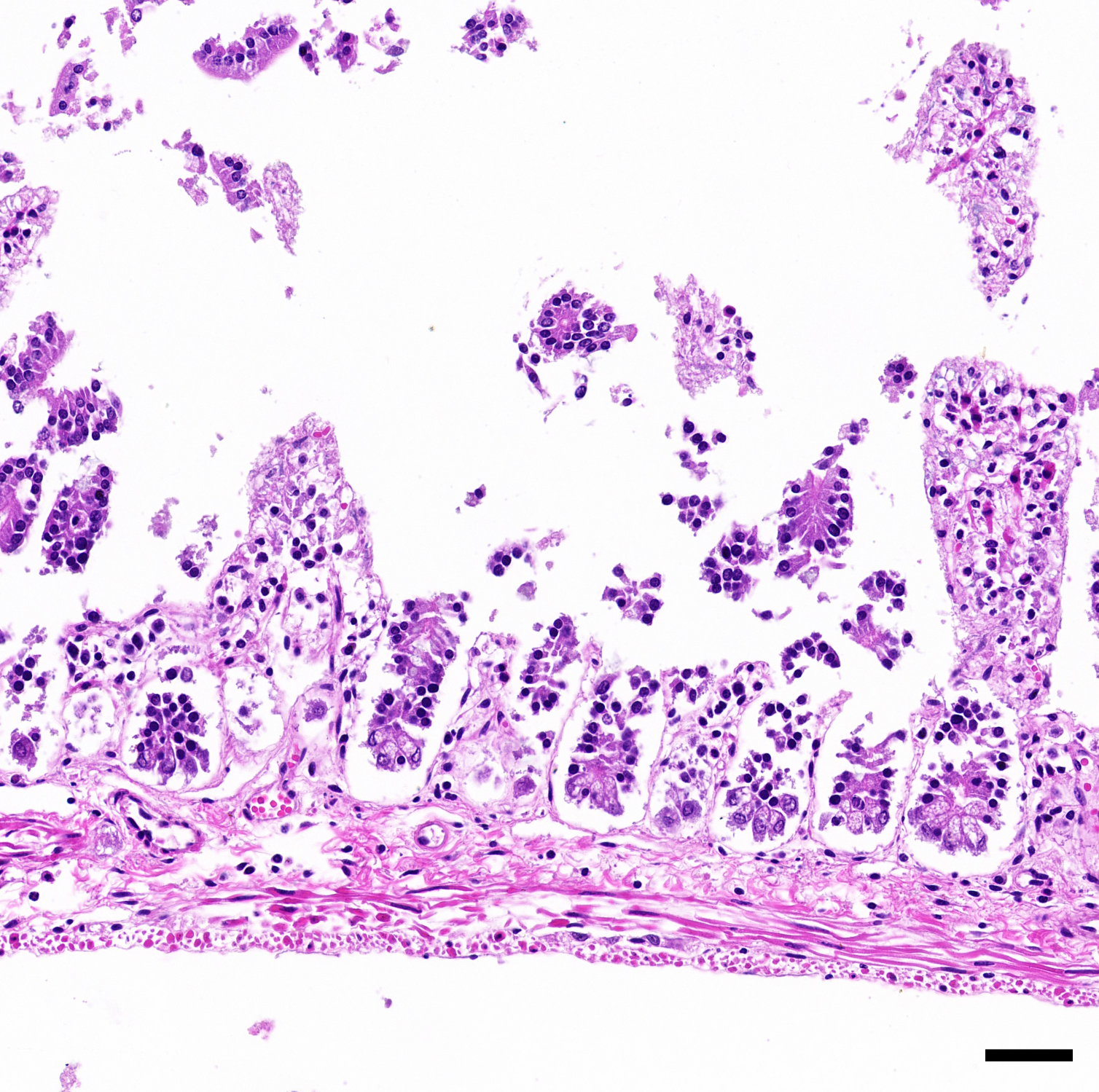

Supplement: Supplementary file 11 — Source data Fig. 7 [file 44318_2024_281_MOESM11_ESM.zip › Figure7/7M/HE Omicron SARS-CoV-2.tif]

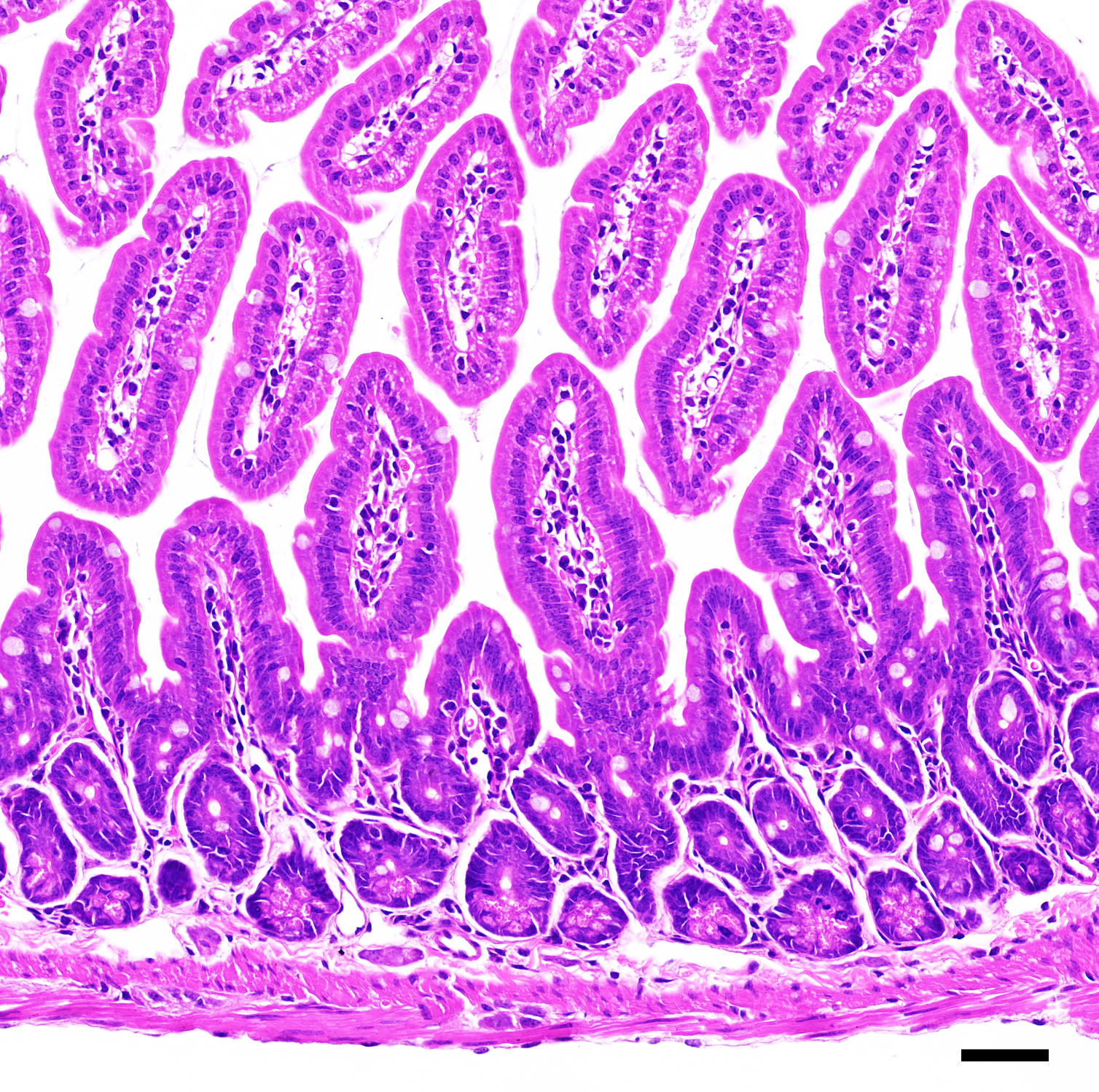

Supplement: Supplementary file 11 — Source data Fig. 7 [file 44318_2024_281_MOESM11_ESM.zip › Figure7/7M/HE Omicron control.tif]

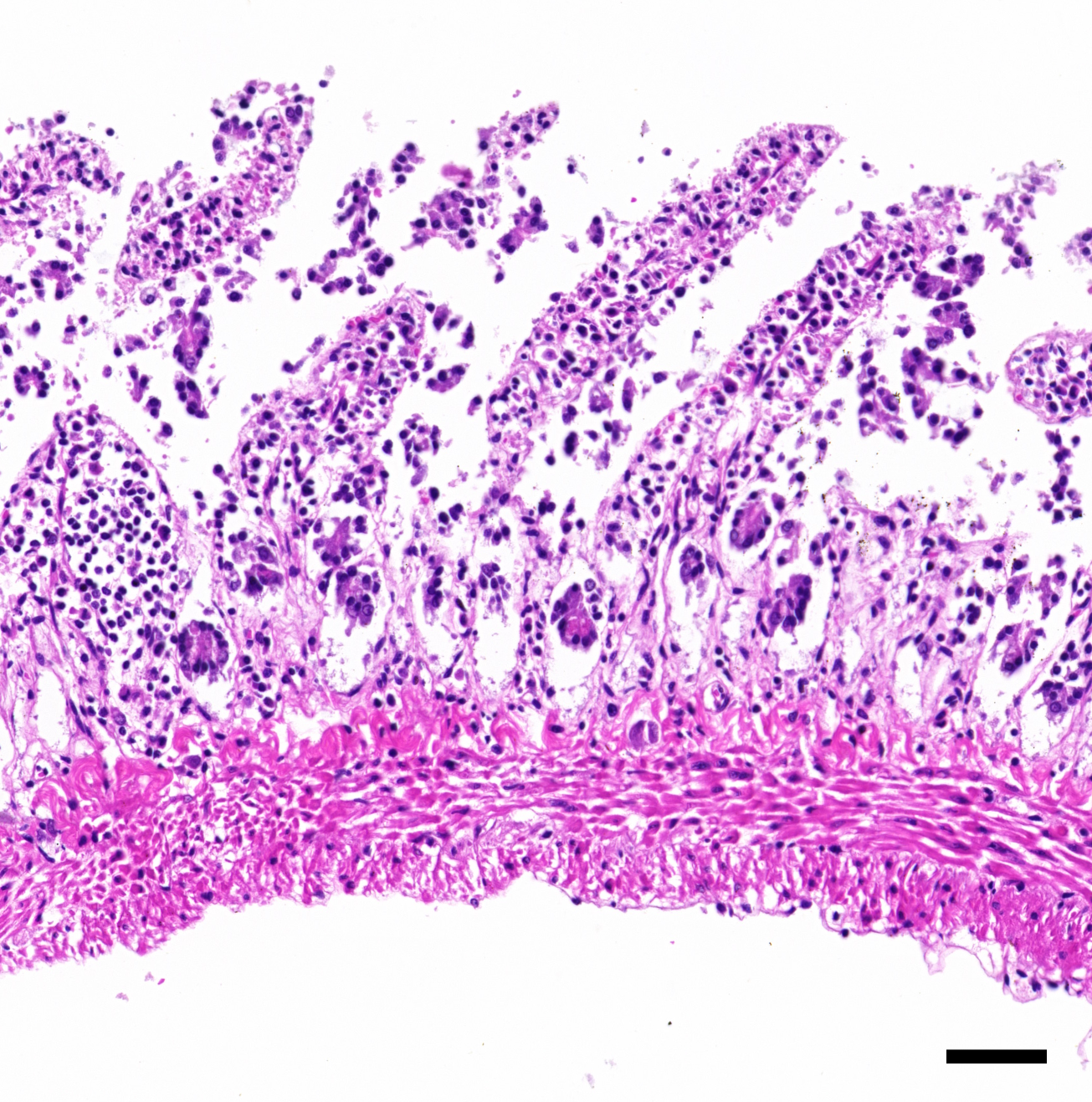

Supplement: Supplementary file 11 — Source data Fig. 7 [file 44318_2024_281_MOESM11_ESM.zip › Figure7/7M/HE WT SARS-CoV-2+CRD1.tif]

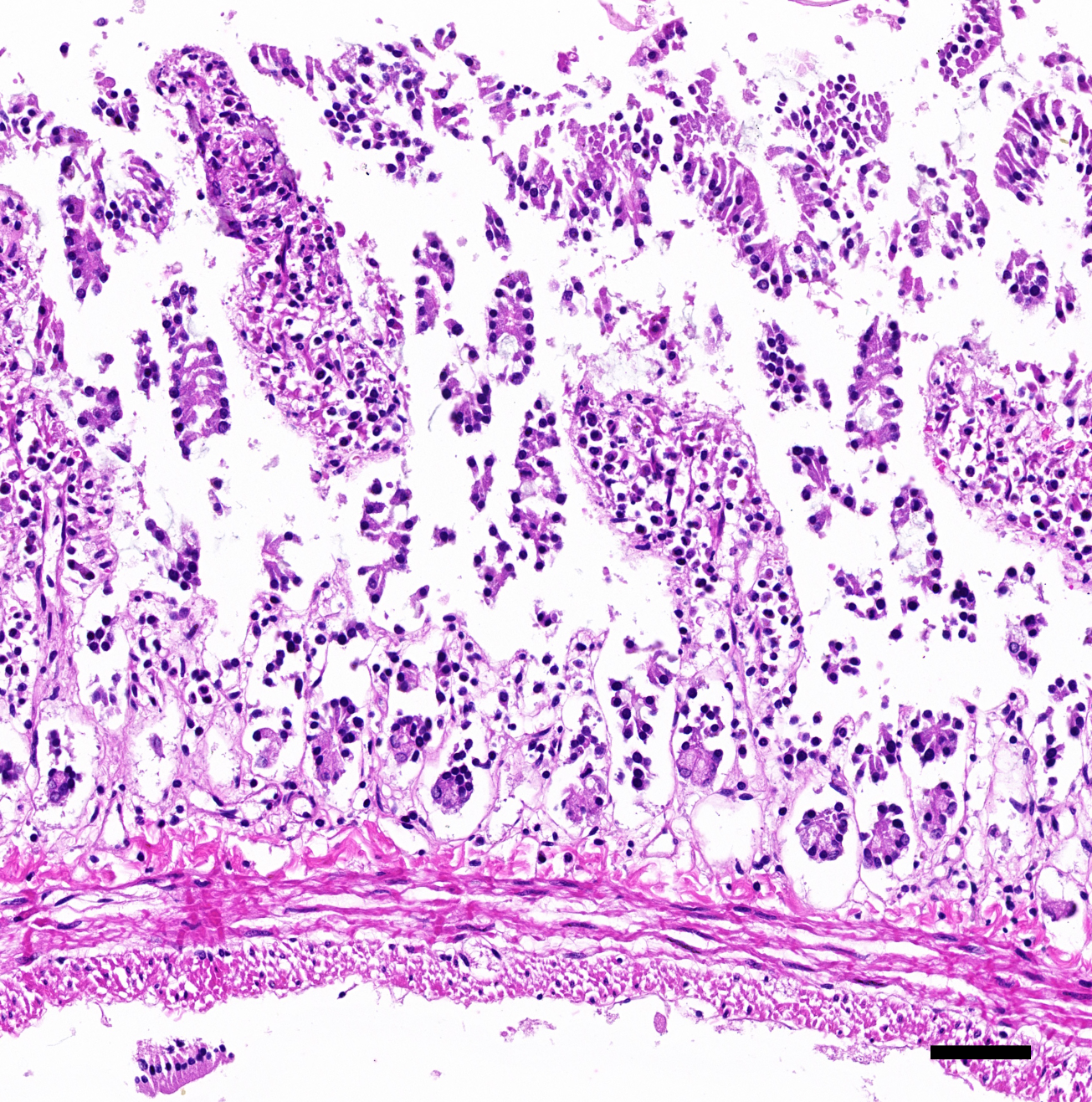

Supplement: Supplementary file 11 — Source data Fig. 7 [file 44318_2024_281_MOESM11_ESM.zip › Figure7/7M/HE WT SARS-CoV-2.tif]

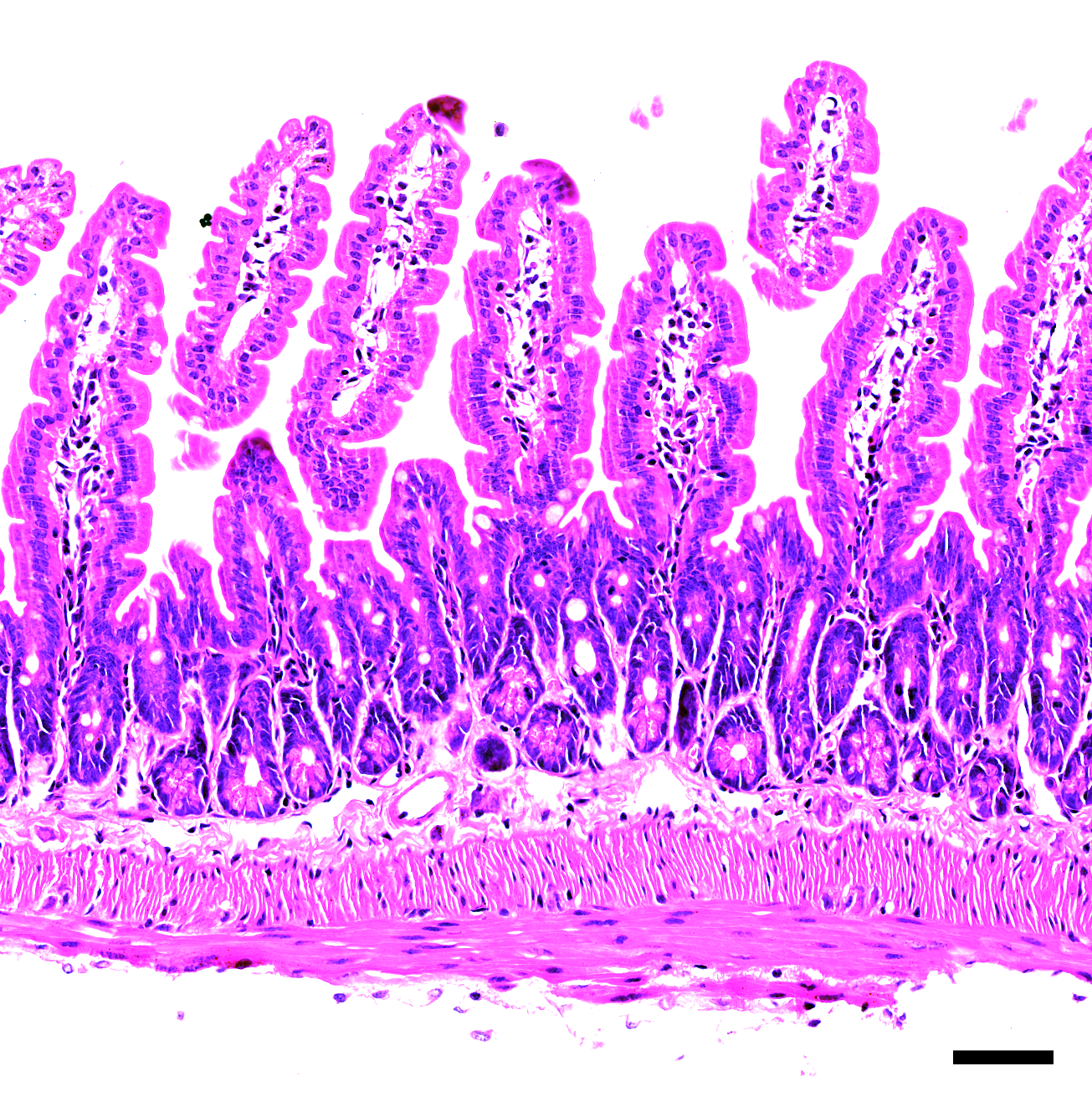

Supplement: Supplementary file 11 — Source data Fig. 7 [file 44318_2024_281_MOESM11_ESM.zip › Figure7/7M/HE WT control.tif]

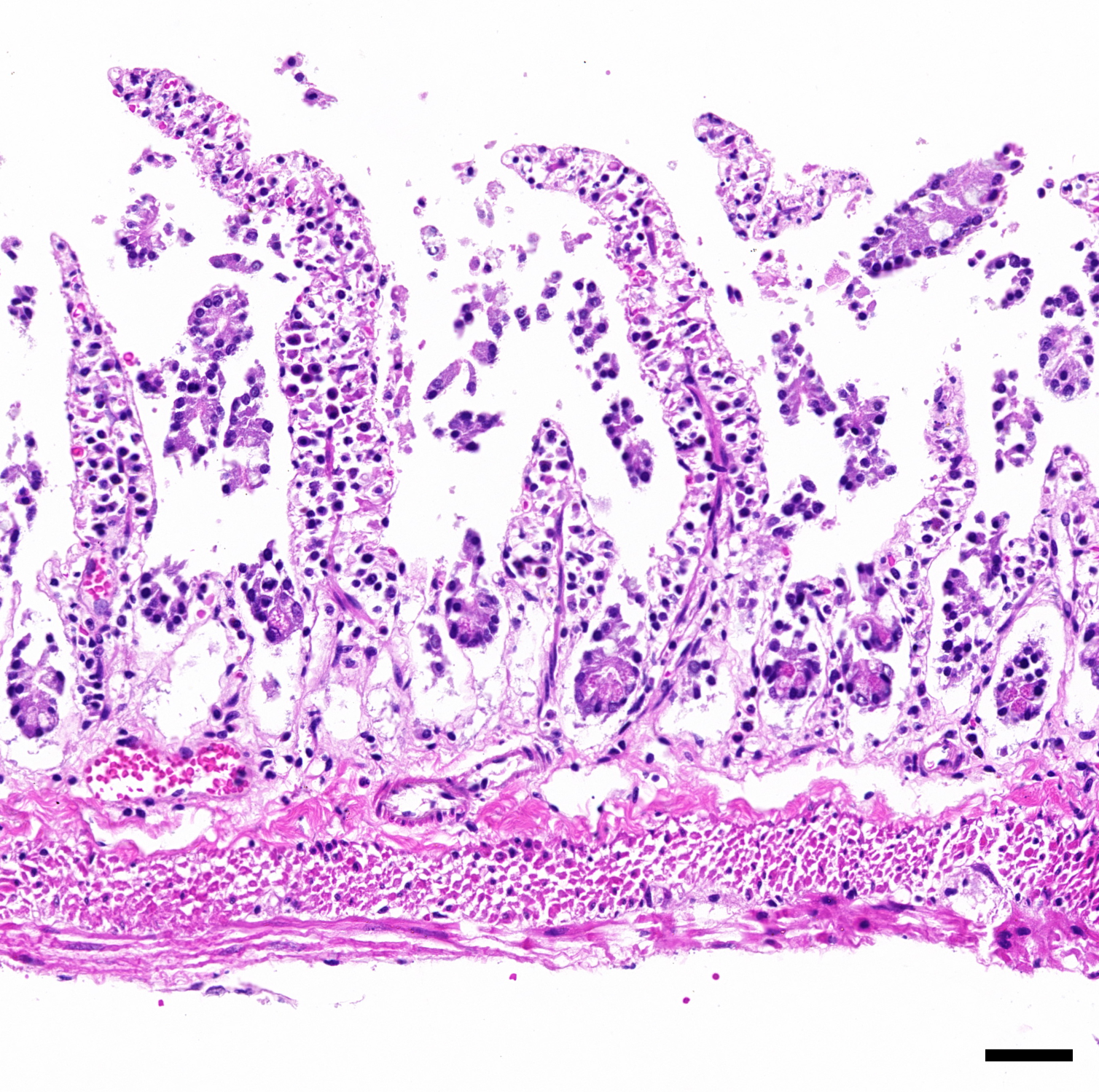

Supplement: Supplementary file 11 — Source data Fig. 7 [file 44318_2024_281_MOESM11_ESM.zip › Figure7/7M/HE XBB SARS-CoV-2+CRD1.tif]

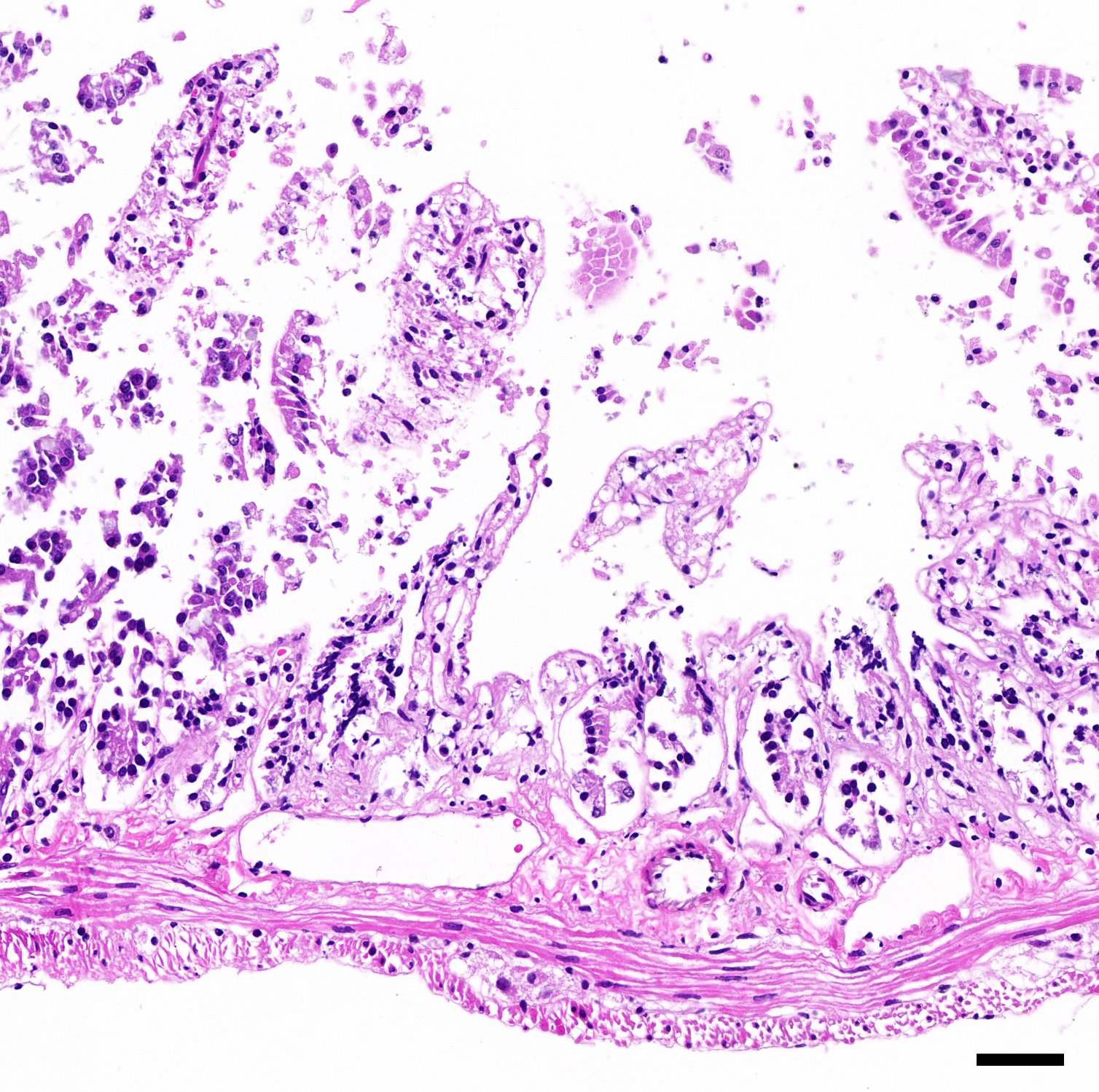

Supplement: Supplementary file 11 — Source data Fig. 7 [file 44318_2024_281_MOESM11_ESM.zip › Figure7/7M/HE XBB SARS-CoV-2.tif]

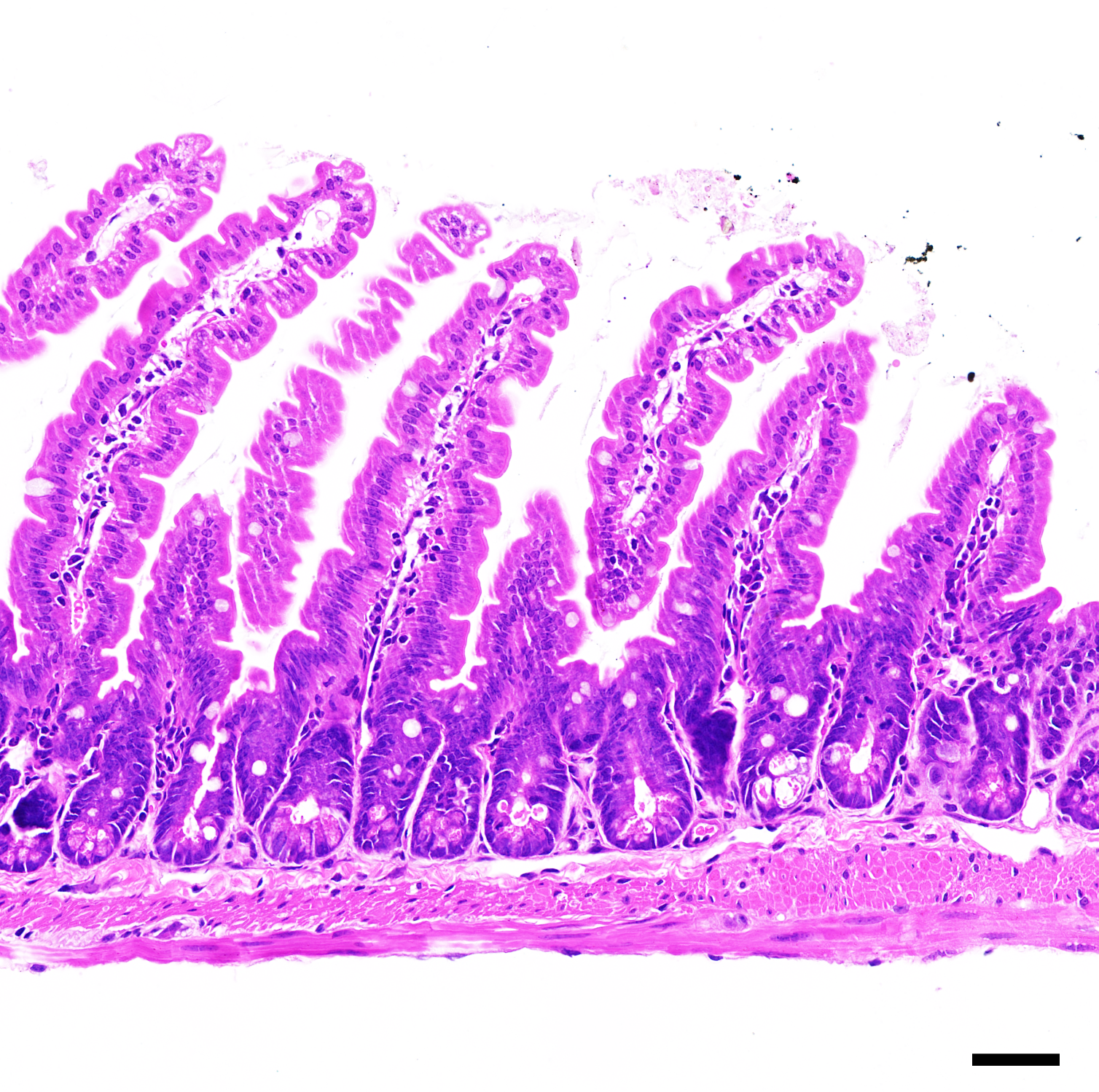

Supplement: Supplementary file 11 — Source data Fig. 7 [file 44318_2024_281_MOESM11_ESM.zip › Figure7/7M/HE XBB control.tif]

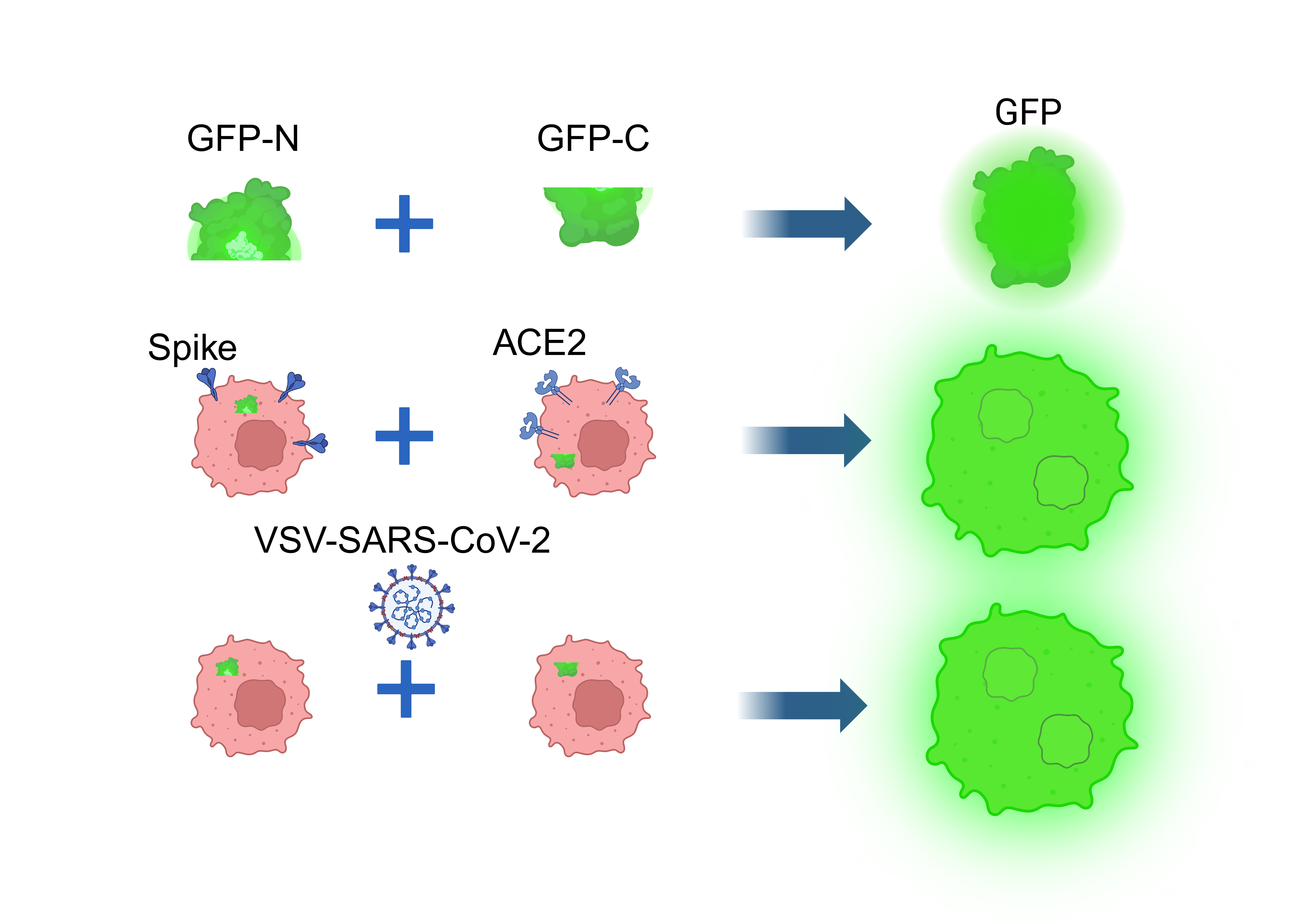

Supplement: Supplementary file 12 — Source data Fig. 8 [file 44318_2024_281_MOESM12_ESM.zip › Figure8/8A/The schematic principle of the GFP-based BiFC assay.jpeg]

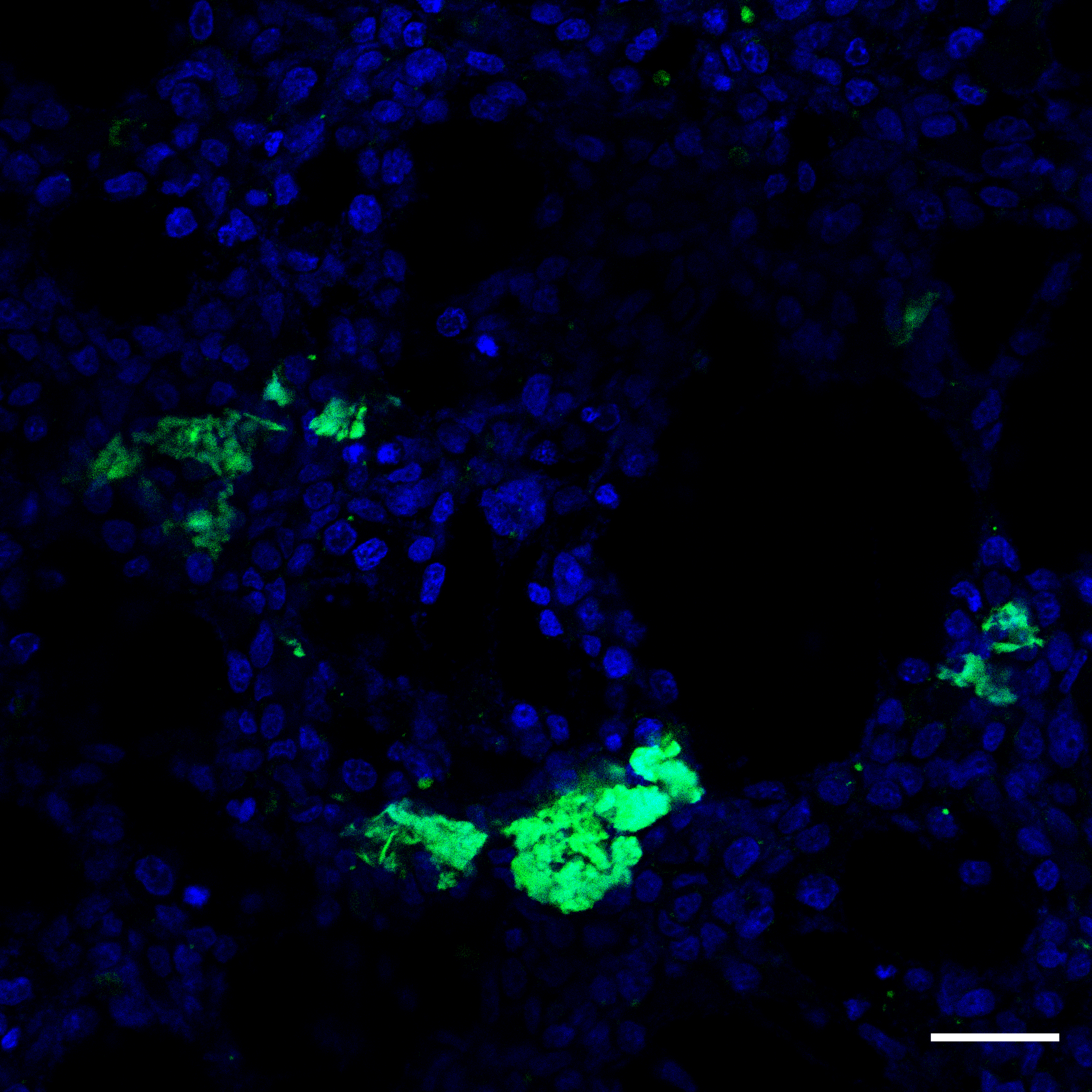

Supplement: Supplementary file 12 — Source data Fig. 8 [file 44318_2024_281_MOESM12_ESM.zip › Figure8/8B/GFP 0.1 μg LSR.tif]

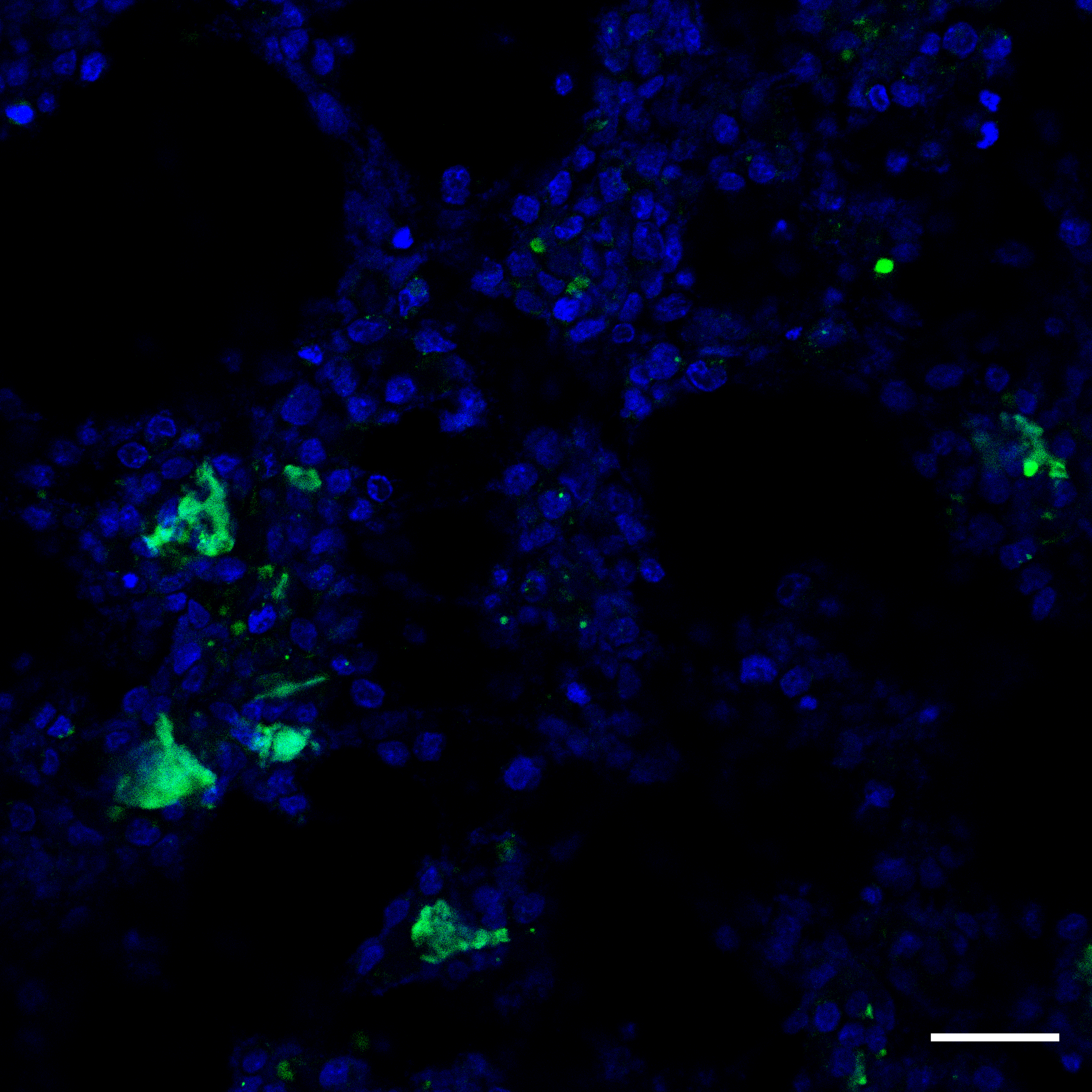

Supplement: Supplementary file 12 — Source data Fig. 8 [file 44318_2024_281_MOESM12_ESM.zip › Figure8/8B/GFP 0.5 μg LSR.tif]

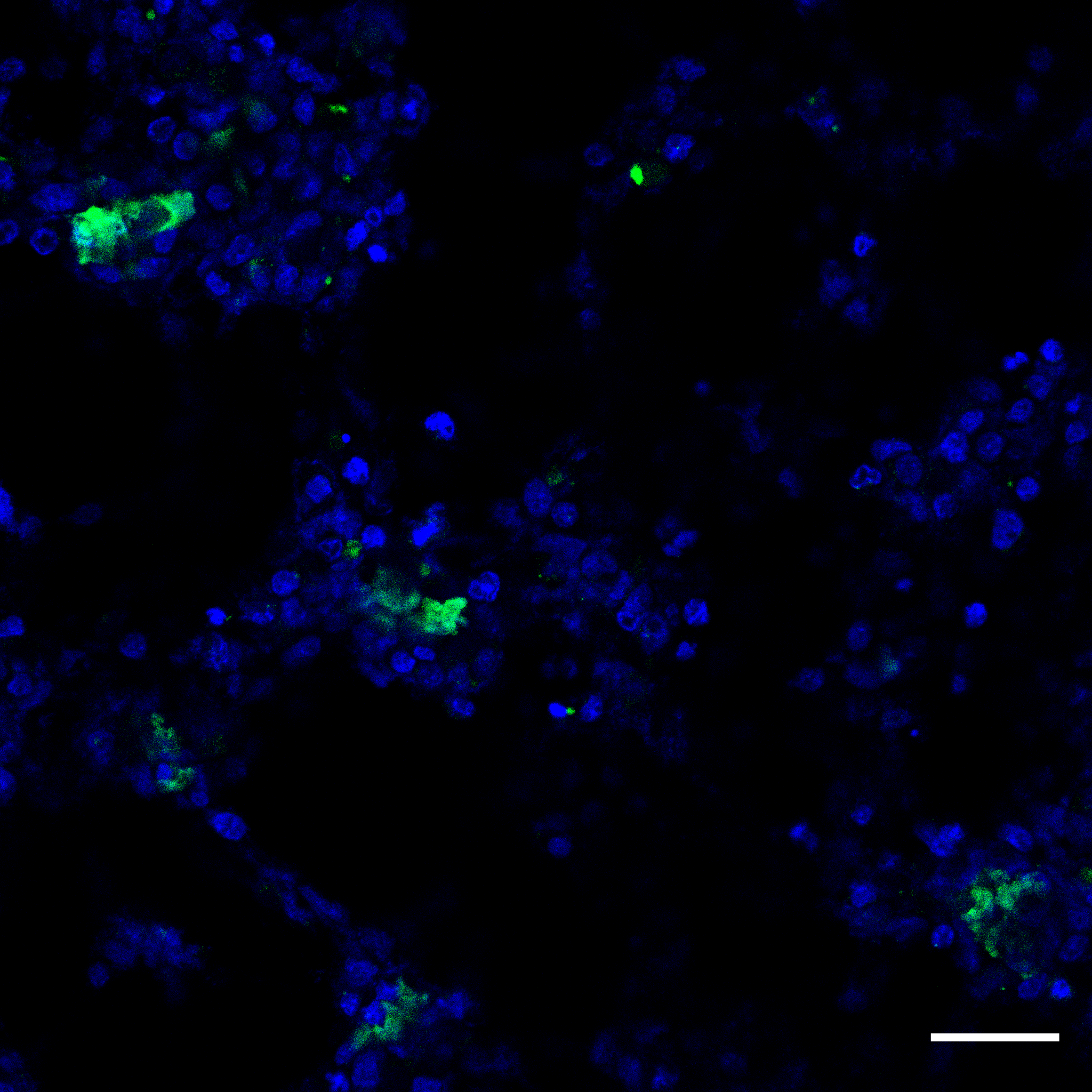

Supplement: Supplementary file 12 — Source data Fig. 8 [file 44318_2024_281_MOESM12_ESM.zip › Figure8/8B/GFP 1 μg LSR.tif]

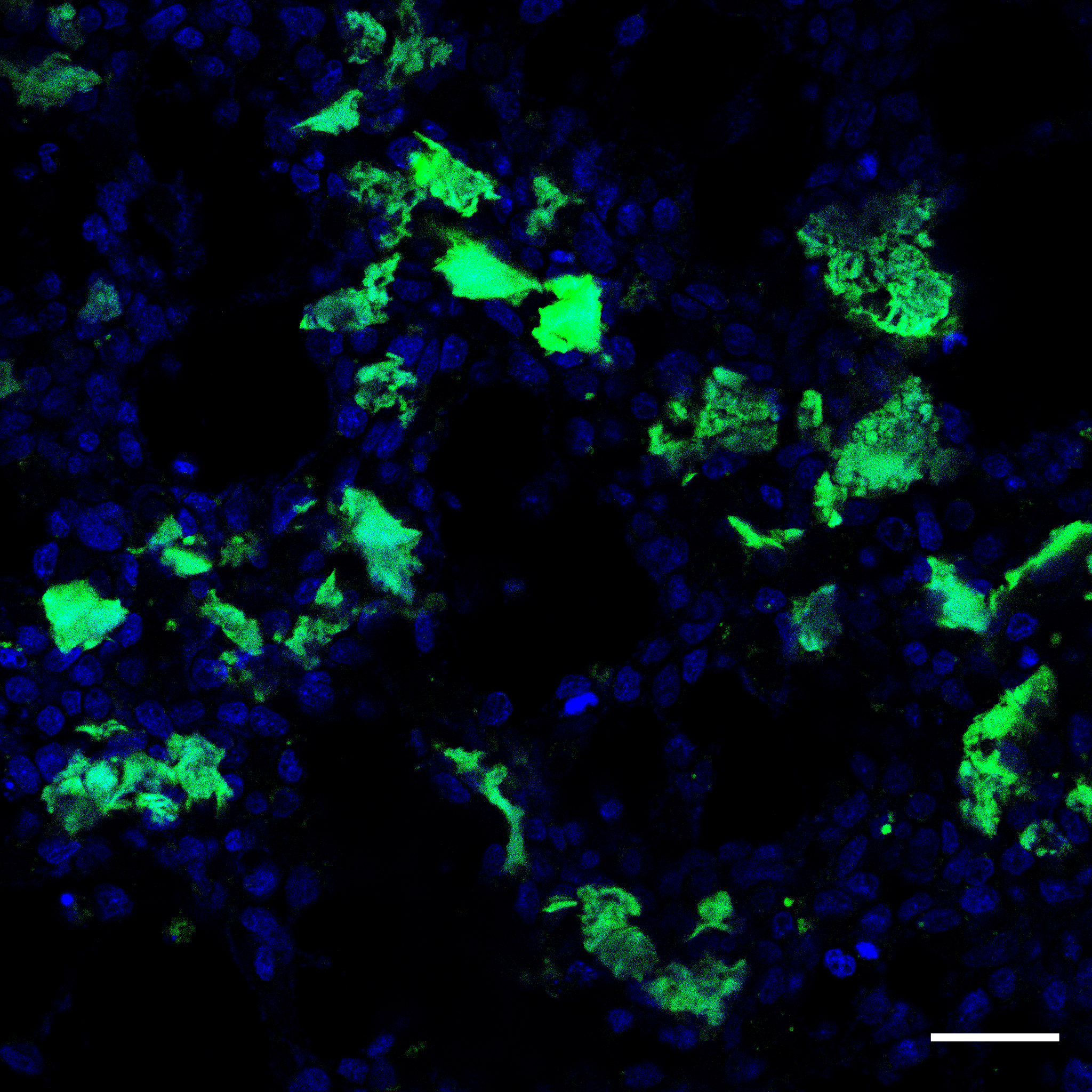

Supplement: Supplementary file 12 — Source data Fig. 8 [file 44318_2024_281_MOESM12_ESM.zip › Figure8/8B/GFP LSR-KD.tif]

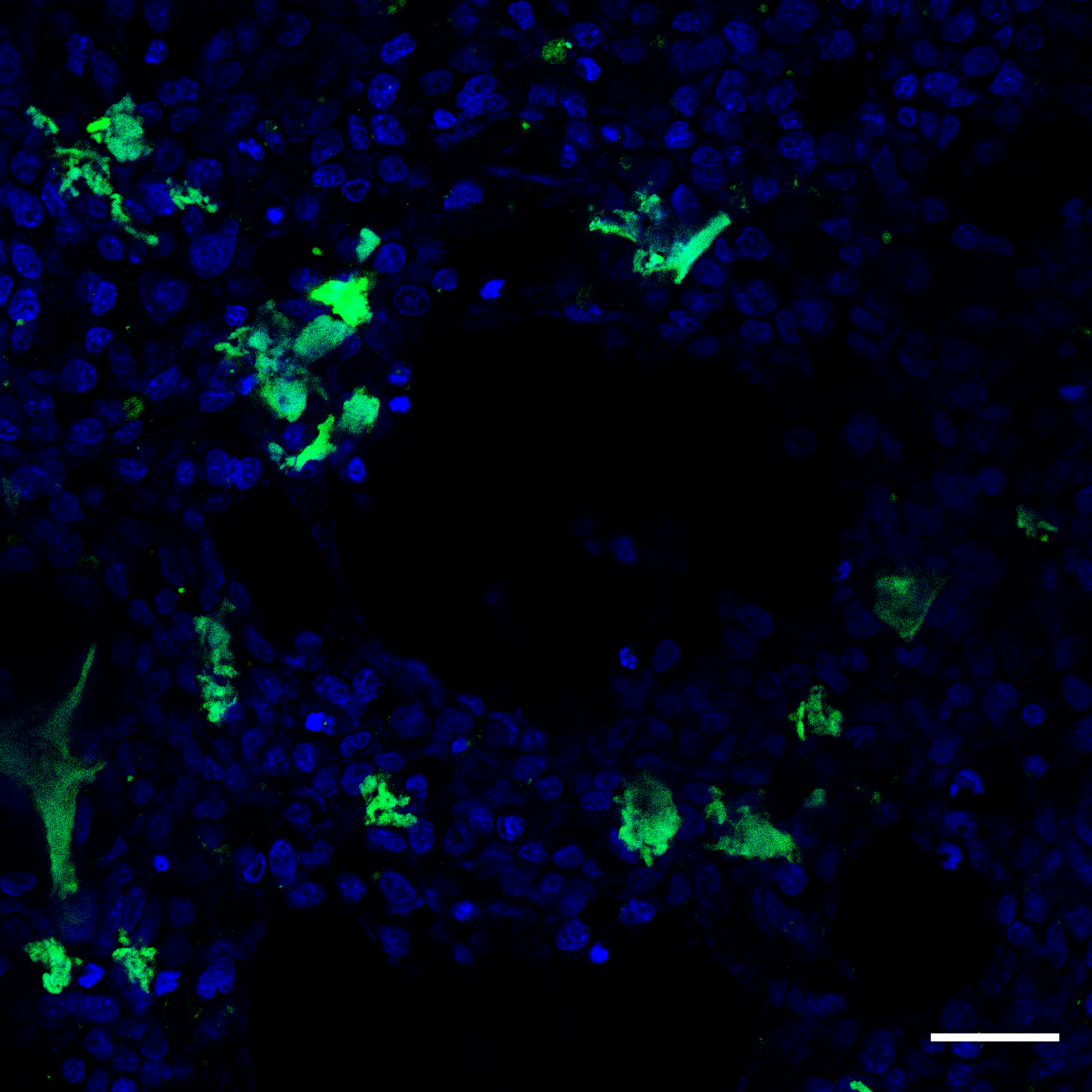

Supplement: Supplementary file 12 — Source data Fig. 8 [file 44318_2024_281_MOESM12_ESM.zip › Figure8/8B/GFP control.tif]

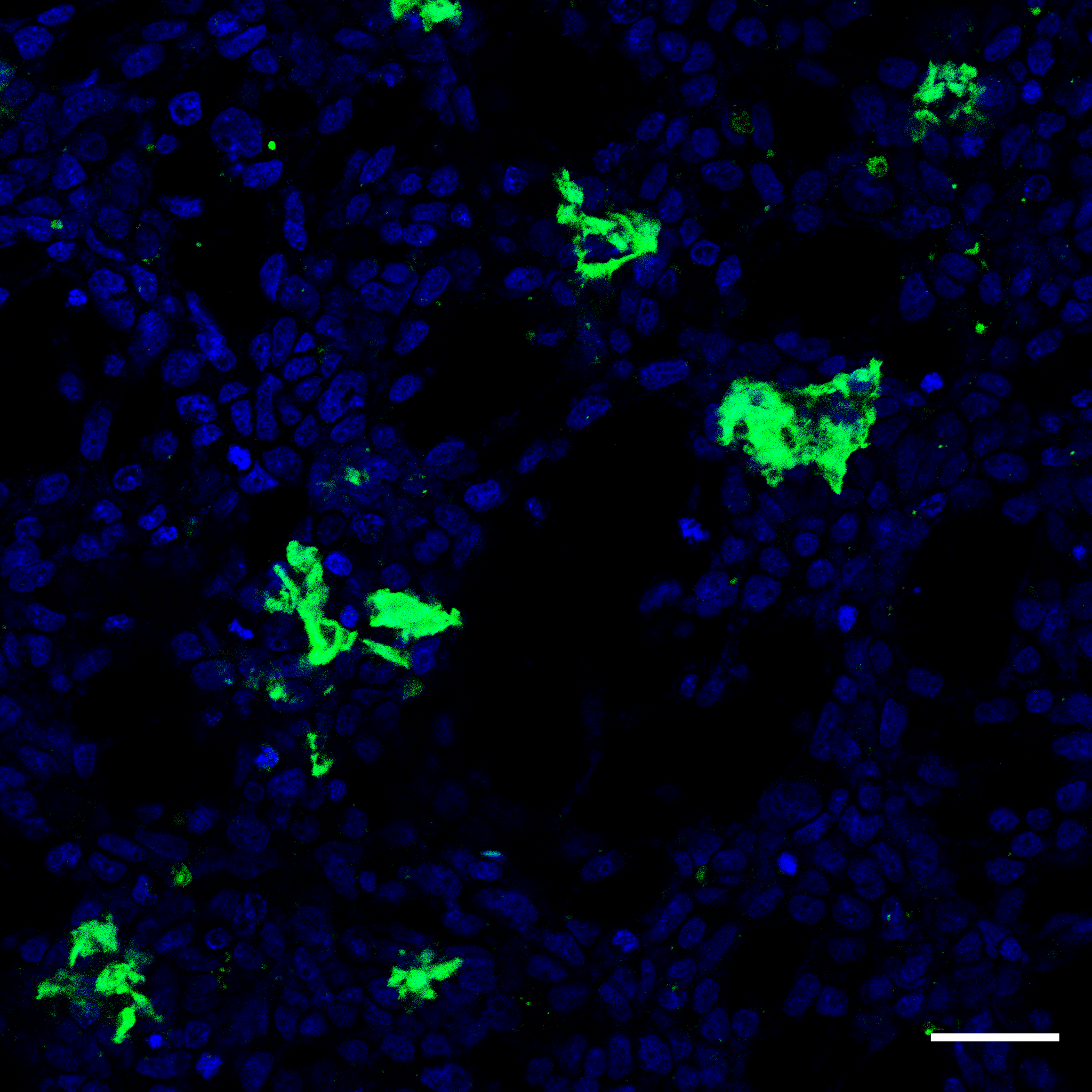

Supplement: Supplementary file 12 — Source data Fig. 8 [file 44318_2024_281_MOESM12_ESM.zip › Figure8/8C/GFP 0.1 μg LSR.tif]

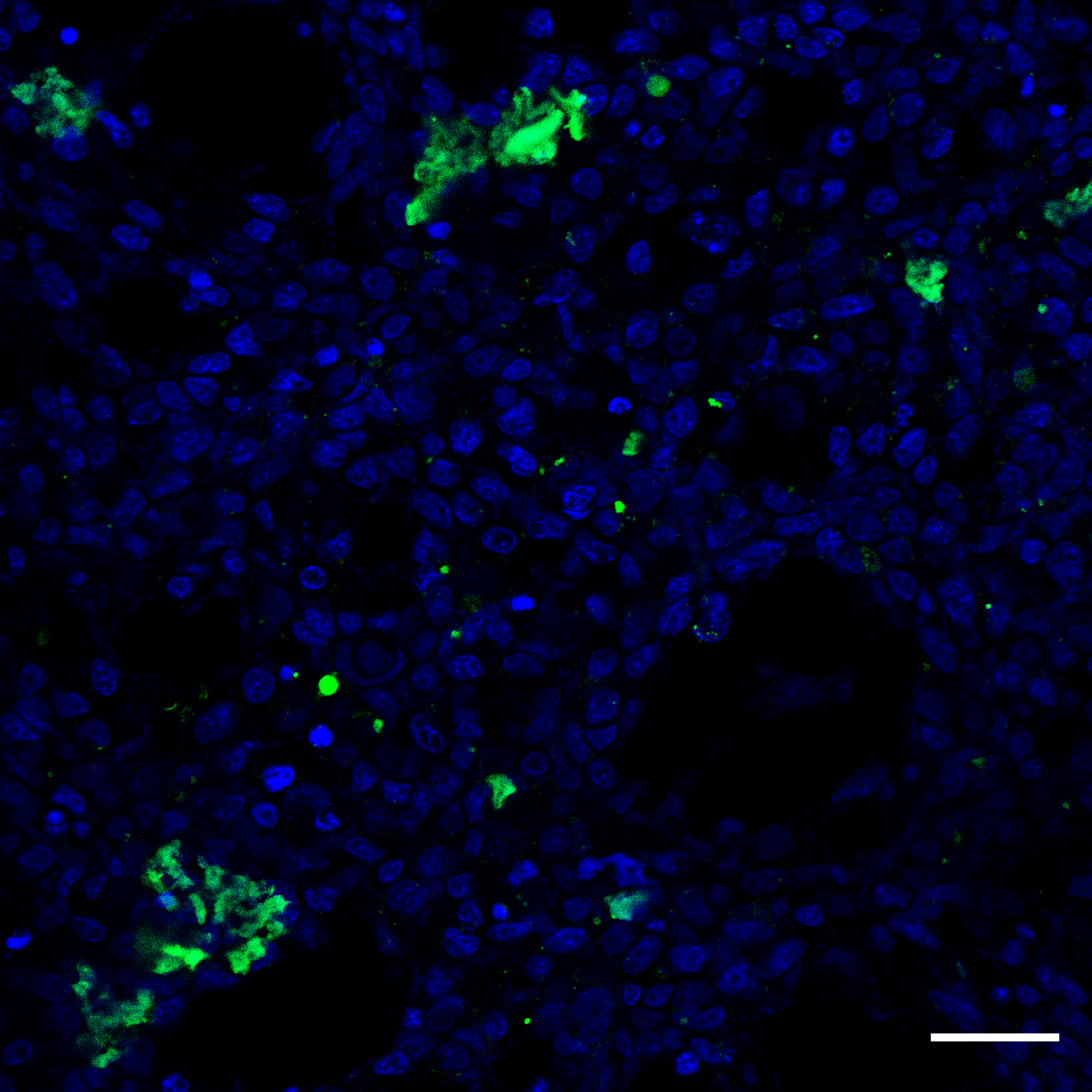

Supplement: Supplementary file 12 — Source data Fig. 8 [file 44318_2024_281_MOESM12_ESM.zip › Figure8/8C/GFP 0.5 μg LSR.tif]

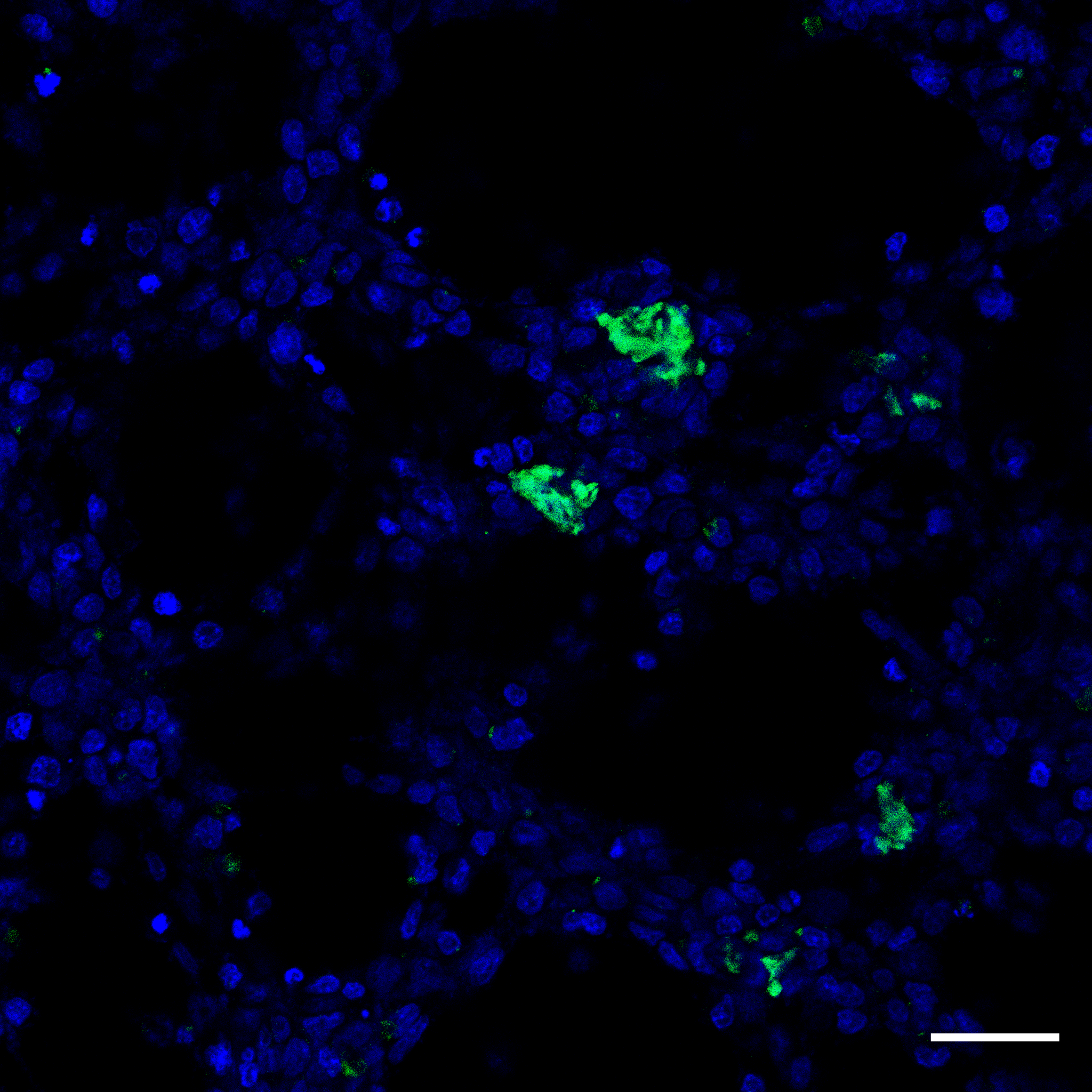

Supplement: Supplementary file 12 — Source data Fig. 8 [file 44318_2024_281_MOESM12_ESM.zip › Figure8/8C/GFP 1 μg LSR.tif]

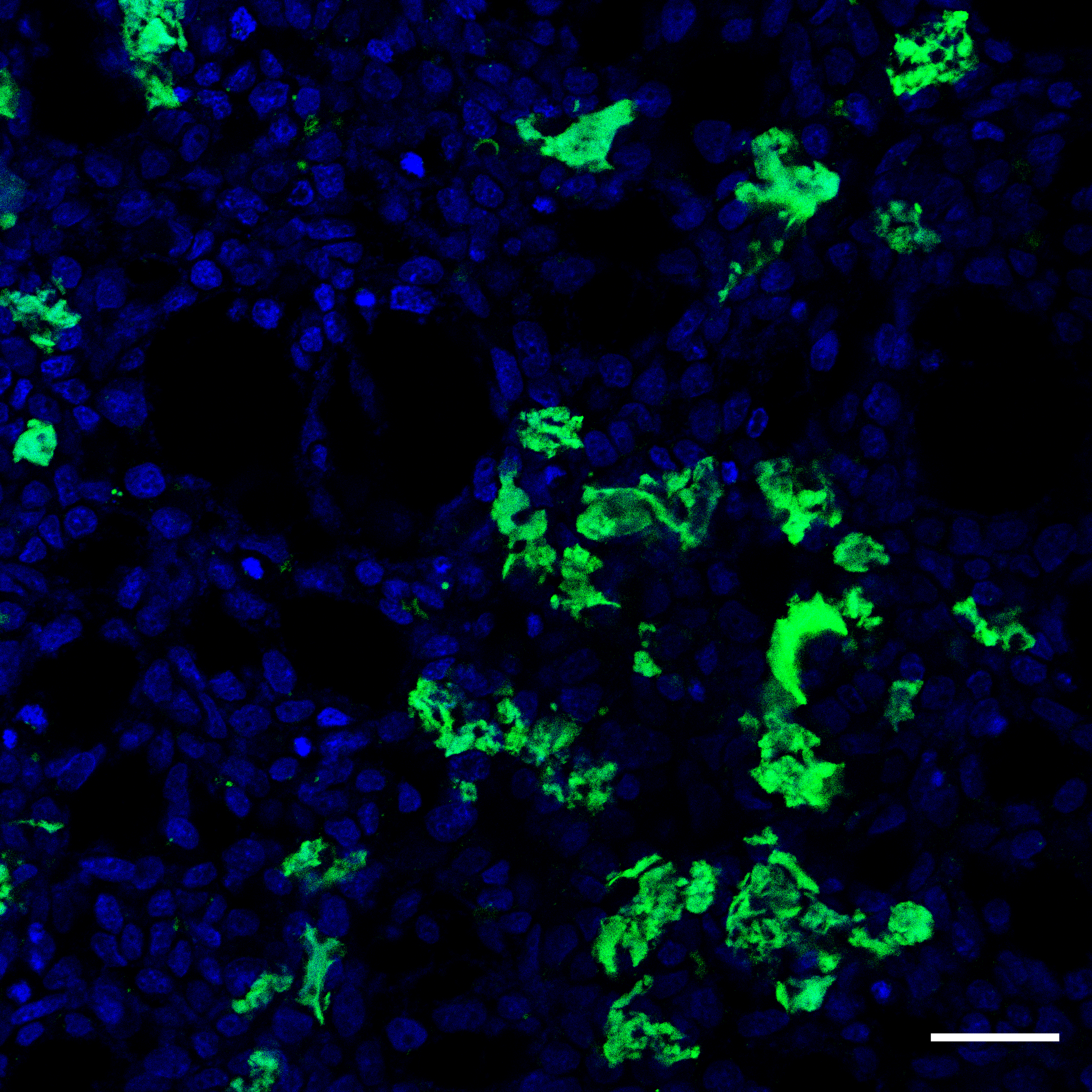

Supplement: Supplementary file 12 — Source data Fig. 8 [file 44318_2024_281_MOESM12_ESM.zip › Figure8/8C/GFP LSR-KD.tif]

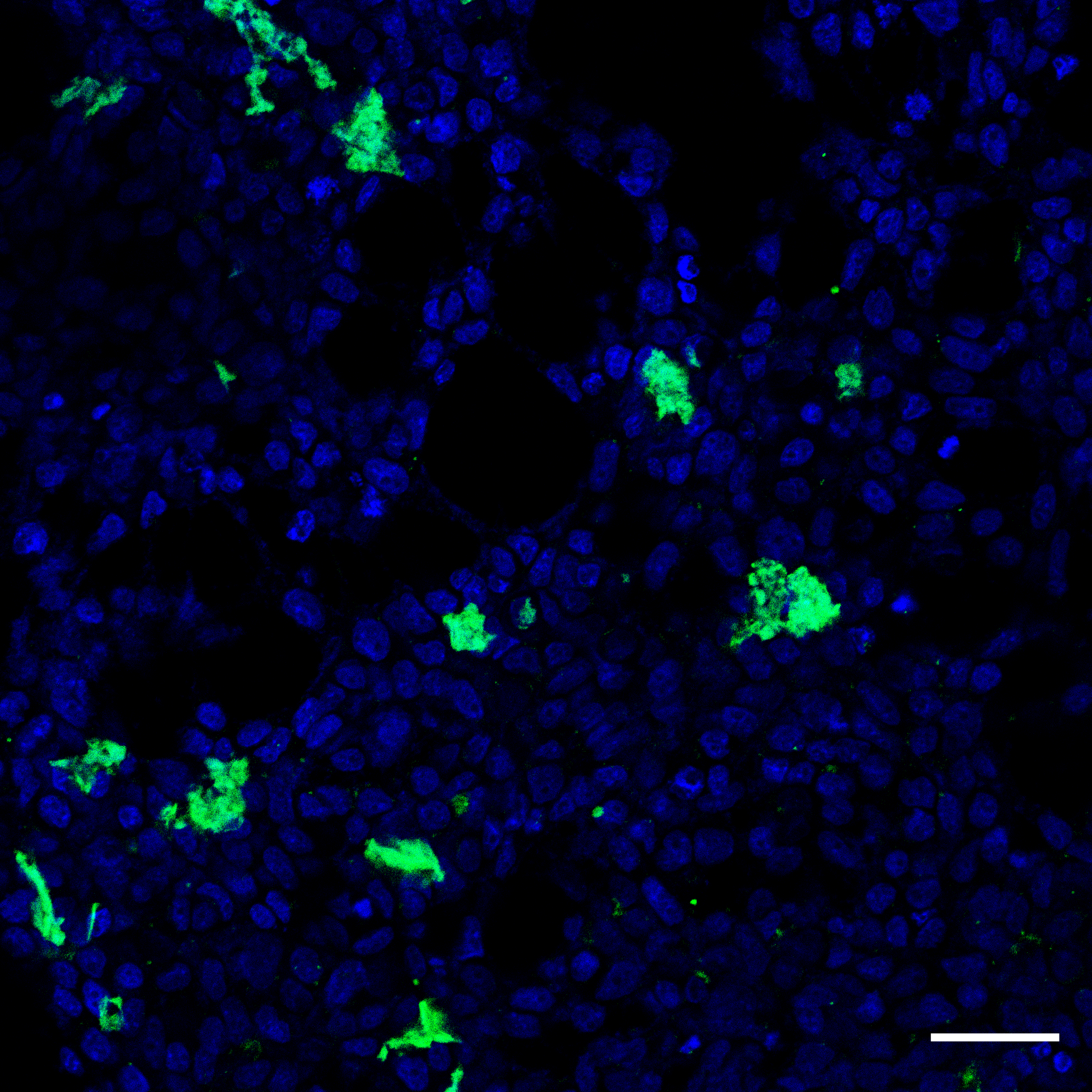

Supplement: Supplementary file 12 — Source data Fig. 8 [file 44318_2024_281_MOESM12_ESM.zip › Figure8/8C/GFP control.tif]

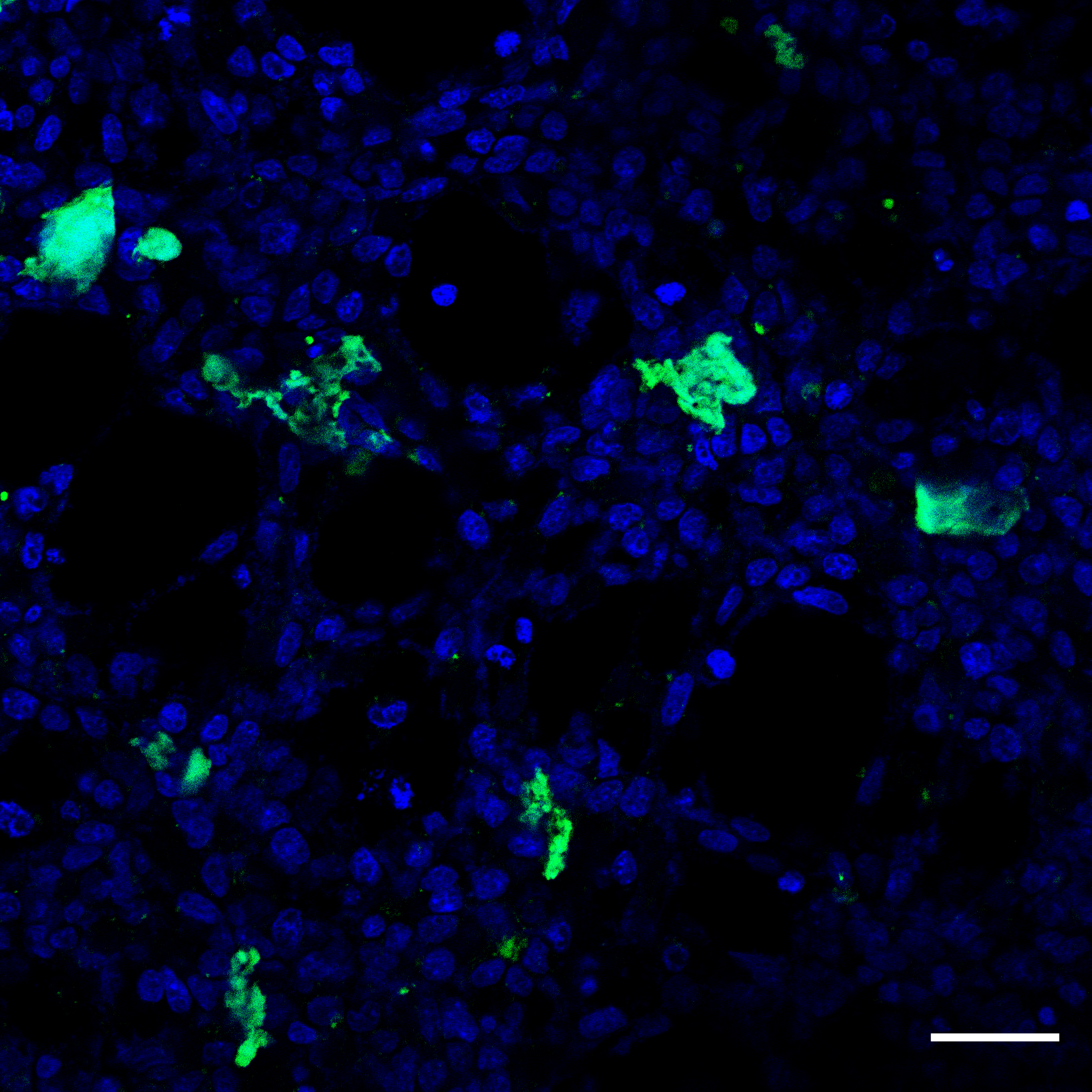

Supplement: Supplementary file 12 — Source data Fig. 8 [file 44318_2024_281_MOESM12_ESM.zip › Figure8/8D/GFP 0.1 μg LSR.tif]

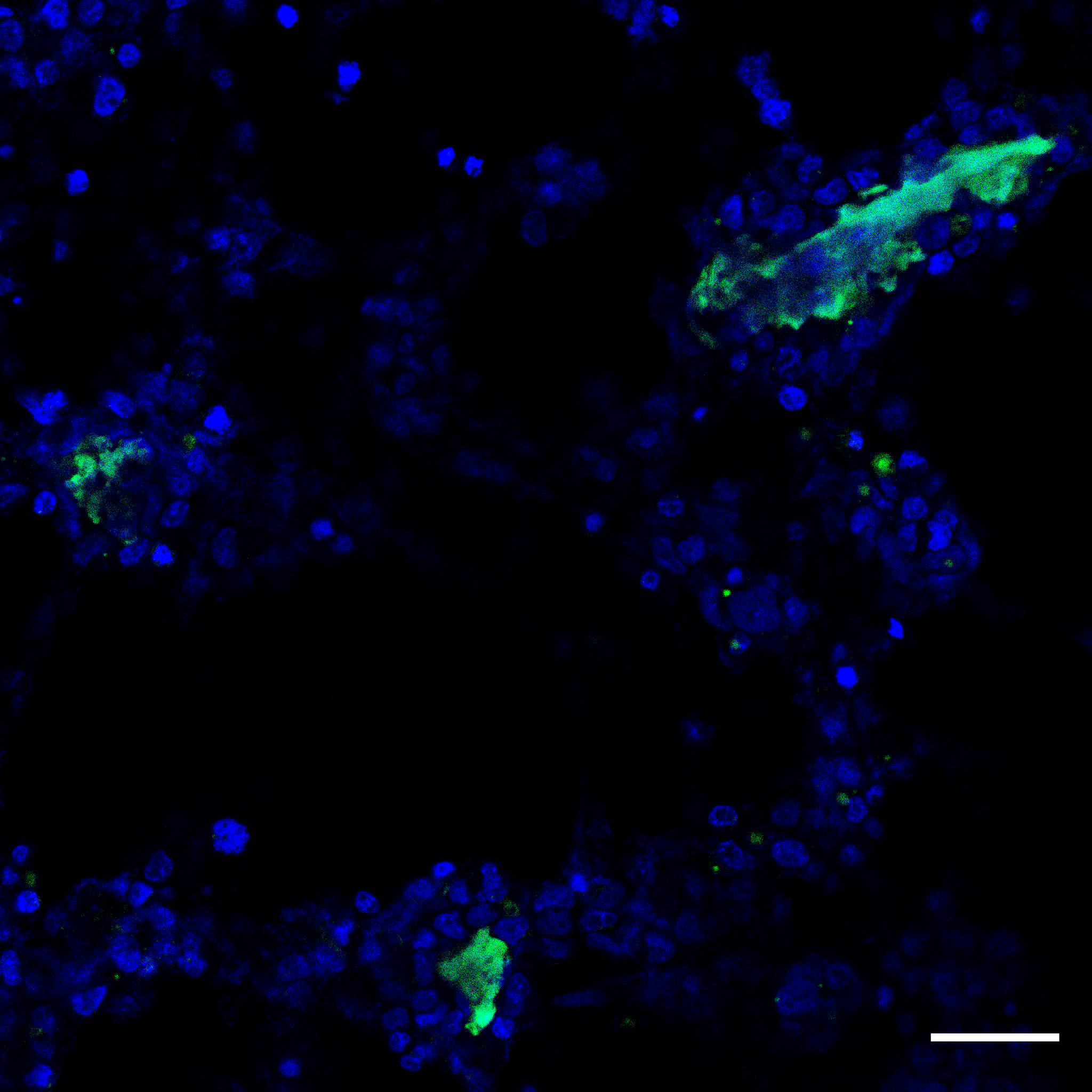

Supplement: Supplementary file 12 — Source data Fig. 8 [file 44318_2024_281_MOESM12_ESM.zip › Figure8/8D/GFP 0.5 μg LSR.tif]

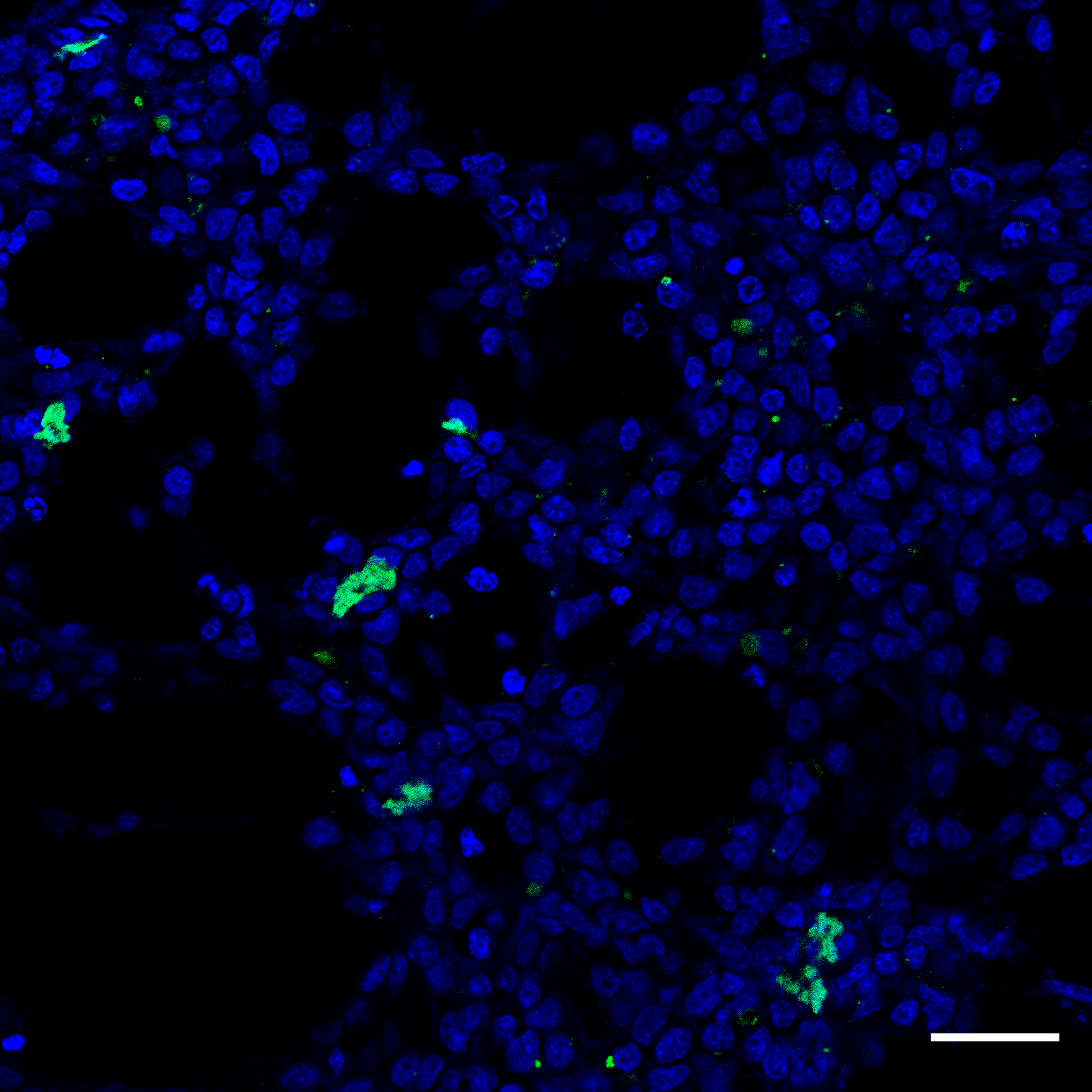

Supplement: Supplementary file 12 — Source data Fig. 8 [file 44318_2024_281_MOESM12_ESM.zip › Figure8/8D/GFP 1 μg LSR.tif]

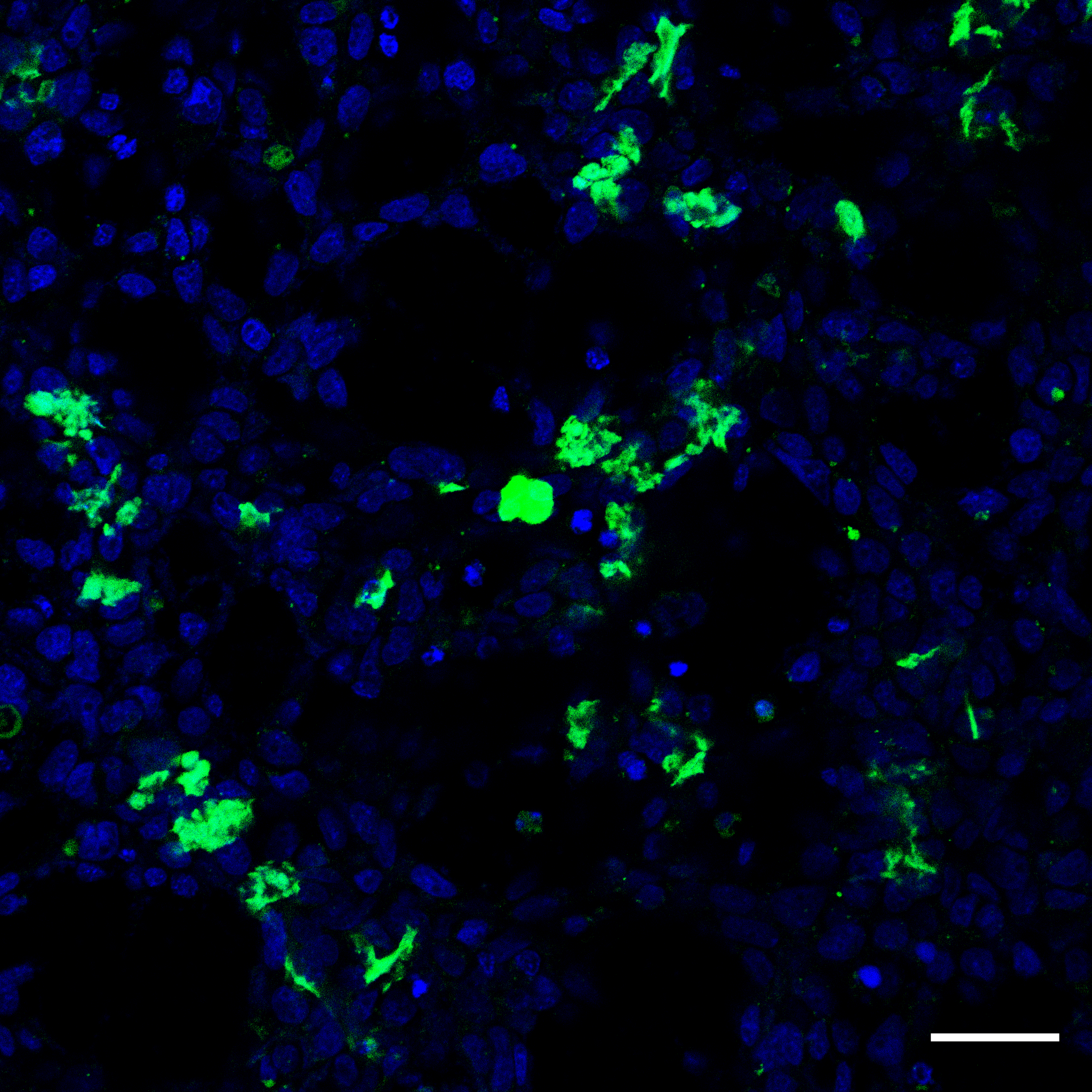

Supplement: Supplementary file 12 — Source data Fig. 8 [file 44318_2024_281_MOESM12_ESM.zip › Figure8/8D/GFP LSR-KD.tif]

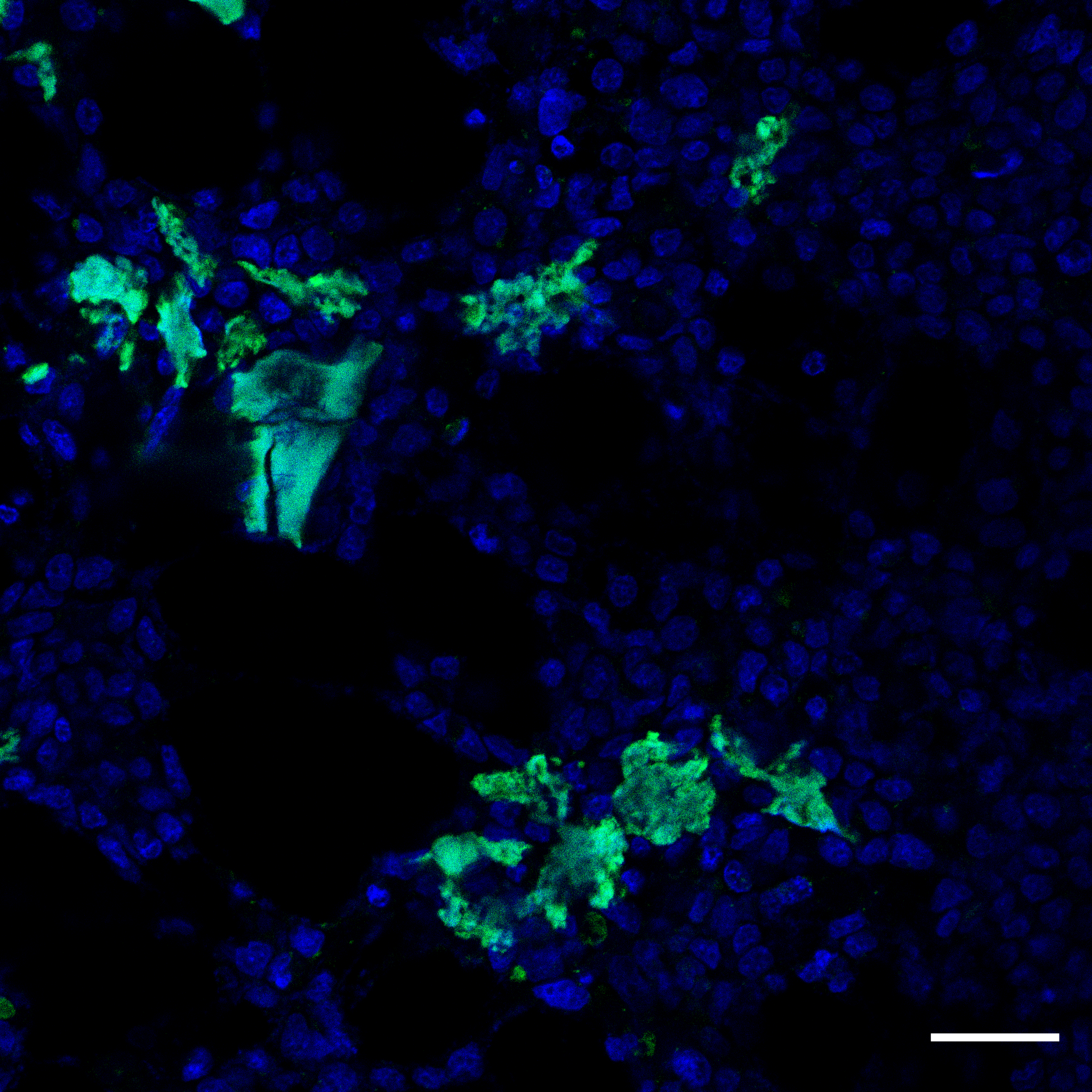

Supplement: Supplementary file 12 — Source data Fig. 8 [file 44318_2024_281_MOESM12_ESM.zip › Figure8/8D/GFP control.tif]

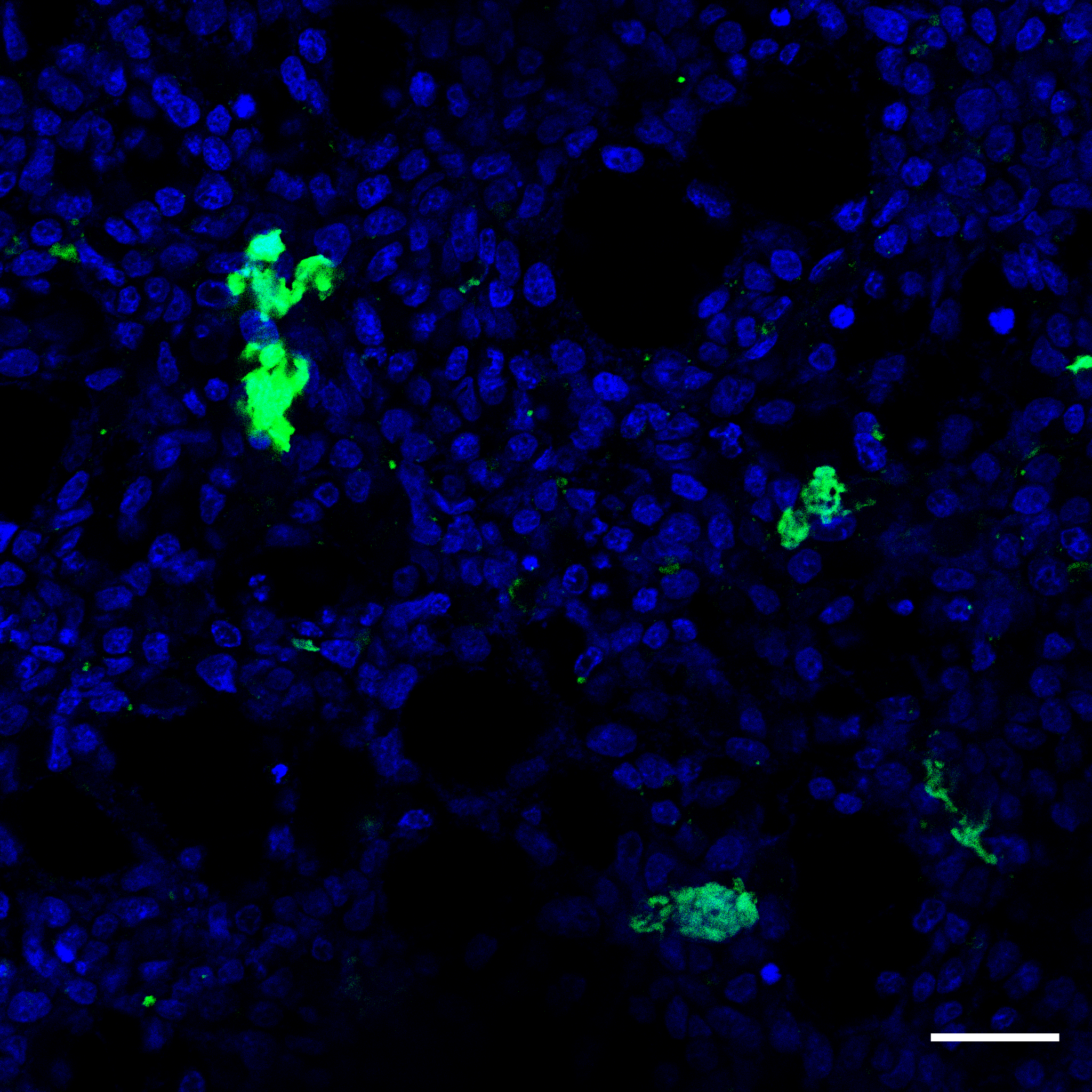

Supplement: Supplementary file 12 — Source data Fig. 8 [file 44318_2024_281_MOESM12_ESM.zip › Figure8/8E/GFP 10 CRD1.tif]

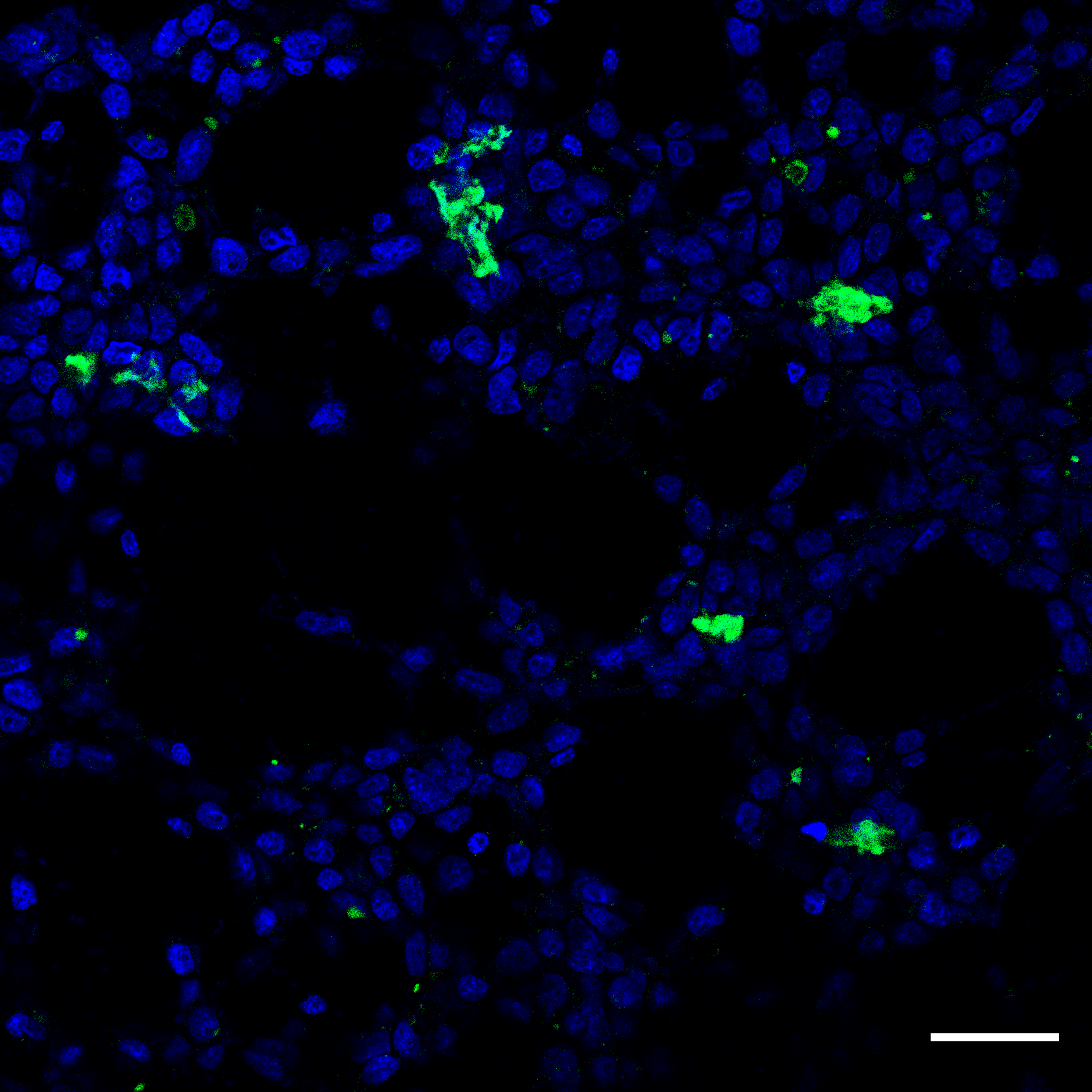

Supplement: Supplementary file 12 — Source data Fig. 8 [file 44318_2024_281_MOESM12_ESM.zip › Figure8/8E/GFP 20 CRD1.tif]

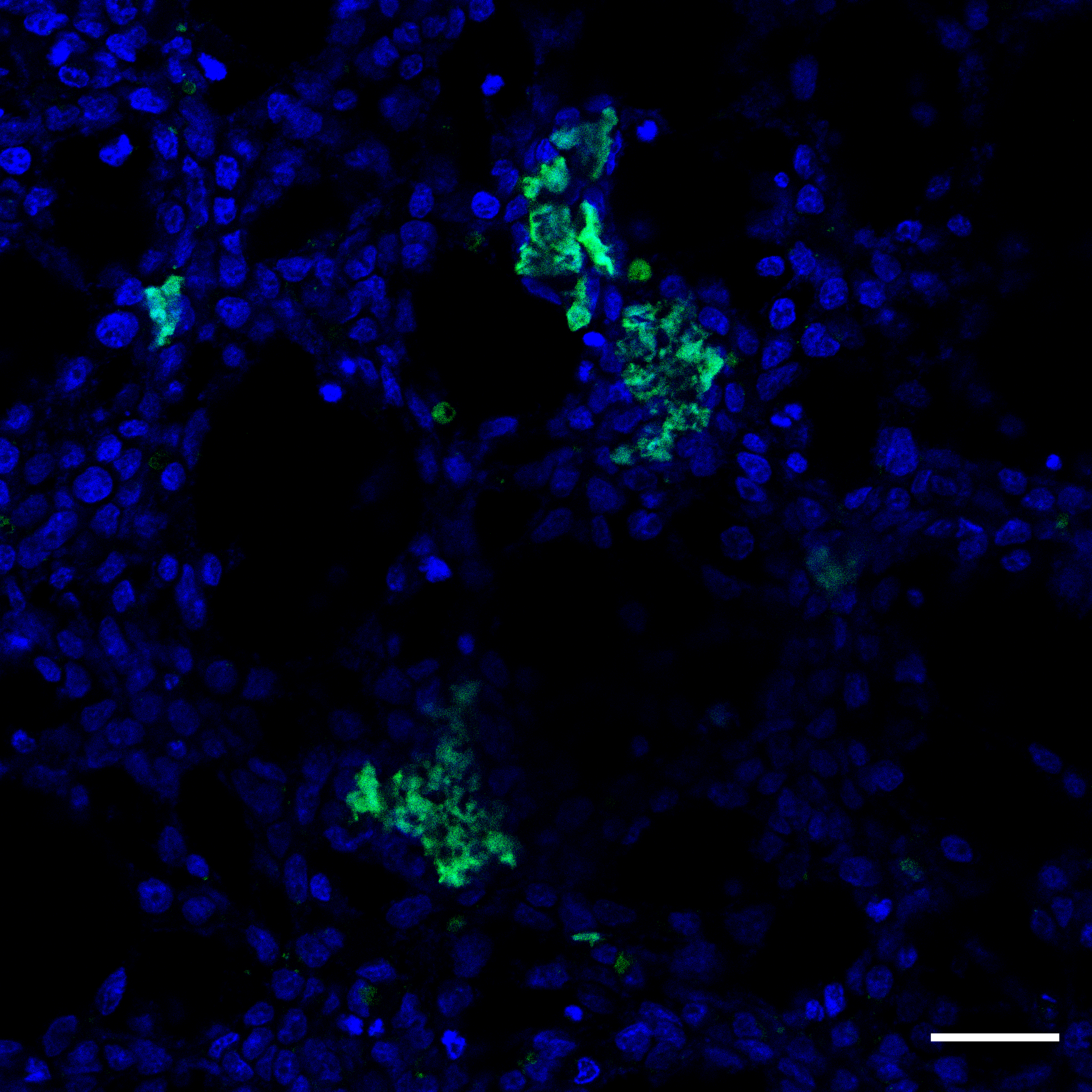

Supplement: Supplementary file 12 — Source data Fig. 8 [file 44318_2024_281_MOESM12_ESM.zip › Figure8/8E/GFP 5 CRD1.tif]

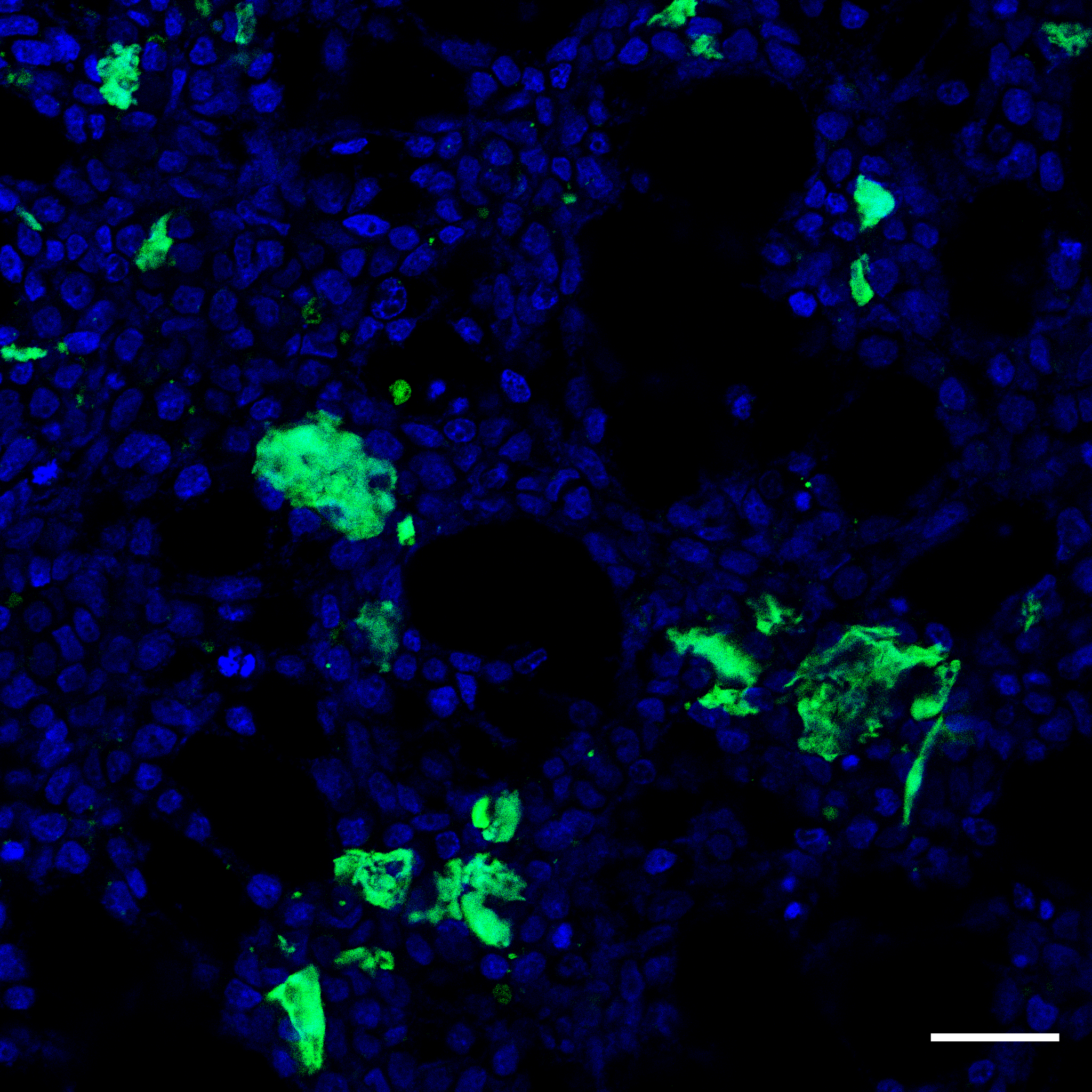

Supplement: Supplementary file 12 — Source data Fig. 8 [file 44318_2024_281_MOESM12_ESM.zip › Figure8/8E/GFP control.tif]

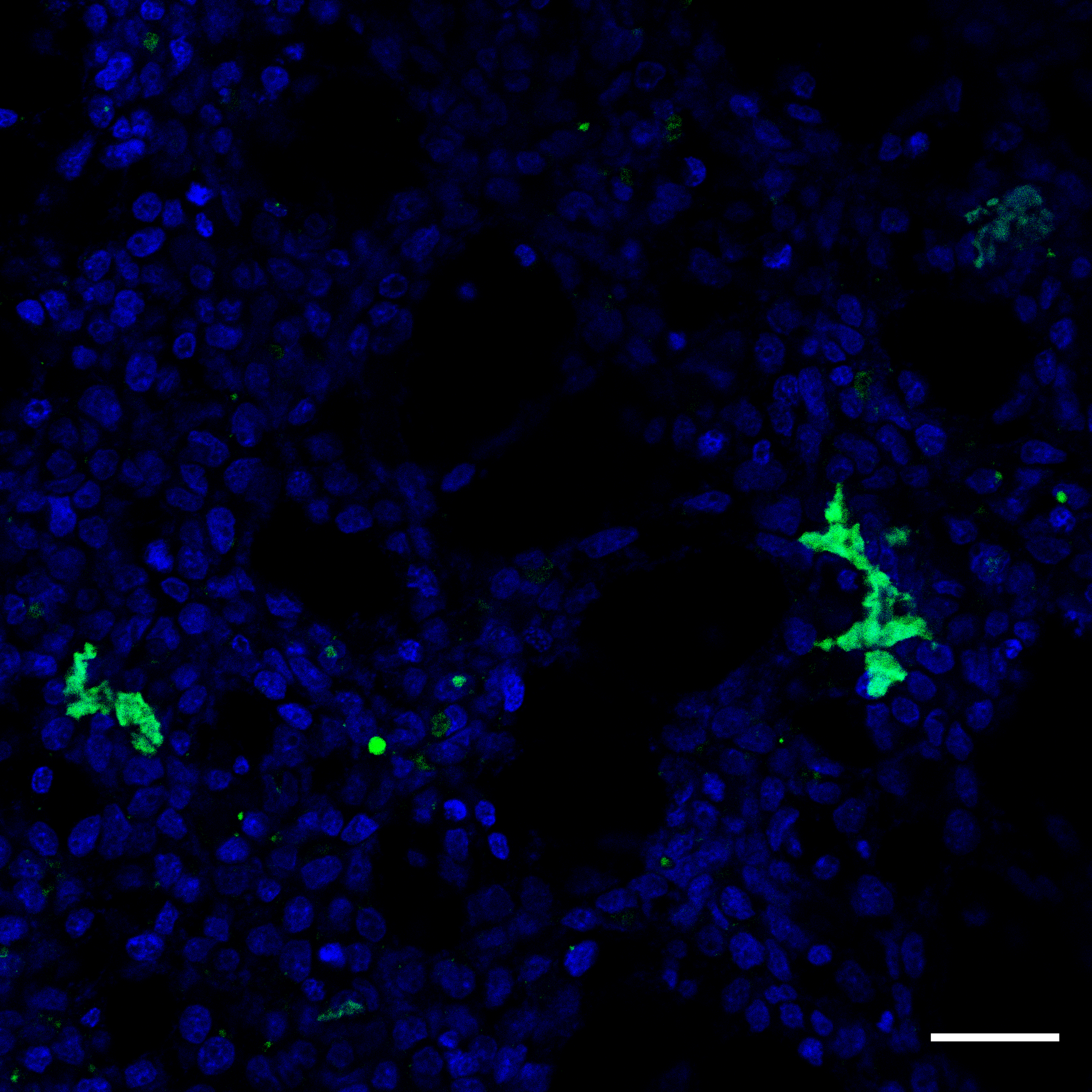

Supplement: Supplementary file 12 — Source data Fig. 8 [file 44318_2024_281_MOESM12_ESM.zip › Figure8/8F/GFP 10 CRD1.tif]

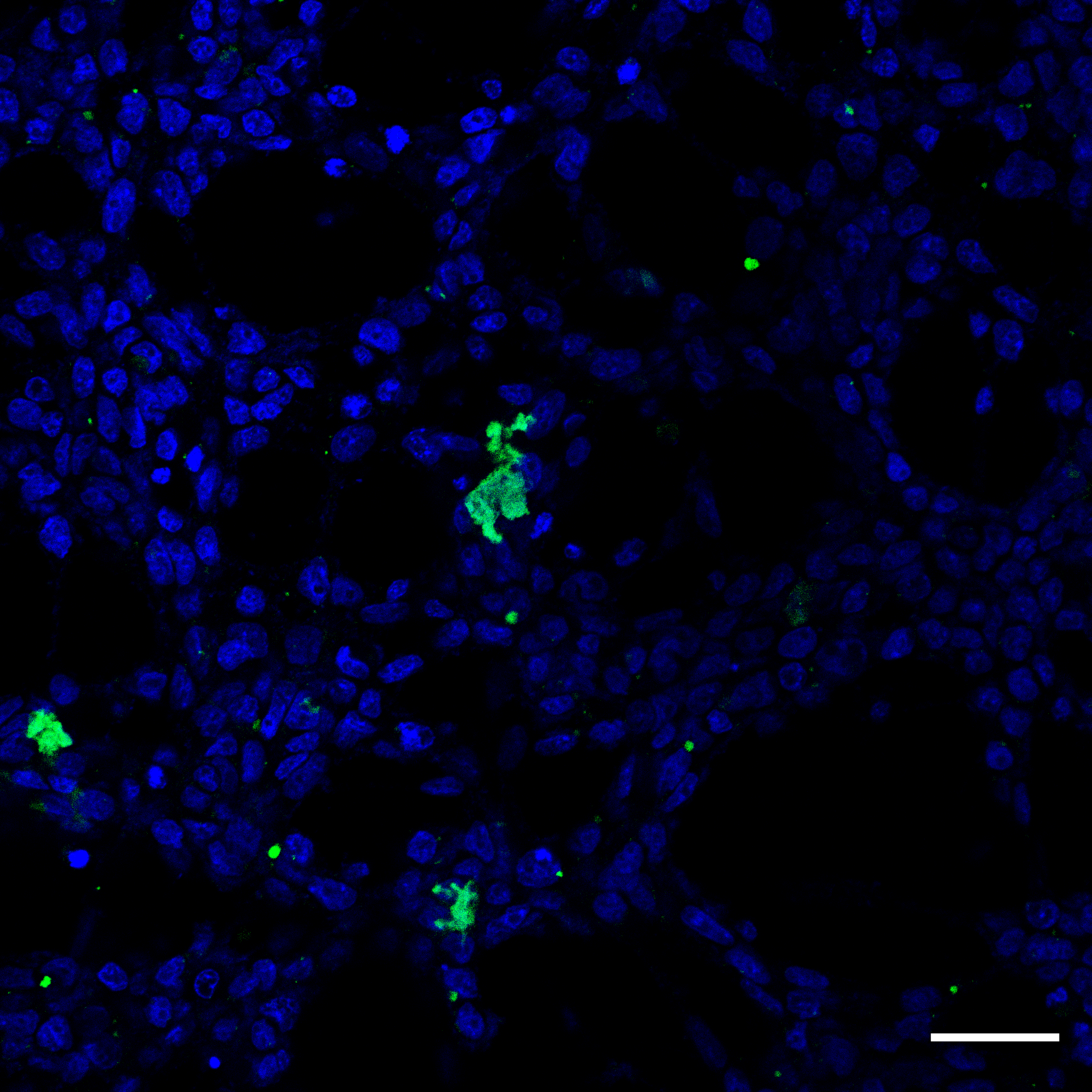

Supplement: Supplementary file 12 — Source data Fig. 8 [file 44318_2024_281_MOESM12_ESM.zip › Figure8/8F/GFP 20 CRD1.tif]

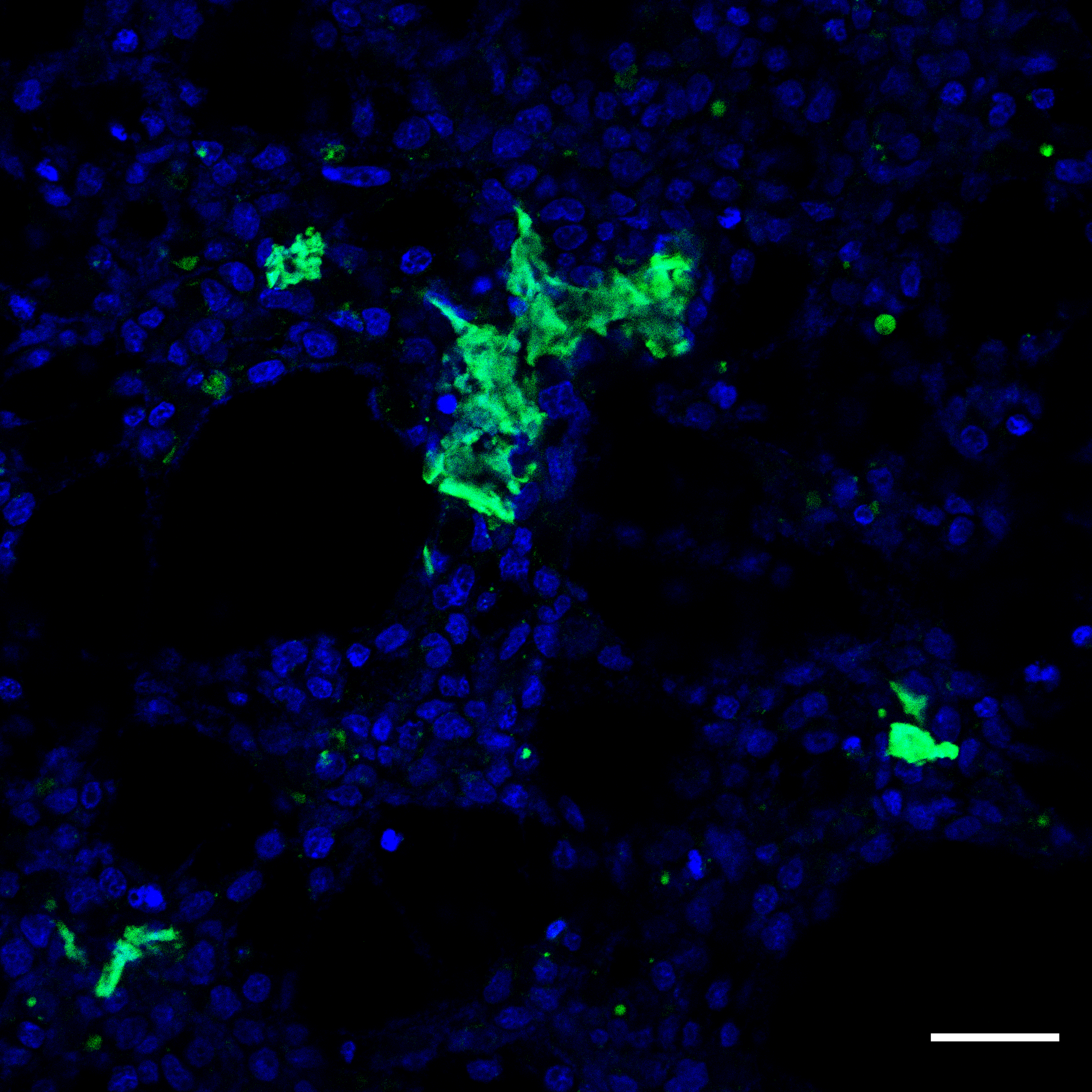

Supplement: Supplementary file 12 — Source data Fig. 8 [file 44318_2024_281_MOESM12_ESM.zip › Figure8/8F/GFP 5 CRD1.tif]

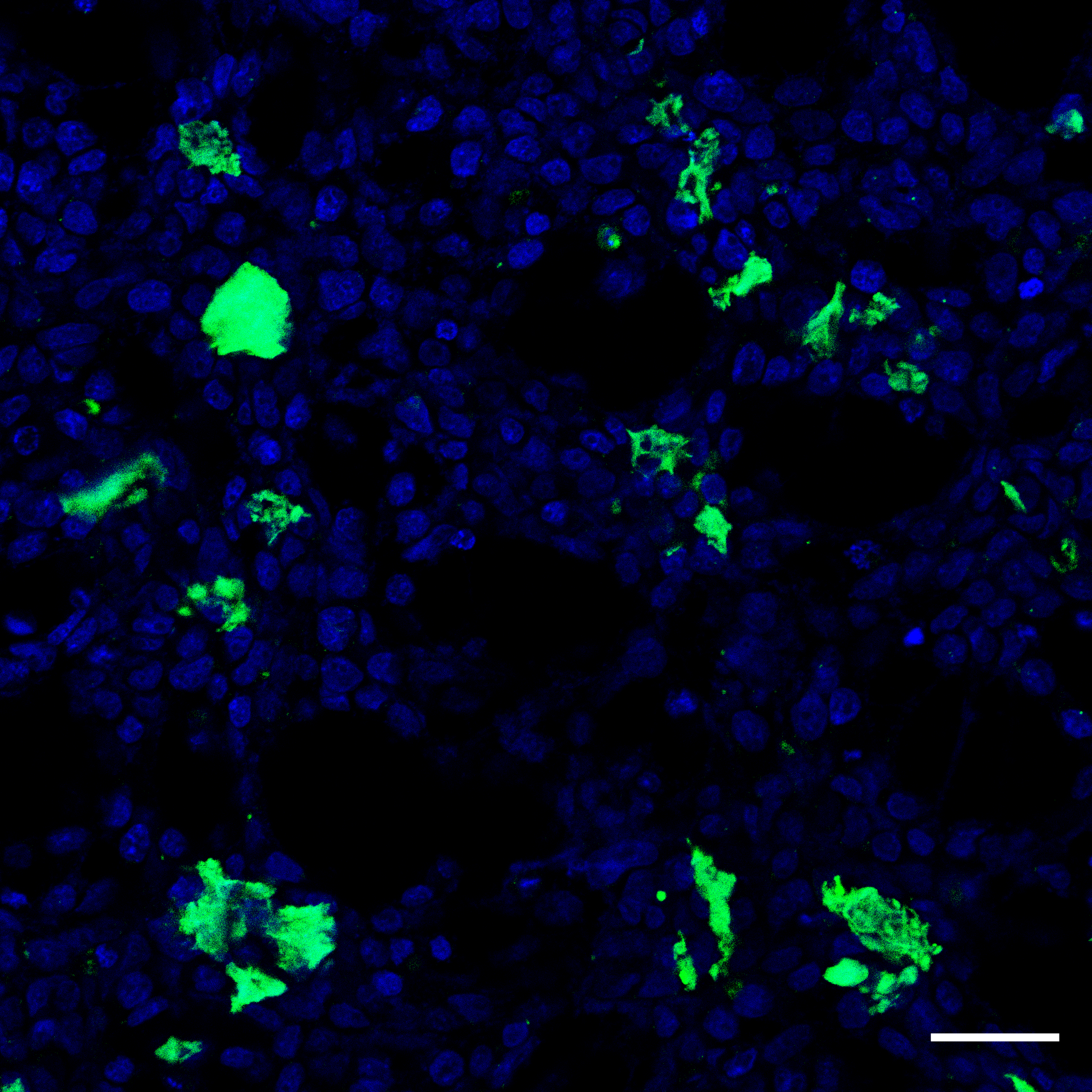

Supplement: Supplementary file 12 — Source data Fig. 8 [file 44318_2024_281_MOESM12_ESM.zip › Figure8/8F/GFP control.tif]

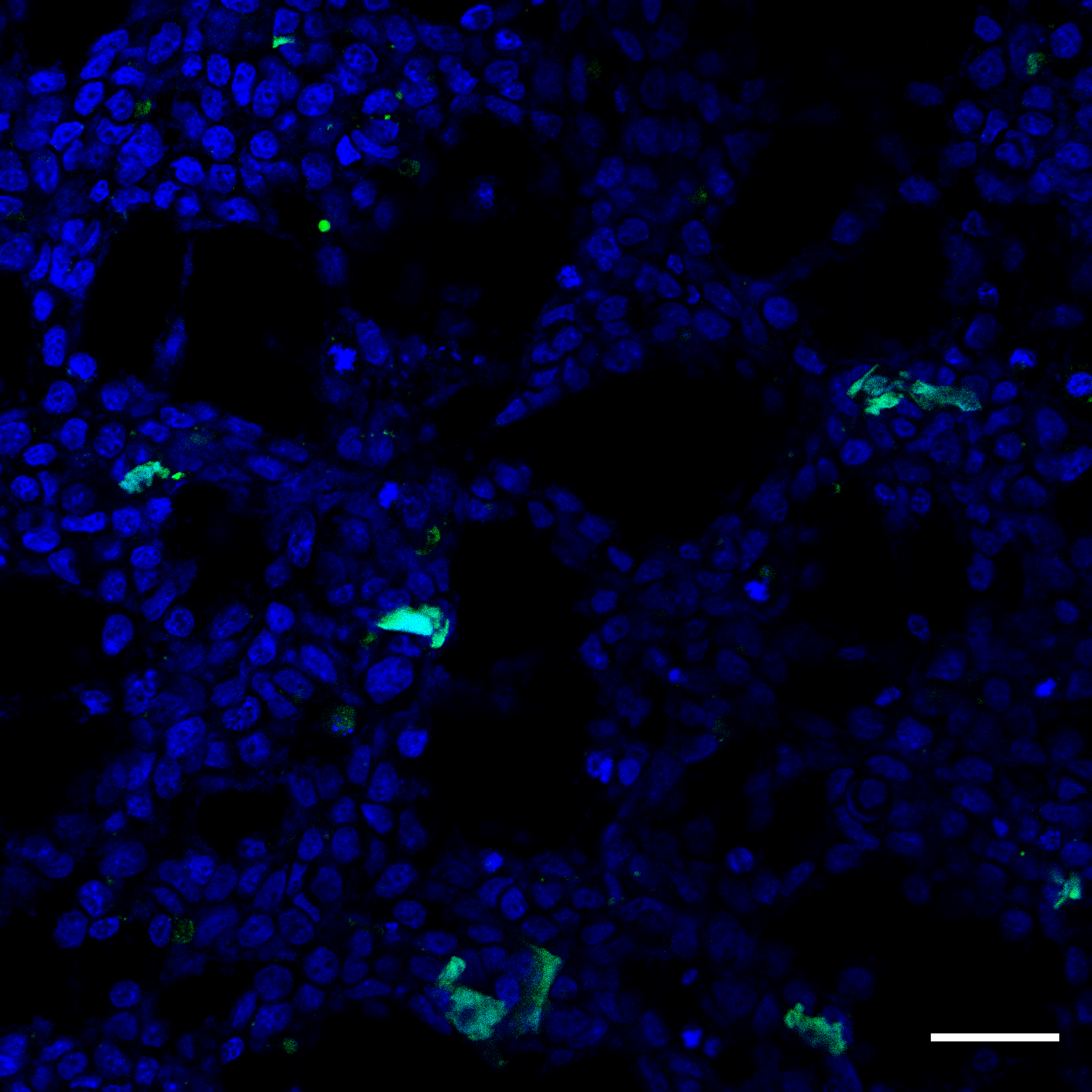

Supplement: Supplementary file 12 — Source data Fig. 8 [file 44318_2024_281_MOESM12_ESM.zip › Figure8/8G/GFP ACE2 LSR+Spike.tif]

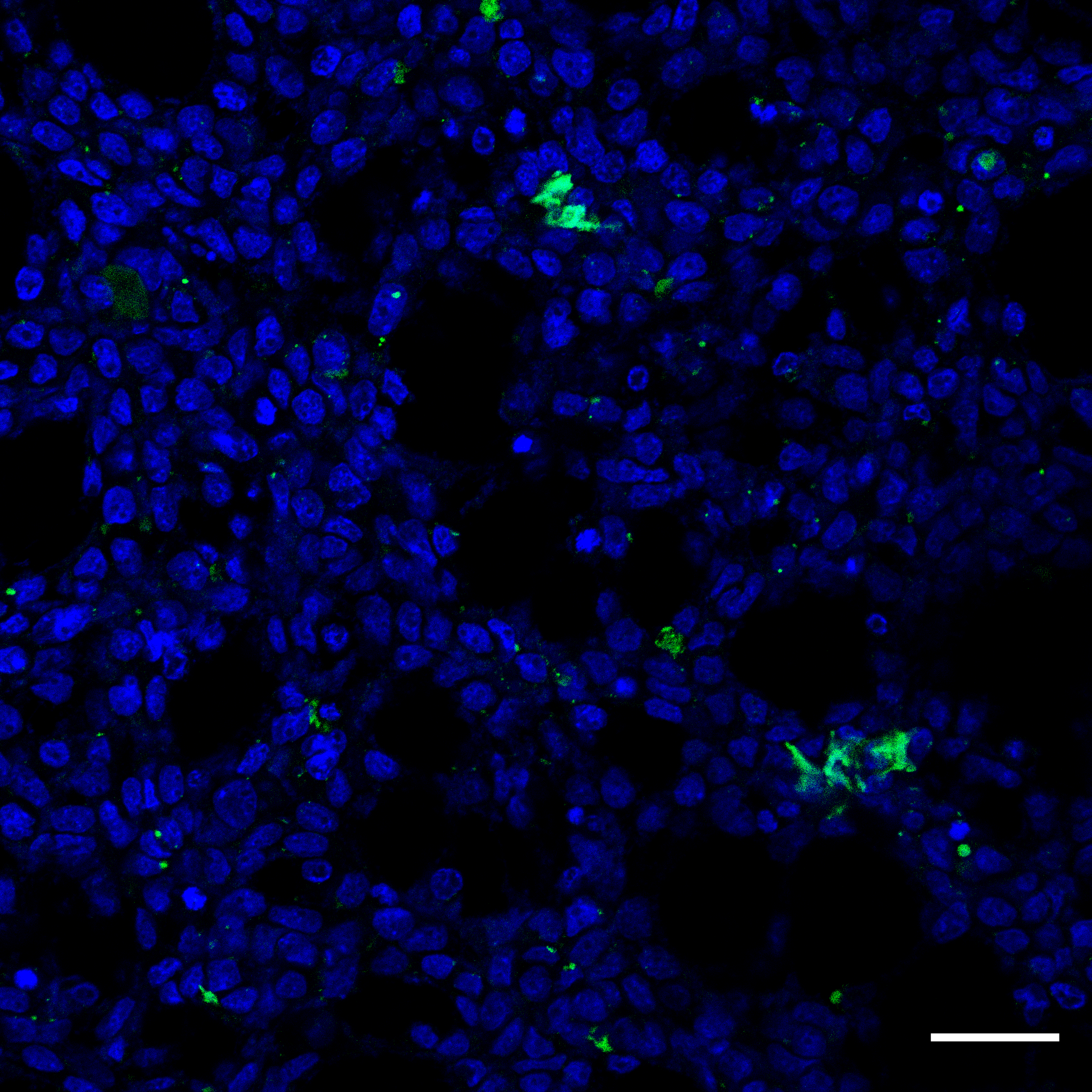

Supplement: Supplementary file 12 — Source data Fig. 8 [file 44318_2024_281_MOESM12_ESM.zip › Figure8/8G/GFP ACE2+Spike LSR.tif]

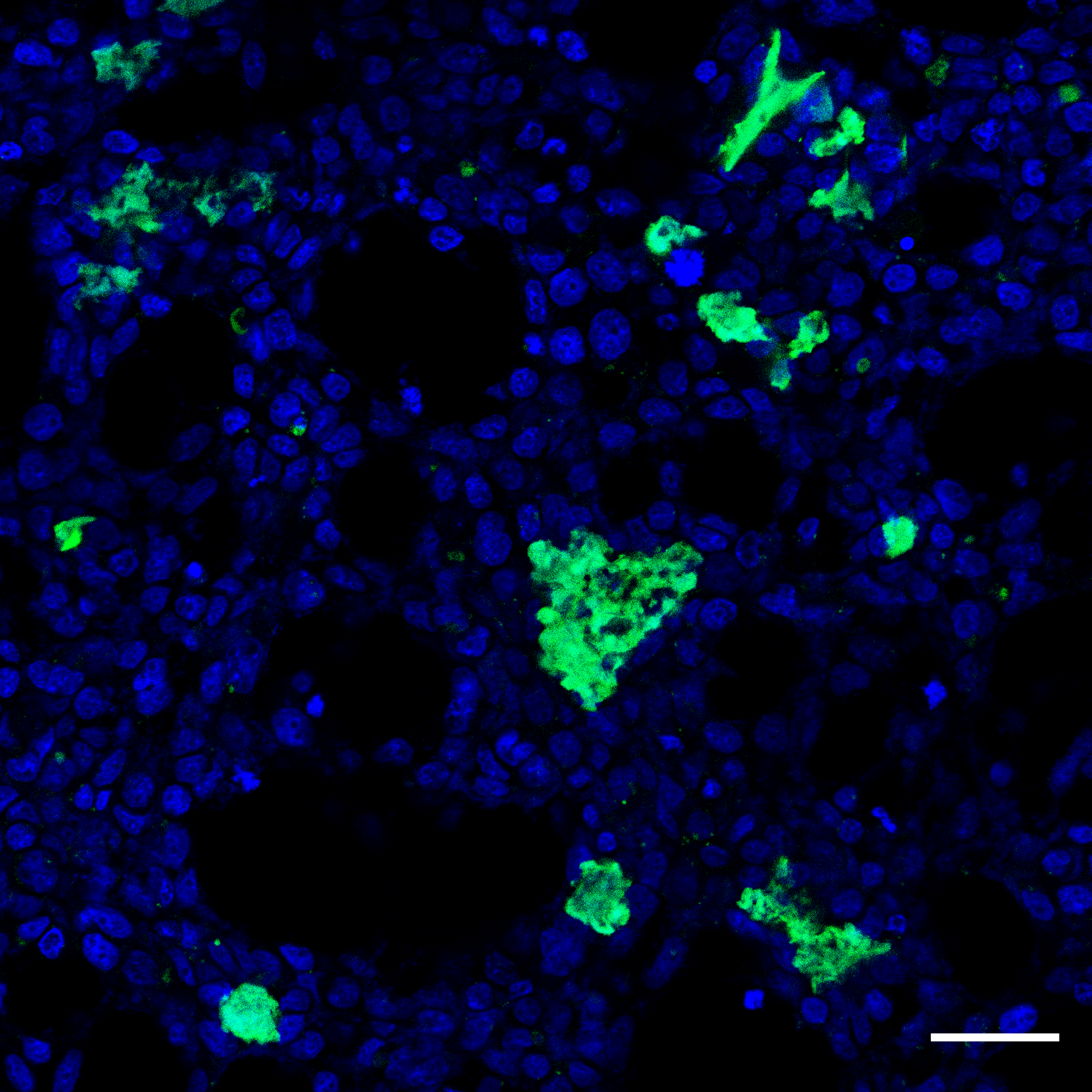

Supplement: Supplementary file 12 — Source data Fig. 8 [file 44318_2024_281_MOESM12_ESM.zip › Figure8/8G/GFP ACE2+Spike.tif]

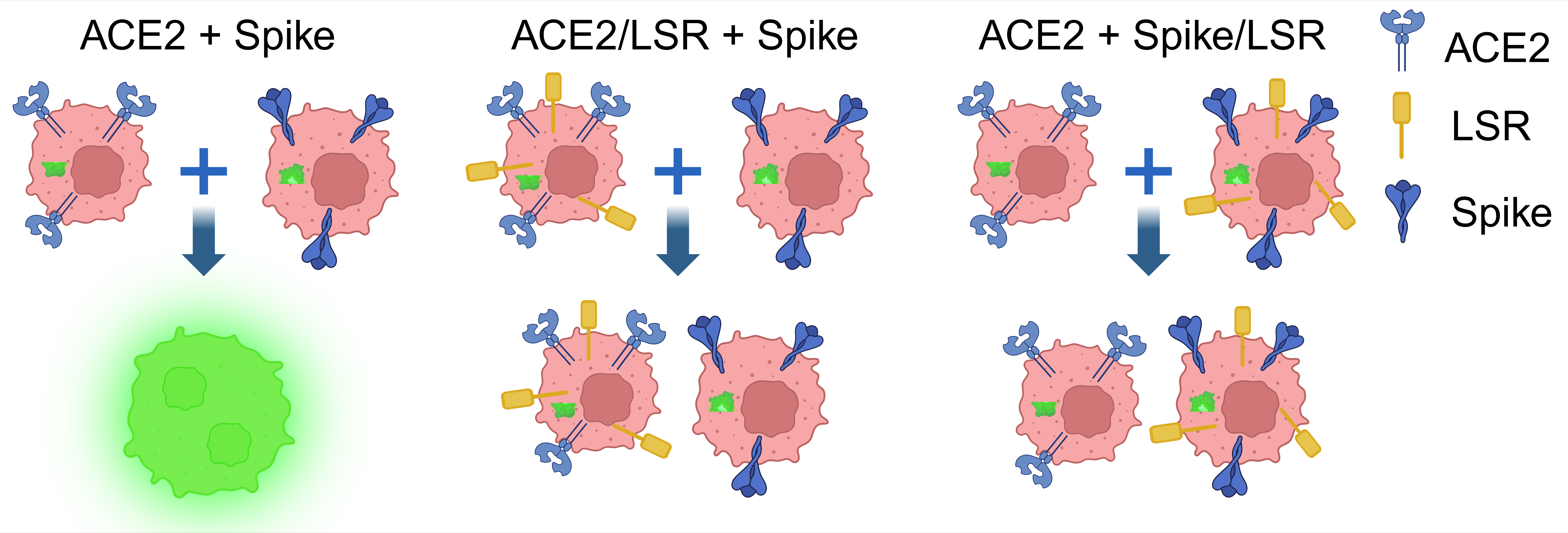

Supplement: Supplementary file 12 — Source data Fig. 8 [file 44318_2024_281_MOESM12_ESM.zip › Figure8/8G/The schematic principle of theImpact of LSR on syncytia formation on Spike- or ACE2-expressing HEK293 cells.jpeg]

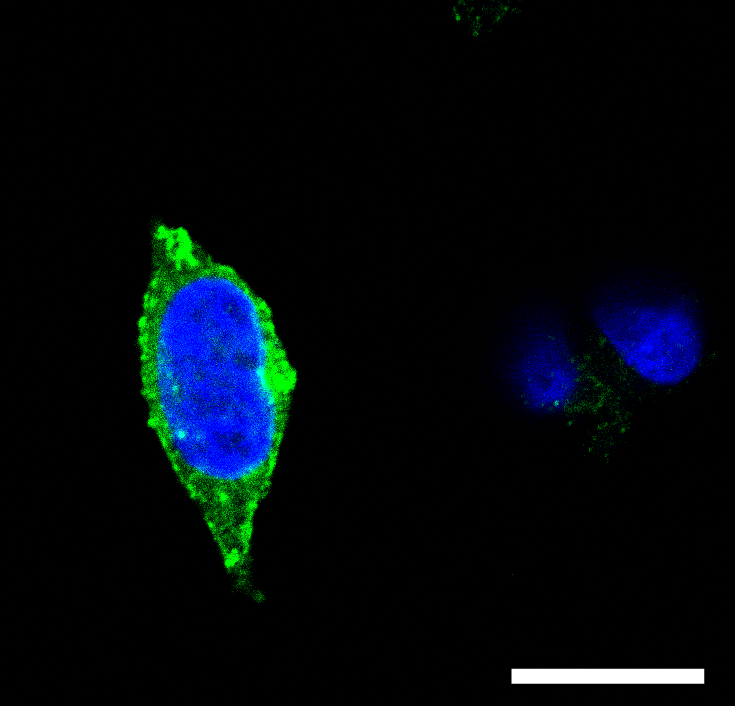

Supplement: Supplementary file 12 — Source data Fig. 8 [file 44318_2024_281_MOESM12_ESM.zip › Figure8/8H/IF ACE2.tif]

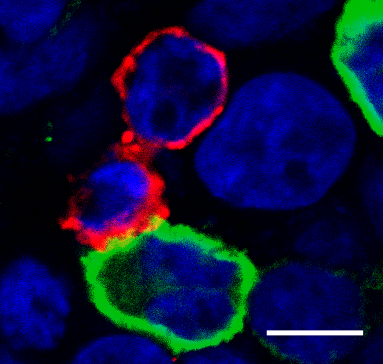

Supplement: Supplementary file 12 — Source data Fig. 8 [file 44318_2024_281_MOESM12_ESM.zip › Figure8/8H/IF Interaction.tif]

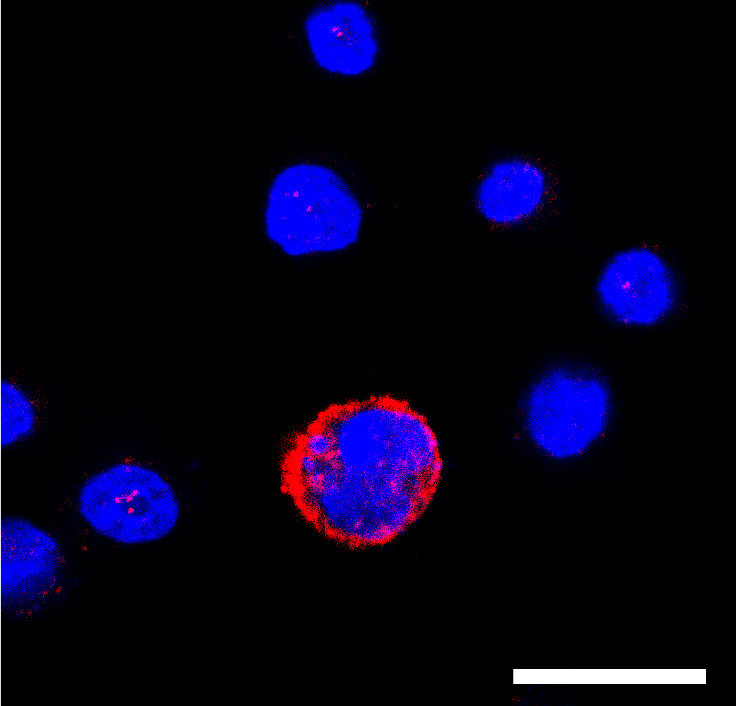

Supplement: Supplementary file 12 — Source data Fig. 8 [file 44318_2024_281_MOESM12_ESM.zip › Figure8/8H/IF LSR.tif]

kDa

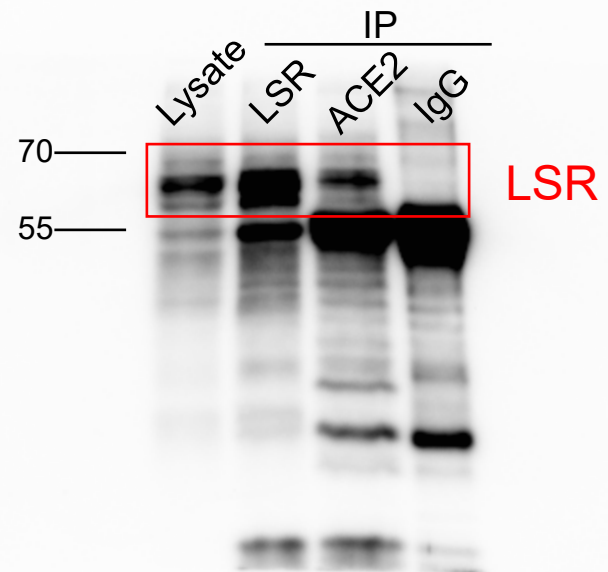

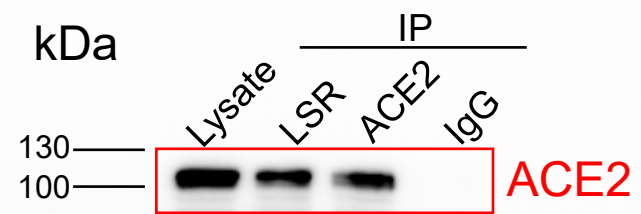

Supplement: Supplementary file 12 — Source data Fig. 8 [file 44318_2024_281_MOESM12_ESM.zip › Figure8/8I/8I.pdf]

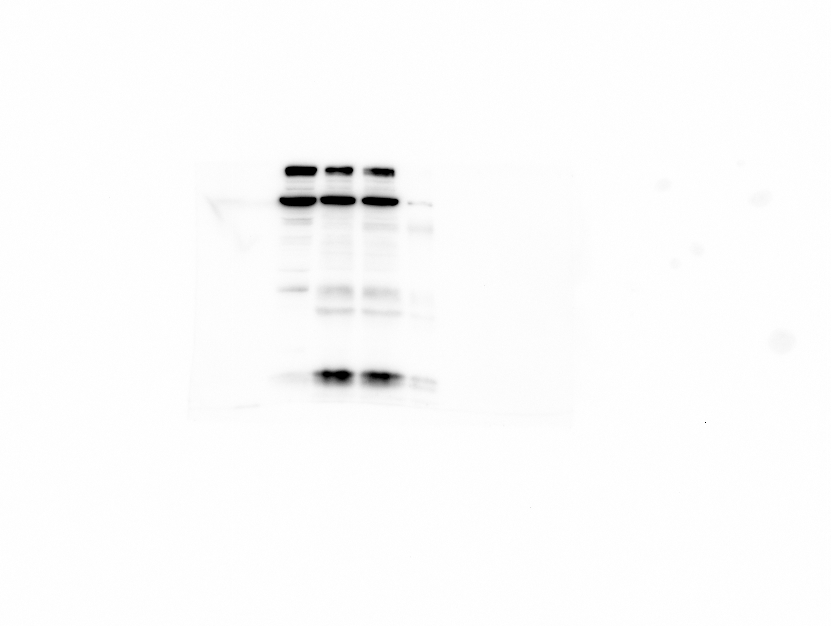

Supplement: Supplementary file 12 — Source data Fig. 8 [file 44318_2024_281_MOESM12_ESM.zip › Figure8/8I/western ACE2.png]

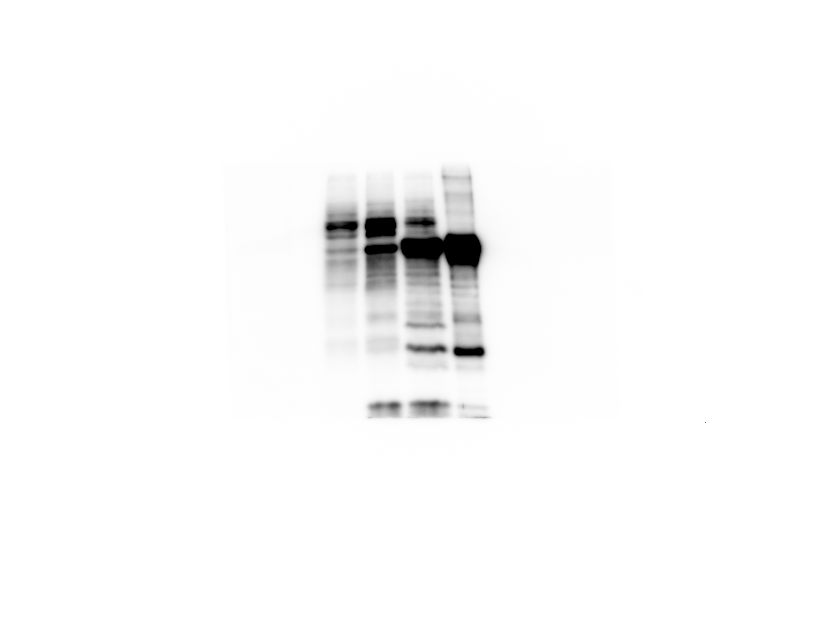

Supplement: Supplementary file 12 — Source data Fig. 8 [file 44318_2024_281_MOESM12_ESM.zip › Figure8/8I/western LSR.png]

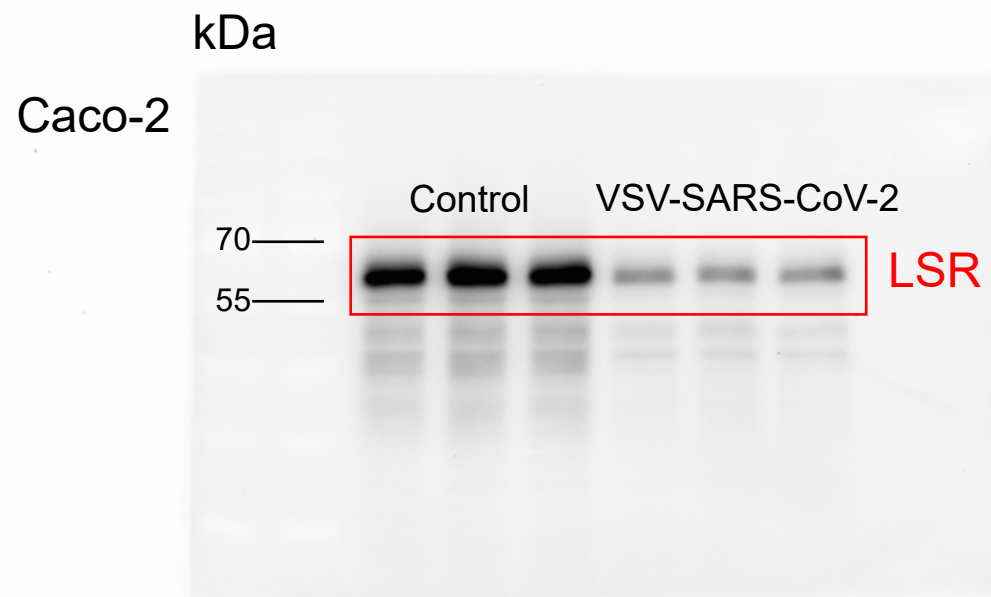

kDa

Caco-2

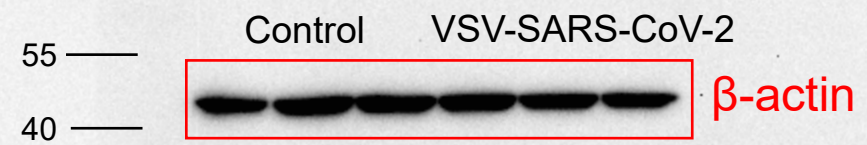

kDa

hACE2-HIEC-6

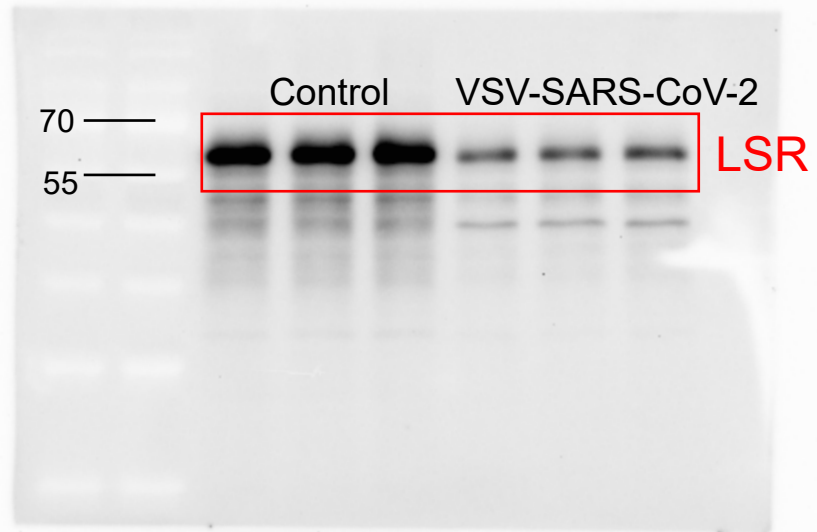

kDa

hACE2-HIEC-6

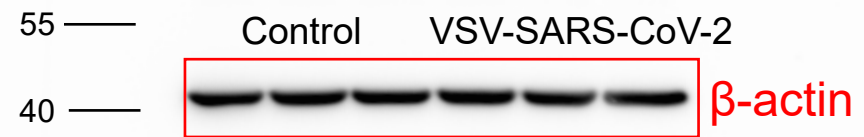

Supplement: Supplementary file 13 — Figure EV1-4 Source Data [file 44318_2024_281_MOESM13_ESM.zip › Figure EV1/EV1A/EV1A.pdf]

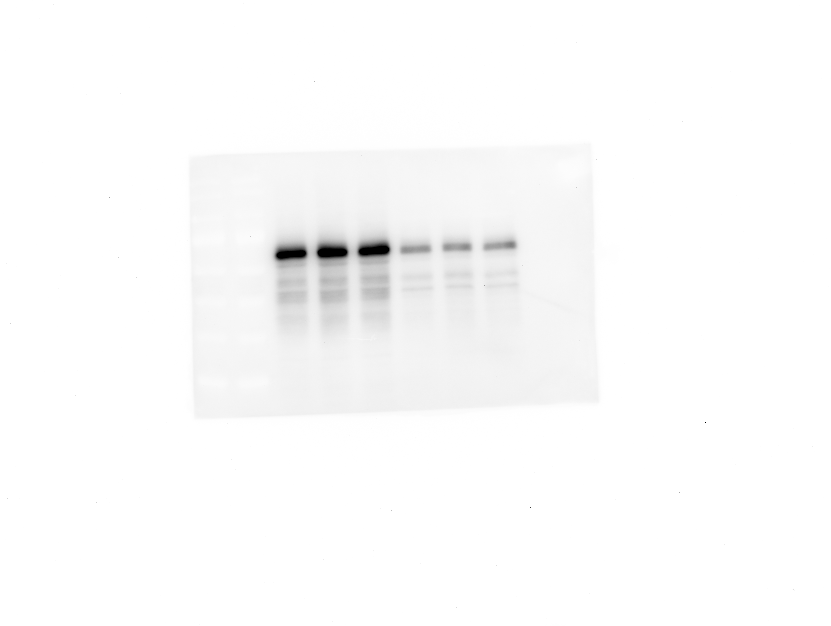

Supplement: Supplementary file 13 — Figure EV1-4 Source Data [file 44318_2024_281_MOESM13_ESM.zip › Figure EV1/EV1A/western LSR Caco-2.png]

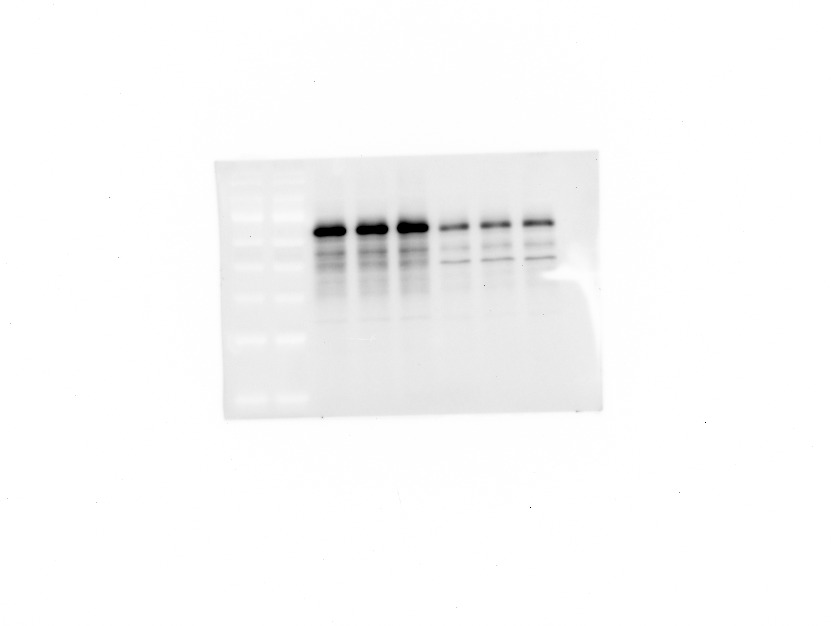

Supplement: Supplementary file 13 — Figure EV1-4 Source Data [file 44318_2024_281_MOESM13_ESM.zip › Figure EV1/EV1A/western LSR hACE2-HIEC-6.png]

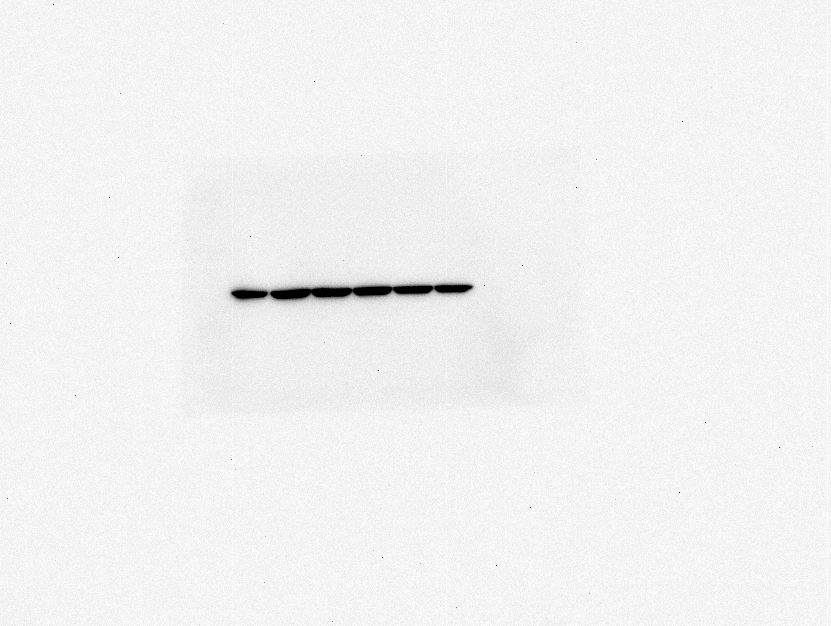

Supplement: Supplementary file 13 — Figure EV1-4 Source Data [file 44318_2024_281_MOESM13_ESM.zip › Figure EV1/EV1A/western actin Caco-2.png]

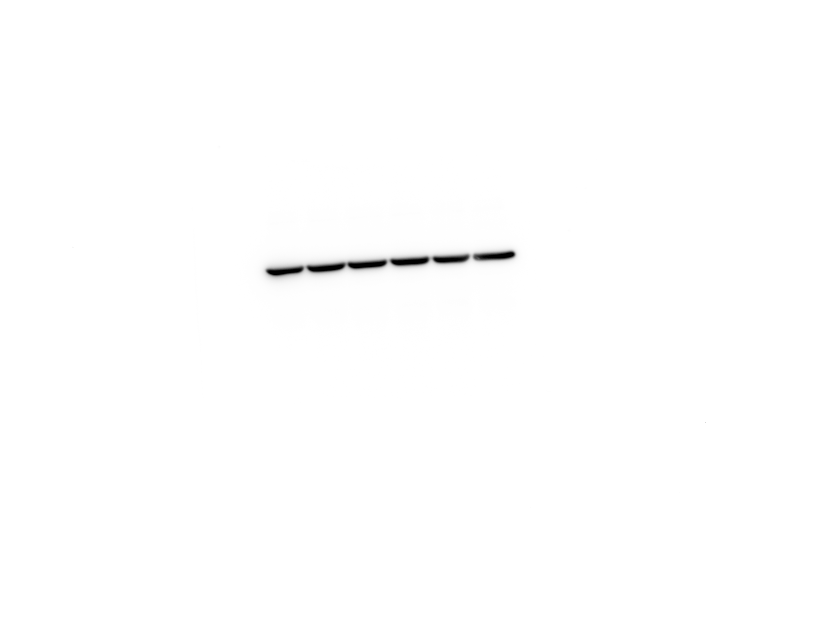

Supplement: Supplementary file 13 — Figure EV1-4 Source Data [file 44318_2024_281_MOESM13_ESM.zip › Figure EV1/EV1A/western actin hACE2-HIEC-6.png]

kDa

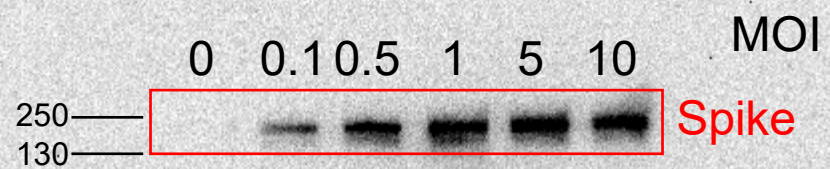

kDa

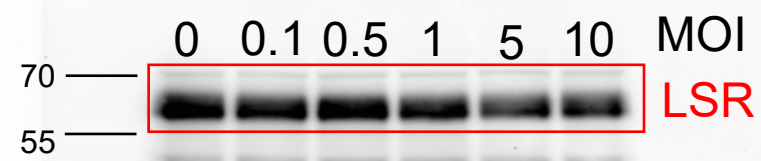

kDa

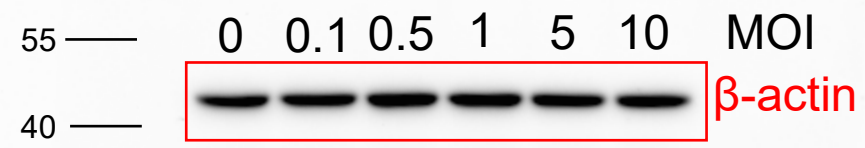

Supplement: Supplementary file 13 — Figure EV1-4 Source Data [file 44318_2024_281_MOESM13_ESM.zip › Figure EV1/EV1B/EV1B.pdf]

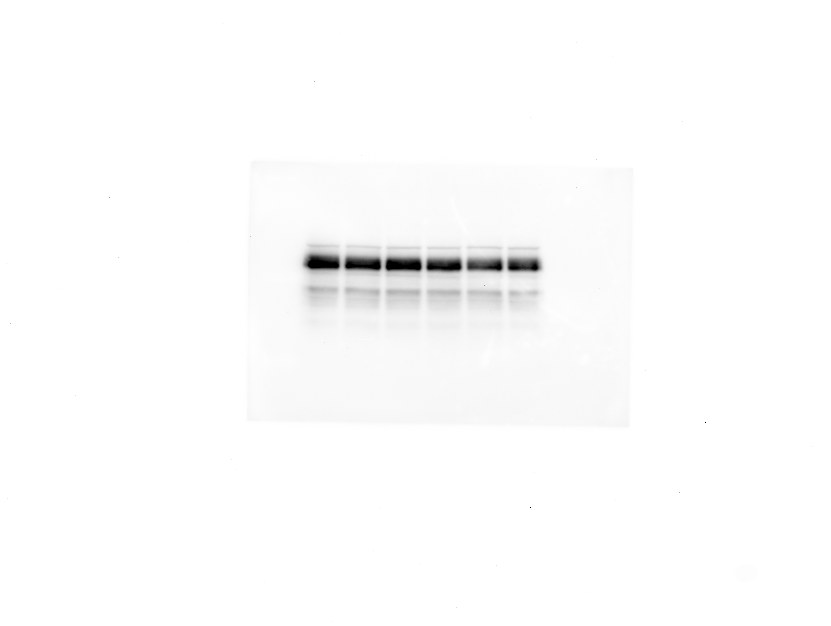

Supplement: Supplementary file 13 — Figure EV1-4 Source Data [file 44318_2024_281_MOESM13_ESM.zip › Figure EV1/EV1B/western LSR.png]

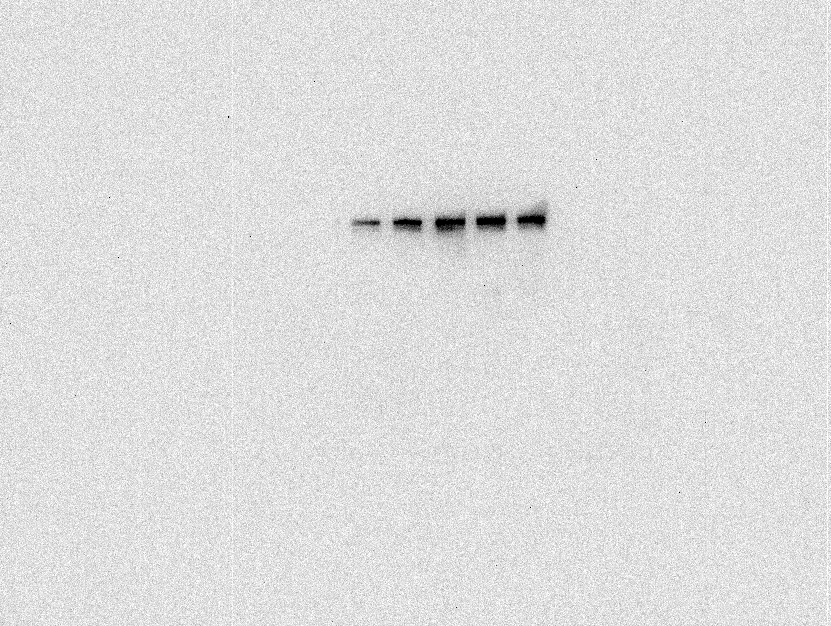

Supplement: Supplementary file 13 — Figure EV1-4 Source Data [file 44318_2024_281_MOESM13_ESM.zip › Figure EV1/EV1B/western Spike.png]

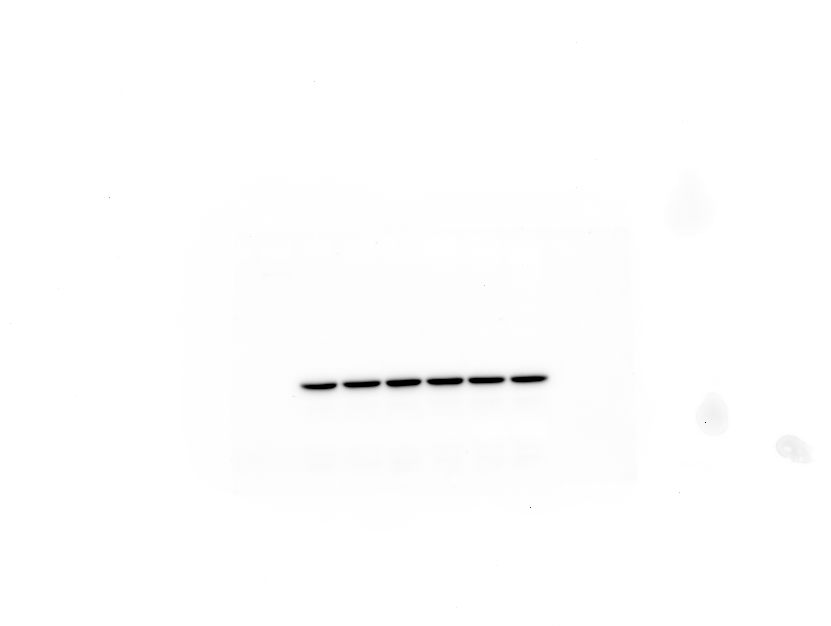

Supplement: Supplementary file 13 — Figure EV1-4 Source Data [file 44318_2024_281_MOESM13_ESM.zip › Figure EV1/EV1B/western actin.png]

kDa

250  
130

0 0.1 0.5 1 5 10 MOI

Spike

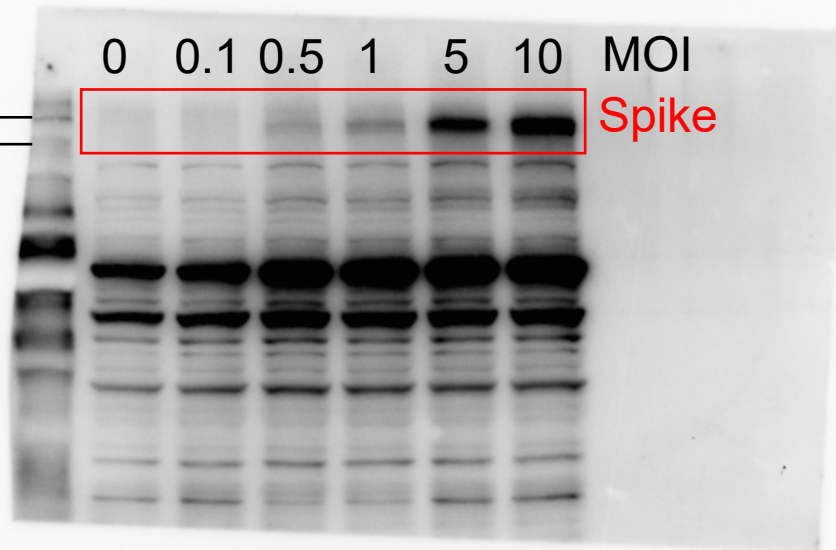

kDa

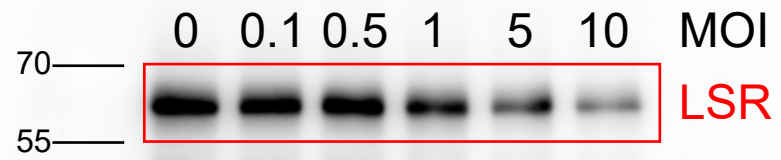

kDa

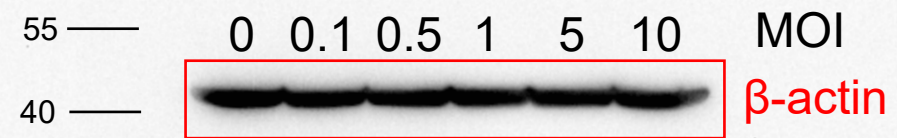

Supplement: Supplementary file 13 — Figure EV1-4 Source Data [file 44318_2024_281_MOESM13_ESM.zip › Figure EV1/EV1D/EV1D.pdf]

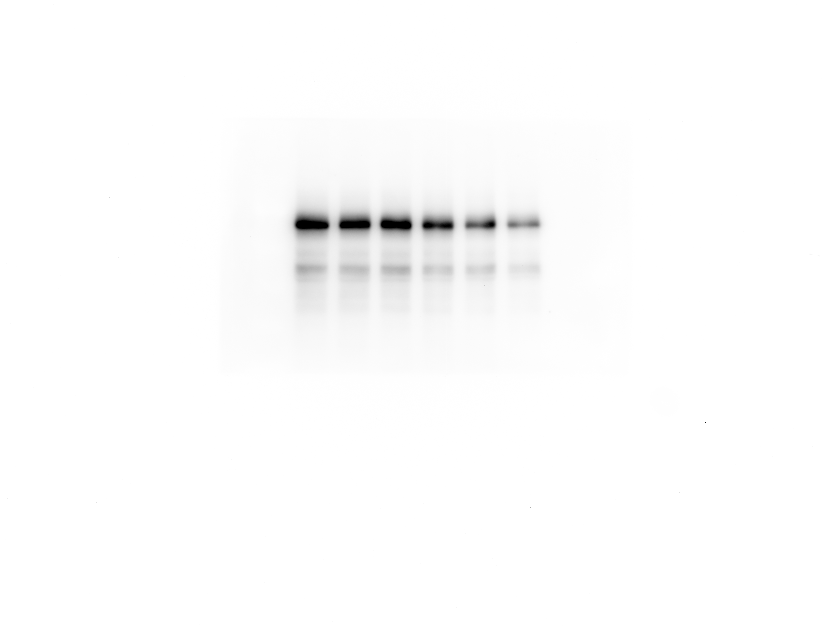

Supplement: Supplementary file 13 — Figure EV1-4 Source Data [file 44318_2024_281_MOESM13_ESM.zip › Figure EV1/EV1D/western LSR.png]

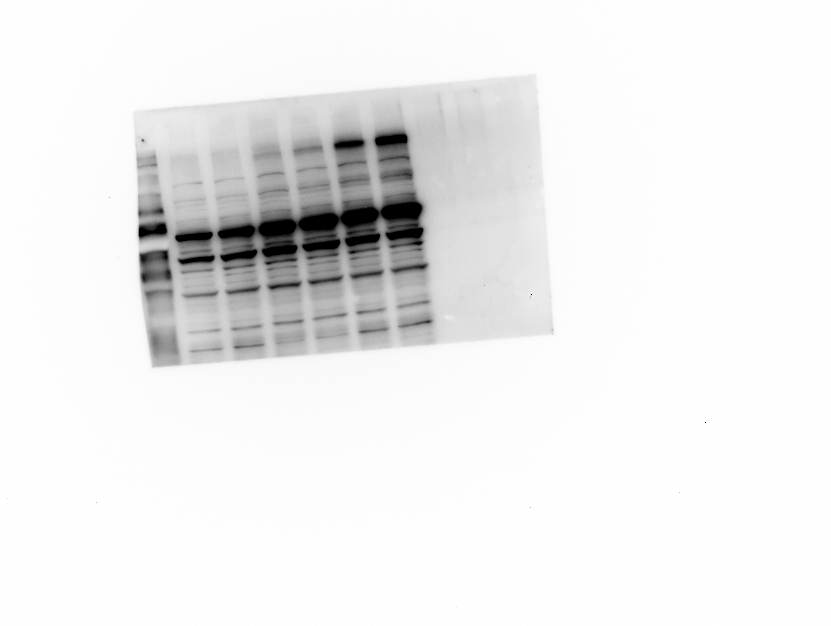

Supplement: Supplementary file 13 — Figure EV1-4 Source Data [file 44318_2024_281_MOESM13_ESM.zip › Figure EV1/EV1D/western Spike.png]

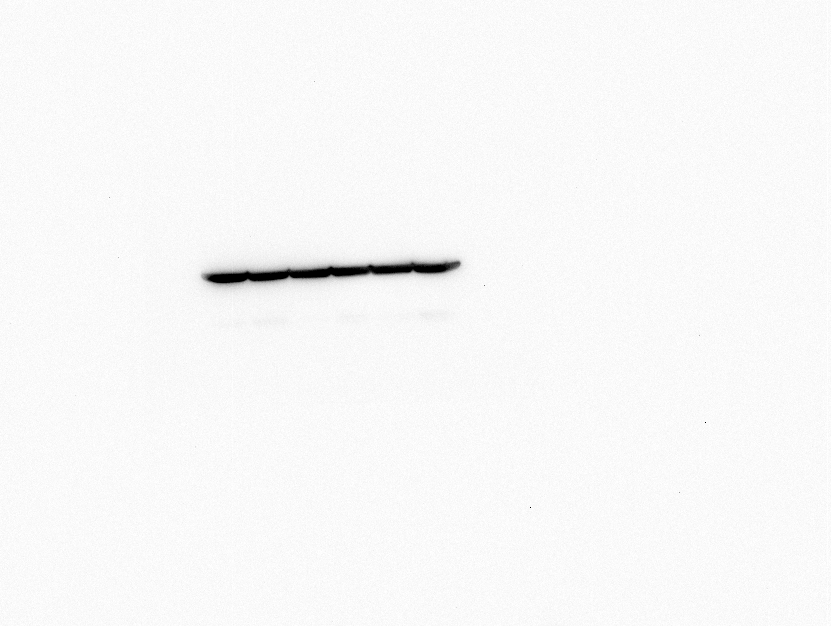

Supplement: Supplementary file 13 — Figure EV1-4 Source Data [file 44318_2024_281_MOESM13_ESM.zip › Figure EV1/EV1D/western actin.png]

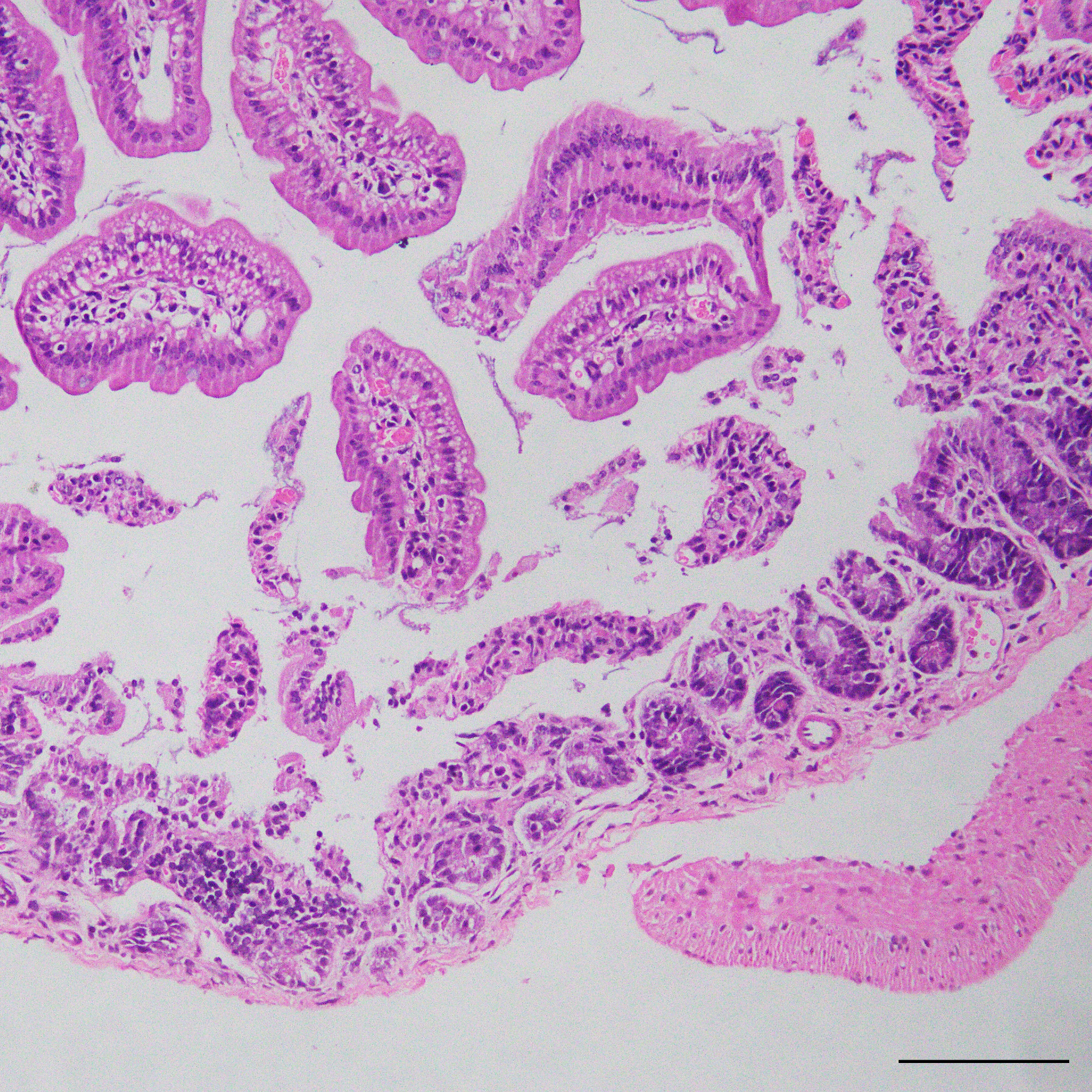

Supplement: Supplementary file 13 — Figure EV1-4 Source Data [file 44318_2024_281_MOESM13_ESM.zip › Figure EV1/EV1F/HE Duodenum VSV-SARS-CoV-2.tif]

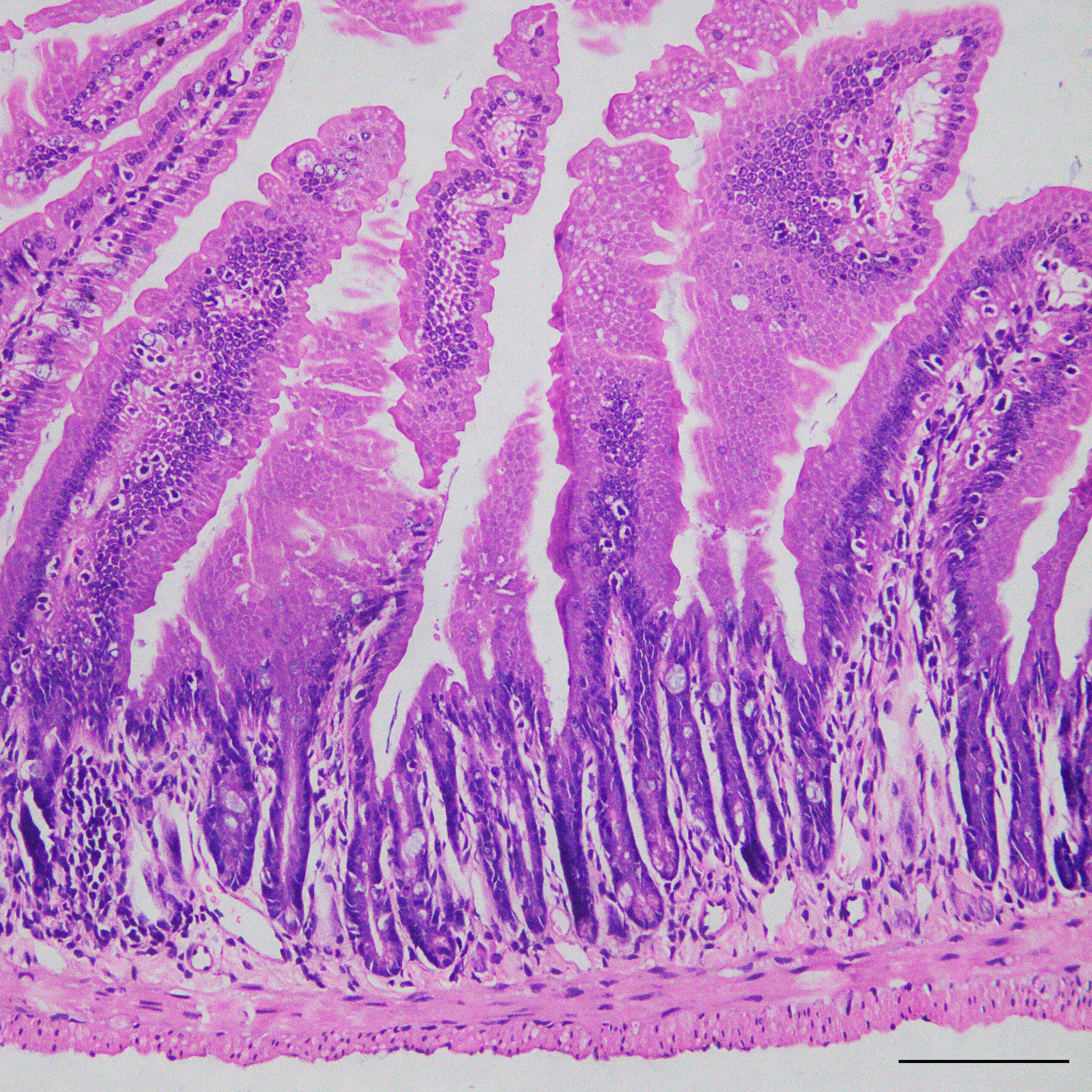

Supplement: Supplementary file 13 — Figure EV1-4 Source Data [file 44318_2024_281_MOESM13_ESM.zip › Figure EV1/EV1F/HE Duodenum control.tif]

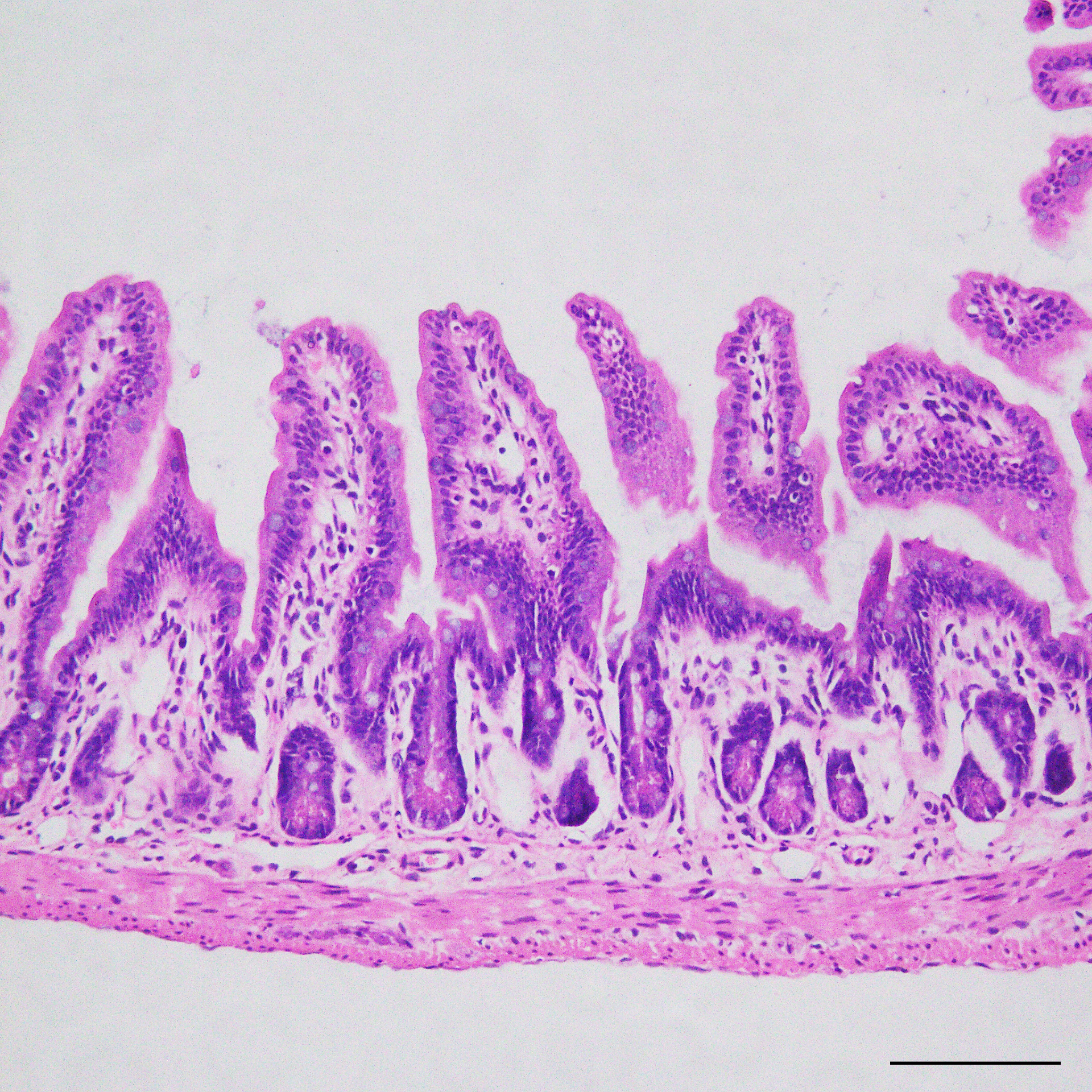

Supplement: Supplementary file 13 — Figure EV1-4 Source Data [file 44318_2024_281_MOESM13_ESM.zip › Figure EV1/EV1F/HE Ileum VSV-SARS-CoV-2.tif]

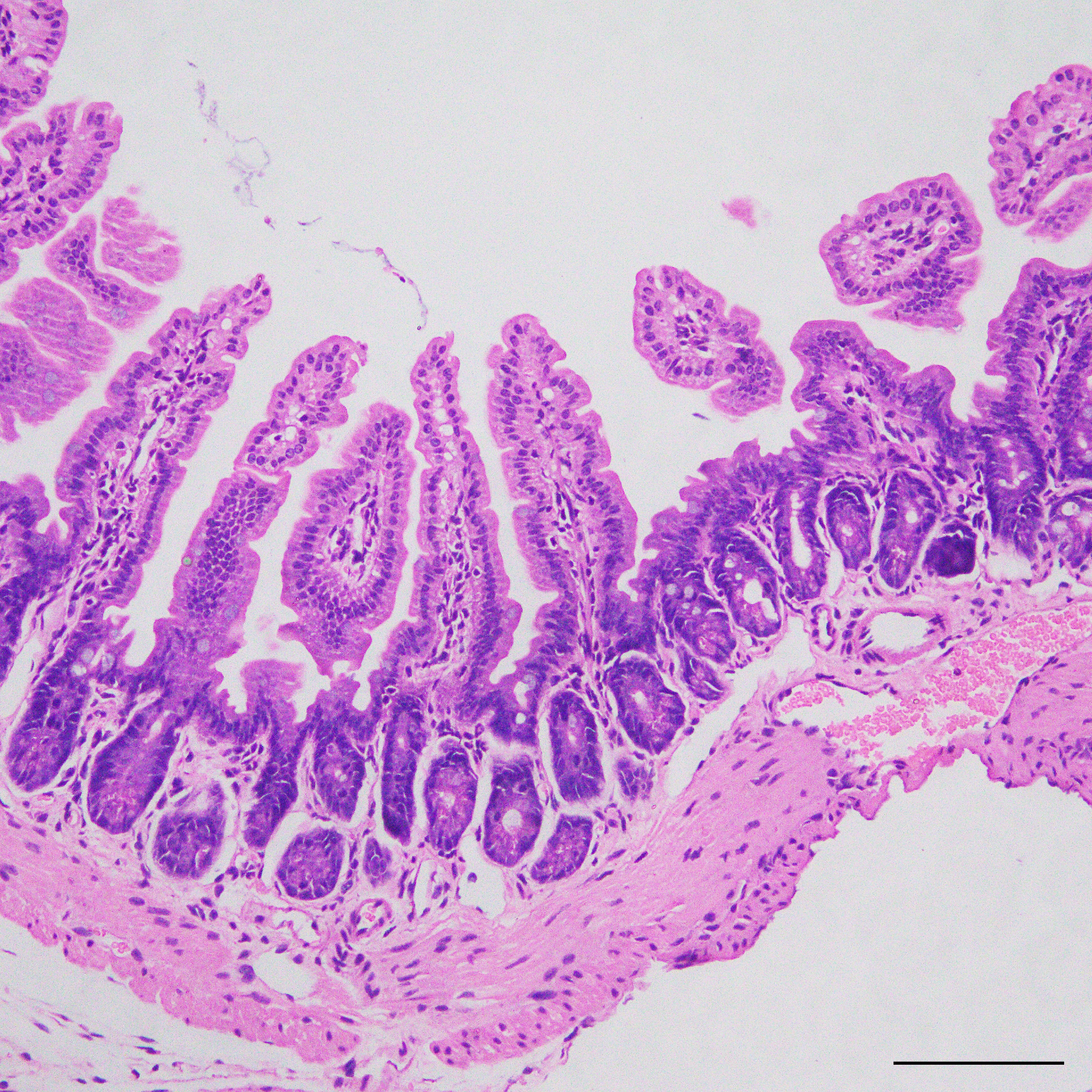

Supplement: Supplementary file 13 — Figure EV1-4 Source Data [file 44318_2024_281_MOESM13_ESM.zip › Figure EV1/EV1F/HE Ileum control.tif]

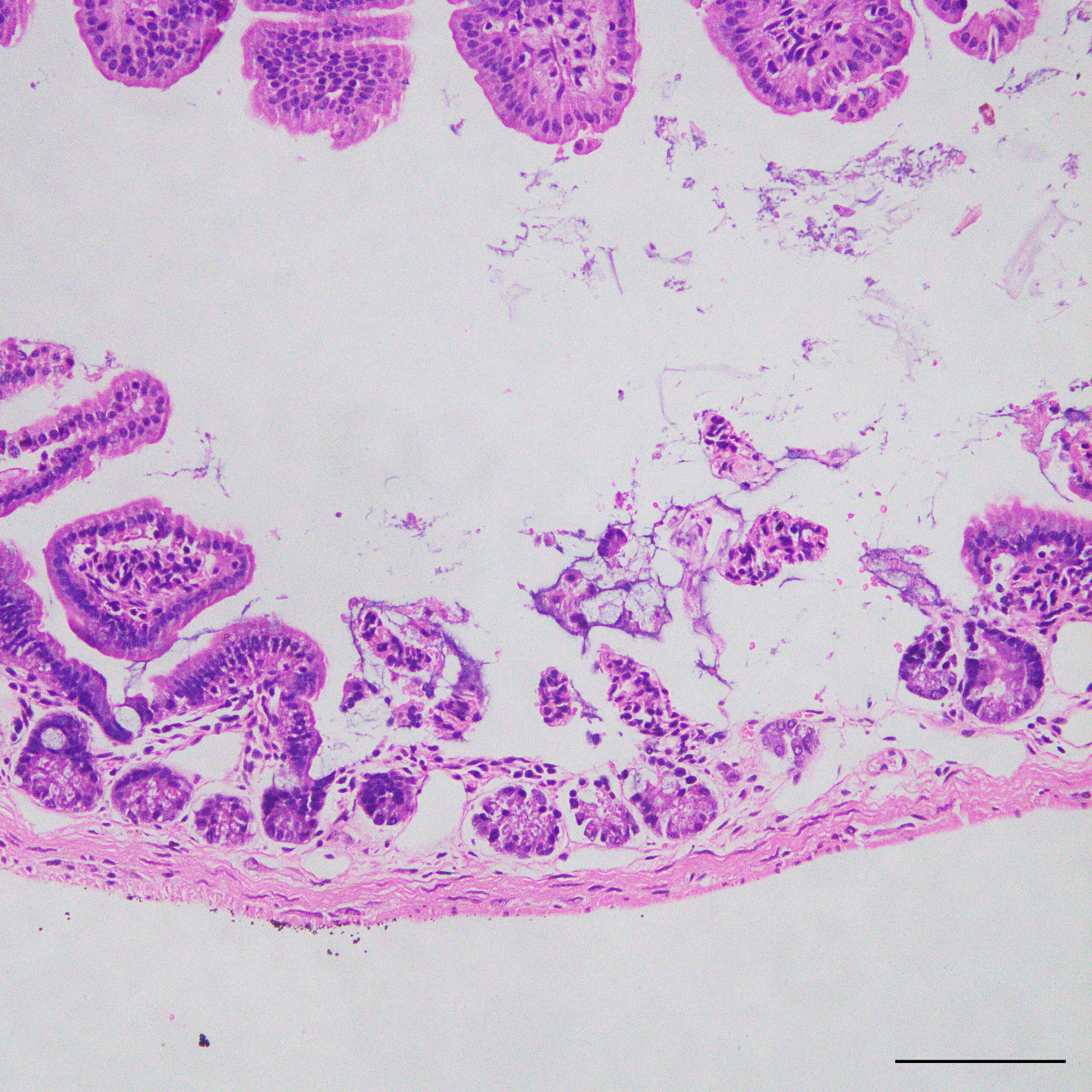

Supplement: Supplementary file 13 — Figure EV1-4 Source Data [file 44318_2024_281_MOESM13_ESM.zip › Figure EV1/EV1F/HE Jejunum VSV-SARS-CoV-2.tif]

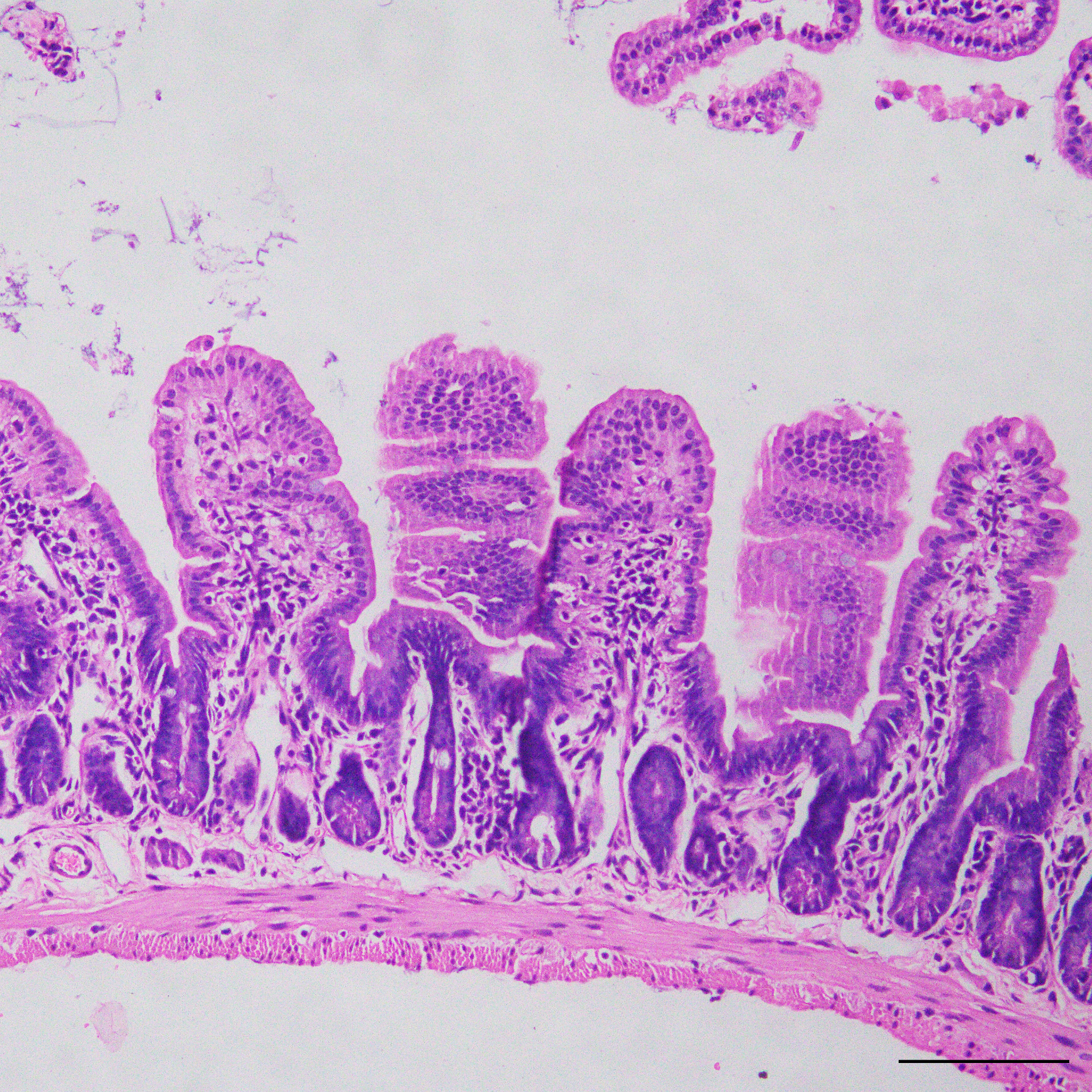

Supplement: Supplementary file 13 — Figure EV1-4 Source Data [file 44318_2024_281_MOESM13_ESM.zip › Figure EV1/EV1F/HE Jejunum control.tif]

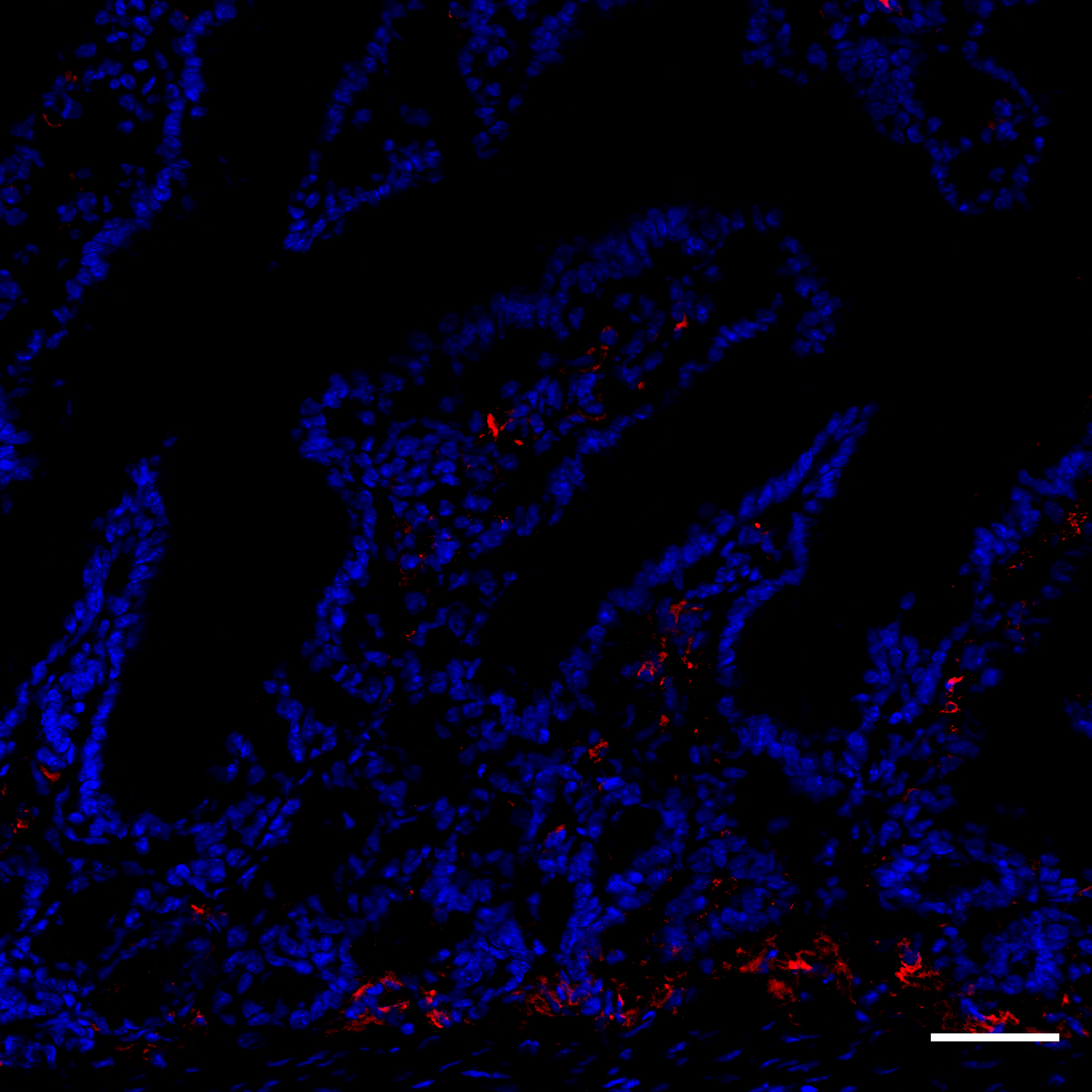

Supplement: Supplementary file 13 — Figure EV1-4 Source Data [file 44318_2024_281_MOESM13_ESM.zip › Figure EV1/EV1J/IF LY6G Duodenum VSV-SARS-CoV-2.tif]

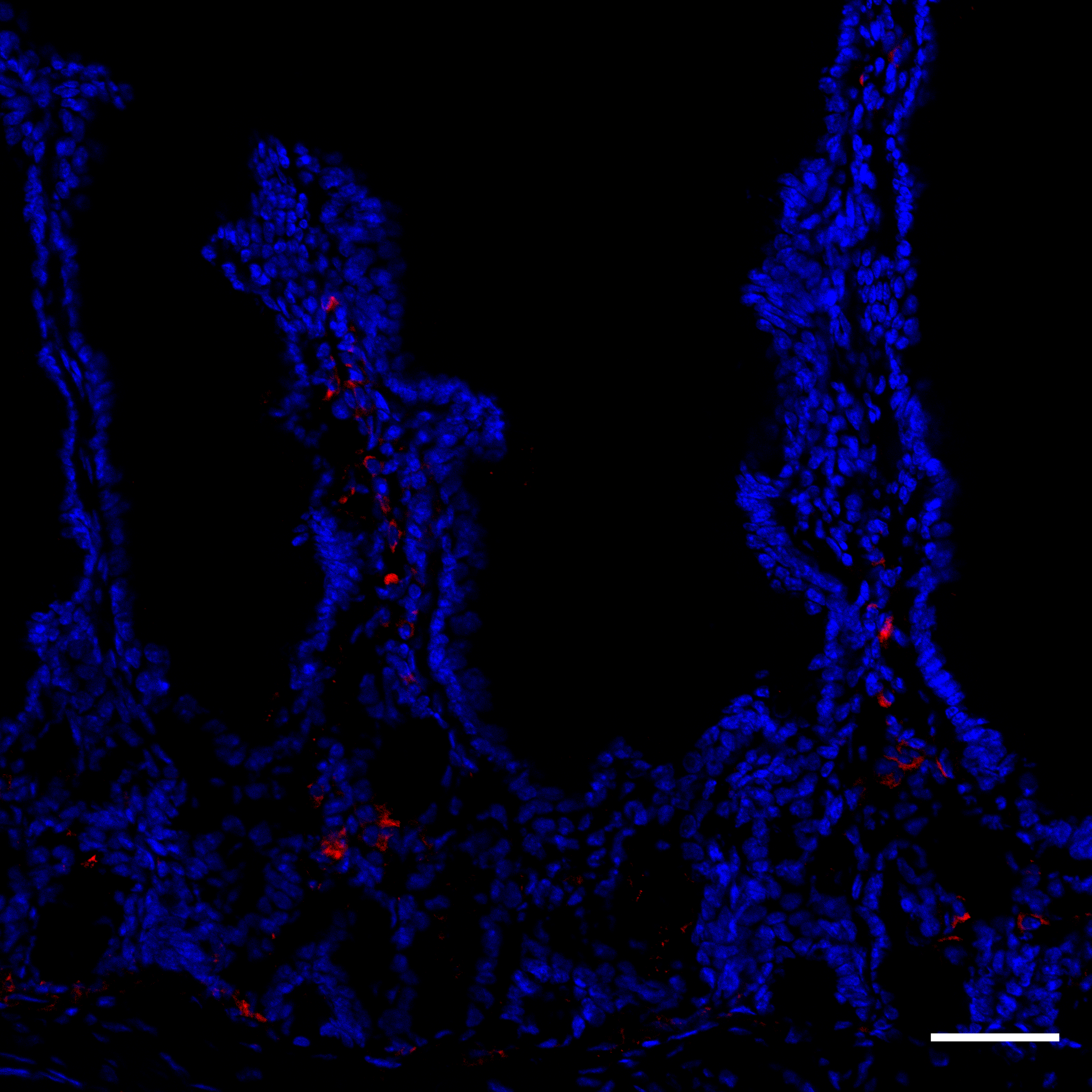

Supplement: Supplementary file 13 — Figure EV1-4 Source Data [file 44318_2024_281_MOESM13_ESM.zip › Figure EV1/EV1J/IF LY6G Duodenum control.tif]

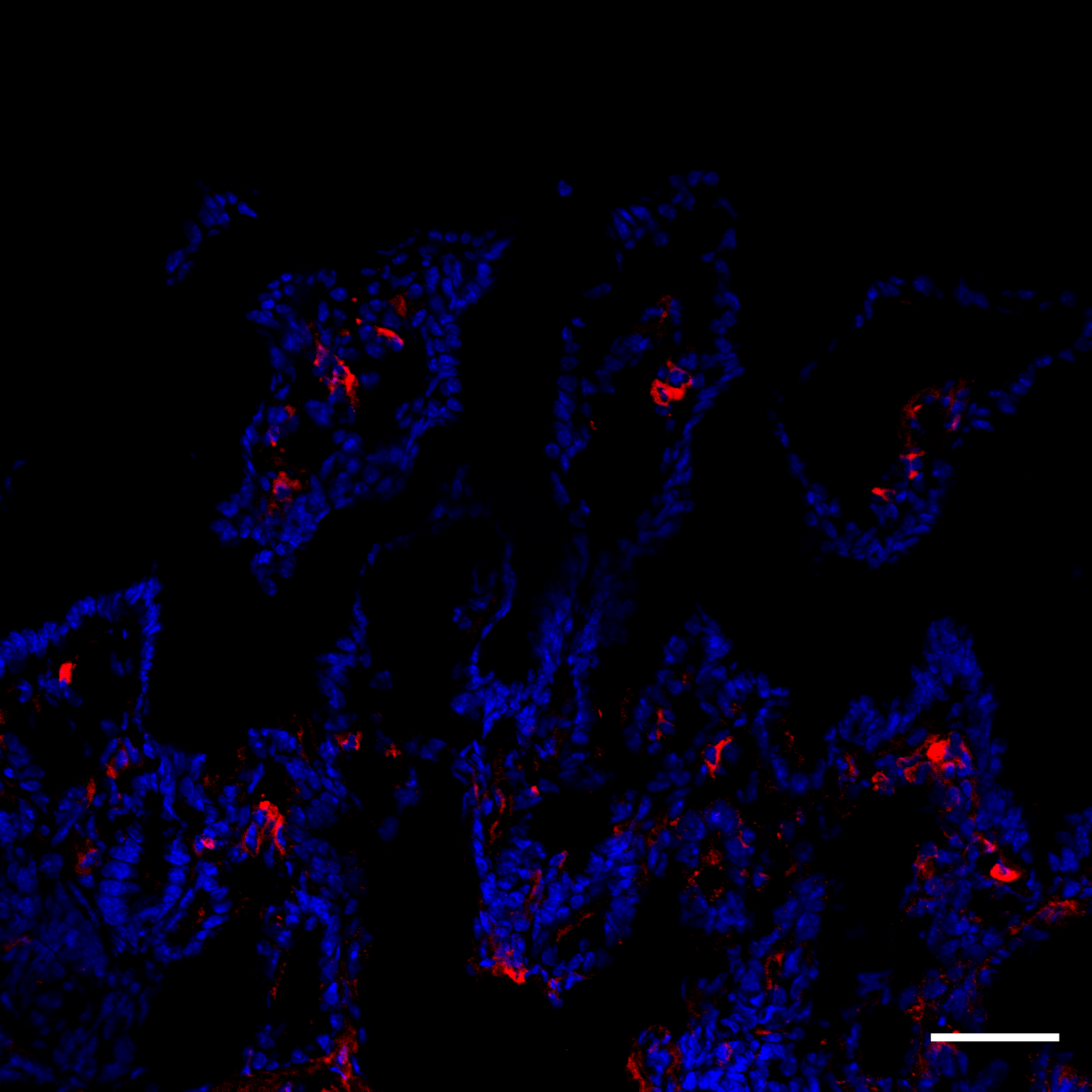

Supplement: Supplementary file 13 — Figure EV1-4 Source Data [file 44318_2024_281_MOESM13_ESM.zip › Figure EV1/EV1J/IF LY6G Ileum VSV-SARS-CoV-2.tif]

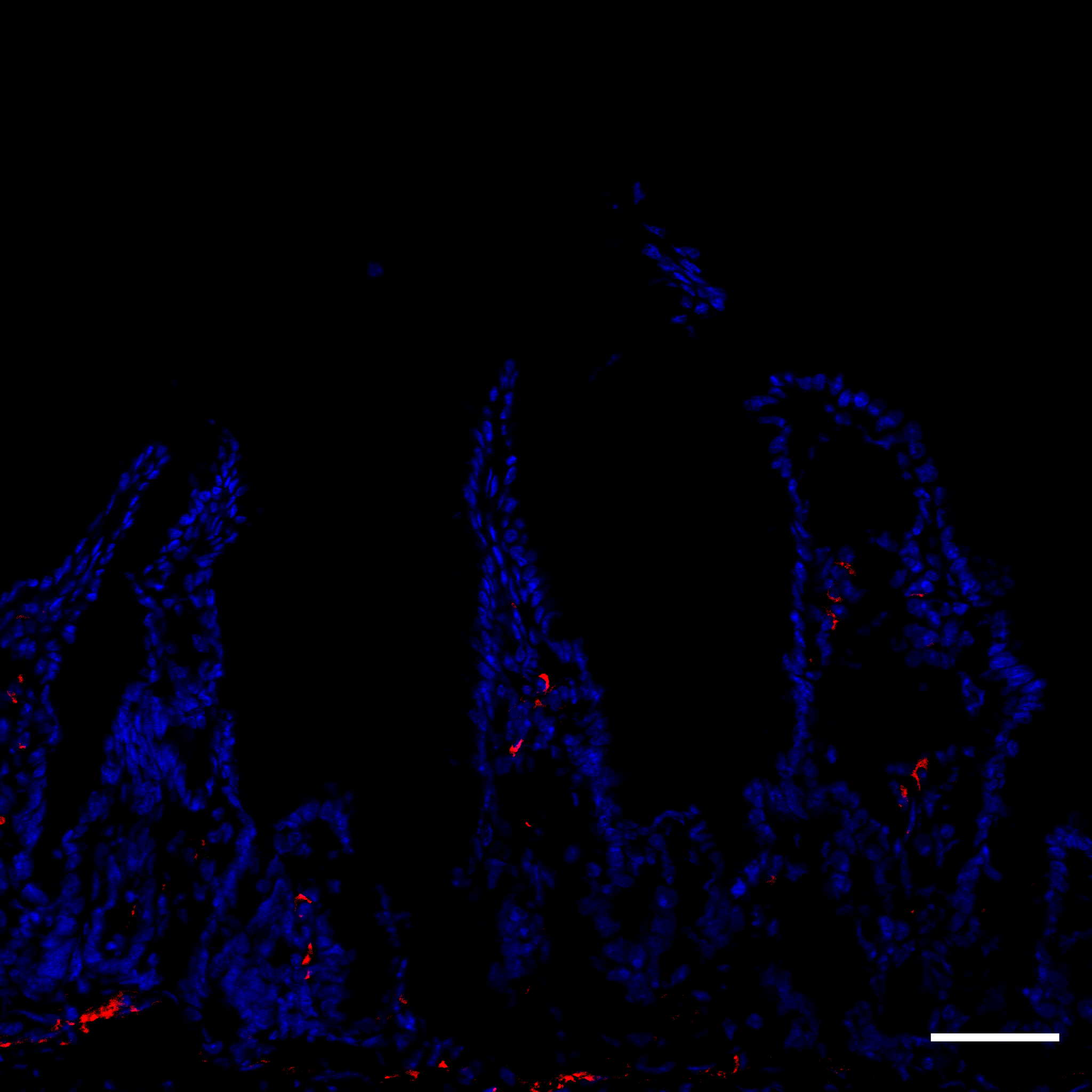

Supplement: Supplementary file 13 — Figure EV1-4 Source Data [file 44318_2024_281_MOESM13_ESM.zip › Figure EV1/EV1J/IF LY6G Ileum control.tif]

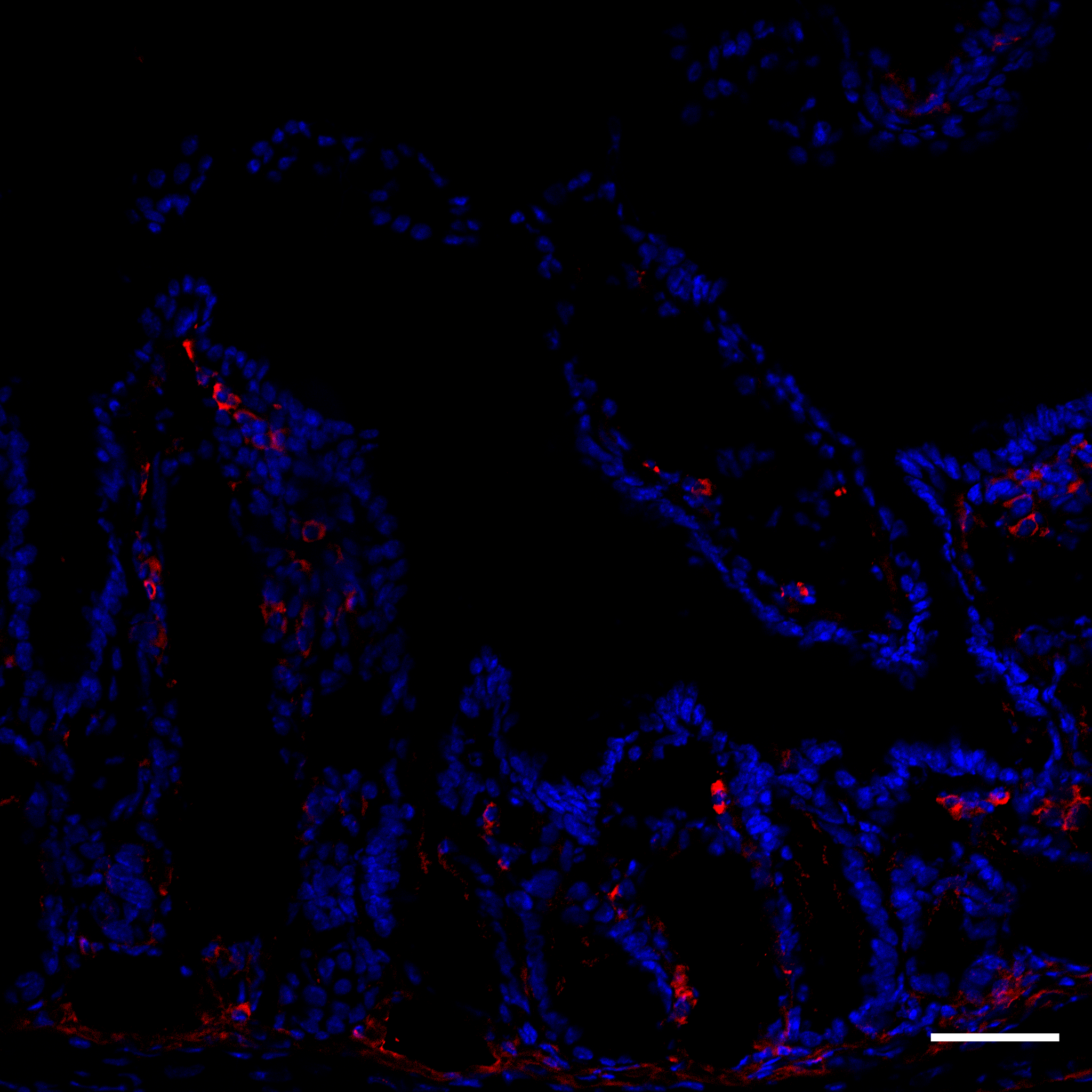

Supplement: Supplementary file 13 — Figure EV1-4 Source Data [file 44318_2024_281_MOESM13_ESM.zip › Figure EV1/EV1J/IF LY6G Jejunum VSV-SARS-CoV-2.tif]

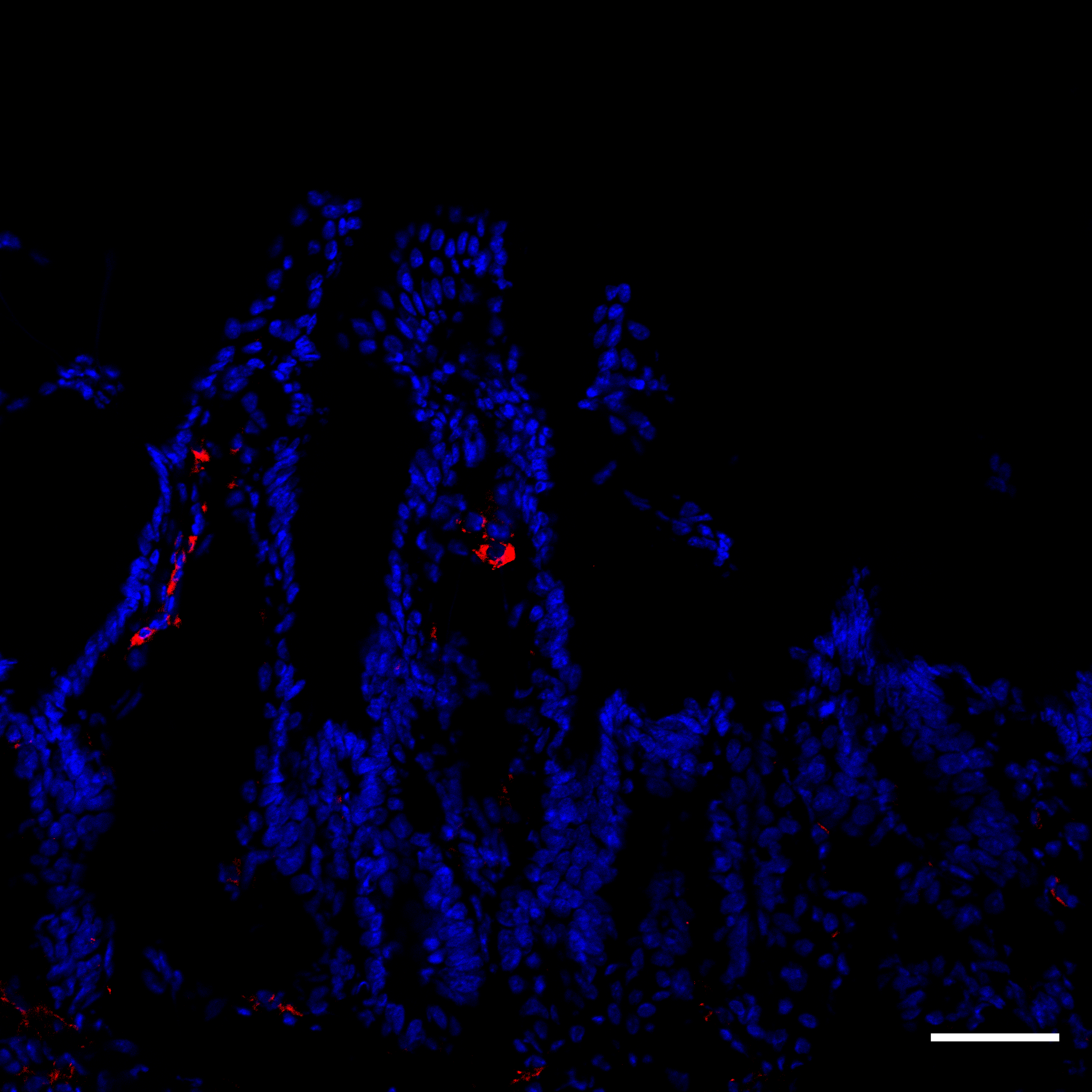

Supplement: Supplementary file 13 — Figure EV1-4 Source Data [file 44318_2024_281_MOESM13_ESM.zip › Figure EV1/EV1J/IF LY6G Jejunum control.tif]

kDa

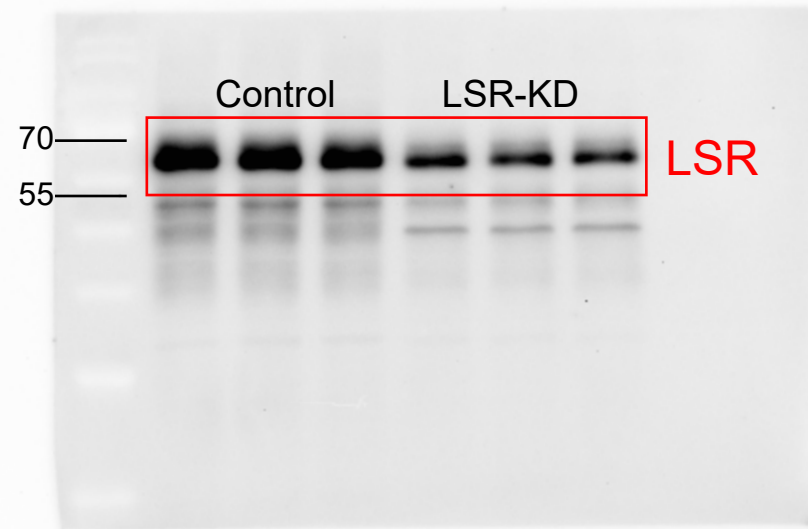

kDa

55 —

Control

LSR-KD

40 —

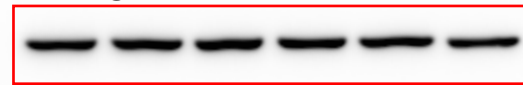

$\beta$ -actin

kDa

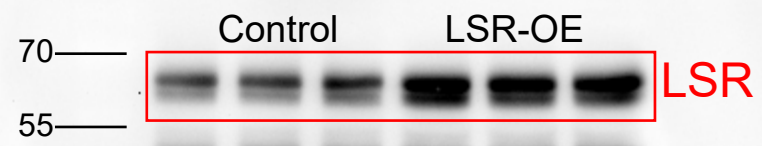

kDa

55 —

Control

LSR-OE

40 —

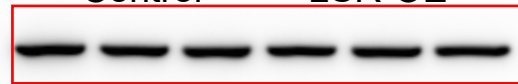

β-actin

Supplement: Supplementary file 13 — Figure EV1-4 Source Data [file 44318_2024_281_MOESM13_ESM.zip › Figure EV2/EV2B/EV2B.pdf]

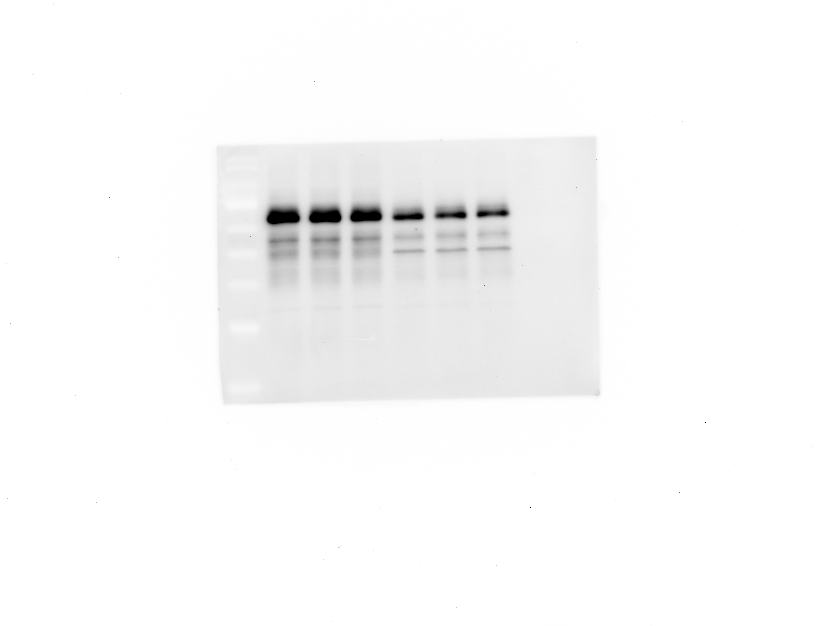

Supplement: Supplementary file 13 — Figure EV1-4 Source Data [file 44318_2024_281_MOESM13_ESM.zip › Figure EV2/EV2B/western LSR LSR-KD.png]

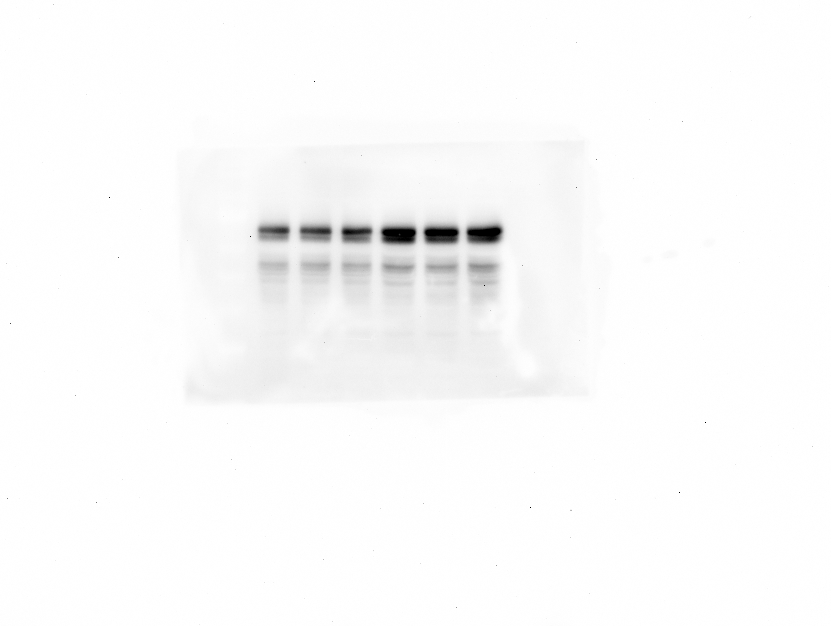

Supplement: Supplementary file 13 — Figure EV1-4 Source Data [file 44318_2024_281_MOESM13_ESM.zip › Figure EV2/EV2B/western LSR LSR-OE.png]

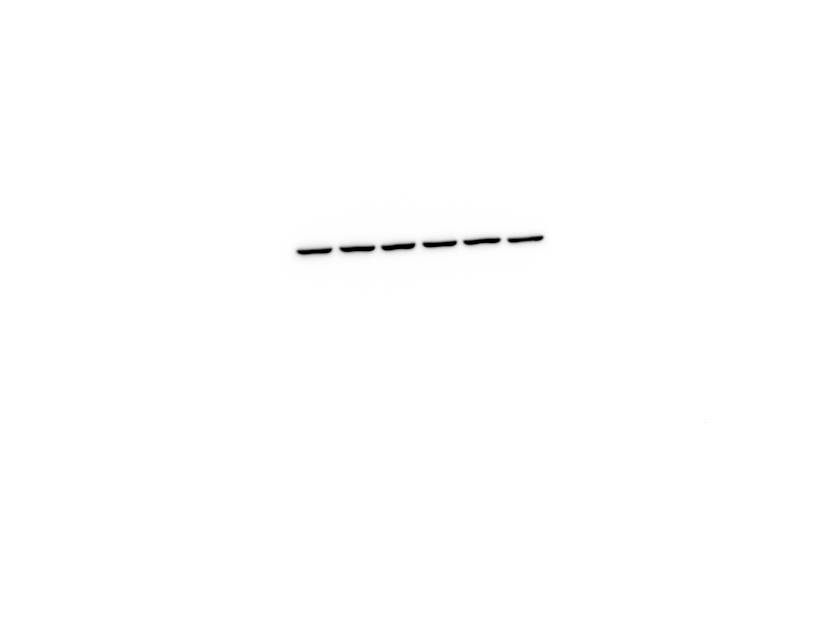

Supplement: Supplementary file 13 — Figure EV1-4 Source Data [file 44318_2024_281_MOESM13_ESM.zip › Figure EV2/EV2B/western actin LSR-KD.png]

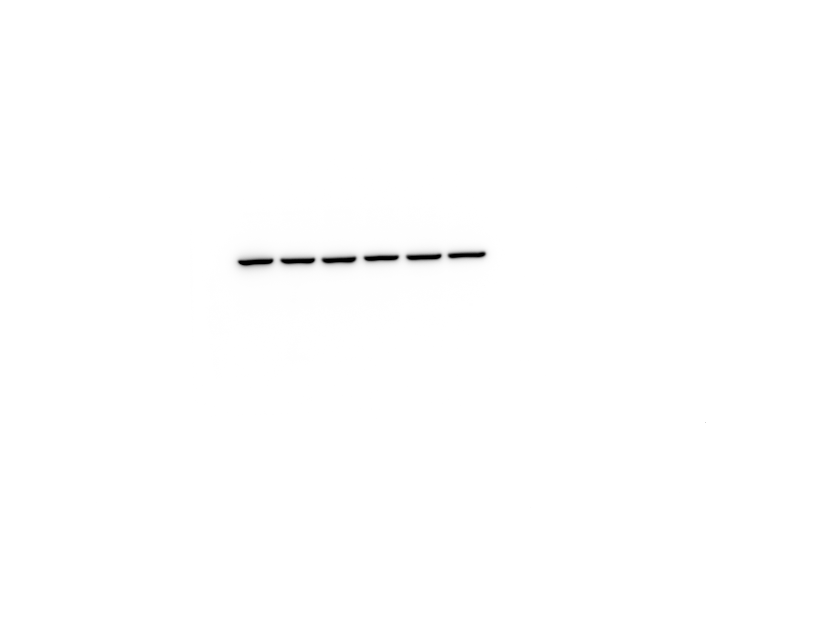

Supplement: Supplementary file 13 — Figure EV1-4 Source Data [file 44318_2024_281_MOESM13_ESM.zip › Figure EV2/EV2B/western actin LSR-OE.png]

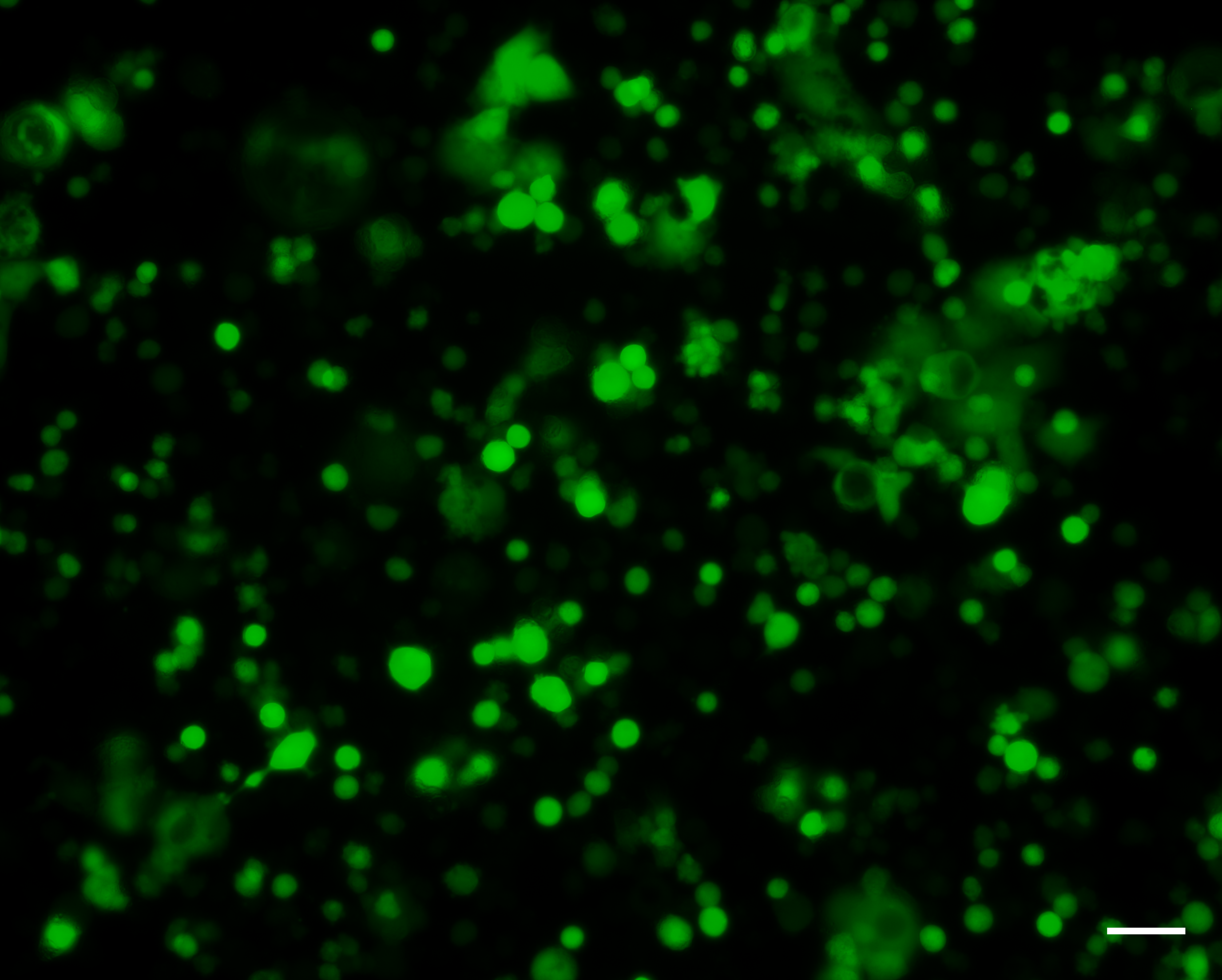

Supplement: Supplementary file 13 — Figure EV1-4 Source Data [file 44318_2024_281_MOESM13_ESM.zip › Figure EV2/EV2E/GFP 0.1μgLSR.tif]

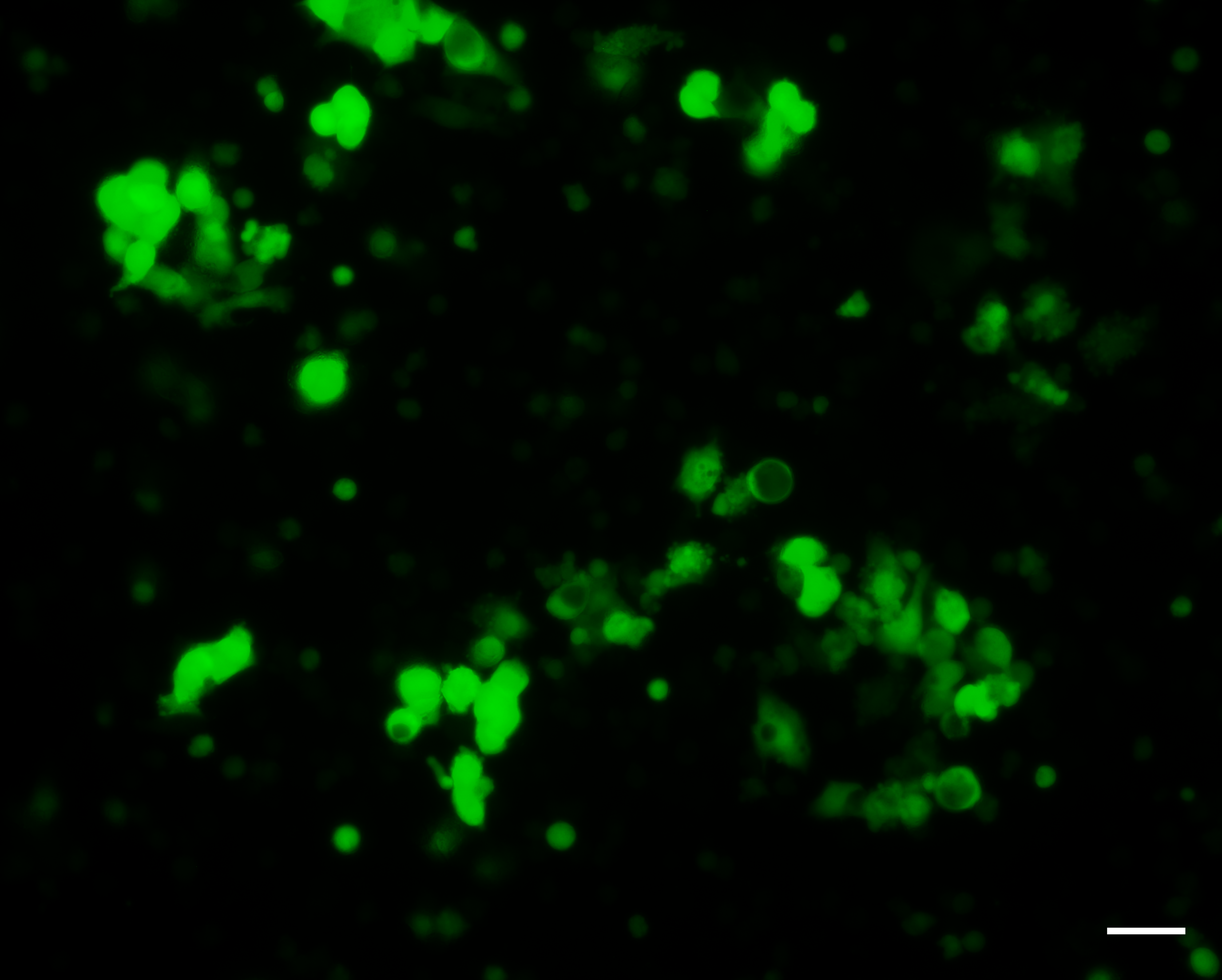

Supplement: Supplementary file 13 — Figure EV1-4 Source Data [file 44318_2024_281_MOESM13_ESM.zip › Figure EV2/EV2E/GFP 0.5μgLSR.tif]

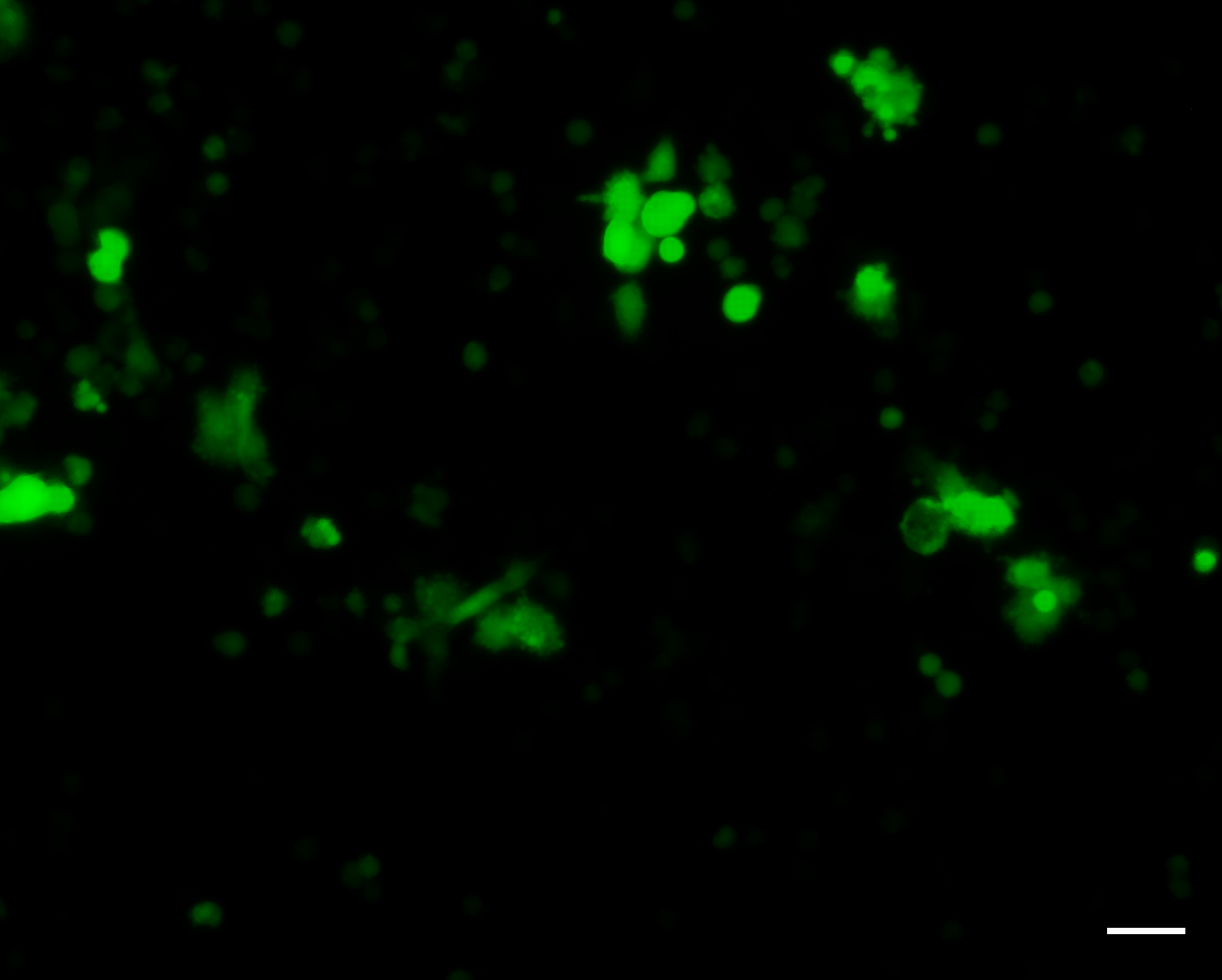

Supplement: Supplementary file 13 — Figure EV1-4 Source Data [file 44318_2024_281_MOESM13_ESM.zip › Figure EV2/EV2E/GFP 1μgLSR.tif]

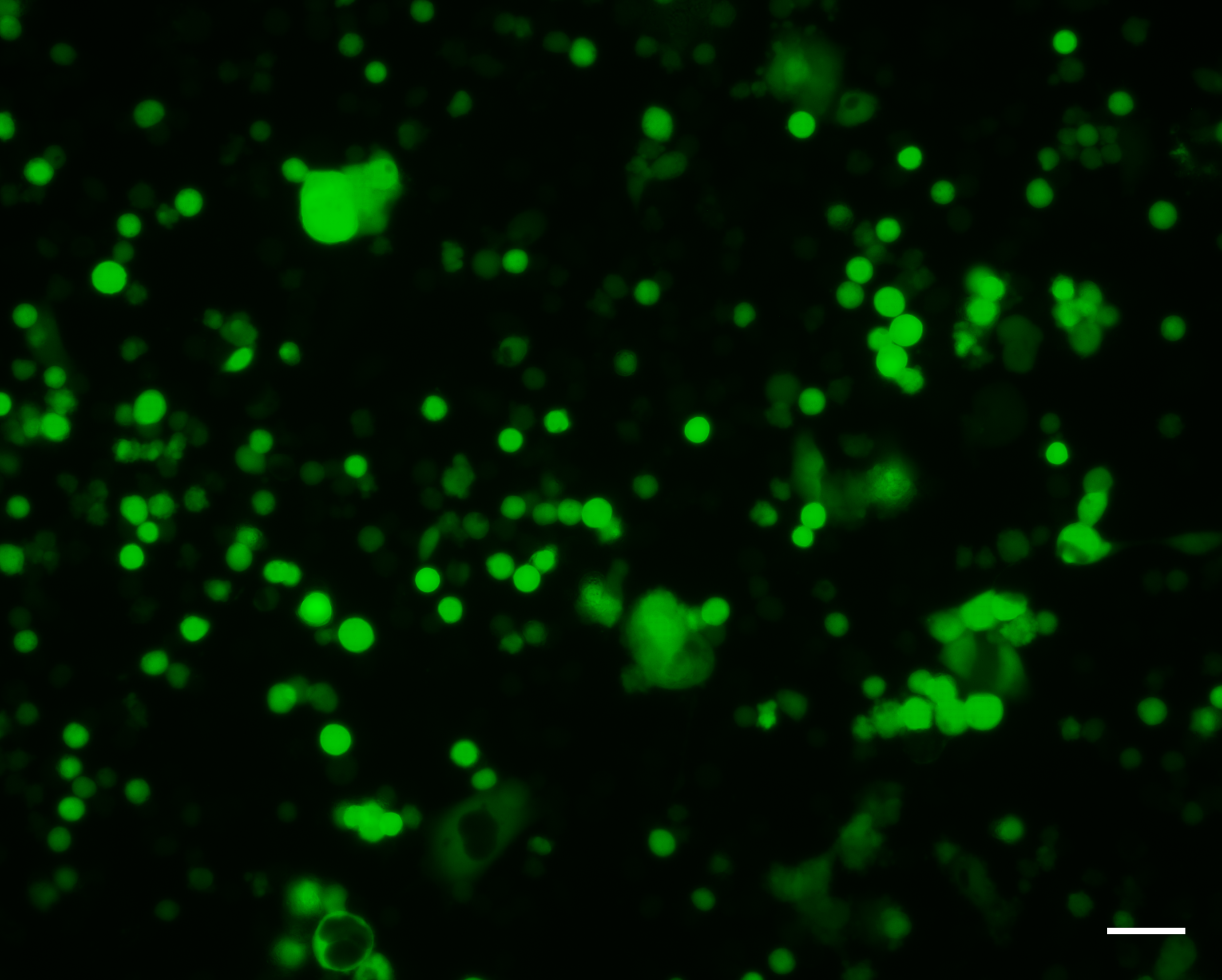

Supplement: Supplementary file 13 — Figure EV1-4 Source Data [file 44318_2024_281_MOESM13_ESM.zip › Figure EV2/EV2E/GFP control.tif]

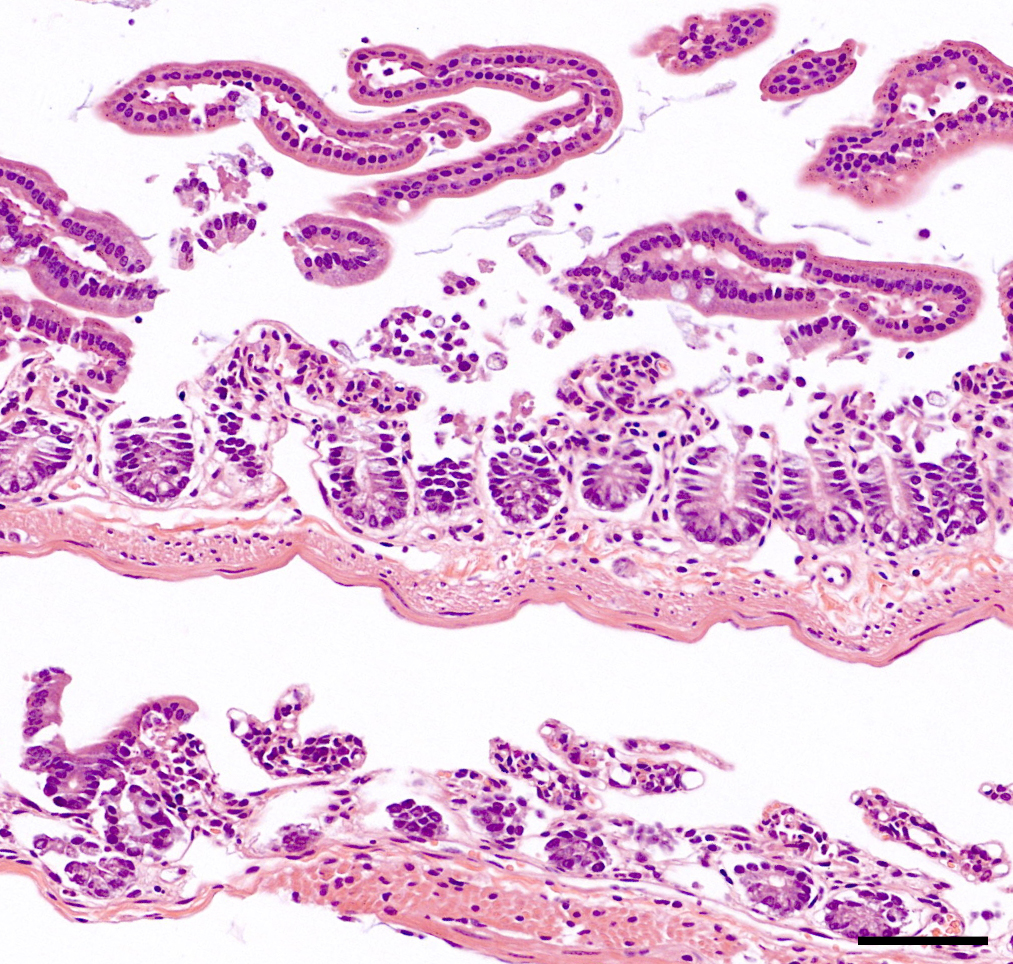

Supplement: Supplementary file 13 — Figure EV1-4 Source Data [file 44318_2024_281_MOESM13_ESM.zip › Figure EV2/EV2I/HE LsrvillKO.tif]

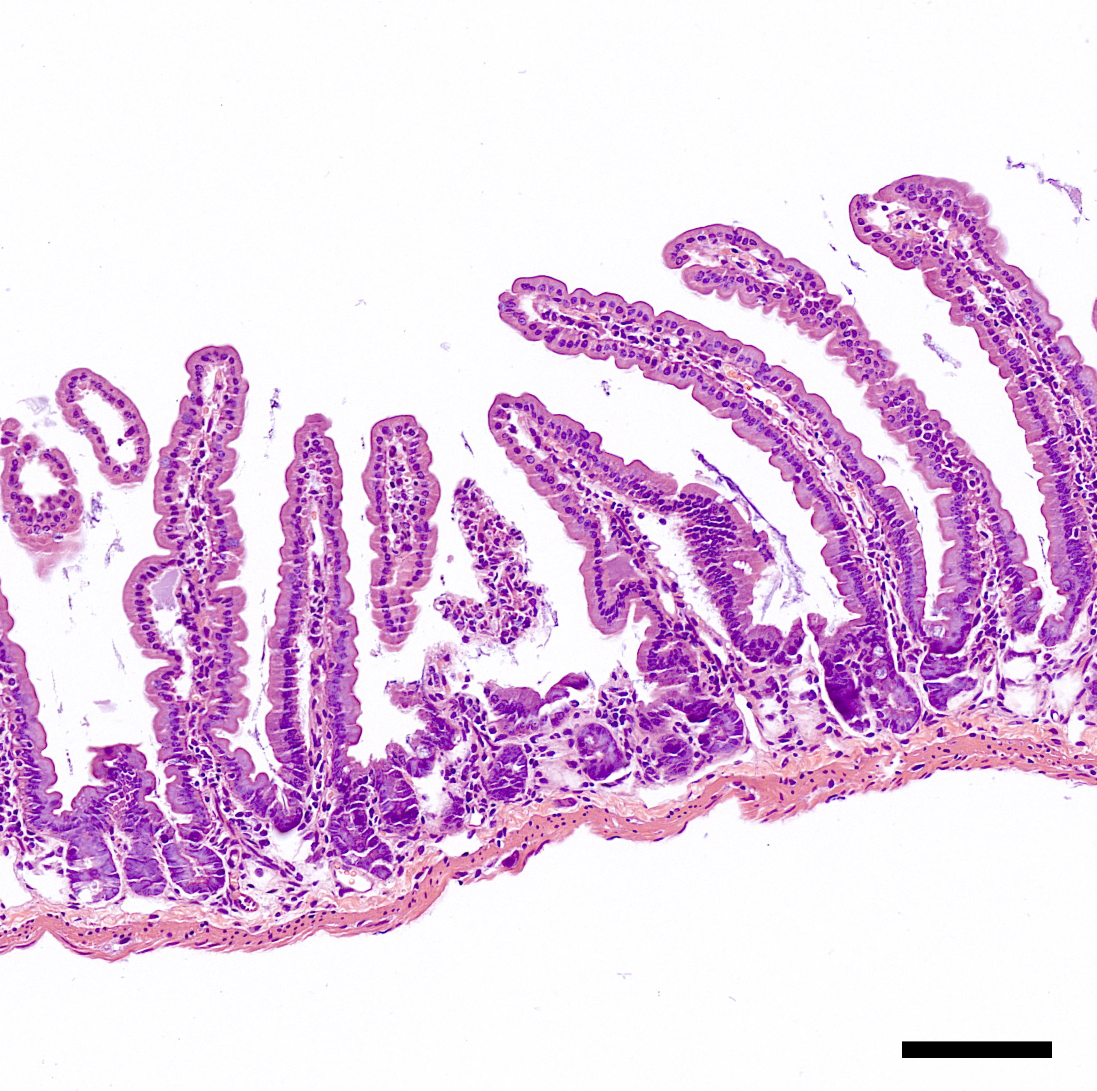

Supplement: Supplementary file 13 — Figure EV1-4 Source Data [file 44318_2024_281_MOESM13_ESM.zip › Figure EV2/EV2I/HE WT.tif]

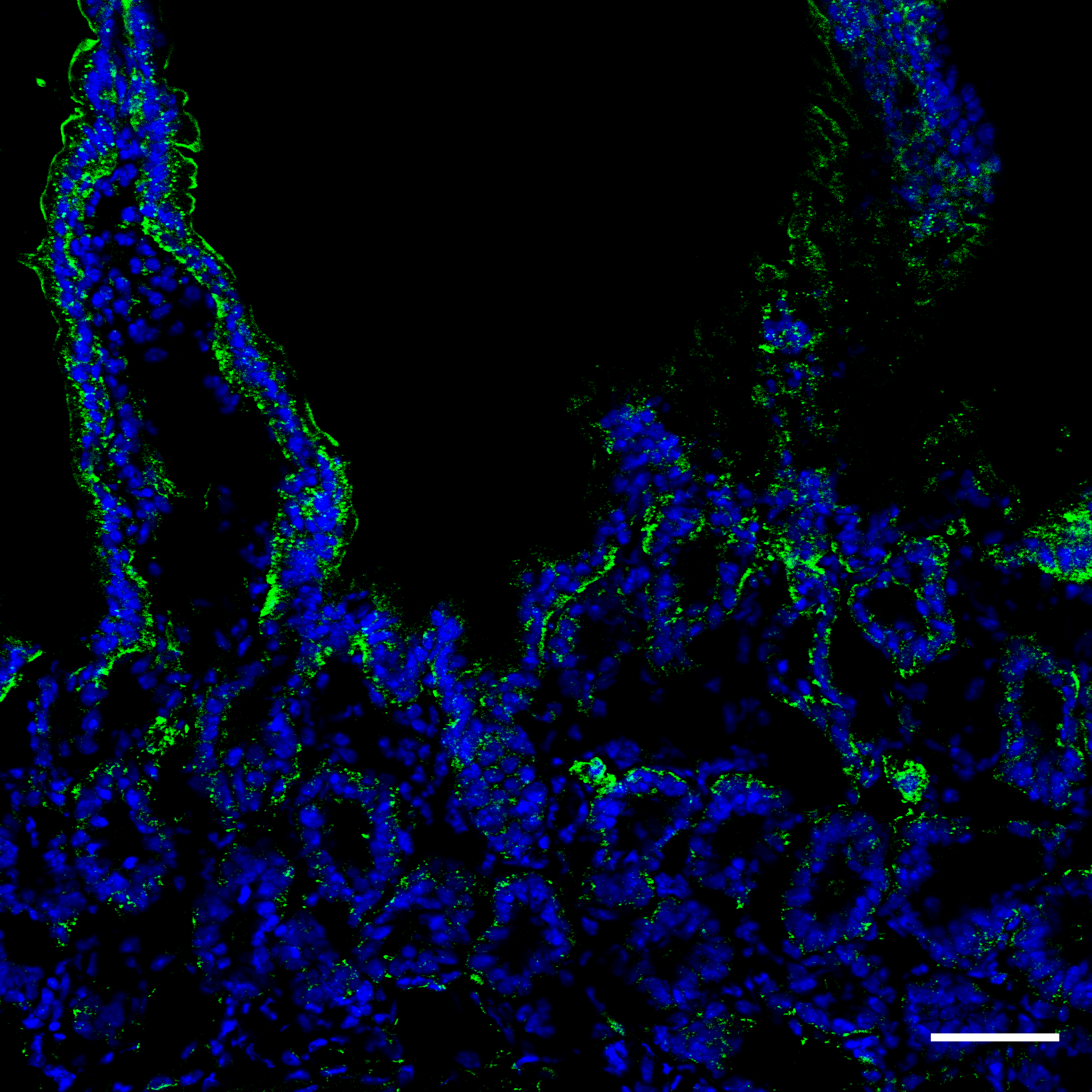

Supplement: Supplementary file 13 — Figure EV1-4 Source Data [file 44318_2024_281_MOESM13_ESM.zip › Figure EV2/EV2K/GFP VSV-SARS-CoV-2 LsrvillKO.tif]

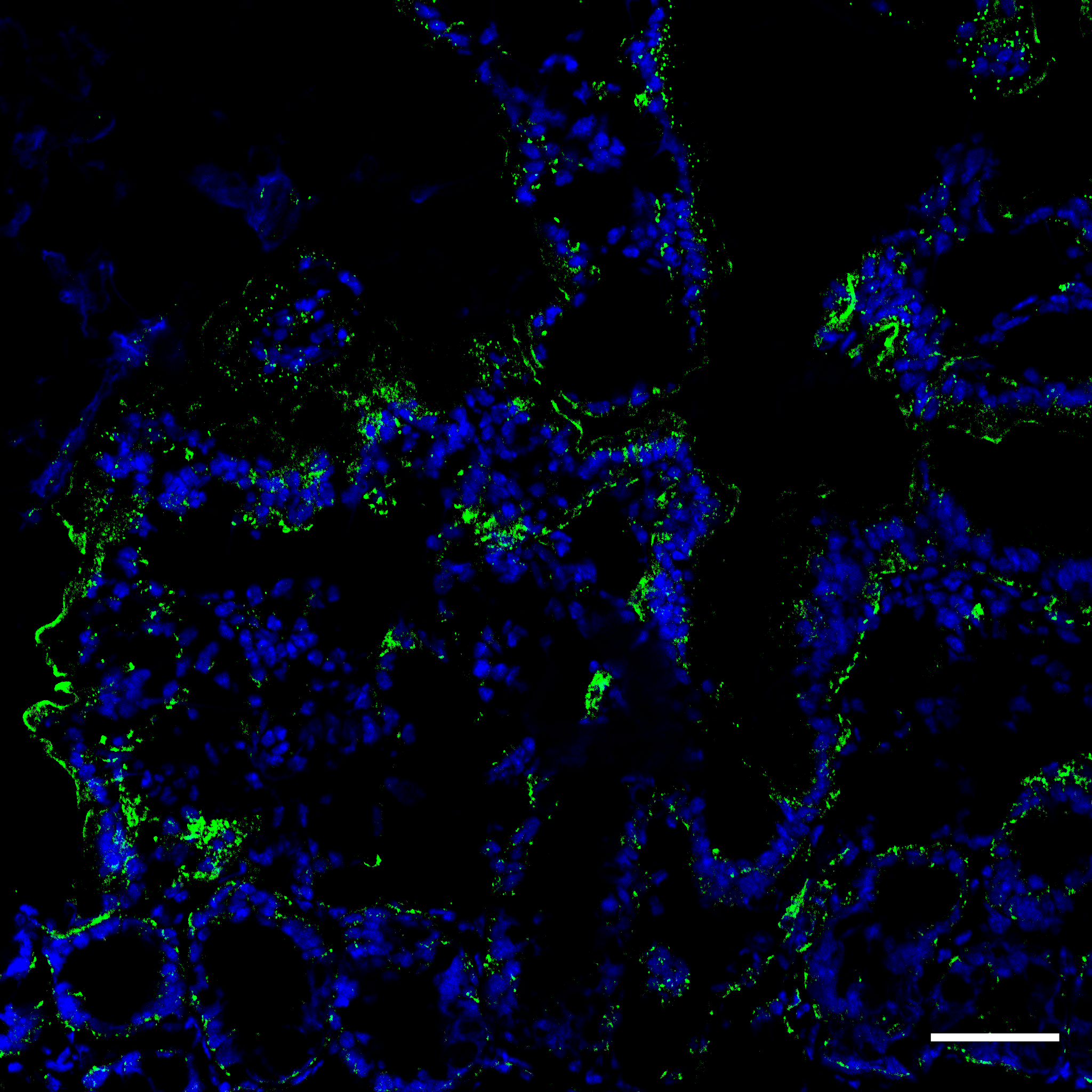

Supplement: Supplementary file 13 — Figure EV1-4 Source Data [file 44318_2024_281_MOESM13_ESM.zip › Figure EV2/EV2K/GFP VSV-SARS-CoV-2 WT.tif]

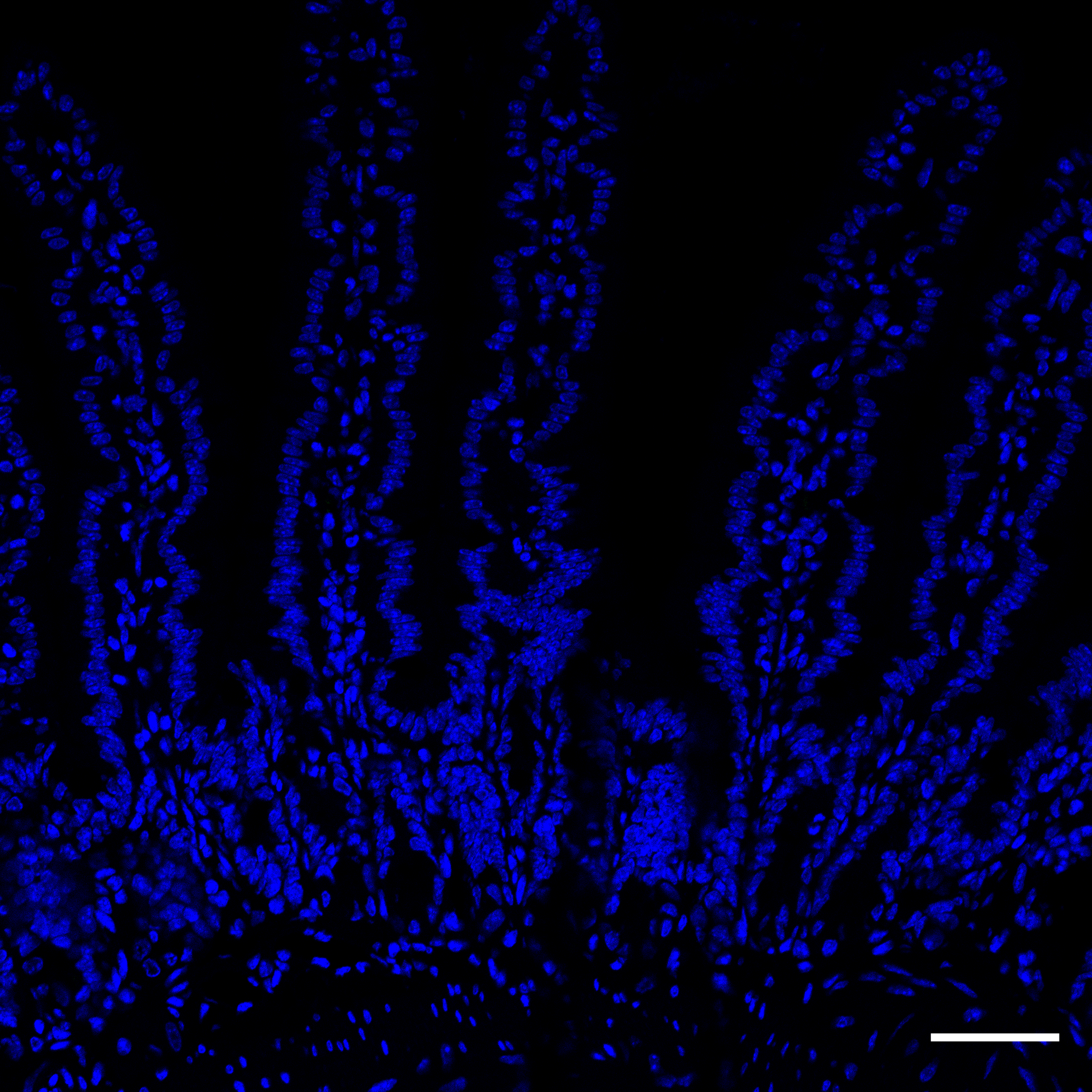

Supplement: Supplementary file 13 — Figure EV1-4 Source Data [file 44318_2024_281_MOESM13_ESM.zip › Figure EV2/EV2K/GFP control LsrvillKO.tif]

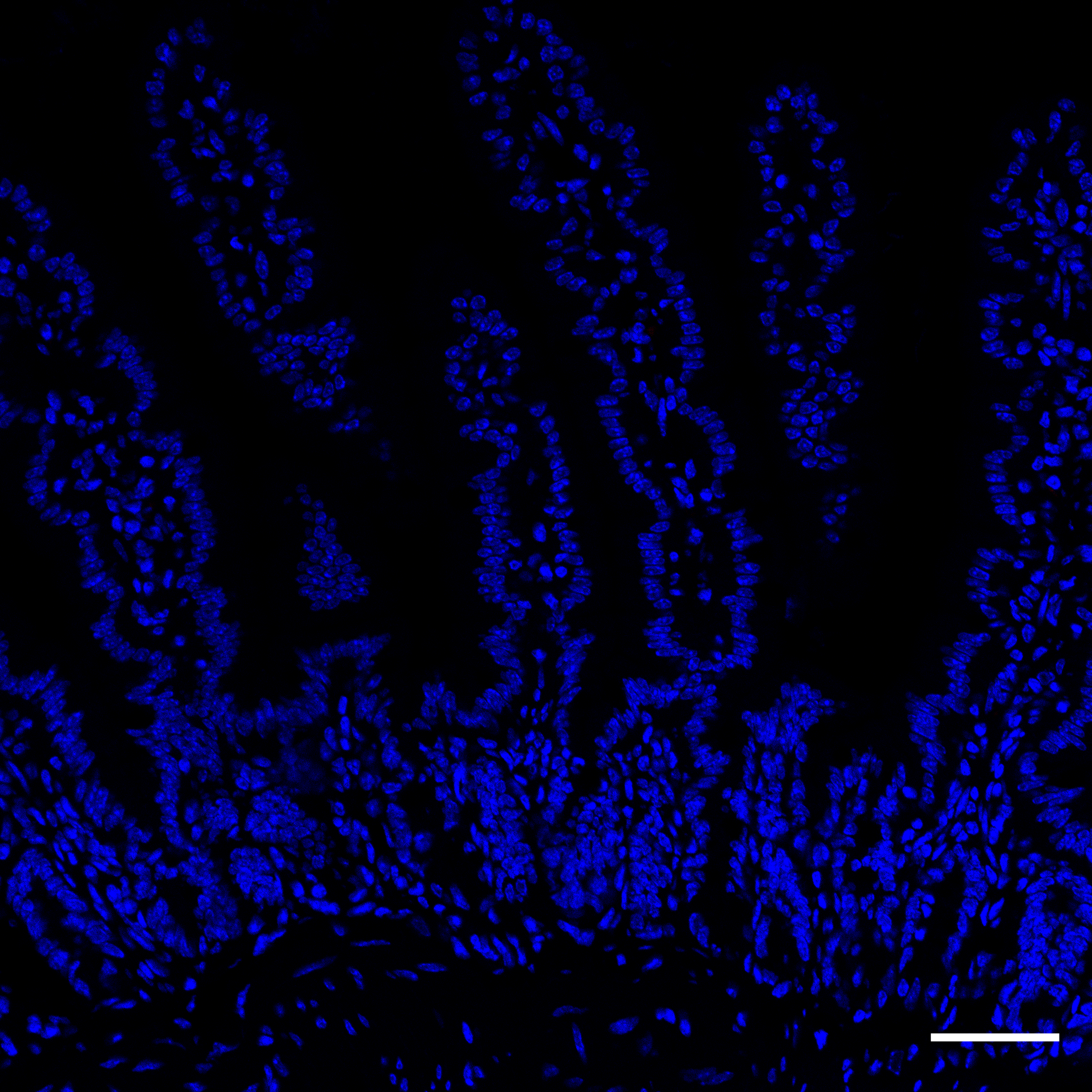

Supplement: Supplementary file 13 — Figure EV1-4 Source Data [file 44318_2024_281_MOESM13_ESM.zip › Figure EV2/EV2K/GFP control WT.tif]

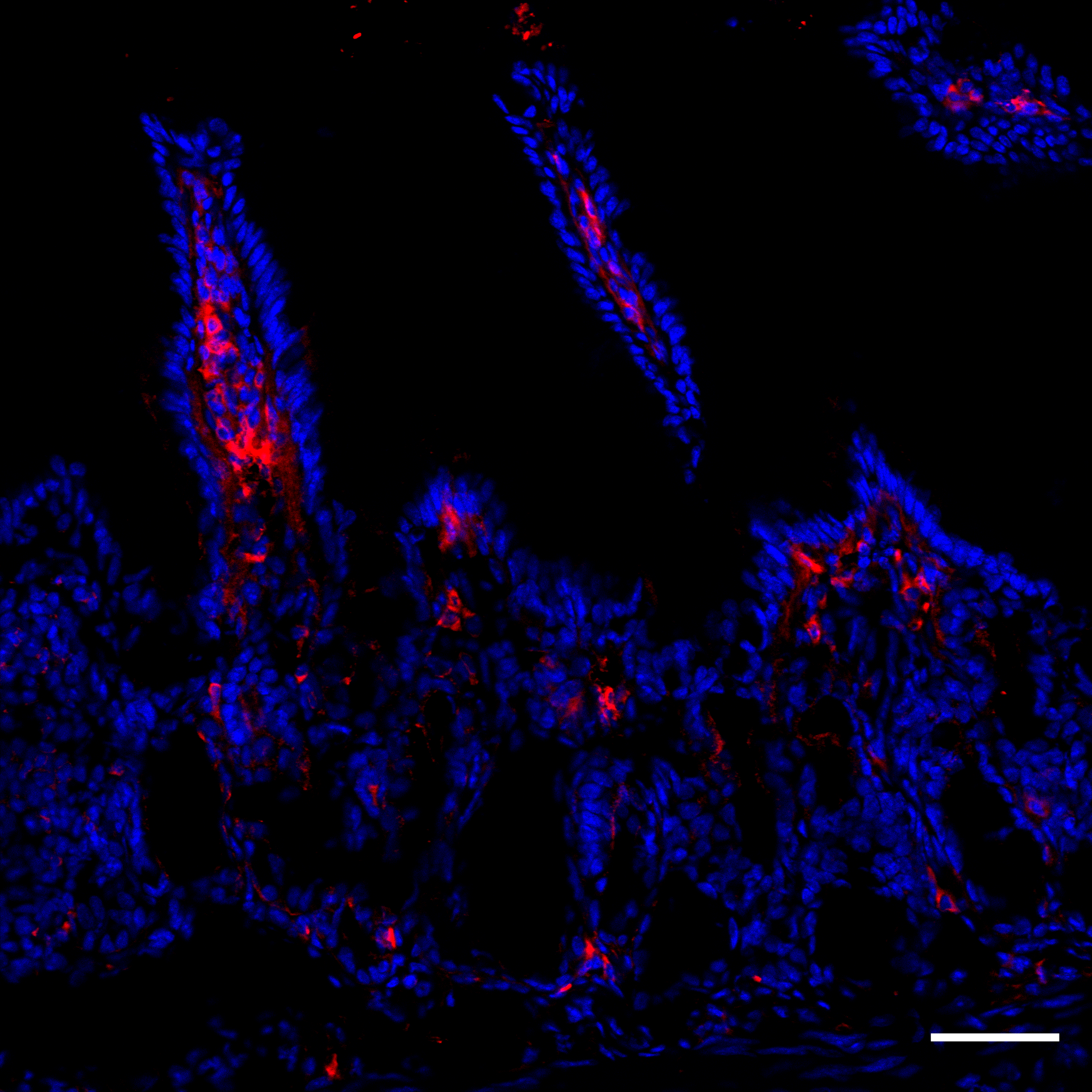

Supplement: Supplementary file 13 — Figure EV1-4 Source Data [file 44318_2024_281_MOESM13_ESM.zip › Figure EV2/EV2N/IF LY6G Duodenum LSR-OE.tif]

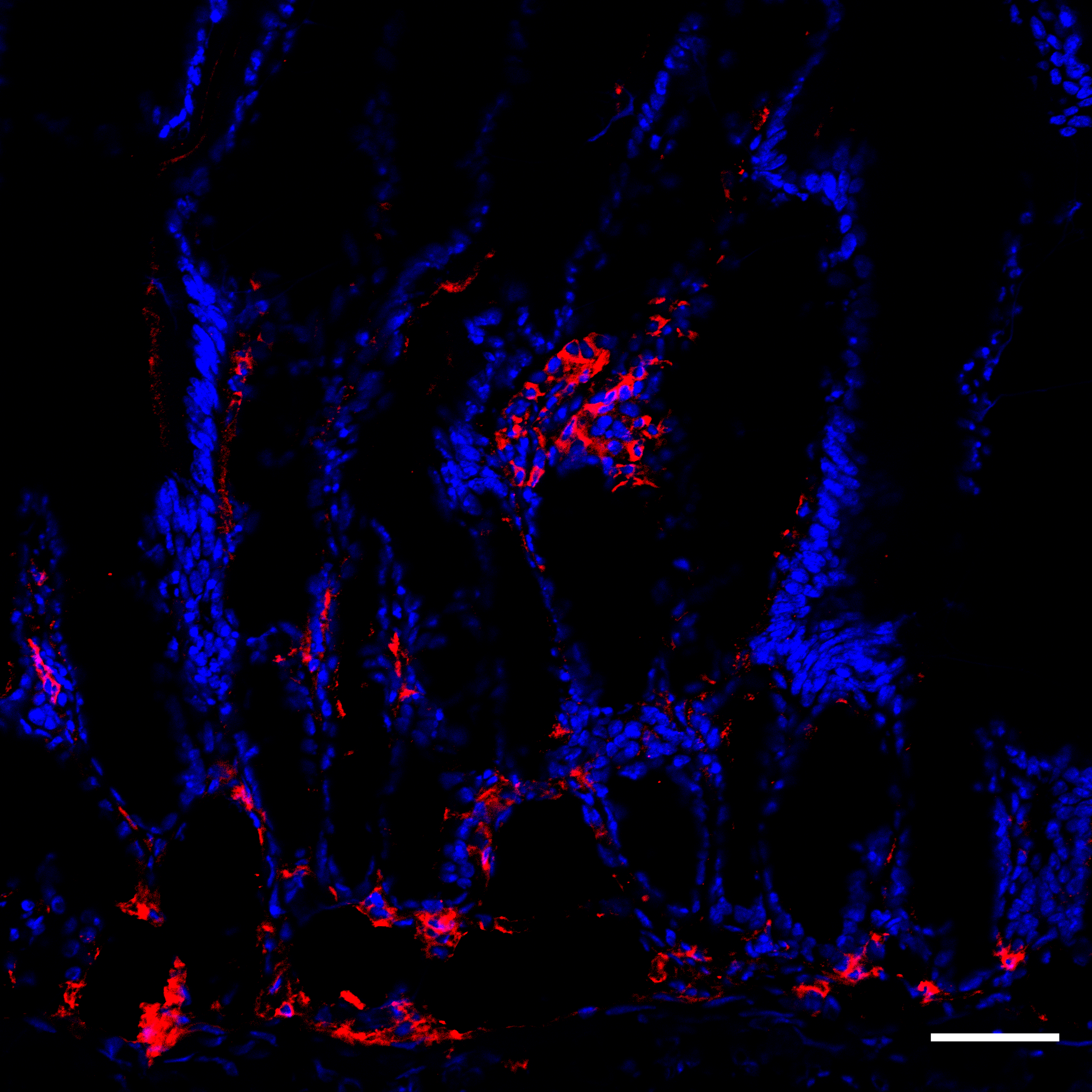

Supplement: Supplementary file 13 — Figure EV1-4 Source Data [file 44318_2024_281_MOESM13_ESM.zip › Figure EV2/EV2N/IF LY6G Duodenum WT.tif]

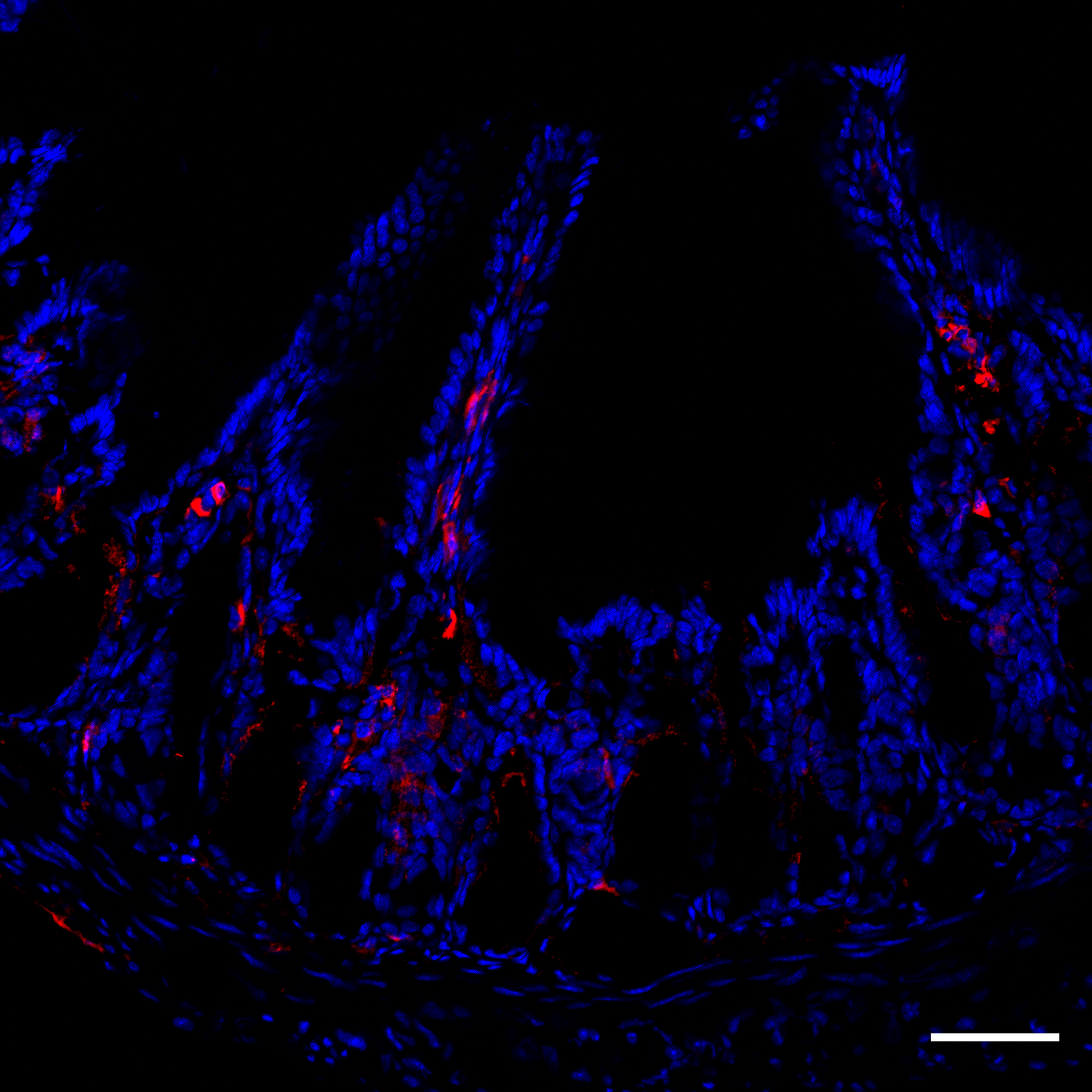

Supplement: Supplementary file 13 — Figure EV1-4 Source Data [file 44318_2024_281_MOESM13_ESM.zip › Figure EV2/EV2N/IF LY6G Ileum LSR-OE.tif]
