# Supplementary figures and images for: Tight junction protein LSR is a host defense factor against SARS-CoV-2 infection in the small intestine (part 4 of 4)
Source: EMBO J. 2024 Oct 23;43(23):6124–51. doi: 10.1038/s44318-024-00281-4 (PMC11612383; doi:10.1038/s44318-024-00281-4)

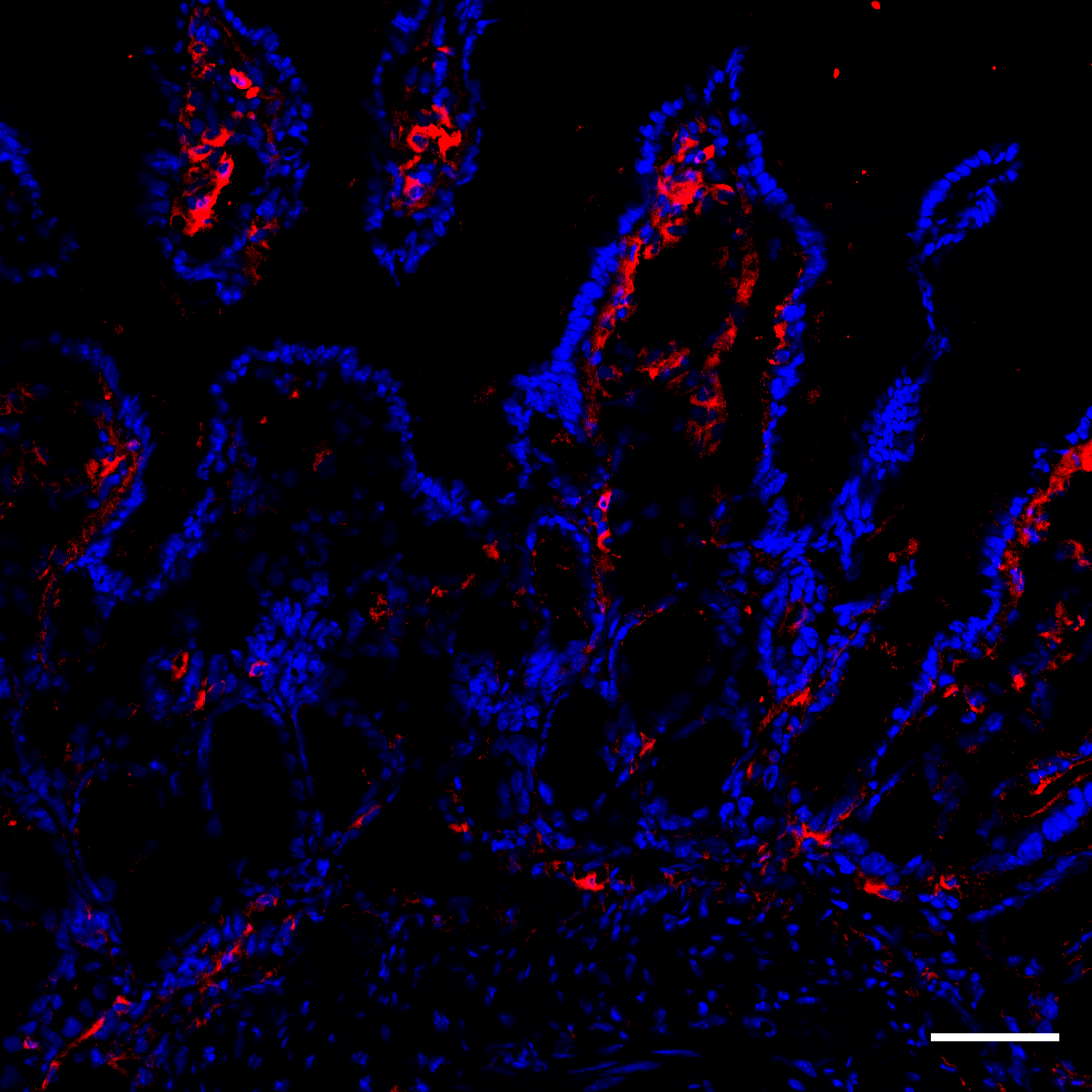

Supplement: Supplementary file 13 — Figure EV1-4 Source Data [file 44318_2024_281_MOESM13_ESM.zip › Figure EV2/EV2N/IF LY6G Ileum WT.tif]

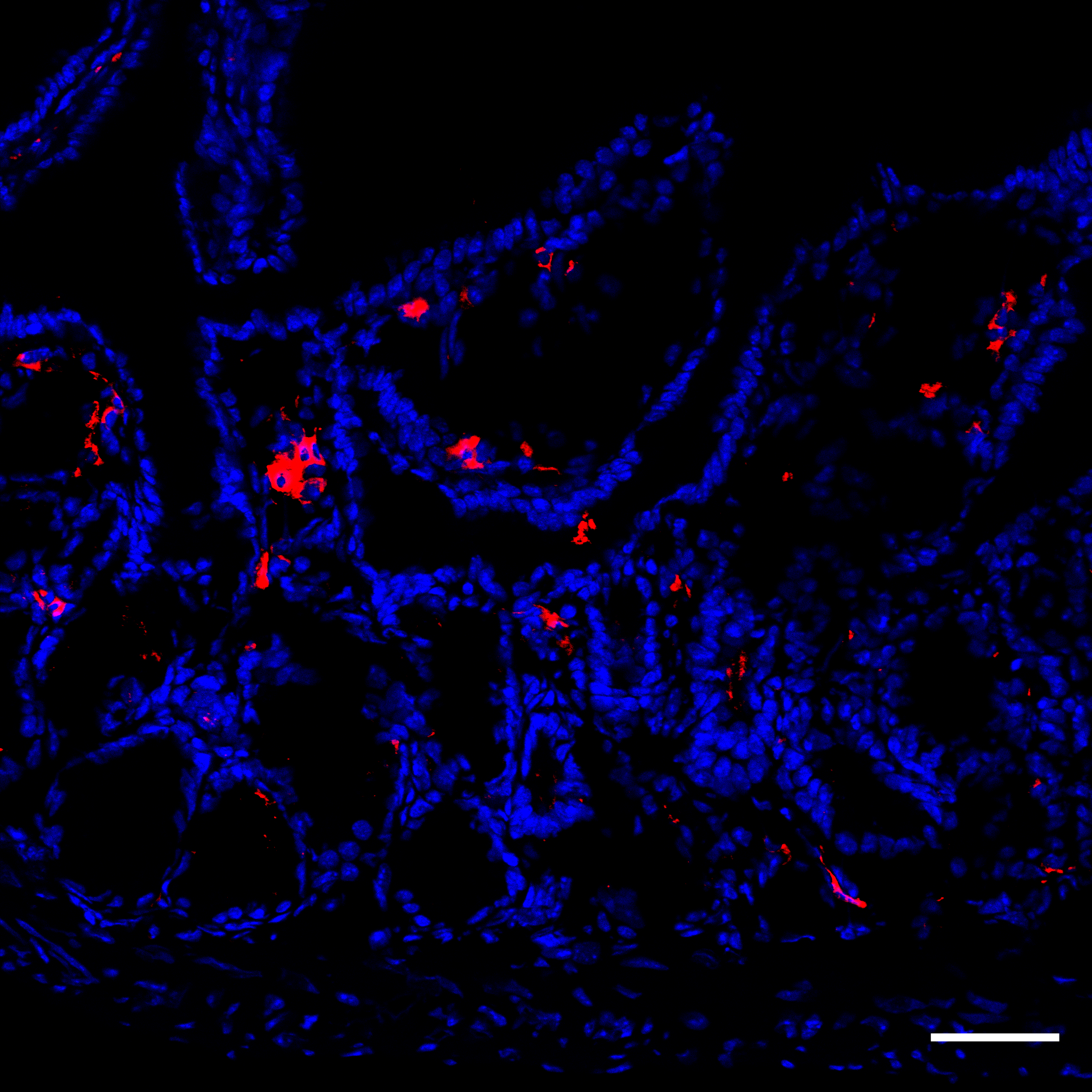

Supplement: Supplementary file 13 — Figure EV1-4 Source Data [file 44318_2024_281_MOESM13_ESM.zip › Figure EV2/EV2N/IF LY6G Jejunum LSR-OE.tif]

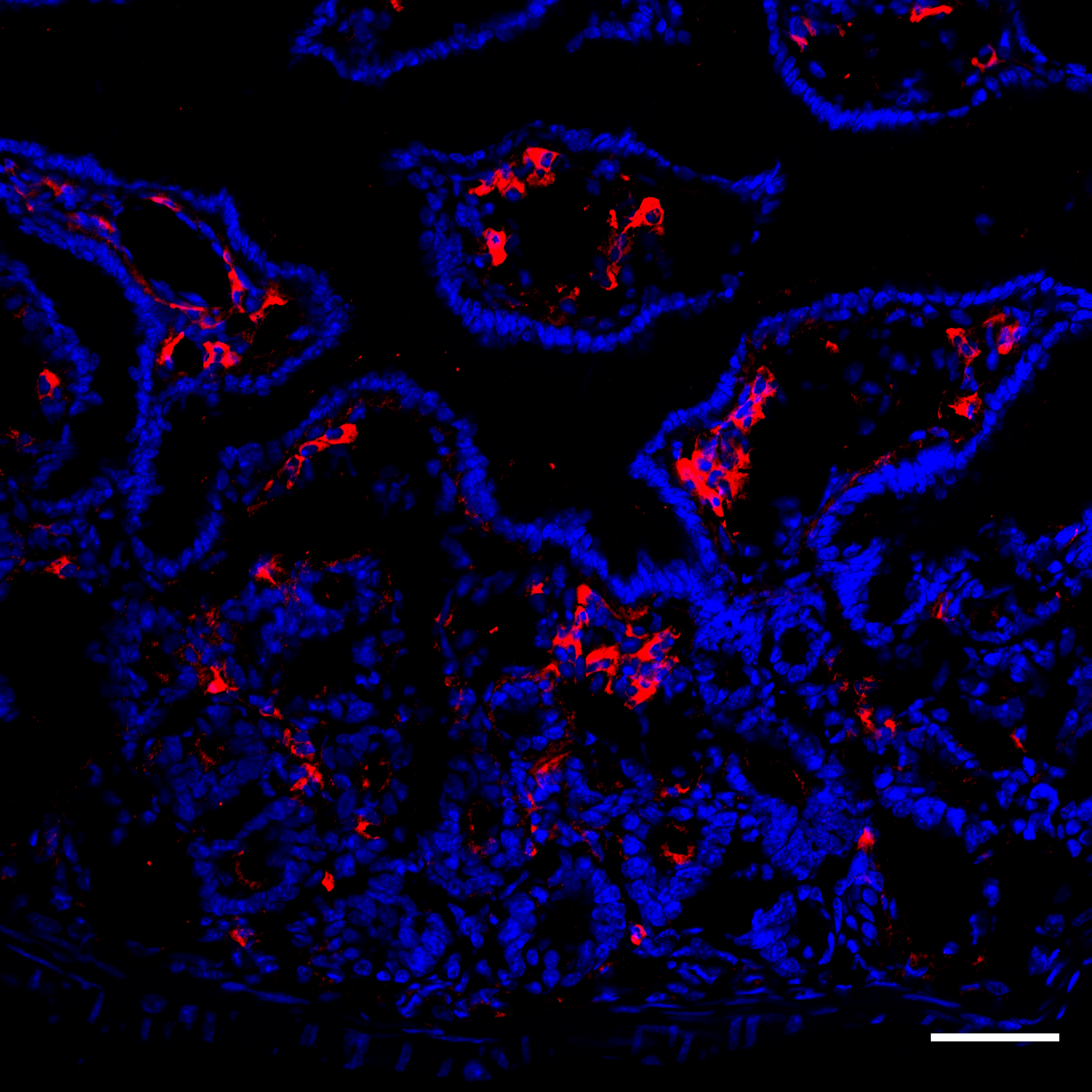

Supplement: Supplementary file 13 — Figure EV1-4 Source Data [file 44318_2024_281_MOESM13_ESM.zip › Figure EV2/EV2N/IF LY6G Jejunum WT.tif]

kDa

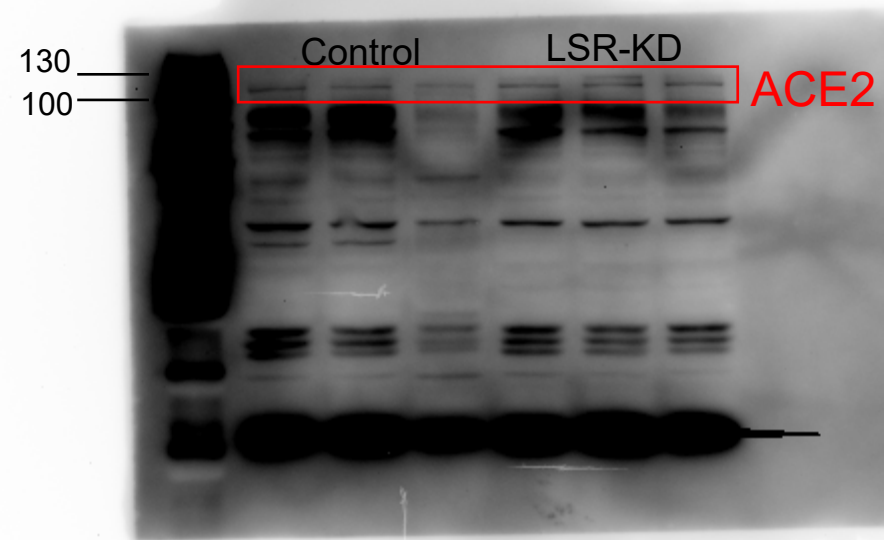

kDa

55 —

40 —

Control

LSR-KD

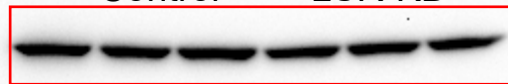

β-actin

kDa

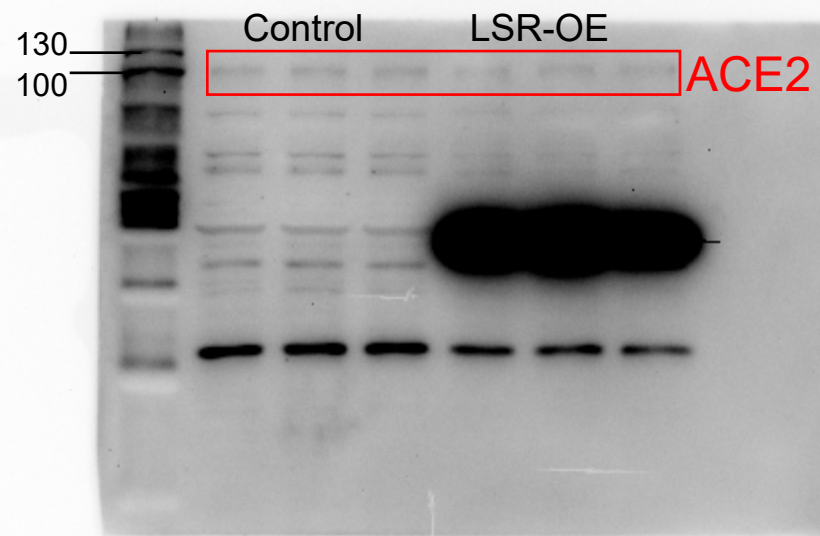

kDa

130—  
100—

Control

LSR-OE

Sodium-potassium ATPase

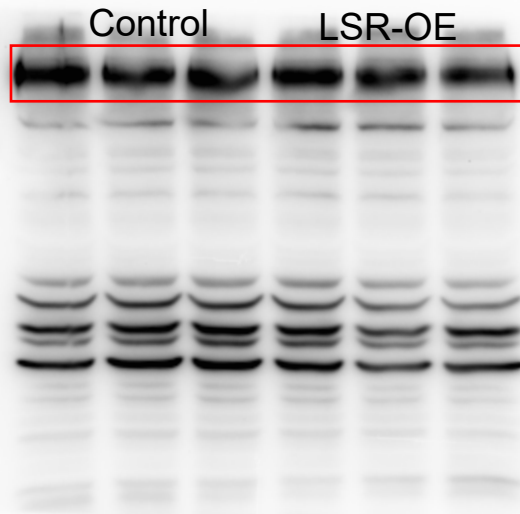

Supplement: Supplementary file 13 — Figure EV1-4 Source Data [file 44318_2024_281_MOESM13_ESM.zip › Figure EV3/EV3A/EV3A.pdf]

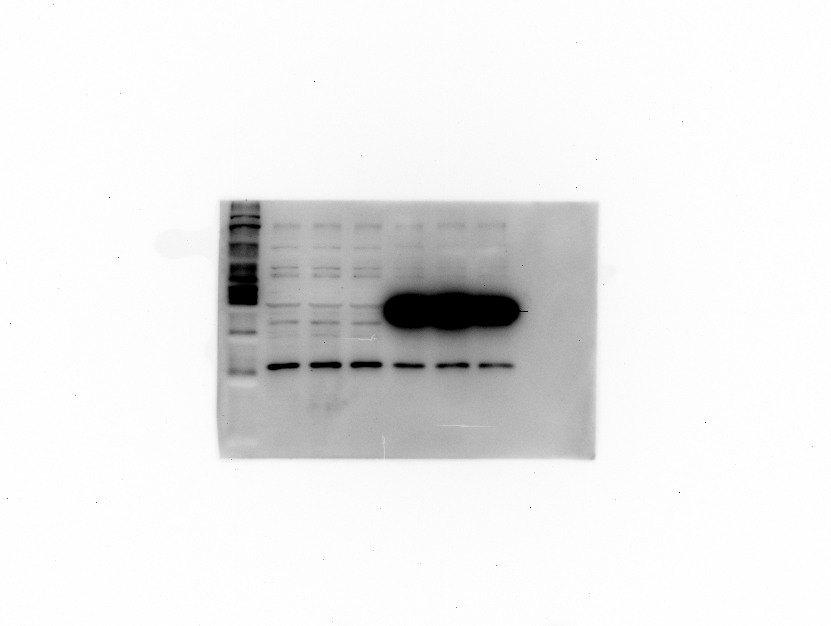

Supplement: Supplementary file 13 — Figure EV1-4 Source Data [file 44318_2024_281_MOESM13_ESM.zip › Figure EV3/EV3A/western ACE2 membrane.png]

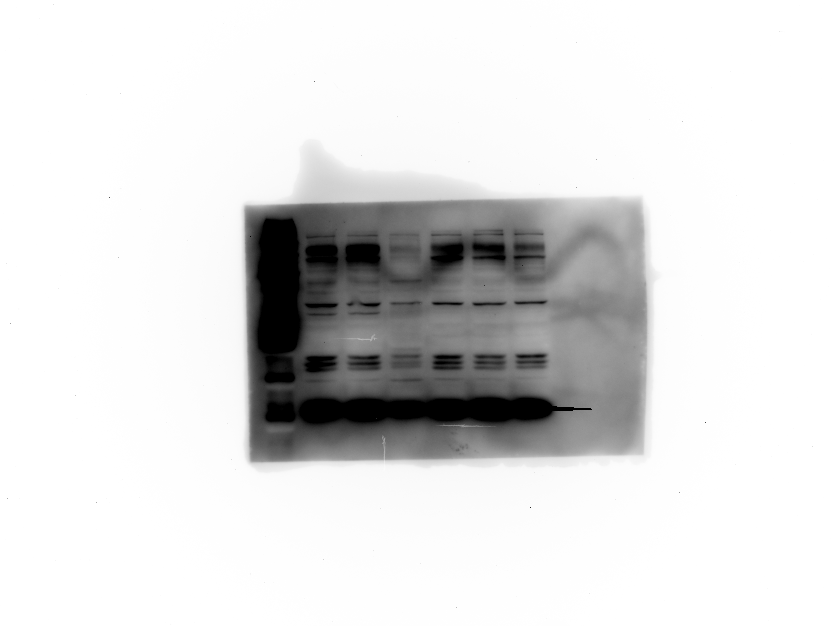

Supplement: Supplementary file 13 — Figure EV1-4 Source Data [file 44318_2024_281_MOESM13_ESM.zip › Figure EV3/EV3A/western ACE2 total.png]

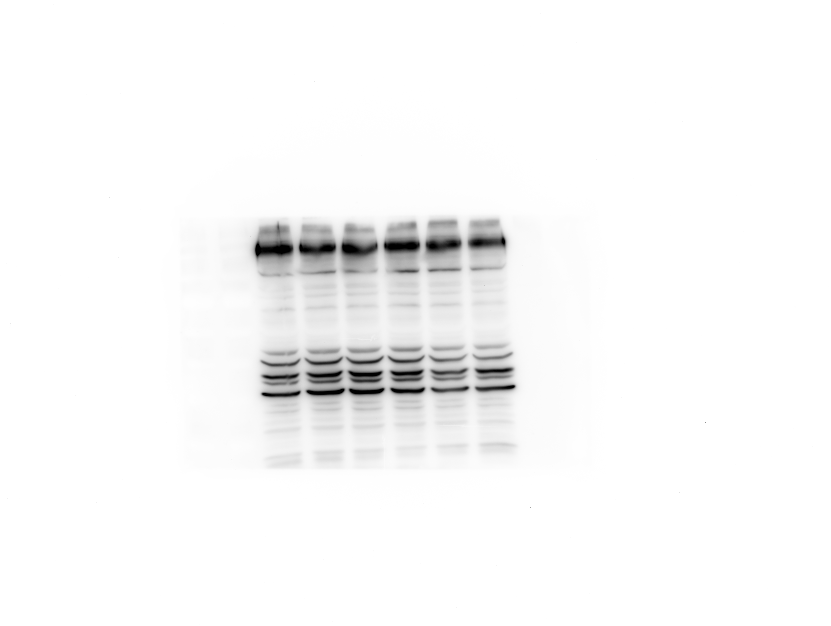

Supplement: Supplementary file 13 — Figure EV1-4 Source Data [file 44318_2024_281_MOESM13_ESM.zip › Figure EV3/EV3A/western Sodium-potassium ATPase membrane.png]

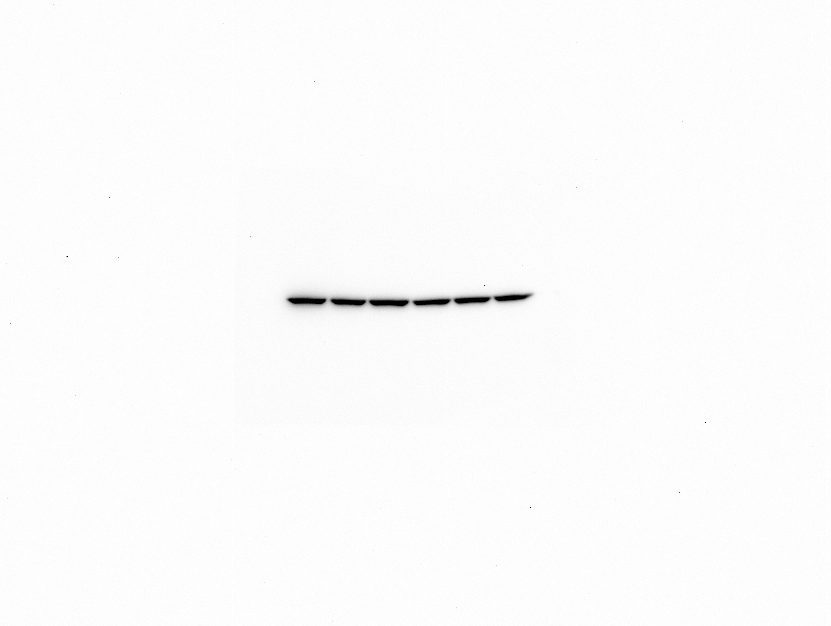

Supplement: Supplementary file 13 — Figure EV1-4 Source Data [file 44318_2024_281_MOESM13_ESM.zip › Figure EV3/EV3A/western actin total.png]

kDa

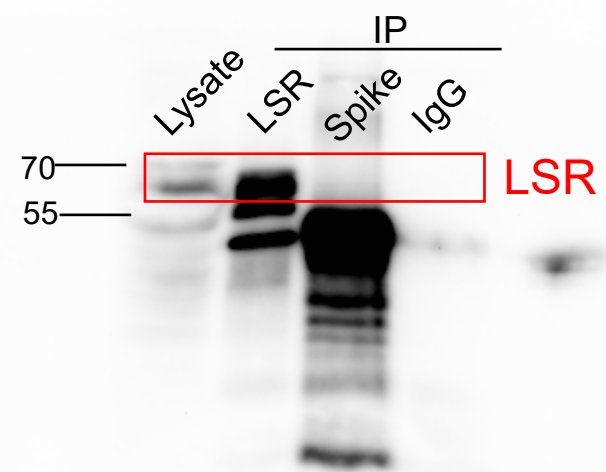

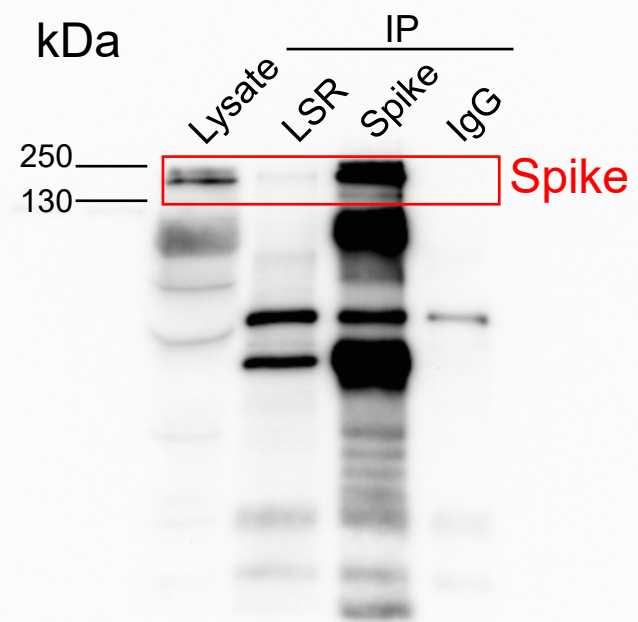

Supplement: Supplementary file 13 — Figure EV1-4 Source Data [file 44318_2024_281_MOESM13_ESM.zip › Figure EV3/EV3B/EV3B.pdf]

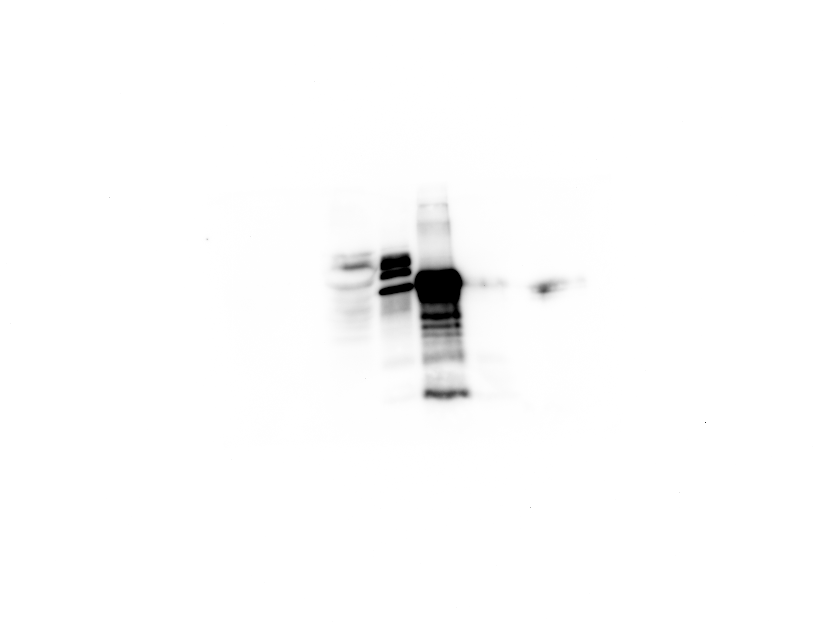

Supplement: Supplementary file 13 — Figure EV1-4 Source Data [file 44318_2024_281_MOESM13_ESM.zip › Figure EV3/EV3B/western LSR.png]

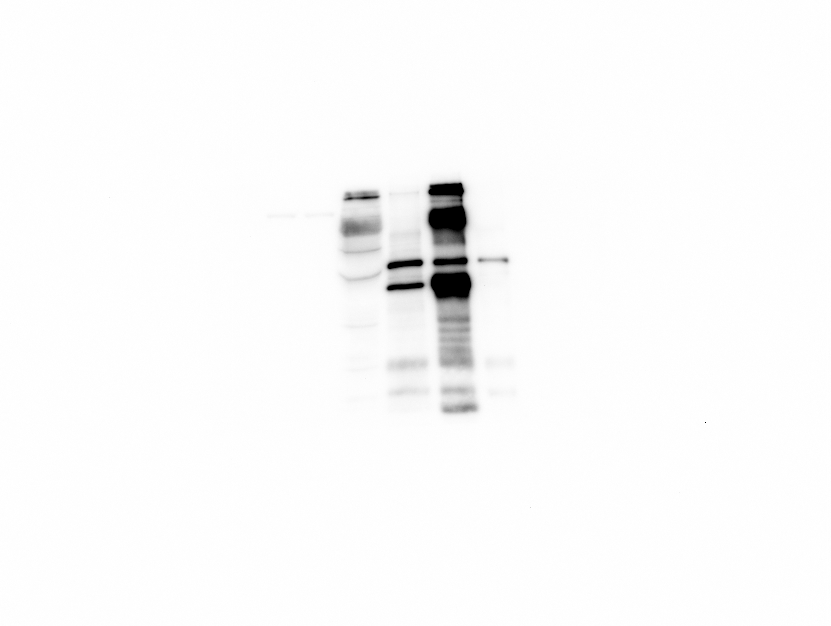

Supplement: Supplementary file 13 — Figure EV1-4 Source Data [file 44318_2024_281_MOESM13_ESM.zip › Figure EV3/EV3B/western Spike.png]

kDa

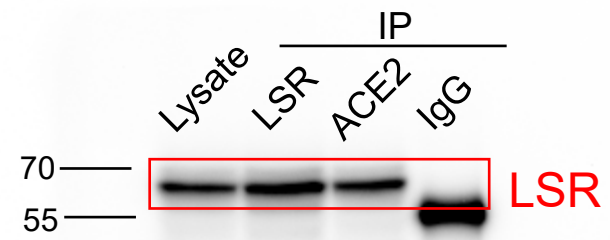

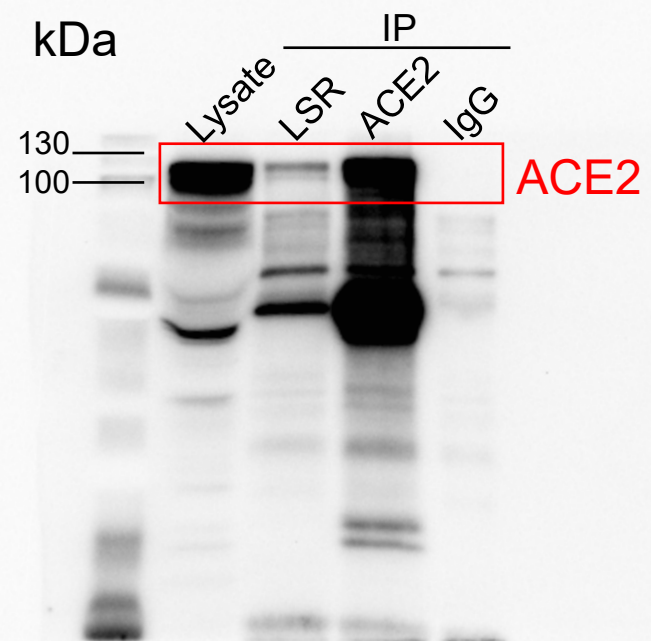

Supplement: Supplementary file 13 — Figure EV1-4 Source Data [file 44318_2024_281_MOESM13_ESM.zip › Figure EV3/EV3D/EV3D.pdf]

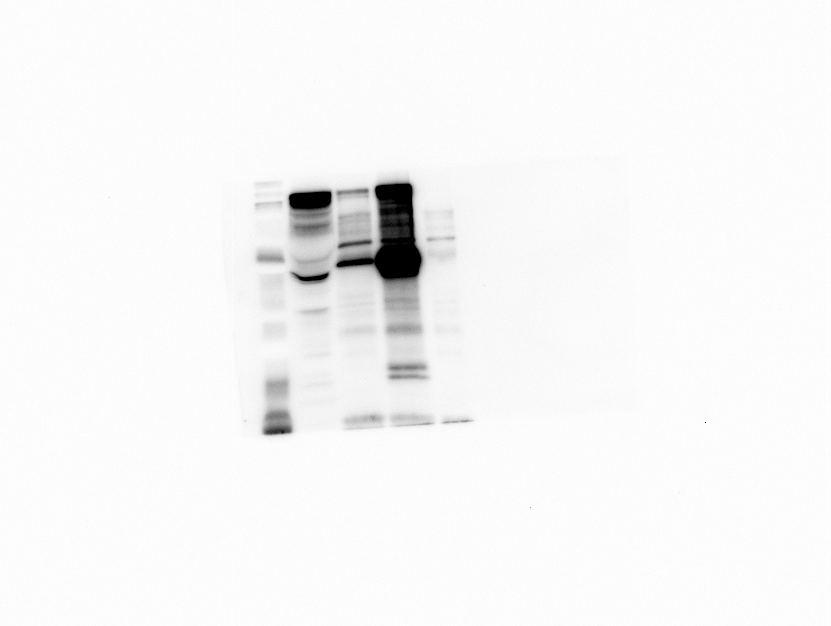

Supplement: Supplementary file 13 — Figure EV1-4 Source Data [file 44318_2024_281_MOESM13_ESM.zip › Figure EV3/EV3D/western ACE2.png]

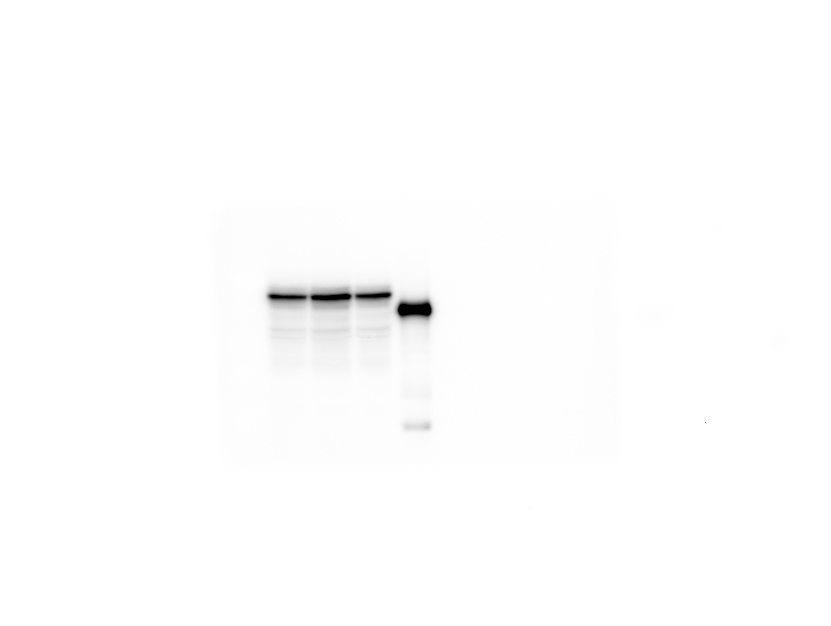

Supplement: Supplementary file 13 — Figure EV1-4 Source Data [file 44318_2024_281_MOESM13_ESM.zip › Figure EV3/EV3D/western LSR.png]

kDa

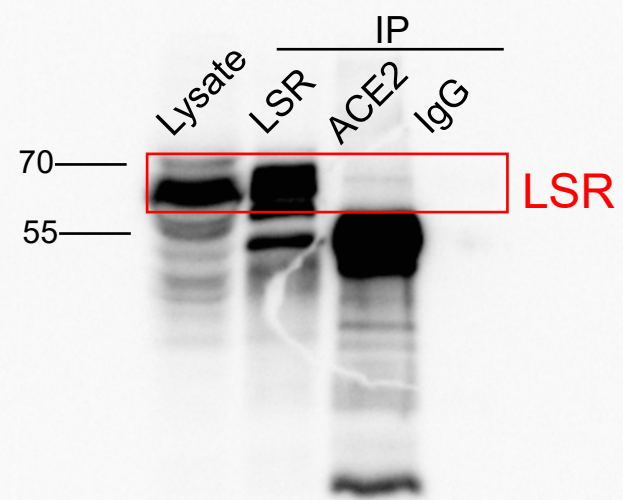

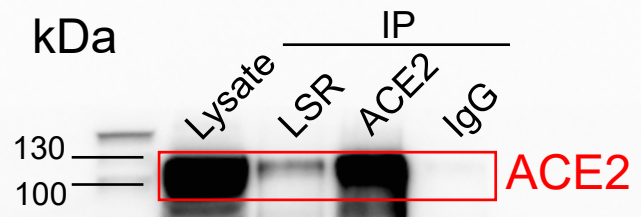

Supplement: Supplementary file 13 — Figure EV1-4 Source Data [file 44318_2024_281_MOESM13_ESM.zip › Figure EV3/EV3E/EV3E.pdf]

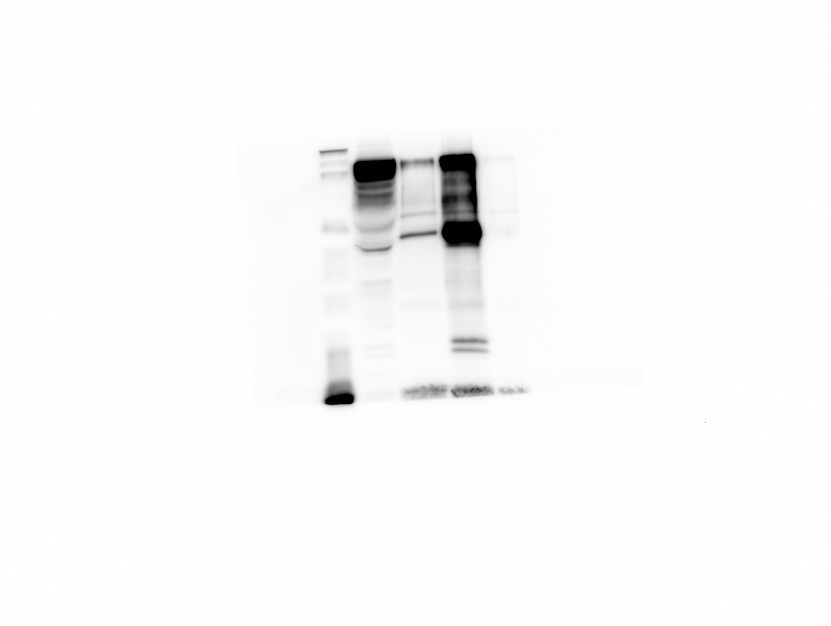

Supplement: Supplementary file 13 — Figure EV1-4 Source Data [file 44318_2024_281_MOESM13_ESM.zip › Figure EV3/EV3E/western ACE2.png]

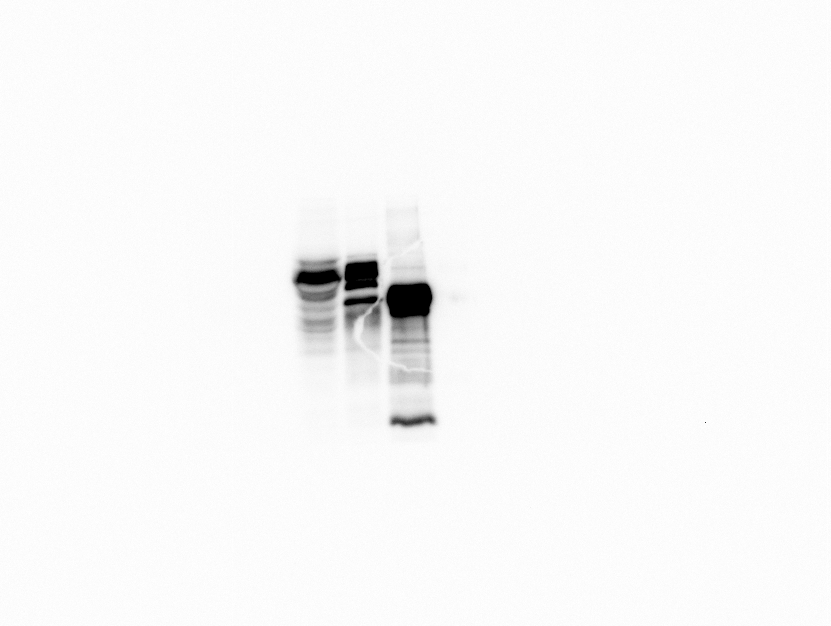

Supplement: Supplementary file 13 — Figure EV1-4 Source Data [file 44318_2024_281_MOESM13_ESM.zip › Figure EV3/EV3E/western LSR.png]

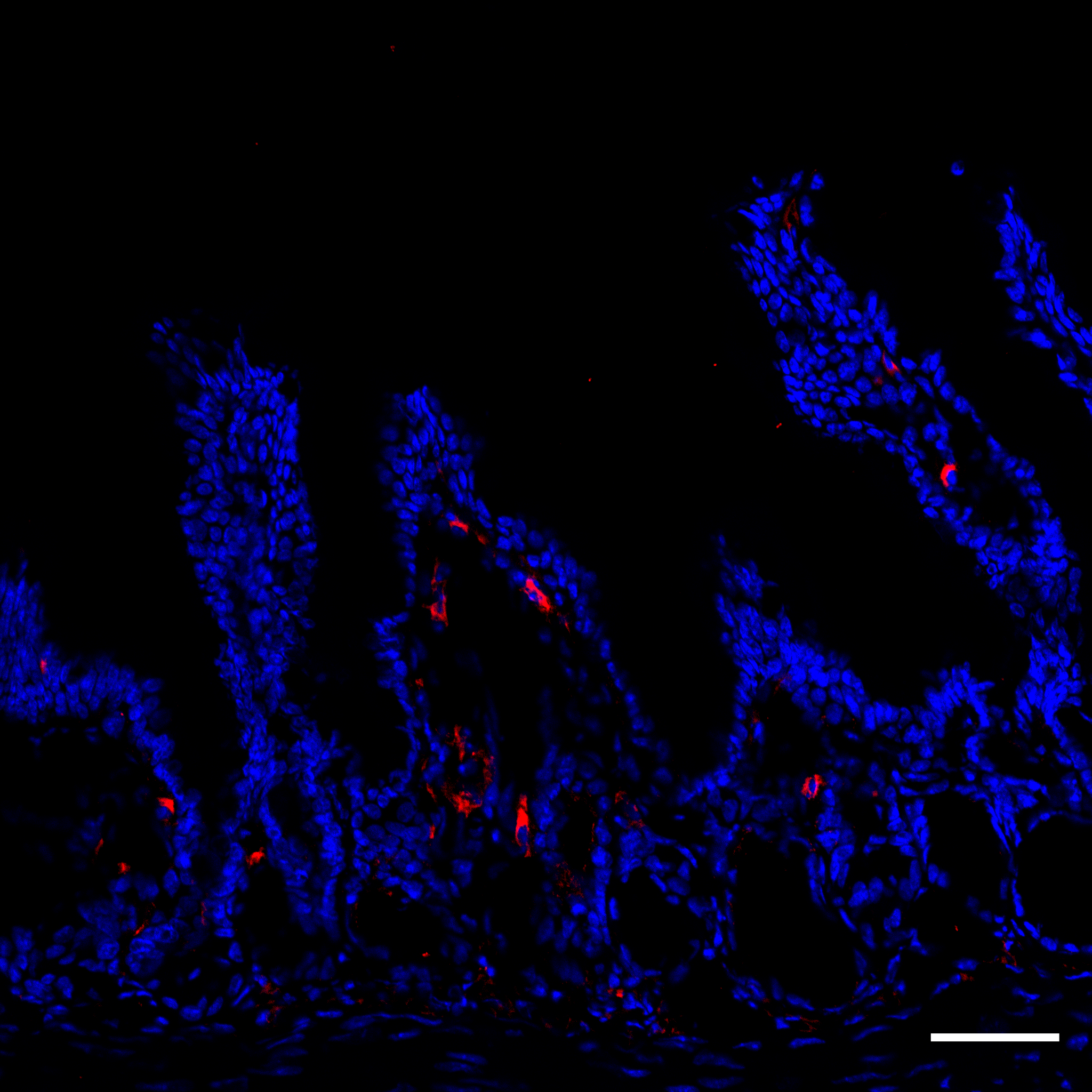

Supplement: Supplementary file 13 — Figure EV1-4 Source Data [file 44318_2024_281_MOESM13_ESM.zip › Figure EV4/EV4G/IF LY6G Duodenum VSV-SARS-CoV-2+CRD1.tif]

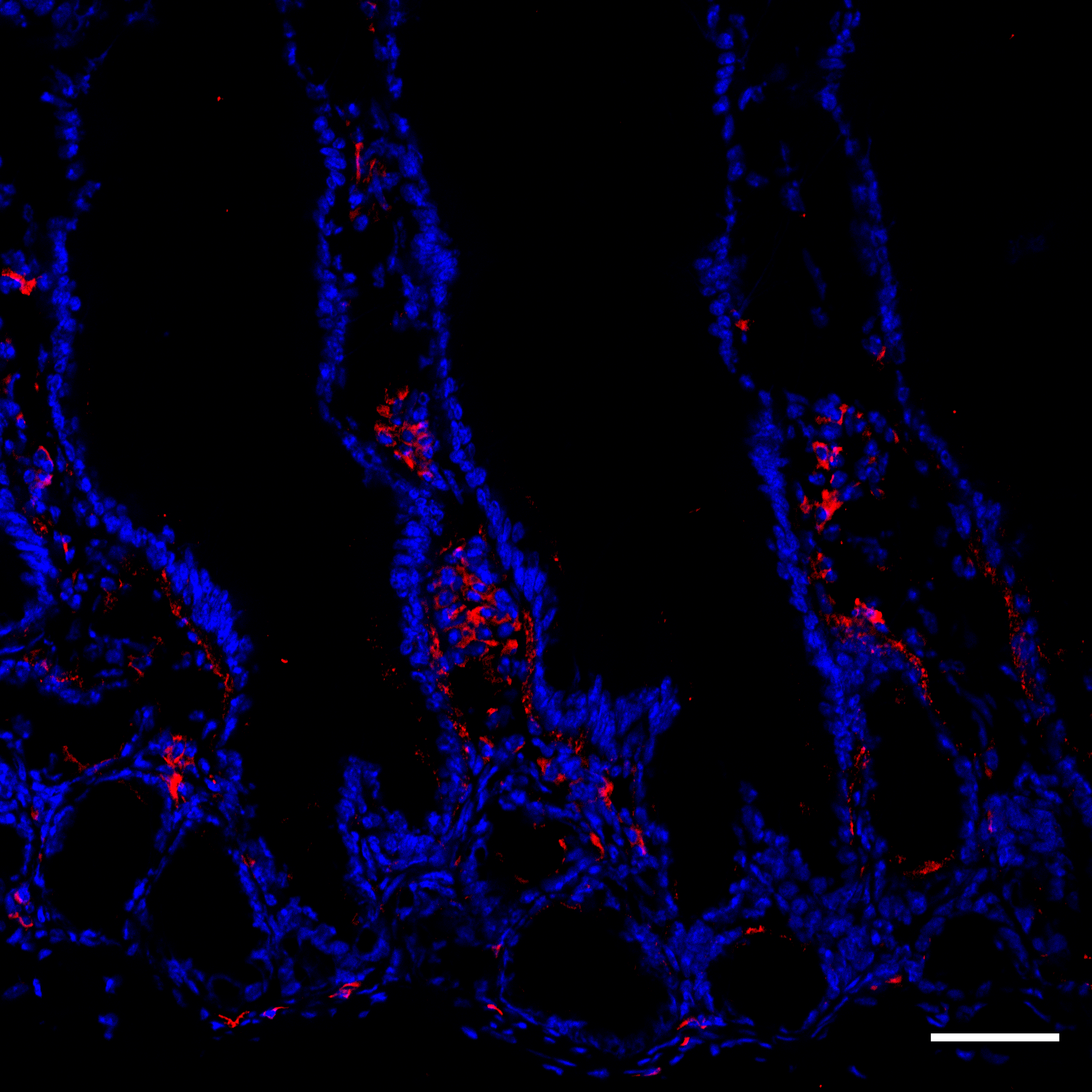

Supplement: Supplementary file 13 — Figure EV1-4 Source Data [file 44318_2024_281_MOESM13_ESM.zip › Figure EV4/EV4G/IF LY6G Duodenum VSV-SARS-CoV-2.tif]

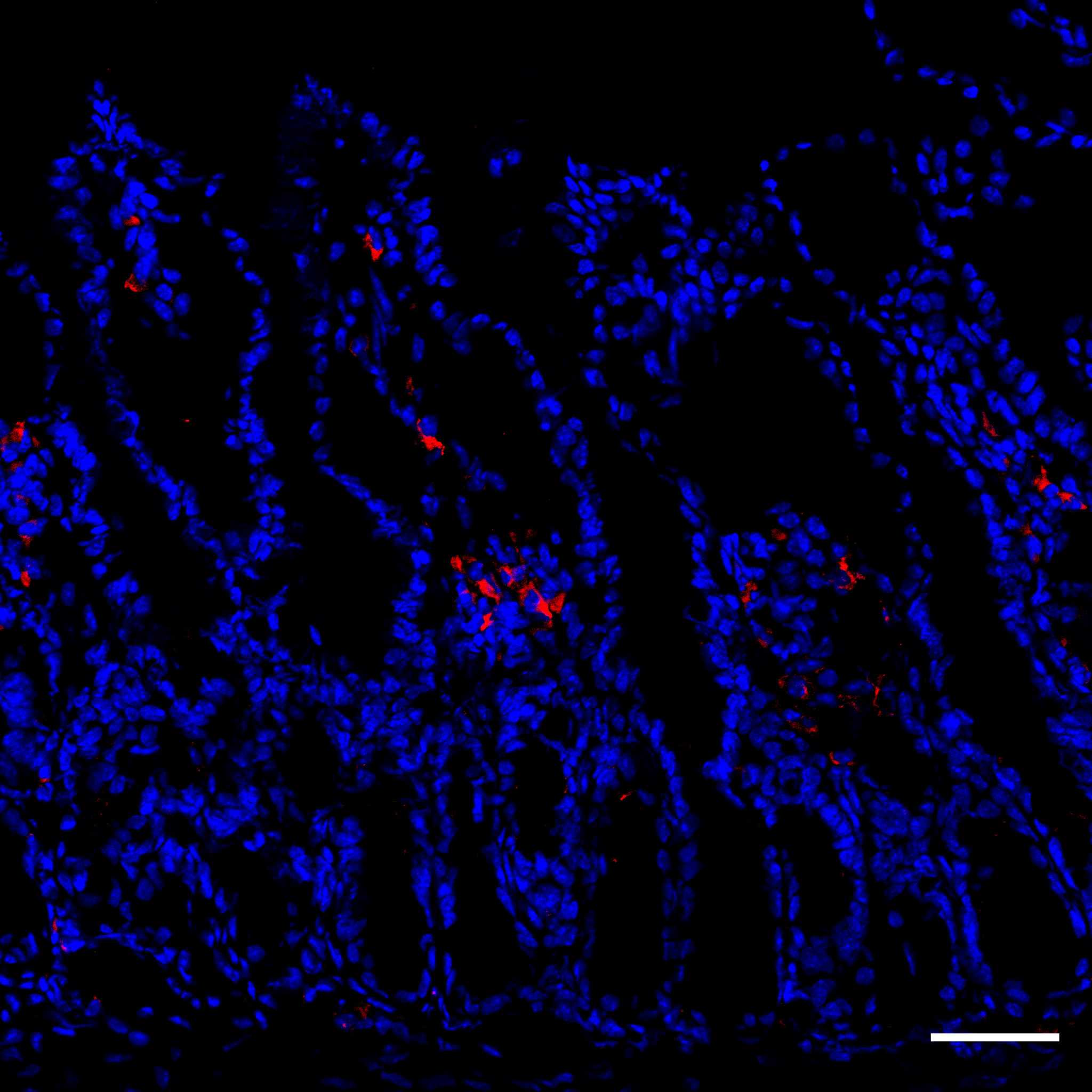

Supplement: Supplementary file 13 — Figure EV1-4 Source Data [file 44318_2024_281_MOESM13_ESM.zip › Figure EV4/EV4G/IF LY6G Duodenum control.tif]

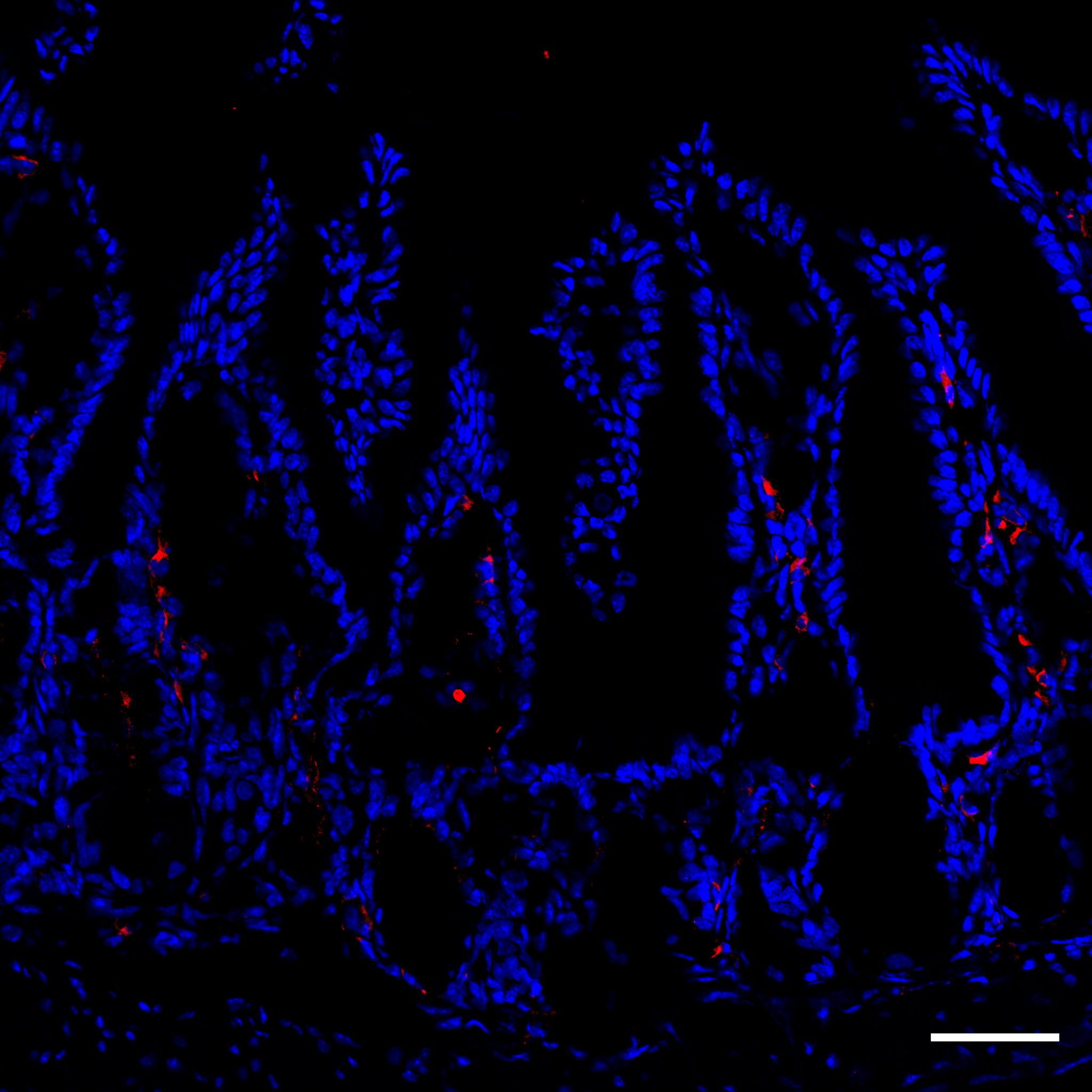

Supplement: Supplementary file 13 — Figure EV1-4 Source Data [file 44318_2024_281_MOESM13_ESM.zip › Figure EV4/EV4G/IF LY6G Ileum VSV-SARS-CoV-2+CRD1.tif]

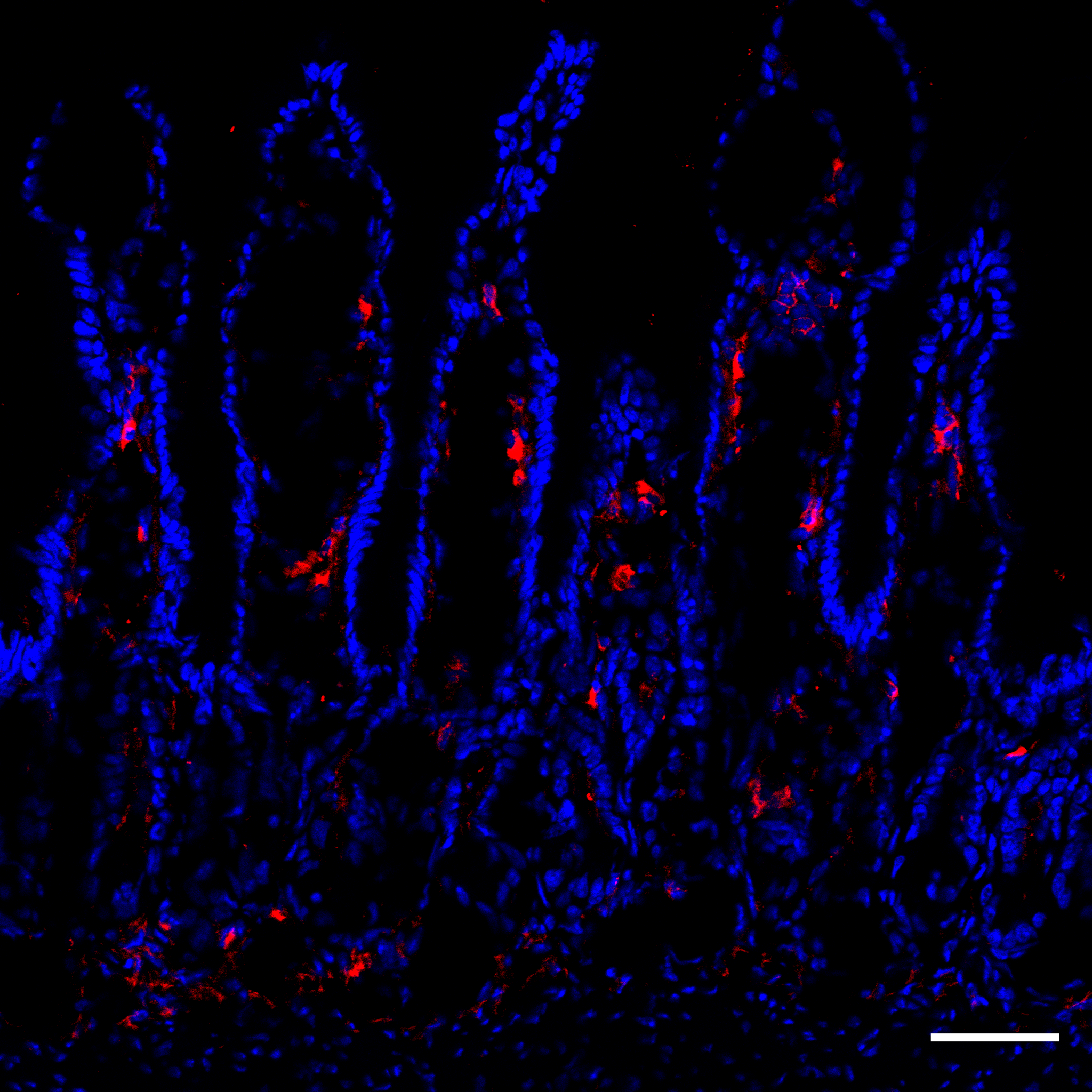

Supplement: Supplementary file 13 — Figure EV1-4 Source Data [file 44318_2024_281_MOESM13_ESM.zip › Figure EV4/EV4G/IF LY6G Ileum VSV-SARS-CoV-2.tif]

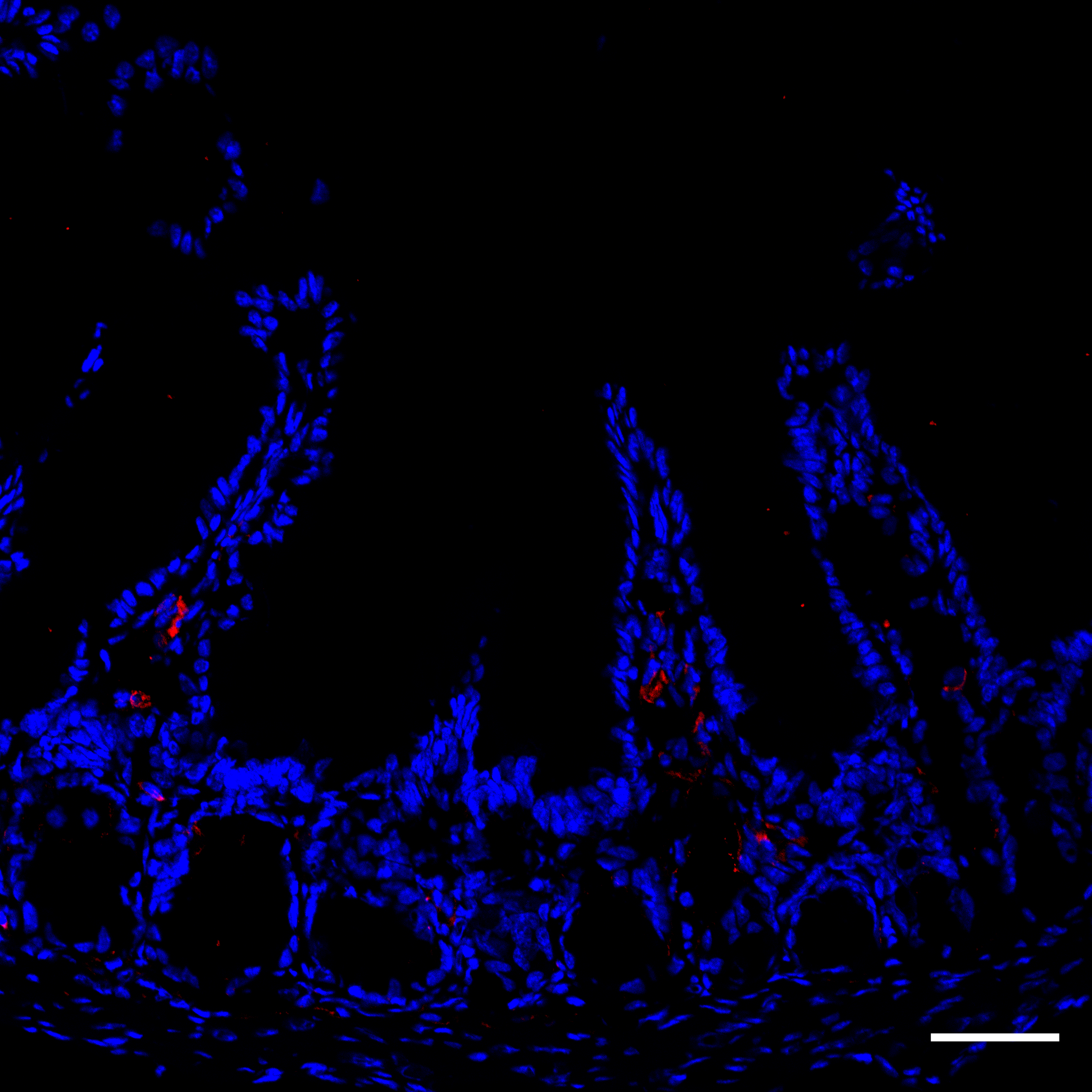

Supplement: Supplementary file 13 — Figure EV1-4 Source Data [file 44318_2024_281_MOESM13_ESM.zip › Figure EV4/EV4G/IF LY6G Ileum control.tif]

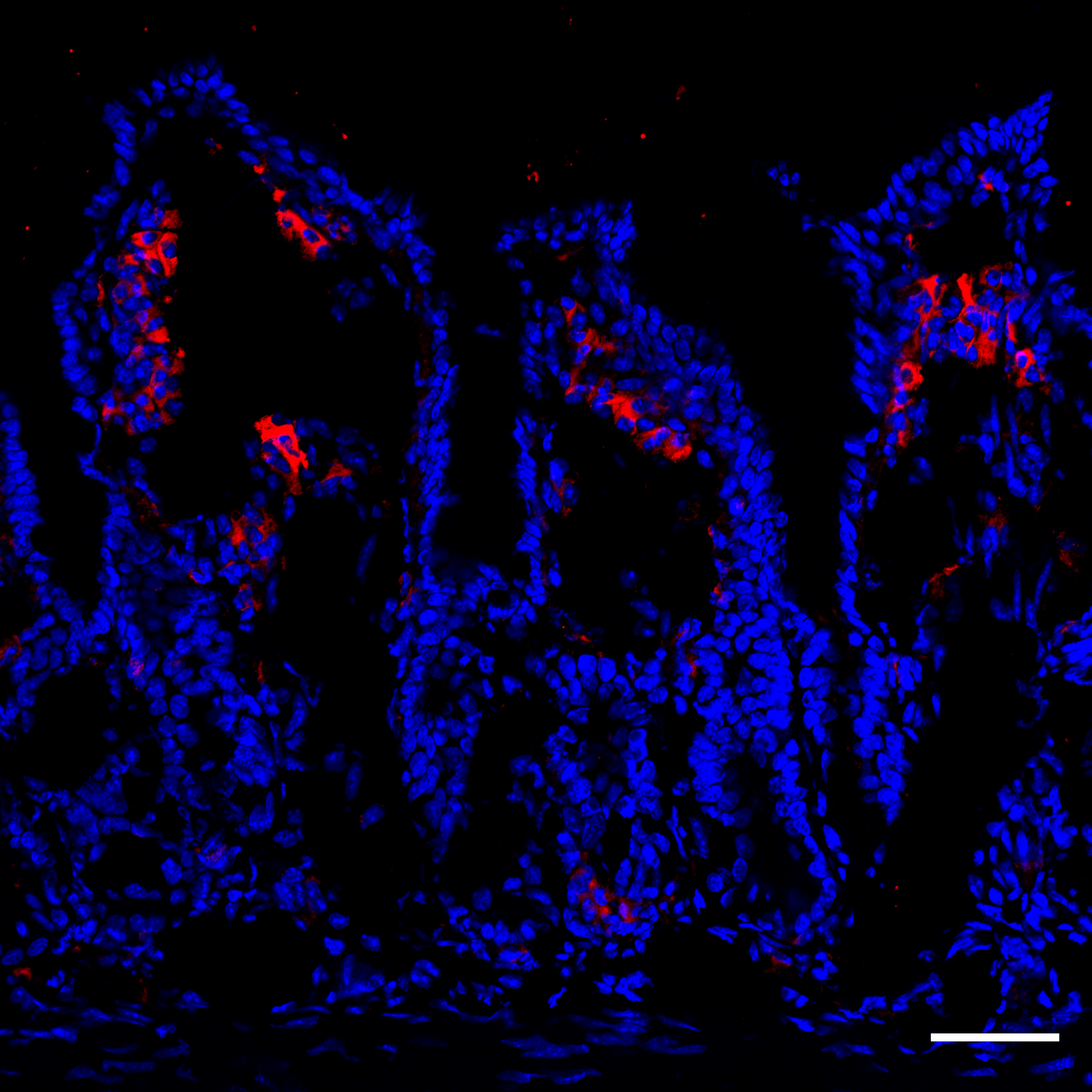

Supplement: Supplementary file 13 — Figure EV1-4 Source Data [file 44318_2024_281_MOESM13_ESM.zip › Figure EV4/EV4G/IF LY6G Jejunum VSV-SARS-CoV-2+CRD1.tif]

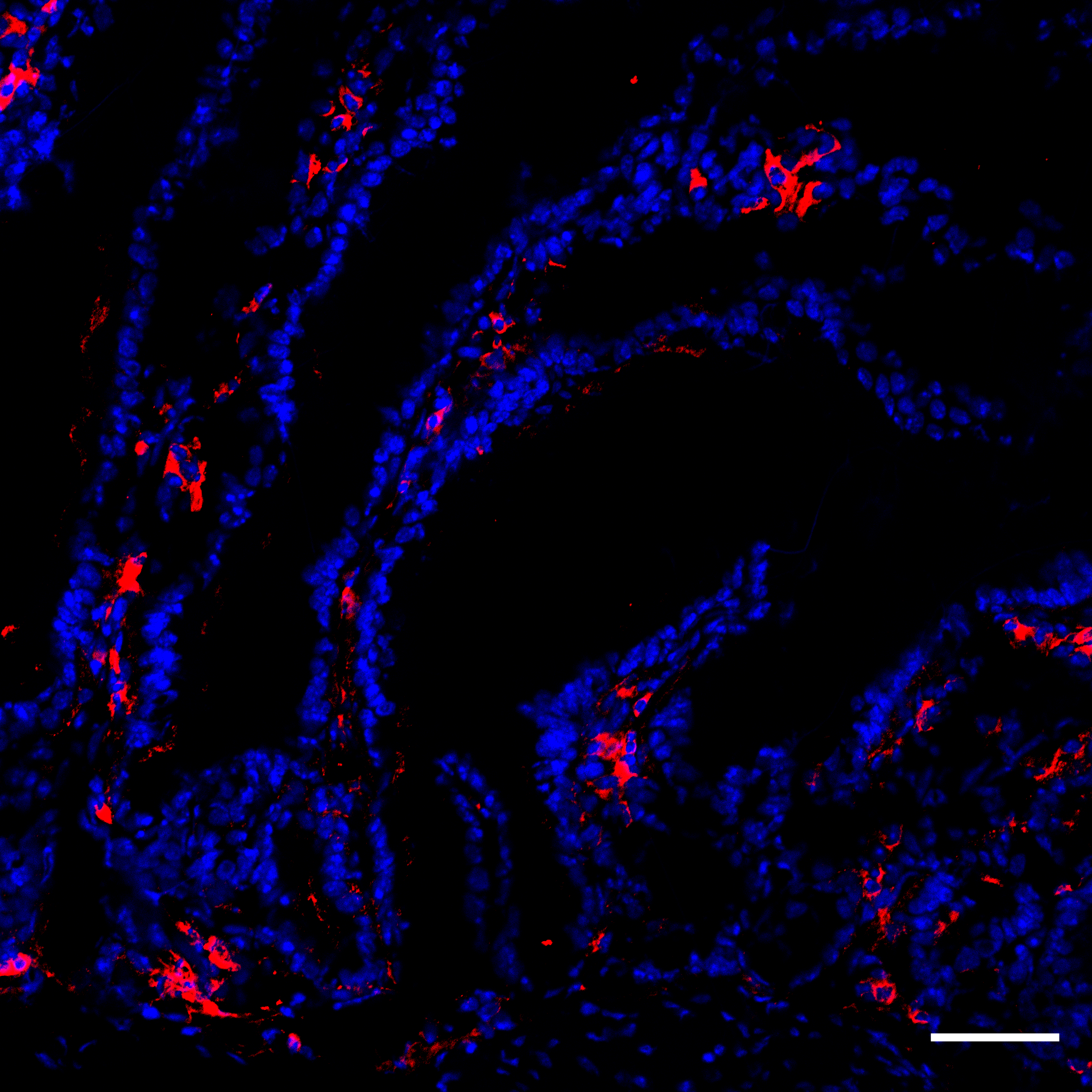

Supplement: Supplementary file 13 — Figure EV1-4 Source Data [file 44318_2024_281_MOESM13_ESM.zip › Figure EV4/EV4G/IF LY6G Jejunum VSV-SARS-CoV-2.tif]

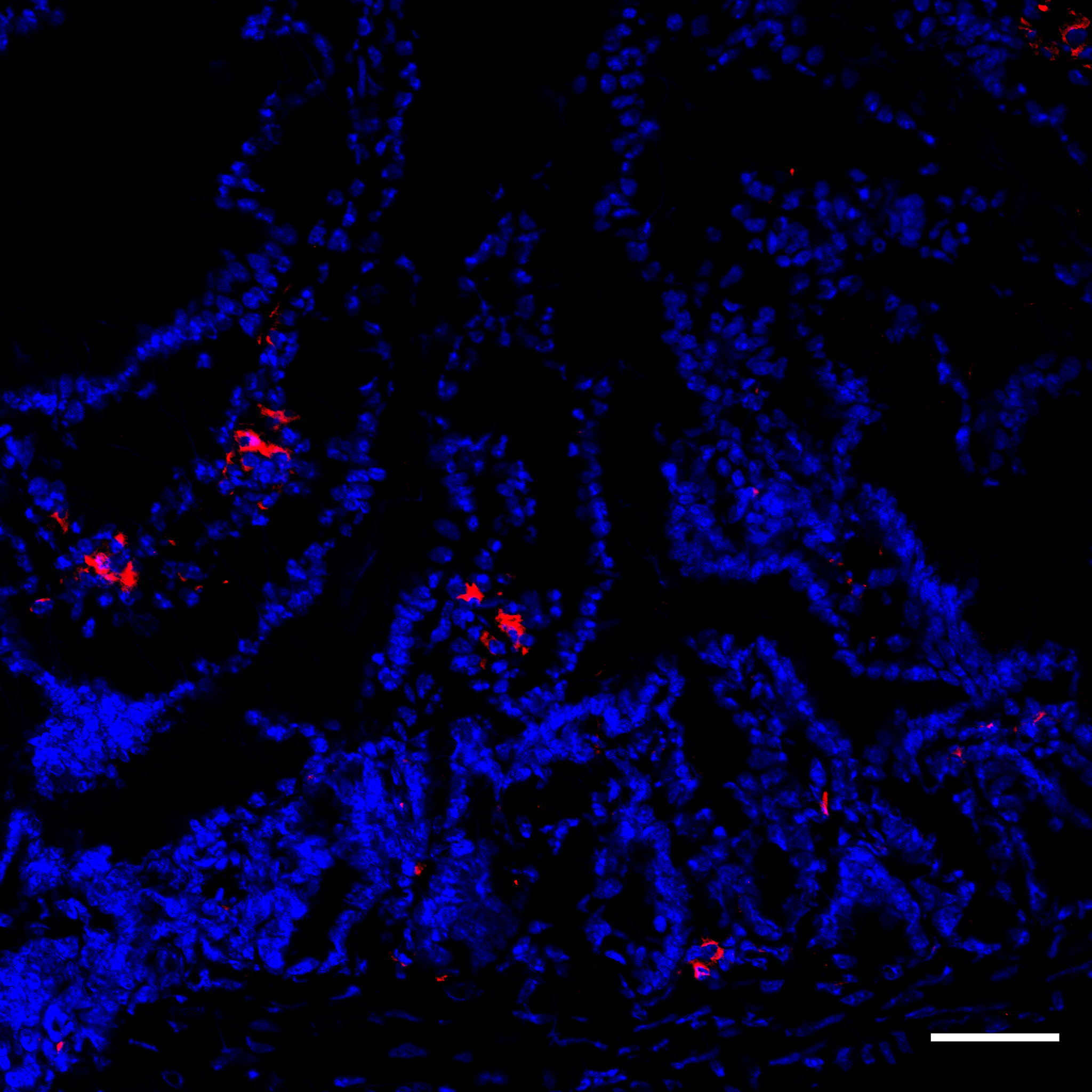

Supplement: Supplementary file 13 — Figure EV1-4 Source Data [file 44318_2024_281_MOESM13_ESM.zip › Figure EV4/EV4G/IF LY6G Jejunum control.tif]
