# Supplementary material for: Tight junction protein LSR is a host defense factor against SARS-CoV-2 infection in the small intestine
Source: EMBO J. 2024 Oct 23;43(23):6124–51. doi: 10.1038/s44318-024-00281-4 (PMC11612383; doi:10.1038/s44318-024-00281-4)
Supplement: Supplementary file 14 — Expanded View Figures [file 44318_2024_281_MOESM14_ESM.pdf]

Expanded View Figures

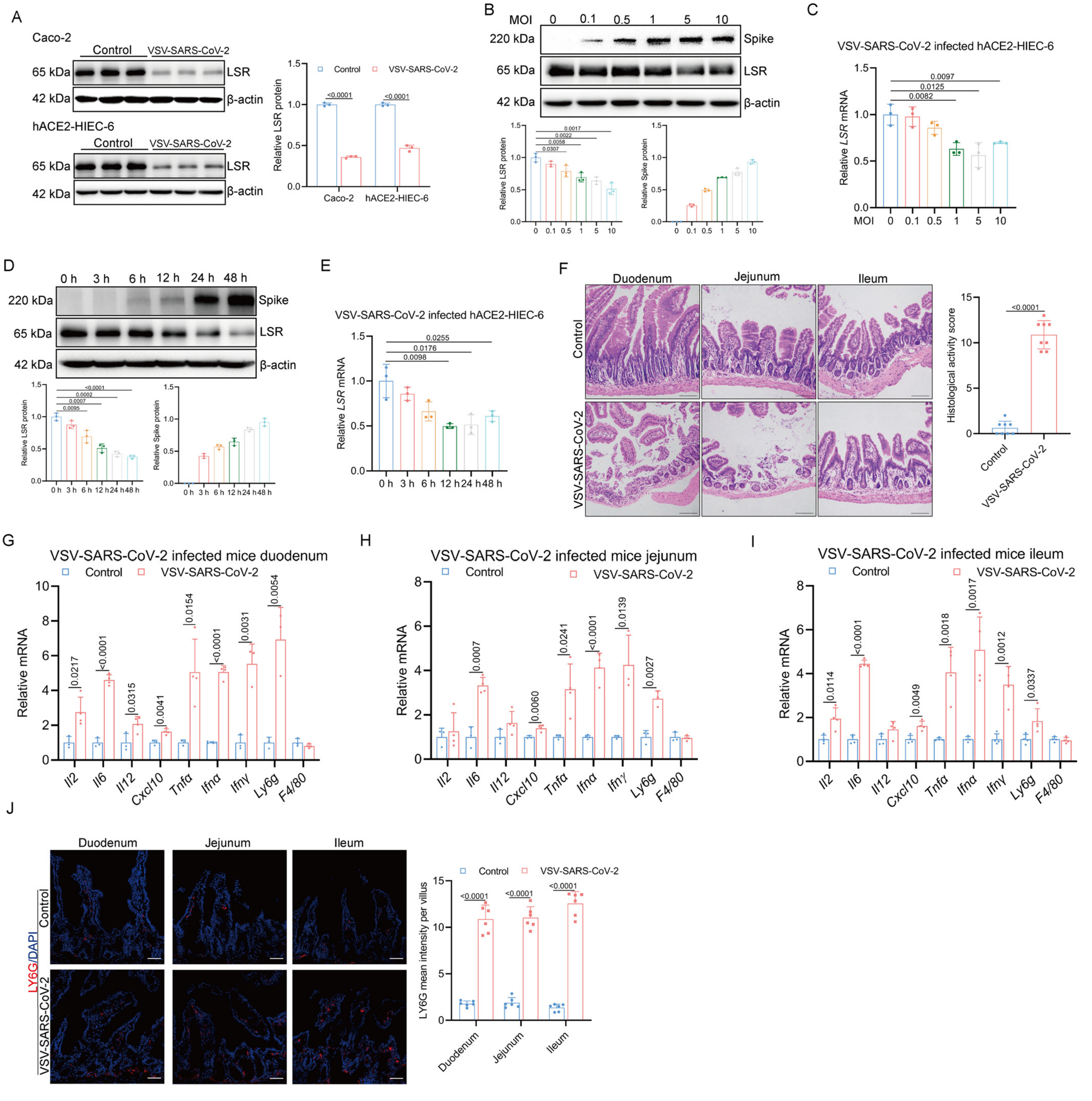

Figure EV1. SARS-CoV-2 infection alters the expression of LSR in intestine.

(**A**) Western blot and densitometric analysis of LSR in Caco-2 and hACE2-HIEC-6 cells with and without VSV-SARS-CoV-2 infection at 1 MOI for 24 h ($n = 3$). (**B**, **C**) Western blot and densitometric analysis of Spike and LSR (**B**), and expression of *LSR* mRNA analyzed by RT-qPCR (**C**) in hACE2-HIEC-6 cells infected with VSV-SARS-CoV-2 at the indicated MOI for 24 h ($n = 3$). (**D**, **E**) Western blot and densitometric analysis of Spike and LSR (**D**), and expression of *LSR* mRNA analyzed by RT-qPCR (**E**) in hACE2-HIEC-6 cells infected with VSV-SARS-CoV-2 at 1 MOI for the indicated time points ($n = 3$). (**F**) H&E staining and histological activity score in small intestines from hACE2-WT mice with and without VSV-SARS-CoV-2 infection ($n = 6$). (**G–I**) mRNA expression of *Il2, Il6, Il12, Cxcl10, Tnfa, Ifna, Ifnγ, Ly6g*, and *F4/80* analyzed by RT-qPCR in duodenal (**G**), jejunal (**H**), and ileal (**I**) segments from humanized ACE2 mice with and without VSV-SARS-CoV-2 infection ($n = 4$). (**J**) Immunofluorescence staining images and quantitative analysis of LY6G in small intestines from humanized ACE2 mice with and without VSV-SARS-CoV-2 infection ($n = 6$). "*n*" represents number of biological replicates. Scale bars: (**F**) and (**J**), 50 μm. Data represent mean ± SEM. Unpaired t test was performed. $p < 0.05$, the exact *p*-value is displayed; $p > 0.05$, the *p*-value is not displayed. Source data are available online for this figure.

© The Author(s)

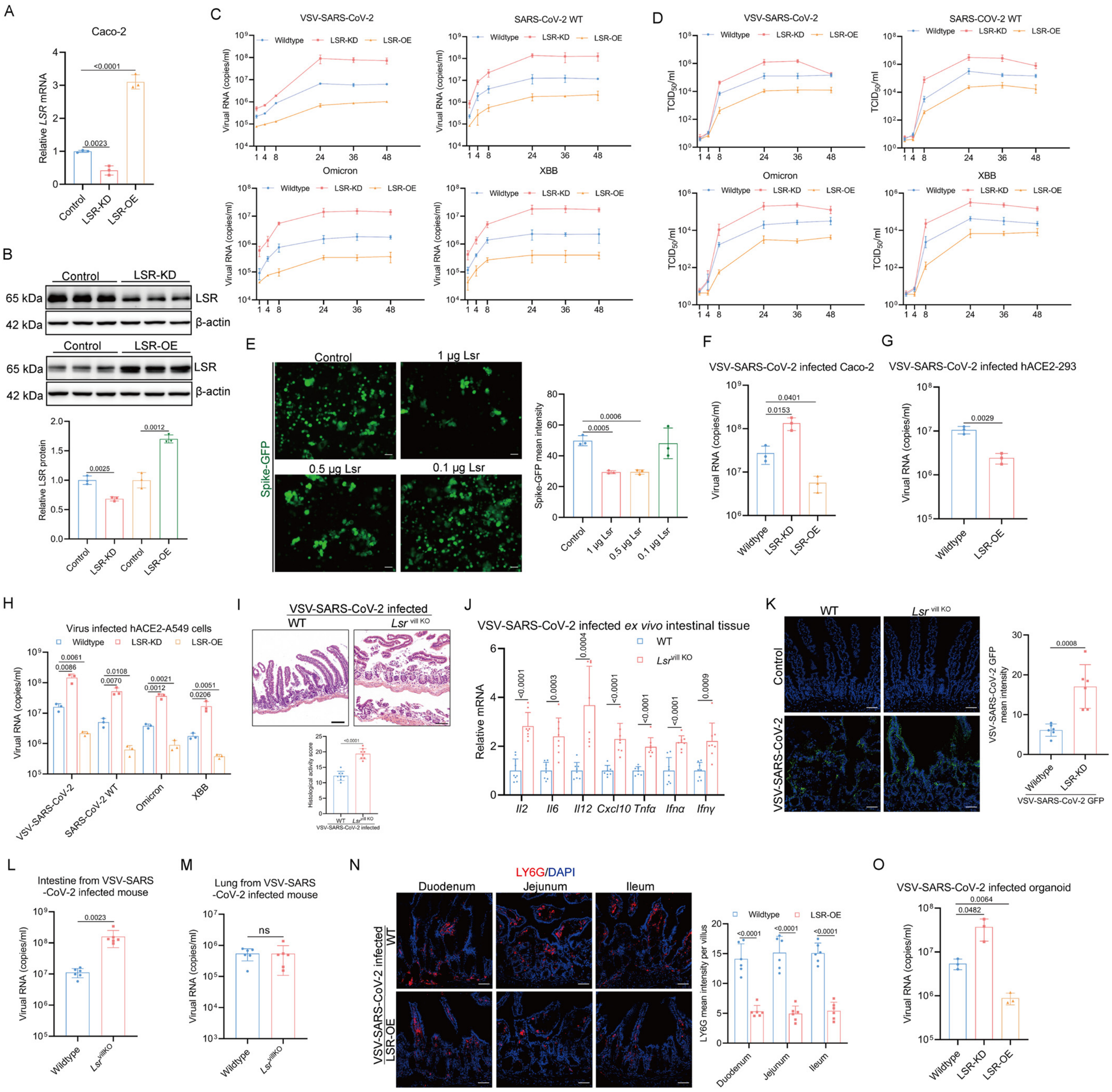
A
Caco-2
Relative LSR mRNA
Control
LSR-KD
LSR-OE
B
Control
LSR-KD
LSR-OE
65 kDa
42 kDa
LSR
β-actin
Relative LSR protein
C
VSV-SARS-CoV-2
SARS-CoV-2 WT
Omicron
XBB
Wildtype
Virual RNA (copies/ml)
D
SARS-COV-2 WT
TCID50/ml
E
Spike-GFP
1 μg Lsr
0.5 μg Lsr
0.1 μg Lsr
Spike-GFP mean intensity
F
VSV-SARS-CoV-2 infected Caco-2
G
VSV-SARS-CoV-2 infected hACE2-293
H
Virus infected hACE2-A549 cells
I
VSV-SARS-CoV-2 infected
WT
Lsr vill KO
Histological activity score
J
VSV-SARS-CoV-2 infected ex vivo intestinal tissue
Relative mRNA
Il2
Il6
Il12
Cxcl10
Tnfa
Ifna
Ifnγ
K
VSV-SARS-CoV-2 GFP mean intensity
VSV-SARS-CoV-2 GFP
L
Intestine from VSV-SARS -CoV-2 infected mouse
M
Lung from VSV-SARS -CoV-2 infected mouse
N
LY6G/DAPI
Duodenum
Jejunum
Ileum
VSV-SARS-CoV-2 infected
LY6G mean intensity per villus
O
VSV-SARS-CoV-2 infected organoid

 © The Author(s)

Figure EV2. LSR acts as a host defense factor against SARS-CoV-2 in intestine.

(**A**, **B**) Expression of *LSR* mRNA analyzed by RT-qPCR (**A**), and western blot and densitometric analysis of LSR (**B**) in Caco-2 cells transduced with retrovirus containing LSR shRNA, human LSR transcript variant 2, or control vector ($n = 3$). (**C**) Viral RNA copies analyzed by RT-qPCR using primers targeting SARS-CoV-2 N gene in Caco-2 cells infected with VSV-SARS-CoV-2 and SARS-CoV-2 WT, Omicron, and XBB at 1 MOI for 1, 4, 8, 24, 36, and 48 h ($n = 3$). (**D**) Viral production assessed by $TCID_{50}$ assay on naïve Vero cells treated with supernatants from infected Caco-2 cells collected during the time course in (**C**) ($n = 5$). (**E**) Fluorescence images and quantitative analysis of Caco-2 cells transfected with control vector, or indicated amounts of plasmids containing mouse Lsr, and infected with VSV-SARS-CoV-2 at 1 MOI for 24 h ($n = 3$). (**F**) Viral RNA copies analyzed by RT-qPCR using primers targeting Spike gene in wild type, LSR-KD, and LSR-OE Caco-2 cells infected with VSV-SARS-CoV-2 at 1 MOI for 24 h ($n = 3$). (**G**) Viral RNA copies analyzed by RT-qPCR using primers targeting VSV-P in hACE2-293 cells infected with VSV-SARS-CoV-2 at 1 MOI for 24 h ($n = 3$). (**H**) Viral RNA copies analyzed by RT-qPCR using primers targeting VSV-P or SARS-CoV-2 N gene in wild type, LSR-KD, and LSR-OE hACE2-A549 cells infected with VSV-SARS-CoV-2 and SARS-CoV-2 WT, Omicron, and XBB at 1 MOI for 24 h ($n = 3$). (**I**, **J**) H&E staining and histological activity score (**I**), and expression of *Il2, Il6, Il12, Cxcl10, Tnfa, Ifna*, and *Ifnγ* mRNA analyzed by RT-qPCR (**J**) in VSV-SARS-CoV-2 infected ex vivo intestinal tissues from hACE2-WT and hACE2-$Lsr^{\text{vill KO}}$ mice ($n = 8$). (**K**) Fluorescence images and quantitative analysis of GFP in VSV-SARS-CoV-2 infected ex vivo intestinal tissues harvested from hACE2-WT and hACE2-$Lsr^{\text{vill KO}}$ mice ($n = 6$). (**L**, **M**) Viral RNA copies analyzed by RT-qPCR using primers targeting VSV-P in intestines (**L**) and lungs (**M**) from VSV-SARS-CoV-2 infected hACE2-WT and hACE2-$Lsr^{\text{vill KO}}$ mice ($n = 6$). (**N**) Immunofluorescence staining images and quantitative analysis of LY6G in small intestines from K18-hACE2-WT (WT) and K18-hACE2-LSR-OE (LSR-OE) mice intraperitoneally infected with 100 μl VSV-SARS-CoV-2 (2×10^8 p.f.u./ml) ($n = 6$). (**O**) Viral RNA copies analyzed by RT-qPCR using primers targeting VSV-P in wild type, LSR-KD, and LSR-OE intestinal organoid infected with VSV-SARS-CoV-2 ($n = 3$). "*n*" represents number of biological replicates. Scale bars: (**E**), 100 μm; (**I**), (**K**), and (**N**), 50 μm. Data represent mean ± SEM. Unpaired t test was performed. $p < 0.05$, the exact *p*-value is displayed; $p > 0.05$, the *p*-value is not displayed. Source data are available online for this figure.

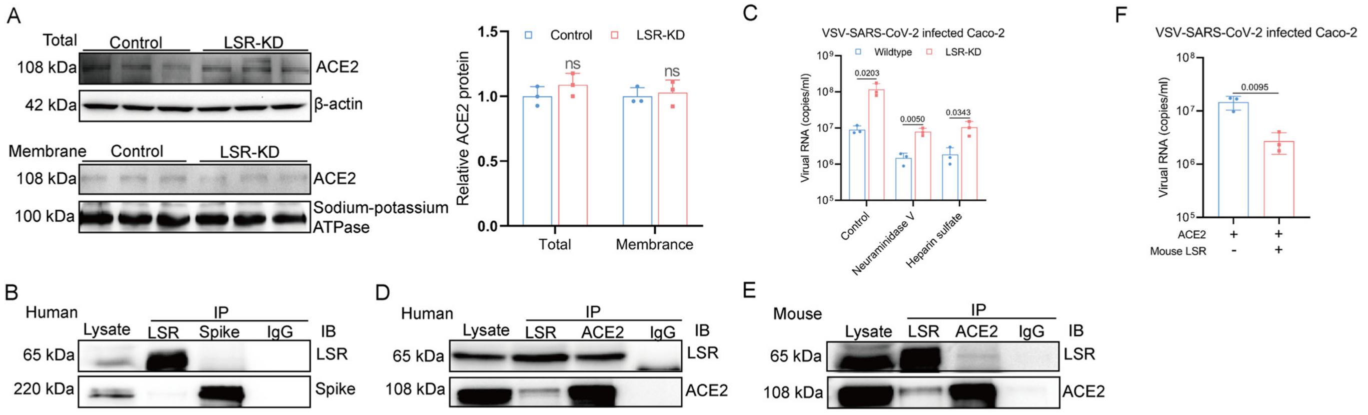

Figure EV3. LSR alters interactions between Spike and ACE2.

(**A**) Western blot and densitometric analysis of total ACE2 and membrane ACE2 in wild type and LSR-KD Caco-2 cells ($n = 3$). (**B**) Co-IP showing that human LSR cannot interact with Spike. (**C**) Viral RNA copies analyzed by RT-qPCR using primers targeting VSV-P in Caco-2 cells treated with DMSO, 200 mU/ml neuraminidase V, or 300 µg/ml heparin sulfate at 37 °C for 1 h, then infected with VSV-SARS-CoV-2 at 1 MOI at 4 °C for 1 h ($n = 3$). (**D**) Co-IP showing that human LSR interacts with human ACE2 in HEK293 cells. (**E**) Co-IP showing that mouse LSR interacts with human ACE2. (**F**) Viral RNA copies analyzed by RT-qPCR using primers targeting VSV-P in Caco-2 cells transfected with human ACE2 and mouse LSR expressing plasmids simultaneously or human ACE2 expressing plasmid alone, and infected with VSV-SARS-CoV-2 at 1 MOI for 24 h ($n = 3$). All the Co-IP assays were performed in doubly transfected HEK293 cells. IB, immunoblot; IP, immunoprecipitation. "*n*" represents number of biological replicates. Data represent mean ± SEM. Unpaired t test was performed. $p < 0.05$, the exact p-value is displayed; $p > 0.05$, the p-value is not displayed. Source data are available online for this figure.

 © The Author(s)

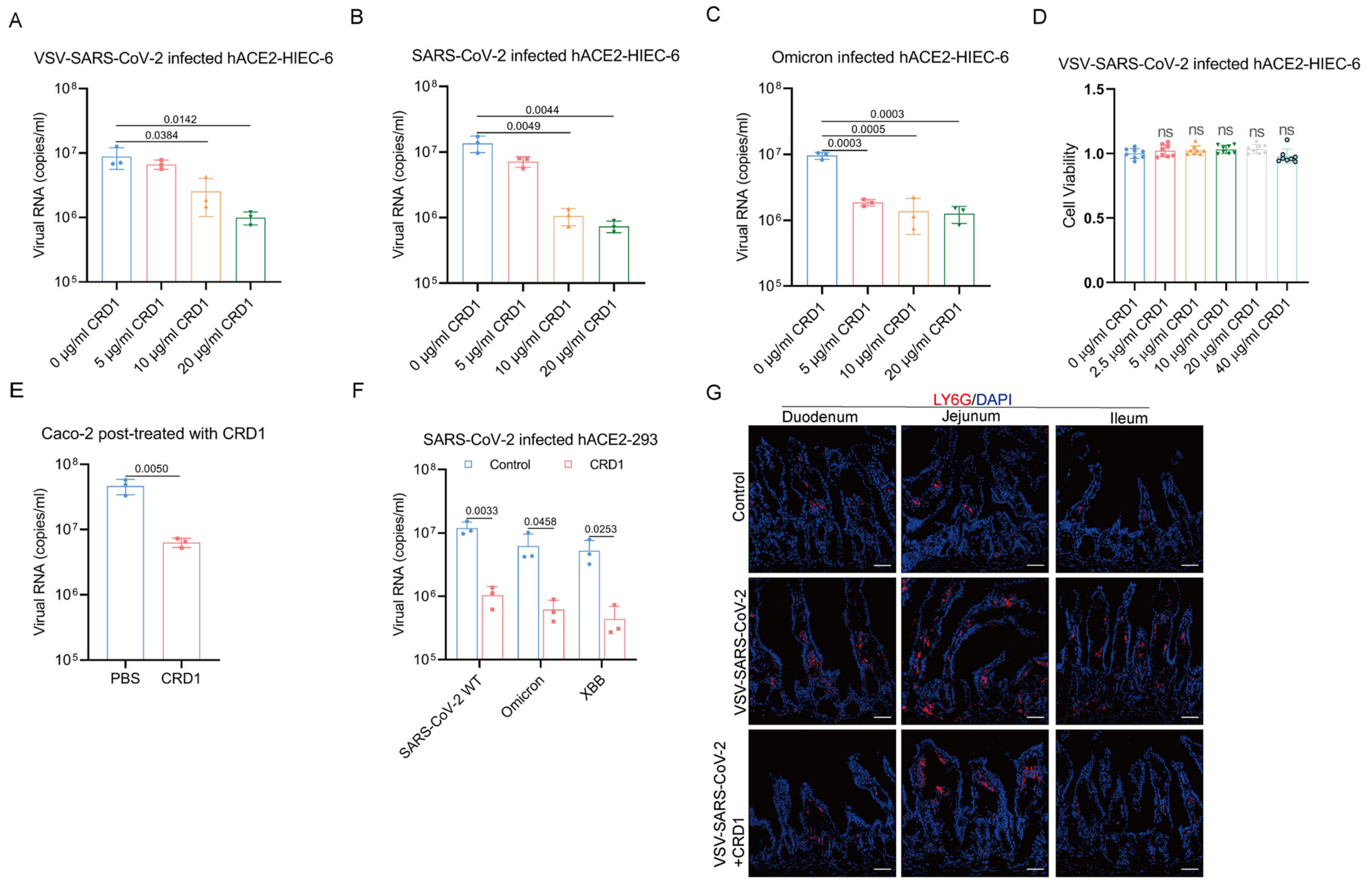

Figure EV4. LSR-derived CRD1 peptide inhibits infection of SARS-CoV-2.

(**A**) Viral RNA copies analyzed by RT-qPCR using primers targeting VSV-P in hACE2-HIEC-6 cells treated with indicated concentrations of CRD1 and infected with VSV-SARS-CoV-2 ($n = 3$). (**B**, **C**) Viral RNA copies analyzed by RT-qPCR using primers targeting SARS-CoV-2 N gene in hACE2-HIEC-6 cells treated with indicated concentrations of CRD1 and infected with SARS-CoV-2 WT (**B**) and Omicron (**C**) ($n = 3$). (**D**) Cell viability determined by CCK8 assay in hACE2-HIEC-6 cells treated with indicated concentrations of CRD1 ($n = 8$). (**E**) Viral RNA copies analyzed by RT-qPCR using primers targeting VSV-P in Caco-2 cells infected with VSV-SARS-CoV-2 at 1 MOI at 4 °C for 1 h, and treated with PBS or 10 μg/ml CRD1 for 24 h ($n = 3$). (**F**) Viral RNA copies analyzed by RT-qPCR using primers targeting SARS-CoV-2 N gene in hACE2-293 cells treated with PBS or 10 μg/ml CRD1 before being infected with SARS-CoV-2 WT, Omicron, and XBB at 1 MOI for 24 h ($n = 3$). (**G**) Immunofluorescence images of LY6G in small intestines from control and VSV-SARS-CoV-2 infected K18-hACE2 mice with and without CRD1 treatment before infection. "*n*" represents number of biological replicates. Scale bars: (**G**), 50 μm. Data represent mean ± SEM. Unpaired t test was performed. $p < 0.05$, the exact *p*-value is displayed; $p > 0.05$, the *p*-value is not displayed. Source data are available online for this figure.

© The Author(s)

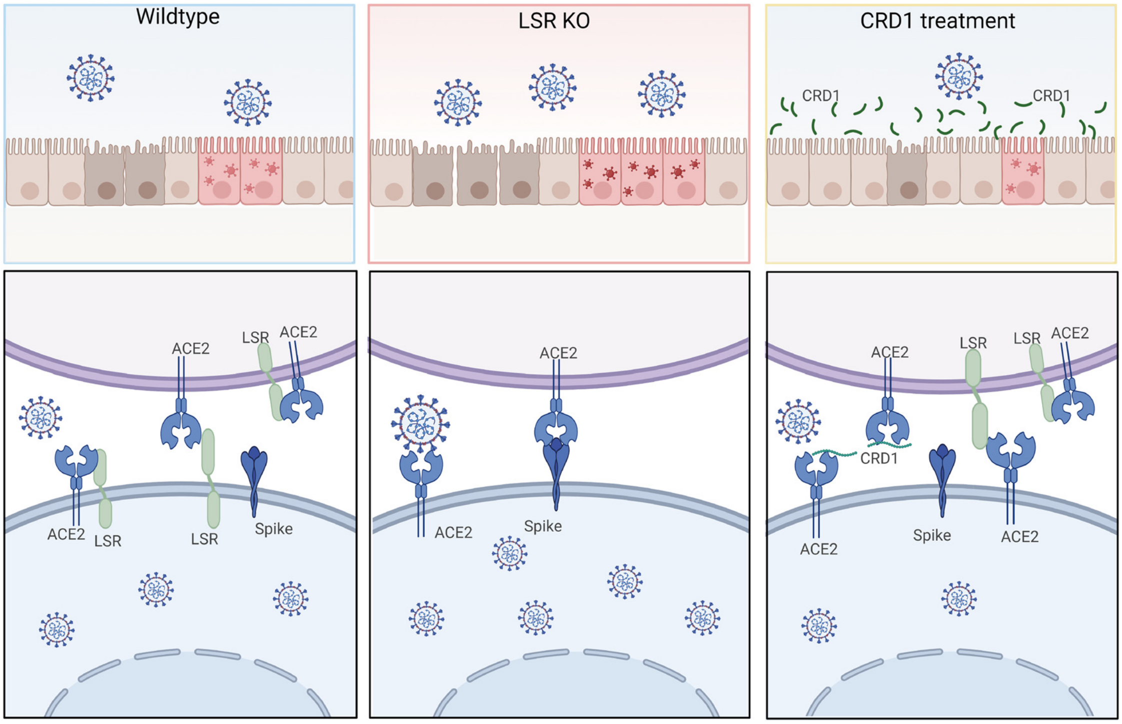

Figure EV5. Summary.

LSR interacts with ACE2 both in *cis* and in *trans* through its extracellular C-terminal region, preventing ACE2 binding to Spike protein, and thus blocking viral entry and inhibiting Spike-mediated cell–cell fusion. LSR knockout promotes SARS-CoV-2 infection in the small intestine, while LSR-derived peptide CRD1 blocks Spike binding with ACE2 to protect against SARS-CoV-2 infection. The graphic was created with BioRender.com.

 © The Author(s)
